# Enantioselective Desymmetrization of Prochiral Cyclohexanones by Organocatalytic Intramolecular Michael Additions to α,β-Unsaturated Esters**

**DOI:** 10.1002/anie.201411924

**Published:** 2015-02-27

**Authors:** Adam D Gammack Yamagata, Swarup Datta, Kelvin E Jackson, Linus Stegbauer, Robert S Paton, Darren J Dixon

**Affiliations:** Department of Chemistry, Chemistry Research Laboratory University of Oxford, Mansfield Road, Oxford, OX1 3TA (UK)

**Keywords:** desymmetrization, enamine catalysis, Michael addition, organocatalysis, quantum-chemical calculations

## Abstract

A new catalytic asymmetric desymmetrization reaction for the synthesis of enantioenriched derivatives of 2-azabicyclo[3.3.1]nonane, a key motif common to many alkaloids, has been developed. Employing a cyclohexanediamine-derived primary amine organocatalyst, a range of prochiral cyclohexanone derivatives possessing an α,β-unsaturated ester moiety linked to the 4-position afforded the bicyclic products, which possess three stereogenic centers, as single diastereoisomers in high enantioselectivity (83–99 % *ee*) and in good yields (60–90 %). Calculations revealed that stepwise C–C bond formation and proton transfer via a chair-shaped transition state dictate the exclusive *endo* selectivity and enabled the development of a highly enantioselective primary amine catalyst.

The morphan structural motif (2-azabicyclo[3.3.1]nonane) is common to many biologically relevant alkaloid natural products. This core subunit is found within over 300 natural products, including the strychnos, daphniphyllum, and madangamine families.^1^ Furthermore, it is present in many other biologically relevant molecules, such as the immunosuppressant FR901483, the cytotoxic agent aspernomine, and the analgesic morphine.1a

As part of our research program directed towards the synthesis of various alkaloid natural products, including daphniphyllum and manzamine^2^ targets, we sought to develop a new catalytic asymmetric method to access the morphan motif with high efficiency and selectivity. Retrosynthetic analysis revealed that a direct approach could exploit a desymmetrization^3^ of prochiral ketone **I** by an intramolecular Michael addition reaction to an α,β-unsaturated ester under enamine catalysis.^4^ Although aldol variants of this type are known,^5^ such a catalytic asymmetric Michael reaction has not been reported to date, despite its potential to directly provide morphan skeleton **II**, which possesses three stereocenters and a two carbon appendage useful for subsequent synthetic manipulation (Scheme 1).^6^ Accordingly, we viewed this proposed desymmetrization reaction as a good opportunity to unveil new reactivity in organocatalysis whilst accessing key bicyclic building blocks that are useful for the synthesis of morphan-like natural product libraries.

**scheme 1:**
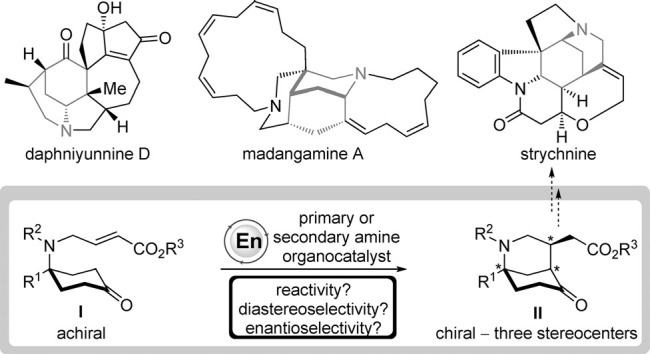
Desymmetrization strategy for the generation of morphans.

Initially, substrate **2 a** was chosen as a model system to test our concept; its precursor **1** was accessible on scale,^7^ the spirocyclic pyrrolidinone backbone would place reactive functionality in close proximity,^8^ and its synthesis by cross metathesis would provide a point of diversity if the desymmetrization proved successful.

Proof of concept was established quickly and unexpectedly; the attempted purification of **2 a** from ruthenium residues that had remained from the cross metathesis reaction with QuadraSil AP, a propylamine-functionalized silica gel scavenger, facilitated the formation of cyclized product (±)-**3 a** in quantitative yield and excellent diastereoselectivity (Scheme 2). Control experiments using propylamine in CH_2_Cl_2_ at room temperature, with and without additional benzoic acid as a co-catalyst, afforded the same product in high yields as a single diastereomer, and the primary amine functionality was thus identified as a catalytically competent species.

**scheme 2:**
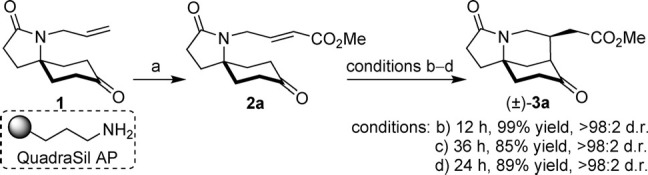
Synthesis of a model substrate and proof-of-concept transformations. Reagents and conditions: a) Hoveyda–Grubbs II catalyst, methyl acrylate, CH_2_Cl_2_, 45 °C, 48 h; b) QuadraSil AP, 0.5 mg per mg of substrate, CH_2_Cl_2_, RT; c) propylamine (20 mol %), CH_2_Cl_2_, RT; d) propylamine (20 mol %), PhCO_2_H (20 mol %), CH_2_Cl_2_, RT.

Consequently, a range of commonly used chiral single-enantiomer primary^9^ and secondary^10^ amine organocatalysts **4 a**–**4 e** were screened at 20 mol % loading against the model system in the presence of benzoic acid as a co-catalyst^11^ (Table 1). In terms of reactivity and enantioselectivity, (1*R*,2*R*)-cyclohexanediamine (**4 e**) was the most promising lead, and accordingly, derivatives were sought with the aim to boost enantioselectivity. Commercially available (1*R*,2*R*)-*trans*-*N*-Boc-1,2-cyclohexanediamine (**4 j**) gave similar results to **4 e**. However, Jacobsen’s thiourea catalyst **4 k**,4d with its increased hydrogen-bond-donor ability arising from the thiourea moiety,^12^ resulted in a significant increase in enantioselectivity whilst maintaining a short reaction time and high diastereoselectivity; the major diastereomeric product **3 a** was obtained in 90 % *ee* and >98:2 d.r. The loading of the primary amine catalyst could be reduced to 5 mol % with a nominal increase in enantioselectivity albeit with a longer reaction time, which was subsequently overcome by elevating the reaction temperature to 45 °C.

**Table 1:**
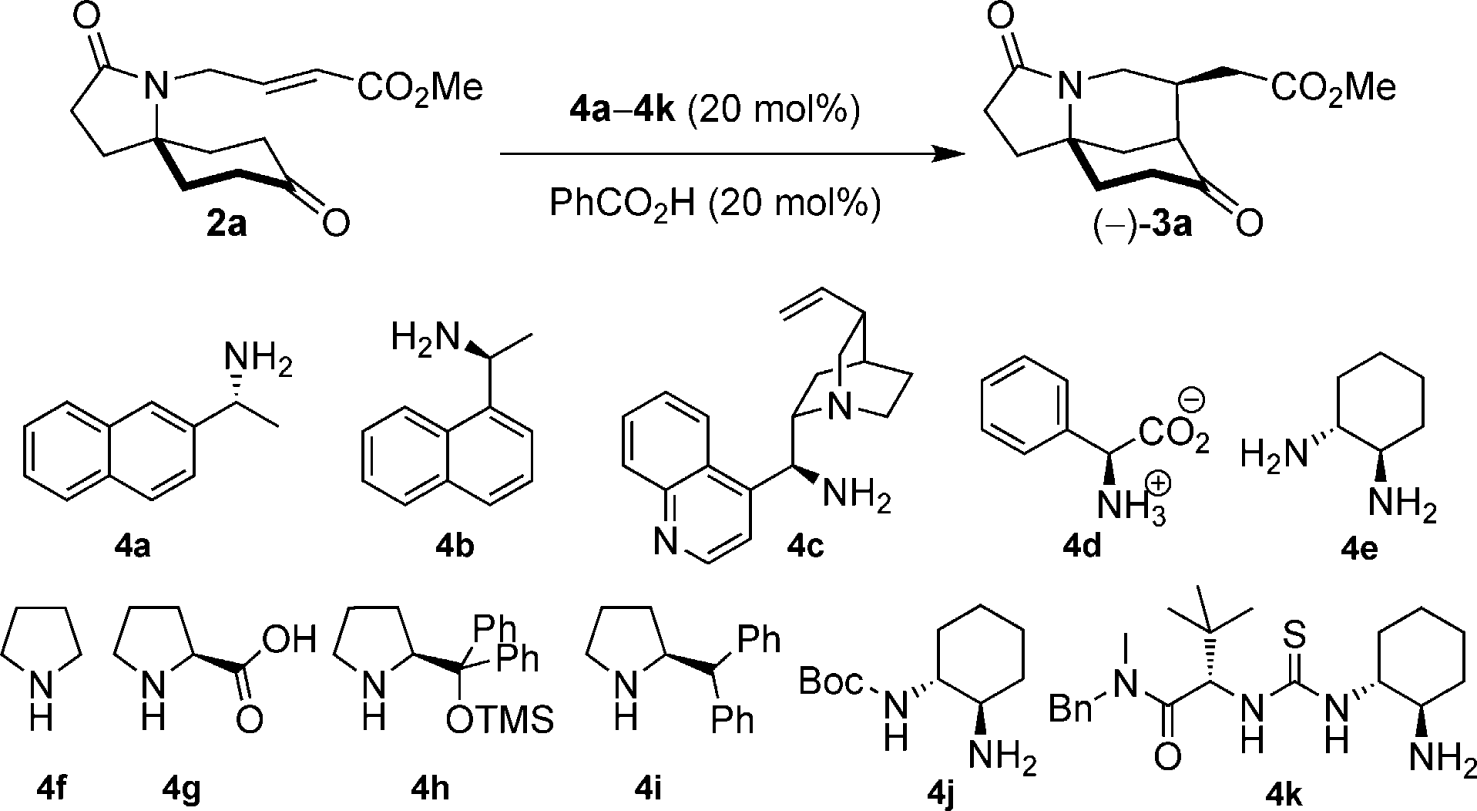
Reaction development and optimization.

Entry	Cat.	*t*	Yield^[a]^ [%]	d.r.^[b]^	*ee*^[c]^ [%]
1	**4 a**	5 days	72	>98:2	63
2	**4 b**	8 days	74	>98:2	69^[d]^
3	**4 c**	7 days	69	>98:2	31
4	**4 d**	NR	–	–	–
5	**4 e**	24 h	82	>98:2	63
6	**4 f**–**4 i**	NR	–	–	–
7	**4 j**	22 h	87	>98:2	64
8	**4 k**	26 h	86	>98:2	90
9^[e]^	**4 k**	25 h	78	>98:2	90
10^[f]^	**4 k**	96 h	80	>98:2	92
11^[g]^	**4 k**	48 h	88	>98:2	93

[a] Yield of isolated product after flash column chromatography.

[b] Diastereomeric ratios (d.r.) were determined by ^1^H NMR spectroscopy.

[c] The *ee* values were determined by HPLC analysis on a chiral stationary phase.

[d] (+)-**3 a** was obtained.

[e] CHCl_3_ as the solvent.

[f] **4 k** (5 mol %), benzoic acid (1.25 mol %), RT.

[g] **4 k** (5 mol %), benzoic acid (1.25 mol %), 45 °C. See the Supporting Information for details. Bn=benzyl, Boc=*tert*-butyloxycarbonyl, NR=no reaction. TMS=trimethylsilyl.

With optimized conditions established, the scope of the reaction with respect to the scaffold and the ester group was assessed (Table 2). Initially, changes to the ester group on the spirocyclic pyrrolidinone-containing construct were investigated (**2 a**—**2 d**), and pleasingly, the results for various esters were consistently good; enantioselectivities ranged from 90 to 93 % *ee*, and yields above 80 % were achieved (entries 1–4). Variations to the prochiral scaffold were next investigated. The spirocyclic pyrrolidine-containing α,β-unsaturated esters **2 e**–**2 h** were excellent substrates, although it was necessary to increase the benzoic acid co-catalyst loading to 2.5 mol % to maintain good reaction rates (entries 5–8). Pleasingly a non-spirocyclic substrate possessing *N*-ethyl and 4-methyl substituents underwent cyclization with equally high diastereo- and enantiocontrol (96 % *ee*) but at a marginally diminished reaction rate (entry 9). Related secondary amine substrates **2 j** and **2 k** possessing larger C4 substituents also reacted to afford the *N*-unprotected morphan products **3 j** and **3 k** as single diastereomers with 84 and 83 % *ee*, respectively (entries 10 and 11). Substrates with a hydrogen atom at the C4 position would be the most relevant to natural product synthesis (Scheme 1), and accordingly, this scaffold type was assessed in the desymmetrization reaction. A series of ester substrates, **2 l**–**2 q**, each possessing an *N*-benzyl protecting group, were then examined. Pleasingly, these were found to give the highest enantioselectivities (96-99 % *ee*) of all of the scaffolds tested, albeit with diminished reaction rates (entries 12–17). Furthermore, a range of differentially *N*-protected substrates **2 r**–**2 v** gave the desired cyclized products **3 r**–**3 v** with excellent enantioselectivities (95–98 % *ee*; entries 18–22). In total, 22 unactivated α,β-unsaturated esters substrates with three points of diversity successfully cyclized under the action of catalyst **4 k** to give the bicyclic products with the morphan skeleton in high diastereo- and enantioselectivity. The relative stereochemical configuration of **3 p** and the absolute stereochemical configuration of a sulfonamide derivative of **3 r** were established by single-crystal X-ray analysis (see the Supporting Information).

**Table 2:** Scope of the intramolecular desymmetrization.^[a]^

Entry	Product		R	*t* [h]	Yield^[b]^ [%]	*ee*^[c]^ [%]
1	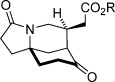	**3 a**	Me	40	88	93
2		**3 b**^[d]^	Et	40	89	90
3		**3 c**	*t*Bu	45	87	92
4		**3 d**	Bn	40	81	92
5	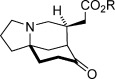	**3 e**	Me	36	83	94
6		**3 f**	Et	36	89	94
7		**3 g**	Bn	36	85	95
8		**3 h**	Ph	36	83	94
9	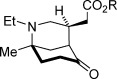	**3 i**	Et	96	83	96
10	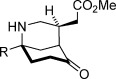	**3 j**	Ph(CH_2_)_2_	96	92	84
11		**3 k**	CH_3_(CH_2_)_16_	96	86	83
12	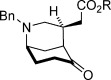	**3 l**	Et	84	84	96
13		**3 m**	Bn	84	89	97
14		**3 n**	Cy	120	85	96
15		**3 o**	*i*Pr	120	76	99
16		**3 p**	Me	96	90	97
17		**3 q**	Ph	96	72	97
18	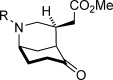	**3 r**	Boc	96	85	98
19		**3 s**	allyl	96	87	97
20		**3 t**	DPP	96	79	95
21		**3 u**	CH_2_(CH)_2_CO_2_Me	96	90	97
22		**3 v**	Me	96	84	96

[a] Catalyst **4 k** (5 mol %), PhCO_2_H (1.25 mol % for **3 a**–**d**, 2.5 mol % for **3 e**–**v**), CH_2_Cl_2_ (0.2 m), 45 °C (**3 a**–**i**) or 50 °C (**3 j**–**v**).

[b] Yield of isolated product after flash column chromatography (d.r. >98:2 for all products as determined by ^1^H NMR spectroscopy).

[c] The *ee* values were determined by HPLC analysis on a chiral stationary phase.

[d] The *Z* isomer of **2 b** gave (±)-**3 b** with >98:2 d.r. and in 82 % yield. Cy=cyclohexyl, DPP=diphenylphosphinoyl.

Interestingly, when *Z*-configured Michael acceptor (*Z*)-**2 b** was subjected to the optimized reaction conditions, it afforded the same morphan product (±)-**3 b** as the racemate indicating that geometrically pure *trans-*configured starting materials were crucial to achieving the high enantioselectivities observed. Taken together, these results clearly demonstrate that diastereoselectivity is a result of inherent substrate control and not a consequence of the chiral catalyst employed, which governs enantioselectivity. To understand the mechanism and origins of the high stereocontrol, quantum-chemical calculations were performed for the racemic and enantioselective series of our reaction.^13^ Stationary points were optimized at the M06-2X/6-311+G(d,p) level of theory;^14^ implicit solvation^15^ by CH_2_Cl_2_ was included using a conductor-like polarizable continuum model (CPCM). These results were corroborated by other computational methods (see the Supporting Information for details).^16^ In the interest of tractability, calculations were performed on *N*-methyl substrate **2 v** with a methylamine catalyst as a model for propylamine. The s*-cis* enamine conformation is more stable than the s*-trans* conformation by 2.9 kcal mol^−1^; however, we found that only the latter is able to undergo Michael addition as the enamine N–H must be oriented towards the ester: Proton transfer to the oxygen atom occurs along the (intrinsic) reaction coordinate, which is not possible for the s*-cis* conformer. A concerted ene reaction can be dismissed, as this step has an unfeasibly high activation barrier of 33.4 kcal mol^−1^. From the s-*trans* enamine, formation of the Michael *endo* diastereomer was computed to occur via chair-shaped **TS-A**, with a staggered conformation about the incipient C–C bond (Figure 1). In this transition state, proton transfer occurs asynchronously with C–C bond formation, giving enol adduct **E**. The keto tautomer results from a 1,3-prototropic shift in **TS-C**, assisted by the imine N atom to form *endo* adduct **F**. Formation of the alternative *exo* diastereomer is possible via **TS-B**. In this transition state, the forming six-membered ring adopts a boat conformation with greater eclipsing interactions about the incipient C–C bond than in *endo*-**TS-A**. The *exo* pathway is kinetically disfavored by 1.7 kcal mol^−1^, but more importantly, intramolecular proton transfer to the ester α-carbon atom is geometrically impossible for this diastereomer. We thus predict that *exo* enol **G** will revert back to the more stable starting enamine and not proceed to the keto tautomer. The exclusive *endo* selectivity results from an irreversible, kinetically favored pathway. Transition states corresponding to the formation of cyclobutane^17^ and cyclic enol ether intermediates^18^ were also located. Only cyclobutane **I** was computed to be more stable than the starting enamine, however, its formation is disfavored (**TS-D**) with respect to proton transfer to **TS-C** and is thus unlikely to constitute a significant resting state in the catalytic cycle.

**Figure 1:**
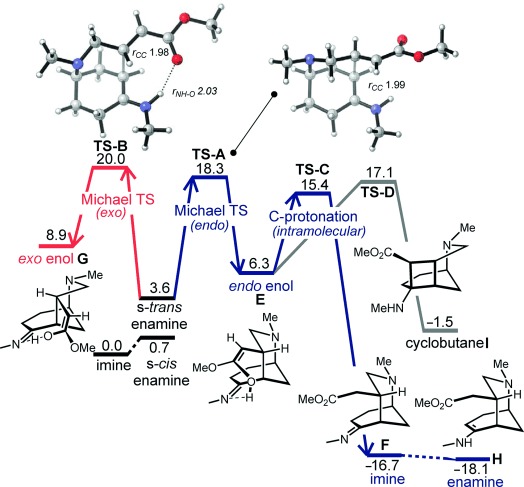
Free-energy profile for the cyclization of 2 v catalyzed by methylamine at the CPCM-M06-2X/6-311+G(d,p) level of theory (*G*_rel_ values in kcal mol^−1^ at 45 °C, 1 mol L^−1^).

We then considered the asymmetric induction arising from thiourea catalyst **4 k**. Our computations considered an enamine derived from substrate **2 v** with a simplified, truncated thiourea catalyst **4 l**. Low-energy conformations for each stationary point along the potential energy surface were located with Monte Carlo conformational searches^19^ employing semi-empirical PM6-DH2 calculations^20^ and subsequent refinement with M06-2X/6-311+G(d) optimizations (Figure 2). The reactive enamine geometry differs between the two pathways, with the s*-cis* enamine yielding the major enantiomer and the s*-trans* enamine yielding the minor enantiomer, as these conformations enable both pathways to benefit from stabilizing hydrogen bonding interactions between ester and thiourea. Energetic discrimination between Michael transition states **TS-J** and **TS-K** results from differing cyclohexylthiourea conformations: Rotation about the C(cyclohexyl)–N(thiourea) bond reveals a 4 kcal mol^−1^ conformational preference for the thiourea C–N bond to be *syn*-coplanar with the cyclohexyl C–H bond (*ϕ*_CNCH_=30°) over the antiperiplanar conformation (*ϕ*_CNCH_=180°). In the favored transition state **TS-J**, the catalyst adopts this preferred conformation (*ϕ*_CNCH_=31°) whereas in disfavored **TS-K**, the less stable form is adopted (*ϕ*_CNCH_=178°). The thiourea catalyst stabilizes ester enolate formation such that C–C bond formation and proton transfer now occur in two separate steps (see the Supporting Information for a full energy profile). The calculated enantioselectivity imparted by catalyst **4 l** amounts to 96 % *ee* based on a ΔΔ*G*^≠^ value of 2.4 kcal mol^−1^ between the selectivity-determining transition states along the two pathways, which agrees with the absolute sense and magnitude (96 % *ee*) obtained with **4 k**. Our computational studies predicted that thiourea catalyst **4 l** would thus be as competent as **4 k** despite being greatly simplified. The computed transition state **TS-J** suggests a lack of any significant contribution from the *tert*-leucine fragment in catalyst **4 k**, as it would be oriented away from the substrate into space. Accordingly, **4 l** was then synthesized and tested in the cyclization of **2 v** to validate the computational prediction of enantioselectivity (Scheme 3). Pleasingly, product **3 v** was obtained in 83 % yield and 97 % *ee* as a single diastereomer, showing excellent agreement between experiment and theory.

**Figure 2:**
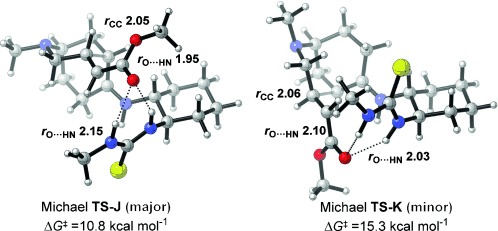
Transition states of the aminothiourea-catalyzed Michael reaction forming enantiomeric adducts of the *endo* diastereomer computed at the CPCM-M06-2X/6-311+G(d,p) level of theory.

**scheme 3:**
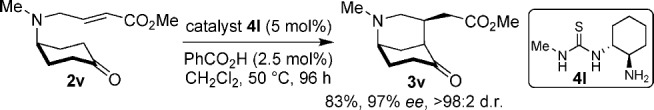
Computer-aided catalyst design of 4 l.

In summary, we have developed a highly enantioselective primary amine catalyzed Michael addition of a ketone to unactivated α,β-unsaturated esters. The reaction benefits from three points of diversity—the C4 substituent, the nitrogen group, and the ester moiety—and provides access to the morphan scaffold in high enantio- and diastereoselectivity (up to 99 % *ee* and >98:2 d.r.). Computational studies to probe the origins of the high enantiocontrol have been performed, and the results of the calculations identified a new low-molecular-weight catalyst that can impart the same level of enantioselectivity. The application of this new enantioselective desymmetrization method to complex natural product synthesis is ongoing in our group, and the findings will be reported in due course.

## Supporting Information

As a service to our authors and readers, this journal provides supporting information supplied by the authors. Such materials are peer reviewed and may be re-organized for online delivery, but are not copy-edited or typeset. Technical support issues arising from supporting information (other than missing files) should be addressed to the authors.

miscellaneous_information
