# Supplementary material for: Enantioselective Desymmetrization of Prochiral Cyclohexanones by Organocatalytic Intramolecular Michael Additions to α,β-Unsaturated Esters
Source: Angew Chem Int Ed Engl. 2015 Feb 27;54(16):4899–903. doi: 10.1002/anie.201411924 (PMC4678487; doi:10.1002/anie.201411924)
Supplement: Supplementary file 1 [file anie0054-4899-sd1.pdf]

Angewandte Chemie
Eine Zeitschrift der Gesellschaft Deutscher Chemiker

Supporting Information

Enantioselective Desymmetrization of Prochiral Cyclohexanones by Organocatalytic Intramolecular Michael Additions to α,β-Unsaturated Esters**

Adam D. Gammack Yamagata, Swarup Datta, Kelvin E. Jackson, Linus Stegbauer, Robert S. Paton, and Darren J. Dixon**

anie_201411924_sm_miscellaneous_information.pdf

Table of Contents

Contents

1. General experimental 3
2. Synthesis & characterization of substrates 2a-d. 4
3. Synthesis & characterization of substrate *Z*-2b. 7
4. Synthesis & characterization of 4-bromocrotonates 10a-d 9
5. Synthesis and characterization of substrates 2e-2h 12
6. Synthesis and characterization of substrate 2i 18
7. Synthesis and characterization of 2j 21
8. Synthetic scheme for the synthesis of 2k 24
9. Synthesis and characterization of substrates 2l-q: 27
10. Synthesis and characterization of substrates 2r and 2t 34
11. Synthesis and characterization of substrate 2s 37
12. Synthesis and characterization of substrate 2u 39
13. Synthesis and characterization of substrate 2v 40
14. Catalyst screen and optimization 41
15. Synthesis and characterization of chiral compounds 3a-k, m-n 43
16. General Procedure F- for the synthesis of chiral products (3a-d): 43
17. General Procedure G - for the synthesis of chiral products (3e-3v): 43
18. General procedure H - for the synthesis of racemic products (3a-3v): 43
19. Synthesis and characterization of catalyst 4l 59
20. Cyclisation of 2v using truncated 4l 60
21. Single crystal preparation for X-ray analysis 61
22. NMR spectra 63
23. HPLC traces 153
24. Computational SI........ 176

General experimental

Organocatalytic reactions were performed with standard solvents without any additional drying under a nitrogen atmosphere. All other reactions were performed under anhydrous conditions using dried solvents in oven dried glassware (100 °C) and carried out under a nitrogen atmosphere. All solvents were commercially supplied or provided by the communal stills of the Chemistry Research Laboratory, Oxford. Petroleum ether (PE) refers to the fraction collected between 30-40 °C.

Column chromatography was carried out using Merck Kieselgel 60 silica gel (230-400 mesh). All reactions were followed by thin-layer chromatography (TLC) where practical, using Merck Kieselgel 60 F254 (230-400 mesh) fluorescent treated silica plates which were visualised under UV light (254nm) or by staining with aqueous basic potassium permanganate solutions as appropriate.

All ^{1}H and ^{13}C NMR spectra were recorded using a Bruker 500 MHz and Bruker 400 MHz spectrometers with residual protic solvents as the internal standard. Chemical shifts (δ) are given in parts per million (ppm), and coupling constants (J) are given in Hertz (Hz). The ^{1}H NMR spectra are reported as follows: δ/ppm (multiplicity (s = singlet, d = doublet, t = triplet, q = quartet, m = multiplet), coupling constants J/Hz (where appropriate), number of protons). IR spectra were recorded on an ATI Mattson: Genesis Series FT spectrometer or a Bruker Tensor 27 FT-IR spectrometer, from a thin film deposited on a diamond plate and only selected maximum absorbances are reported. High resolution mass spectra (accurate mass) were recorded on a Thermo Finnigan Mat 95XP mass spectrometer or a Bruker MicroTof mass spectrometer (electrospray technique ES). Melting points were recorded in degrees Celsius (°C) and measured on a Leica Galen III apparatus, at ambient temperature.

Compound names are those generated by ACD LABS 12.0 software following the IUPAC nomenclature.

Synthesis & characterization of substrates 2a-d.

<u>General procedure A</u> for synthesis of substrates (2a-d):

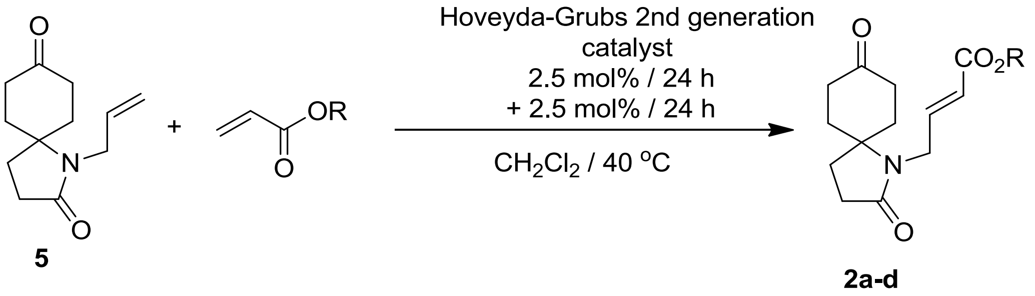

To a mixture of **5**[1] (500 mg, 2.4 mmol, 1 eq.) and appropriate acrylate (48 mmol, 20 eq.) in dichloromethane (23 mL) was added Hoveyda-Grubs catalyst 2nd generation (2.5 mol %) and stirred at 40 °C for 24 h. After cooling to room temperature another portion of Hoveyda-Grubs catalyst 2nd generation (2.5 mol %) was added and the mixture was stirred for another 24 h at 40 °C. The resulting solution was filtered through a short silica pad and the solvent was removed under reduced pressure. The crude product was purified by flash column chromatography.

Synthesis and characterization of (*E*)-methyl 4-(2,8-dioxo-1-azaspiro[4.5]decan-1-yl)but-2-enoate (2a):

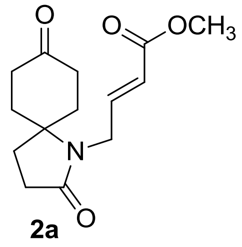

2a was obtained according to the **general procedure A** using methyl acrylate as a starting material. The crude reaction mixture was purified by flash column chromatography (Ether/MeOH 95:5) to yield 470 mg (74 %) of product as a white solid.

mp. 95-97 °C; **FT-IR** ν_{max}/cm^{-1} 2951, 2360, 1717, 1683; **^{1}H NMR** (400 MHz, $CDCl_3$) δ 6.82 (dt, *J* = 15.7, 5.4 Hz, 1H), 5.88 (dt, *J* = 15.7, 1.7 Hz, 1H), 3.95 (dd, *J* = 5.3, 1.6 Hz, 2H), 3.70 (s, 3H), 2.56 – 2.45 (m, 4H), 2.46 – 2.37 (m, 2H), 2.24 (t, *J* = 8.0 Hz, 2H), 2.03 (td, *J* = 13.5, 5.0 Hz, 2H), 1.89 – 1.76 (m, 2H). **^{13}C NMR** (100 MHz, $CDCl_3$) δ 208.2, 174.3, 166.2, 144.0, 122.2, 62.4, 51.7, 40.0, 37.5 (2C), 34.1 (2C), 28.9, 28.8; **HRMS** (ESI+) calcd. for $C_{14}H_{19}NNaO_4$ $[M+Na]^+$ 288.1206, found 288.1207.

---

(1) Compound 1-allyl-1-azaspiro[4.5]decane-2,8-dione (**5**) was synthesized following the procedure of Kan, T.; Fujimoto, T.; Ieda, S.; Asoh, Y.; Kitaoka, H.; Fukuyama, T., *Org. Lett.*, **2004,** 6, 2729-2731.

Synthesis and characterization of (*E*)-ethyl 4-(2,8-dioxo-1-azaspiro[4.5]decan-1-yl)but-2-enoate (2b):

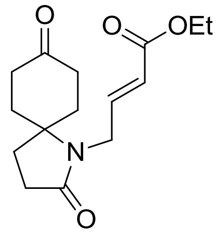

2b

2b was obtained according to the **general procedure A** using ethyl acrylate as a starting material. The crude reaction mixture was purified by flash column chromatography (Ether/MeOH 95:5) to yield 543 mg (81 %) of product as a white solid.

mp. 111-113 °C; **FT-IR** ν_{max}/cm^{-1} 2938, 1712, 1678; **^{1}H NMR** (400 MHz, $CDCl_3$) δ 6.78 (dt, *J* = 15.7, 5.3 Hz, 1H), 5.83 (dt, *J* = 15.7, 1.6 Hz, 1H), 4.12 (q, *J* = 7.1 Hz, 2H), 3.92 (dd, *J* = 5.3, 1.5 Hz, 2H), 2.56 – 2.42 (m, 4H), 2.42 – 2.31 (m, 2H), 2.21 (t, *J* = 8.0 Hz, 2H), 2.00 (td, *J* = 13.5, 4.9 Hz, 2H), 1.86 – 1.72 (m, 2H), 1.22 (t, *J* = 7.1 Hz, 3H). **^{13}C NMR** (100 MHz, $CDCl_3$) δ 208.2, 174.2, 165.7, 143.6, 122.5, 62.3, 60.5, 39.9, 37.5 (2C), 34.0 (2C), 28.8, 28.7, 14.2; **HRMS** (ESI+) calcd. for $C_{15}H_{20}NO_4$ $[M-H]^-$ 278.1398, found 278.1408.

Synthesis and characterization of (*E*)-*tert*-butyl 4-(2,8-dioxo-1-azaspiro[4.5]decan-1-yl)but-2-enoate (2c):

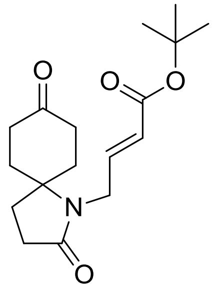

2c

2c was obtained according to the **general procedure A** using *tert*-butyl acrylate as a starting material. The crude reaction mixture was purified by flash column chromatography (Ether/MeOH 95:5) to afford 604 mg (82%) of product as a solid.

mp. 138-140 °C; **FT-IR** ν_{max}/cm^{-1} 2976, 1717, 1680; **^{1}H NMR** (400 MHz, $CDCl_3$) δ 6.66 (dt, *J* = 15.7, 4.6 Hz, 1H), 5.73 (d, *J* = 15.7 Hz, 1H), 3.88 (d, *J* = 5.0 Hz, 2H), 2.54 – 2.41 (m, 4H), 2.41 – 2.31 (m, 2H), 2.20 (t, *J* = 7.8 Hz, 2H), 1.99 (td, *J* = 13.4, 4.3 Hz, 2H), 1.85 – 1.72 (m, 2H), 1.40 (s, 9H); **^{13}C NMR** (101 MHz, $CDCl_3$) δ 208.3, 174.2, 165.0, 142.3, 124.1, 80.6, 62.3, 39.8, 37.5 (2C), 34.0 (2C), 28.8, 28.6, 28.0 (3C); **HRMS** (ESI+) calcd. for $C_{17}H_{24}NO_4$ $[M-H]^-$ 306.1711 found 306.1708.

Synthesis and characterization of (*E*)-benzyl 4-(2,8-dioxo-1-azaspiro[4.5]decan-1-yl)but-2-enoate (2d):

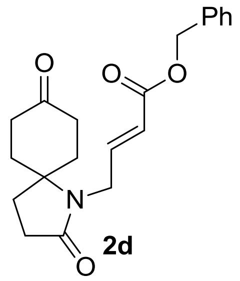

2d

2d was obtained according to the **general procedure A** using benzyl acrylate as a starting material. The crude reaction mixture was purified by flash column chromatography (Ether/MeOH 95:5) to afford 450 mg (55%) of product as a solid.

mp. 90-93 °C; **FT-IR** ν_{max}/cm^{-1} 2940, 1714, 1679; **^{1}H NMR** (400 MHz, $CDCl_3$) δ 7.40 – 7.18 (m, 5H), 6.85 (dt, *J* = 15.7, 5.2 Hz, 1H), 5.89 (dt, *J* = 15.7, 1.6 Hz, 1H), 5.12 (s, 2H), 3.93 (dd, *J* = 5.1, 1.5 Hz, 2H), 2.54 – 2.41 (m, 4H), 2.41 – 2.31 (m, 2H), 2.20 (t, *J* = 7.9 Hz, 2H), 1.99 (td, *J* = 13.4, 4.9 Hz, 2H), 1.85 – 1.72 (m, 2H); **^{13}C NMR** (100 MHz, $CDCl_3$) δ 208.2, 174.2, 165.5, 144.3, 135.6, 128.5 (2C), 128.3 (2C), 128.2, 122.0, 66.3, 62.3, 39.9, 37.4 (2C), 33.9 (2C), 28.8, 28.6; **HRMS** (ESI+) calcd. for $C_{20}H_{22}NO_4$ $[M-H]^-$ 340.1554 found 340.1549.

Synthesis & characterization of substrate *Z*-2b.

Synthetic scheme:

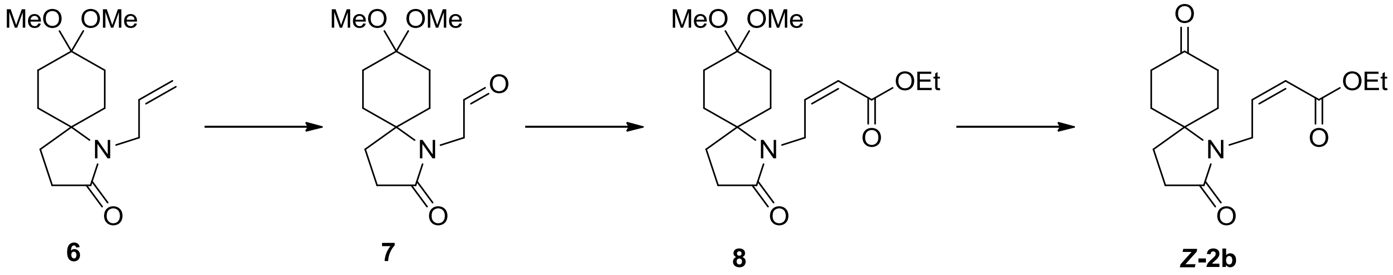

(8,8-Dimethoxy-2-oxo-1-azaspiro[4.5]dec-1-yl)acetaldehyde (7):

A solution of alkene **6**[2] (1 eq., 3.3 mmol, 840 mg) in a mixture of CH_2CL_2:MeOH (1:1; 10ml) was cooled to -78 °C. Ozone from an ozone generator was bubbled through the reaction mixture for 10 min at -78 °C upon which a faint blue color appeared. The resulting solution was quenched with dimethyl sulfide (10 eq., 33 mmol, 2.05 g) and allowed to stir for 3 h at -78 °C. The reaction mixture was concentrated *in vacuo* to give **7** as a yellow oil (2.6g, contaminated with DMSO). The compound was sufficiently pure to use in the next step with no further purification: A small amount of aldehyde was rapidly purified by FCC (EtOAc:MeOH 95:5) for analytical purposes. Purifying the bulk aldehyde led to decomposition products.

FT-IR ν_{max}/cm^{-1} 2898.73 (H-CO), 2829.99 (H-CO), 1730.70 (C=O); **^{1}H NMR** (250 MHz, C_6D_6) δ 9.26 (s, 1H), 3.60 (s, 2H), 3.10 (s, 3H), 3.01 (s, 3H), 2.15 (t, *J* = 8.1, 2H), 1.91 – 1.78 (m, 2H), 1.51 – 1.36 (m, 4H), 1.31 – 1.16 (m, 2H), 1.09 – 0.99 (m, 2H). **^{13}C NMR** (63 MHz, C_6C_6) δ 197.4, 174.1, 98.7, 62.1, 49.3, 47.7, 47.4, 31.5(2C), 29.3 (2C), 28.9, 28.8; **HRMS** (ES+) exact mass calculated for $[M+H]^+$ ($C_{13}H_{22}NO_4$) requires *m/z* 256.1543, found *m/z* 256.1536

Ethyl (2*Z*)-4-(8,8-dimethoxy-2-oxo-1-azaspiro[4.5]dec-1-yl)but-2-enoate (8):

To a solution of 18-crown-6 (2.50 eq., 3.43 mmol, 905 mg) and ethyl [bis(2,2,2-trifluoroethoxy)phosphinyl]acetate (1.20 eq., 1.647 mmol, 569 mg) in THF (15 ml) at -78 °C was added KHMDS (0.5M; 1.15 eq., 3.15 ml) dropwise and allowed to stir for 1 h at -40 °C. The reaction mixture was cooled to -78 °C and a solution of aldehyde **7** (1.00 eq., 1,37 mmol, 350 mg) in THF (5 ml) was added dropwise. The reaction was kept at -78 °C for 3 h and subsequently warmed to RT, quenched with sat. NH_4Cl (10 ml) and extracted with EtOAc (4×30 ml). The combined organic extracts were washed

(2) Compound **6** was synthesized following the procedure of Kan, T.; Fujimoto, T.; Ieda, S.; Asoh, Y.; Kitaoka, H.; Fukuyama, T., *Org. Lett.*, **2004,** 6, 2729-2731.

with brine, dried over $MgSO_4$, filtered and concentrated *in vacuo*. The reside was purified by FCC (EtOAc/PE 90:10) to the title compound **8** contaminated with its E isomer as a colourless oil (274 mg, 61%; mixture of 71:29 E:Z): The mixed isomers were further purified by FCC (iPA:Hexane 10:90 → 30:70) to afforded some pure Z isomer and fractions containing mixed isomers. This purification was repeated twice more on the remaining mixed fractions and the purified Z isomer was combined to afford **8** as a colourless oil (165 mg, 37%).

FT-IR ν_{max}/cm^{-1} 1713, 1684, 1370; **^{1}H NMR** (300 MHz, $CDCl_3$) δ 6.06 (dt, *J* = 11.5, 5.8 Hz, 1H), 5.74 (dt, *J* = 11.5, 2.1 Hz, 1H), 4.39 (dd, *J* = 5.7, 2.1 Hz, 2H), 4.13 (q, *J* = 7.1 Hz, 2H), 3.12 (s, 3H), 3.08 (s, 3H), 2.34 (t, *J* = 8.0 Hz, 2H), 2.00 – 1.86 (m, 4H), 1.77 (td, *J* = 13.3, 3.6 Hz, 2H), 1.40 (td, *J* = 13.7, 3.8 Hz, 2H), 1.30 (s, 1H), 1.24 (t, *J* = 7.1 Hz, 4H). **^{13}C NMR** (75 MHz, $CDCl_3$) δ 174.5, 166.0, 147.9, 120.1, 98.5, 63.4, 60.2, 47.9, 47.6, 38.0, 31.2 (2C), 29.1, 29.0 (2C), 28.9, 14.2. **HRMS** (ES+) exact mass calculated for $[M+Na]^+$ ($C_{17}H_{27}NNaO_5$) requires *m/z* 348.1781, found *m/z* 348.1784

Synthesis and characterization of (*Z*)-ethyl 4-(2,8-dioxo-1-azaspiro[4.5]decan-1-yl)but-2-enoate (Z-2b):

To **8** (68.9 mg, 0.212 mmol) was added a freshly prepared stock solution of $LiBF_4$ (19.9 mg, 1 eq.) in acetonitrile/water (98:2; 848 mL, 0.25 M wrt $LiBF_4$) at 0°C. The resulting solution was heated to 60°C for 25 minutes. The mixture was allowed to cool to room temperature; water (0.4 ml) was added and subsequently extracted with EtOAc (0.5 mL x 5), dried over Na_2SO_4 and concentrated *in vacuo*. The resulting oil was purified by FCC (PE:Acetone 70:30) to give **Z-2b** as a white solid (57 mg, 96%).

mp. 101-102 °C; **FT-IR** ν_{max}/cm^{-1} 1713, 1684, 1402; **^{1}H NMR** (400 MHz, $CDCl_3$) δ 6.08 (dt, *J* = 11.8, 6.0 Hz, 1H), 5.75 (dt, *J* = 11.4, 2.1 Hz, 1H), 4.45 (dd, *J* = 6.0, 2.1 Hz, 2H), 4.12 (q, *J* = 7.1 Hz, 2H), 2.49 – 2.42 (m, 3H), 2.35 (dd, *J* = 5.4, 2.3 Hz, 3H), 2.15 (t, *J* = 8.0 Hz, 2H), 2.07 (dt, *J* = 13.4, 6.5 Hz, 2H), 1.76 – 1.67 (m, 2H), 1.23 (t, *J* = 7.1 Hz, 3H). **^{13}C NMR** (75 MHz, $CDCl_3$) δ 208.32, 174.26, 165.97, 147.09, 120.43, 62.42, 60.37, 37.78, 37.53 (2C), 33.95 (2C), 28.97, 28.74, 14.20. **HRMS** (ES+) exact mass calculated for $[M+Na]^+$ ($C_{15}H_{21}NNaO_4$) requires *m/z* 302.1363, found *m/z* 302.1359

Synthesis & characterization of 4-bromocrotonates 10a-d

(2E)-4-bromobut-2-enoic acid (9):

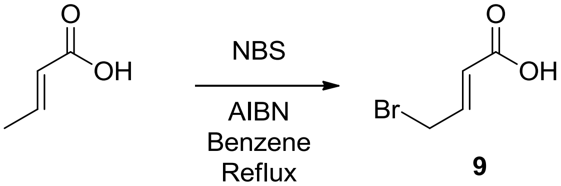

A modified literature procedure was used to synthesise **9**.[3] To a solution of crotonic acid (1eq., 116mmol, 10g) in benzene (100ml) was added NBS (1.03eq., 120mmol, 21.4g) and AIBN (0.016eq., 2.01mmol, 330mg). The resulting solution was refluxed for 2h, cooled to 5 ^{o}C and filtered through celite. The filter cake was washed with cold toluene and concentrated in *vacuo*. The residue was extracted (4 x 100ml) refluxing hexane, concentrated and recrystalised from hexane to give the title compound **9** as a white colorless solid (8.681g, 46%):

^{1}H NMR (400 MHz, CDCl3) δ 7.13 (dt, J=15.3, 7.3, 1H), 6.06 (dt, *J* = 15.3, 1.2, 1H), 4.04 (dd, *J* = 7.3, 1.3, 3H); Data consistent with literature.[3]

General procedure B for synthesis of 4-Bromocrotonates

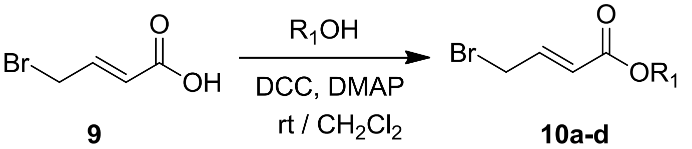

R$_1$ = CH$_2$Ph , c-Hex, i-Pr, Ph

To a solution of 4-bromocrotonic acid (1.0 eq.) in CH_2Cl_2 was added alcohol (R$_1$OH, 1.1 eq.) and DMAP (0.1 eq.). The solution was cooed to 0 ^{o}C and DCC (1.05 equiv.) was added portionwise. The reaction mixture was allowed to warm to RT and stirred overnight. The reaction mixture then filtered through short celite-pad and the residue was washed with CH_2Cl_2. Combined organic extracts were dried over Na_2SO_4 and concentrated under reduced pressure. The residue was purified by flash column chromatography (Et_2O/PE 1:50) to give compounds **10a-d**.

(3) den Hartog, T.; Maci, B.; Minnaard, A.; Feringa, B.; *Adv. Synth. Catal.* **2010**, 352, 999 – 1013

Benzyl (2E)-4-bromobut-2-enoate (10a):

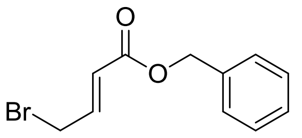

Using **general procedure B**: To a solution of bromocrotonic acid **9** (1eq., 6.1mmol, 1g), in CH_2Cl_2 (30ml) was added benzyl alcohol (1.1eq., 6.7mmol, 720mg), DMAP (0.1eq., 0.61mmol, 74mg) and cooled too 0^oC. DCC (1.05eq., 6.4mmol, 1.32g) was added in portions and the solution was allowed to warm to RT and stir for 8h. The solution was filtered, concentrated in *vacuo* and the residue was purified by FCC (98:2 PE :EtOAc) to give the title compound **10a** as a colourless oil (975mg, 63%):

^{1}H NMR (400 MHz, CDCl3) δ 7.42 – 7.33 (m, 5H), 7.06 (dt, J = 15.2, 7.4, 1H), 6.09 (dt, J = 15.4, 1.2, 1H), 5.21 (s, 2H), 4.02 (dd, J = 7.3, 1.2, 2H). Data consistent with literature.[4]

Phenyl (2E)-4-bromobut-2-enoate (10b):

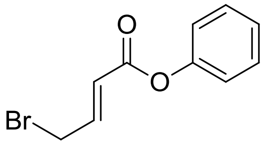

Using **general procedure B**: To a solution of bromocrotonic acid **9** (1eq., 9.1mmol, 1.5g), in CH_2Cl_2 (45ml) was added phenol (1.1eq., 10.05mmol, 944mg), DMAP (0.1eq., 0.915mmol, 117mg) and cooled too 0^oC. DCC (1.05eq., 10.06 mmol, 1.980g) was added in portions and the solution was allowed to warm to RT and stir for 8h. The resulting solution was filtered, concentrated in *vacuo* and the residue was purified by FCC (98:2 PE:EtOAc) to the title compound **10b** as a colourless oil (70%, 1.55g):

FT-IR ν_{max}/cm^{-1} 2935, 2860, 1722; **^{1}H NMR** (400 MHz, CDCl3) δ 7.44 – 7.38 (m, 2H), 7.29 – 7.18 (m, 2H), 7.13 (d, J = 7.5, 2H), 6.25 (dt, J = 15.3, 1.2, 1H), 4.08 (dd, J = 7.3, 1.3, 2H). ^{13}C NMR (101 MHz, $CDCl_3$) δ 163.9, 150.4, 143.6, 129.4 (2C), 126.0, 123.9, 121.4 (2C), 28.9. **HRMS** (ESI+) calcd. for $C_{10}H_9BrNaO_2$+ $[M+Na]^+$ 262.9678, found 262.9679.

---

(4) Sun, G.; Savle, P.; Gandour, D.; Níc a' Bhaírd, N.; Ramsay, R.; Fronczekt, F.; *J. Org. Chem.*, **1995**, 60, 6688-6695

(*E*)-cyclohexyl 4-bromobut-2-enoate (10c):

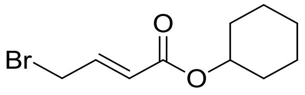

10c obtained according to **general procedure B** using 4-bromocrotonic acid **9** (1.0 g, 6.06 mmol) and cyclohexanol (0.69 mL, 6.66 mmol,) as a starting materials. The crude reaction mixture was purified by flash column chromatography to afford 596 mg (40%) of product.

FT-IR ν_{max}/cm^{-1} 2936, 2859, 1714; **^{1}H NMR** (400 MHz, $CDCl_3$) δ 7.08 – 6.89 (m, 1H), 6.02 (d, *J* = 15.3 Hz, 1H), 4.01 (d, *J* = 7.4 Hz, 2H), 1.95 – 1.82 (m, 2H), 1.79 – 1.68 (m, 2H), 1.62 – 1.51 (m, 1H), 1.51 – 1.20 (m, 6H); **^{13}C NMR** (100 MHz, $CDCl_3$) δ 164.9, 141.2, 125.2, 73.1, 31.5 (2C), 29.2, 25.33, 23.7 (2C); **HRMS** (ESI+) calcd. for $C_{10}H_{15}BrNaO_2$ $[M+Na]^+$ 271.0127, found 271.0123.

(*E*)-*iso*propyl 4-bromobut-2-enoate (10d):

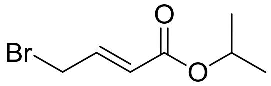

10d was obtained according to **general procedure B** using 4-bromocrotonic acid **9** (1.0 g, 6.06mmol) and isopropyl alcohol (6.66 mmol, 0.51 mL) as a starting materials. The crude reaction mixture was purified by flash column chromatography to afford 221mg (42%) of product as a colourless oil.

FT-IR ν_{max}/cm^{-1} 2938, 2858, 1716; **^{1}H NMR** (400 MHz, $CDCl_3$) δ 6.98 (dt, *J* = 15.3, 7.5 Hz, 1H), 6.00 (dt, *J* = 15.3, 1.2 Hz, 1H), 5.07 (dt, *J* = 12.5, 6.3 Hz, 1H), 4.01 (dd, *J* = 7.4, 1.2 Hz, 2H), 1.28 (d, *J* = 6.2 Hz, 6H). **^{13}C NMR** (101 MHz, $CDCl_3$) δ 165.03, 141.31, 125.20, 68.21, 29.23, 21.82. **HRMS** (ESI+) calcd. for $[M+Na]^+$ $C_7H_{11}BrNaO_2$+ 228.9835, found 228.9840.

Synthesis and characterization of substrates 2e-2h

Synthetic scheme:

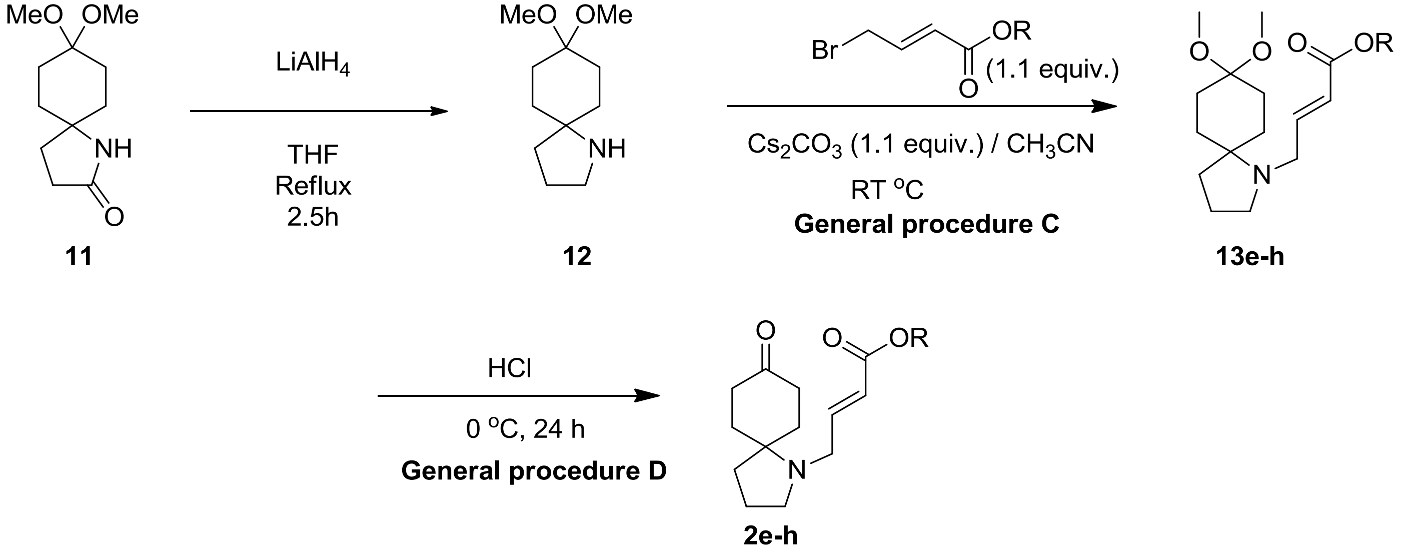

Synthesis and characterization of 8,8-Dimethoxy-1-azaspiro[4.5]decane (12):

To $LiAlH_4$ (4.0 eq., 22 mmol, 1.7g) under nitrogen atmosphere was added THF (5 ml) and subsequently cooled to at -78 °C. A solution of amide **11**[5] (1.0 eq., 5.4 mmol, 2.3g) in THF (25 ml) was added dropwise at this temperature and allowed to stir for 10 minutes. The resulting grey suspension was refluxed for 2.5 h and then it cooled to -78 °C, quenched with MeOH (30 ml) and allowed to stir for 2 h at RT. The reaction suspension mixture was diluted with Et_2O (30 ml); Glaubers salt ($Na_2SO_4.10H_20$ 50 g) was added and the resulting mixture was stirred vigourously for 4 h. The reaction mixture was then filtered through a scinter. The filter cake was washed with Et_2O (4 × 50 ml) and the combined organic extracts were concentrated *in vacuo* to afford compound **12** as a pale yellow oil (2.102 g, 98%). The compound was sufficiently pure to use in the next step without further purification:

FT-IR ν_{max}/cm^{-1} 2947.89 (N-H), 1104.94 (O-CH_3), 1053.37 (O-CH_3); **^{1}H NMR** (400 MHz, $CDCl_3$) δ 3.15 (s, 6H,), 2.92 (t, J = 6.8, 2H), 1.85 – 1.69 (m, 5H), 1.65 – 1.45 (m, 8H); **^{13}C NMR** (101 MHz, $CDCl_3$) δ 99.7, 60.0, 47.0, 47.5, 45.7, 36.3, 34.2 (2C), 29.8 (2C), 25.4; **HRMS** (ES+) exact mass calculated for $[M+H]^+$ ($C_{11}H_{22}NO_2$) requires m/z 200.1645, found m/z 200.1649.

(5) Compound **11** was synthesized following the procedure of Kan, T.; Fujimoto, T.; Ieda, S.; Asoh, Y.; Kitaoka, H.; Fukuyama, T., Org. Lett., **2004,** 6, 2729-2731.

<u>General procedure C</u> for amine alkylation and subsequent acetal deprotection (13e-h):

Alkylation to give (13e-h): To a solution of **12** (1 eq.) in CH_3CN (8.0 mL) was added Cs_2CO_3 (2.4 eq.) at room temperature. 4-Bromocrotonate (2.4 mmol) was added to the reaction mixture and subsequently warmed to 40 ^oC for 24 h. Water was added and it was extracted with ethyl acetate (3x). The combined organic layer washed with brine, dried over Na_2SO_4 and concentrated under reduced pressure. The residue was purified by flash column chromatography on silica gel to give the pure product (**13e-h**).

<u>General Procedure D</u> for of dimethyl acetal deprotection (2e-h):

Deprotection of dimethyl acetal (step 2): To a THF solution (3.5 mL) of **13e-h** (0.6 mmol) was added 0.5 M HCl (3.5 mL) and the reaction mixture was stirred at room temperature for 5 days. Saturated $NaHCO_3$ solution was added until pH 9 was obtained. The resulting solution was extracted with ethyl acetate 3 times and the combined organic layer was washed with water, dried over Na_2SO_4 and concentrated under reduced pressure. The residue was purified by flash column chromatography on silica gel to give the pure products (**2e-2h**). Attempts to shorten this deprotection time by increasing the amount of acid led to quantities of cyclisation products (**3e-h**)

Synthesis and characterization of Methyl (2*E*)-4-(8,8-dimethoxy-1-azaspiro[4.5]dec-1-yl)but-2-enoate (13e):

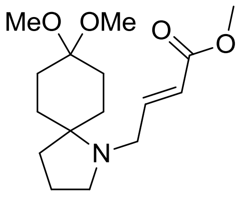

General Procedure C was applied to amine **12** (600 mg, 3.02 mmol) with methyl (2*E*)-4-bromobut-2-enoate. The residue was purified by FCC (2:1 EtOAc:PE) to give the title compound **13e** as a yellow oil (671 mg, 75%):

FT-IR ν_{max}/cm^{-1} 1723.34 (C=O), 1658.07 (C=C); **^{1}H NMR** (400 MHz, $CDCl_3$) δ 6.95 (dt, *J* = 15.5, 5.8, 1H), 5.97 (d, *J* = 15.6, 1H), 3.70 (s, 3H), 3.21 (d, *J* = 5.8, 2H), 3.17 (s, 3H), 3.13 (s, 3H), 2.71 (s, 2H), 1.98 (d, *J* = 11.9, 2H), 1.72 (s, 4H), 1.56 – 1.46 (m, 2H), 1.42 – 1.32 (m, 2H), 1.24 (s, 2H); **^{13}C NMR** (101 MHz, $CDCl_3$) δ 166.9 9, 148.1, 121.3, 99.5, 62.8, 51.4, 51.1, 49.2, 47.8, 47.5, 33.9, 30.1 (2C), 28.1 (2C), 20.9; **HRMS** (ES+) exact mass calculated for $[M+Na]^+$ ($C_{16}H_{27}NNaO_4$) requires *m/z* 320.1833, found *m/z* 320.1826.

Synthesis and characterization of Methyl (2*E*)-4-(8-oxo-1-azaspiro[4.5]dec-1-yl)but-2-enoate (2e):

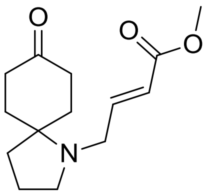

General Procedure D was applied to dimethyl acetal **13e** (2.06 mmol, 612 mg). The residue was purified by FCC (80:20 EtOAc:PE) to give the title compound **2e** as a yellow oil 462 mg, 89%):

FT-IR ν_{max}/cm^{-1} 1746.16 (C=O), 1733.25 (C=O), 1658.09 (C=C); **^{1}H NMR** (400 MHz, $CDCl_3$) δ 6.92 (dt, *J* = 15.6, 5.7, 1H), 5.98 (d, *J* = 15.6, 1H), 3.70 (s, 3H), 3.20 (d, *J* = 5.6, 2H), 2.77 (t, *J* = 7.0, 2H), 2.46 – 2.32 (m, 4H), 1.99 – 1.92 (m, 2H), 1.80 (m, 4H), 1.72 – 1.64 (m, 2H); **^{13}C NMR** (101 MHz, $CDCl_3$) δ 211.2, 166.8, 147.5, 121.5, 62.0, 51.5, 51.2, 49.0, 38.9 (2C), 34.0, 31.5 (2C), 21.0; **HRMS** (ES+) exact mass calculated for $[M+H]^+$ ($C_{14}H_{22}NO_3$) requires *m/z* 252.1594, found *m/z* 253.1594.

Synthesis and characterization of Ethyl (2*E*)-4-(8,8-dimethoxy-1-azaspiro[4.5]dec-1-yl)but-2-enoate (13f):

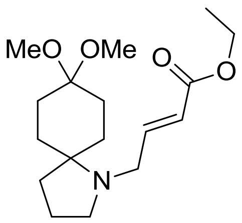

General Procedure C was applied to amine **12** (600 mg, 3.02 mmol) with ethyl (2*E*)-4-bromobut-2-enoate. The residue was purified by FCC (2:1 EtOAc:PE) to give the title compound **13f** as a yellow oil (638 mg, 68%):

FT-IR ν_{max}/cm^{-1} 1717.80 (C=O), 1656.73(C=C); **^{1}H NMR** (400 MHz, $CDCl_3$) δ 6.94 (dt, *J* = 15.5, 5.9, 1H,), 5.96 (d, *J* = 15.6, 1H), 4.15 (q, *J* = 7.1, 2H), 3.21 (d, *J* = 5.4, 2H), 3.16 (s, 3H), 3.12 (s, 3H), 2.71 (bs, 2H), 1.98 (d, *J* = 11.5, 2H), 1.72 (s, 4H), 1.57 – 1.45 (m, 2H), 1.43 – 1.32 (m, 2H), 1.25 (m, 5H); **^{13}C NMR** (101 MHz, $CDCl_3$) δ 166.5, 147.7, 121.8, 99.5, 62.8, 60.2, 51.2 , 49.2, 47.8, 47.5, 33.9, 30.1 (2C), 28.1 (2C), 20.9, 14.2; **HRMS** (ES+) exact mass calculated for $[M+Na]^+$ ($C_{17}H_{29}NNaO_4$) requires *m/z* 334.1989, found *m/z* 334.1987.

Synthesis and characterization of Ethyl (2*E*)-4-(8-oxo-1-azaspiro[4.5]dec-1-yl)but-2-enoate (2f):

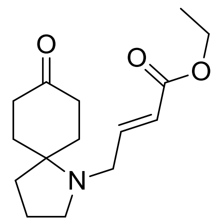

General Procedure D was applied to dimethyl acetal **13f** (1.82 mmol, 566 mg). The residue was purified by FCC (80:20 EtOAc:PE) to give the title compound **2f** as a yellow oil (454 mg, 96%):

FT-IR ν_{max}/cm^{-1} 1712.79 (C=O), 1656.22 (C=C); **^{1}H NMR** (400 MHz, $CDCl_3$) δ 6.91 (dt, *J* = 15.6, 5.7, 1H), 5.97 (d, *J* = 15.6, 1H), 4.15 (q, *J* = 7.1, 2H), 3.19 (dd, *J* = 5.7, 1.3, 2H), 2.77 (t, *J* = 7.0, 2H), 2.39 (m, 4H), 1.98 – 1.92 (m, 2H), 1.87 – 1.74 (m, 4H), 1.72 – 1.64 (m, 2H), 1.25 (t, *J* = 7.1, 3H); **^{13}C NMR** (101 MHz, $CDCl_3$) δ 211.2, 166.4, 147.2, 122.0, 62.0, 60.3, 51.2, 49.1, 38.9 (2C), 34.0, 31.5 (2C), 20.9, 14.2; **HRMS** (ES+) exact mass calculated for $[M+H]^+$ ($C_{15}H_{24}NO_3$) requires *m/z* 266.1751, found *m/z* 266.1752.

Synthesis and characterization of Benzyl (2*E*)-4-(8,8-dimethoxy-1-azaspiro[4.5]dec-1-yl)but-2-enoate (13g):

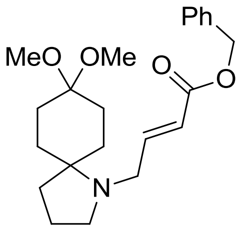

General Procedure C was applied to amine **12** (600 mg, 3.02 mmol) with benzyl (2*E*)-4-bromobut-2-enoate (**10a**). The residue was purified by FCC (2:1 EtOAc:PE) to give the title compound **13g** as a yellow oil (762 mg, 68%):

FT-IR ν_{max}/cm^{-1} 1717.85 (C=O), 1655.73 (C=C); ^{1}**H NMR** (400 MHz, $CDCl_3$) δ 7.37 – 7.27 (m, 5H), 7.01 (dt, *J* = 15.5, 5.8, 1H), 6.04 (d, *J* = 15.6, 1H), 5.15 (s, 2H), 3.24 – 3.20 (m, 2H), 3.17 (s, 3H), 3.13 (s, 3H), 2.74 – 2.68 (m, 2H), 1.99 (d, *J* = 11.8, 2H), 1.75 – 1.70 (m, 4H), 1.56 – 1.47 (m, 2H), 1.43 – 1.34 (m, 2H), 1.24 (d, *J* = 12.2, 2H); ^{13}**C NMR** (101 MHz, $CDCl_3$) δ 166.3, 148.6, 136.1, 128.5 (2C), 128.3 (2C), 128.1, 121.4, 99.5, 66.05, 62.8, 51.2 , 49.3, 47.8, 47.5, 33.9, 30.2 (2C), 28.2 (2C), 21.0; **HRMS** (ES+) exact mass calculated for $[M+H]^+$ ($C_{22}H_{32}NO_4$) requires *m/z* 396.2145, found *m/z* 396.2136.

Synthesis and characterization of Benzyl (2*E*)-4-(8-oxo-1-azaspiro[4.5]dec-1-yl)but-2-enoate (2g):

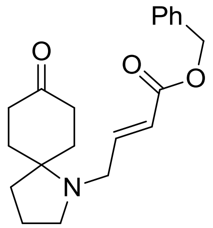

General Procedure D was applied to dimethyl acetal **13g** (1.83 mmol, 681 mg). The residue was purified by FCC (60:40 EtOAc:PE) to give the title compound **2g** as a yellow oil (511 mg, 85% mg):

FT-IR ν_{max}/cm^{-1} 1712.79 (C=O), 1656.22 (C=C); ^{1}**H NMR** (400 MHz, $CDCl_3$) δ 7.39 – 7.29 (m, 5H), 6.98 (dt, *J* = 15.6, 5.6, 1H), 6.05 (d, *J* = 15.6, 1H), 5.16 (s, 2H), 3.21 (d, *J* = 5.4, 2H), 2.78 (t, *J* = 7.0, 2H), 2.40 (t, *J* = 8.8, 4H), 1.99 – 1.93 (m, 2H), 1.89 – 1.74 (m, 4H), 1.73 – 1.64 (m, 2H). ^{13}**C NMR** (101 MHz, $CDCl_3$) δ 211.2, 166.2, 147.9, 136.0, 128.5 (2C), 128.3 (2C), 128.2, 121.6, 66.2, 62.0, 51.3, 49.1, 38.9, 34.0, 31.5, 21.0; **HRMS** (ES+) exact mass calculated for $[M+H]^+$ ($C_{20}H_{26}NO_3$) requires *m/z* 328.1907, found *m/z* 328.1902.

Synthesis and characterization of Phenyl (2E)-4-(8,8-dimethoxy-1-azaspiro[4.5]dec-1-yl)but-2-enoate (13h)

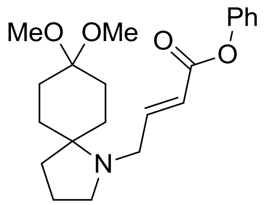

General Procedure C was applied to amine **12** (500 mg, 2.51 mmol) with Phenyl (2*E*)-4-bromobut-2-enoate **10b**. The residue was purified by FCC (1:1 EtOAc:PE) to give compound **13h** as a yellow oil (805 mg, 86%): **FT-IR** ν_{max}/cm^{-1} 1735.58 (C=O), 1652.61 (C=C); **^{1}H NMR** (400 MHz, $CDCl_3$) δ 7.37 (t, *J* = 7.9, 2H), 7.24 – 7.15 (m, 2H), 7.11 (d, *J* = 7.6, 2H), 6.20 (d, *J* = 15.6, 1H), 3.31 (d, *J* = 5.0, 2H), 3.19 (s, 3H), 3.16 (s, 3H), 2.78 (d, *J* = 5.9, 2H), 2.02 (d, *J* = 11.7, 2H), 1.82 – 1.74 (m, 4H), 1.61 – 1.51 (m, 2H), 1.46 – 1.36 (m, 2H), 1.33 – 1.24 (m, 2H); **^{13}C NMR** (101 MHz, $CDCl_3$) δ 164.9, 150.7, 150.2, 129.4 (2C), 125.7, 121.6 (2C), 121.0, 99.5, 62.9, 51.3, 49.3, 47.8, 47.6, 34.0, 30.1 (2C), 28.2 (2C), 21.0; **HRMS** (ES+) exact mass calculated for $[M+Na]^+$ ($C_{21}H_{29}NNaO_4$) requires *m/z* 382.1989, found *m/z* 382.1984.

Synthesis and characterization of Phenyl (2*E*)-4-(8-oxo-1-azaspiro[4.5]dec-1-yl)but-2-enoate (2h):

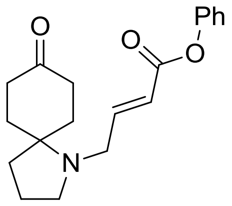

General Procedure D was applied to dimethyl acetal **13h** (1.81 mmol, 655 mg). The residue was purified by FCC (70:30 EtOAc:PE) to give the compound **2h** as a yellow oil (449 mg, 79%):

FT-IR ν_{max}/cm^{-1} 1731.97 (C=O), 1712.94 (C=O), 1653.48 (C=C); **^{1}H NMR** (400 MHz, $CDCl_3$) δ 7.37 (t, *J* = 7.9, 2H), 7.27 – 7.08 (m, 4H), 6.21 (d, *J* = 15.6, 1H), 3.30 (d, *J* = 5.4, 2H), 2.85 (t, *J* = 6.9, 2H), 2.50 – 2.35 (m, 4H), 2.05 – 1.95 (m, 2H), 1.94 – 1.78 (m, 4H), 1.78 – 1.68 (m, 2H); **^{13}C NMR** (101 MHz, $CDCl_3$) δ 211.1, 164.8, 150.7, 149.6, 129.4, 125.7, 121.6, 121.2, 62.1, 51.3, 49.1, 38.9 (2C), 34.0, 31.5 (2C), 21.0; **HRMS** (ES+) exact mass calculated for $[M+H]^+$ ($C_{19}H_{24}NO_3$) requires *m/z* 314.1751, found *m/z* 314.1748.

Synthesis and characterization of substrate 2i

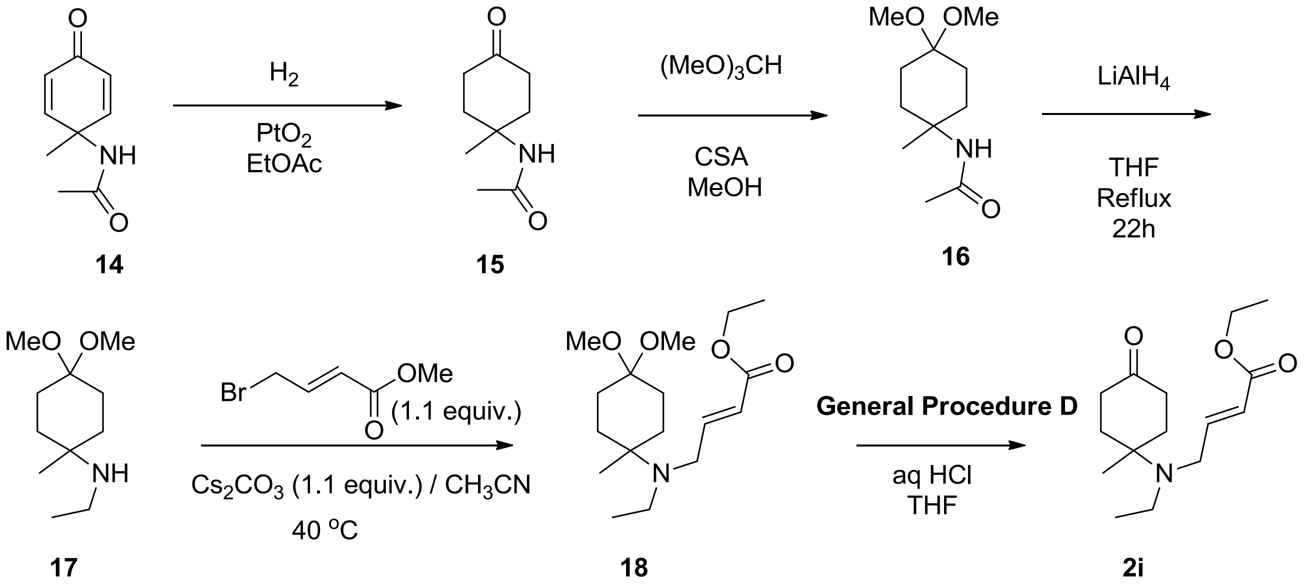

Synthesis and characterization of *N*-(1-Methyl-4-oxocyclohexyl)acetamide (15):

A purged (3 × N_2) stirred suspension of **14**[6] (1 eq., 1.429 g, 8.66 mmol) and PtO_2 (0.025 eq., 0.22 mmol, 50 mg) in EtOAc (45 ml) was subjected to a hydrogen atmosphere using several ballons filled with H_2. The reaction was allowed to stir overnight under a hydrogen atmosphere. The reaction mixture was filtered through Celite® and the filtrate was concentrated *in vacuo* to give a brown oil. The residue was purified by FCC (94:6 CH_2Cl_2:MeOH) to yield **15** as a colorless oil (920 mg, 63%):

FT-IR ν_{max}/cm^{-1} 3309.69 (N-H), 1718.97 (C=O), 1643.62 (C=O), 1555.81 (N-H); **^{1}H NMR** (400 MHz, $CDCl_3$) δ 5.96 (bs, 1H), 2.48 – 2.35 (m, 4H), 2.29 – 2.20 (m, 2H), 1.96 (s, 3H), 1.83 – 1.72 (m, 2H), 1.43 (s, 3H); **^{13}C NMR** (101 MHz, $CDCl_3$) δ 210.8 , 170.6, 52.4, 37.1 (2C), 36.1 (2C), 25.4, 24.4; **HRMS** (ES+) exact mass calculated for $[M+Na]^+$ ($C_9H_{15}NNaO_2$) requires *m/z* 192.0995, found *m/z* 192.1003.

(6) Synthesised according to Liang, H.; Ciufolini, M.; *J. Org. Chem.,* **2008**, 73, 4299–4301.

Synthesis and characterization of *N*-(4,4-dimethoxy-1-methylcyclohexyl)acetamide (16):

To a solution of **15** (1 eq., 2.8 mmol, 473 mg) in MeOH (6 ml) at RT was added $HC(OMe)_3$ (1.25 eq., 3.35 mmol, 0.36 g, 0.366 ml) and CSA (0.04 eq., 0.14 mmol, 32.4 mg). The reaction mixture was stirred for 30 minutes and neutralised with NEt_3. The solvent was removed *in vacuo* to give **16** as a colourless oil. The compound was sufficiently pure to use in the next step without further purification:

FT-IR ν_{max}/cm^{-1} 3296.58 (N-H), 1646.43 (C=O), 1548.84, (N-H); **^{1}H NMR** (400 MHz, $CDCl_3$) δ 5.20 (s, 1H), 3.18 (s, 3H), 3.15 (s, 3H), 1.99 (dd, *J* = 9.1, 5.6, 2H), 1.94 (s, 3H), 1.77 (dd, *J* = 9.0, 5.6, 2H), 1.61 – 1.48 (m, 4H), 1.38 (s, 3H). **^{13}C NMR** (101 MHz, $CDCl_3$) δ 169.8, 99.2, 52.8, 47.6, 47.6, 33.0 (2C), 28.3 (2C), 25.6, 24.6; **HRMS** (ES+) exact mass calculated for $[M+Na]^+$ ($C_{11}H_{21}NNaO_3$) requires *m/z* 238.1414, found *m/z* 238.1424

Synthesis and characterization of *N*-Ethyl-4,4-dimethoxy-1-methylcyclohexanamine (17):

To LiAlH4 (4 eq., 17.5 mmol, 700 mg) at -78 ^{o}C was added dry THF (5 ml). A solution of **16** (1 eq., 4.65 mmol, 1.00 g) in THF (11 ml) was added dropwise. The resulting solution was then refluxed for 22 h. The reaction mixture was cooled to -78 ^{o}C and subsequently quenched with MeOH (15 ml) and allowed to stir for 2 h at RT. The resulting mixture was diluted with ether (20 ml); Glaubers salt ($Na_2SO_4.10H_20$ 25 g) was added and stirred for another 6 h. The reaction mixture was then filtered, the filter cake washed with Et_2O (30 ml) and concentrated *in vacuo* to afford compound **17** as yellow oil (899 mg, 96%). The compound was pure and used in the next step without further purification:

FT-IR ν_{max}/cm^{-1} 3455.18 (N-H); **^{1}H NMR** (400 MHz, $CDCl_3$) δ 3.13 (s, 6H) 2.53 (q, *J* = 7.1, 2H), 1.74 – 1.66 (m, 2H), 1.62 – 1.54 (m, 2H), 1.53 – 1.46 (m, 2H), 1.44 – 1.37 (m, 2H), 1.21 (bs, 1H), 1.10 – 1.03 (m, 6H); **^{13}C NMR** (101 MHz, CDCl3) δ 99.8, 51.4, 47.6, 47.5, 35.6, 33.5 (2C), 28.3 (2C), 24.7, 16.0; **HRMS** (ES+) exact mass calculated for $[M+H]^+$ ($C_{11}H_{24}NO_2$) requires *m/z* 202.1803, found *m/z* 202.1804;

Synthesis and characterization of ethyl (2E)-4-[(4,4-dimethoxy-1-methylcyclohexyl)(ethyl)amino]but-2-enoate (18):

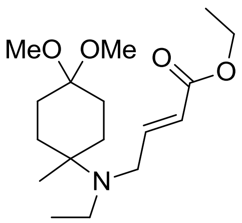

To a solution of amine **17** (1.00 eq., 1.24 mmol, 250 mg) in MeCN (6.5 ml) under an inert nitrogen atmosphere was added Cs_2CO_3 (1.10 eq., 1.37 mmol, 446 mg) and ethyl (2E)-4-bromobut-2-enoate (1.1 eq., 1.37 mmol, 330 mg). The reaction was warmed to 40 °C for 18 h. The reaction mixture was filtered and concentrated *in vacuo*. The residue was purified by FCC to give compound **18** as a yellow oil (293 mg, 75%):

FT-IR ν_{max}/cm^{-1} 1717.92 (C=O), 1655.18 (C=C); **^{1}H NMR** (400 MHz, $CDCl_3$) δ 7.01 (dt, *J* = 15.5, 5.8, 1H), 5.95 (d, *J* = 15.6, 1H), 4.17 (q, *J* = 7.1, 2H), 3.28 (d, *J* = 4.5, 2H), 3.17 (s, 3H), 3.15 (s, 3H), 2.62 – 2.52 (m, 2H), 1.82 – 1.73 (m, 2H), 1.72 – 1.63 (m, 2H), 1.61 – 1.52 (m, 2H), 1.40 – 1.31 (m, 2H), 1.28 (t, *J* = 7.1, 3H), 0.98 (t, *J* = 6.8, 3H), 0.94 (s, 3H). **^{13}C NMR** (101 MHz, $CDCl_3$) δ 166.7, 150.2, 120.8, 99.9, 60.2, 56.1, 49.2, 47.5 (2C), 42.5, 33.0 (2C), 28.2 (2C), 20.6, 15.2, 14.3; **HRMS** (ES+) exact mass calculated for $[M+Na]^+$ ($C_{17}H_{31}NNaO_4$) requires *m/z* 336.2151, found *m/z* 336.2132.

Ethyl (2*E*)-4-[ethyl(1-methyl-4-oxocyclohexyl)amino]but-2-enoate (2i):

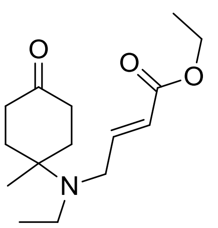

General Procedure D was applied to dimethyl acetal **18** (0.677 mmol, 241 mg). The residue was purified by FCC (60:40 PE EtOAc to give the title compound the title compound **2i** as a pale yellow oil (173 mg, 82%):

FT-IR ν_{max}/cm^{-1} 1710.56 (C=O) 1653.92 (C=C); **^{1}H NMR** (400 MHz, $CDCl_3$) δ 6.99 (dt, *J* = 15.6, 5.8, 1H), 5.94 (d, *J* = 15.7, 1H), 4.15 (q, *J* = 7.1, 2H), 3.34 (d, *J* = 5.5, 2H), 2.67 – 2.51 (m, 4H), 2.09 (m, 4H), 1.64 – 1.54 (m, 2H), 1.25 (t, *J* = 7.1, 3H), 1.08 – 0.96 (m, 6H); **^{13}C NMR** (101 MHz, $CDCl_3$) δ 211.9, 166.4, 149.3, 121.2, 60.3, 55.9, 49.3, 42.8, 37.0 (2C), 36.5 (2C), 20.6, 15.2, 14.2; **HRMS** (ES+) exact mass calculated for $[M+Na]^+$ ($C_{15}H_{25}NNaO_3$) requires *m/z* 290.1727, found *m/z* 290.1730.

Synthesis and characterization of 2j

Synthetic scheme:

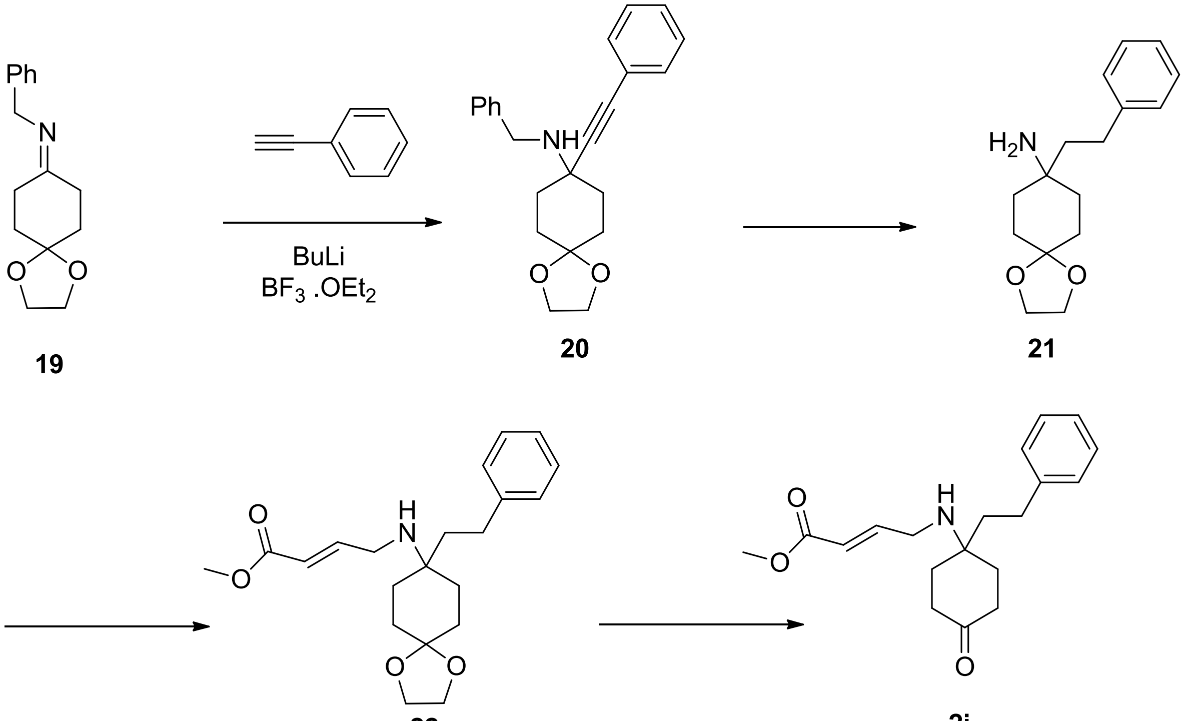

19 20 21 22 2j

N-benzyl-8-(phenylethynyl)-1,4-dioxaspiro[4.5]decan-8-amine (20):

According to a modified literature procedure.[7] To a solution of imine **19**[8] (1 eq., 4.06 mmol, 1 g) in THF (0.1 M) was added $BF_3.OEt_2$ (2eq., 8.16 mmol, 1.151 g) at room temperature and immediately cooled to -78 °C. The solution was stirred for 10 minutes to allow for the imine-boron complex to form. Separately, to a solution of phenylacetylene (3 eq., 12.2 mmol, 1.26 g) in THF (0.5 M) at -78 °C was added BuLi (1.6 M THF; 3.2 eq., 13.1 mol, 8.16 ml) dropwise and allowed to stir for 5 minutes. The resulting slurry was added to the imine solution above *via* cannula at -78 °C, washing with THF (2ml) to aid transfer. The reaction was allowed to stir at -78 °C for 30 minutes and then warmed to RT, stirred for another 2 h at RT and quenched with sat. NH_4Cl. The solution was extracted with CH_2Cl_2 and the combined organic extracts were concentrated *in vacuo* and purified by FCC (PE:EtOAc 70:30) to give compound **20** (77%, 1.093g).

(7) Ma, Y.; Lobkovsky, E.; Collum, D. B. *J. Org. Chem.*, **2005**, 70, 2335–2337
(8) **19** was synthesised according to Diaba, F.; Ricou, E.; Bonjoch, J. *Org. Lett.*, **2007**, 9, 2633–2636

FT-IR ν_{max}/cm^{-1} 1947, 1738, 1722, 1688; **^{1}H NMR** (400 MHz, $CDCl_3$) δ 7.51 – 7.41 (m, 4H), 7.38 – 7.24 (m, 6H), 4.00 (s, 2H), 3.99 – 3.98 (m, 4H), 2.10 – 2.02 (m, 2H), 1.97 – 1.84 (m, 6H), 1.44 (bs, 1H). **^{13}C NMR** (101 MHz, $CDCl_3$) δ 141.0, 131.7, 128.5 (2C), 128.4 (2C), 128.3 (2C), 128.0, 127.0, 123.4, 108.6, 92.7, 84.3, 64.3, 64.3, 54.1, 48.5, 35.5 (2C), 31.4 (2C); **HRMS** (ES+) exact mass calculated for $[M+H]^+$ ($C_{23}H_{26}NO_2$) requires *m/z* 348.1958, found *m/z* 348.1953

8-phenethyl-1,4-dioxaspiro[4.5]decan-8-amine (21):

To a solution of **20** (1 eq. 3.12 mmol, 1.08 g) in methanol (0.1 M) was added $Pd(OH)_2$ (20% wt, 50% wet; 0.1 eq., 0.3 mmol, 0.44 g). The resulting suspension was purged and regassed with nitrogen under vaccum (4x) and subsequently purged and regassed with hydrogen under vacuum (4x). The reaction was left under hydrogen pressure (4 ballons within eachother) for 12 hours. The resulting suspension was filtered through GFA filter paper to remove the $Pd(OH)_2$ and concentrated in vacuo to give title compound **21** as a pure yellow oil in (91%, 741 mg).

FT-IR ν_{max}/cm^{-1} 2944, 1739; **^{1}H NMR** (400 MHz, $CDCl_3$) δ 7.20 (dd, *J* = 9.8, 5.0 Hz, 2H), 7.14 – 7.07 (m, 3H), 3.90 – 3.83 (m, 4H), 2.63 – 2.56 (m, 2H), 1.80 – 1.46 (m, 12H). **^{13}C NMR** (101 MHz, $CDCl_3$) δ 142.7, 128.4 (2C), 128.4 (2C), 125.7, 108.7, 64.3, 64.2, 50.3, 44.5, 35.7 (2C), 30.8 (2C), 29.9. **HRMS** (ES+) exact mass calculated for $[M+H]^+$ ($C_{16}H_{24}NO_2$) requires *m/z* 262.1802, found *m/z* 262.1792;

(E)-methyl 4-((8-phenethyl-1,4-dioxaspiro[4.5]decan-8-yl)amino)but-2-enoate (22):

To a stirred solution of the amine **21** (1.0 eq., 2.47 mmol, 646 mg) in acetonitrile (0.4 M) at RT was added K_2CO_3 (1.1 eq., 2.97 mmol, 410 mg) and methyl 4-bromocrotonate (85%; 1.1 eq., 2.7 mmol, 523 g, 0.38 ml). The resulting mixture was stirred at RT for 14 h. The reaction mixture was subsequently filtered through Celite®to remove residual solid particles and the filtrate concentrated *in vacuo*. The residue was purified by FCC (PE:EtOAc 70:30), to yield the title compounds title compound **22** as a yellow oil (86%, 760 mg).

FT-IR ν_{max}/cm^{-1} 2931, 2847, 1719, 1658; **^{1}H NMR** (400 MHz, $CDCl_3$) δ 7.32 – 7.27 (m, 2H), 7.21 – 7.16 (m, 3H), 7.08 (dt, *J* = 15.6, 5.1 Hz, 1H), 6.10 (dt, *J* = 15.6, 1.8 Hz, 1H), 4.09 – 3.89 (m, 4H), 3.76 (s, 3H), 3.32 (dd, *J* = 5.2, 1.8 Hz, 2H), 2.63 – 2.49 (m, 2H), 1.87 (td, *J* = 12.1, 4.2 Hz, 2H), 1.79 – 1.53 (m, 8H), 1.04 (s, 1H). **^{13}C NMR** (101 MHz, $CDCl_3$) δ 167.0, 148.2, 142.7, 128.5 (2C), 128.3 (2C), 125.8, 120.5, 109.0, 64.3, 64.2, 52.9, 51.5, 42.2, 39.7, 32.8 (2C), 30.4 (2C), 29.6. **HRMS** (ES+) exact mass calculated for $[M+Na]^+$ ($C_{21}H_{29}NNaO_4$+) requires *m/z* 382.1989, found *m/z* 382.1996.

(E)-methyl 4-((4-oxo-1-phenethylcyclohexyl)amino)but-2-enoate 2j

To a solution of the acetal **22** (1.0 eq., 2.56 mmol, 664 mg) in THF (0.2 M) at 0 °C was added HCl (1 M; 5 eq., 12.8 ml). The resulting solution was stirred for 12 h at RT °C. The reaction mixture was basified with NaOH (4.0 M) until a pH 10 was obtained and extracted quickly with CH_2Cl_2. The combined organic extracts were dried over Na_2SO_4, filtered and concentrated *in vacuo*. The residue was purified by FCC to yield the title compounds **2j** as a yellow oil (80%, 465 mg).

FT-IR ν_{max}/cm^{-1} 2947, 1738, 1717, 1658; **^{1}H NMR** (400 MHz, $CDCl_3$) δ 7.32 – 7.26 (m, 1H), 7.22 – 7.16 (m, 1H), 7.06 (dt, *J* = 15.7, 5.1 Hz, 1H), 6.09 (dt, *J* = 15.6, 1.9 Hz, 1H), 3.75 (s, 2H), 3.37 (dd, *J* = 5.1, 1.9 Hz, 1H), 2.72 – 2.60 (m, 1H), 2.60 – 2.52 (m, 1H), 2.26 – 2.17 (m, 1H), 2.01 – 1.92 (m, 1H), 1.75 (ddd, *J* = 19.9, 11.1, 5.0 Hz, 2H), 1.16 (bs, 1H). **^{13}C NMR** (101 MHz, $CDCl_3$) δ 211.8, 166.9, 147.4, 141.9, 128.6 (2C), 128.2 (2C), 126.1, 120.7, 53.1, 51.6, 42.3, 39.7, 36.8 (2C), 35.0 (2C), 29.7. **HRMS** (ES+) exact mass calculated for $[M+Na]^+$ ($C_{19}H_{25}NNaO_3$+) requires *m/z 338.1727*, found *m/z 338.1736*.

Synthetic scheme for the synthesis of 2k

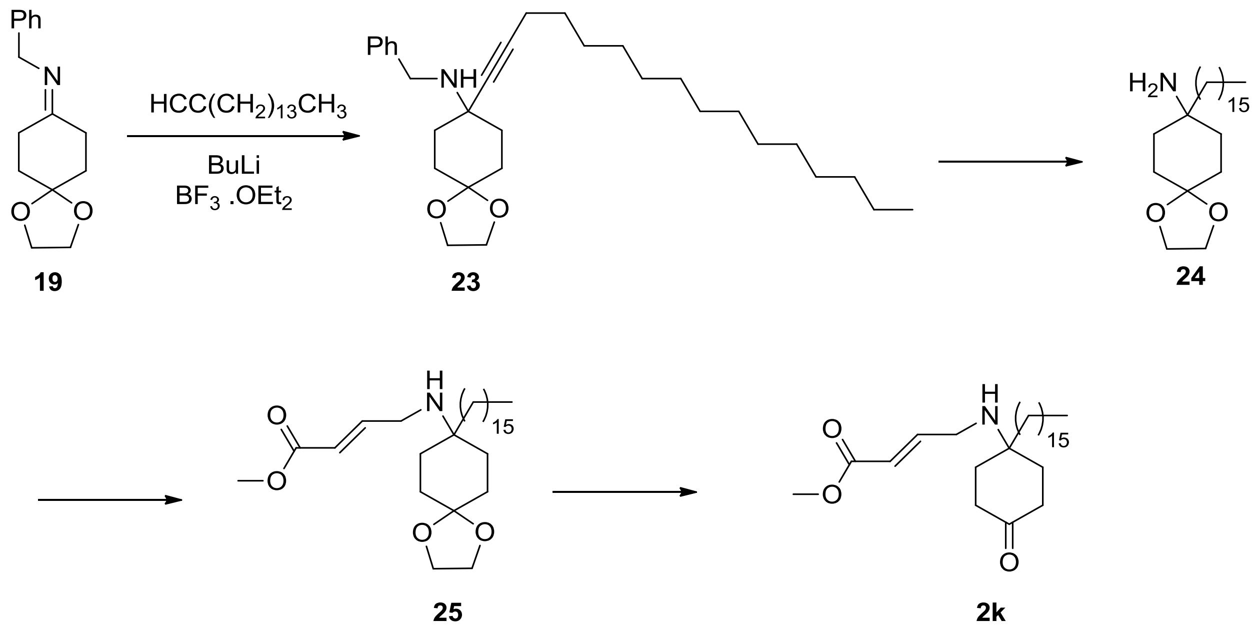

N-benzyl-8-(hexadec-1-yn-1-yl)-1,4-dioxaspiro[4.5]decan-8-amine (23):

According to a modified literature procedure. [9] To a solution of imine **19**[8] (1 eq., 1.53 mmol, 375 mg) in THF (0.1 M) was added $BF_3.OEt_2$ (2eq. 3.1 mmol, 432 mg) at room temperature and immediately cooled to -78 °C. The solution was stirred for 10 minutes to allow for the imine-boron complex to form. Separately, to a solution of alkyne 1-hexadecyne (2 eq., 3.1 mmol, 680 mg) in THF (0.5 M) at -78 °C was added BuLi (1.6 M THF; 2.2 eq., 3.37 mol, 2.1 ml) dropwise and allowed to stir for 5 minutes. The resulting slurry was added to the imine solution above *via* cannula at -78 °C washing with THF (2 ml) to aid transfer. The reaction was allowed to stir at -78 °C for 30 minutes and then warmed to RT, stirred for another 2 h at RT and quenched with sat. NH_4Cl. The solution was extracted with CH_2Cl_2 and the combined organic extracts were concentrated *in vacuo* and purified by FCC (PE:EtOAc 80:20), title compound **23** (64%, 461 mg).

FT-IR ν_{max}/cm^{-1} 2929, 1742; **^{1}H NMR** (400 MHz, $CDCl_3$) δ 7.40 – 7.36 (m, 2H), 7.34 – 7.29 (m, 2H), 7.27 – 7.22 (m, 1H), 3.95 (t, *J* = 2.6 Hz, 4H), 3.88 (s, 2H), 2.25 (t, *J* = 7.0 Hz, 2H), 1.93 – 1.85 (m, 3H), 1.79 (m, 5H), 1.58 – 1.49 (m, 2H), 1.42 (dd, *J* = 14.2, 7.1 Hz, 2H), 1.36 – 1.20 (m, 22H), 0.89 (t, *J* = 6.9 Hz, 3H). **^{13}C NMR** (101 MHz, $CDCl_3$) δ 141.1, 128.4, 128.3, 126.8, 108.7, 64.3, 64.2, 53.6, 48.3, 35.7 (2C), 31.9 (2C), 31.4, 29.7-29.6 (10C), 29.4, 29.2, 29.2, 28.9, 22.7, 18.7, 14.1; **HRMS** (ES+) exact mass calculated for $[M+H]^+$ ($C_{31}H_{50}NO_2$) requires *m/z* 468.3836, found *m/z* 468.3830

---

(9) Ma, Y.; Lobkovsky, E.; Collum, D. B. *J. Org. Chem.*, **2005**, 70, 2335–2337

N-benzyl-8-hexadecyl-1,4-dioxaspiro[4.5]decan-8-amine (24):

To a solution of **23** (1 eq. 0.91 mmol, 424 mg) in methanol (0.1 M) was added $Pd(OH)_2$ (20% wt, 50% wet; 0.1 eq., 0.09 mmol, 127 mg). The resulting suspension was purged and regassed with nitrogen under vaccum (4x) and subsequently purged and regassed with hydrogen under vacuum (4x). The reaction was left under hydrogen pressure (4 ballons within eachother) for 12 hours. The resulting suspension was filtered through GFA filter paper to remove the $Pd(OH)_2$ and concentrated in vacuo to give title compound **24** as a pure colourless oil in (87%, 300 mg).

FT-IR ν_{max}/cm^{-1} 2916, 2847, 1738; **^{1}H NMR** (400 MHz, $CDCl_3$) δ 3.90 – 3.83 (m, 4H), 1.76 – 1.66 (m, 2H), 1.59 – 1.49 (m, 4H), 1.46 – 1.37 (m, 2H), 1.31 (d, *J* = 8.9 Hz, 2H), 1.20 (d, *J* = 9.2 Hz, 30H), 0.81 (t, *J* = 6.8 Hz, 3H). **^{13}C NMR** (101 MHz, $CDCl_3$) δ 108.9, 64.2 (2C), 50.0, 35.7 (2C), 31.9, 30.9 (2C), 30.4, 29.71- 29.68 (10C), 29.4, 23.2, 22.7, 14.1. **HRMS** (ES+) exact mass calculated for $[M+H]^+$ ($C_{24}H_{48}NO_2$) requires *m/z 382.3680*, found *m/z 382.3678*

(E)-methyl 4-((8-hexadecyl-1,4-dioxaspiro[4.5]decan-8-yl)amino)but-2-enoate (25):

To a stirred solution of the amine **24** (1.0 eq., 0.772 mmol, 295 mg)) in acetonitrile (0.4 M) at RT was added K_2CO_3 (1.1 eq., 0.93 mmol, 128 mg) and methyl 4-bromocrotonate (85%; 1.1 eq., 0.85 mmol, 179 mg, 0.12 ml). The resulting mixture was stirred at RT for 14 h. The reaction mixture was subsequently filtered through Celite® to remove residual solid particles and the filtrate concentrated *in vacuo*. The residue was purified by FCC to yield the title compounds title compound **25** as a yellow oil (83%, 308 mg).

FT-IR ν_{max}/cm^{-1} 2926, 2717, 1658; **^{1}H NMR** (400 MHz, $CDCl_3$) δ 6.97 (dt, *J* = 15.6, 5.1 Hz, 1H), 5.98 (dt, *J* = 15.6, 1.6 Hz, 1H), 3.86 (t, *J* = 2.0 Hz, 4H), 3.66 (s, 3H), 3.16 (dd, *J* = 5.1, 1.8 Hz, 2H), 1.83 – 1.70 (m, 2H), 1.56 – 1.39 (m, 6H), 1.27 (dd, *J* = 9.9, 6.1 Hz, 2H), 1.25 – 1.06 (m, 29H), 0.81 (t, *J* = 6.8 Hz, 3H). **^{13}C NMR** (101 MHz, $CDCl_3$) δ 167.1, 148.4, 120.3, 109.1, 64.2, 64.2, 52.7, 51.5, 42.2, 37.5, 32.8 (2C), 31.9, 30.3 (2C), 30.2, 29.70-29.68 (9C), 29.4, 22.8, 22.7, 14.1. **HRMS** (ES+) exact mass calculated for $[M+Na]^+$ ($C_{29}H_{53}NNaO_4+$) requires *m/z 502.3867*, found *m/z 502.3864*.

(E)-methyl 4-((1-hexadecyl-4-oxocyclohexyl)amino)but-2-enoate (2k):

To a solution of the acetal **25** (1.0 eq., 0.577 mmol, 277 mg) in THF (0.2 M) at 0 °C was added HCl (1 M; 5 eq., 2.891 ml). The resulting solution was stirred for 12 h at RT °C. The reaction mixture was basified with NaOH (4.0 M) until a pH 10 was obtained and extracted quickly with CH_2Cl_2. The combined organic extracts were dried over Na_2SO_4, filtered and concentrated *in vacuo*. The residue was purified by FCC to yield the title compounds **2k** as a yellow oil (89%, 233 mg).

FT-IR ν_{max}/cm^{-1} 2922, 2852, 1716, 1659; **^{1}H NMR** (400 MHz, $CDCl_3$) δ 7.04 (dt, *J* = 15.6, 5.1 Hz, 1H), 6.06 (dd, *J* = 15.6, 1.6 Hz, 1H), 3.72 (s, 3H), 3.32 – 3.26 (m, 2H), 2.62 (td, *J* = 13.9, 5.9 Hz, 2H), 2.14 (d, *J* = 14.8 Hz, 2H), 1.89 – 1.81 (m, 2H), 1.62 (td, *J* = 13.4, 4.6 Hz, 2H), 1.40 (dd, *J* = 10.0, 6.0 Hz, 2H), 1.29 – 1.15 (m, 29H), 0.85 (t, *J* = 6.2 Hz, 3H). **^{13}C NMR** (101 MHz, $CDCl_3$) δ 212.0, 166.9, 147.7, 120.5, 52.9, 51.5, 42.2, 37.6, 36.8 (2C), 35.0, 31.9, 30.1 (2C), 29.66-29.59 (9C), 29.3, 23.0, 22.7, 14.1. **HRMS** (ES+) exact mass calculated for $[M+Na]^+$ ($C_{27}H_{49}NNaO_3$+) requires *m/z 458.3605*, found *m/z 458.3604*.

Synthesis and characterization of substrates 2l-q:

General sequence for synthesis of **2l-q** via an amine alkylation and subsequent acetal deprotection:

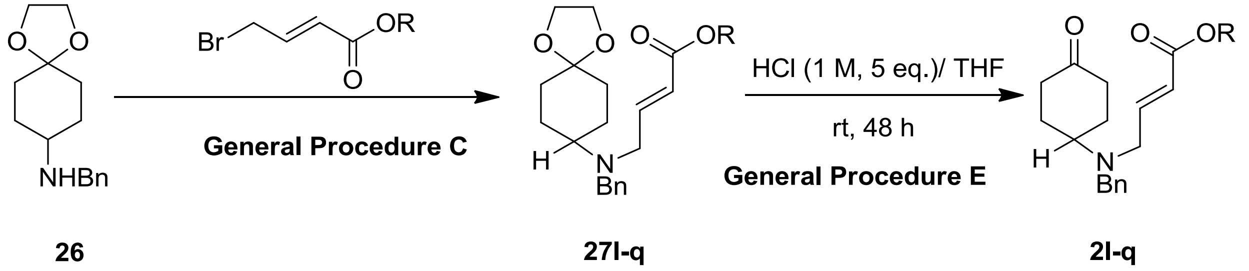

General procedure E - Deprotection of the cyclic acetal: To a solution **27l-q** (1eq) in THF (0.2M) was added 1 M HCl (5eq) and the reaction mixture was stirred at room temperature for 48 h. Saturated NaOH solution was added until pH 10 was obtained. The resulting solution was extracted with ethyl acetate (3x) and the combined organic layer was washed with water, dried over Na_2SO_4 and concentrated under reduced pressure. The residue was purified by flash column chromatography (PE/Et_2O = 1:1) on silica gel to give the pure product (**2l-q**).

Synthesis and characterization of (*E*)-ethyl 4-(benzyl(1,4-dioxaspiro[4.5]decan-8-yl)amino)but-2-enoate (27l):

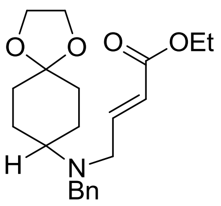

27l

27l was obtained according to the **general procedure C** (step1) on **26** (300mg, 1.21 mmol) using ethyl 4-bromocrotonate. The crude reaction mixture was purified by flash column chromatography (PE/Et_2O = 1:1) to afford 365 mg (84%) of product as a viscous oil.

FT-IR ν_{max}/cm^{-1} 2937, 1717, 1657; **^{1}H NMR** (400 MHz, $CDCl_3$) δ 7.36 – 7.25 (m, 4H), 7.22 (ddd, *J* = 7.0, 3.8, 1.4 Hz, 1H), 6.90 (dt, *J* = 15.7, 5.6 Hz, 1H), 5.99 (dt, *J* = 15.7, 1.7 Hz, 1H), 4.17 (q, *J* = 7.1 Hz, 2H), 3.92 (s, 4H), 3.63 (s, 2H), 3.25 (dd, *J* = 5.6, 1.6 Hz, 2H), 2.58 (tt, *J* = 11.5, 3.3 Hz, 1H), 1.89 – 1.71 (m, 4H), 1.62 (ddd, *J* = 24.7, 12.5, 3.3 Hz, 2H), 1.55 – 1.44 (m, 2H), 1.28 (t, *J* = 7.1 Hz, 3H). **^{13}C NMR** (100 MHz, $CDCl_3$) δ 166.5, 147.9, 140.2, 128.2 (2C), 128.1 (2C), 126.7, 121.8, 108.4, 64.2, 64.1, 60.2, 57.6, 54.2, 50.9, 33.9 (2C), 25.3 (2C), 14.2; HRMS (ESI+) calcd. for $C_{21}H_{30}NO_4$ $[M+H]^+$ 360.2169 found 360.2167.

Synthesis and characterization of (*E*)-ethyl 3-(benzyl(4-oxocyclohexyl)amino)acrylate (2l):

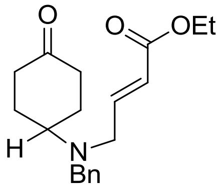

2l

2l was obtained according to the **general procedure E** on **27l** (0.6 mmol) as starting material. The crude reaction mixture was purified by flash column chromatography (PE/Et_2O = 1:1) to afford 153 mg (81%) of product as a viscous oil.

FT-IR ν_{max}/cm^{-1} 2938, 1715; **^{1}H NMR** (400 MHz, $CDCl_3$) δ 7.37 – 7.21 (m, 5H), 6.93 (dt, *J* = 15.7, 5.7 Hz, 1H), 6.02 (dt, *J* = 15.7, 1.6 Hz, 1H), 4.18 (q, *J* = 7.1 Hz, 2H), 3.66 (s, 2H), 3.28 (dd, *J* = 5.7, 1.5 Hz, 2H), 3.02 (tt, *J* = 11.1, 3.3 Hz, 1H), 2.43 (d, *J* = 14.6 Hz, 2H), 2.37 – 2.23 (m, 2H), 2.12 (ddd, *J* = 12.5, 5.9, 2.9 Hz, 2H), 1.79 (ddd, *J* = 24.4, 12.8, 4.6 Hz, 2H), 1.29 (t, *J* = 7.1 Hz, 3H). **^{13}C NMR** (100 MHz, $CDCl_3$) δ 210.7, 166.3, 147.0, 139.5, 128.3 (2C), 128.2 (2C), 127.0, 122.3, 60.3, 56.6, 54.2, 50.9, 39.7 (2C), 27.6 (2C), 14.2; **HRMS** (ESI+) calcd. for $C_{19}H_{25}NNaO_3$ $[M+Na]^+$ 338.1727 found 338.1713.

Synthesis and characterization of (*E*)-benzyl 4-(benzyl(1,4-dioxaspiro[4.5]decan-8-yl)amino)but-2-enoate (27m):

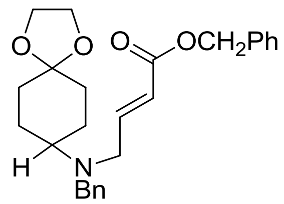

27m

27m was obtained according to the **general procedure C** (step1) on **26** (300mg, 1.21 mmol) using benzyl 4-bromocrotonate **10a**. The crude reaction mixture was purified by flash column chromatography (PE/Et_2O = 1:1) to afford 315 mg of **27m** (62%) as a viscous oil.

FT-IR ν_{max}/cm^{-1} 2938, 1715; **^{1}H NMR** (500 MHz, $CDCl_3$) δ 7.45 – 7.15 (m, 10H), 6.96 (dt, *J* = 10.3, 4.9 Hz, 1H), 6.06 (d, *J* = 1.5 Hz, 1H), 5.17 (s, 2H), 3.92 (s, 4H), 3.64 (s, 2H), 3.26 (d, *J* = 4.3 Hz, 2H), 2.58 (t, *J* = 11.0 Hz, 1H), 1.88 – 1.71 (m, 4H), 1.69 – 1.55 (m, 2H), 1.55 – 1.42 (m, 2H). **^{13}C NMR** (125 MHz, $CDCl_3$) δ 166.30, 148.79, 140.1, 136.0 128.5 (2C), 128.3 (2C), 128.2 (4C), 128.1, 126.7, 121.4, 108.4, 66.1, 64.2, 64.2, 57.7, 54.3, 51.0, 33.9 (2C), 25.3 (2C); **HRMS** (ESI+) calcd. for $C_{26}H_{32}NO_4$ $[M+H]^+$ 422.2326 found 422.2324.

Synthesis and characterization of (*E*)-benzyl 4-(benzyl(4-oxocyclohexyl)amino)but-2-enoate (2m):

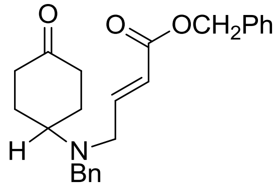

2m

2m was obtained according to the **general procedure E** using **27m** (0.6 mmol) as a starting material. The crude reaction mixture was purified by flash column chromatography (PE/Et_2O = 1:1) to afford 176 mg (78%) of product as a viscous oil.

FT-IR ν_{max}/cm^{-1} 2938, 1715; **^{1}H NMR** (400 MHz, $CDCl_3$) δ 7.45 – 7.19 (m, 10H), 7.00 (dt, *J* = 15.7, 5.6 Hz, 1H), 6.09 (d, *J* = 15.7 Hz, 1H), 5.19 (s, 2H), 3.67 (s, 2H), 3.30 (dd, *J* = 5.6, 1.3 Hz, 2H), 3.03 (ddd, *J* = 11.0, 7.2, 3.2 Hz, 1H), 2.44 (d, *J* = 14.5 Hz, 2H), 2.41 – 2.23 (m, 2H), 2.18 – 2.05 (m, 2H), 1.79 (qd, *J* = 12.8, 4.5 Hz, 2H). **^{13}C NMR** (100 MHz, $CDCl_3$) δ 210.7, 166.1, 147.9, 139.5, 135.9, 128.5 (2C), 128.4 (2C), 128.3 (4C), 128.2, 127.1, 121.9, 66.2, 56.8, 54.4, 51.0, 39.8 (2C), 27.7 (2C)**.** **HRMS** (ESI+) calcd. for $C_{24}H_{28}NO_3$ $[M+H]^+$ 378.2064, found 378.2049.

Synthesis and characterization of (*E*)-cyclohexyl 4-(benzyl(1,4-dioxaspiro[4.5]decan-8-yl)amino)but-2-enoate (27n):

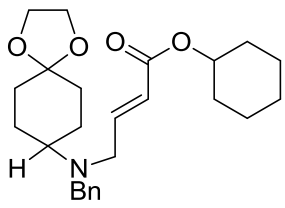

27n

27n was obtained according to the **general procedure C** (step1) on **26** (300mg, 1.21 mmol) using (*E*)-cyclohexyl 4-bromobut-2-enoate (**10c**) as a starting material. The crude reaction mixture was purified by flash column chromatography (PE/Et_2O = 1:1) to afford 289 mg (58%) of product as a viscous oil.

FT-IR ν_{max}/cm^{-1} 2936, 1713, 1655; **^{1}H NMR** (^{1}H NMR (500 MHz, $CDCl_3$) δ 7.37 – 7.27 (m, 4H), 7.22 (t, *J* = 6.9 Hz, 1H), 6.88 (dt, *J* = 10.6, 5.0 Hz, 1H), 5.97 (d, *J* = 15.7 Hz, 1H), 4.94 – 4.66 (m, 1H), 3.92 (s, 4H), 3.64 (s, 2H), 3.25 (d, *J* = 4.3 Hz, 2H), 2.59 (t, *J* = 10.8 Hz, 1H), 2.03 – 1.14 (m, 18H); **^{13}C NMR** (125 MHz, $CDCl_3$) δ 165.9, 147.5, 140.2, 128.3 (2C), 128.2 (2C), 126.7, 122.3, 108.5, 72.4, 64.3, 64.2, 57.7, 54.3, 51.1, 33.9 (2C), 31.6 (2C), 25.4, 25.3 (2C), 23.7 (2C); **HRMS** (ESI+) calcd. for $C_{25}H_{36}NO_4$ $[M+H]^+$ 414.2639 found 414.2644.

Synthesis and characterization of (*E*)-cyclohexyl 4-(benzyl(4-oxocyclohexyl)amino)but-2-enoate (2n):

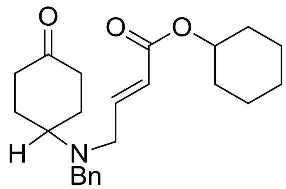

2n

2n was obtained according to the **general procedure E** using **27n** (0.6 mmol) as a starting material. The crude reaction mixture was purified by flash column chromatography (PE/Et_2O = 1:1) to afford 148 mg (67%) of product as a viscous oil.

FT-IR ν_{max}/cm^{-1} 2936, 2859, 1713; **^{1}H NMR** (400 MHz, $CDCl_3$) δ 7.43 – 7.27 (m, 4H), 7.27 – 7.18 (m, 1H), 6.90 (dt, *J* = 15.7, 5.7 Hz, 1H), 6.12 – 5.85 (m, 1H), 4.89 – 4.69 (m, 1H), 3.65 (s, 2H), 3.27 (dd, *J* = 5.7, 1.4 Hz, 2H), 3.02 (tt, *J* = 11.0, 3.2 Hz, 1H), 2.42 (d, *J* = 14.6 Hz, 2H), 2.36 – 2.24 (m, 2H), 2.17 – 2.05 (m, 2H), 1.91 – 1.63 (m, 6H), 1.59 – 1.48 (m, 1H), 1.48 – 1.17 (m, 5H); **^{13}C NMR** (100 MHz, $CDCl_3$) δ 210.6, 165.6, 146.5, 139.5, 128.2 (2xC), 128.1 (2C), 126.9, 122.7, 72.4, 56.6, 54.2, 50.9, 39.6 (2C), 31.5 (2C), 27.5 (2C), 25.2, 23.6 (2C); **HRMS** (ESI+) calcd. for $C_{23}H_{31}NNaO_3$ $[M+Na]^+$ 392.2196 found 392.2184.

Synthesis and characterization of (*E*)-isopropyl 4-(benzyl(1,4-dioxaspiro[4.5]decan-8-yl)amino)but-2-enoate (27o):

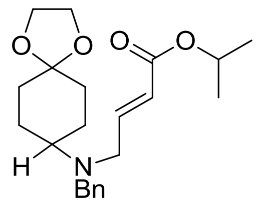

27o

27o was obtained according to the **general procedure C** (step1) on **26** (300mg, 1.21 mmol) using (*E*)-isopropyl 4-bromobut-2-enoate **10d** as a starting material. The crude reaction mixture was purified by flash column chromatography (PE/Et_2O = 1:1) to afford 248 mg (55%) of product as a viscous oil.

FT-IR ν_{max}/cm^{-1} 2937, 1713, 1655; **^{1}H NMR** (200 MHz, $CDCl_3$) δ 7.46 – 7.10 (m, 5H), 6.87 (dt, *J* = 15.7, 5.6 Hz, 1H), 5.96 (dt, *J* = 15.7, 1.6 Hz, 1H), 5.04 (hept, *J* = 6.3 Hz, 1H), 3.92 (s, 4H), 3.63 (s, 2H), 3.25 (dd, *J* = 5.6, 1.5 Hz, 2H), 2.73 – 2.42 (m, 1H), 1.98 – 1.39 (m, 8H), 1.25 (d, *J* = 6.3 Hz, 6H); **^{13}C NMR** (100 MHz, $CDCl_3$) δ 166.1, 147.7, 140.2, 128.3 (2C), 128.2 (2C), 126.7, 122.2, 108.5, 67.5, 64.2, 64.1, 57.6, 54.2, 51.0, 33.9 (2C), 25.3 (2C), 21.8 (2C); **HRMS** (ESI+) $[M+H]^+$ calcd. for $C_{22}H_{32}NO_4$ $[M+H]^+$ 374.2326 found 374.2330.

Synthesis and characterization of (*E*)-isopropyl 4-(benzyl(4-oxocyclohexyl)amino)but-2-enoate (2o):

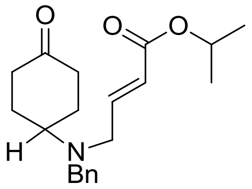

2o

2o was obtained according to the **general procedure E** using **27o** (247 mg) as a starting material. The crude reaction mixture was purified by flash column chromatography (PE/Et_2O = 1:1) to afford 138 mg (70%) of product as a viscous oil.

FT-IR ν_{max}/cm^{-1} 2935, 1714; **^{1}H NMR** (500 MHz, $CDCl_3$) δ 7.38 – 7.28 (m, 4H), 7.23 (t, *J* = 7.0 Hz, 1H), 6.90 (dt, *J* = 15.7, 5.7 Hz, 1H), 5.98 (dt, *J* = 15.7, 1.6 Hz, 1H), 5.04 (hept, *J* = 6.2 Hz, 1H), 3.66 (s, 2H), 3.28 (dd, *J* = 5.6, 1.1 Hz, 2H), 3.11 – 2.92 (m, 1H), 2.48 – 2.37 (m, 2H), 2.37 – 2.23 (m, 2H), 2.16 – 2.02 (m, 2H), 1.78 (qd, *J* = 12.8, 4.5 Hz, 2H), 1.25 (d, *J* = 6.3 Hz, 6H); **^{13}C NMR** (125 MHz, $CDCl_3$) δ 210.6, 165.7, 146.6, 139.5, 128.3 (2C), 128.2 (2C), 127.0, 122.7, 67.6, 56.6, 54.2, 50.9, 39.7 (2C), 27.6 (2C), 21.7 (2C); **HRMS** (ESI+) calcd. for $C_{20}H_{28}NO_3$ $[M+H]^+$ 330.2064 found 330.2056.

Synthesis and characterization of (*E*)-methyl 4-(benzyl(1,4-dioxaspiro[4.5]decan-8-yl)amino)but-2-enoate (27pq):

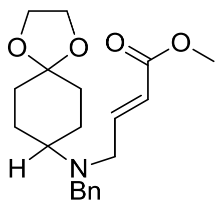

27p

27p was obtained according to the **general procedure C** (step1) on **26** (300mg, 1.21 mmol) using (*E*)-methyl 4-bromobut-2-enoate as a starting material. The crude reaction mixture was purified by flash column chromatography (PE/Et_2O = 1:1) to afford 246 mg (59%) of product as a viscous oil.

FT-IR ν_{max}/cm^{-1} 2947, 2360, 1722; **^{1}H NMR** (500 MHz, $CDCl_3$) δ 7.39 – 7.15 (m, 5H), 6.92 (dt, *J* = 15.6, 5.5 Hz, 1H), 6.01 (d, *J* = 15.7 Hz, 1H), 3.92 (s, 4H), 3.72 (s, 3H), 3.64 (s, 2H), 3.26 (d, *J* = 4.6 Hz, 2H), 2.57 (dd, *J* = 15.6, 7.4 Hz, 1H), 1.86 – 1.72 (m, 4H), 1.67 – 1.59 (m, 2H), 1.56 – 1.44 (m, 2H).; **^{13}C NMR** (125 MHz, $CDCl_3$) δ 166.9, 148.4, 140.2, 128.2 (2C), 128.2 (2C), 126.8, 121.3, 108.5, 64.3, 64.2, 57.6, 54.2, 51.4, 50.9, 33.9 (2C), 25.4 (2C). **HRMS** (ESI+) calcd. for $C_{20}H_{28}NO_4$ $[M+H]^+$ 346.2013 found 346.2019.

Synthesis and characterization of (*E*)-methyl 4-(benzyl(4-oxocyclohexyl)amino)but-2-enoate (2q):

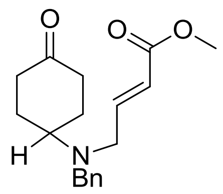

2p

2p was obtained according to the general procedure E using **27p** (207 mg) as a starting material. The crude reaction mixture was purified by flash column chromatography (PE/Et_2O = 1:1) to afford 110 mg (61%) of product as a viscous oil.

FT-IR ν_{max}/cm^{-1} 2949, 1717; **^{1}H NMR** (400 MHz, $CDCl_3$) δ 7.37 – 7.27 (m, 4H), 7.27 – 7.19 (m, 1H), 6.94 (dt, *J* = 15.7, 5.7 Hz, 1H), 6.02 (dt, *J* = 15.7, 1.5 Hz, 1H), 3.72 (s, 3H), 3.66 (s, 2H), 3.28 (dd, *J* = 5.7, 1.5 Hz, 2H), 3.01 (tt, *J* = 11.1, 3.2 Hz, 1H), 2.47 – 2.37 (m, 2H), 2.37 – 2.24 (m, 2H), 2.18 – 2.04 (m, 2H), 1.78 (qd, *J* = 12.8, 4.6 Hz, 2H). **^{13}C NMR** (100 MHz, $CDCl_3$) δ 210.6, 166.6, 147.3, 139.4, 128.3 (2C), 128.1 (2C), 127.0, 121.8, 56.6, 54.2, 51.4, 50.8, 39.7 (2C), 27.6 (2C). **HRMS** (ESI+) calcd. for $C_{18}H_{23}NNaO_3$ $[M+Na]^+$ 324.1570 found 324.1560.

Synthesis and characterization of (*E*)-phenyl 4-(benzyl(1,4-dioxaspiro[4.5]decan-8-yl)amino)but-2-enoate (27q):

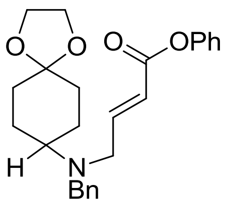

27q

27q was obtained according to the **general procedure C** (step1) on **26** (300mg, 1.21 mmol) using (*E*)-phenyl 4-bromobut-2-enoate (**10b**) as a starting material. The crude reaction mixture was purified by flash column chromatography (PE/Et_2O = 1:1) to afford 320 mg (65%) of product as a viscous oil.

FT-IR ν_{max}/cm^{-1} 2937, 1713, 1655; **^{1}H NMR** (200 MHz, $CDCl_3$) δ 7.56 – 6.94 (m, 11H), 6.21 (dt, *J* = 15.6, 1.6 Hz, 1H), 3.94 (s, 4H), 3.69 (s, 2H), 3.35 (dd, *J* = 5.4, 1.6 Hz, 2H), 2.77 – 2.49 (m, 1H), 1.97 – 1.38 (m, 8H); **^{13}C NMR** (100 MHz, $CDCl_3$) δ 164.8, 150.6, 150.5, 140.1, 129.3 (2C), 128.3 (2C), 128.2 (2C), 126.6, 125.6, 121.5 (2C), 120.8, 108.4, 64.3, 64.2, 57.9, 54.5, 51.1, 33.9 (2C), 25.3 (2C), **HRMS** (ESI+) calcd. for $C_{25}H_{30}NO_4$ $[M+H]^+$ 408.2169 found 408.2170.

Synthesis and characterization of (*E*)-phenyl 4-(benzyl(4-oxocyclohexyl)amino)but-2-enoate (2q):

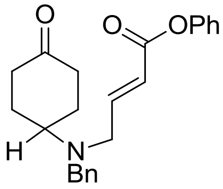

2q

FT-IR ν_{max}/cm^{-1} 2937, 1734; **2q** was obtained according to the **general procedure E** using **27q** (244 mg) as a starting material. The crude reaction mixture was purified by flash column chromatography (PE/Et_2O = 1:1) to afford 161 mg (74%) of product as a viscous oil.

^{1}H NMR (400 MHz, $CDCl_3$) δ 7.43 – 7.31 (m, 6H), 7.30 – 7.21 (m, 2H), 7.19 – 7.07 (m, 3H), 6.24 (d, *J* = 15.7 Hz, 1H), 3.72 (s, 2H), 3.38 (d, *J* = 5.3 Hz, 2H), 3.13 – 3.02 (m, 1H), 2.47 (d, *J* = 14.5 Hz, 2H), 2.41 – 2.29 (m, 2H), 2.23 – 2.11 (m, 2H), 1.83 (qd, *J* = 12.7, 4.4 Hz, 2H).; **^{13}C NMR** (100 MHz, $CDCl_3$) δ 210.6, 164.6, 150.6, 149.5, 139.4, 129.3 (2C), 128.4 (2C), 128.3 (2C), 127.1, 125.7, 121.5 (2C), 121.3, 56.9, 54.5, 51.1, 39.8 (2C), 27.6 (2C); **HRMS** (ESI+) calcd. for $C_{23}H_{26}NO_3$ $[M+H]^+$ 364.1907 found 364.1907.

Synthesis and characterization of substrates 2r and 2t

Synthetic Scheme:

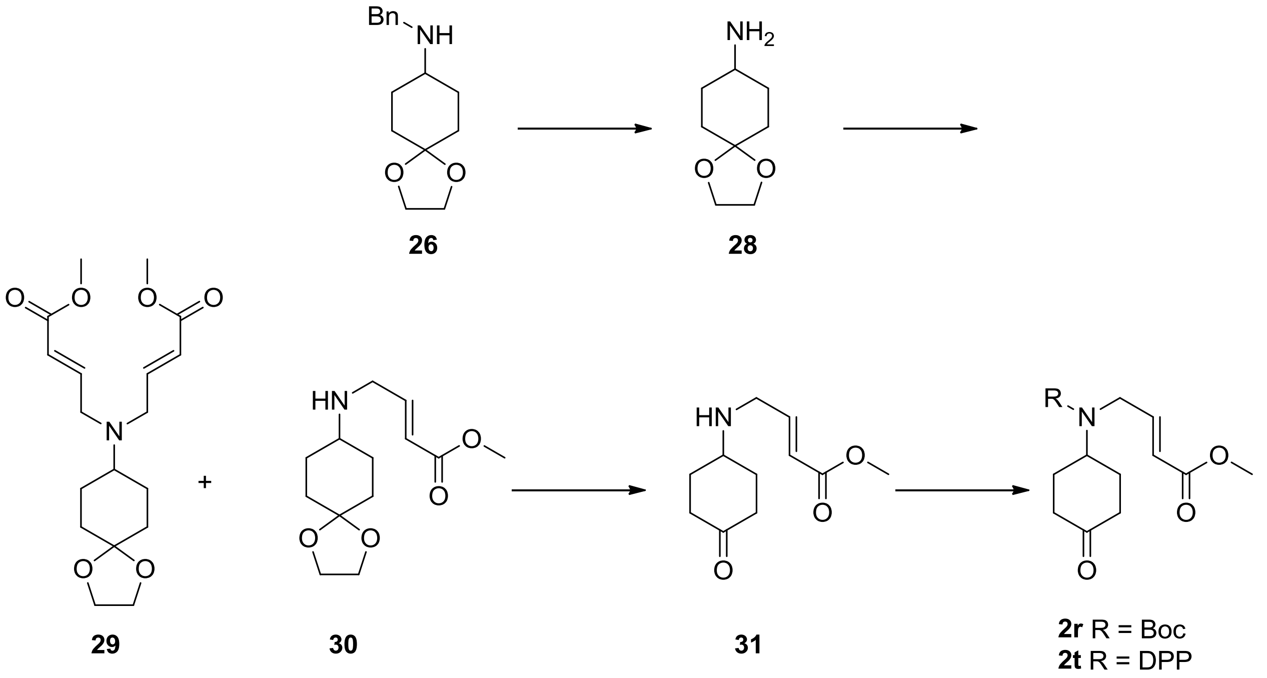

1,4-dioxaspiro[4.5]decan-8-amine (28):

To a solution of amine **26**[10] (1 eq., 20.8 mmol, 5 g) in methanol (120 ml) was added $Pd(OH)_2$ (20% wt, 50% wet; 0.025 eq., 0.50mmol, 716 mg). The resulting suspension was purged and regassed with nitrogen under vacuum (4x) and subsequently purged and regassed with hydrogen under vacuum (4x). The reaction was left under hydrogen pressure (4 balloons within each other) for 15 hours. The resulting suspension was filtered through GFA paper to remove the $Pd(OH)_2$ and concentrated in vacuo to give **28** a pure yellow oil (80%, 2.55 g)

FT-IR ν_{max}/cm^{-1} 2949, 1738; **^{1}H NMR** (400 MHz, MeOD) δ 3.94 (s, 4H), 2.99 (m, 1H), 1.97 – 1.88 (m, 2H), 1.82 – 1.75 (m, 2H), 1.66 – 1.57 (m, 4H). **^{13}C NMR** (101 MHz, MeOD) δ, 107.7, 64.43, 64.35, 49.1, 32.8 (2C), 30.0 (2C). **HRMS** (ESI+) calcd. for $C_8H_{16}NO_2$+ $[M+H]^+$ 158.1176 found 158.1181.

[10] Ahmed F. Abdel-Magid,* Kenneth G. Carson, Bruce D. Harris, Cynthia A. Maryanoff, Rekha D. Shah., J. Org. Chem., 1996, 61, 3849, and used without further purification.

(E)-methyl 4-(1,4-dioxaspiro[4.5]decan-8-ylamino)but-2-enoate (30):

To a solution of primary amine **28** (1 eq., 15.9 mmol, 2.493 g) in Acetonitrile (117 ml) was added K_2CO_3 (1.2 eq., 19.05 mmol, 1.63 g). To this resulting suspension at -10 °C was added dropwise a solution of methyl 4-bromocrotonate (0.95 eq., 15.07 mmol, 2.698 g) in acetonitrile (20 ml) *via* syringe pump over 2 hours. The solution was allowed to stir for a further 2 hours at this temperature and was subsequently warmed to room temperature for 2 hours. The reaction mixture was filtered through Celite® and concentrated *in vacuo*. The resulting oil was purified by FCC (EtOAc:MeOH:Et_3N 50:0:0 → 95:2.5:2.5) to give compound **30** (62% 2.54 g) as a yellow oil and double alkylation product **29** (15%, 840 mg) as a yellow oil.

FT-IR ν_{max}/cm^{-1} 3012, 2358, 1724; **^{1}H NMR** (400 MHz, $CDCl_3$) δ 6.95 (dtd, *J* = 15.5, 5.2, 3.1 Hz, 1H), 5.94 (d, *J* = 15.7 Hz, 1H), 3.88 (s, 4H), 3.68 (d, *J* = 2.7 Hz, 3H), 3.39 (dd, *J* = 4.7, 2.9 Hz, 2H), 2.56 – 2.46 (m, 1H), 1.86 – 1.76 (m, 2H), 1.75 – 1.68 (m, 2H), 1.49 (t, *J* = 12.6 Hz, 2H), 1.42 – 1.34 (m, 2H), 1.29 (bs, 1H). **^{13}C NMR** (101 MHz, $CDCl_3$) δ 166.8, 147.6, 120.8, 108.5, 64.22, 64.18, 54.4, 51.5, 47.6, 32.8 (2C), 30.1 (2C). **HRMS** (ESI+) calcd. for $[M+H]^+$ $C_{13}H_{22}NO_4$+ *256.1543* found *256.1550*;

(2E,2'E)-dimethyl 4,4'-(1,4-dioxaspiro[4.5]decan-8-ylazanediyl)bis(but-2-enoate) (29):

FT-IR ν_{max}/cm^{-1} 2948, 1719, 1656; **^{1}H NMR** (400 MHz, $CDCl_3$) δ 6.88 (dt, *J* = 15.6, 5.4 Hz, 2H), 5.99 (d, *J* = 15.7 Hz, 2H), 3.89 (s, 4H), 3.71 (s, 6H), 3.23 (dd, *J* = 5.4, 1.4 Hz, 4H), 2.57 – 2.47 (m, 1H), 1.74 (dd, *J* = 22.1, 12.3 Hz, 4H), 1.57 – 1.42 (m, 4H). **^{13}C NMR** (101 MHz, $CDCl_3$) δ 166.8 (2C), 147.6 (2C), 121.7 (2C), 108.3, 64.3, 64.2, 58.3, 51.5 (2C), 51.3 (2C), 33.8 (2C), 25.5 (2C). **HRMS** (ESI+) calcd. for $C_{18}H_{28}NO_6$+ $[M+H]^+$ 354.1911 found 354.1905.

(E)-methyl 4-((4-oxocyclohexyl)amino)but-2-enoate (31):

To a solution of acetal **30** (1 eq., 9.94 mmol, 2.54 g) in THF (50 ml) was added 1M HCl (50 ml) at RT and the resulting solution was stirred for 50 h at RT. The resulting acidic solution was basified with 5M KOH, saturated with NaCl(s) and extracted quickly with EtOAc. The combined organic layers were dried over Na_2SO_4 and concentrated in vacuo. The resulting yellow oil was purified by FCC (EtOAc:MeOH:Et_3N 95:2.5:2.5) to afford the title compound **31** as dark yellow viscous oil (66%, 1.38 g). **FT-IR** ν_{max}/cm^{-1} 3014, 1718, 1650; **^{1}H NMR** (400 MHz, MeOD) δ 6.99 (dt, *J* = 15.7, 5.5 Hz, 1H), 5.99 (dt, *J* = 15.7, 1.8 Hz, 1H), 3.71 (s, 3H), 3.45 (dd, *J* = 5.5, 1.8 Hz, 2H), 3.01 – 2.93 (m, 1H), 2.46 (dt, *J* = 12.0, 5.7 Hz, 2H), 2.32 – 2.22 (m, 2H), 2.05 (ddd, *J* = 13.3, 7.2, 4.2 Hz, 2H), 1.72 – 1.60 (m, 2H). **^{13}C NMR** (101 MHz, MeOD) δ 207.1, 162.8, 143.1, 117.2, 59.7, 49.0, 47.6, 43.8, 34.4 (2C), 27.9 (2C). **HRMS** (ESI+) calcd. for $[M+H]^+$ $C_{11}H_{18}NO_3$ 212.1281 found 212.1279.

(E)-methyl 4-((tert-butoxycarbonyl)(4-oxocyclohexyl)amino)but-2-enoate (2r):

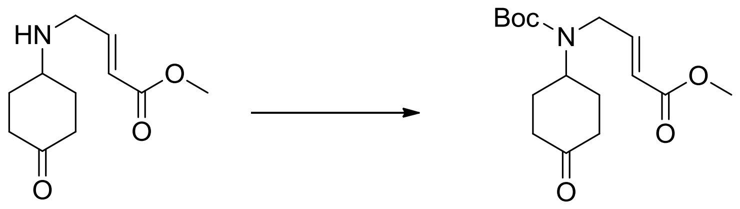

31 **2r**

To a solution of amine **31** (1 eq., 0.95 mmol, 200 mg) in MeOH (3.2 ml) was added Boc_2O (1.15 eq., 1.09 mmol, 237 mg) and stirred for 10 hours. The resulting solution was concentrated and purified by FCC (EtOAc:PE 40:60) to give compound **2r** as a white solid (83%, 245mg).

FT-IR ν_{max}/cm^{-1} 1739,1722, 1685; **^{1}H NMR** (400 MHz, MeOD) **Major rotamer:** δ 6.88 (dt, *J* = 15.7, 5.0 Hz, 1H), 5.91 (dt, *J* = 15.7, 1.7 Hz, 1H), 4.54 – 4.21 (m, 1H), 3.98 (d, *J* = 3.3 Hz, 2H), 3.72 (s, 3H), 2.56 (td, *J* = 14.2, 6.6 Hz, 2H), 2.38 – 2.29 (m, 2H), 2.06 – 1.86 (m, 4H), 1.47 (s, 9H). **^{13}C NMR** (101 MHz, MeOD) **Major rotamer:** δ 210.9, 167.2, 155.7, 146.6, 121.13, 80.8, 51.2, 44.1, 39.6 (2C), 29.7 (2C), 27.7 (3C). **HRMS** (ESI+) calcd. for $[M+Na]^+$ $C_{16}H_{25}NNaO_5$ 334.1625 found 334.1627.

(E)-methyl 4-((diphenylphosphoryl)(4-oxocyclohexyl)amino)but-2-enoate (2u):

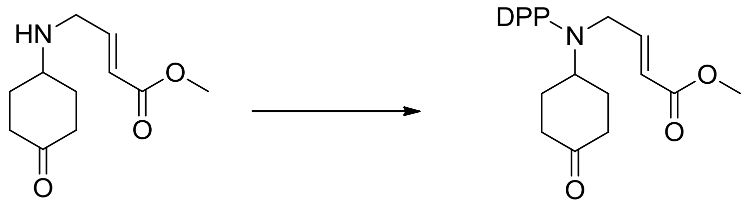

31 **2t**

To a solution of amine **31** (1 eq., 0.95 mmol, 200 mg) in DCM (3.2 ml) at 0 °C was added Et_3N (1.2 eq., 1.14 mmol,114 mg), DMAP (0.50 eq., 0.475 mmol, 58 mg) and diphenylphosphinic chloride and stirred for 24 h. The resulting solution was concentrated and purified by FCC (EtOAc:PE 50:50) to give compound **2t** as a glassy oil (70%, 268 mg).

FT-IR ν_{max}/cm^{-1} 1722, 1685, 1659; **^{1}H NMR** (400 MHz, $CDCl_3$) δ 7.90 – 7.80 (m, 4H), 7.55 – 7.40 (m, 6H), 6.73 (dt, *J* = 15.5, 5.6 Hz, 1H), 5.65 (dd, *J* = 15.7, 1.3 Hz, 1H), 3.77 – 3.68 (m, 3H), 3.68 (s, 3H), 2.36 – 2.14 (m, 6H), 2.02 – 1.88 (m, 2H). **^{13}C NMR** (101 MHz, $CDCl_3$) δ 209.1, 166.1, 147.3 (d, *J* = 4.0 Hz), 132.2 (d, *J* = 2.7 Hz, 2C), 132.2 (d, *J* = 9.4 Hz, 4C), 131.4 (d, *J* = 127.8 Hz, 2C), 128.7 (d, *J* = 12.4 Hz, 4C), 121.6, 54.6 (d, *J* = 3.4 Hz), 51.7, 43.8 (d, *J* = 4.1 Hz), 40.1 (2C), 31.2 (d, *J* = 3.5 Hz, 2C). **HRMS** (ESI+) calcd. for $[M+Na]^+$ $C_{23}H_{26}NNaO_4P$ 434.1492 found 434.1490.

Synthesis and characterization of substrate 2s

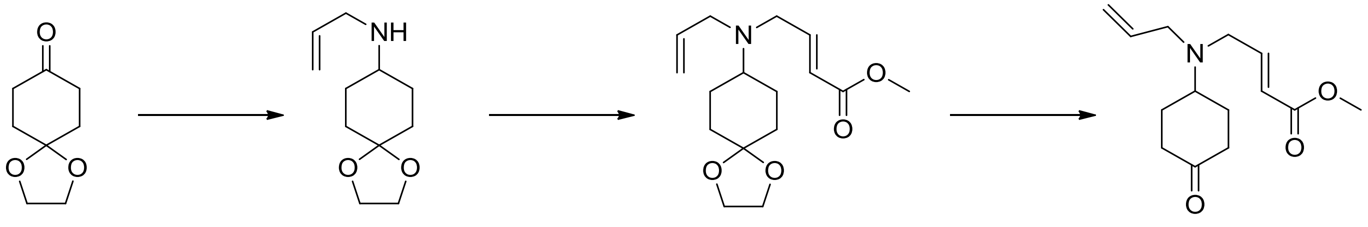

32 **33** **2s**

N-allyl-1,4-dioxaspiro[4.5]decan-8-amine (32):

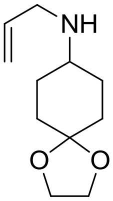

To a solution of 1,4-cyclohexanedione monoethylene acetal (1 eq., 4.49 mmol, 700 mg) and allyl amine (1.05 eq., 4.71 mmol, 269 mg) in DCE (15.7 ml) was added $NaBH(OAc)_3$ (1.5 eq., 6.73 mmol, 1.42 g) portionwise. The resulting suspension was stirred for 6 hours, basified to pH 10 with $NaOH_{aq}$ (1 M) and the organic and aquous layers seperated. The aq layer was extracted with CH_2Cl_2. The combined organic extracts were dried over Na_2SO_4 with vigorous stirring to remove residual borate salts. The organic layer was filtered and concentrated *in vacuo* to give the title compound **32** as a pale yellow oil (90%, 793 mg). The compound was sufficiently pure to use in the next step with no further purification.

FT-IR ν_{max}/cm^{-1} 2940.^{1}**H NMR** (400 MHz, $CDCl_3$) δ 5.86 – 5.73 (m, 1H), 5.06 (d, *J* = 17.2 Hz, 1H), 4.97 (d, *J* = 10.2 Hz, 1H), 3.81 (s, 4H), 3.17 (dd, *J* = 6.0, 0.9 Hz, 2H), 2.52 – 2.40 (m, 1H), 1.81 – 1.70 (m, 3H), 1.69 – 1.61 (m, 2H), 1.43 (td, *J* = 12.7, 3.4 Hz, 2H), 1.38 – 1.27 (m, 2H). ^{13}**C NMR** (101 MHz, $CDCl_3$) δ 136.85, 115.69, 108.47, 64.14, 64.10, 54.28, 49.55, 32.85, 29.95. **HRMS** (ES+) exact mass calculated for $[M+Na]^+$ ($C_{11}H_{19}NNaO_2$+) requires *m/z* 220.1308, found *m/z* 220.131.

(E)-methyl 4-(allyl(1,4-dioxaspiro[4.5]decan-8-yl)amino)but-2-enoate (33):

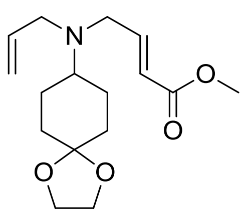

To a solution of amine **32** (1 eq., 4.025 mmol, 793 mg) in MeCN (13.4 ml) and K_2CO_3 (1.2 eq., 4.83 mmol, 666 mg) at 0 °C was added methyl 4-bromocrotonate (85%; 1.1 eq., 4.43 mmol, 932 mg). The resulting suspension was warmed to RT and stirred for 12 h and subsequently filtered through Celite® and concentrated *in vacuo* to give title compound **33** as viscous yellow oil (81%, 969 mg). The resulting yellow oil was purified by FCC (50:50 EtOAc:PE).

FT-IR ν_{max}/cm^{-1} 1720, 1659; **^{1}H NMR** (400 MHz, $CDCl_3$) δ 6.92 (dtd, *J* = 15.6, 5.4, 0.5 Hz, 1H), 6.00 (ddd, *J* = 15.6, 1.8, 1.2 Hz, 1H), 5.82 – 5.71 (m, 1H), 5.14 (dd, *J* = 17.2, 1.3 Hz, 1H), 5.05 (dd, *J* = 10.1, 1.2 Hz, 1H), 3.90 (d, *J* = 0.7 Hz, 4H), 3.71 (d, *J* = 0.7 Hz, 3H), 3.23 (dd, *J* = 5.5, 1.6 Hz, 2H), 3.09 (d, *J* = 6.1 Hz, 2H), 2.56 (ddd, *J* = 10.8, 7.4, 3.7 Hz, 1H), 1.78 – 1.67 (m, 4H), 1.57 – 1.44 (m, 4H). **^{13}C NMR** (101 MHz, $CDCl_3$) δ 166.99, 148.52, 136.90, 121.31, 116.61, 108.43, 64.26, 64.18, 58.07, 53.65, 51.41, 50.74, 33.89, 25.54. **HRMS** (ES+) exact mass calculated for $[M+H]^+$ ($C_{16}H_{26}NO_4$) requires *m/z* 296.1856, found *m/z* 296.1857.

(E)-methyl 4-(allyl(4-oxocyclohexyl)amino)but-2-enoate (2s):

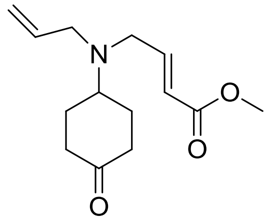

According to **general procedure E** using acetal **33** (1 eq., 2.89 mmol, 851 mg) as starting material. The reslting crude yellow oil was purified by FCC (PE/EtOAc) = 1:1) to afford the title compound **2s** as a pale yellow viscous oil (94%, 680 mg).

FT-IR ν_{max}/cm^{-1} 1722, 1656; **^{1}H NMR** (400 MHz, $CDCl_3$) δ 6.92 (dtd, *J* = 15.6, 5.5, 1.4 Hz, 1H), 6.00 (dd, *J* = 15.7, 1.5 Hz, 1H), 5.78 (qdd, *J* = 12.0, 6.1, 1.2 Hz, 1H), 5.16 (d, *J* = 17.2 Hz, 1H), 5.09 (d, *J* = 10.1 Hz, 1H), 3.70 (s, 3H), 3.26 (d, *J* = 5.6 Hz, 2H), 3.17 – 3.10 (m, 2H), 3.02 – 2.94 (m, 1H), 2.39 (d, *J* = 14.1 Hz, 2H), 2.34 – 2.22 (m, 2H), 2.05 – 1.96 (m, 2H), 1.70 (qd, *J* = 12.2, 4.6 Hz, 2H). **^{13}C NMR** (101 MHz, $CDCl_3$) δ 210.76, 166.77, 147.58, 136.17, 121.77, 117.21, 56.85, 53.52, 51.49, 50.74, 39.59, 27.94. **HRMS** (ES+) exact mass calculated for $[M+H]^+$ ($C_{14}H_{22}NO_3$) requires *m/z* 252.1593, found *m/z* 252.1587.

Synthesis and characterization of substrate 2u

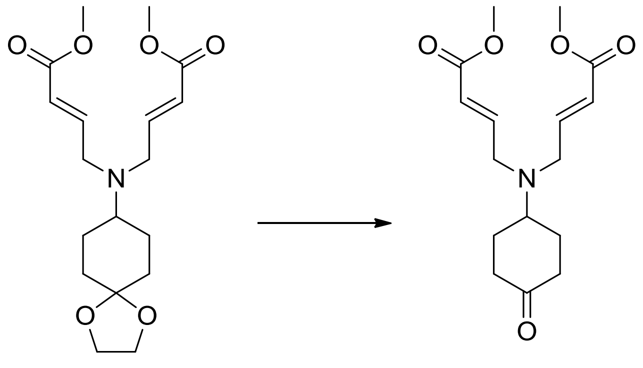

29 2u

(2E,2'E)-dimethyl 4,4'-((4-oxocyclohexyl)azanediyl)bis(but-2-enoate) (2u):

According to general procedure E using **29** (1 eq., 2.27 mmol, 800mg) as starting material. The resulting yellow oil was purified by FCC (EtOAc:PE 30:70) to afford the title compound **2u** as a yellow viscous oil (86%, 601 mg).

FT-IR ν_{max}/cm^{-1} 1710, 1653; **^{1}H NMR** (400 MHz, $CDCl_3$) δ 6.90 (dt, *J* = 15.7, 5.6 Hz, 2H), 6.01 (dt, *J* = 15.7, 1.5 Hz, 2H), 3.71 (s, 6H), 3.28 (dd, *J* = 5.6, 1.6 Hz, 4H), 2.97 (tt, *J* = 10.8, 3.3 Hz, 1H), 2.46 – 2.23 (m, 4H), 2.08 – 1.97 (m, 2H), 1.70 (ddd, *J* = 24.3, 12.4, 5.0 Hz, 2H). **^{13}C NMR** (101 MHz, $CDCl_3$) δ 210.22, 166.57, 146.58, 122.27, 57.27, 51.59, 51.28, 39.53, 27.97. **HRMS** (ESI+) calcd. for $[M+H]^+$ $C_{16}H_{24}NO_5$ 310.1653 found 310.1657.

Synthesis and characterization of substrate 2v

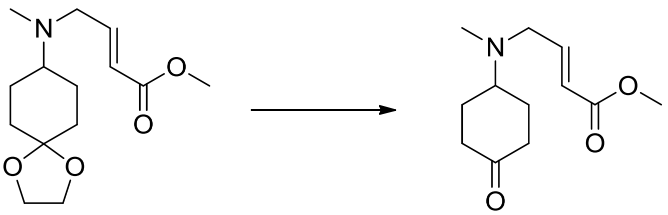

34 2v

According to **general procedure E** using acetal **34** (500 mg, 1.85 mmol) as starting material. The reslting yellow oil was purified by FCC (PE:Acetone 20:80) to afford the title compound **2v** as a pale yellow viscous oil (84%, 351 mg)

FT-IR ν_{max}/cm^{-1} 2950, 1713, 1574; **^{1}H NMR** (400 MHz, $CDCl_3$) δ 6.98 (dt, *J* = 15.7, 6.0 Hz, 1H), 6.03 (dt, *J* = 15.7, 1.7 Hz, 1H), 3.75 (s, 3H), 3.29 (dd, *J* = 6.0, 1.6 Hz, 2H), 2.85 (tt, *J* = 9.7, 3.3 Hz, 1H), 2.51 – 2.44 (m, 2H), 2.37 – 2.30 (m, 2H), 2.29 (s, 3H), 2.07 – 1.99 (m, 2H), 1.88 – 1.77 (m, 2H). **^{13}C NMR** (101 MHz, $CDCl_3$) δ 210.8, 166.7, 146.4, 122.4, 59.3, 55.0, 51.6, 39.1, 38.3, 27.9. Chemical **HRMS** (ESI+) calcd. for $[M+H]^+$: $C_{12}H_{20}NO_3$+ 226.1438 found 226.1432.

Catalyst screen and optimization

Reaction conditions for catalyst screen and optimization:

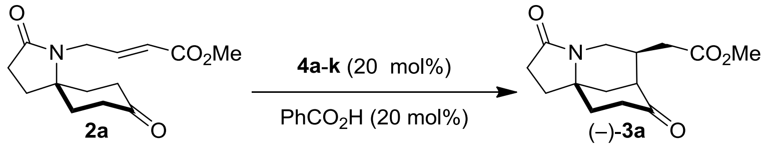

To a solution of prochiral substrate **2a** (1 eq., 40mg, 0.151 mmol) in solvents CH_2Cl_2 or $CHCl_3$ (0.2 M; see table) was added catalyst **4a-k** (20 mol% - 5mol%, see table stock solution was used) and benzoic acid (20 mol% - 1.25 mol%, see table, stock solution was used) and sealed. The reaction mixture was stirred at RT-45 °C (see table) until completion (monitored by TLC, for time see table). After completion the solvent was removed and the crude product was purified by flash column chromatography.

Table 1. Reaction development and optimization[a]

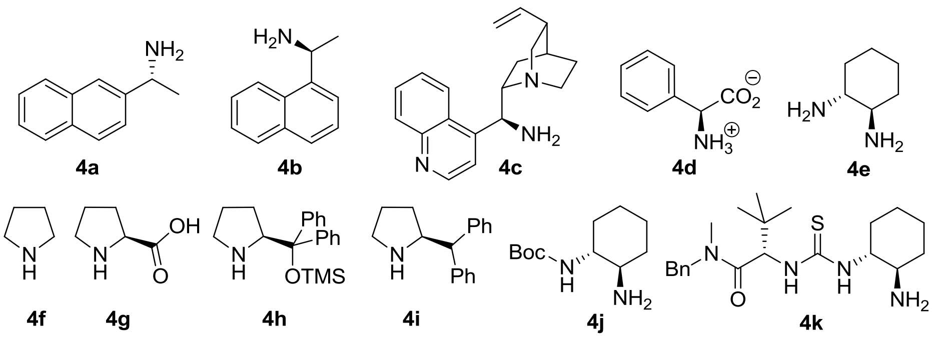

	4	Catalyst loading mol%	Benzoic acid mol%	Solvent	time	Yield (%)	dr[c]	ee[d] (%)
1	**a**	20	20	CH_2Cl_2	5 days	72	>98:2	63
2	**b**	20	20	CH_2Cl_2	8 days	74	>98:2	69[e]
3	**c**	20	20	CH_2Cl_2	7 days	69	>98:2	31
4	**d**	20	20	CH_2Cl_2	NR	-	-	-
5	**e**	20	20	CH_2Cl_2	24 h	82	>98:2	63
6	**f/g/h/i**	20	20	CH_2Cl_2	NR	-	-	-
7	**j**	20	20	CH_2Cl_2	22h	87	>98:2	64
8	**k**	20	20	CH_2Cl_2	26 h	86	>98:2	90
9	**k**	20	20[f]	CH_2Cl_2	72 h	73	>98:2	92
10	**k**	20	20	$CHCl_3$	25 h	78	>98:2	90
11	**k**	5	1.25	CH_2Cl_2	96h	80	>98:2	92
12	**k**[b]	5	1.25	CH_2Cl_2	48 h	88	>98:2	93

[a]Reaction conditions: 20 mol% catalyst, 20 mol% $PhCO_2H$, 0.2 M CH_2Cl_2, RT. [b]5 mol% 4k, 1.25 mol% benzoic acid, **45 °C**. [c]Diastereomeric ratio (dr) was determined by ^1H NMR, [d]Enantiomeric excess (ee) was determined by HPLC analysis using a chiral stationary phase. [e](+)-**3a** was observed. [f]*p*-Nitrophenol was used as acid cocatalyst.

Naphthyl methyl amines **4a** and **4b** (entrees 1&2) were first investigated; both imparted the same high diastereoselectivity as propylamine and, pleasingly, gave moderate enantiocontrol (63% ee and 69% ee respectively). **4b** gave the other enantiomer to **4a** unsurprisingly as it has the opposite configuration about the primary amine.

Although reaction times proved long, these data provided an encouraging starting point in the search for catalysts with improved stereocontrol.

9-Amino(9-deoxy)-*epi*-cinchonidine catalyst **4c** (entry 3) imparted high diasteroselectivity but with poor enantioselectivity (31% ee) and a long reaction time (7 days).

Amino acid **4d** (entry 4) was an ineffective catalyst however this was possibly due in part to its poor solubility in CH_2Cl_2.

(1*R*,2*R*)-(–)-1,2-Diaminocyclohexane **4e** (entry 5) afforded similar enantioselectivity as naphthyl amines **4a** and **4b**, but with an improved reaction time of 24 h.

Presuming that reactivity was originating from enamine intermediates, we next investigated the performance of a range of commonly used cyclic secondary amine enamine organocatalysts. Interestingly however, pyrrolidine **4f**, proline **4g**, and diphenylprolinol derivatives **4h** and **4i** (entry 6) and all failed to catalyze this transformation.

In terms of reactivity and enantioselectivity, catalyst **4e** represented the most promising lead and accordingly derivatives were sought with the aim to boost enantioselectivity. Initially commercial (1*R*,2*R*)-trans-*N*-Boc-1,2-cyclohexanediamine **4j** (entry 7) was assessed; it imparted moderate enantioselectivity (64% ee), high diastereoselectivity but most importantly the reaction time was short (22h) and gave a good yield of **3**.

We postulated that increasing the H-bond-donor capability of the cyclohexanediamine by employing a thiourea derivative could boost the enantioselectivity whilst maintaining high reactivity. Commercially available Jacobsen's thiourea catalyst **4k** (entry 8) resulted in a significant increase in enantioselectivity whilst maintaining a short reaction time and high diastereoselectivity; the major diastereomeric product **3a** was obtained in 90% ee and >98:2 dr.

A brief acid co-catalyst screen, maintaining **4k** as the primary amine catalyst, revealed that benzoic acid and *p*-nitrophenol were both superior to other common acid additives in terms of selectivity (entry 9). However, the reaction with the former was significantly quicker (26 h vs. 72 h) and consequently selected for further optimization.

$CHCl_3$ was screened as the solvent but yields were lower and the reaction was not as clean (entry 10).

Primary amine catalyst loading could be reduced to 5 mol% with no change in enantioselectivity albeit with a longer reaction time (entry 11).

Pleasingly, this could be overcome by elevating the reaction temperature to 45 °C with no loss of stereoselectivity (entry 12).

Synthesis and characterization of chiral compounds 3a-k, m-n

General procedure for the cyclisation of substrates

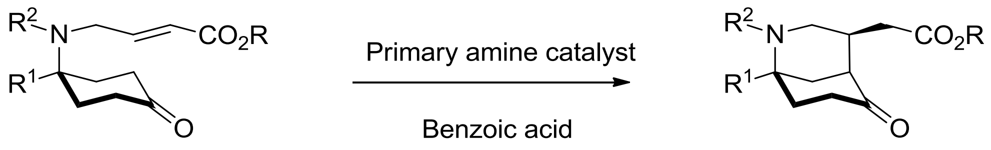

General Procedure F- for the synthesis of chiral products (3a-d):

To a solution of prochiral substrate (**2a-2d**) in CH_2Cl_2 (0.2 M) was added Jacobsen catalyst (**4k**) (5 mol%, stock solution was used) and benzoic acid (1.25 mol%, stock solution was used) and sealed. The reaction mixture was stirred at 45 ^oC until completion (monitored by TLC). After completion the solvent was removed and the crude product was purified by flash column chromatography.

General Procedure G - for the synthesis of chiral products (3e-3v):

To a solution of prochiral substrate (**2e-2v**) in CH_2Cl_2 (0.2 M) was added Jacobsen catalyst (**4k**) (5 mol%, stock solution was used) and benzoic acid (2.5 mol%, stock solution was used) and sealed. The reaction mixture was stirred at 50 ^oC until completion (monitored by TLC). After completion the solvent was removed and the crude product was purified by flash column chromatography.

General procedure H - for the synthesis of racemic products (3a-3v):

To a solution of substrates **2a-v** (30 mg) in CH_2Cl_2 (0.2 M) was added propylamine (20 mol%, stock solution was used) and benzoic acid (20 mol%, stock solution was used) and sealed. The reaction mixture was stirred at room temperature until completion 12-24h (monitored by TLC). After completion the solvent was removed and the crude product was purified by flash column chromatography yielding (±)-**3a-v** in 75-91% yield.

Synthesis and characterization of (-)-3a:

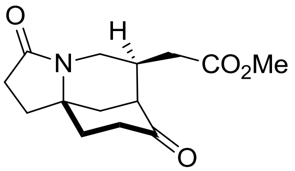

(-) 3a was obtained according to the **general procedure F** using **2a** (50 mg, 0.188 mmol), Jacobsen catalyst **4k** (3.5 mg, 0.0094 mmol) and benzoic acid (0.28 mg, 0.0023 mmol) for 40 hr. The crude reaction mixture was purified by flash column chromatography (Et_2O/MeOH = 97:3) to afford 43.8 mg (88%) of product as a solid**.**

mp 86-88 °C; **[*α*]$_D$^{20} =** -60.5 (C 0.76, $CHCl_3$); **FT-IR** ν_{max}/cm^{-1} 2948, 1732, 1675; **^{1}H NMR** (400 MHz, $CDCl_3$) δ 4.21 (dd, *J* = 14.1, 6.2 Hz, 1H), 3.64 (s, 3H), 2.83 – 2.69 (m, 2H), 2.65 – 2.55 (m, 1H), 2.54 – 2.23 (m, 6H), 2.22 – 2.04 (m, 2H), 1.96 (dd, *J* = 8.3, 5.6 Hz, 1H), 1.94 – 1.87 (m, 2H), 1.87 – 1.79 (m, 1H). **^{13}C NMR** (100 MHz, $CDCl_3$) δ 210.3, 173.4, 171.4, 57.3, 51.9, 48.8, 42.3, 39.5, 39.2, 35.9, 35.5, 34.1, 32.2, 29.6. **HRMS** (ESI+) calcd. for $C_{14}H_{18}NO_4$ [M-H]$^-$ 264.1241 found 264.1238. **HPLC:** ee = 93% (Chiralcel AD-H, 80:20 hexane/isopropanol, flow rate 1.0 ml/min, 210 nm, t_R (major) = 15.9 min, t_R (minor) = 13.9 min.

Synthesis and characterization of (-)-3b

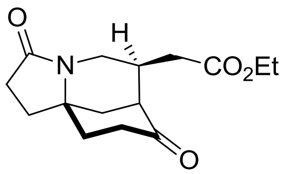

(-)-3b was obtained according to the **general procedure F** using **2b** (50 mg, 0.179 mmol), Jacobsen catalyst **4k** (3.3 mg, 0.0089 mmol) and benzoic acid (0.27 mg, 0.0022 mmol) after 40 hr. The crude reaction mixture was purified by flash column chromatography (Et_2O/MeOH = 97:3) to afford 42.2 mg (89%) of product as a solid.

mp 106-108 °C; **[*α*]$_D$^{20}** = -58.8 (C 3.4, $CHCl_3$); **FT-IR** ν_{max}/cm^{-1} 2934, 1728, 1676; **^{1}H NMR** (500 MHz, $CDCl_3$) δ 4.26 (dd, *J* = 14.1, 6.4 Hz, 1H), 4.14 (q, *J* = 7.1 Hz, 2H), 2.87 – 2.74 (m, 2H), 2.63 (dd, *J* = 18.5, 8.4 Hz, 1H), 2.58 – 2.48 (m, 1H), 2.47 – 2.26 (m, 4H), 2.25 – 2.15 (m, 2H), 2.11 (dd, *J* = 16.4, 7.1 Hz, 1H), 2.02 – 1.90 (m, 3H), 1.90 – 1.83 (m, 1H), 1.25 (t, *J* = 7.1 Hz, 3H); **^{13}C NMR** (126 MHz, $CDCl_3$) δ 210.4, 173.5, 171.1, 60.9, 57.4, 49.0, 42.4, 39.7, 39.3, 36.0, 35.9, 34.1, 32.3, 29.7, 14.2; **HRMS** (ESI+) calcd. for $C_{15}H_{20}NO_4$ [M-H]$^-$ 278.1398 found 278.1407. **HPLC:** ee = 90% (Chiralcel AD-H, 80:20 hexane/isopropanol, flow rate 1.0 ml/min, 210 nm, t_R (major) = 15.7 min, t_R (minor) = 12.6 min.

Synthesis and characterization of (-)-3c:

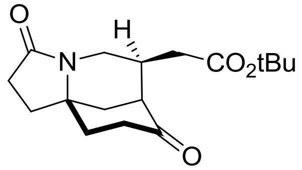

(-)-3c was obtained according to the **general procedure F** using **2c** (50 mg, 0.163 mmol), Jacobsen catalyst **4k** (3.0 mg, 0.0081 mmol) and benzoic acid (0.27 mg, 0.002 mmol) after 45 hr. The crude reaction mixture was purified by flash column chromatography (Et_2O/MeOH = 97:3) to afford 43.5 mg (87%) of product as viscous oil;

[α]$_D$ ^{20} = -47.9 (C 0.97, $CHCl_3$); **FT-IR** ν_{max}/cm^{-1} 2959, 1728, 1679; **^{1}H NMR** (400 MHz, $CDCl_3$) δ 4.26 (dd, *J* = 14.1, 6.5 Hz, 1H), 2.87 – 2.71 (m, 2H), 2.62 (dd, *J* = 18.2, 8.2 Hz, 1H), 2.56 – 2.28 (m, 4H), 2.28 – 2.12 (m, 3H), 2.09 – 1.80 (m, 5H), 1.44 (s, 9H). **^{13}C NMR** (100 MHz, $CDCl_3$) δ 210.2, 173.3, 170.2, 81.1, 57.2, 48.8, 42.3, 39.6, 39.2, 36.9, 35.9, 34.0, 32.1, 29.5, 28.0 (3C); **HRMS** (ESI+) calcd. for $C_{17}H_{24}NO_4$ $[M-H]^-$ 306.1711 found 306.1707. **HPLC:** ee = 92% (Chiralcel AD-H, 80:20 hexane/isopropanol, flow rate 0.8 ml/min, 210 nm, t_R (major) = 13.4 min, t_R (minor) = 10.1 min.

Synthesis and characterization of (-)-3d:

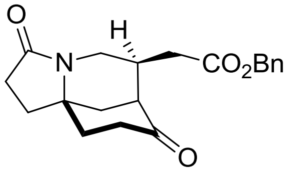

(-) 3d was obtained according to the **general procedure F** using **2d** (50 mg, 0.146 mmol), Jacobsen catalyst **4k** (2.7 mg, 0.0073 mmol) and benzoic acid (0.22 mg, 0.002 mmol) after 40 hr. The crude reaction mixture was purified by flash column chromatography (Et_2O/MeOH = 97:3) to afford 40.3 mg (81%) of product as solid.

mp 116-118 °C, **[α]$_D$ ^{20}** = -41.6 (C 0.54, $CHCl_3$); **FT-IR** ν_{max}/cm^{-1} 2979, 1732, 1682; **^{1}H NMR** (500 MHz, $CDCl_3$) δ 7.41 – 7.27 (m, 5H), 5.13 (s, 2H), 4.27 (dd, *J* = 14.0, 6.2 Hz, 1H), 2.87 – 2.74 (m, 2H), 2.64 (dd, *J* = 18.5, 8.4 Hz, 1H), 2.58 – 2.49 (m, 1H), 2.48 – 2.31 (m, 4H), 2.25 – 2.14 (m, 3H), 2.02 – 1.91 (m, 3H), 1.90 – 1.83 (m, 1H). **^{13}C NMR** (125 MHz, $CDCl_3$) δ 210.2, 173.4, 170.8, 135.5, 128.5 (2xC), 128.3, 128.2 (2xC), 66.6, 57.2, 48.7, 42.3, 39.5, 39.1, 35.8, 35.7, 34.0, 32.1, 29.5; **HRMS** (ESI+) calcd. for $C_{20}H_{23}NNaO_4$ $[M+Na]^+$ 364.1519 found 364.1508. **HPLC:** ee = 92% (Chiralcel AD-H, 80:20 hexane/isopropanol, flow rate 1 ml/min, 210 nm, t_R (major) = 30.2 min, t_R (minor) = 23.6 min.

Synthesis and characterization of Methyl [(6S,7S,10aR)-8-oxooctahydro-1H-7,10a-methanopyrrolo[1,2-a]azocin-6-yl]acetate (-)-3e.

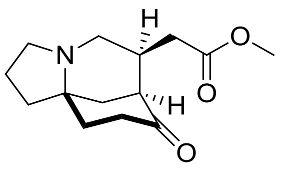

(-) 3e was obtained according to the **general procedure G** using **2e** (40 mg, 0.159 mmol), Jacobsen's catalyst **4k** (5 mol%) and benzoic acid (2.5 mol%) for 36 h. The crude reaction mixture was purified by flash column chromatography (EtOAc) to afford 33 mg (83%) of product as a pale yellow oil.

[α]$_D^{25}$ = -47.2 (C 1.1, $CHCl_3$); **FT-IR** ν_{max}/cm^{-1} 1734.30 (C=O), 1702.57 (C=O);; **^{1}H NMR** (500 MHz, $CDCl_3$) δ 3.67 (s, 3H), 3.11 (td, *J* = 9.2, 5.3, 1H), 3.05 (dd, *J* = 11.7, 3.9, 1H), 2.87 – 2.75 (m, 1H), 2.66 – 2.48 (m, 4H), 2.38 – 2.27 (m, 2H), 2.18 – 2.08 (m, 1H), 2.06 – 1.99 (m, 2H) 1.93 – 1.78 (m, 3H), 1.75 – 1.61 (m, 3H,). **^{13}C NMR** (126 MHz, $CDCl_3$) δ 213.6, 172.8, 57.4, 52.2, 50.8, 50.6, 49.7, 39.0, 38.8, 36.6, 36.3, 33.5, 29.8, 20.8. **HRMS** (ES+) exact mass calculated for $[M+H]^+$ ($C_{14}H_{22}NO_3$) requires *m/z* 252.1594, found *m/z* 253.1596; **HPLC:** ee = 94% (Chiralpak AD, hexane/isopropanol 90:10, 210 nm, 1.0 mL/min) t_R (major) = 13.8, T_R minor = 12.0);

Synthesis and characterization of Ethyl [(6S,7S,10aR)-8-oxooctahydro-1H-7,10a-methanopyrrolo[1,2-a]azocin-6-yl]acetate (-)-3f

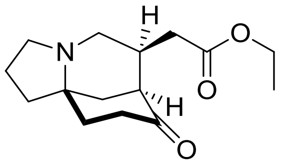

(-) 3f was obtained according to the **general procedure G** using **2f** (40 mg, 0.151 mmol), Jacobsen's catalyst **4k** (5 mol%) and benzoic acid (2.5 mol%) for 36 h. The crude reaction mixture was purified by flash column chromatography (EtOAc) to afford 35 mg (89%) of product as a pale yellow oil.

[α]$_D^{25}$ = -56.2 (C 2.1, $CHCl_3$); **FT-IR** ν_{max}/cm^{-1} 1730.73 (C=O), 1702.56 (C=O); **^{1}H NMR** (500 MHz, $CDCl_3$) δ 4.11 (q, *J* = 7.1, 2H), 3.13 – 3.01 (m, 2H), 2.79 (dd, *J* = 15.0, 9.1, 1H), 2.65 – 2.48 (m, 4H), 2.37 – 2.27 (m, 2H), 2.15 – 2.07 (m, 1H), 2.04 – 1.95 (m, 2H), 1.92 – 175 (m, 3H), 1.72 – 1.66 (m, 1H), 1.63 (t, *J* = 7.9, 2H), 1.23 (t, *J* = 7.1, 3H); **^{13}C NMR** (126 MHz, $CDCl_3$) δ 213.6, 172.3, 61.0, 57.3, 50.9, 50.6, 49.8, 40.0, 39.8, 36.6, 36.3, 33.5, 29.6, 20.8, 14.6; **HRMS** (ES+) exact mass calculated for $[M+H]^+$ ($C_{15}H_{24}NO_3$) requires *m/z* 266.1751, found *m/z* 266.1749. **HPLC**: ee=94% (Chiralpak AD, hexane/isopropanol 90:10, 210 nm, 1.0 mL/min) t_R (major) = 12.5, t_R minor = 10.6).

Synthesis and characterization of Benzyl [(6S,7S,10aR)-8-oxooctahydro-1H-7,10a-methanopyrrolo[1,2-a]azocin-6-yl]acetate (-)-3g

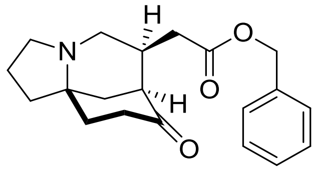

(-) 3g was obtained according to the **general procedure G** using **2g** (40 mg, 0.122 mmol), Jacobsen's catalyst **4k** (5 mol%) and benzoic acid (2.5 mol%) for 36 h. The crude reaction mixture was purified by flash column chromatography (EtOAc) to afford 34 mg (85%) of product as a pale yellow oil.

[α]$_D^{25}$ = -32.2 (C 0.8, $CHCl_3$); **FT-IR** ν_{max}/cm^{-1} 1731.96 (C=O), 1702.19 (C=O); ^{1}**H NMR** (500 MHz, $CDCl_3$) δ 7.39 – 7.30 (m, 4H), 5.13 (s, 2H), 3.17 – 3.04 (m, 2H), 2.89 (s, 1H), 2.74 – 2.51 (m, 4H), 2.45 – 2.31 (m, 3H), 2.25 – 2.16 (m, 1H), 2.09 (dd, *J* = 16.4, 7.8, 1H), 2.03 (dd, *J* = 13.3, 3.4, 1H), 1.96 – 1.85 (m, 3H), 1.80 – 1.64 (m, 3H); ^{13}**C NMR** (126 MHz, CDCl3) δ 212.7, 171.6, 135.7, 128.6 (2C), 128.3, 128.2 (2C), 66.4, 57.4, 50.2, 50.0, 48.9, 39.5, 39.2, 38.1, 35.9, 32.6, 29.2, 20.2. **HRMS** (ES+) exact mass calculated for $[M+H]^+$ ($C_{20}H_{26}NO_3$) requires *m/z* 328.1907, found *m/z* 328.1902. **HPLC: ee = 95%** (Chiralpak AD, hexane/isopropanol 95:5, 210 nm, 1.0 mL/min) t_R (major) = 21.4, t_R minor = 19.0);

Synthesis and characterization of Phenyl [(6S,7S,10aR)-8-oxooctahydro-1H-7,10a-methanopyrrolo[1,2-a]azocin-6-yl]acetate (-)-3h

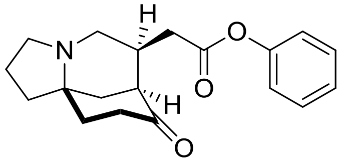

(-) 3h was obtained according to the **general procedure G** using **2h** (40 mg, 0.128 mmol), Jacobsen's catalyst **4k** (5 mol%) and benzoic acid (2.5 mol%) for 36 h. The crude reaction mixture was purified by flash column chromatography (EtOAc) to afford 33 mg (83%) of product as a pale yellow oil.

[α]$_D^{25}$ = -32.8 (C 0.75, $CHCl_3$); **FT-IR** ν_{max}/cm^{-1} 1753.56 (C=O), 1701.32 (C=O), ^{1}**H NMR** (500 MHz, $CDCl_3$) δ 7.36 (t, *J* = 7.9, 2H), 7.21 (t, *J* = 7.4, 1H), 7.08 (d, *J* = 7.6, 2H), 3.18 – 3.08 (m, 2H), 2.80 (td, *J* = 9.3, 6.0, 1H), 2.70 (dd, *J* = 12.9, 6.7, 3H), 2.62 – 2.52 (m, 2H), 2.37 (dt, *J* = 18.5, 9.2, 1H), 2.32 – 2.25 (m, 1H), 2.16 – 2.08 (m, 1H), 2.06 (dd, *J* = 13.2, 3.3, 1H), 1.94 – 1.78 (m, 3H), 1.72 (dt, *J* = 14.5, 9.3, 1H), 1.65 (t, *J* = 7.9, 2H). ^{13}**C NMR** (126 MHz, $CDCl_3$) δ 213.7, 170.9, 151.0, 129.8 (2×C), 126.3, 122.0 (2×C), 57.2, 50.8, 50.6, 49.6, 39.9, 38.9, 36.6, 36.1, 33.6, 29.5, 20.8; **HRMS** (ES+) exact mass calculated for $[M+H]^+$ ($C_{19}H_{24}NO_3$) requires *m/z* 314.1751, found *m/z* 314.1754. **HPLC:** ee = 94% (Chiralpak OD, hexane/isopropanol 95:5, 210 nm, 1.0 mL/min) t_R (major) = 18.2, t_R minor = 15.7).

Synthesis and characterization of Ethyl [(1*R*,4*S*,5*S*)-2-ethyl-1-methyl-6-oxo-2-azabicyclo[3.3.1]non-4-yl]acetate (-)-3i

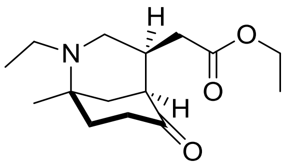

(-) 3i was obtained according to the **general procedure G** using **2i** (40 mg, 0.150 mmol), Jacobsen's catalyst **4k** (5 mol%) and benzoic acid (2.5 mol%) for 96 h. The crude reaction mixture was purified by flash column chromatography (EtOAc) to afford 33 mg (83%) of product as a pale yellow oil.

FT-IR ν_{max}/cm^{-1} 1732.06 (C=O), 1703.37 (C=O); $[\alpha]_D^{25}$ = -38.8 (C 0.9, $CHCl_3$) **^{1}H NMR** (500 MHz, $CDCl_3$) δ 4.13 (q, *J* = 7.1 , 2H), 3.23 – 3.11 (m, 1H), 3.03 (dd, , 1H), 2.63 – 2.45 (m, 3H), 2.34 (dd, *J* = 16.1, 6.2 , 1H), 2.31 – 2.17 (m, 4H), 2.04 (dd, *J* = 16.1, 7.6 , 2H), 1.95 (dd, *J* = 13.4, 3.3 , 1H), 1.65 – 1.54 (m, 1H), 1.25 (t, *J* = 7.1 , 3H), 1.23 (s, 3H), 1.13 (t, *J* = 6.9 , 3H); **^{13}C NMR** (126 MHz, CDCl3) δ 213.2, 171.7, 60.5, 51.9, 48.2, 44.0, 41.1, 39.7, 36.6, 35.2, 29.7, 29.3, 28.9, 14.2, 13.8; **HRMS** (ES+) exact mass calculated for $[M+Na]^+$ ($C_{15}H_{25}NNaO_3$) requires *m/z* 290.1727, found *m/z* 290.1732; **HPLC:** ee = 96% (Chiralpak AD, hexane/isopropanol 97:3, 210 nm, 1.0 mL/min) t_R (major) = 9.2, t_R minor = 8.3).

methyl 2-((1S,4S,5S)-6-oxo-1-phenethyl-2-azabicyclo[3.3.1]nonan-4-yl)acetate (-)-3j

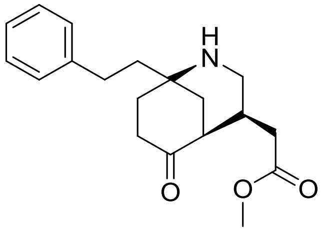

(-) 3j was obtained according to the **general procedure G** using **2j** (50 mg, 0.159 mmol), Jacobsen's catalyst **4k** (5 mol%) and benzoic acid (2.5 mol%) for 96 h. The crude reaction mixture was purified by flash column chromatography (EtOAc) to afford 46 mg (92%) of product as a pale yellow oil.

$[\alpha]_D^{20}$ = -22.3 (C 1.5, $CHCl_3$); **FT-IR** ν_{max}/cm^{-1} 2953, 1733, 1704; **^{1}H NMR** (400 MHz, $CDCl_3$) δ 7.32 – 7.26 (m, 2H), 7.20 (m, 3H), 3.68 (s, 3H), 3.09 (dd, *J* = 13.5, 4.8 Hz, 1H), 2.83 – 2.65 (m, 4H), 2.61 – 2.29 (m, 4H), 2.11 – 1.98 (m, 4H), 1.82 (d, *J* = 13.2 Hz, 1H), 1.73 (t, *J* = 8.7 Hz, 2H), 1.62 (bs, 1H). **^{13}C NMR** (101 MHz, $CDCl_3$) δ 213.9, 172.5, 142.1, 128.5 (2C), 128.3 (2C), 125.9, 51.7, 49.8, 48.2, 46.4, 46.3, 39.3, 38.1, 36.7, 35.9, 33.5, 29.7. **HRMS** (ES+) exact mass calculated for $[M+Na]^+$ (*$C_{19}H_{25}NNaO_3$+*) requires *m/z 338.1727*, found *m/z 338.1721*. **HPLC:** ee = 84% (Chiralpak AD-H, hexane/isopropanol 90:10, 210 nm, 1.0 mL/min) t_R (major) = 31.2, t_R minor = 25.4).

methyl 2-((1R,4S,5S)-1-hexadecyl-6-oxo-2-azabicyclo[3.3.1]nonan-4-yl)acetate (-)-3k

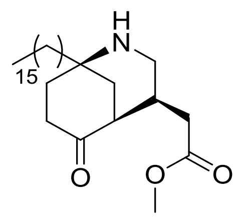

(-) 3k was obtained according to the **general procedure G** using **2k** (43 mg, 0.099 mmol), Jacobsen's catalyst **4k** (5 mol%) and benzoic acid (2.5 mol%) for 96 h. The crude reaction mixture was purified by flash column chromatography (EtOAc) to afford 46 mg (86%) of product as a pale yellow oil.

$[\alpha]_D^{20}$ = -11.6 (C 1.5, $CHCl_3$); **FT-IR** ν_{max}/cm^{-1} 2957, 1732, 1704; **^{1}H NMR** (400 MHz, $CDCl_3$) δ 3.67 (s, 3H), 3.05 (dd, *J* = 13.6, 4.8 Hz, 1H), 2.80 – 2.67 (m, 1H), 2.63 (d, *J* = 2.9 Hz, 1H), 2.56 – 2.25 (m, 4H), 2.05 (dd, *J* = 15.8, 7.3 Hz, 1H), 2.00 – 1.89 (m, 3H), 1.73 (d, *J* = 13.2 Hz, 1H), 1.43 – 1.18 (m, 31H), 0.88 (t, *J* = 6.8 Hz, 3H). **^{13}C NMR** (101 MHz, $CDCl_3$) δ 214.1, 172.5, 51.7, 49.7, 48.2, 46.3, 44.2, 39.4, 38.0, 36.7, 35.9, 33.3, 31.9, 30.2, 29.75 – 29.53 (9C), 29.4, 23.1, 22.7, 14.1. **HRMS** (ES+) exact mass calculated for $[M+Na]^+$ ($C_{27}H_{49}NNaO_3+$) requires *m/z 458.3605*, found *m/z 458.3599*. **HPLC:** ee = 83% (Chiralpak AD-H, hexane/isopropanol 90:10, 210 nm, 1.0 mL/min) t_R (major) = 7.33, t_R minor = 6.3).

Synthesis and characterization of (-)-3l

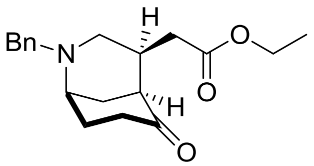

(-)-3l was obtained according to **general procedure G** using **2l** (50 mg, 0.158 mmol), Jacobsen catalyst **4k** (3.5 mg, 0.0079 mmol) and benzoic acid (0.48 mg, 0.0039 mmol) after 3.5 days. The crude reaction mixture was purified by flash column chromatography (PE/Et_2O = 3:2) to afford 41.9 mg (84%) of product as a solid as viscous oil.

$[\alpha]_D^{20}$ = -20.9 (C 0.41, $CHCl_3$); **FT-IR** ν_{max}/cm^{-1} 2932, 2361, 1731, 1701; **^{1}H NMR** (500 MHz, $CDCl_3$) δ 7.44 – 7.22 (m, 5H), 4.09 (qd, *J* = 7.1, 3.0 Hz, 2H), 3.82 – 3.60 (m, 2H), 3.05 (brs, 1H), 2.83 (brs, 1H), 2.68 – 2.49 (m, 3H), 2.41 – 2.12 (m, 4H), 2.07 – 1.92 (m, 2H), 1.78 – 1.51 (m, 2H), 1.20 (t, *J* = 7.1 Hz, 3H); **^{13}C NMR** (100 MHz, $CDCl_3$) δ 213.3, 171.8, 138.2, 128.7 (2C), 128.3 (2C), 127.1, 60.4, 59.1, 52.1, 48.9, 47.4, 39.8, 36.4, 34.8, 32.5, 21.9, 14.0; **HRMS** (ESI+) calcd. for $C_{19}H_{25}NNaO_3$ $[M+Na]^+$ 338.1727, found 338.1720. HPLC: ee = 96% (Chiralcel AD-H, 90:10 hexane/isopropanol, flow rate 1.0 ml/min, 210 nm, t_R (major) = 7.8 min, t_R (minor) = 6.9 min.

Synthesis and characterization of (-)-3m:

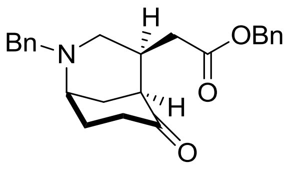

(-)-3m was obtained according to **general procedure G** using **2m** (50 mg, 0.132 mmol), Jacobsen catalyst **4k** (2.4 mg, 0.0066 mmol) and benzoic acid (0.40 mg, 0.0033 mmol) after 3.5 days. The crude reaction mixture was purified by flash column chromatography (PE/Et_2O = 3:2) to afford 44.2 mg (89%) of product as a solid as a solid.

mp: 100-102 °C; **[α]$_D$^{20}** = -5.1 (C 1.3, $CHCl_3$); **FT-IR** ν_{max}/cm^{-1} 2934, 2360, 1730, 1701; **^{1}H NMR** (400 MHz, $CDCl_3$) δ 7.40 – 7.22 (m, 10H), 5.09 (s, 2H), 3.74, 3.65 (ABq, *J* = 13.5 Hz, 2H), 3.09 – 2.99 (m, 1H), 2.83 (dd, *J* = 11.9, 5.1 Hz, 1H), 2.67 – 2.49 (m, 3H), 2.43 – 2.21 (m, 4H), 2.21 – 2.12 (m, 1H), 2.07 (dd, *J* = 16.3, 7.3 Hz, 1H), 1.99 (dt, *J* = 13.2, 3.1 Hz, 1H), 1.77 – 1.62 (m, 1H); **^{13}C NMR** (100 MHz, $CDCl_3$) δ 213.4, 171.6, 138.6, 135.7, 128.5 (2xC), 128.4 (2xC), 128.3 (2xC), 128.1, 128.0 (2xC), 127.0, 66.2, 59.1, 52.3, 48.9, 47.4, 39.8, 36.3, 35.0, 32.6, 21.8; **HRMS** (ESI+) calcd. for $C_{24}H_{27}NNaO_3$ $[M+Na]^+$ 400.1883, found 400.1870. **HPLC:** ee = 97% (Chiralcel AD-H, 90:10 hexane/isopropanol, flow rate 1.0 ml/min, 210 nm, t_R (major) = 13.4 min, minor t_R (minor) = 11.2 min.

X-Ray data available for this compound:

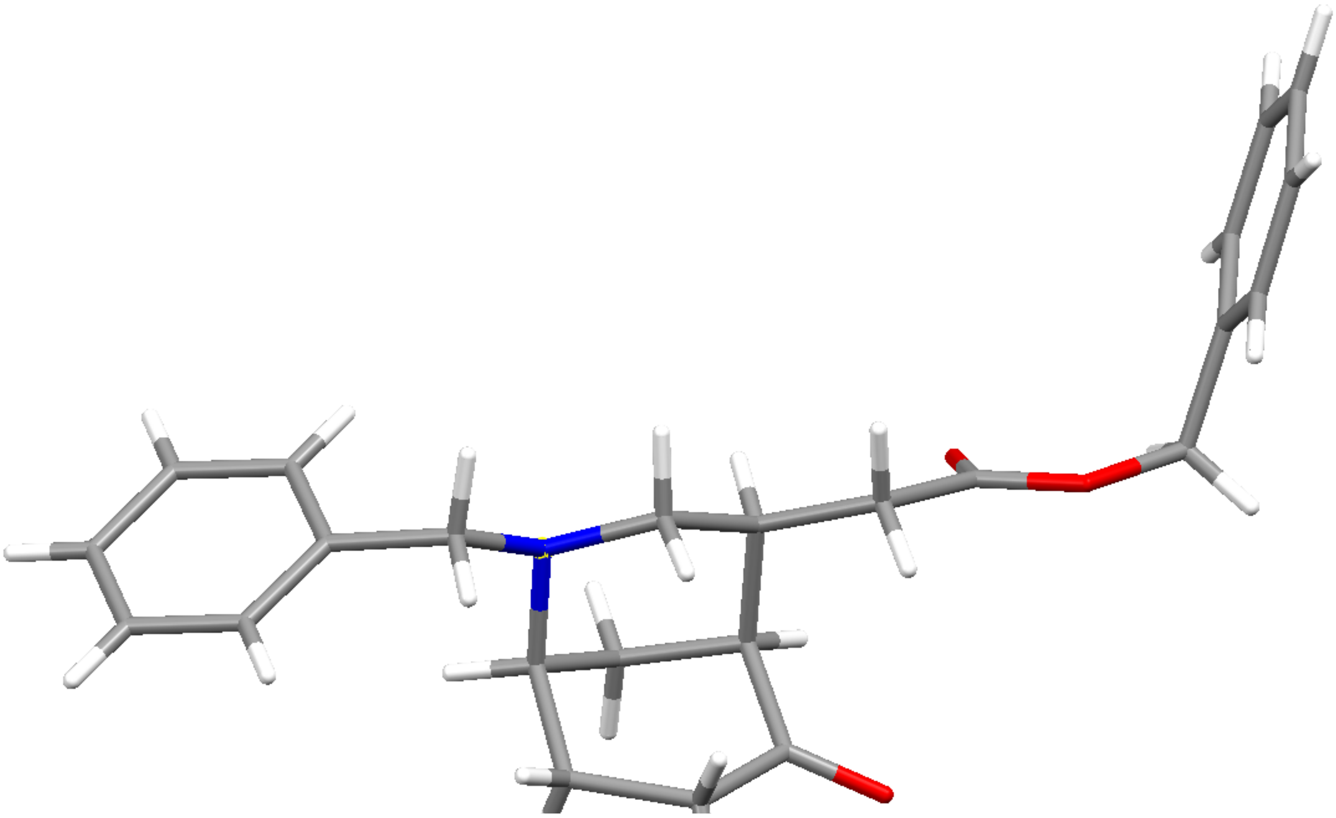

Single crystal X-ray structure of **3m**

Full crystallographic data (in CIF format) is available as ESI and has been deposited with the Cambridge Crystallographic Data Centre (reference code **CCDC 1026421**).

Synthesis and characterization of (-)-3n

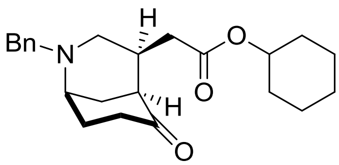

(-) 3n was obtained according to **general procedure G** using **2n** (50 mg, 0.135 mmol), Jacobsen catalyst **4k** (2.5 mg, 0.0067 mmol) and benzoic acid (0.40 mg, 0.0033 mmol) after 5 days. The crude reaction mixture was purified by flash column chromatography (PE/Et_2O = 3:2) to afford 42.5 mg (85%) of product as viscous oil.

$[\alpha]_D^{20}$ = -15.7 (C 0.61, $CHCl_3$); **FT-IR** ν_{max}/cm^{-1} 2932, 1733, 1701; **^{1}H NMR** (400 MHz, $CDCl_3$) δ 7.43 – 7.11 (m, 5H), 4.78 – 4.60 (m, 1H), 3.75, 3.64 (ABq, *J* = 13.4 Hz, 2H), 3.12 – 2.99 (m, 1H), 2.82 (dd, *J* = 12.0, 5.1 Hz, 1H), 2.65 – 2.46 (m, 3H), 2.39 – 2.11 (m, 5H), 2.05 – 1.88 (m, 2H), 1.80 – 1.59 (m, 5H), 1.57 – 1.44 (m, 1H), 1.38 – 1.13 (m, 5H); **^{13}C NMR** (100 MHz, $CDCl_3$) δ 213.3, 171.2, 138.7, 128.6 (2C), 128.2 (2C), 127.0, 72.6, 59.3, 52.0, 49.4, 47.7, 39.9, 37.0, 35.1, 32.9, 31.4 (2C), 25.2, 23.6 (2C), 22.1; **HRMS** (ESI+) calcd. for $C_{23}H_{31}NNaO_3$ $[M+Na]^+$ 392.2196, found 392.2185. **HPLC:** ee = 96% (Chiralcel AD-H, 90:10 hexane/isopropanol, flow rate 1.0 ml/min, 210 nm, t_R (major) = 7.8 min, minor t_R (minor) = 7.1 min.

Synthesis and characterization of (-)-3o:

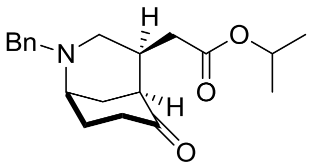

(-)-3o was obtained according to **general procedure G** using **2o** (50 mg, 0.152 mmol), Jacobsen catalyst **4k** (2.8 mg, 0.0075 mmol) and benzoic acid (0.46 mg, 0.0038 mmol) after 5 days. The crude reaction mixture was purified by flash column chromatography (PE/Et_2O = 3:2) to afford 38.0 mg (76%) of product as viscous oil.

[α]$_D$^{20} = -12.7 (C 0.33, $CHCl_3$); **FT-IR** ν_{max}/cm^{-1} 2932, 1733, 1701; **^{1}H NMR** (500 MHz, $CDCl_3$) δ 7.40 – 7.23 (m, 5H), 5.04 – 4.83 (m, 1H), 3.73 (t, *J* = 26.2 Hz, 2H), 3.06 (brs, 1H), 2.83 (brs, 1H), 2.66 – 2.42 (m, 3H), 2.37 – 2.21 (m, 4H), 1.98 (ddd, *J* = 22.3, 12.6, 5.5 Hz, 2H), 1.66 (d, *J* = 41.6 Hz, 2H), 1.16 (d, *J* = 6.5 Hz, 3H), 1.15 (d, *J* = 6.5 Hz, 3H); **^{13}C NMR** (125 MHz, $CDCl_3$) δ 213.3, 171.3, 138.8, 128.6 (2C), 128.3 (2C), 127.0, 67.8, 59.3, 52.1, 49.3, 47.6, 39.9, 36.9, 35.0, 32.9, 29.6, 21.7, 21.6; **HRMS** (ESI+) calcd. for $C_{20}H_{28}NO_3$ $[M+H]^+$ 330.2064, found 330.2060. **HPLC:** ee = 99% (Chiralcel AD-H, 90:10 hexane/isopropanol, flow rate 1.0 ml/min, 210 nm, t_R (major) = 6.7 min, t_R (minor) = 5.8 min.

Synthesis and characterization of (-)-3p

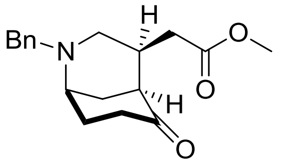

(-)-3p was obtained according to **general procedure G** using **3p** (50 mg, 0.166 mmol), Jacobsen catalyst **4k** (3.1 mg, 0.0083 mmol) and benzoic acid (0.51 mg, 0.0041 mmol) after 4 days. The crude reaction mixture was purified by flash column chromatography (PE/Et_2O = 3:2) to afford 45.0 mg (90%) of product as viscous oil.

[α]$_D$^{20} = -17.8 (C 1.3, $CHCl_3$); **FT-IR** ν_{max}/cm^{-1} 1733, 1701; **^{1}H NMR** (400 MHz, $CDCl_3$) δ 7.46 – 7.13 (m, 5H), 3.66 (ABq, *J* = 13.5 Hz, 2H), 3.64 (s, 3H), 3.09 – 2.98 (m, 1H), 2.83 (dd, *J* = 11.9, 5.0 Hz, 1H), 2.65 – 2.48 (m, 3H), 2.38 – 2.10 (m, 5H), 2.08 – 1.92 (m, 2H), 1.80 – 1.60 (m, 1H); **^{13}C NMR** (100 MHz, $CDCl_3$) δ 213.4, 172.3, 138.6, 128.5 (2C), 128.3 (2C), 127.0, 59.1, 52.3, 51.6, 48.8, 47.4, 39.8, 36.0, 34.9, 32.7, 21.8; **HRMS** (ESI+) calcd. for $C_{18}H_{23}NNaO_3$ $[M+Na]^+$ 324.1570, found 324.1557. **HPLC:** ee = 97% (Chiralcel AD-H, 90:10 hexane/isopropanol, flow rate 1.0 ml/min, 210 nm, t_R (major) = 8.3 min, t_R (minor) = 7.4 min.

Synthesis and characterization of (-)-3q:

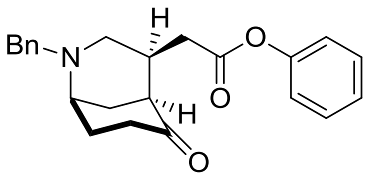

(-)-3q was obtained according to **general procedure G** using **2q** (50 mg, 0.137 mmol), Jacobsen catalyst **4k** (2.5 mg, 0.0068 mmol) and benzoic acid (0.42 mg, 0.0034 mmol) after 4 days. The crude reaction mixture was purified by flash column chromatography (PE/Et_2O = 3:2) to afford 36.0 mg (72%) of product as viscous oil.

$[\alpha]_D^{20}$ = -10.3 (C 1.5, $CHCl_3$); **FT-IR** ν_{max}/cm^{-1} 1753, 1701, **^{1}H NMR** (400 MHz, $CDCl_3$) δ 7.42 – 7.24 (m, 7H), 7.21 (t, *J* = 7.4 Hz, 1H), 6.98 (d, *J* = 7.6 Hz, 2H), 3.78 (d, *J* = 13.4 Hz, 1H), 3.68 (d, *J* = 13.4 Hz, 1H), 3.11 (brs, 1H), 2.92 (dd, *J* = 11.9, 4.7 Hz, 1H), 2.75 – 2.53 (m, 4H), 2.44 – 2.17 (m, 5H), 2.09 – 1.98 (m, 1H), 1.81 – 1.69 (m, 1H); **^{13}C NMR** (100 MHz, $CDCl_3$) δ 213.4, 170.5, 150.5, 129.3 (2C), 128.6 (2C), 128.5, 128.4 (2C), 127.1, 125.8, 121.5 (2C), 59.3, 51.9, 49.4, 47.3, 39.8, 36.5, 35.2, 32.6, 21.8; **HRMS** (ESI+) calcd. for $C_{23}H_{25}NNaO_3$ $[M+Na]^+$ 386.1727, found 386.1709. **HPLC:** ee = 97% (Chiralcel AD-H, 90:10 hexane/isopropanol, flow rate 1.0 ml/min, 210 nm, t_R (major) = 13.7 min, t_R (minor) = 11.5 min.

(1R,4S,5S)-tert-butyl 4-(2-methoxy-2-oxoethyl)-6-oxo-2-azabicyclo[3.3.1]nonane-2-carboxylate (-)-3r

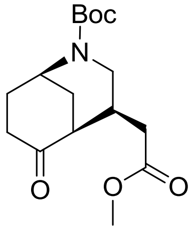

(-)-3r was obtained according to **general procedure G** using **2r** (37.5 mg, 0121 mmol), Jacobsen's catalyst **4k** (5 mol%) and benzoic acid (2.5 mol%) for 96 h. The crude reaction mixture was purified by flash column chromatography (PE/EtOAc = 1:1) to afford 32 mg (85%) of product as a solid.

mp:125-127 °C **[α]$_D$ ^{20}** = -15.6 (C 1.4, $CHCl_3$); ν_{max}/cm^{-1} 1734, 1685; **^{1}H NMR** (400 MHz, MeOD) δ 4.35 (d, *J* = 11.9 Hz, 1H), 4.00 (dd, *J* = 13.7, 2.9 Hz, 1H), 3.57 (s, 3H), 2.71 (t, *J* = 13.2 Hz, 1H), 2.52 – 2.39 (m, 2H), 2.34 – 2.15 (m, 3H), 2.11 – 1.84 (m, 5H), 1.38 (s, 9H); **^{13}C NMR** (101 MHz, MeOD) δ 212.6, 172.1, 155.3, 80.1, 50.8, 47.1, 44.6, 43.9, 38.3, 35.2, 34.5, 31.6, 29.1, 27.3 (3C). **FT-IR** ν_{max}/cm^{-1} 2932, 2361, 1731, 1701; **HRMS** (ESI+) calcd. for $[M+Na]^+$ $C_{16}H_{25}NNaO_5$ 334.1625 found 334.1627.

Compound **3r** did not separate on the available to us and so was derivatized to give **3p**.

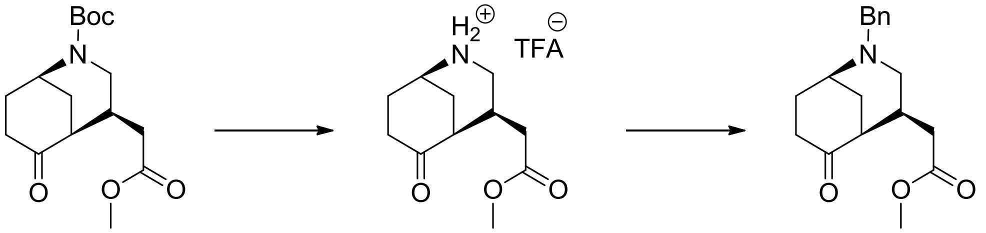

3r **3r'** **3p**

To a solution of **(-)-3r** (1 eq., 0.0964 mmol, 30 mg) in CH_2Cl_2 (0.44 ml) at 0 °C was added TFA (0.147 ml). The reaction mixture was maintained at this temperature for 3 hours. The reaction mixture was then diluted with toluene (1 ml) at 0 °C and concentrated *in vacuo* at RT. Once all the CH_2Cl_2 and TFA were removed the toluene was removed *in vacuo* at 30 °C to give the compound **(-)-3r'** as an off white solid gum. To a solution of this solid in acetonitrile (0.5 ml) was added K_2CO_3 (3.2 eq., 0.369 mmol, 53 mg) and BnBr (1.2 eq., 0.116 mmol, 0.020 ml) and stirred at this temperature for 24 hours. The solution was *in vacuo* and the resulting brown oil was purified by FCC (PE: EtOAc 80:20 → 60:40) to give title compound **3p** as a yellow oil (86%, 25 mg) over two steps. Data as before.

[α]$_D$ ^{20} = -18.6 (C 1.4, $CHCl_3$); **HPLC:** ee = 98% (Chiralcel AD-H, 90:10 hexane/isopropanol, flow rate 1.0 ml/min, 210 nm, t_R (major) = 9.0 min, t_R (minor) = 7.9 min.

methyl 2-((1R,4S,5S)-2-allyl-6-oxo-2-azabicyclo[3.3.1]nonan-4-yl)acetate (-)-3s

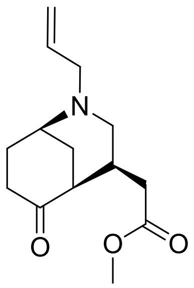

(-)-3s was obtained according to **general procedure G** using **2s** (74 mg, 0.159 mmol), Jacobsen's catalyst **4k** (5 mol%) and benzoic acid (2.5 mol%) for 96 h. The crude reaction mixture was purified by flash column chromatography (PE/EtOAc = 3:2) to afford 65 mg (87%) of product as viscous oil.

[α]$_D$^{20} = -17.3 (C 1.5, $CHCl_3$); **FT-IR** ν_{max}/cm^{-1} 1733, 1701, 1643; **^{1}H NMR** (400 MHz, Chloroform-*d*) δ 5.90 – 5.69 (m, 1H), 5.22 – 5.02 (m, 2H), 3.60 (s, 3H), 3.12 (m, 3H), 2.89 – 2.78 (m, 1H), 2.59 – 2.40 (m, 3H), 2.32 – 2.01 (m, 5H), 2.02 – 1.89 (m, 2H), 1.70 – 1.52 (m, 1H). **^{13}C NMR** (101 MHz, Chloroform-*d*) δ 213.2, 172.2, 135.5, 117.6, 58.3, 52.3, 51.7, 49.2, 47.5, 39.9, 36.2, 34.9, 32.7, 21.8. **HRMS** (ES+) exact mass calculated for $[M+H]^+$ ($C_{14}H_{22}NO_3$) requires *m/z* 252.1594, found *m/z* 252.1594. **HPLC**: ee = 97% (Chiralcel AD-H, 90:10 hexane/isopropanol, flow rate 1.0 ml/min, 210 nm, t_R (major) = 8.6 min, t_R (minor) = 6.9 min.

methyl 2-((1R,4S,5S)-2-(diphenylphosphoryl)-6-oxo-2-azabicyclo[3.3.1]nonan-4-yl)acetate (-)-3t

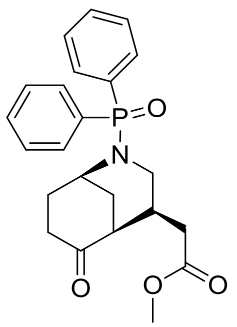

(-)-3t was obtained according to **general procedure G** using **2t** (47.4 mg, 0.073 mmol), Jacobsen's catalyst **4k** (5 mol%) and benzoic acid (2.5 mol%) for 96. The crude reaction mixture was purified by flash column chromatography (PE/EtOAc = 3:2) to afford 37.5 mg (79%) of product as viscous oil.

[α]$_D$ ^{20} = -10.7 (C 1.4, $CHCl_3$). **FT-IR** ν_{max}/cm^{-1} 1733, 1701, 1438;**^{1}H NMR** (400 MHz, $CDCl_3$) δ 7.85 – 7.75 (m, 4H), 7.48 – 7.34 (m, 6H), 3.66 (d, *J* = 9.0 Hz, 1H), 3.47 (s, 3H), 3.13 (ddd, *J* = 13.4, 7.7, 5.5 Hz, 1H), 2.86 (td, *J* = 13.0, 6.3 Hz, 1H), 2.66 – 2.60 (m, 1H), 2.52 – 2.31 (m, 3H), 2.29 – 2.09 (m, 3H), 2.04 – 1.84 (m, 3H); **^{13}C NMR** (101 MHz, $CDCl_3$) δ 212.38, 171.75, 132.24 (d, *J* = 5.9 Hz), 132.15 (d, *J* = 6.2 Hz), 132.00 (d, *J* = 2.7 Hz), 128.78 (d, *J* = 12.5 Hz), 51.68, 47.09, 44.56, 44.54, 39.22, 35.73, 35.59 (d, *J* = 6.2 Hz), 32.50 (d, *J* = 5.9 Hz), 29.33 (d, *J* = 2.1 Hz). **HRMS** (ESI+) calcd. for $[M+Na]^+$ $C_{23}H_{26}NNaO_4P$ 434.1492 found 434.1490.

Compound **3t** did not separate on the available to us and so was derivatized to give **3p**.

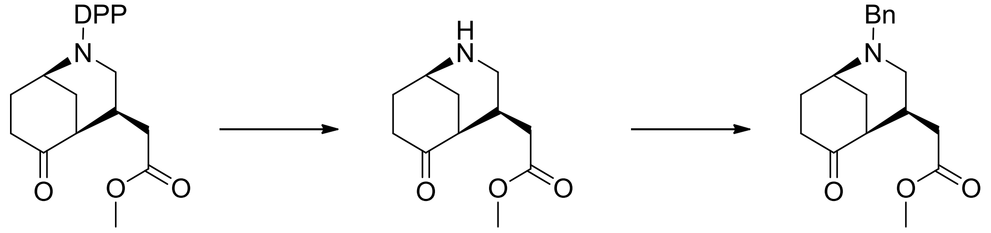

3t **3t'** **3p**

Substrate **(-)-3u** was deprotected according to a modified literature procedure. [11] To a stirring solution of **(-)-3t** (1eq., 0.072 mmol, 30 mg) in methanol 0.2 ml was added HCl (*aq*) *conc* (0.48 ml). After stirring for 30min at RT, the reaction mixture was diluted with H_2O (2 mL) and extracted with EtOAc (3x1.5 mL). To the aqueous phase was added NaOH (1M) was added until pH 7 was attained and subsequently concentrated *in vacuo* (30 °C) azeotroping with toluene (4 x 5 ml) to remove the water to give a brown gum **(-)-3t'**. To a solution of this residue in acetonitrile (0.5 ml) was added K_2CO_3 (2.2 eq., 0.168 mmol, 22 mg) and BnBr (1.2 eq., 0.088 mmol, 0.011 ml) and stirred at this temperature for 24 hours. The solution was *in vacuo* and the resulting brown oil was purified by FCC (PE: EtOAc 80:20 → 60:40) to give title compound **3p** as a yellow oil (78%, 17 mg).

[α]$_D$ ^{20} = -16.2 (C 1.0, $CHCl_3$); **HPLC:** ee = 95% (Chiralcel AD-H, 90:10 hexane/isopropanol, flow rate 1.0 ml/min, 210 nm, t_R (major) = 9.2 min, t_R (minor) = 8.2 min.

[11] (1) Ferna, I.; Roces, L.; Torre-ferna, L.; Garcı, S.; Lo, F. *Org. Lett.* **2008**, 10, 3195-3198.

(E)-methyl 4-((1R,4S,5S)-4-(2-methoxy-2-oxoethyl)-6-oxo-2-azabicyclo[3.3.1]nonan-2-yl)but-2-enoate (-)-3u

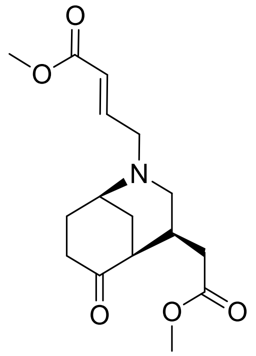

(-)-3u was obtained according to **general procedure G** using **2u** (50 mg, 0.162 mmol), Jacobsen's catalyst **4k** (5 mol%) and benzoic acid (2.5 mol%) for 96h. The crude reaction mixture was purified by flash column chromatography (PE/EtOAc = 3:2) to afford 45 mg (90%) of product as viscous oil.

$[\alpha]_D^{20}$ = -24.3 (C 1.1, $CHCl_3$); **FT-IR** ν_{max}/cm^{-1} 2948, 1722, 1700 1658; **^{1}H NMR** (300 MHz, $CDCl_3$) δ 6.87 (dt, J = 15.7, 5.9 Hz, 1H), 5.97 (dt, J = 15.7, 1.5 Hz, 1H), 3.68 (s, 3H), 3.60 (s, 3H), 3.26 (dddd, J = 33.3, 15.6, 5.8, 1.3 Hz, 2H), 3.02 (s, 1H), 2.78 (dd, J = 11.8, 4.9 Hz, 1H), 2.57 – 2.39 (m, 3H), 2.31 – 2.05 (m, 5H), 2.03 – 1.89 (m, 2H), 1.73 – 1.59 (m, 1H). **^{13}C NMR** (75 MHz, $CDCl_3$) δ 212.82, 172.12, 166.58, 145.66, 122.64, 55.89, 52.45, 51.66, 51.58, 49.78, 47.13, 39.65, 36.02, 34.90, 32.53, 22.04; **HRMS** (ESI+) calcd. for $[M+H]^+$ $C_{16}H_{24}NO_5$ 310.1649 found 310.1653. **HPLC:** ee = 97% (Chiralcel AD-H, 90:10 hexane/isopropanol, flow rate 1.0 ml/min, 210 nm, t_R (major) = 22.6 min, t_R (minor) = 20.0 min.

methyl 2-((1R,4S,5S)-2-methyl-6-oxo-2-azabicyclo[3.3.1]nonan-4-yl)acetate (-)-3v:

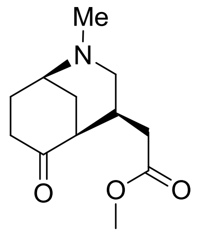

(-)-3v was obtained according to **general procedure G** using **2v** (50 mg, 0.222 mmol), Jacobsen's catalyst **4k** (5 mol%) and benzoic acid (2.5 mol%) for 96h. The crude reaction mixture was purified by flash column chromatography (PE/EtOAc = 3:2) to afford 42 mg (84%) of product as viscous oil.

$[\alpha]_D^{20}$ = -13.3 (C 1.6, $CHCl_3$); **FT-IR** ν_{max}/cm^{-1} 2955, 1726, 1680; **^{1}H NMR** (400 MHz, $CDCl_3$) δ 3.68 (s, 3H), 3.05 – 2.99 (m, 1H), 2.83 (dd, *J* = 11.9, 5.1 Hz, 1H), 2.61 – 2.49 (m, 3H), 2.43 (s, 3H), 2.35 (dd, *J* = 16.2, 6.3 Hz, 1H), 2.31 – 2.25 (m, 2H), 2.24 – 2.12 (m, 2H), 2.07 – 2.00 (m, 2H), 1.68 (dtd, *J* = 14.3, 9.2, 5.4 Hz, 1H); **^{13}C NMR** (101 MHz, $CDCl_3$) δ 213.08, 172.24, 54.18, 51.66, 51.32, 47.10, 42.82, 39.88, 36.25, 35.06, 32.93, 21.18; **HPLC:** ee = 96% (Chiralcel AD-H, 70:30 hexane/isopropanol, flow rate 1.0 ml/min, 210 nm, t_R (major) = 17.6 min, t_R (minor) = 11.8 min. **HRMS** (ESI+) calcd. for $[M+H]^+$ $C_{12}H_{20}NO_3$+ 226.1438 found 226.1431

Synthesis and characterization of catalyst 4l

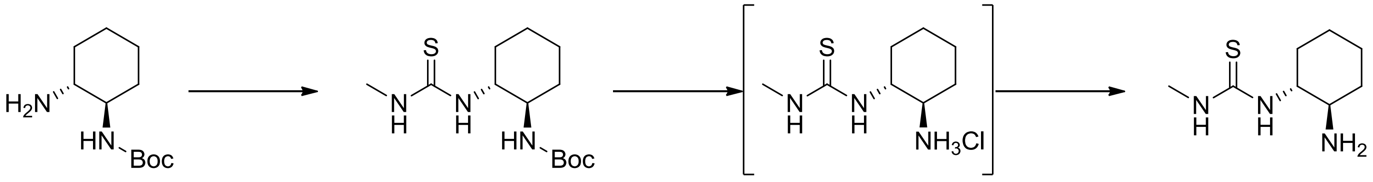

4j 35 36 4l

tert-butyl ((1R,2R)-2-(3-methylthioureido)cyclohexyl)carbamate (35):

To a solution of (1R,2R)-trans-N-Boc-1,2-cyclohexanediamine **4j** (1 eq., 0.9906 mmol, 212 mg) in CH_2Cl_2 (4.6 ml) was added methyl isothiocyanate (3eq., 2.97 mmol, 217 mg) at room temperature and stirred for 24 h. After this the reaction mixture was concentrated *in vacuo* and the resulting gum was purified by FCC (EtOAc:Pe 80:20) to give **35** as a white gum (63%, 179 mg)

$[\alpha]_D^{20}$ = -4.3 (C 2.1, $CHCl_3$); **FT-IR** ν_{max}/cm^{-1} 3253, 2934, 1681, 1555, 1511; **^{1}H NMR** (400 MHz, MeOD) δ 4.07 (s, 1H), 2.89 (bs, 3H), 2.12 – 1.90 (m, 2H), 1.74 (s, 2H), 1.41 (s, 9H), 1.32 (m, 5H). **^{13}C NMR** (101 MHz, CD_3CN) δ 157.4, 79.5, 66.2, 55.1, 33.1, 32.8, 28.6 (3C), 25.6, 25.4, 15.6. $\underline{C}$S undetected; **HRMS** (ESI+) calcd. for $[M+H]^+$ $C_{13}H_{26}N_3O_2S+$ 288.1740 found 288.1739

(1R,2R)-2-(3-methylthioureido)cyclohexanaminium chloride (36):

To a solution of **35** (1 eq., 0.46 mmol, 132 mg) in CH_2Cl_2 (2.8 ml) at RT was added 4M HCl in dioxane (9.6 eq., 4.4 mmol, 1.1 ml) and the resulting solution was allowed to stir for 5 hours. The reaction mixture was diluted with toluene (12 ml) and concentrated *in vacuo* to give hydrochloride salt **36** as a off white gum (99%, 100 mg). Spectrum of the HCl salt is present to aid characterization of **4l** by NMR as some carbon signals belonging to **4l** are not observed.

FT-IR ν_{max}/cm^{-1} 2936,1561; **^{1}H NMR** (400 MHz, Methanol-d_4) δ 4.49 (s, 1H), 3.10 (s, 1H), 2.97 (s, 3H), 2.18 – 2.08 (m, 1H), 2.06 – 1.97 (m, 1H), 1.91 – 1.71 (m, 2H), 1.52 (qd, J = 12.4, 3.5 Hz, 1H), 1.46 – 1.28 (m, 3H). **^{13}C NMR** (101 MHz, MeOD) δ 56.12, 55.24, 31.68, 30.2, 30.2 (detected in HSQC), 24.63, 23.96. $\underline{C}$S undetected. **HRMS** (ESI+) calcd. for $[M+H]^+$ $C_8H_{18}N_3S+$ required 188.1216, found 188.1709.

1-methyl-3-((1R,2R)-2-(methylamino)cyclohexyl)thiourea (+)-4l:

The resulting residue **36** was dissolved in CH_2Cl_2 and free based with 1M NaOH (1 ml). The aq. layer was then saturated with NaCl and the aqueous phase was extracted with CH_2Cl_2 (6 x 1 ml). The combined organic extracts were dried over Na_2SO_4 and concentrated *in vacuo* to give pure title compound **4l** as an off white gum (quant, 28mg).

$[\alpha]_D^{20}$ = +7.3 (C 3.0, $CHCl_3$); **FT-IR** ν_{max}/cm^{-1} 2931, 1557, 1449; **^{1}H NMR** (400 MHz, MeOD) δ 3.93 (bs, 1H), 3.09 – 2.83 (m, 3H), 2.59 – 2.46 (m, 1H), 2.02 – 1.93 (m, 2H), 1.77 – 1.69 (m, 2H), 1.43 – 1.14 (m, 4H). **^{13}C NMR** (101 MHz, MeOD) δ 55.78, 35.17, 33.04, 26.17, 26.01. $\underline{C}H_3NC=S$, $\underline{C}=S$, $CH_3NHCSNH\underline{C}H$ undetected – carbons about the thiourea were undetected in the catalyst free base but present in the HCl salt. **HRMS** (ESI+) calcd. for $[M+H]^+$ $C_8H_{18}N_3S^+$ required 188.1216, found 188.1209

Cyclisation of 2v using truncated 4l

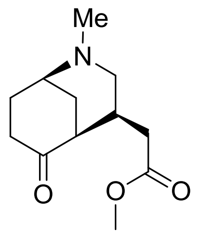

(-)-3v was obtained according to **general procedure G** using **2v** (70 mg), **4l** (5 mol%) and benzoic acid (2.5 mol%) for 96h. The crude reaction mixture was purified by flash column chromatography (Acetone) to afford 58 mg (84%) of product as viscous oil. Data as before.

$[\alpha]_D^{20}$ = -13.9 (C 1.5, $CHCl_3$); **HPLC:** ee = 97% (Chiralcel AD-H, 70:30 hexane/isopropanol, flow rate 1.0 ml/min, 210 nm, t_R (major) = 13.3 min, t_R (minor) = 9.9 min.

Single crystal preparation for X-ray analysis.[12]

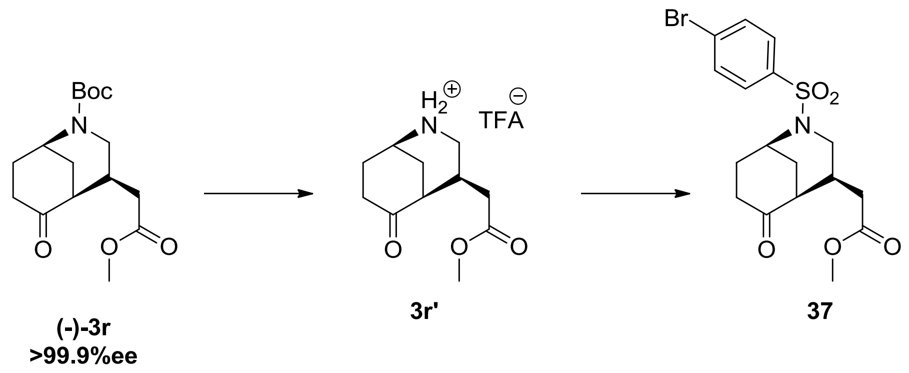

Compound **3r** is highly crystalline and could be crystallized to enantiopurity from refluxing methanol.

To a solution of **(-)-3r** (1 eq., 0.321 mmol, 100 mg) in CH_2Cl_2 (1.5 ml) at 0 °C was added TFA (0.5 ml). The reaction mixture was maintained at this temperature for 4 hours. The reaction mixture was then diluted with toluene (4 ml) at 0 °C and concentrated *in vacuo* at RT. Once all the CH_2Cl_2 and TFA were removed the toluene was removed *in vacuo* at 30 °C to give the compound **(-)-3r'** as an off white solid. To a solution of **3r'** (40 mg, 0.123 mmol) in CH_2Cl_2 (1ml) was added 4-Bromobenzenesulfonyl chloride (34 mg, .135 mmol, 1.1 eq.) and triethylamine (0.1 ml) and allowed to stir at room temperature for 24 h. The reaction mixture was concentrated *in vacuo* and purified by FCC (PE:Et_2O 1:1) to give 35 as a white solid (47 mg, 89%). Compound **37** was crystallized as a single crystal by slow evaporation from EtOAc in a vial with a small hole in the cap over 6 days.

mp: 143-145 °C; **[*α*]$_D$ ^{20}** = -7.2 (C 3, $CHCl_3$); **FT-IR** ν_{max}/cm^{-1} 2950, 1701, 1574; **^{1}H NMR** (400 MHz, CDCl3) δ 7.70 (ABq, *J* = 20.8, 8.6 Hz, 4H), 4.36 (m, 1H), 3.96 (dd, *J* = 13.6, 5.1 Hz, 1H), 3.69 (s, 3H), 2.88 (t, *J* = 12.8 Hz, 1H), 2.62 – 2.56 (m, 1H), 2.51 (ddd, *J* = 18.4, 8.7, 3.4 Hz, 1H), 2.40 – 2.30 (m, 2H), 2.29 – 2.15 (m, 1H), 2.11 – 1.83 (m, 5H). **^{13}C NMR** (101 MHz, CDCl3) δ 210.7**,** 171.5**,** 139.4**,** 132.6 (2C)**,** 128.5 (2C)**,** 127.8**,** 51.9**,** 46.4**,** 46.2**,** 45.3**,** 38.6**,** 35.4**,** 34.5**,** 31.8, 28.2; **HRMS** (ESI+) calcd. for $[M+Na]^+$ $C_{17}H_{20}BrNNaO_5S$+ requires 452.0138, found 452.0146.

---

[12] Low temperature single X-ray diffraction data were collected for **3p** and **37** using a Nonius KCCD diffractometer. Data were reduced using DENZO-SMN [Otwinowski & Minor, Processing of X-ray Diffraction Data Collected in Oscillation Mode, Methods Enzymol. (1997), 276, Eds C. W. Carter, R. M. Sweet, Academic Press.] and solved using SIR92 [A. Altomare, G. Cascarano, C. Giacovazzo, A. Guagliardi, M. C. Burla, G. Polidori & M. Camalli, J. Appl. Cryst. (1994), 27, 435]. Structures were refined using CRYSTALS [P. W. Betteridge, J. R. Carruthers, R. I. Cooper, K. Prout, & D. J. Watkin, J. Appl. Cryst. (2003), 36, 1487; R. I. Cooper, A. L. Thompson & D. J. Watkin, J. Appl. Cryst. (2010), 43, 1100-1107]. For **3p**, the Flack x parameter [H. D. Flack, Acta Cryst. (1983), A39, 876-881; H. D. Flack & G. Bernardinelli, J. Appl. Cryst. (2000), 33, 1143-1148] refined to -0.17 (118) (unrestrained) and -0.20(39) with the application of Bijvoet difference restraints [A. L. Thompson & D. J. Watkin, J. Appl. Cryst. (2011), 44, 1017-1022]. Bayesian analysis of the Bijvoet pairs [R. W. W. Hooft, L. H. Straver & A. L. Spek, J. Appl. Cryst. (2008), 41, 96-103] gave the Hooft y parameter as -0.33(28), G of 1.66(76), and the probability that the structure was the correct hand of >99.6% given that the crystal is enantiopure. For **37**, the Flack x parameter refined to 0.015(5) and Bayesian analysis of the Bijvoet pairs gave the Hooft y parameter as 0.0250(5), G of 0.9500(10), and the probability that the structure was the correct hand of >99.99% given that the crystal is enantiopure or a racemic twin. Full crystallographic data (in CIF format) is available as ESI and has been deposited with the Cambridge Crystallographic Data Centre (reference code **CCDC 1026422**).

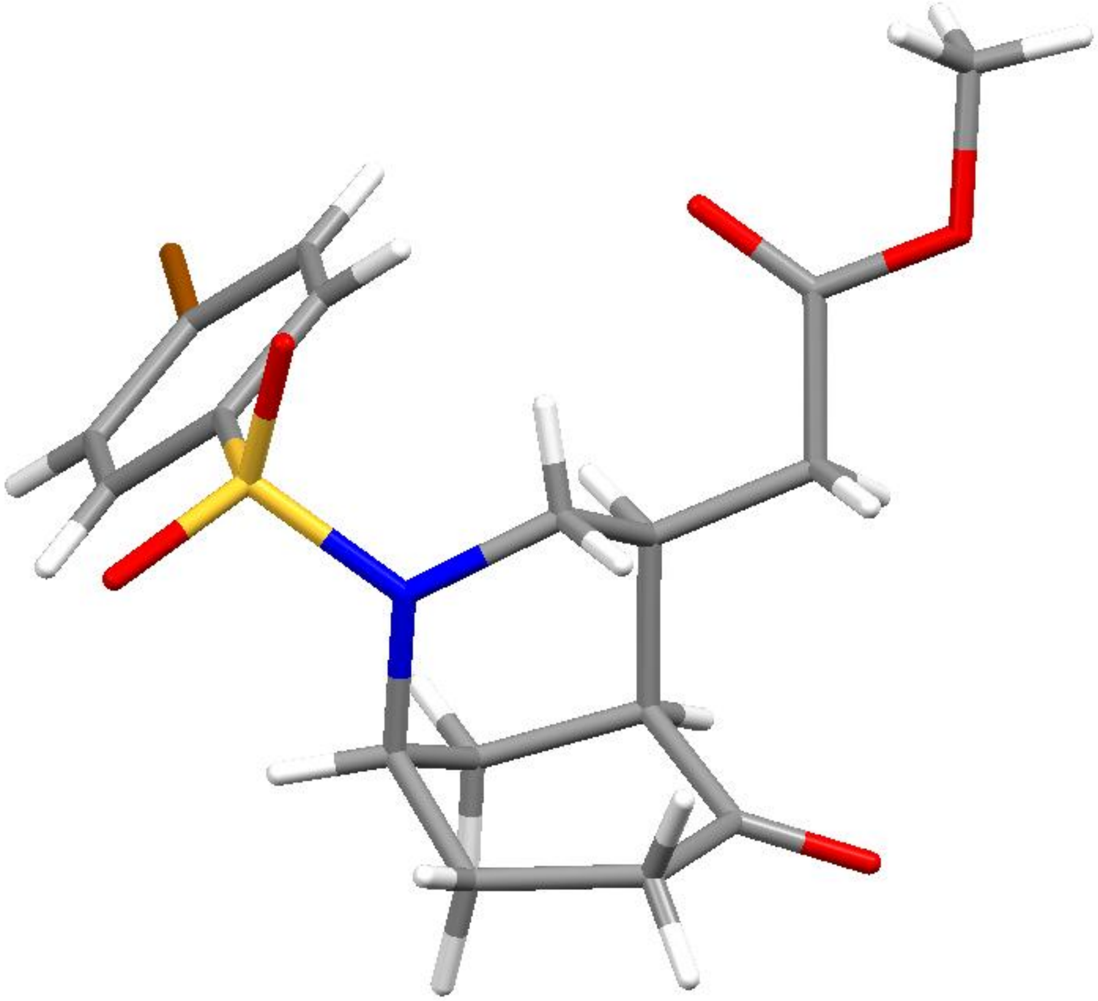

Single crystal X-ray structure of **37**

^{1}H NMR Spectrum of compound 2a

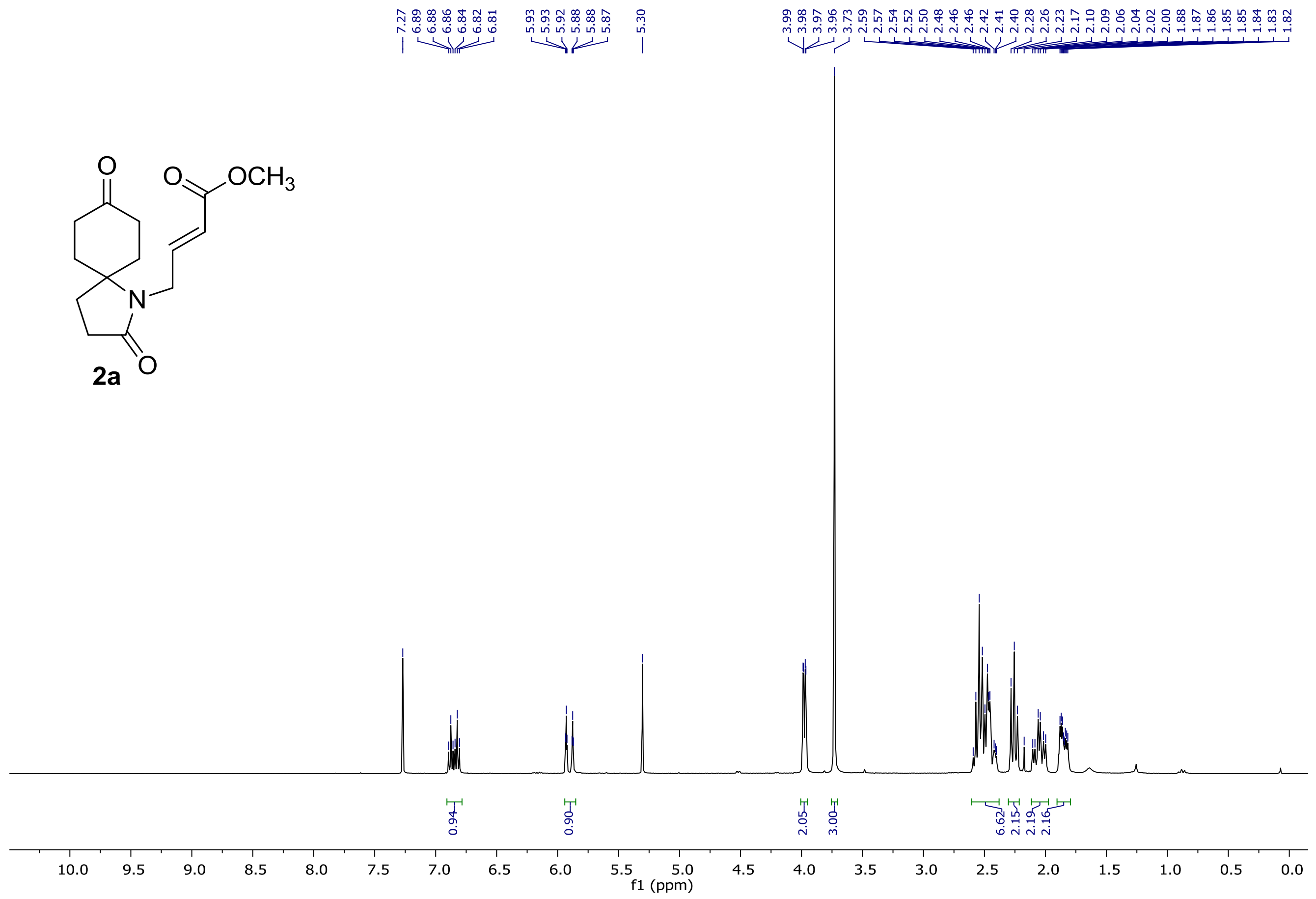

^{13}C NMR Spectrum of compound 2a

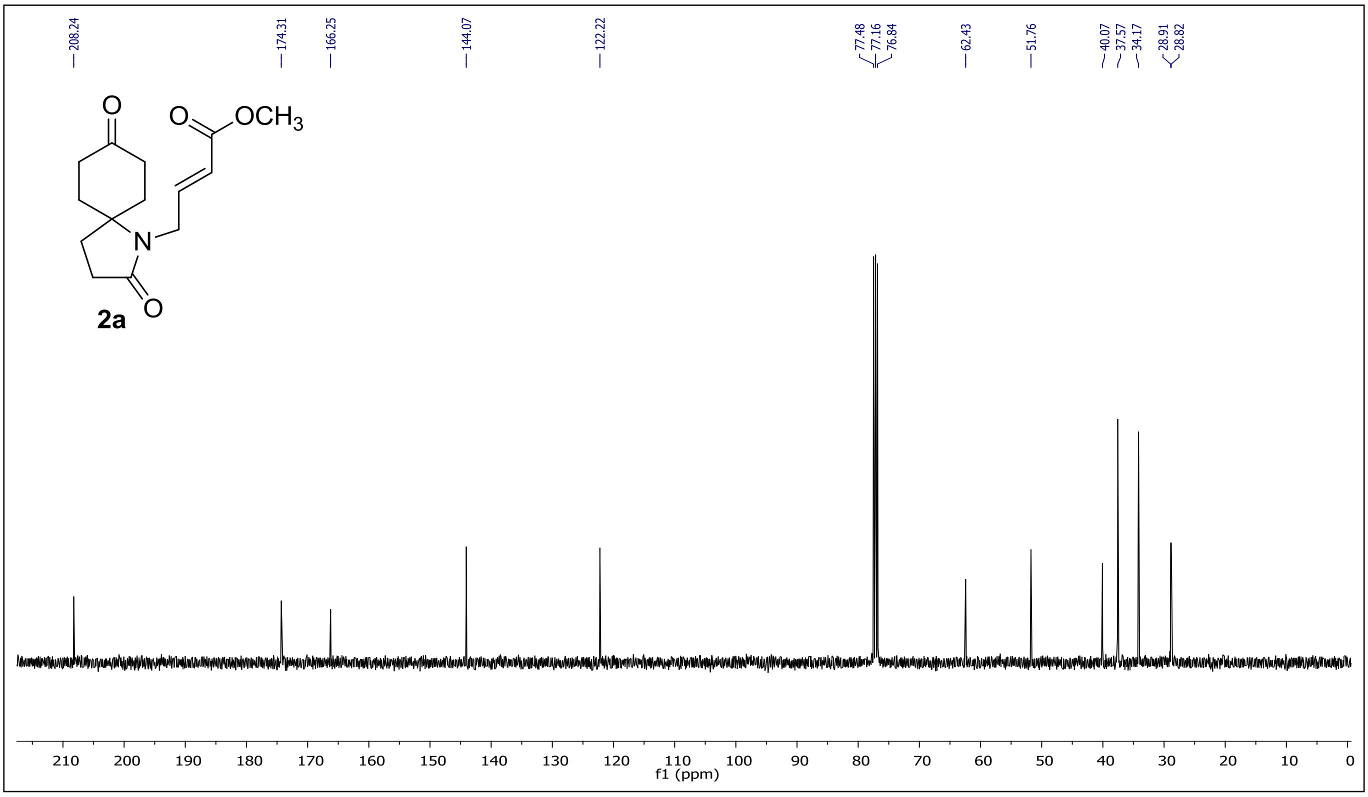

^{1}H NMR Spectrum of compound 2b

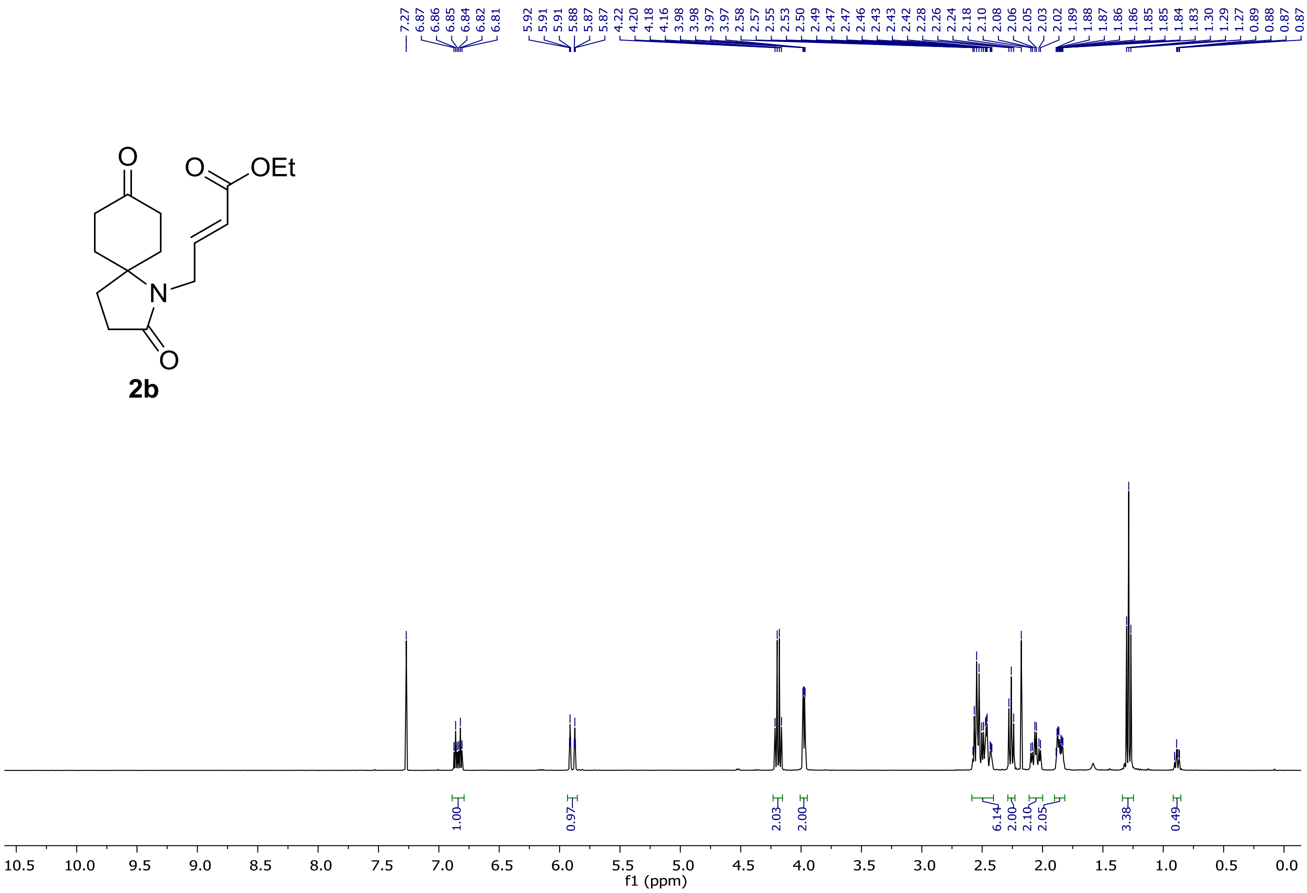

^{13}C NMR Spectrum of compound 2b

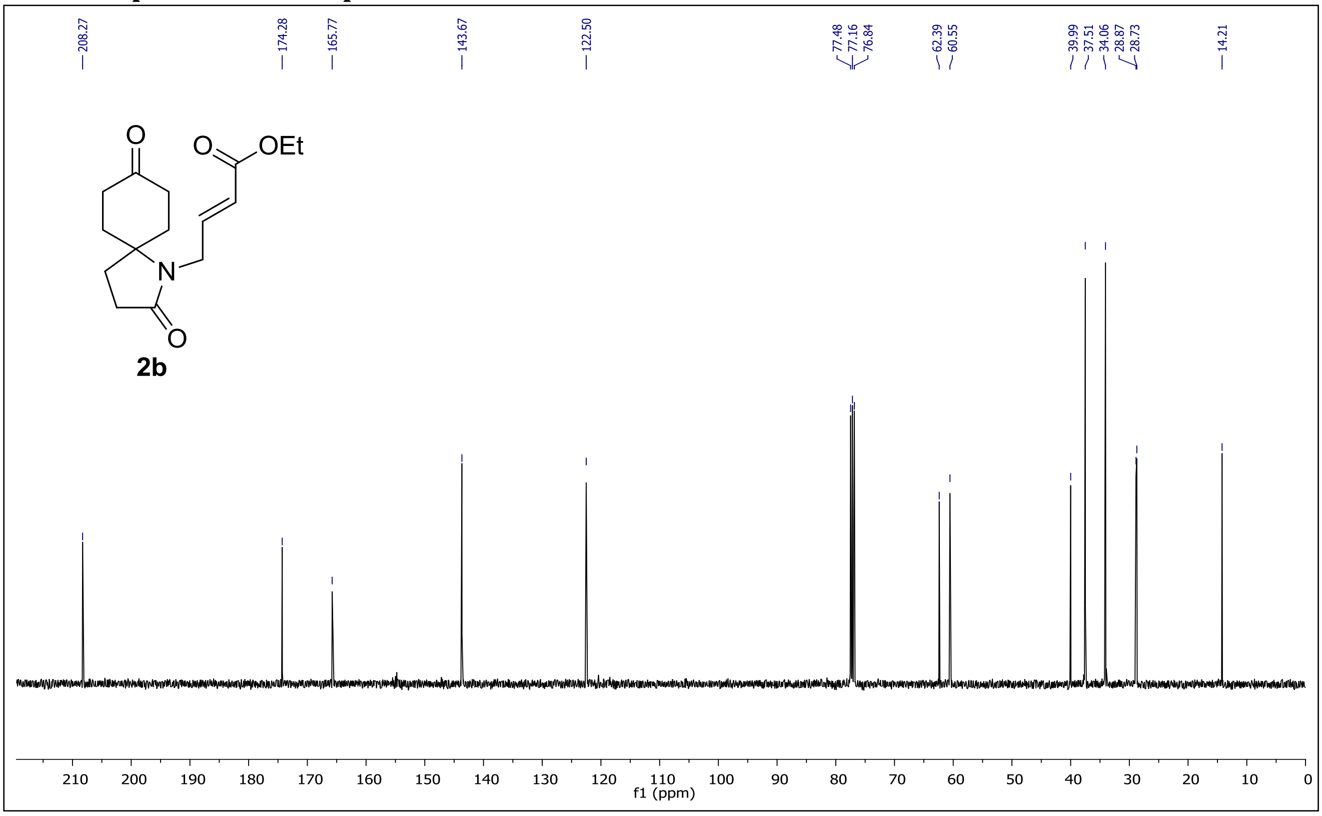

^{1}H NMR Spectrum of compound 2c

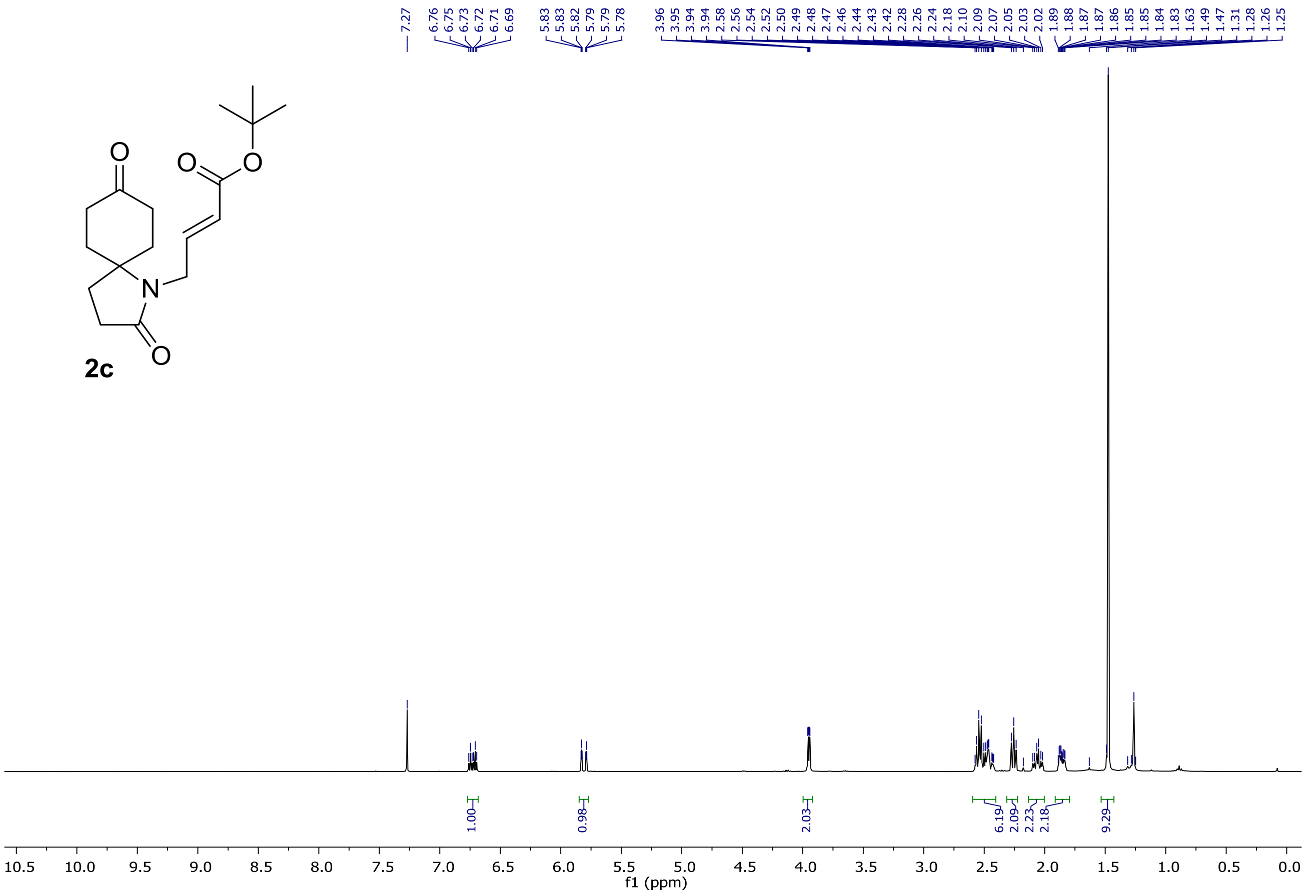

^{13}C NMR Spectrum of compound 2c

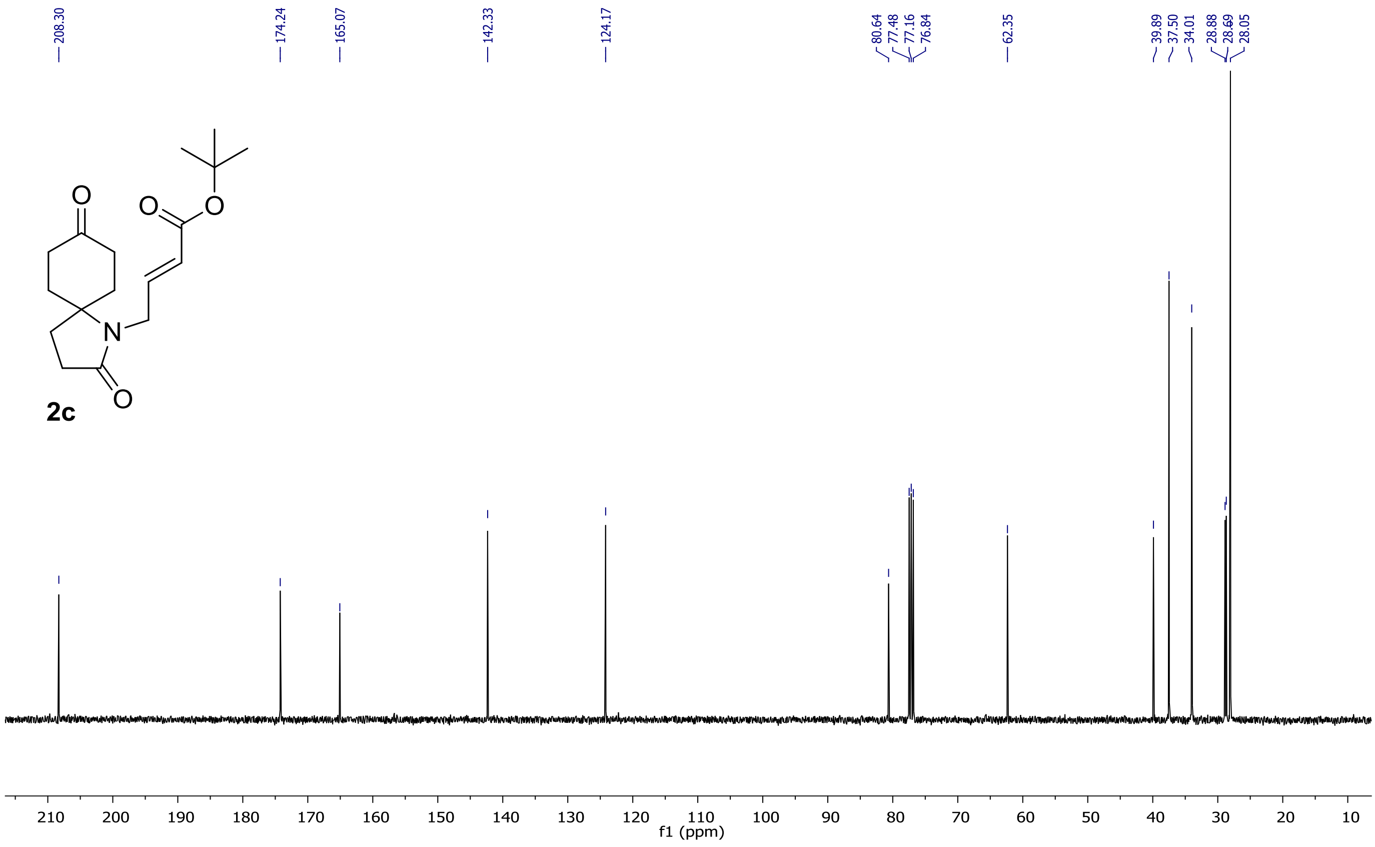

^{1}H NMR Spectrum of compound 2d

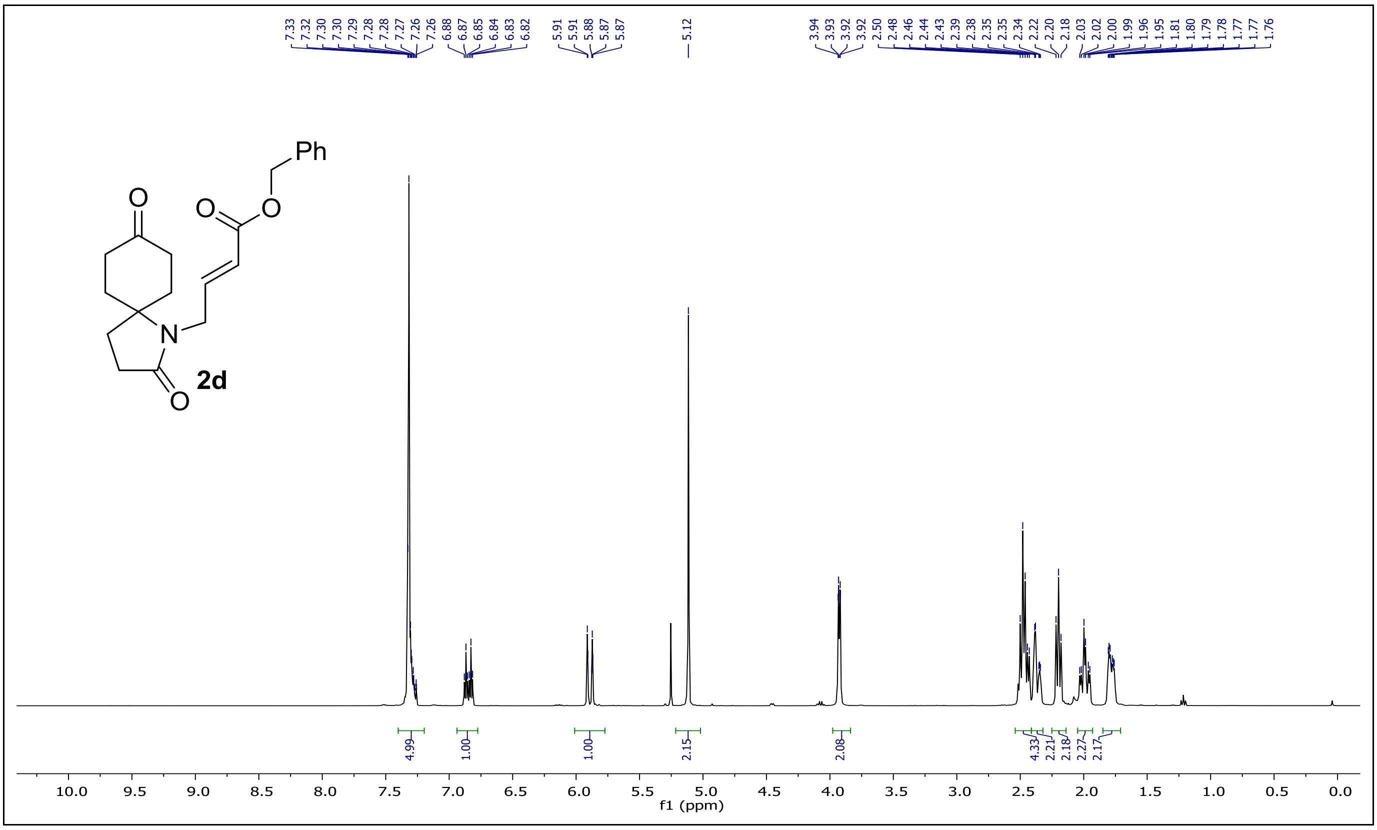

^{13}C NMR Spectrum of compound 2d

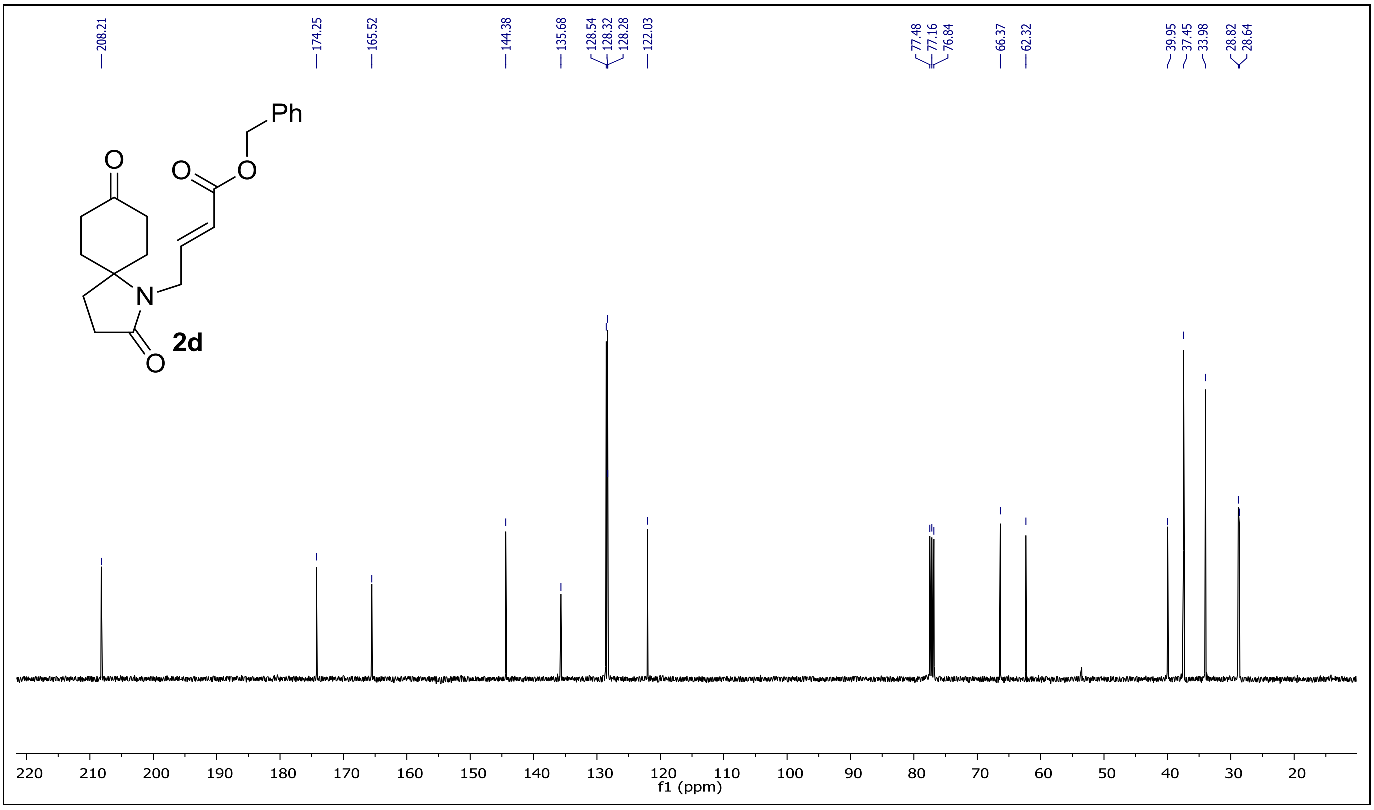

^{1}H NMR Spectrum of compound 7

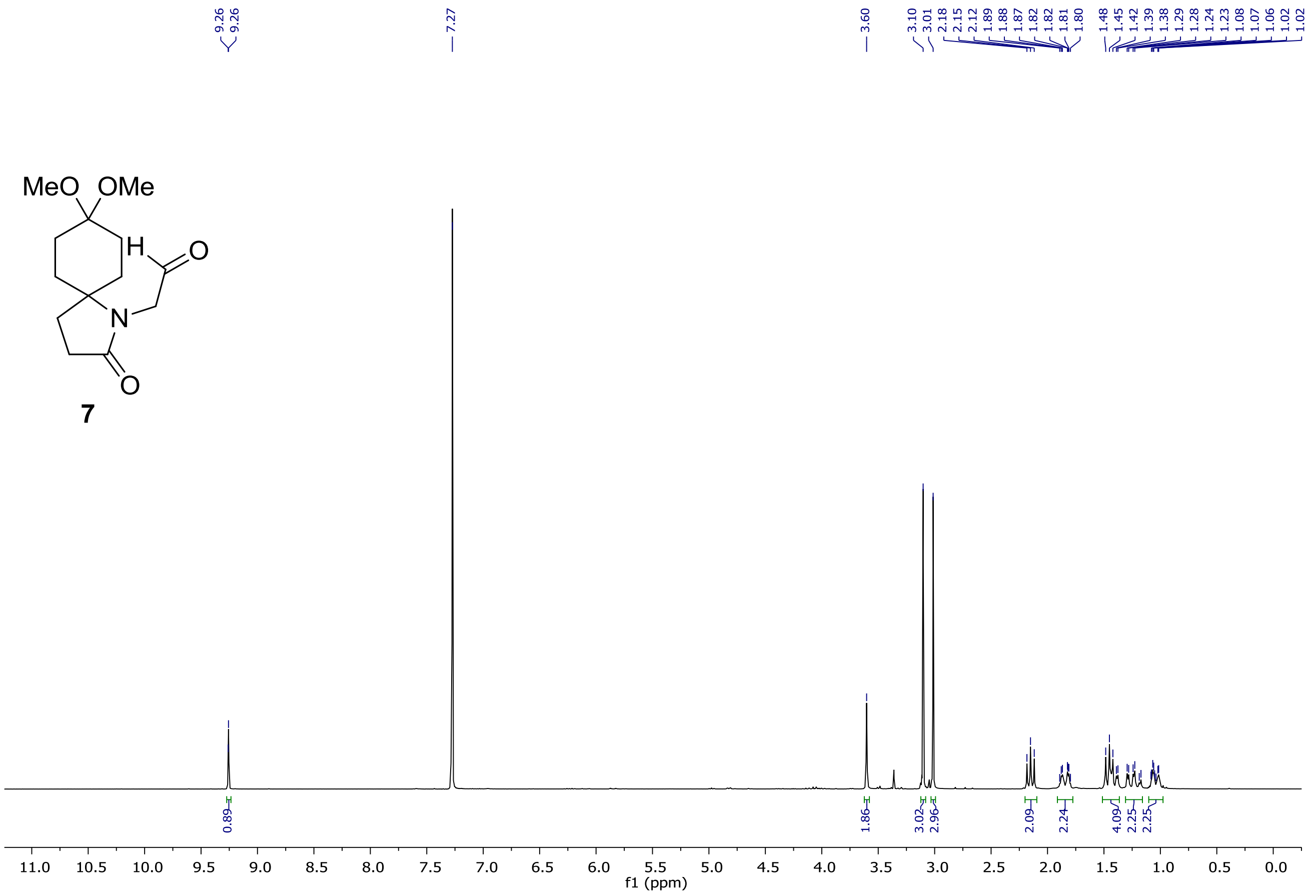

^{13}C NMR Spectrum of compound 7

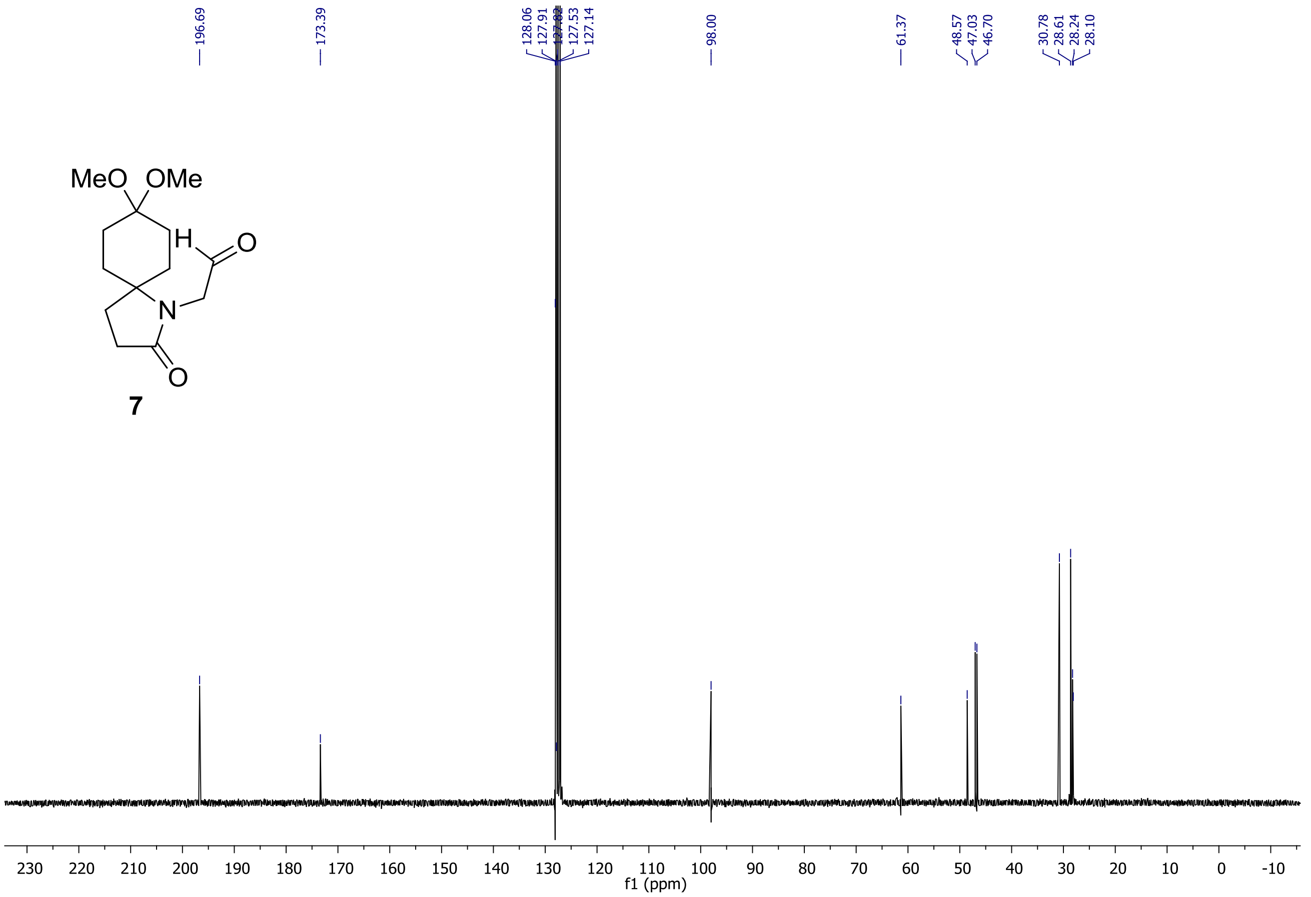

^{1}H NMR Spectrum of compound 8

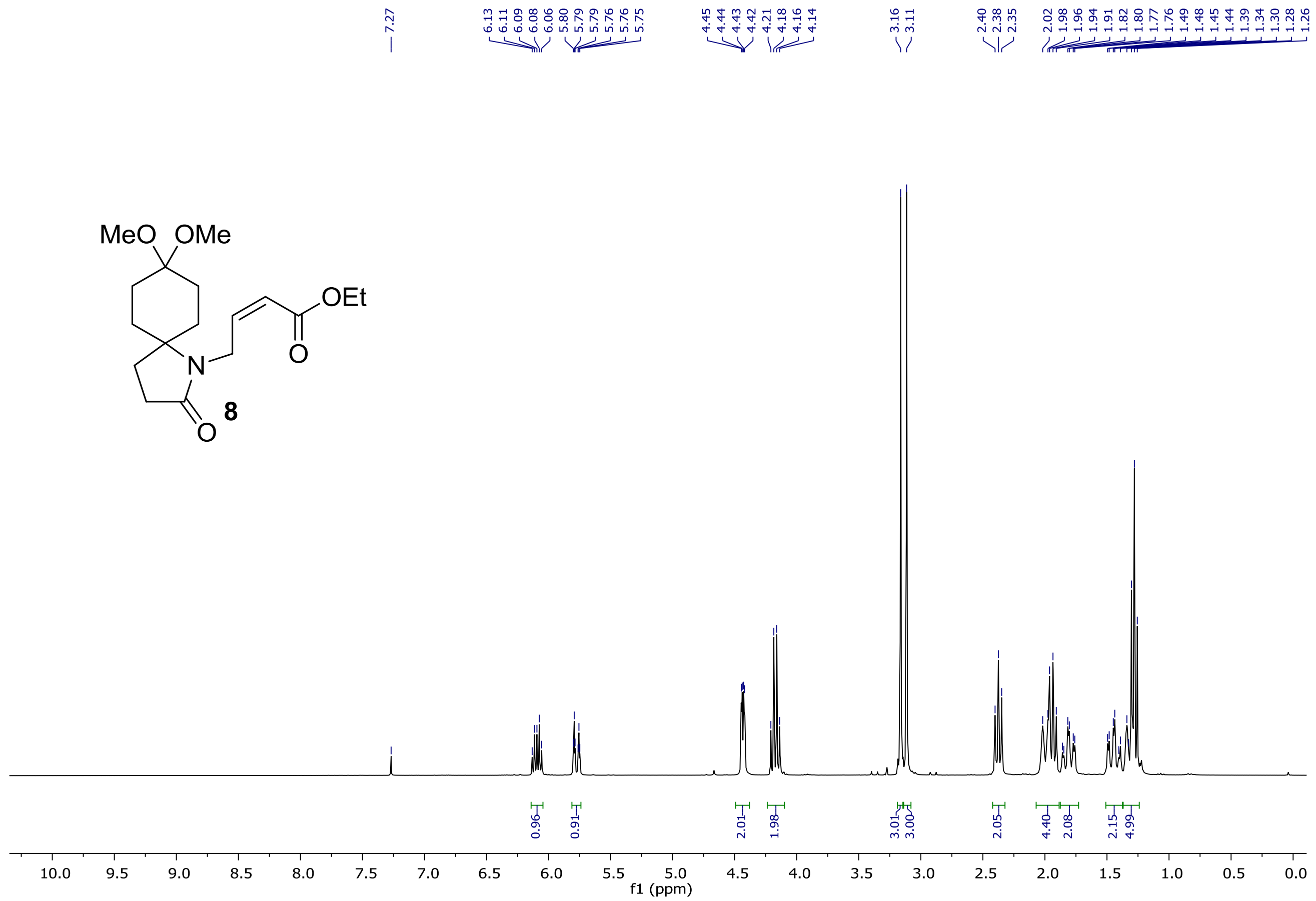

^{13}C NMR Spectrum of compound 8

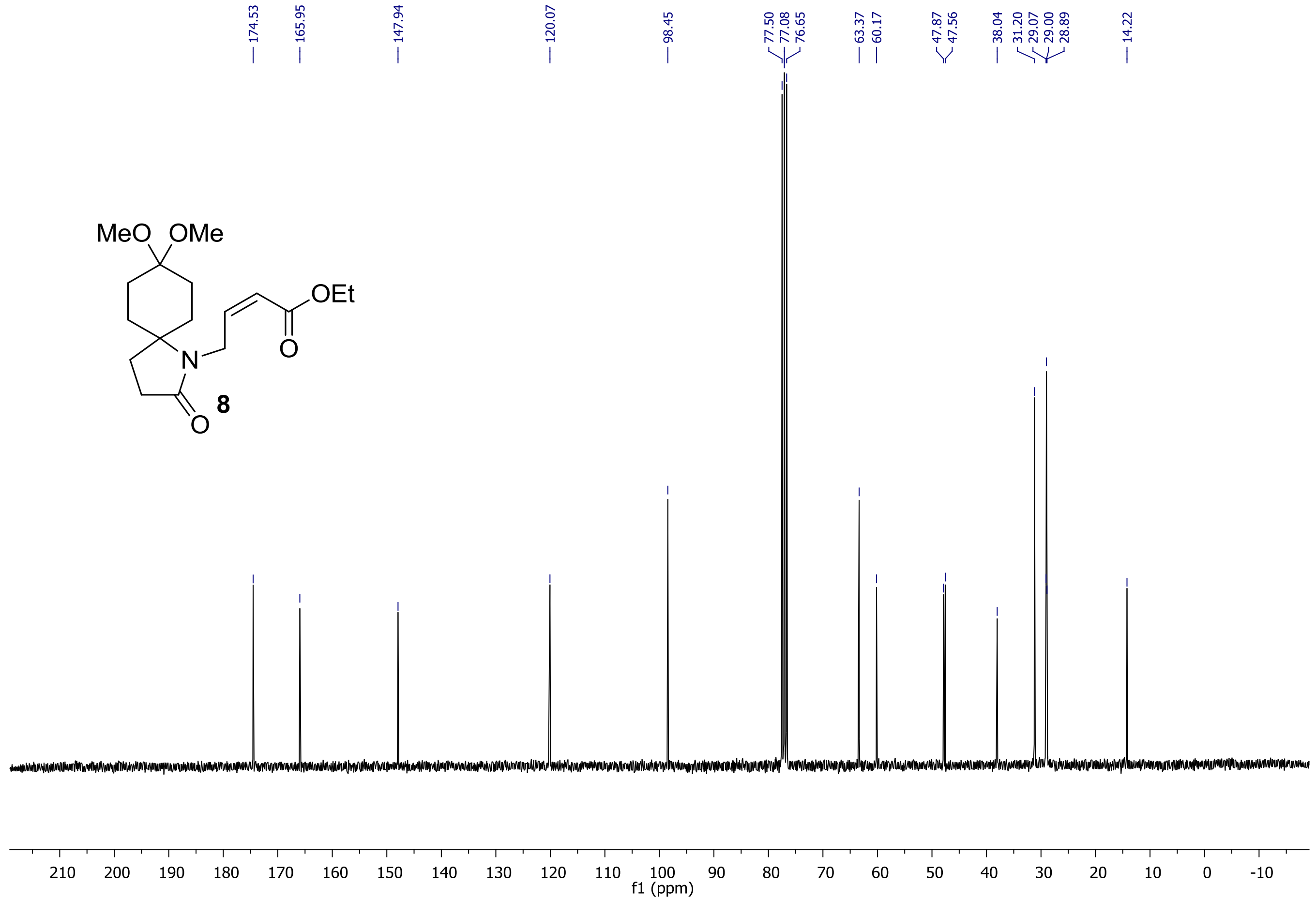

^{1}H NMR Spectrum of compound *Z*-2b

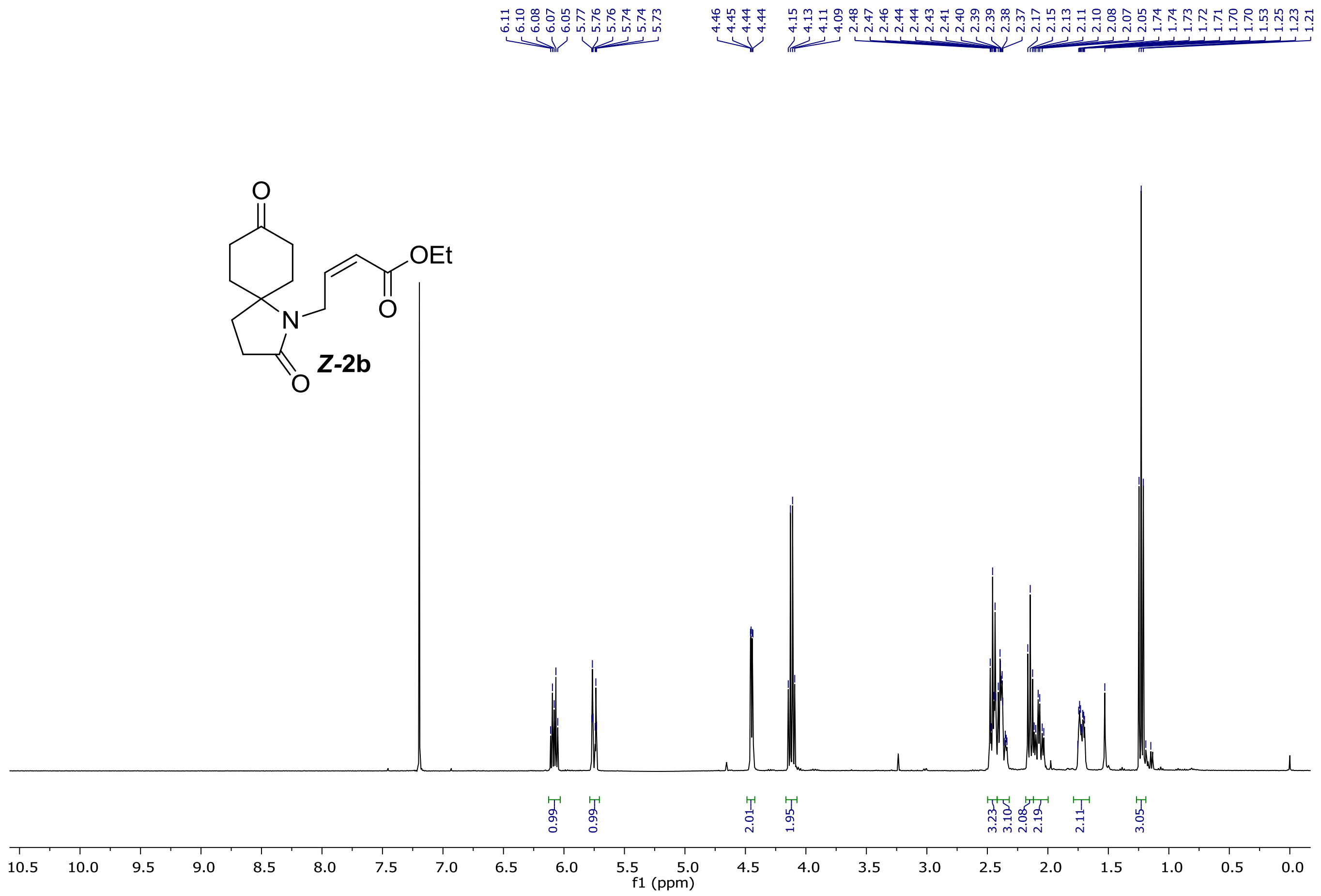

^{13}C NMR Spectrum of compound *Z*-2b

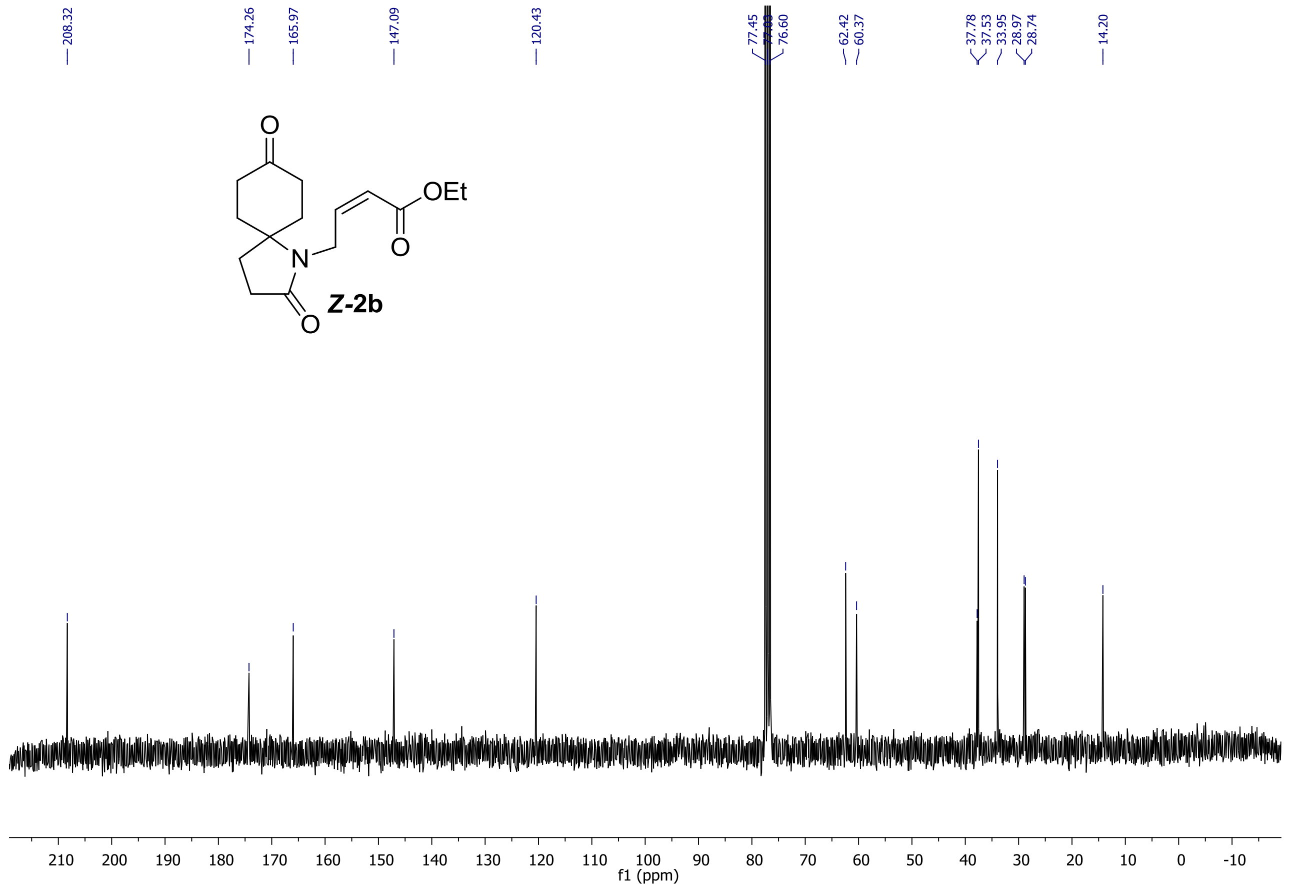

^{1}H NMR Spectrum of compound 10b

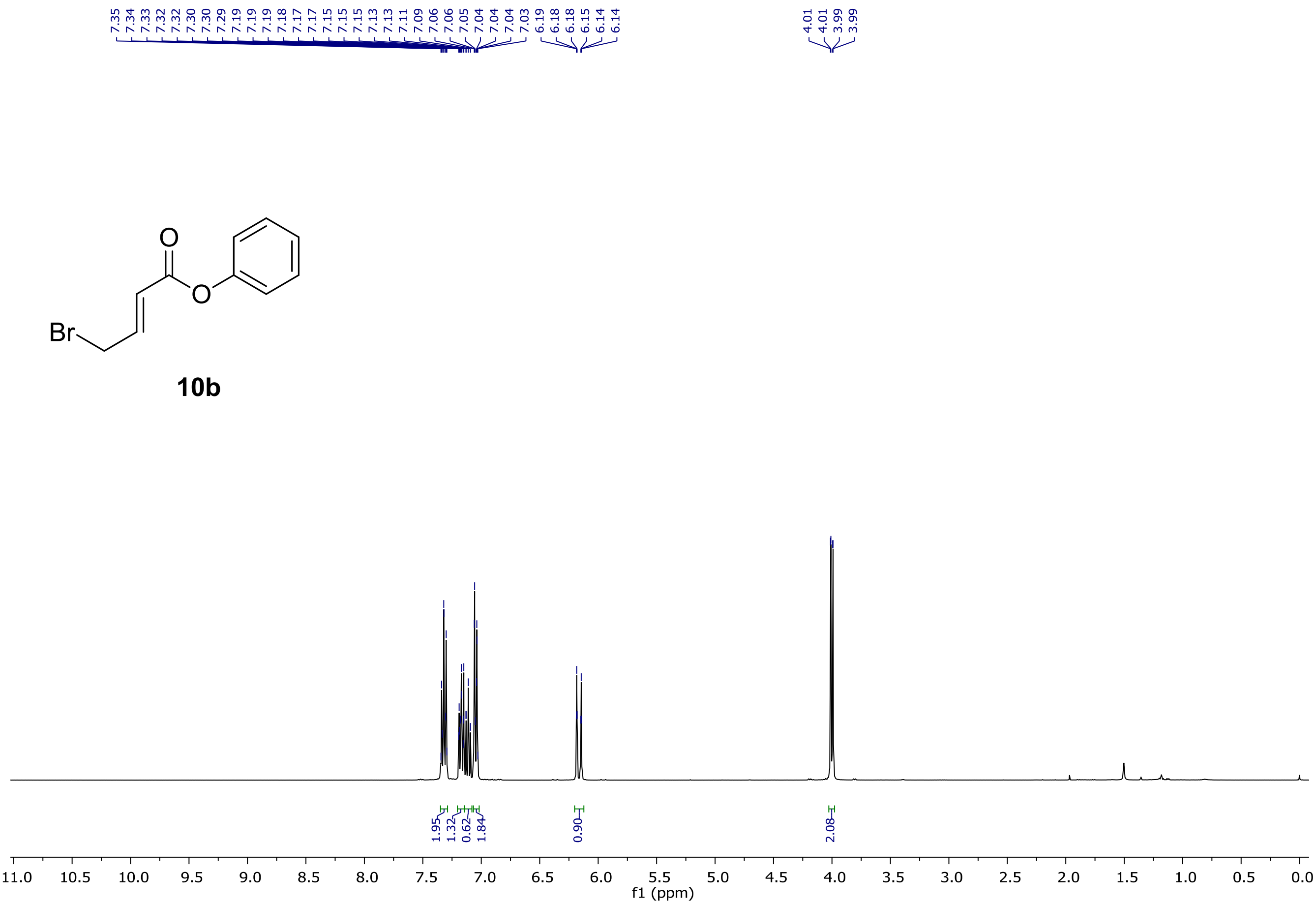

10b

^{13}C NMR Spectrum of compound 10b

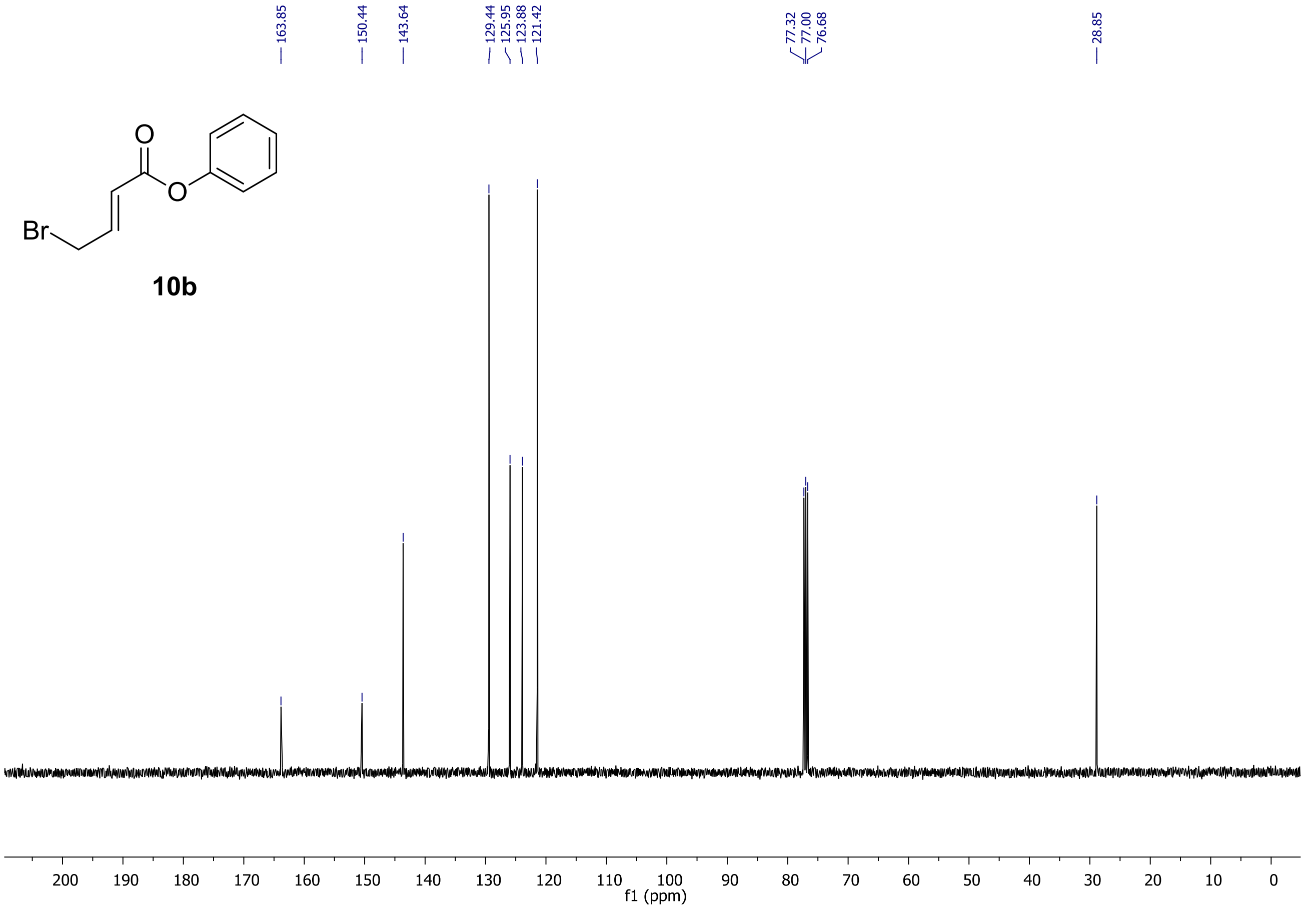

10b

^{1}H NMR Spectrum of compound 10d

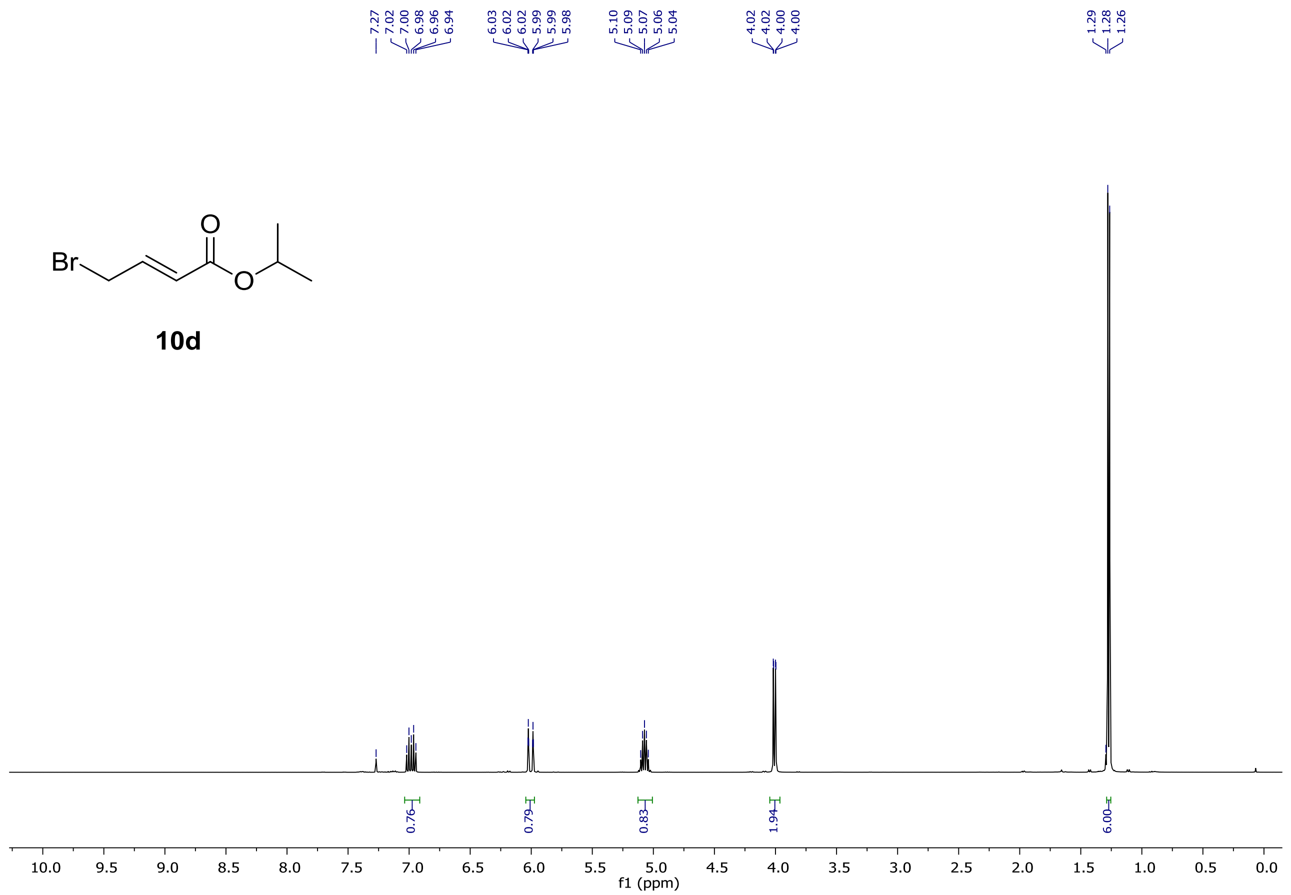

^{13}C NMR Spectrum of compound 10d

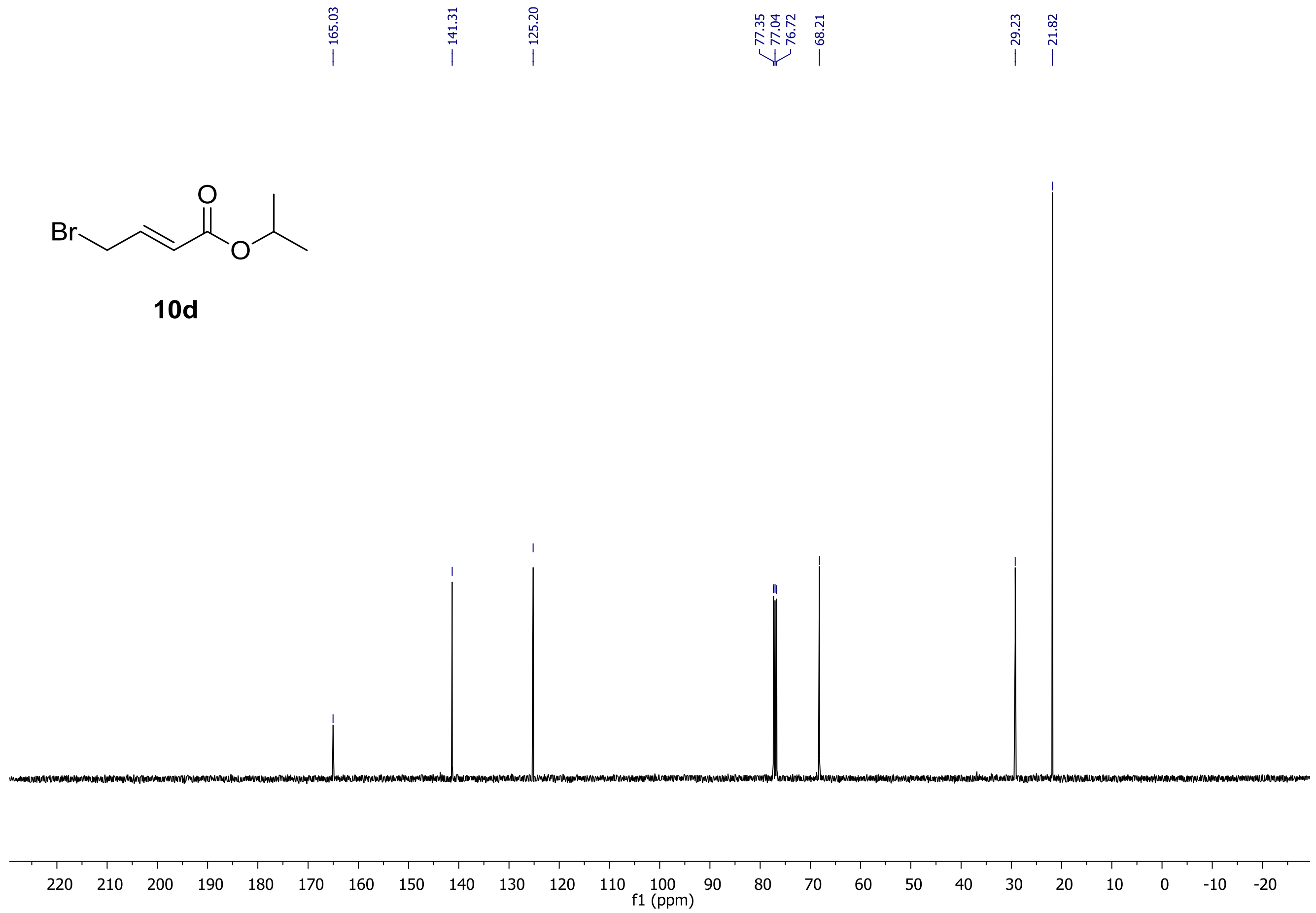

^{1}H NMR Spectrum of compound 12

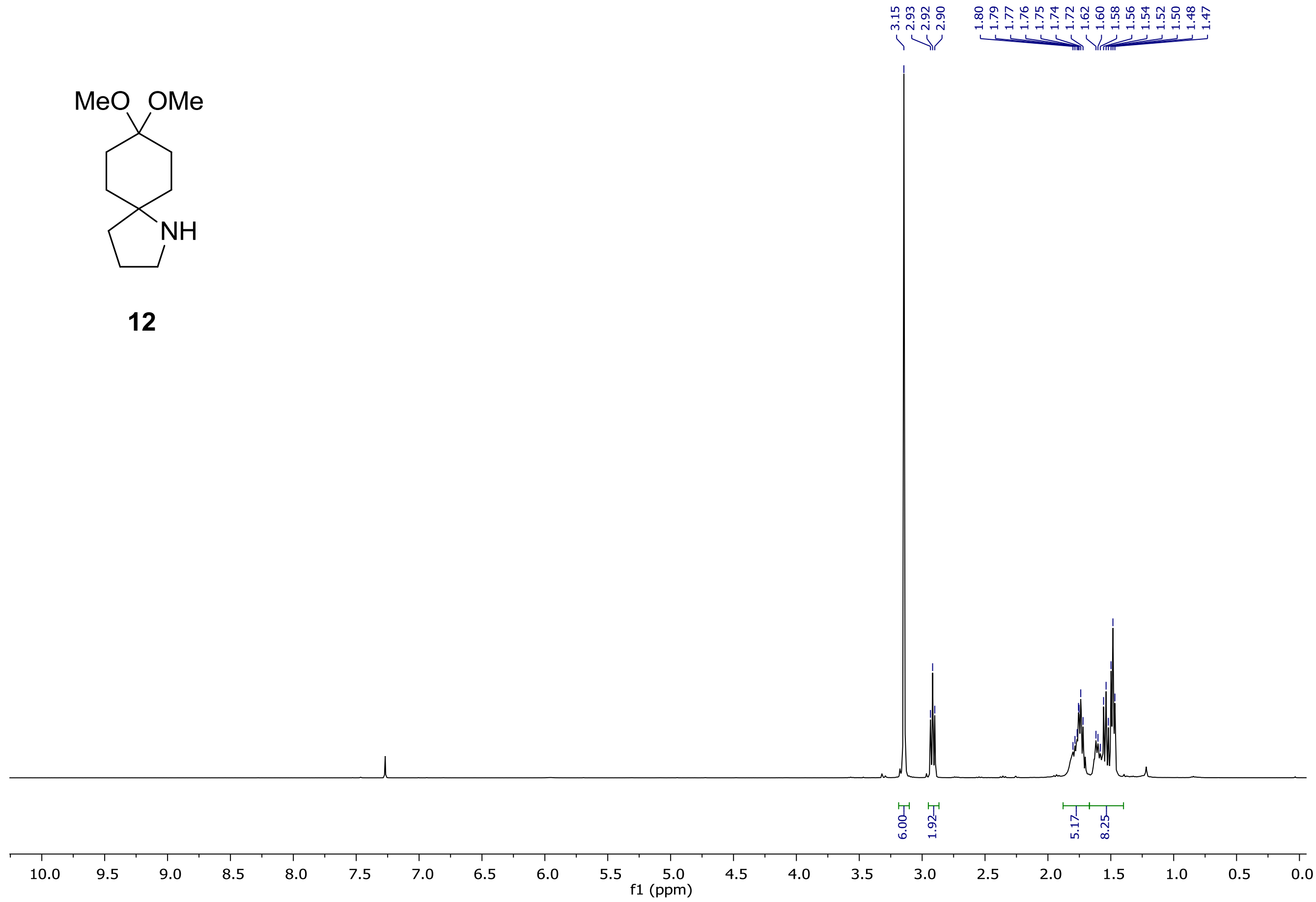

^{13}C NMR Spectrum of compound 12

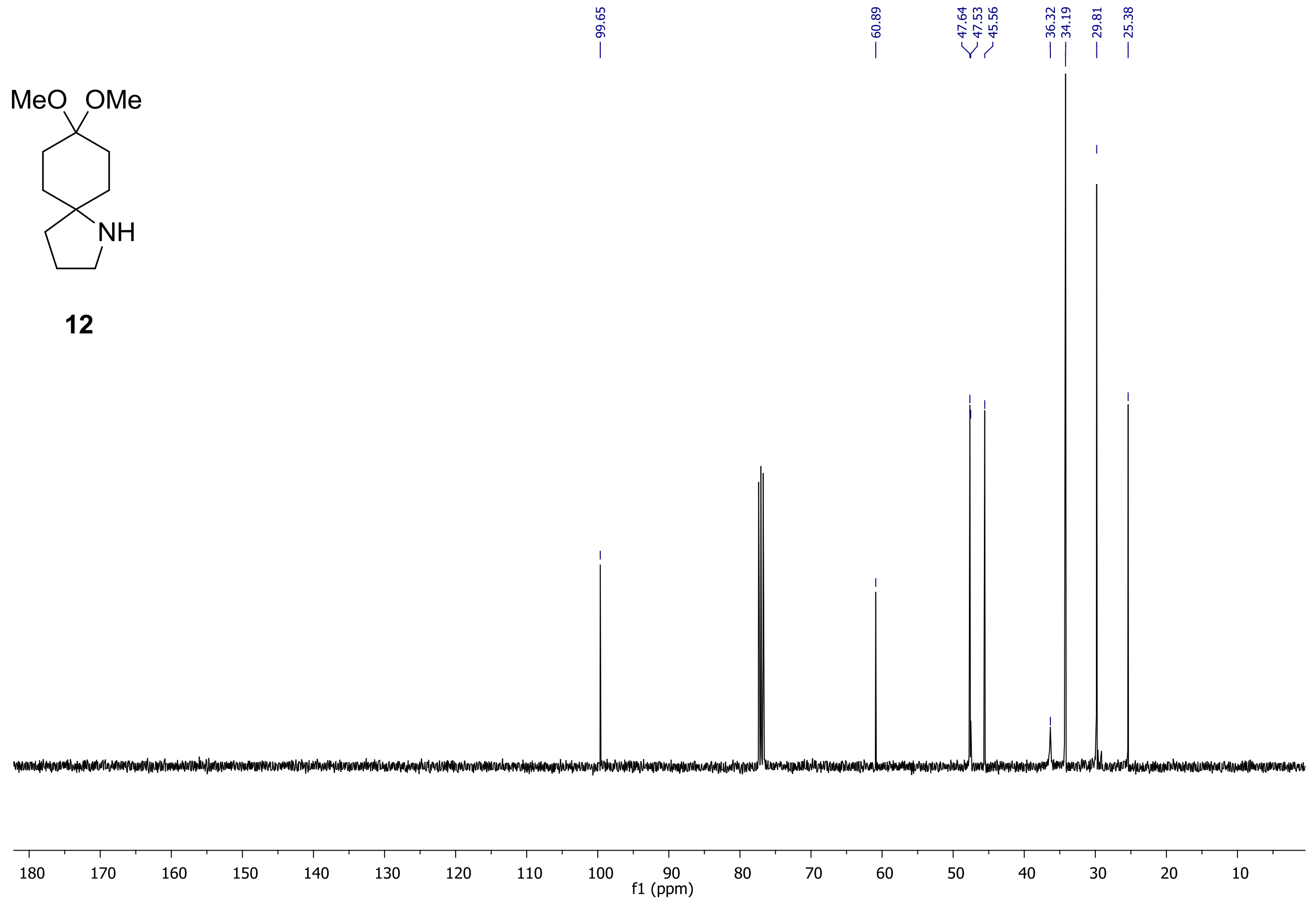

^{1}H NMR Spectrum of compound 13e

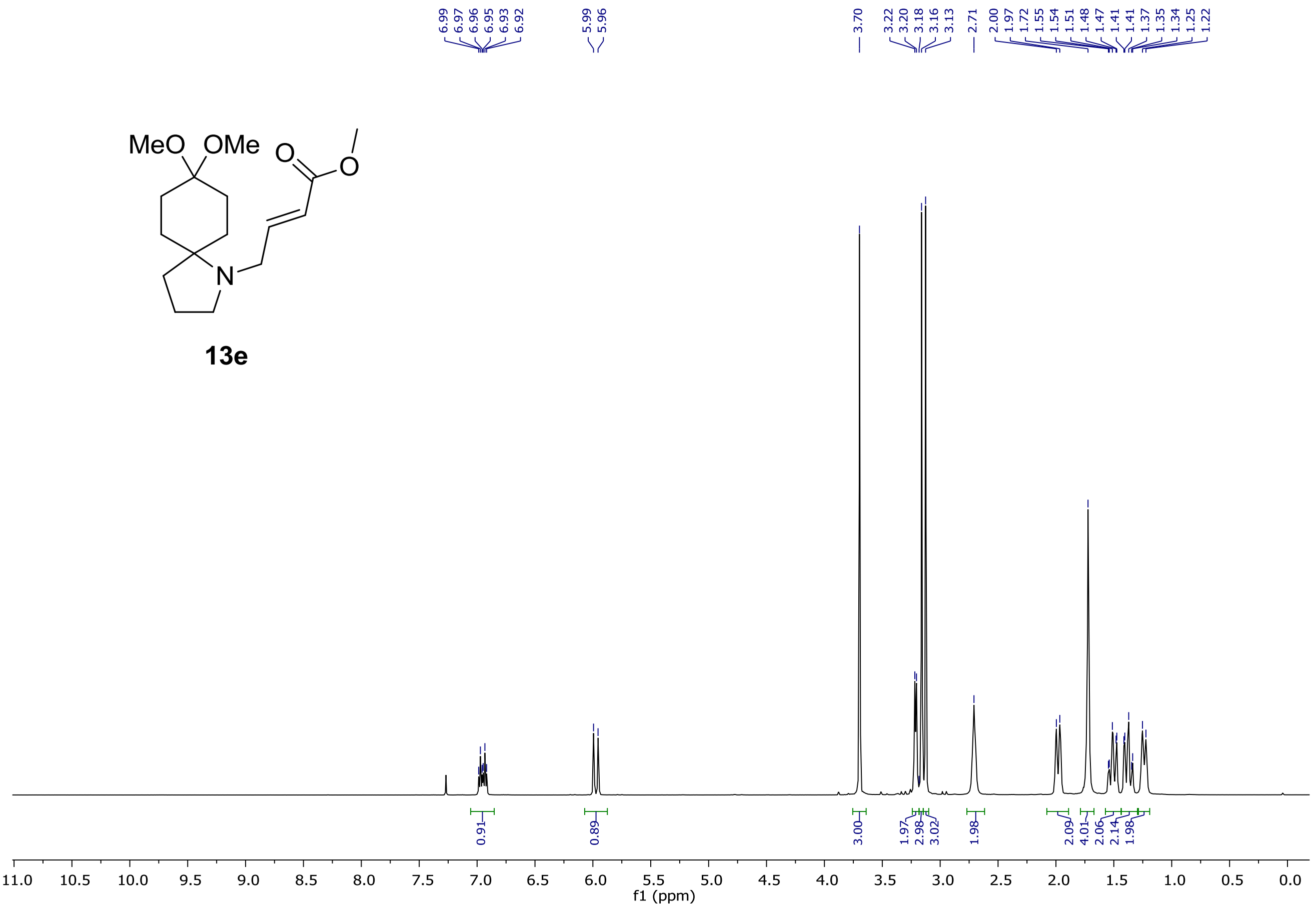

^{13}C NMR Spectrum of compound 13e

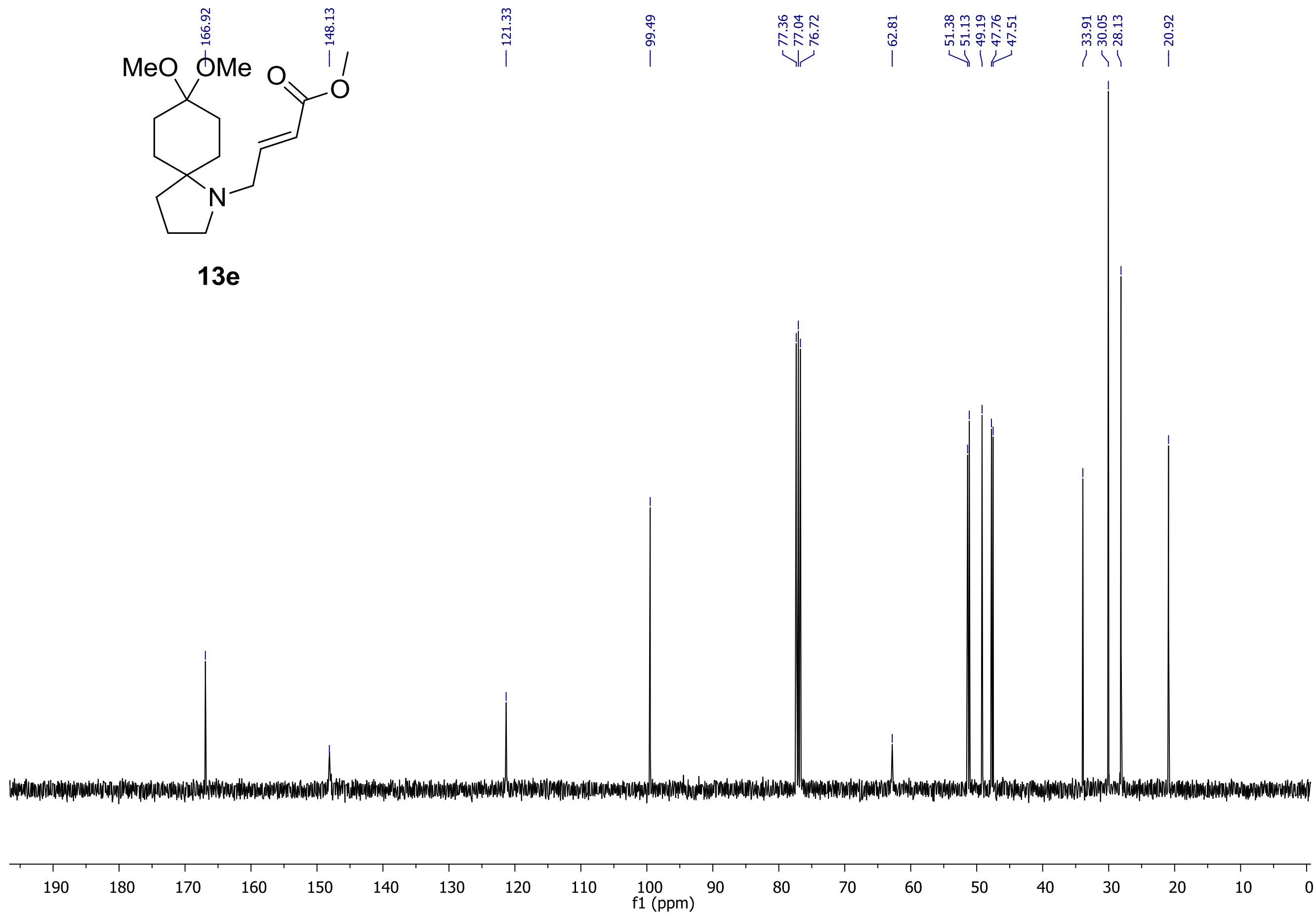

^{1}H NMR Spectrum of compound 2e

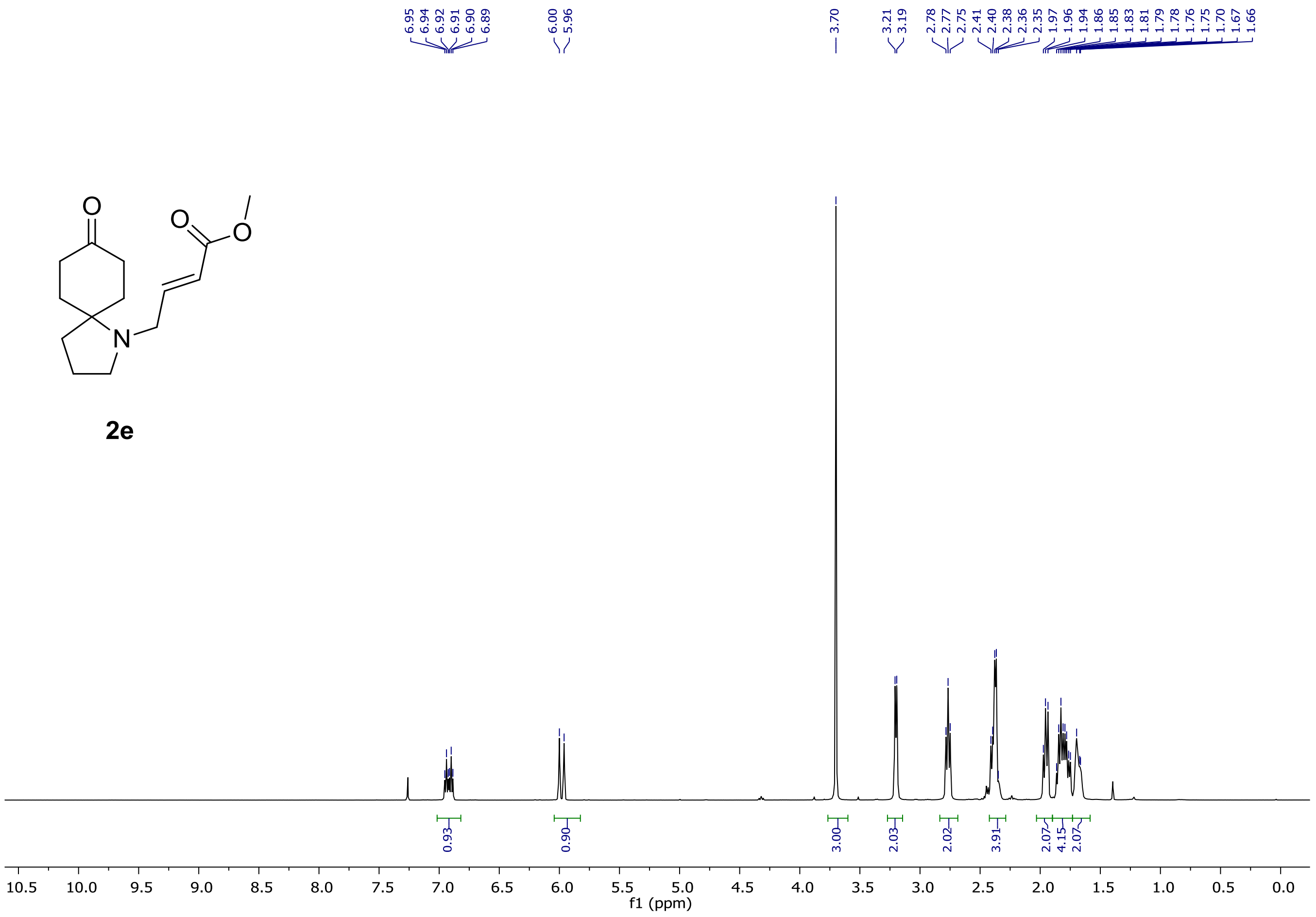

^{13}C NMR Spectrum of compound 2e

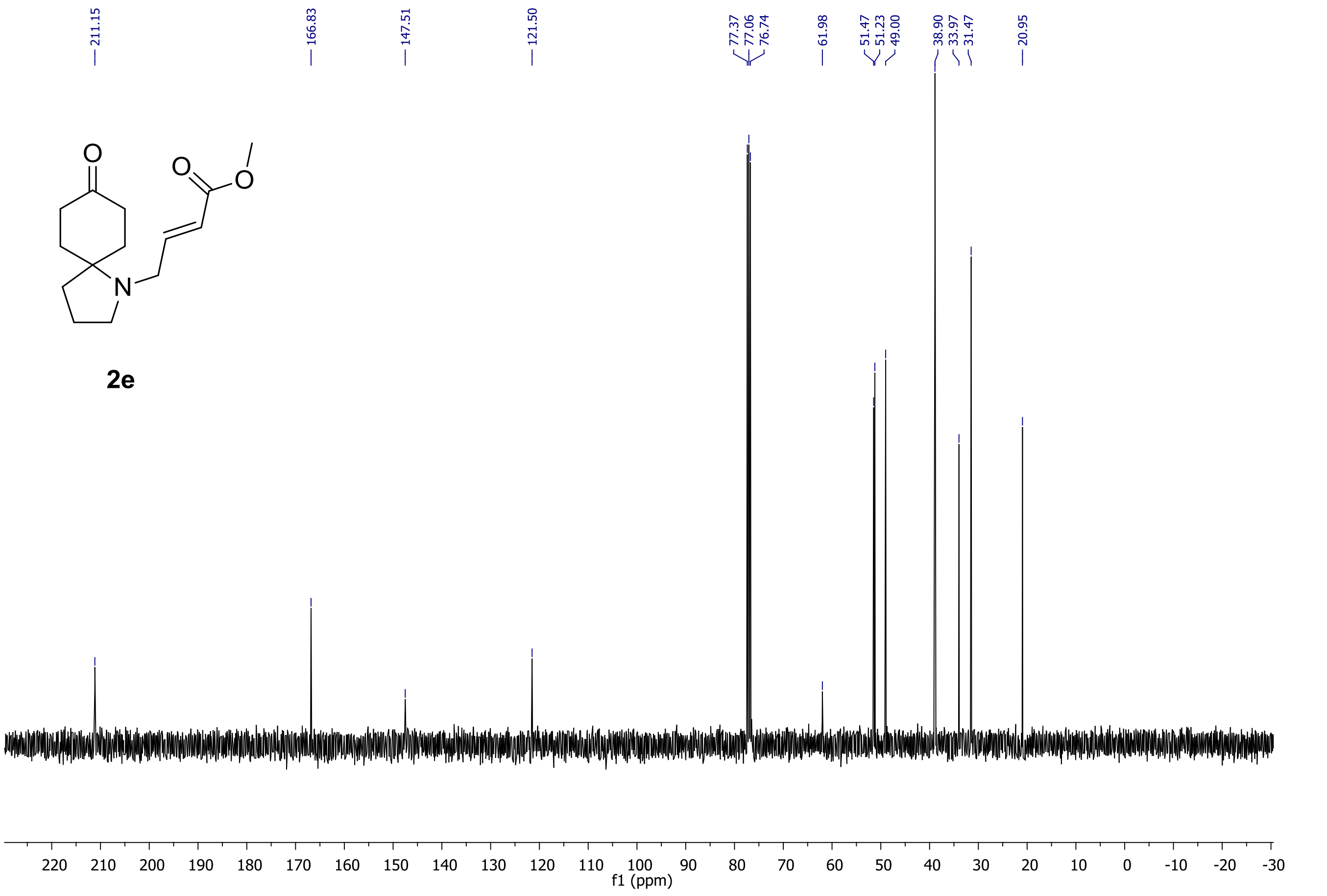

¹H NMR Spectrum of compound 13f

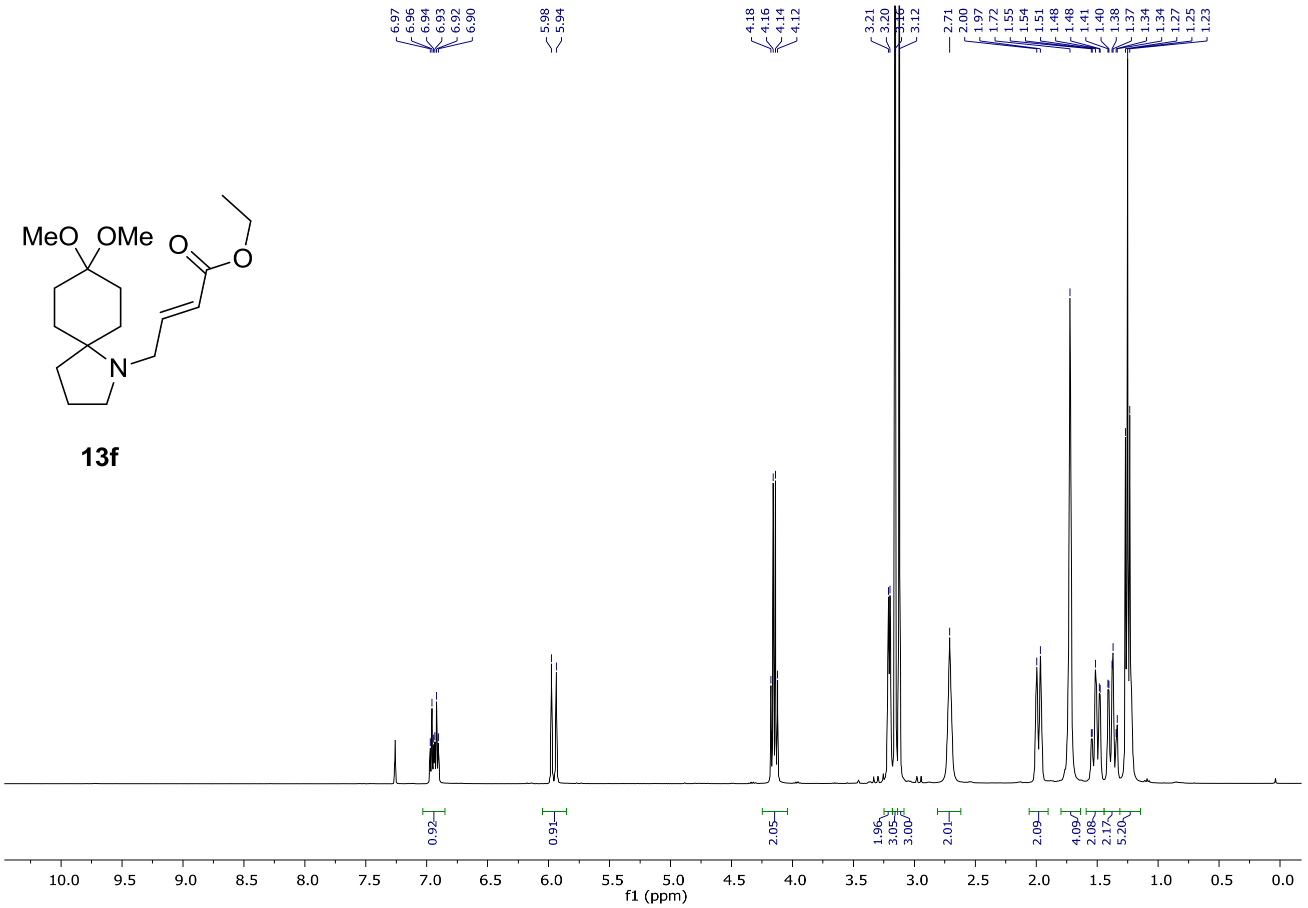

¹³C NMR Spectrum of compound 13f

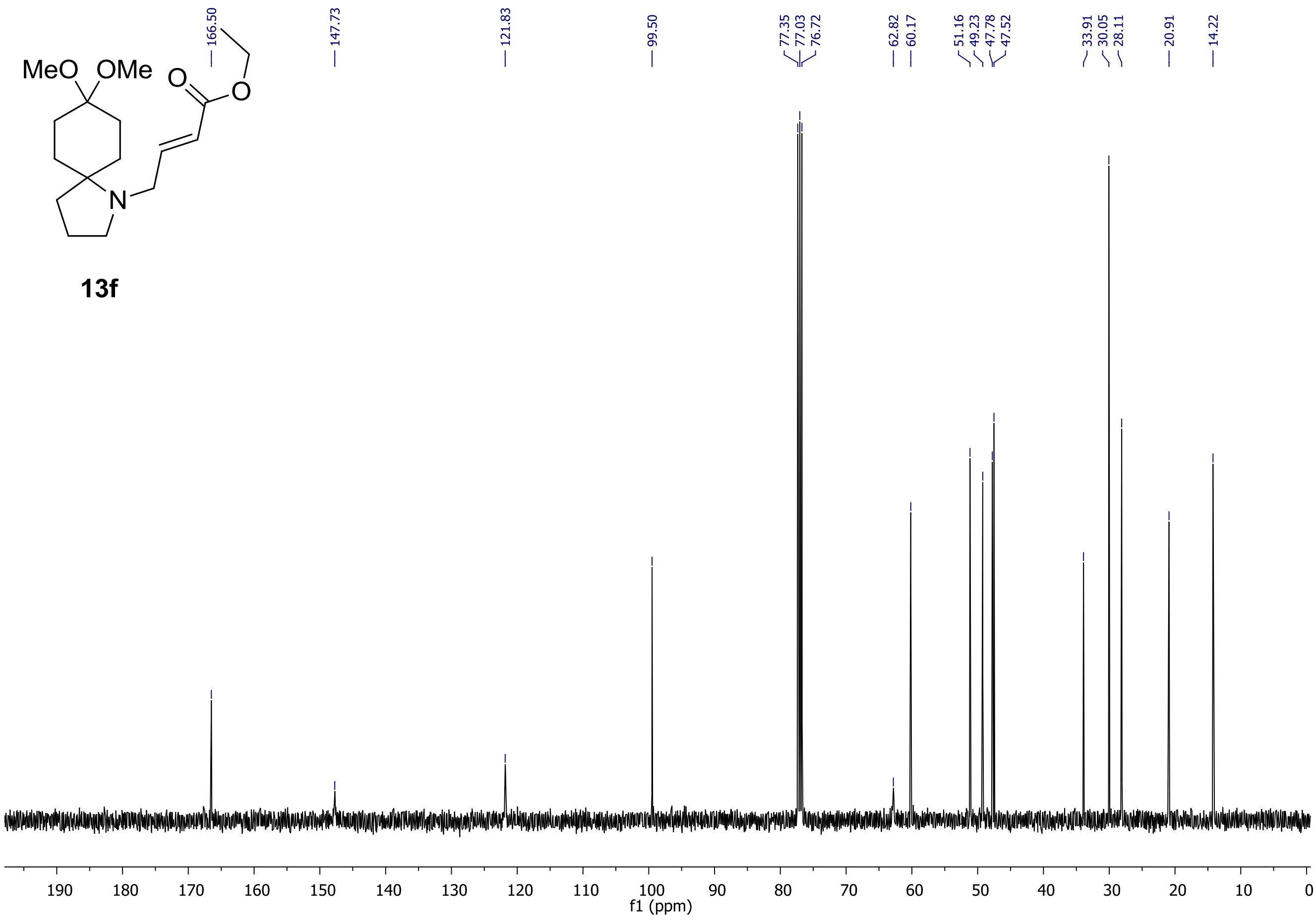

^{1}H NMR Spectrum of compound 2f

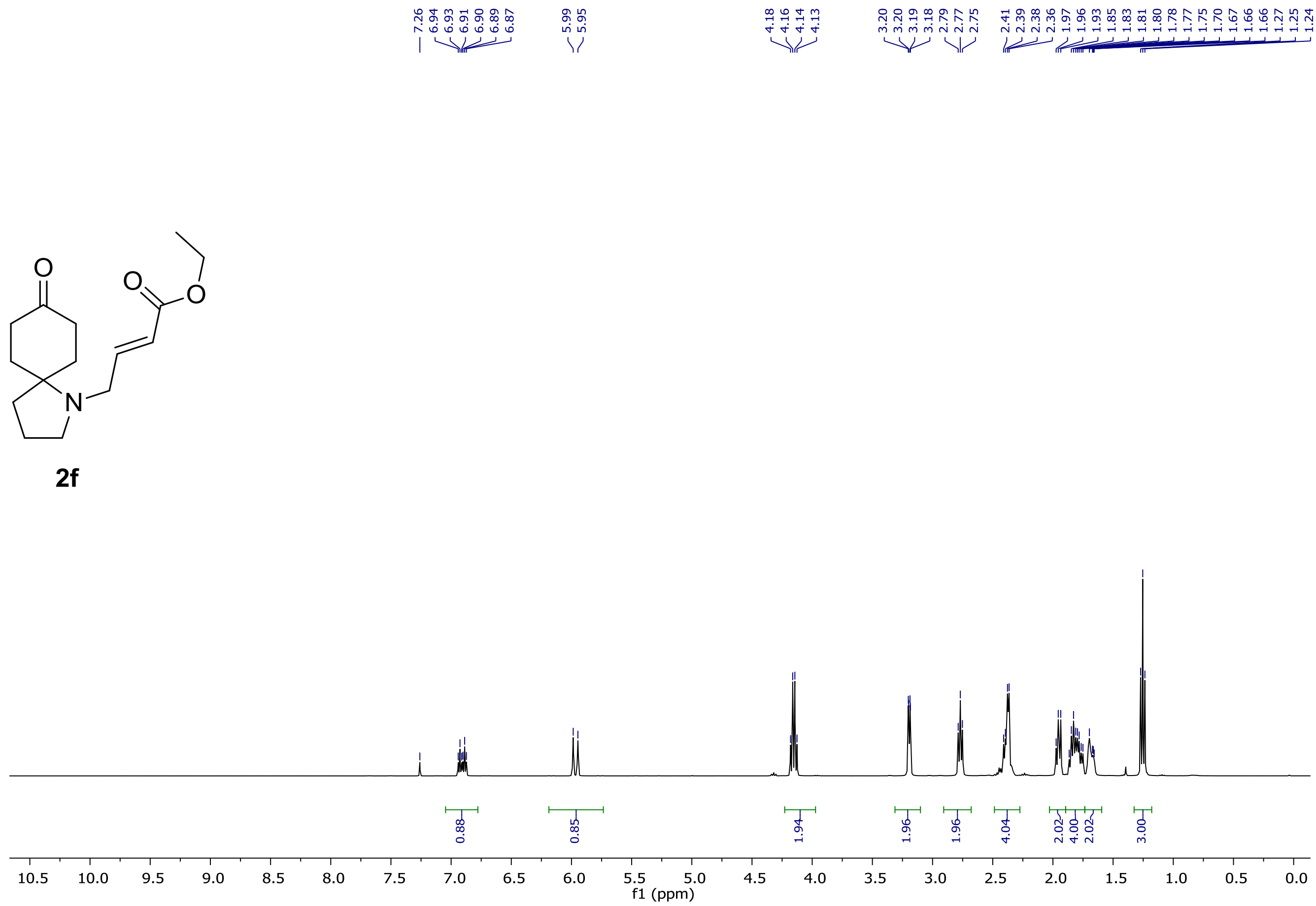

^{13}C NMR Spectrum of compound 2f

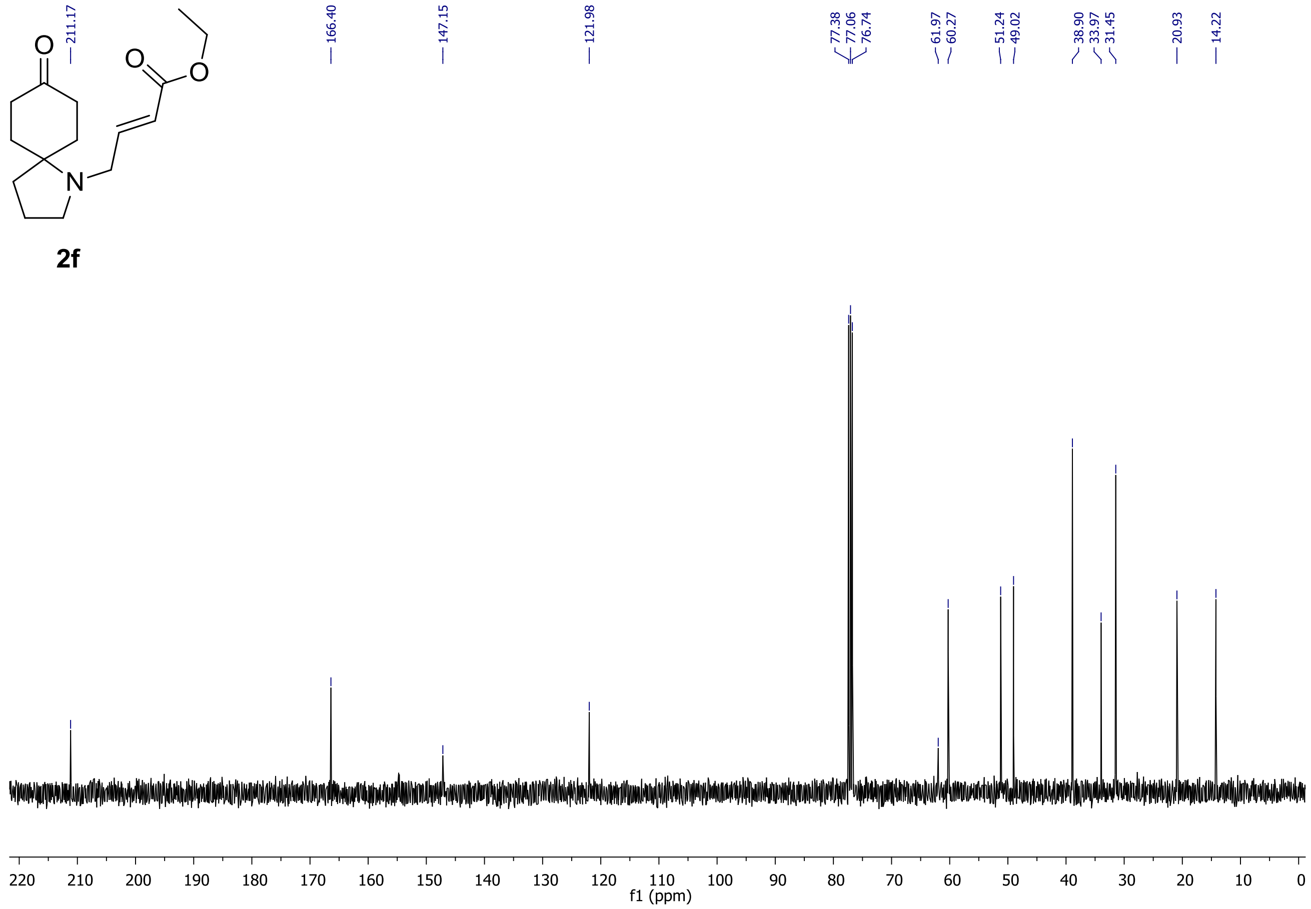

^{1}H NMR Spectrum of compound 13g

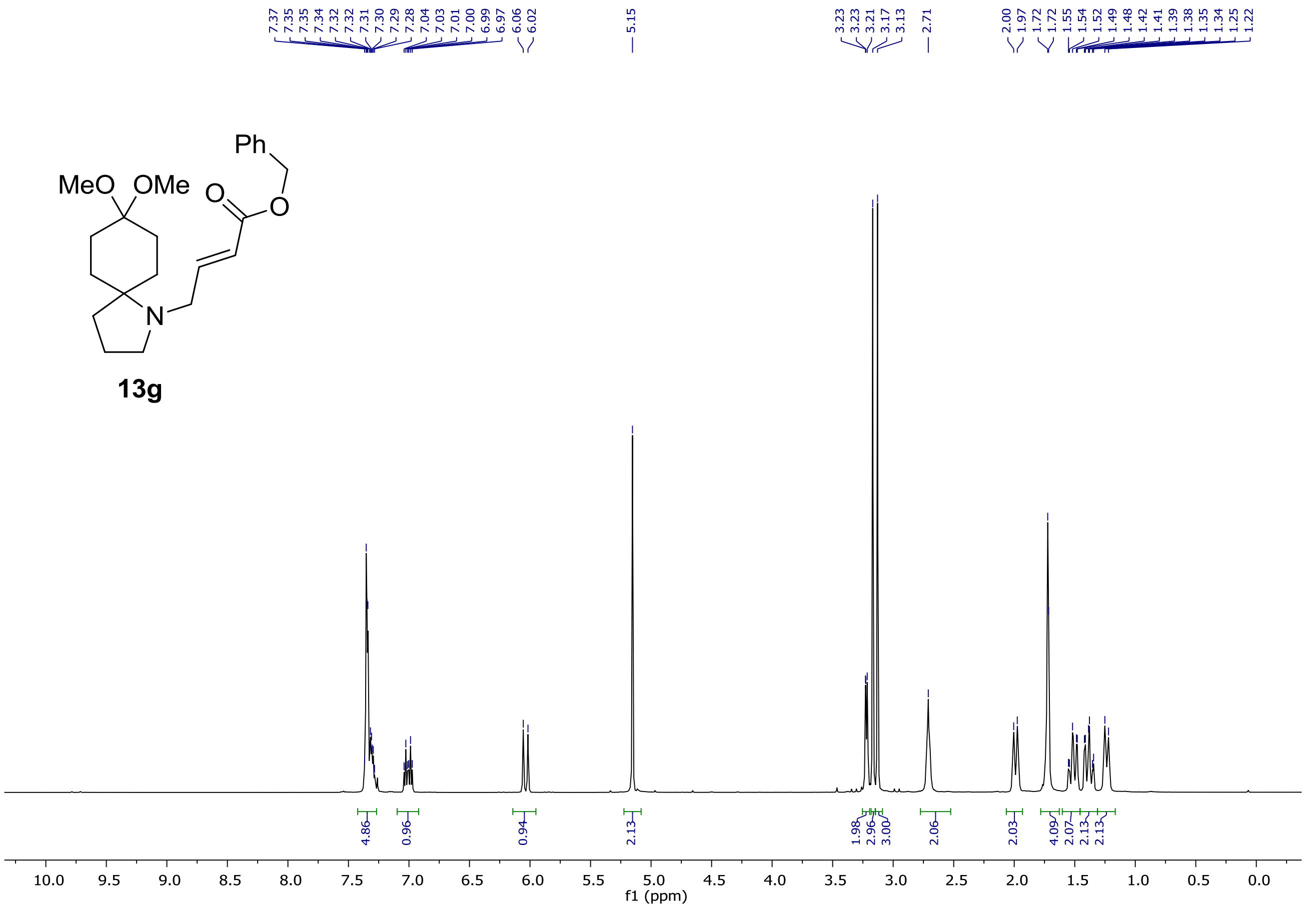

^{13}C NMR Spectrum of compound 13g

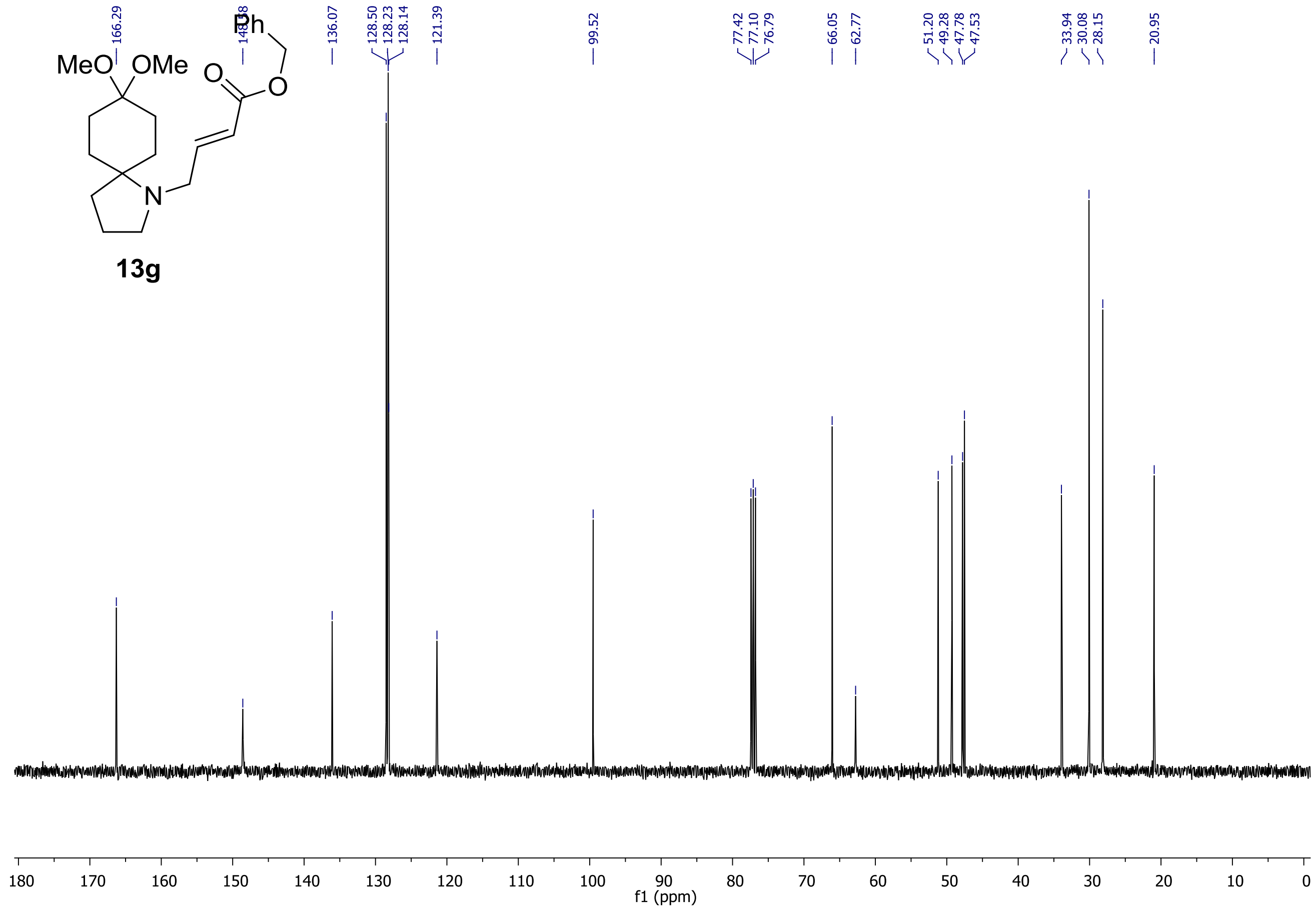

^{1}H NMR Spectrum of compound 2g

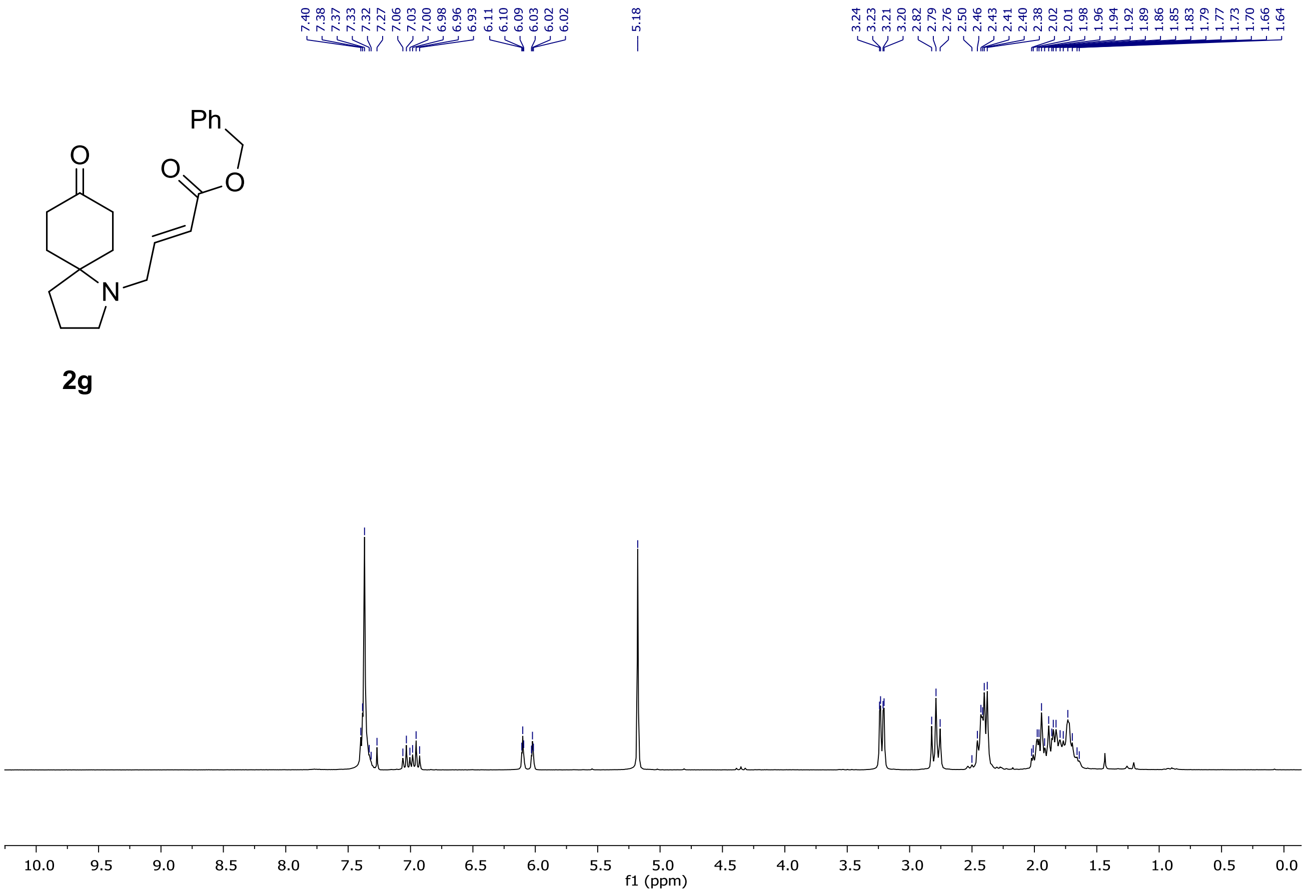

^{13}C NMR Spectrum of compound 2g

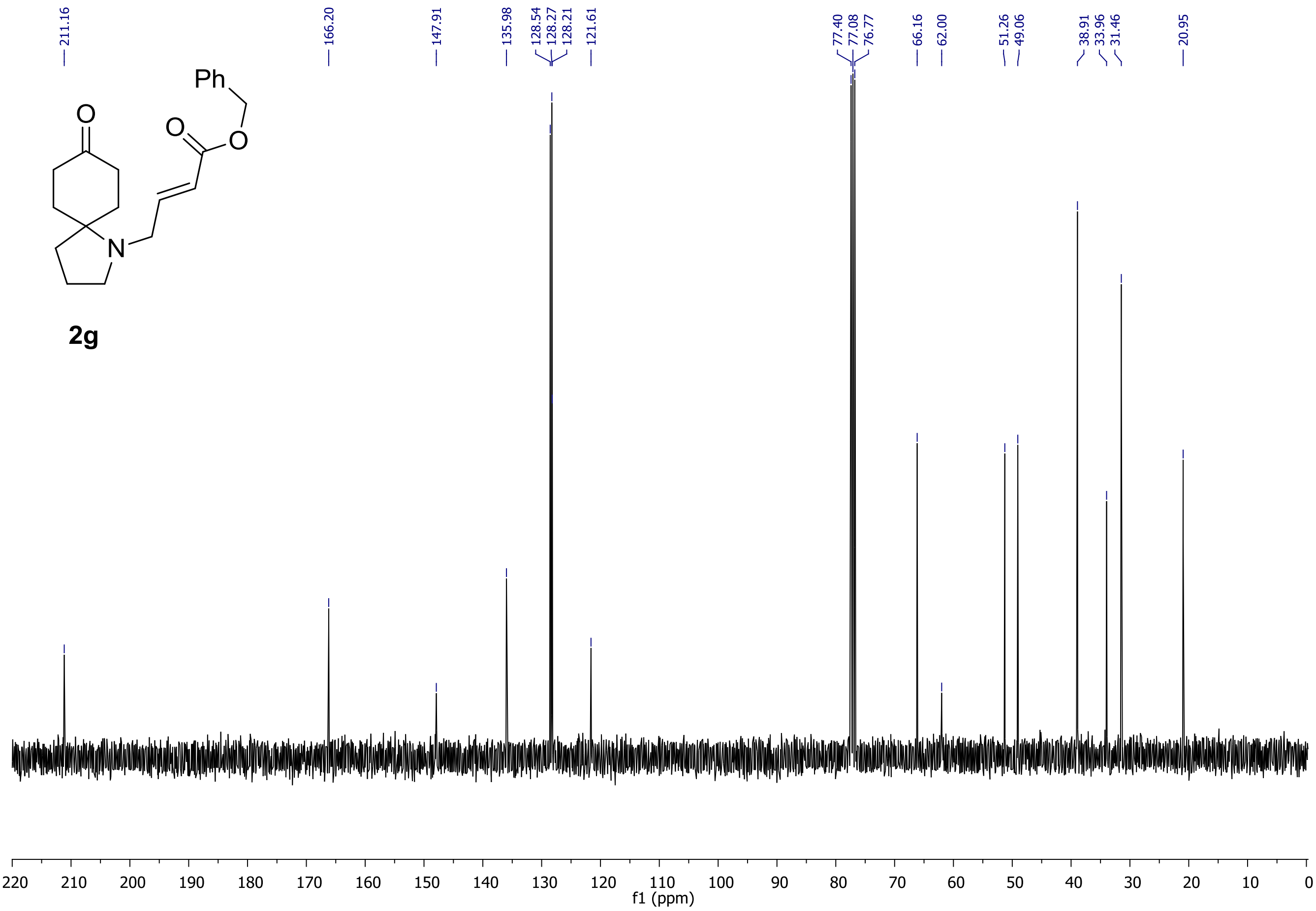

^{1}H NMR Spectrum of compound 13h

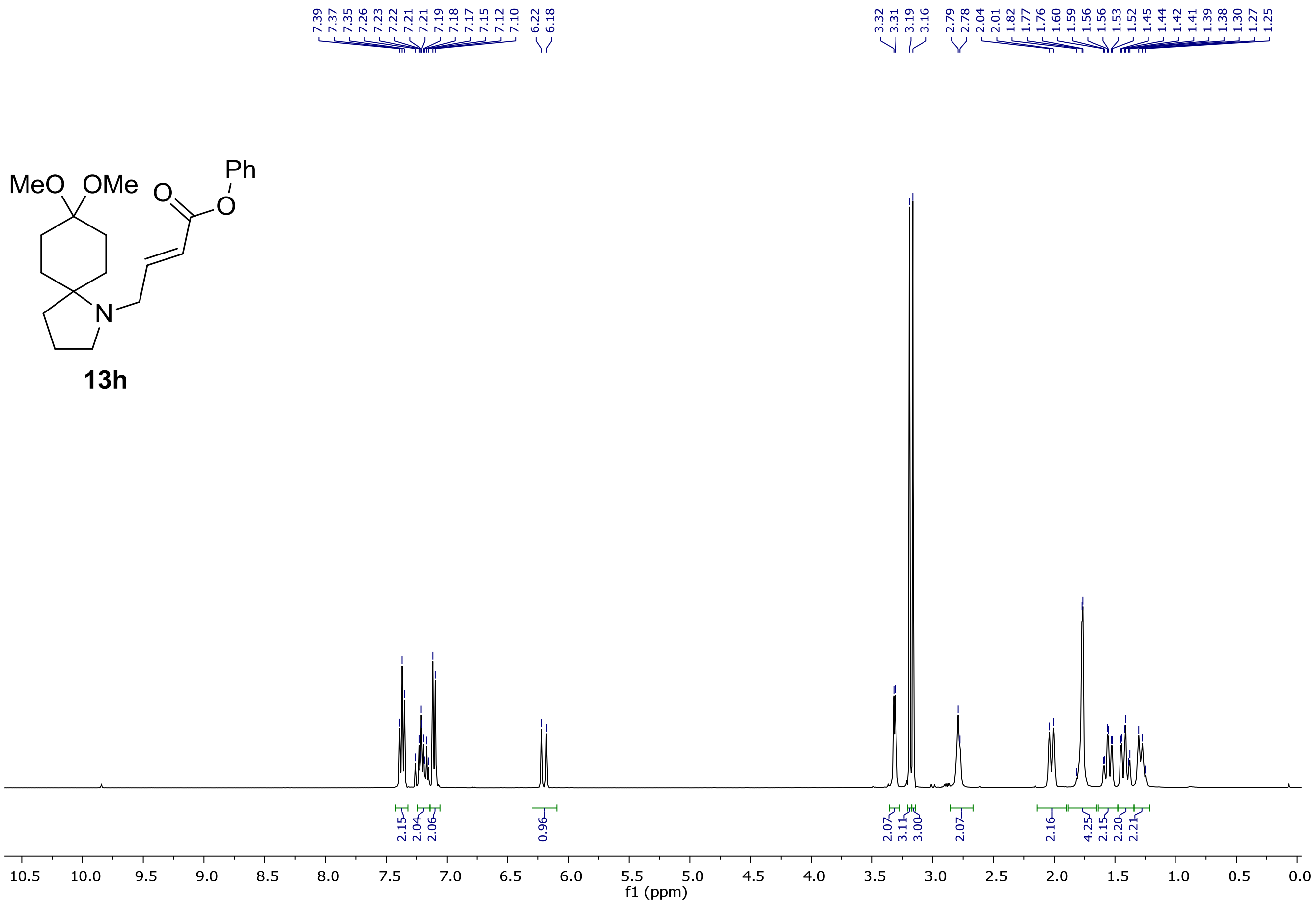

^{13}C NMR Spectrum of compound 13h

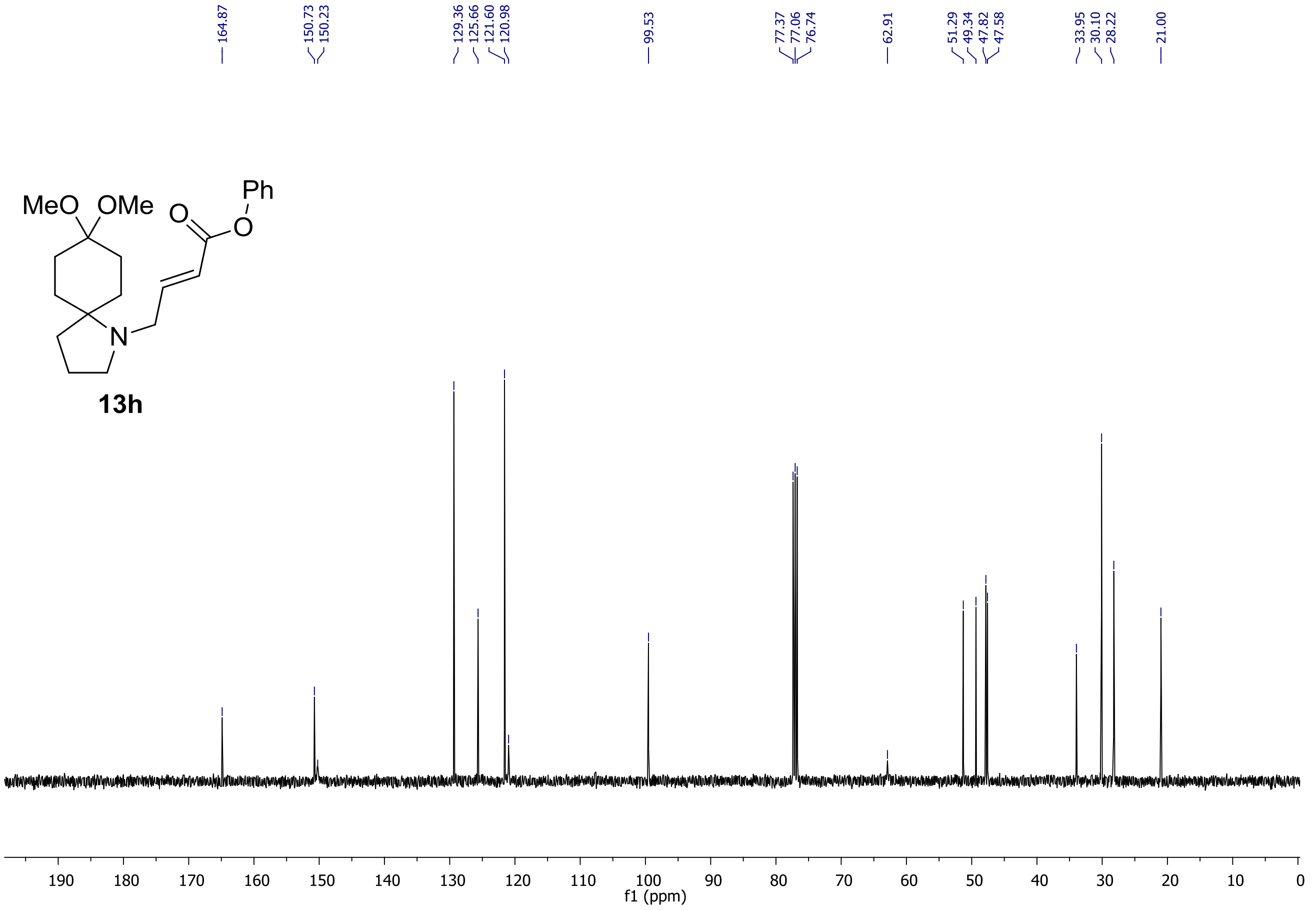

^{1}H NMR Spectrum of compound 2h

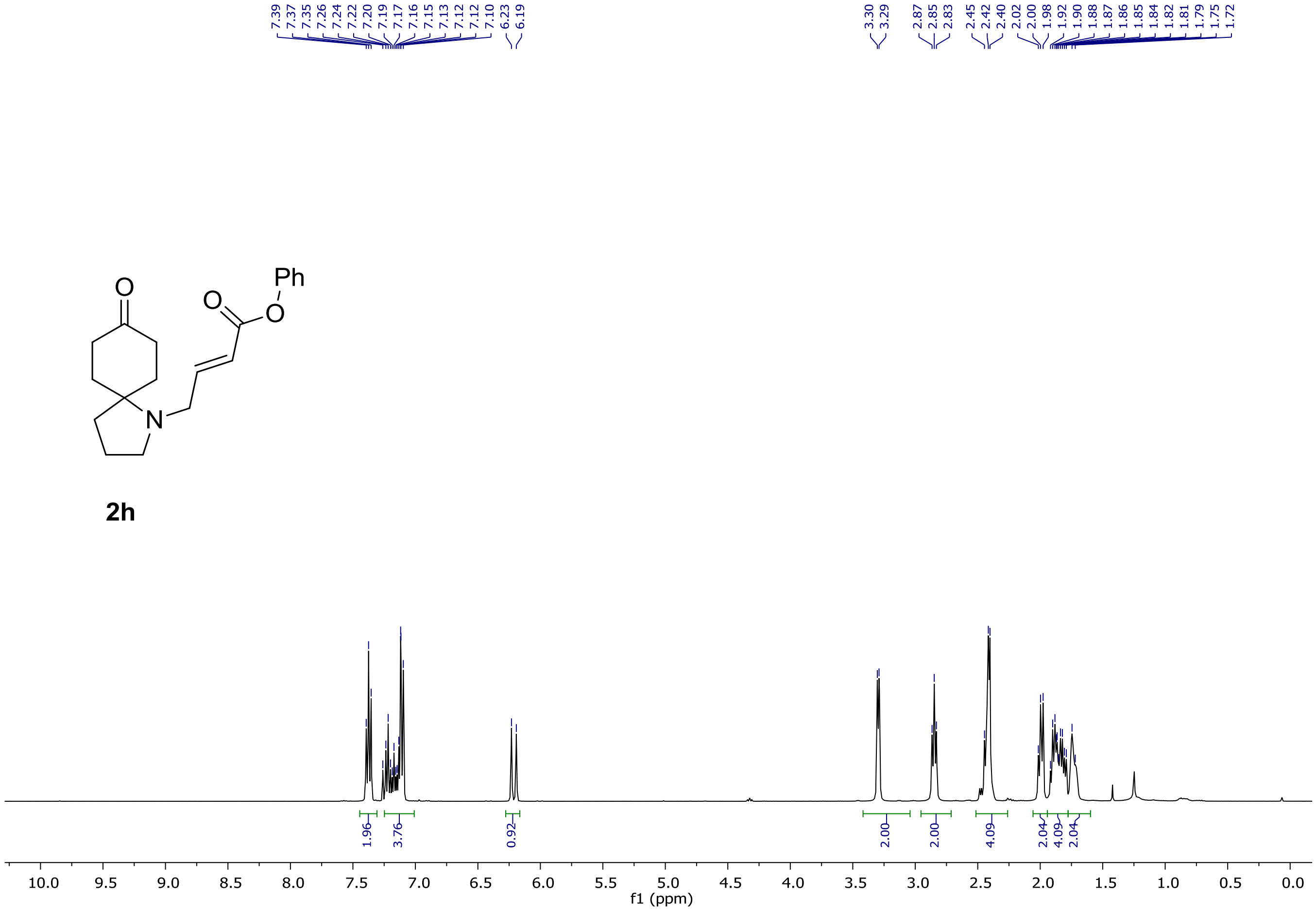

^{13}C NMR Spectrum of compound 2h

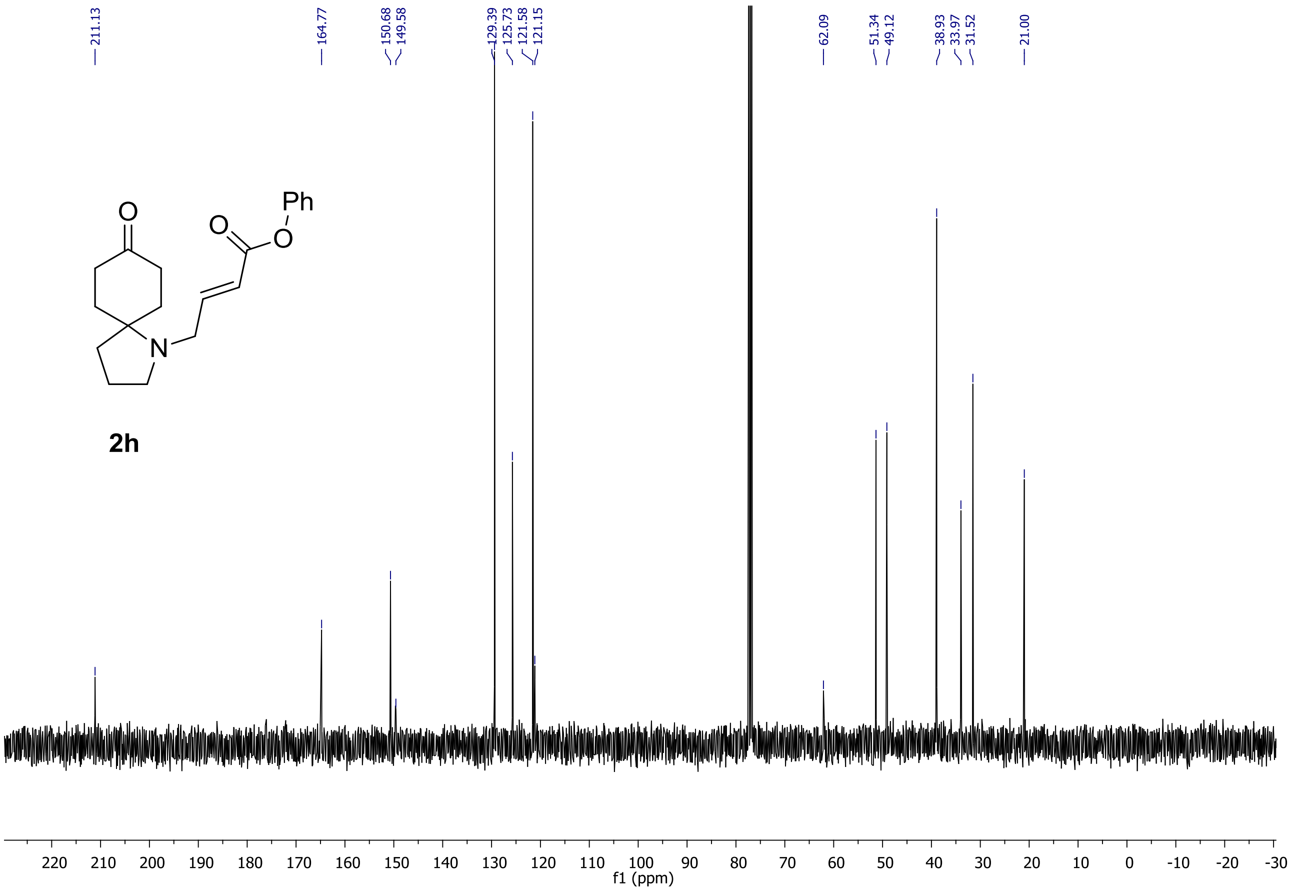

^{1}H NMR Spectrum of compound 15

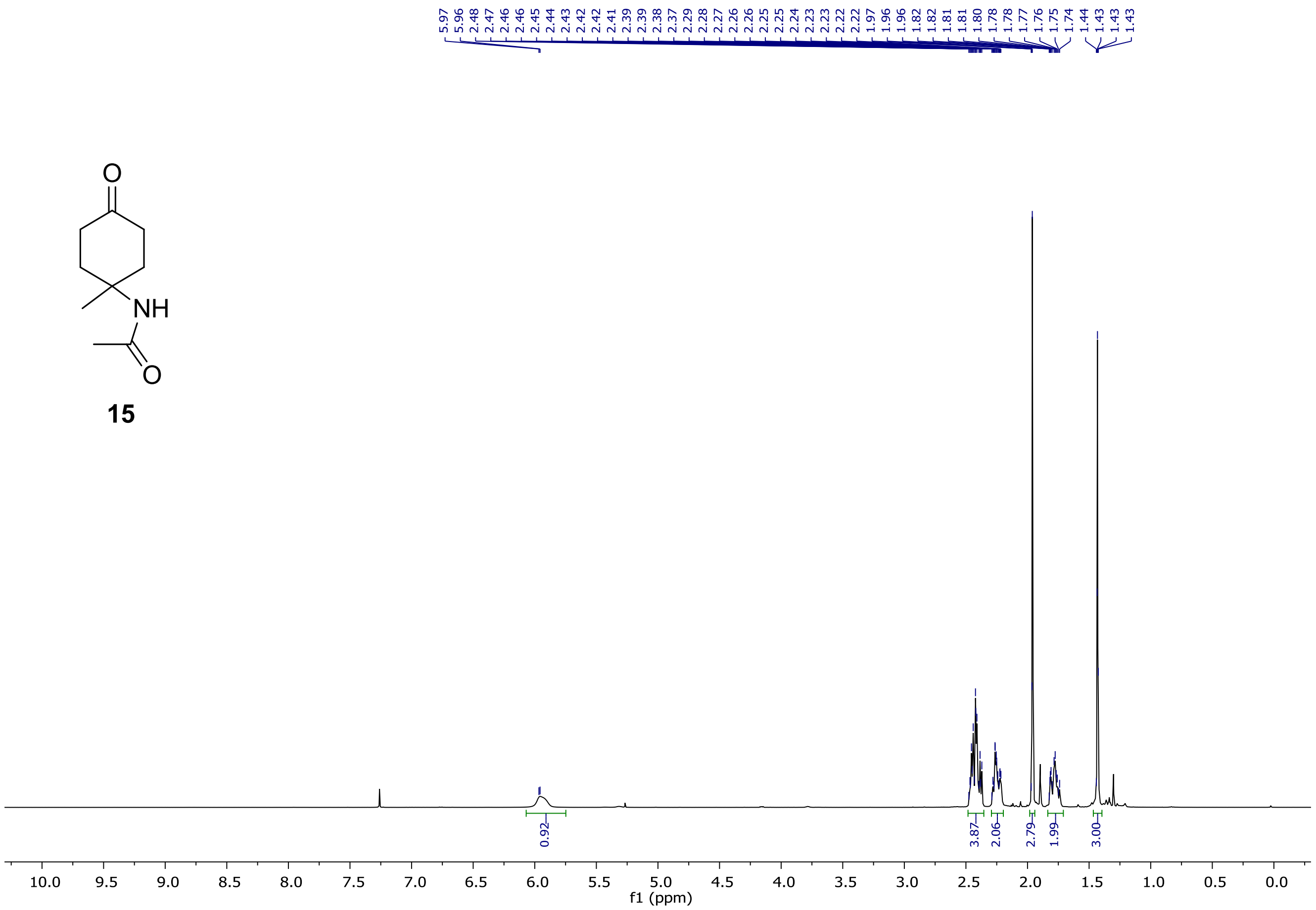

15

^{13}C NMR Spectrum of compound 15

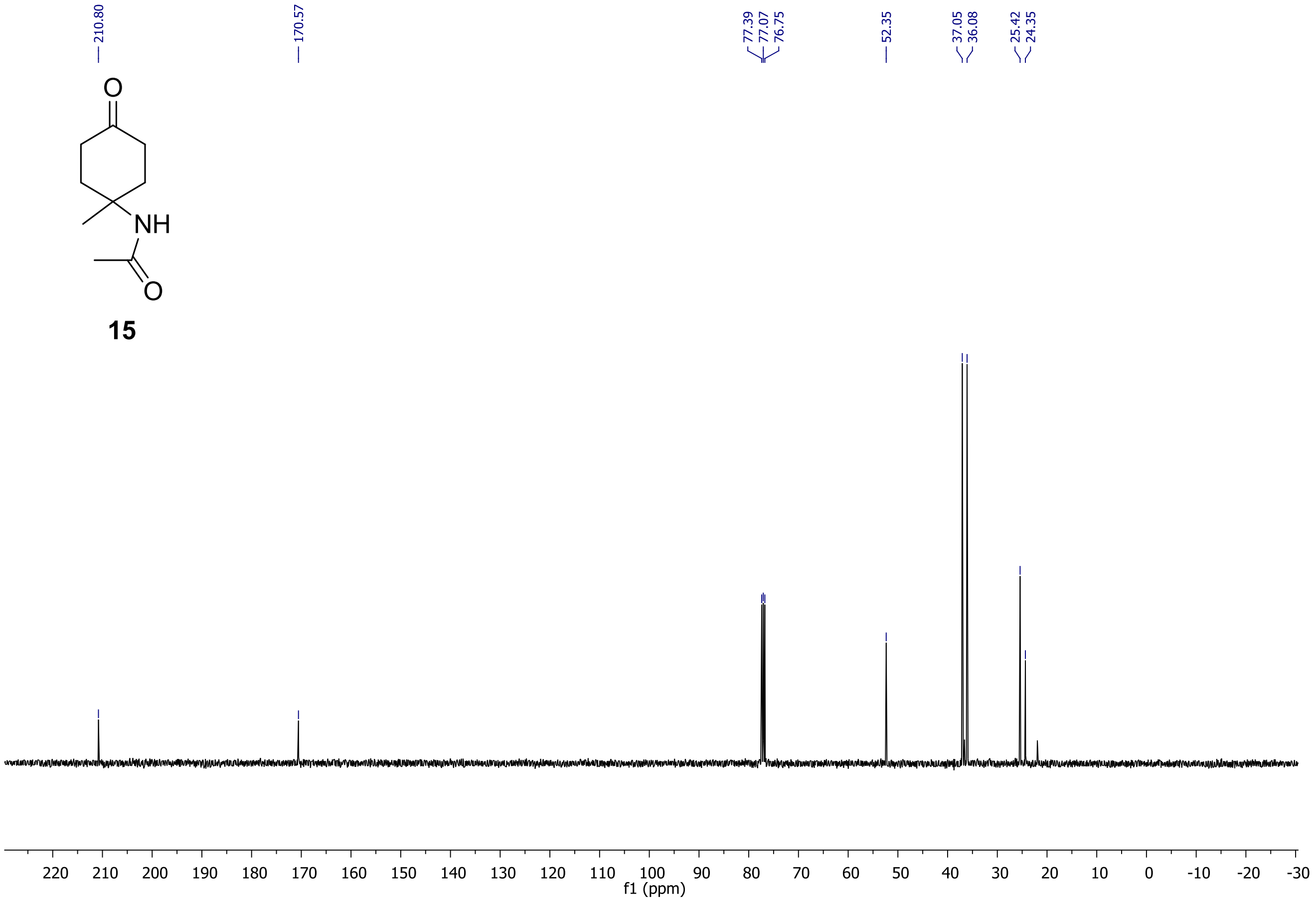

15

^{1}H NMR Spectrum of compound 16

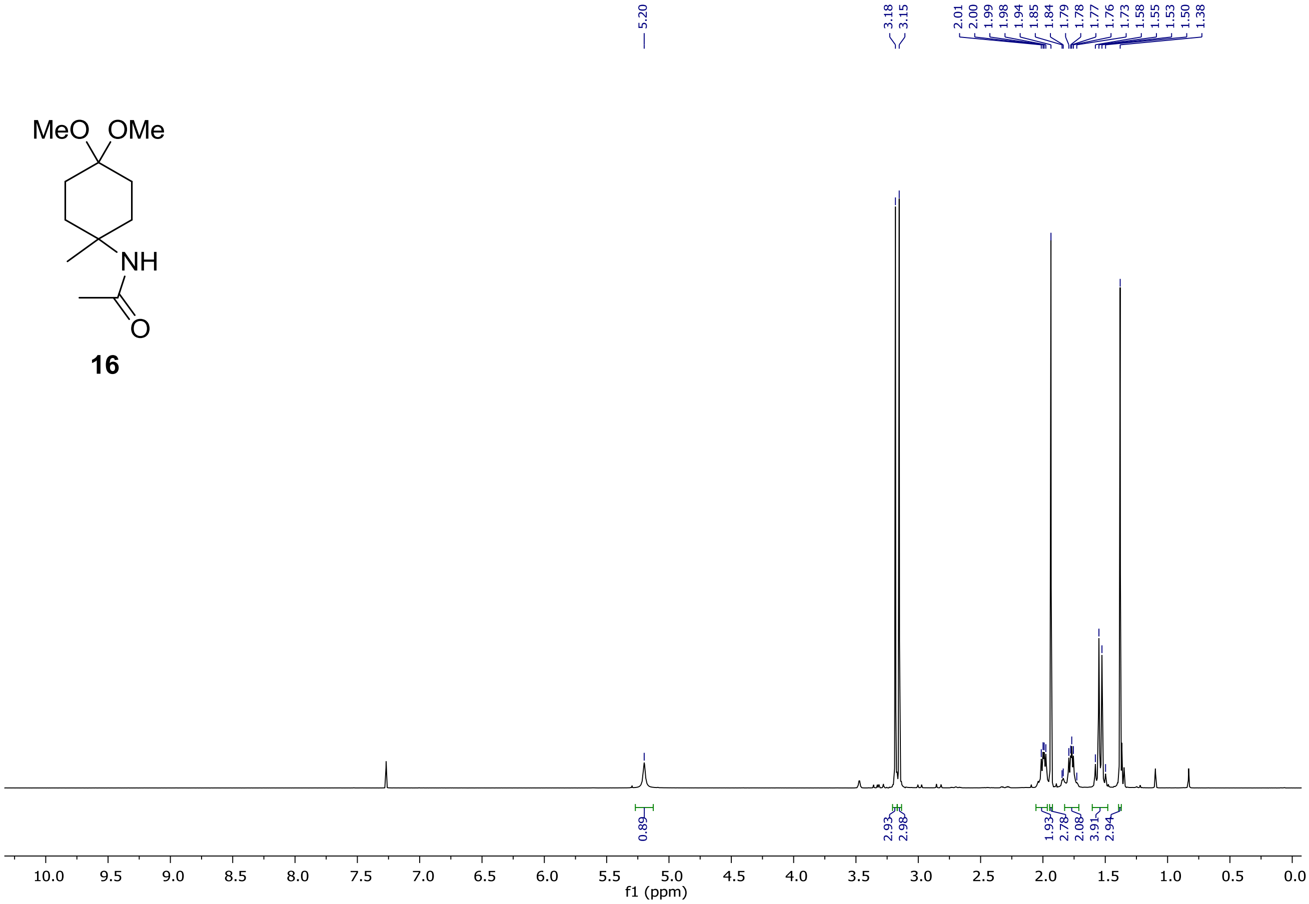

^{13}C NMR Spectrum of compound 16

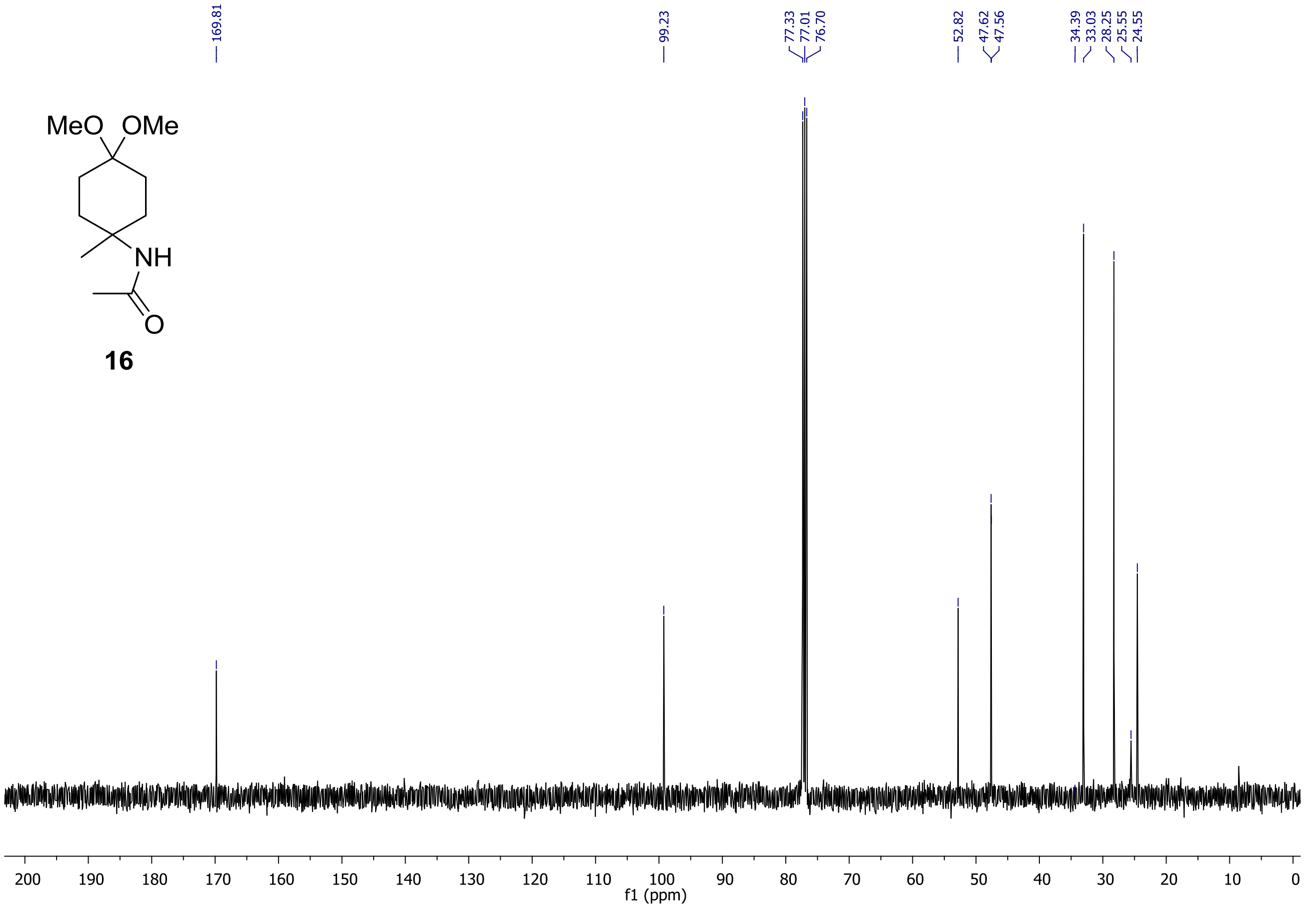

^{1}H NMR Spectrum of compound 17

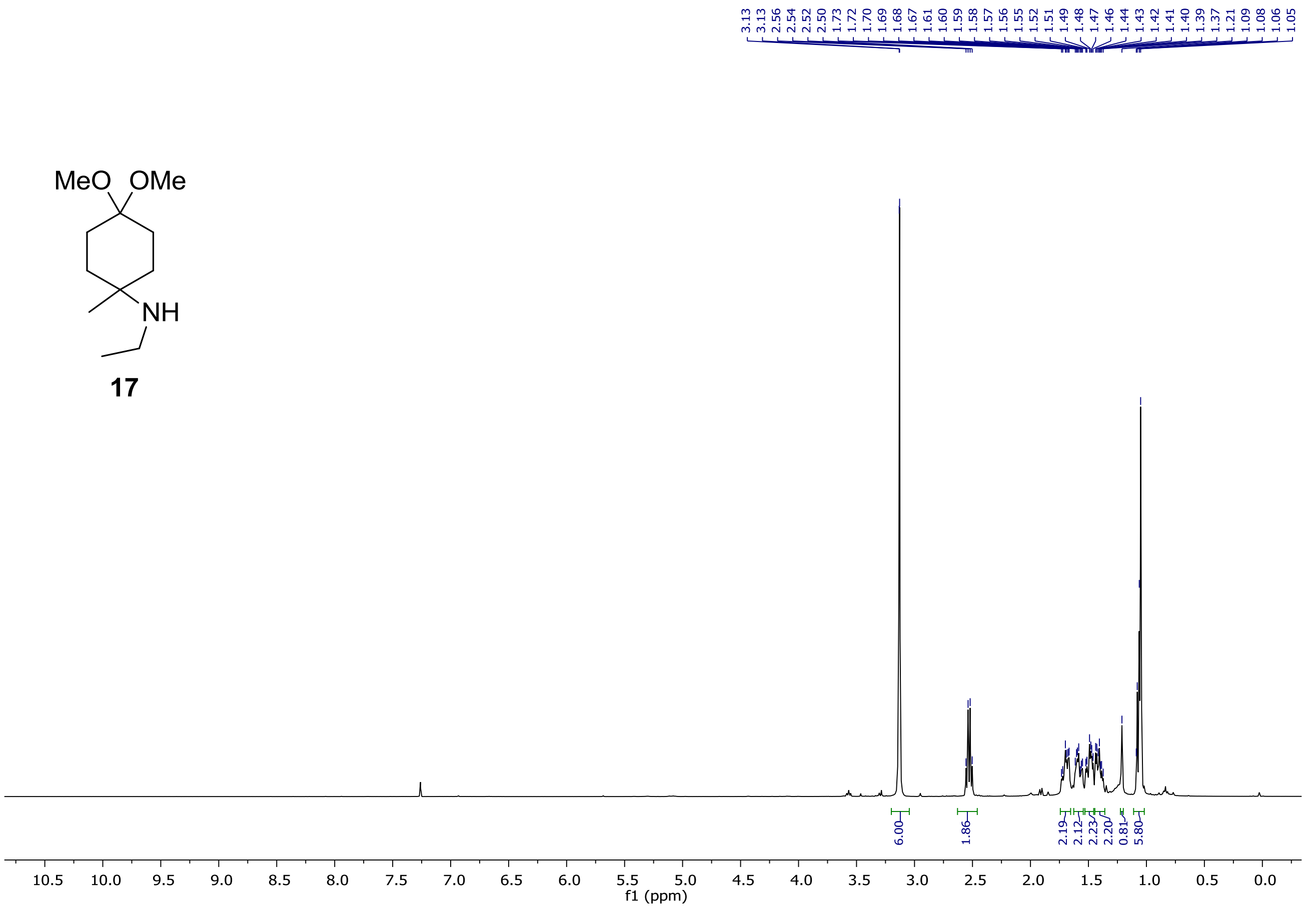

^{13}C NMR Spectrum of compound 17

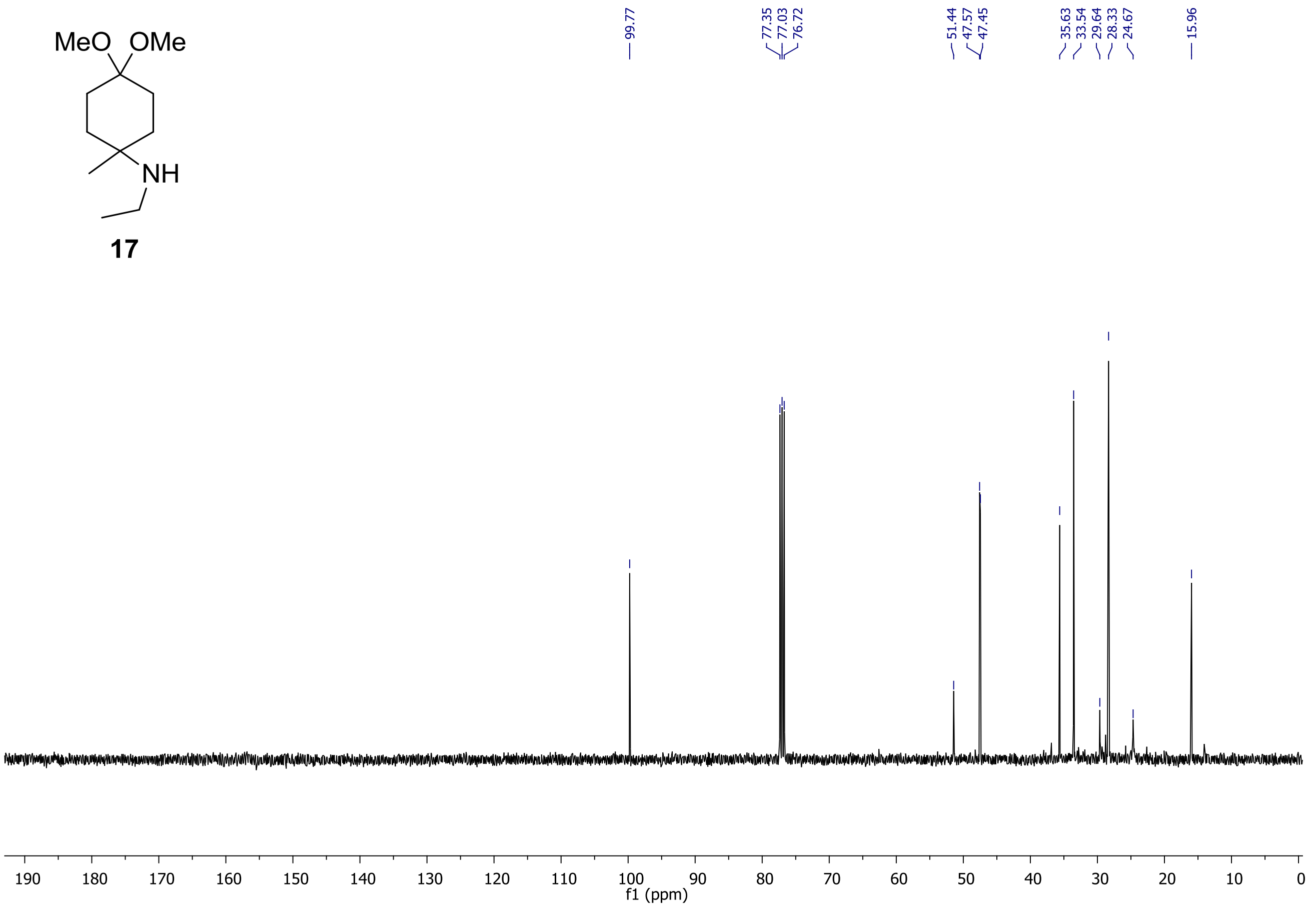

^{1}H NMR Spectrum of compound 18

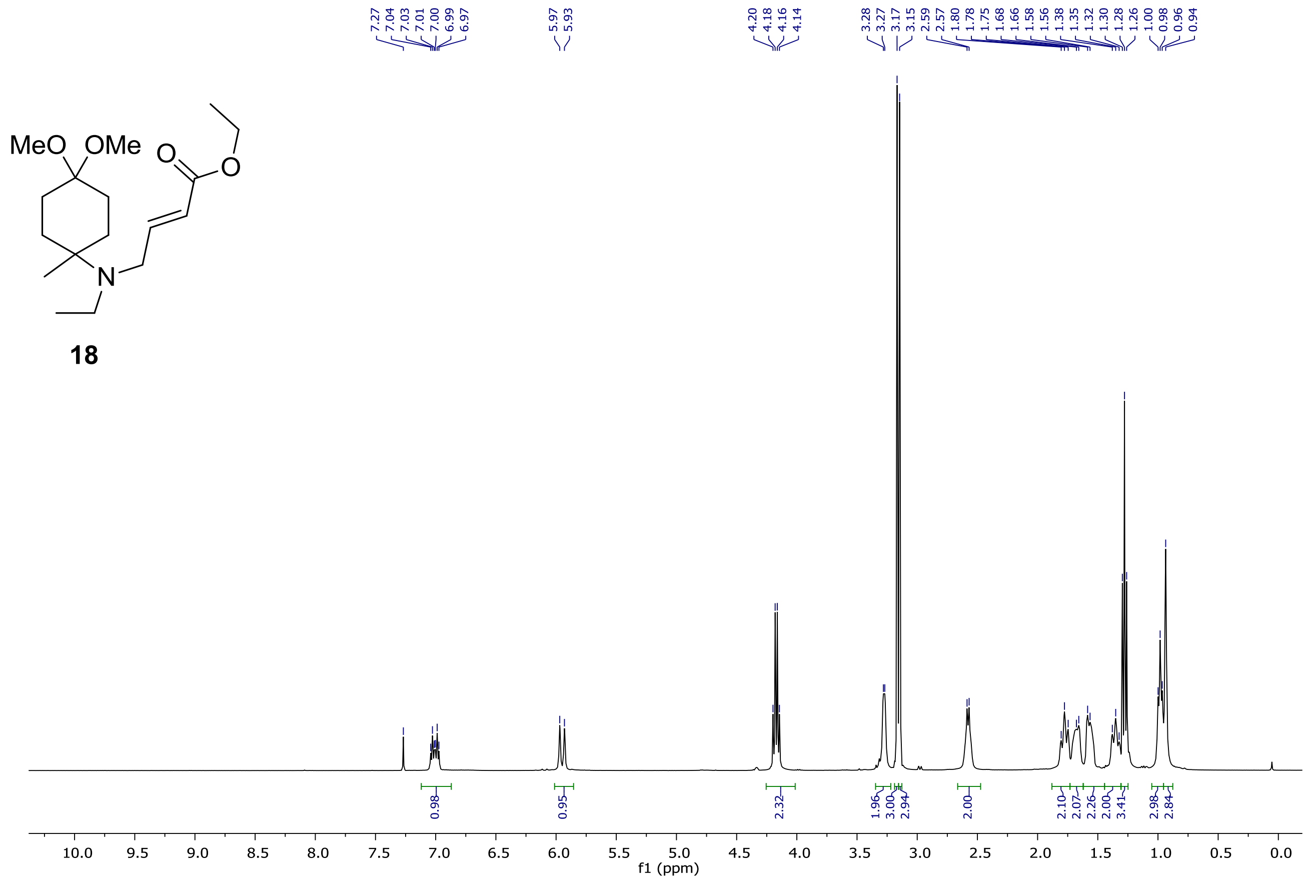

^{13}C NMR Spectrum of compound 18

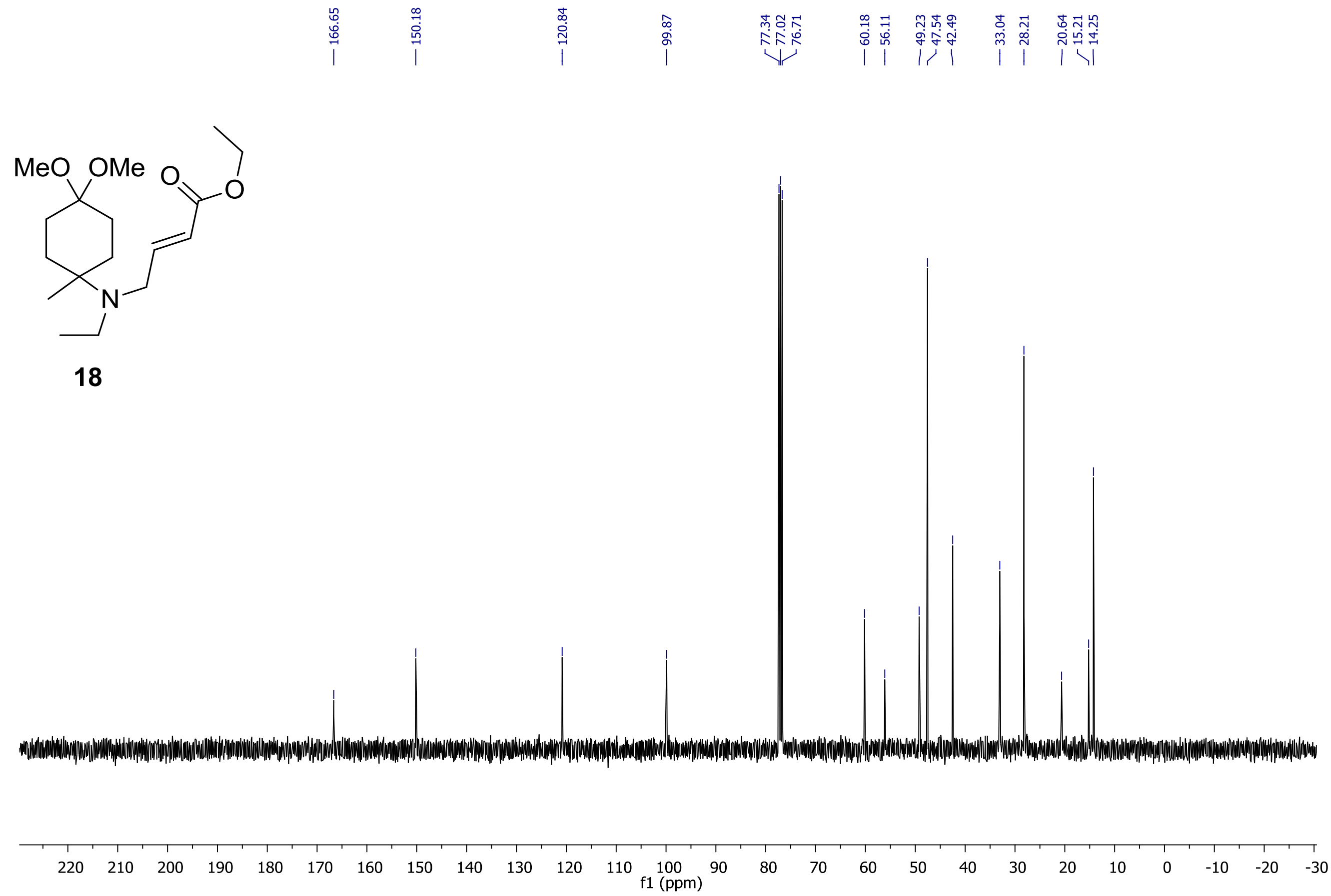

^{1}H NMR Spectrum of compound 2i

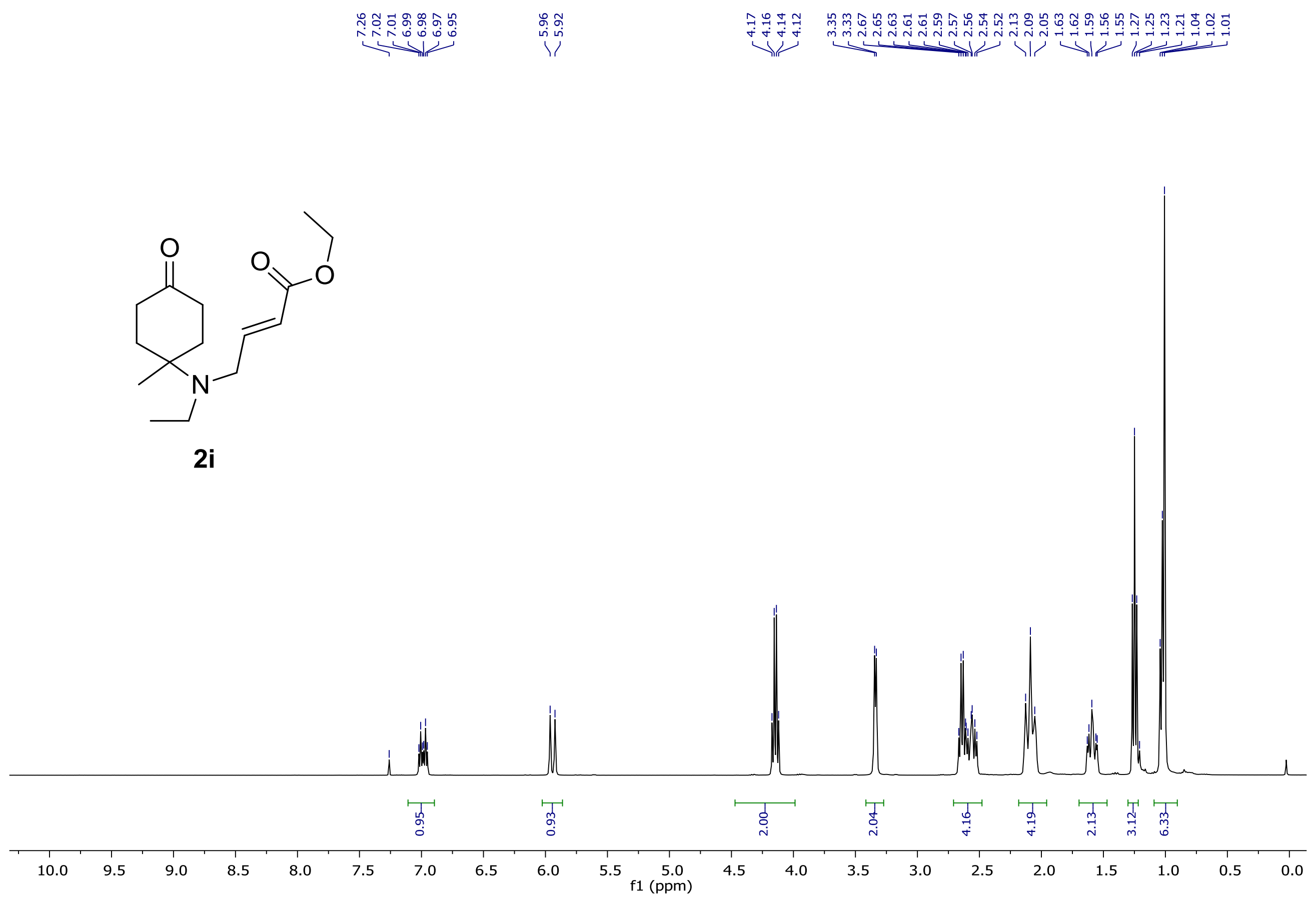

^{13}C NMR Spectrum of compound 2i

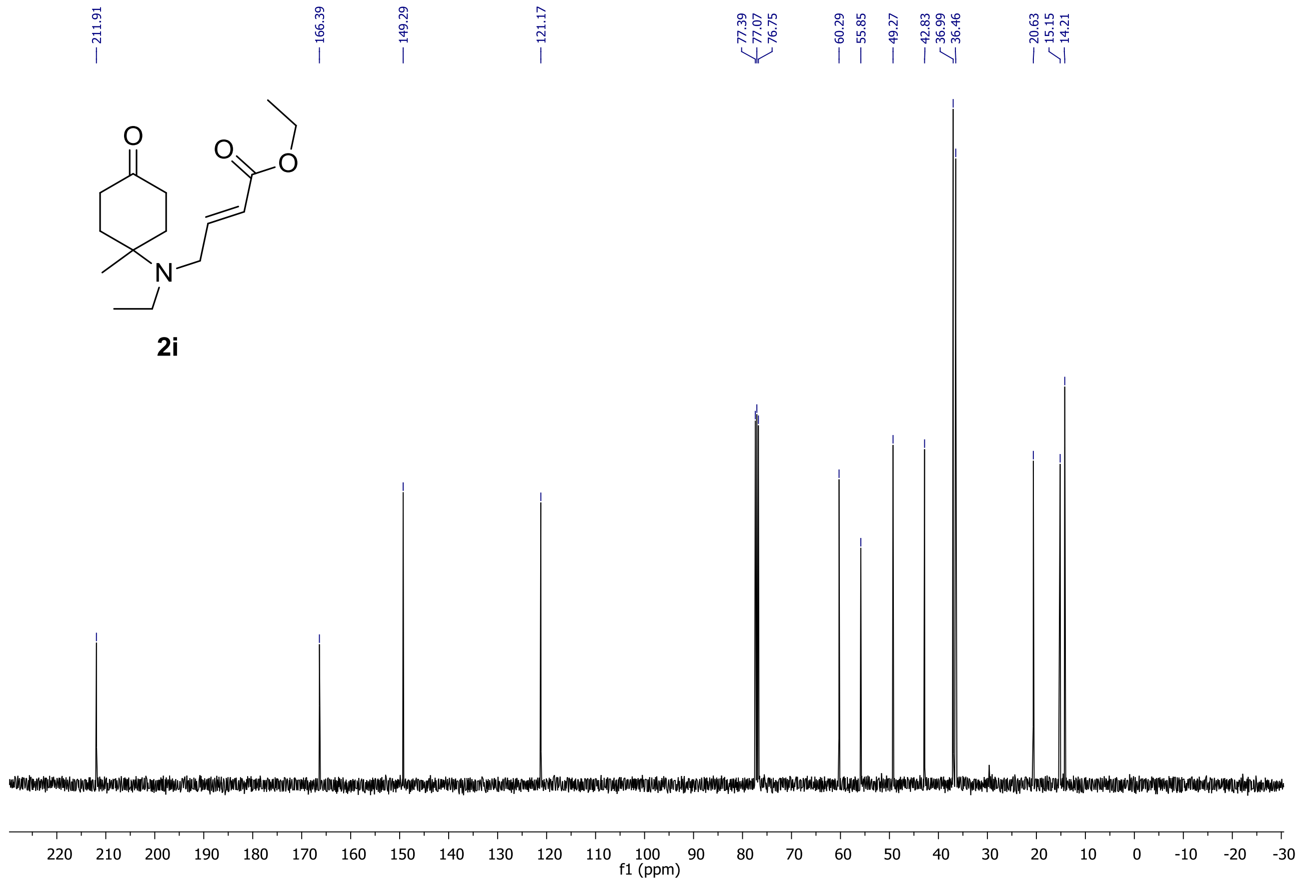

^{1}H NMR Spectrum of compound 20

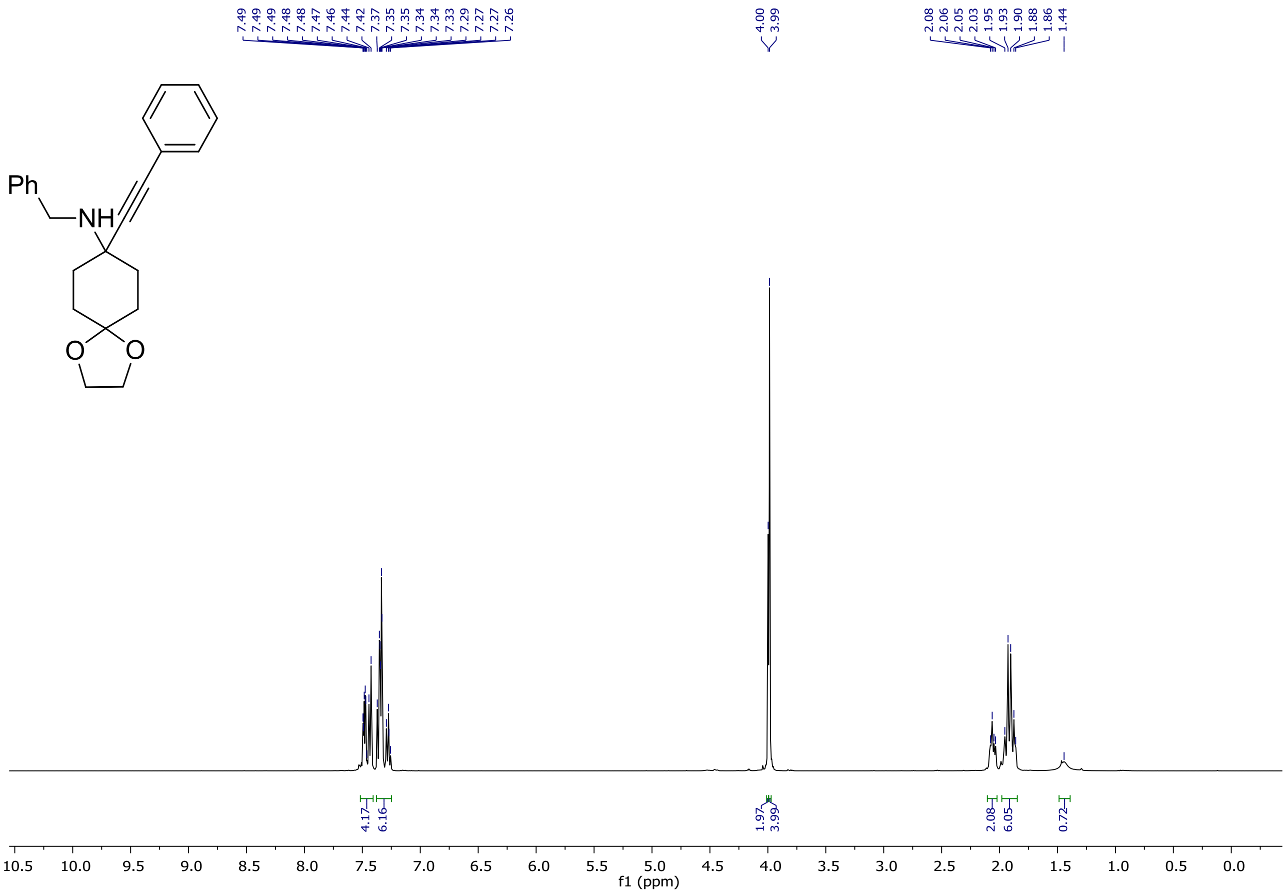

^{13}C NMR Spectrum of compound 20

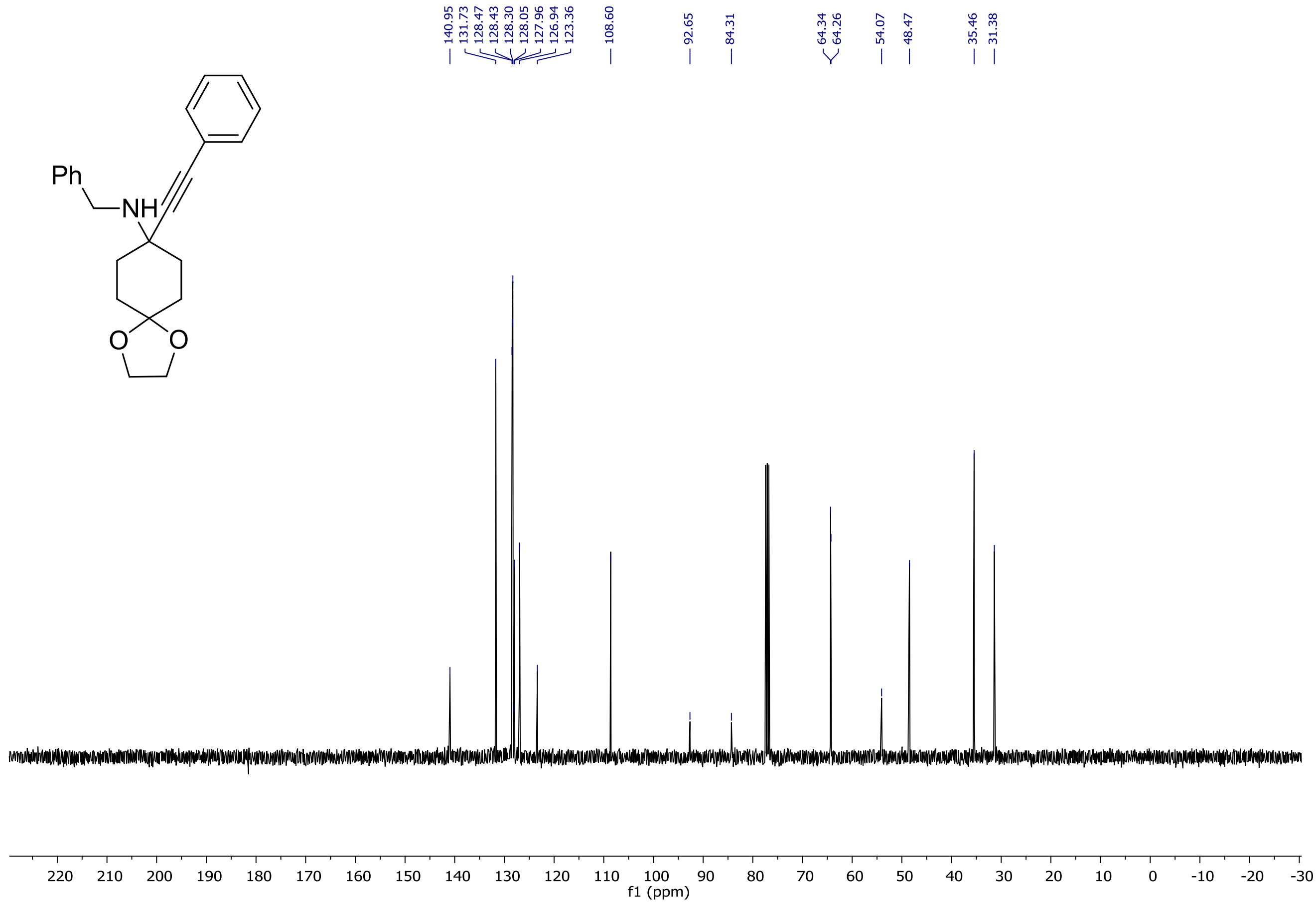

^{1}H NMR Spectrum of compound 21

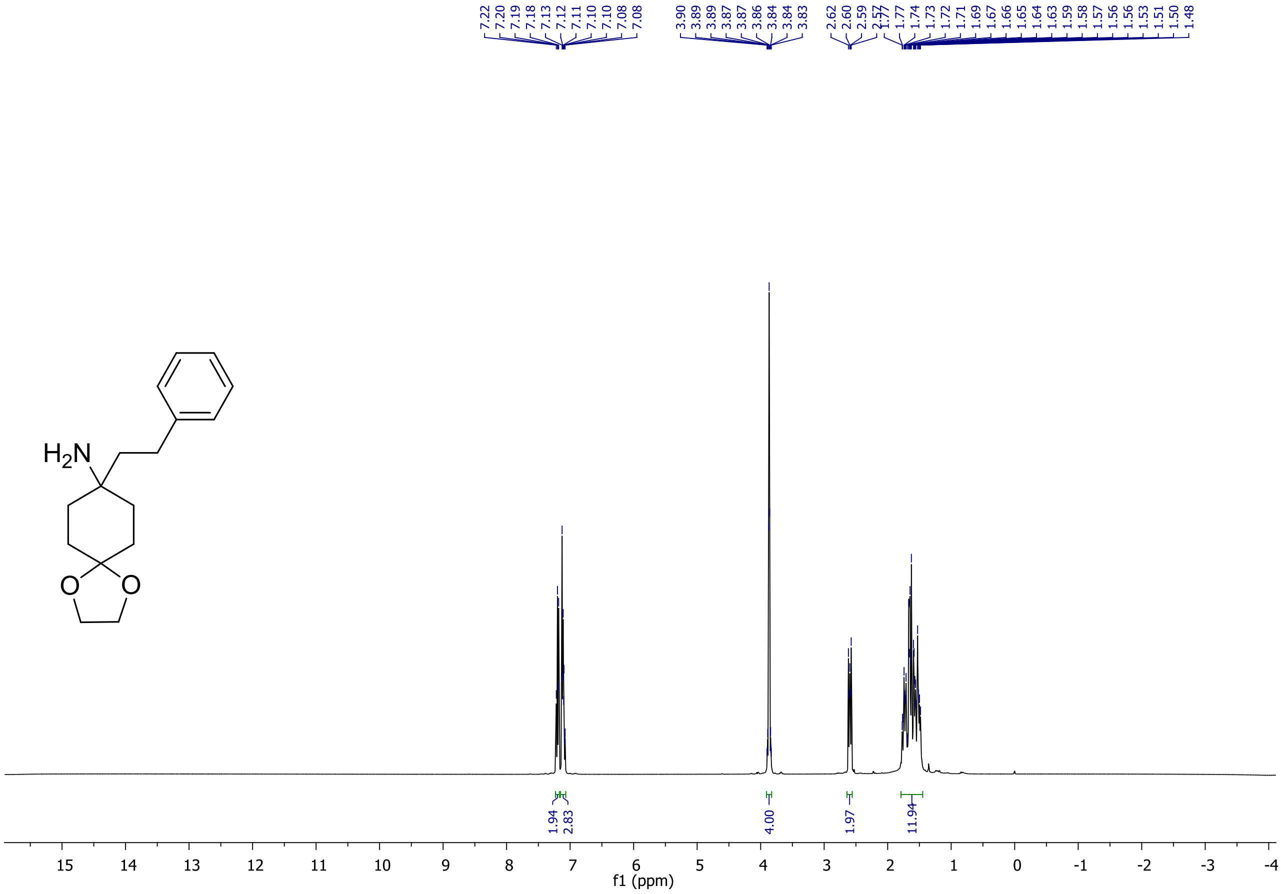

^{13}C NMR Spectrum of compound 21

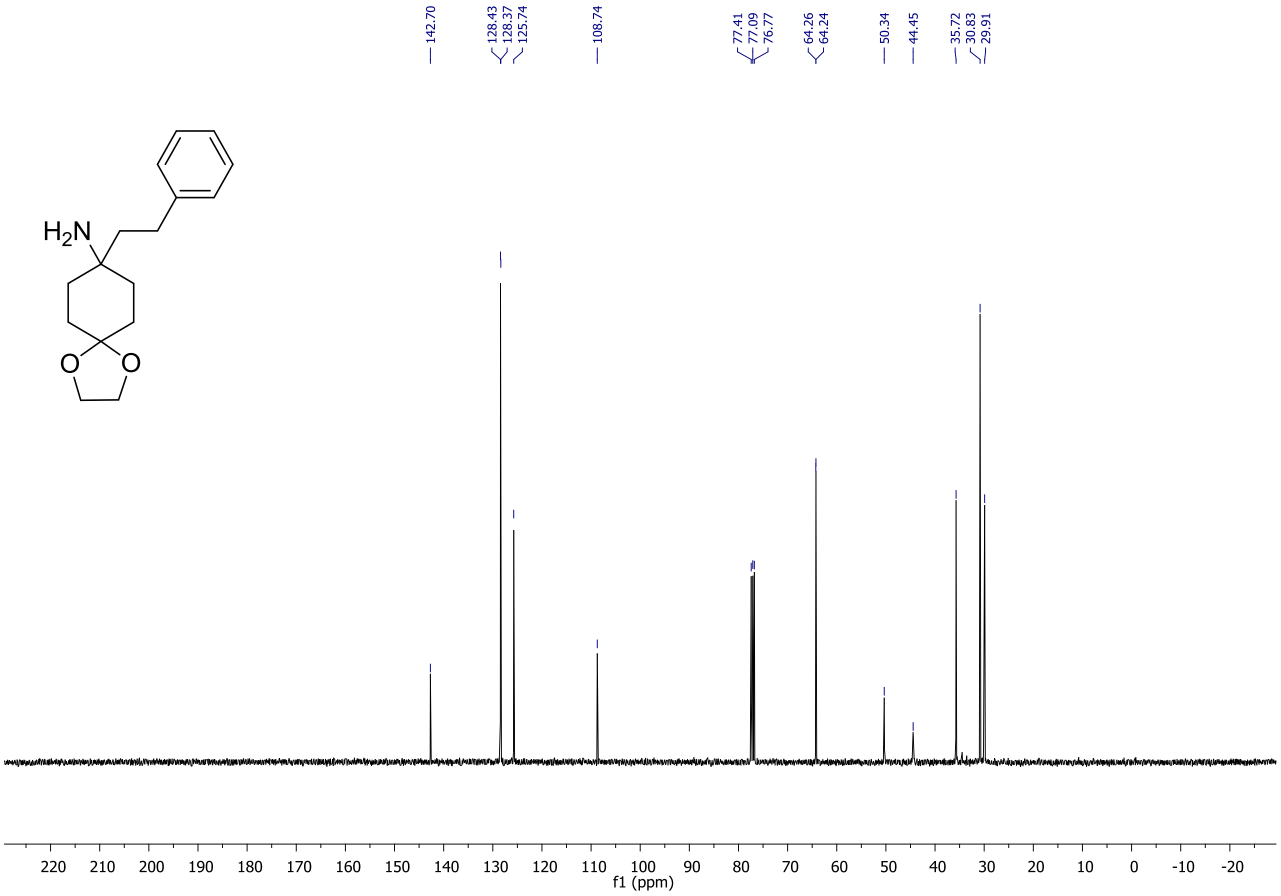

^{1}H NMR Spectrum of compound 22

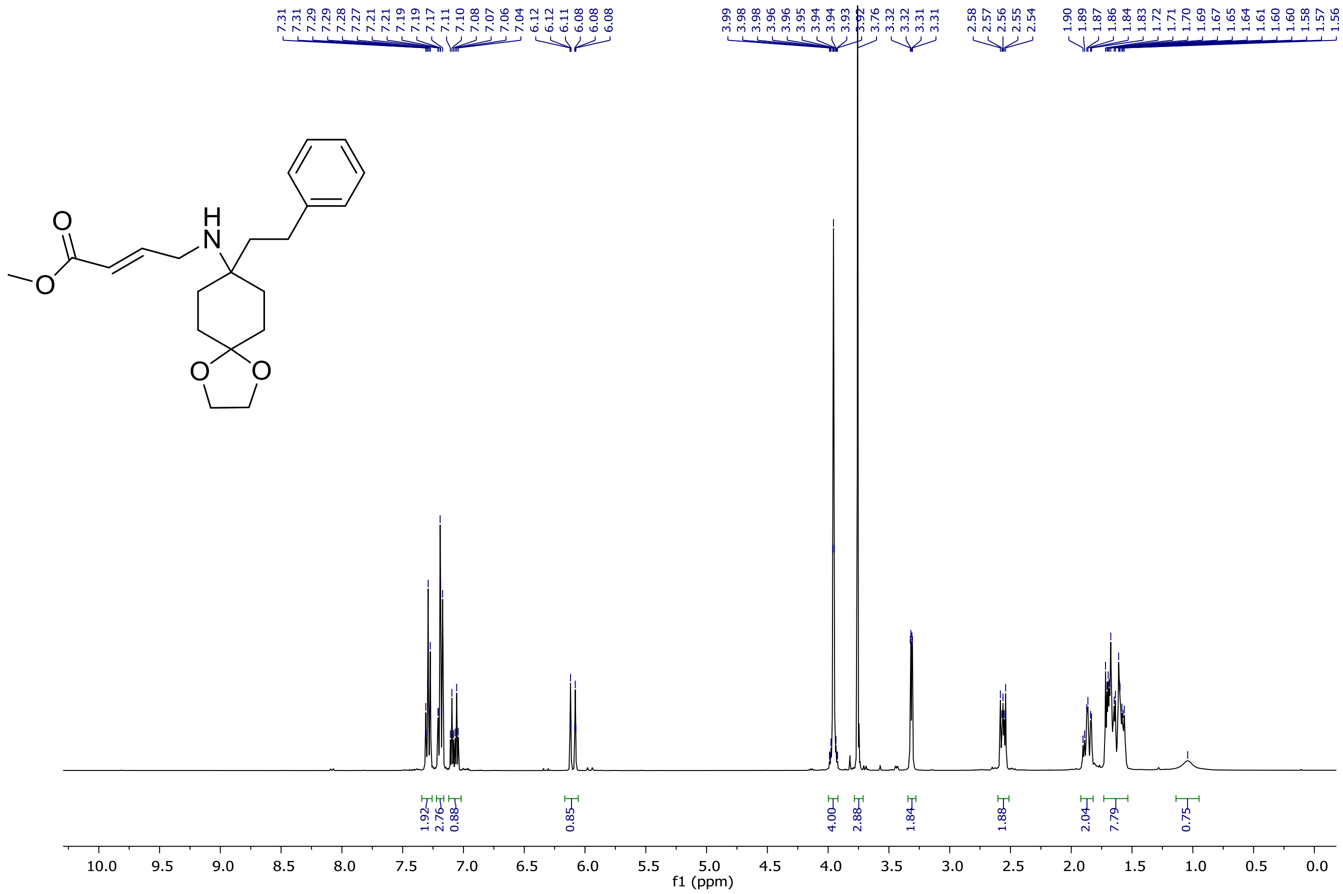

^{13}C NMR Spectrum of compound 22

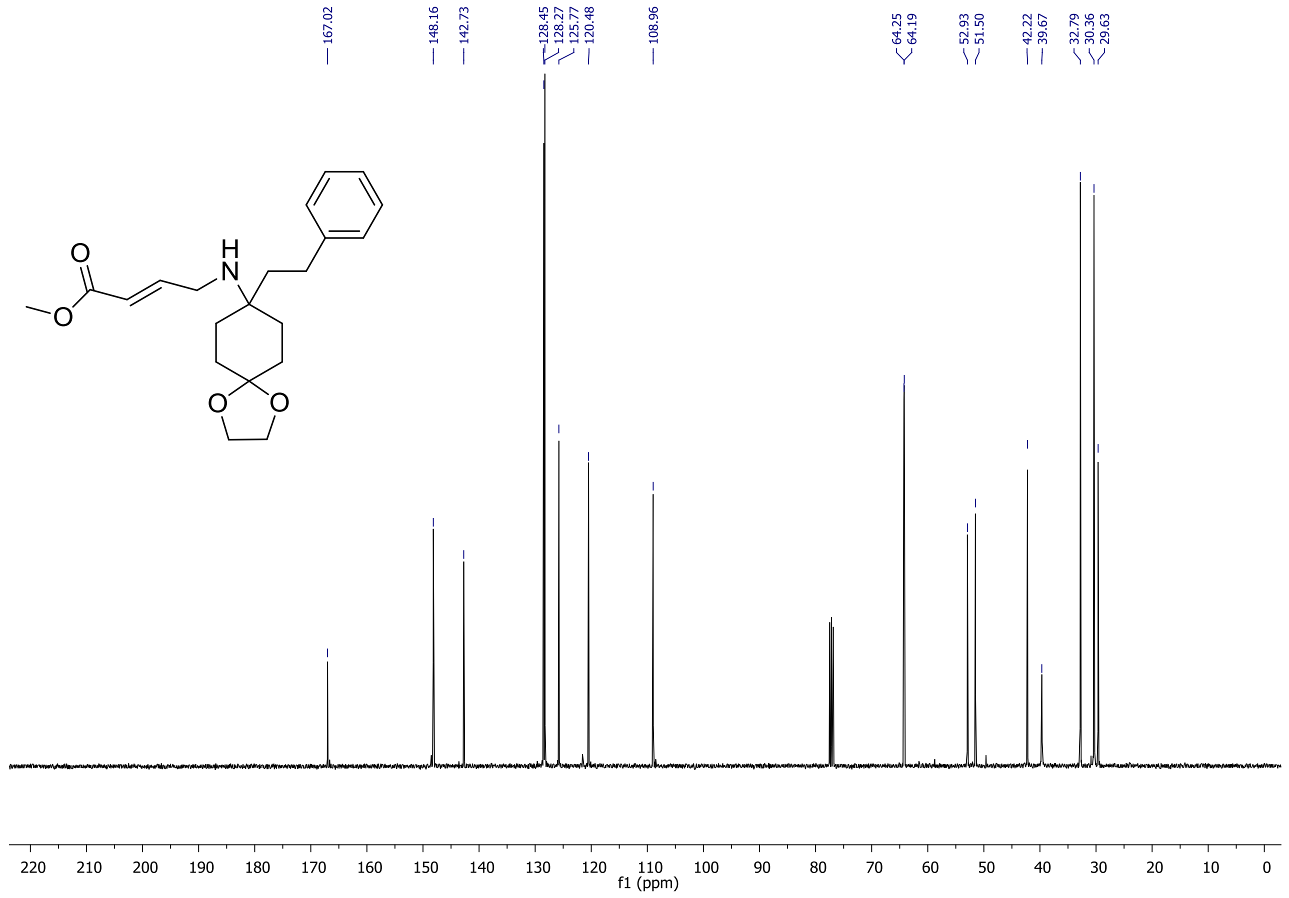

^{1}H NMR Spectrum of compound 2j

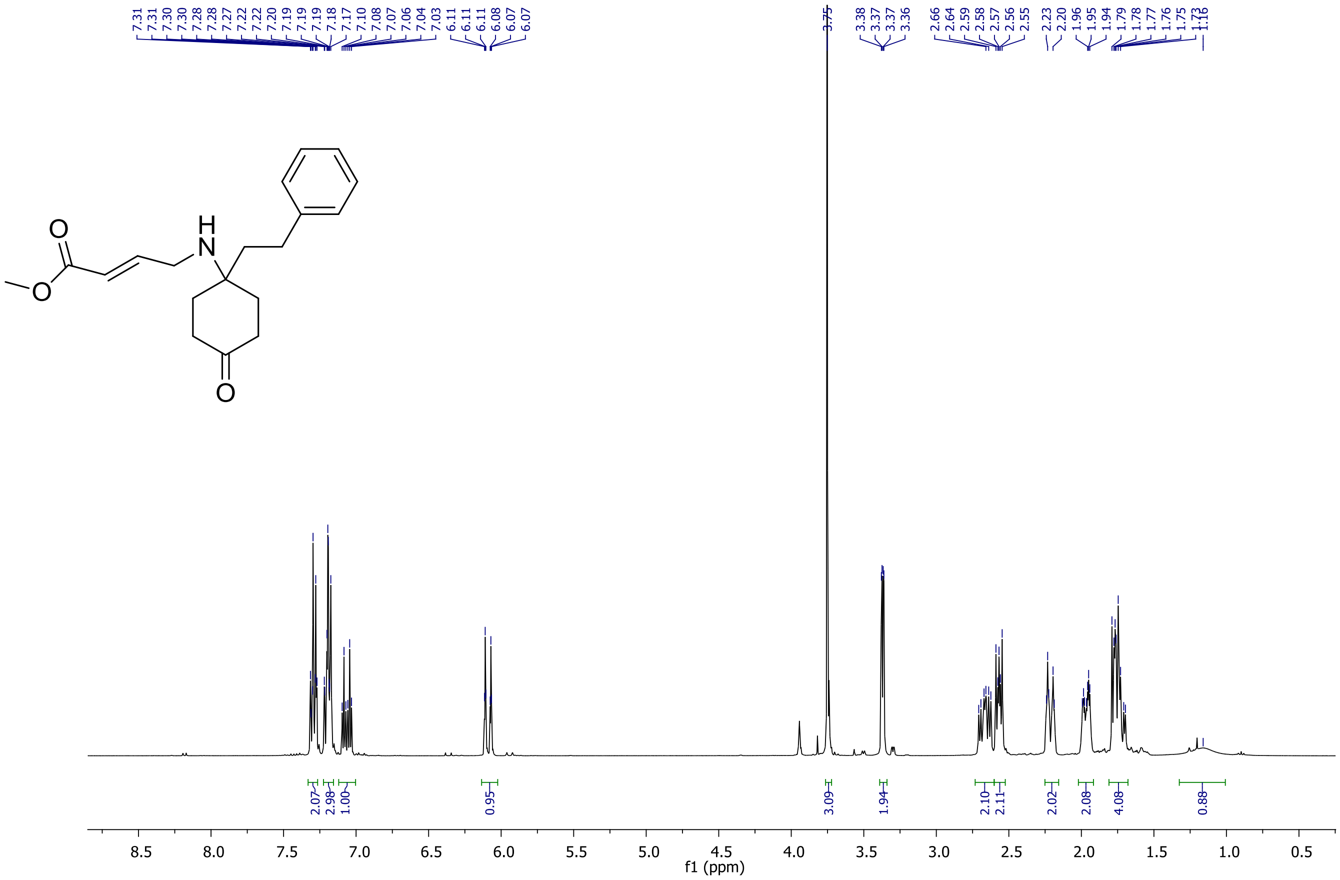

^{13}C NMR Spectrum of compound 2j

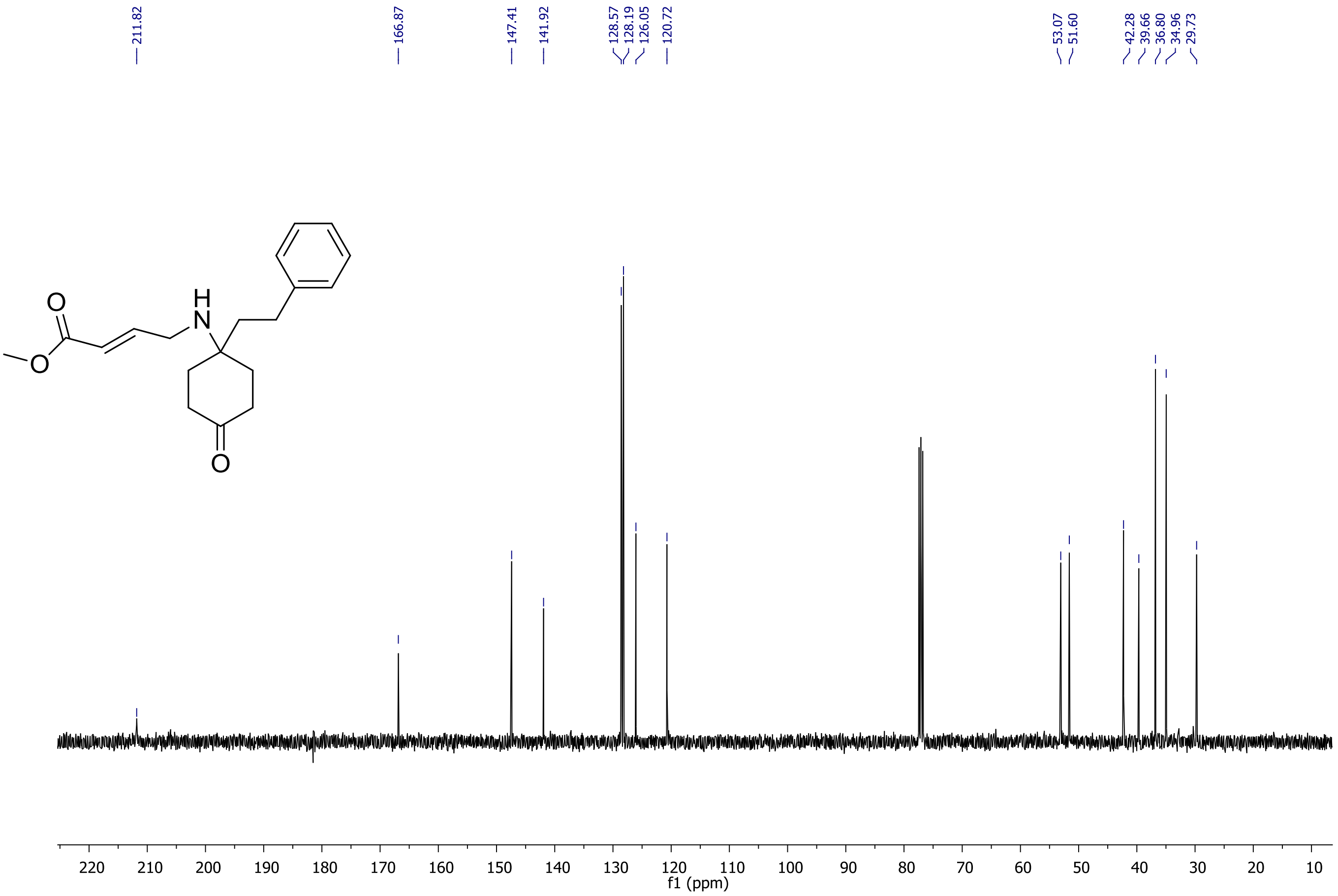

^{1}H NMR Spectrum of compound 23

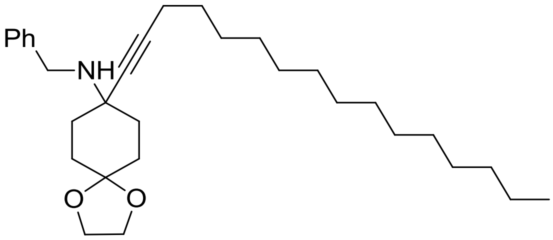

23

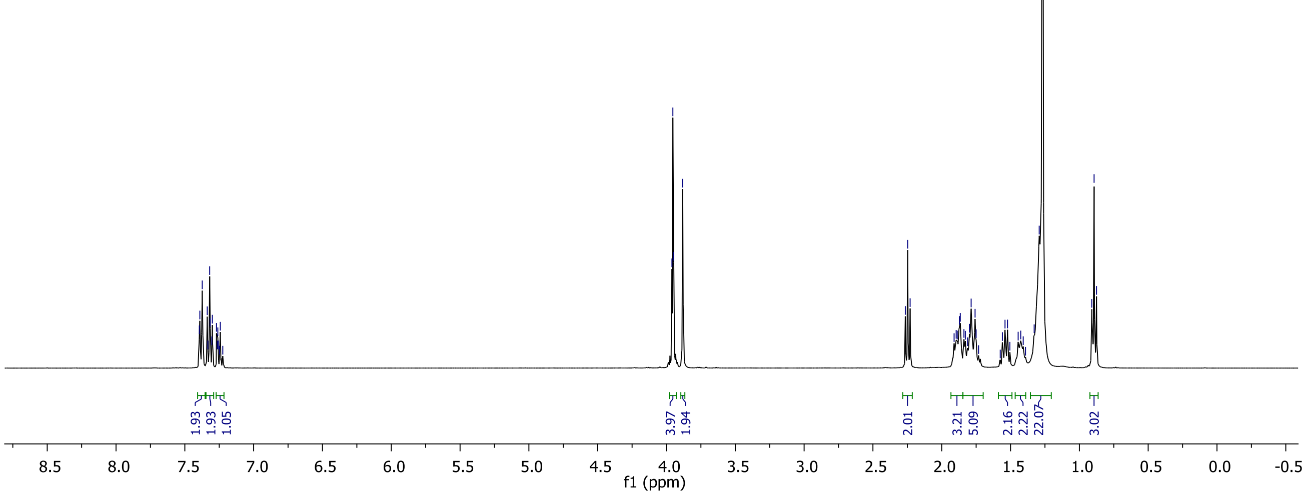

^{13}C NMR Spectrum of compound 23

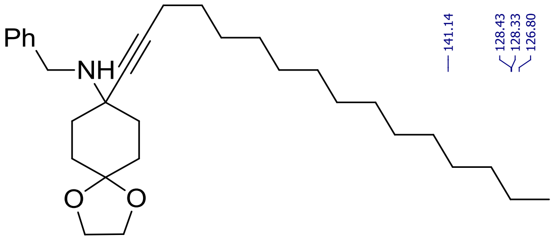

23

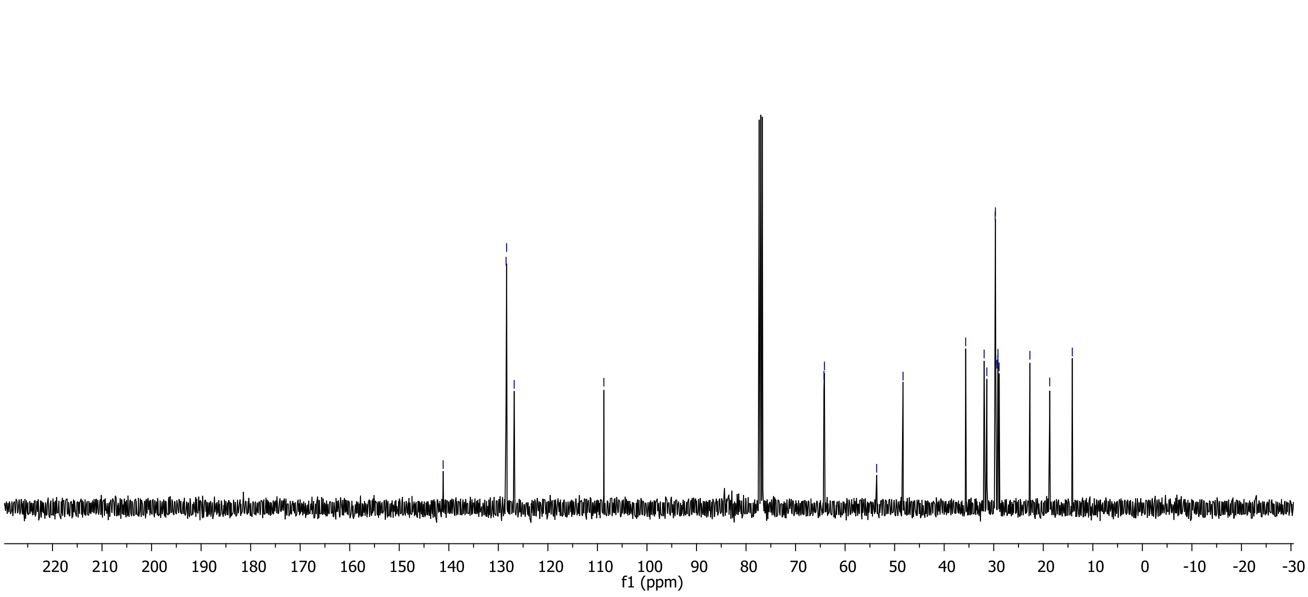

¹H NMR Spectrum of compound 24

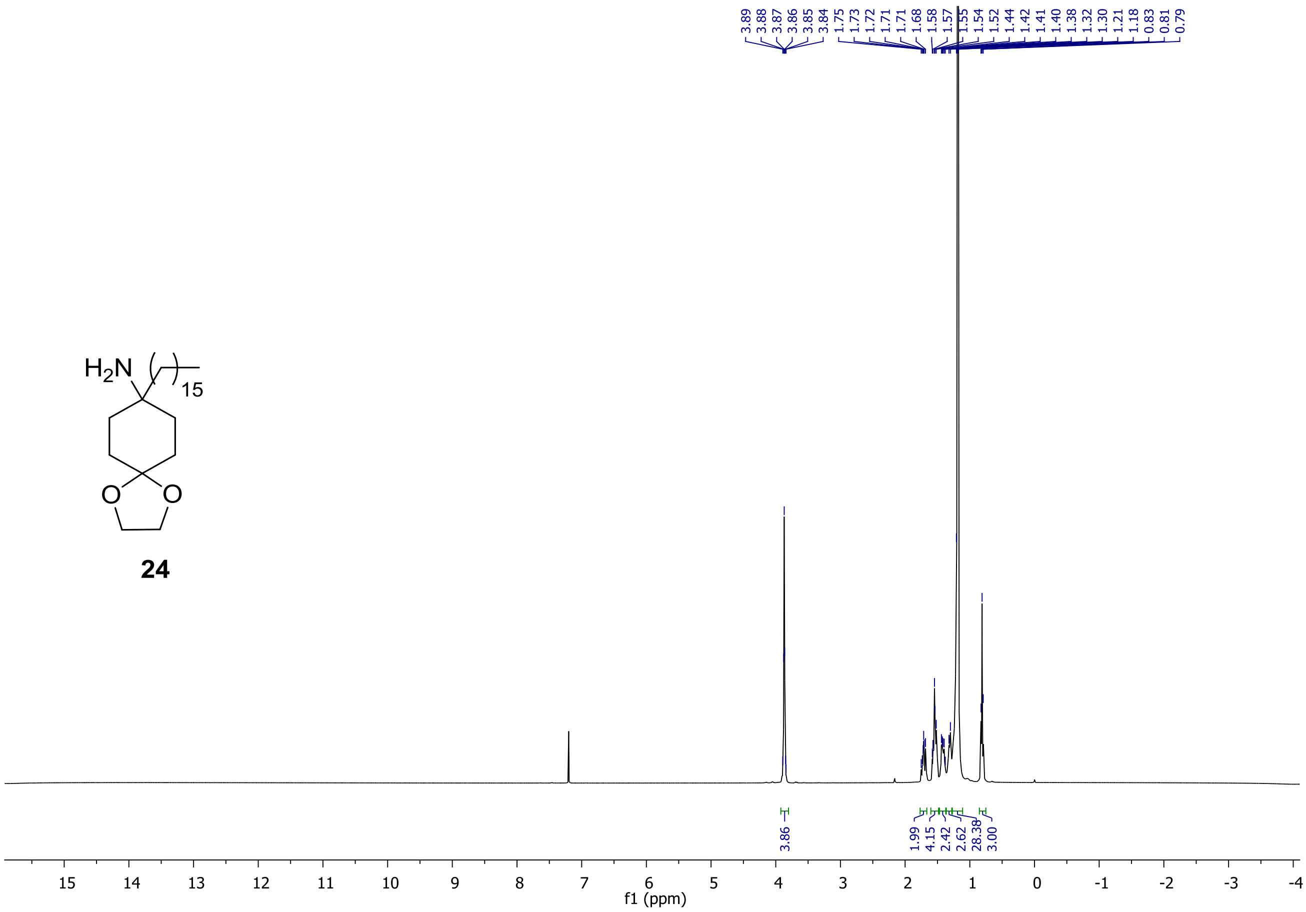

¹³C NMR Spectrum of compound 24

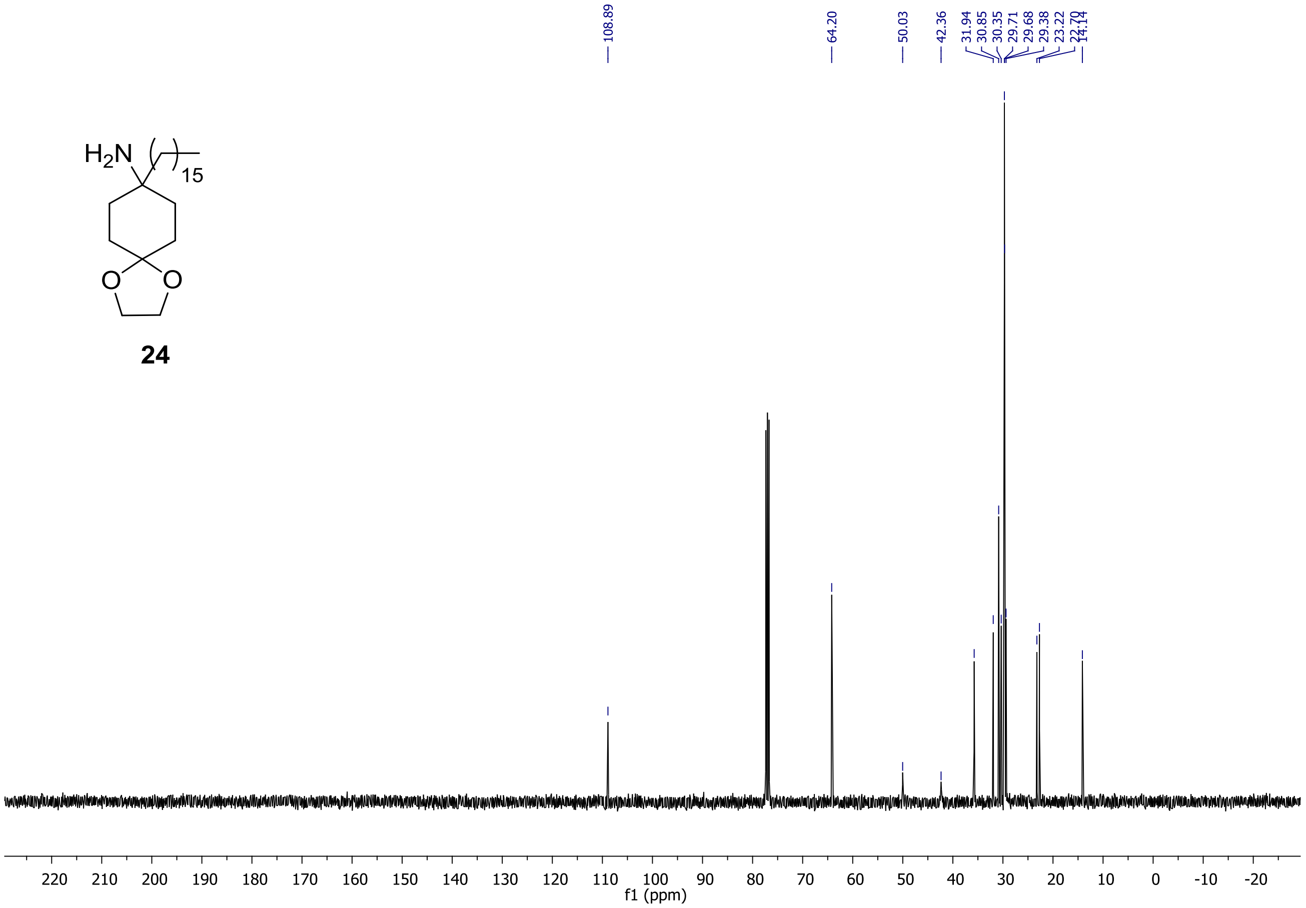

^{1}H NMR Spectrum of compound 25

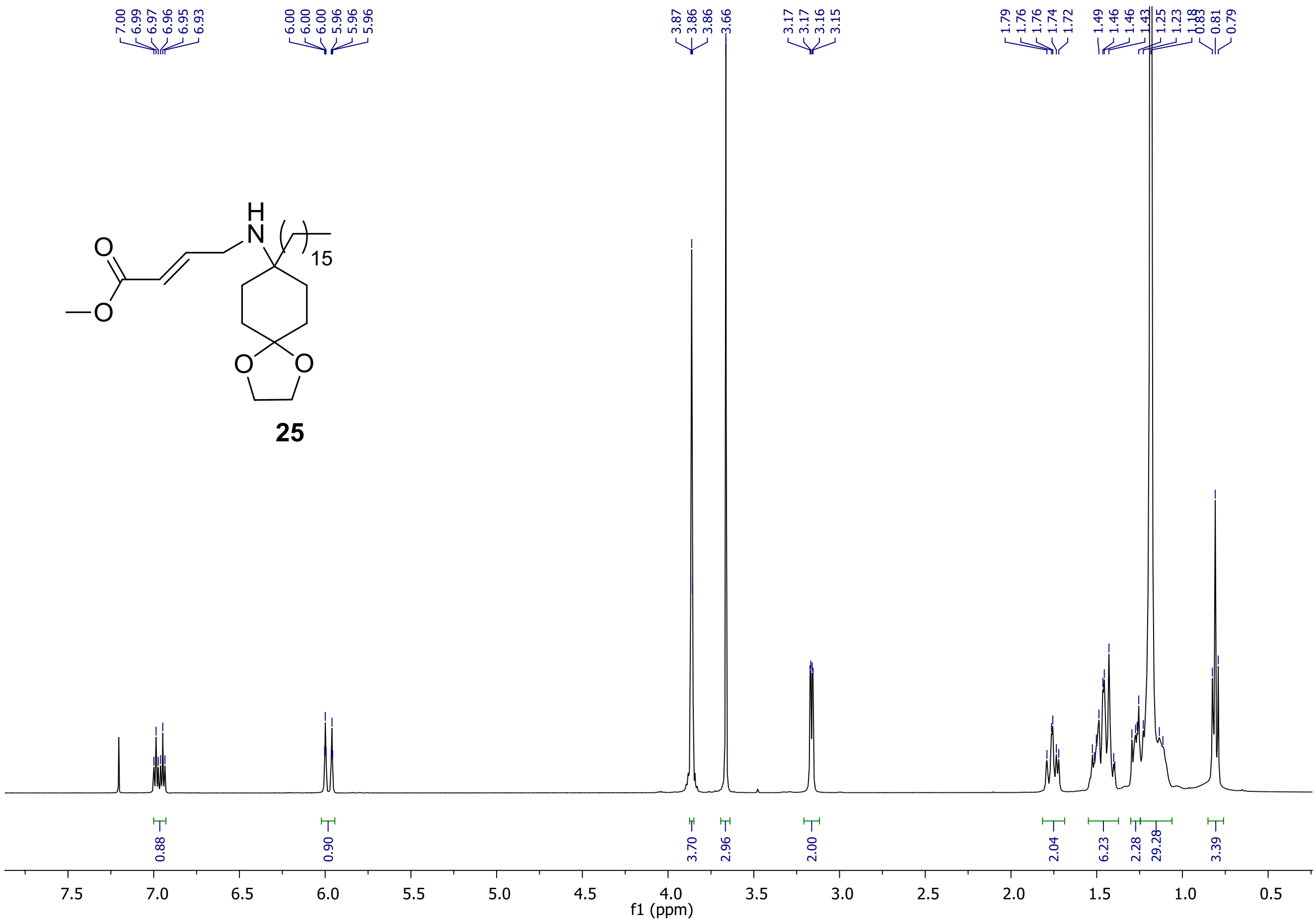

^{13}C NMR Spectrum of compound 25

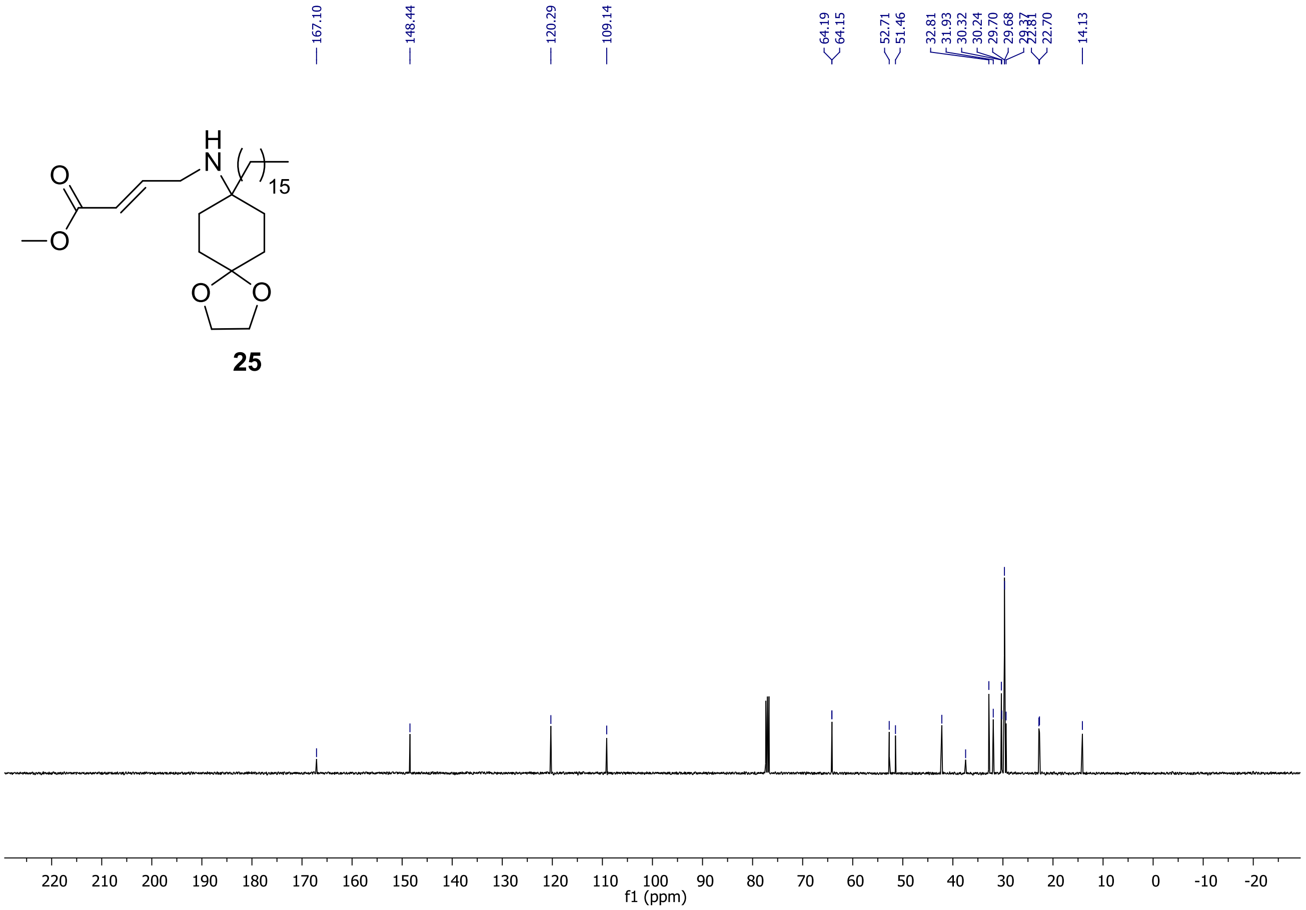

^{1}H NMR Spectrum of compound 2k

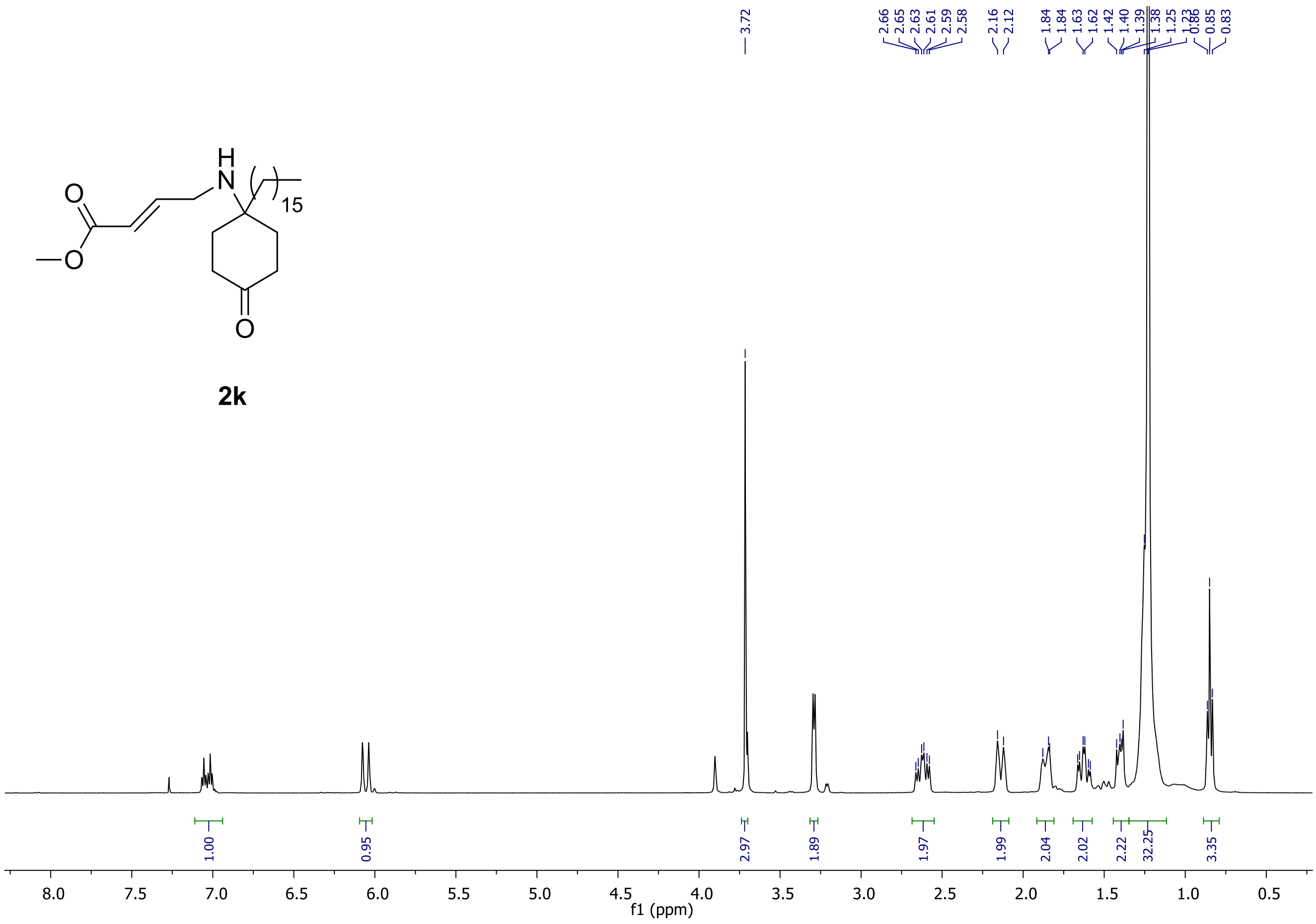

^{13}C NMR Spectrum of compound 2k

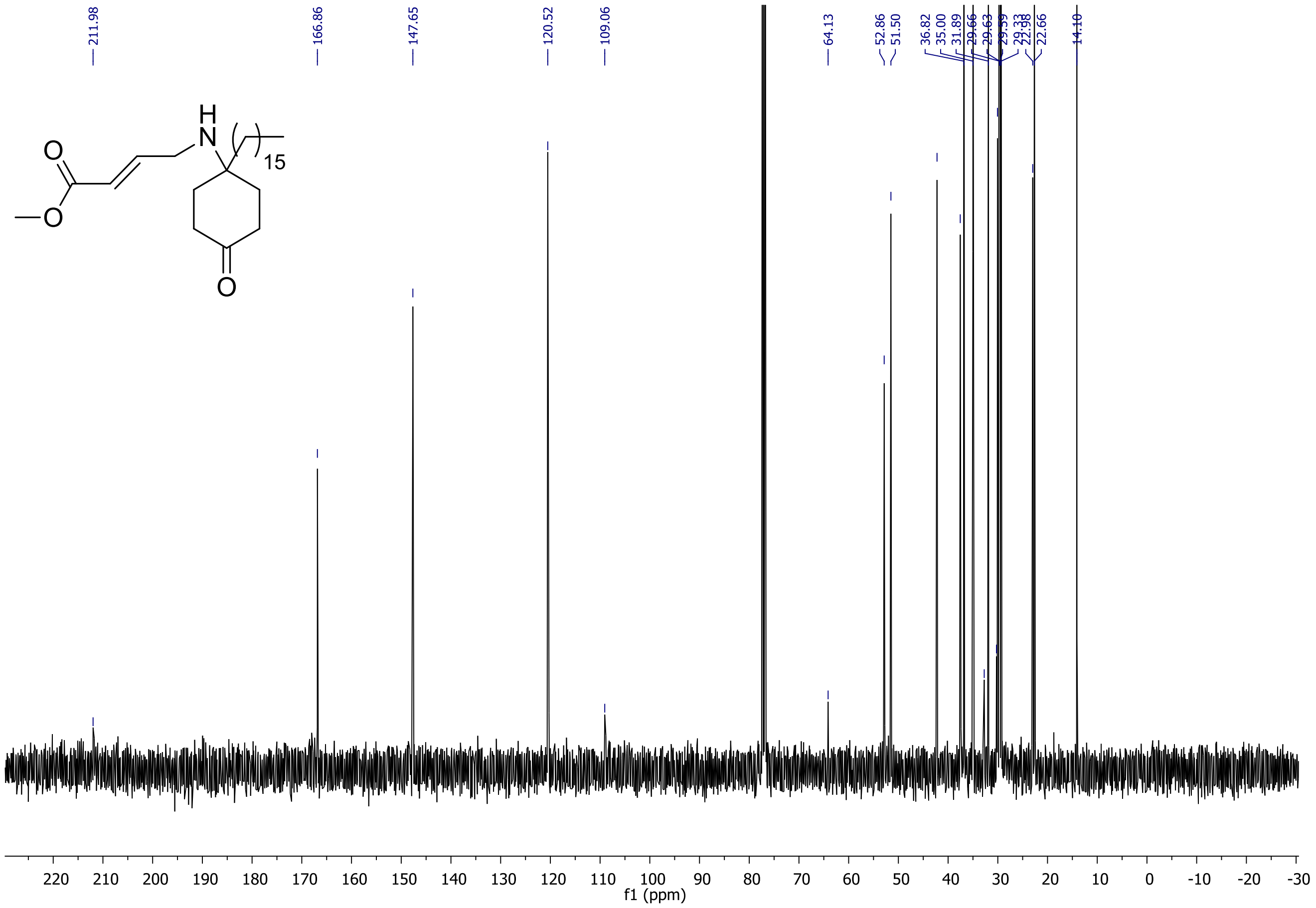

^{1}H NMR Spectrum of compound 27l

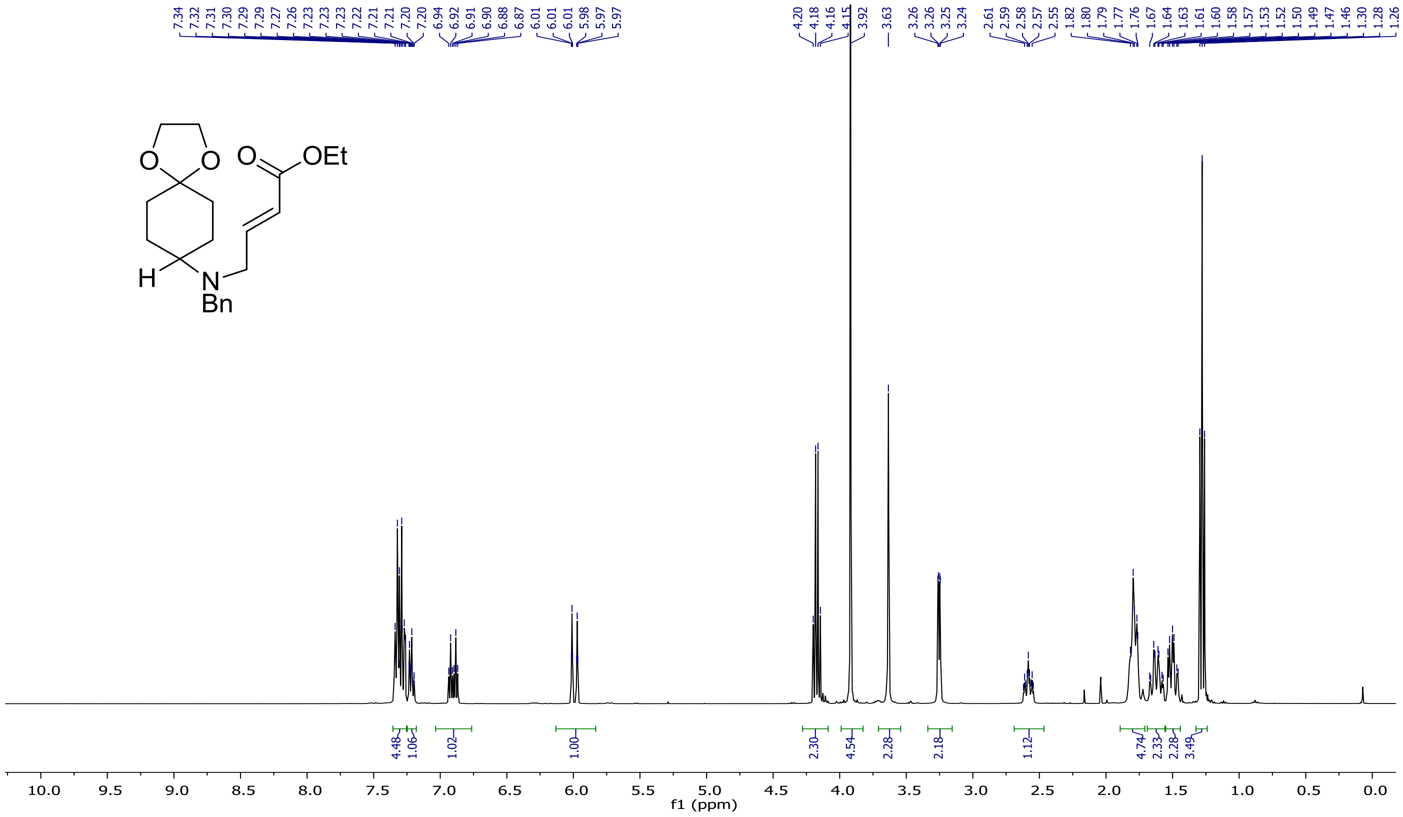

^{13}C NMR Spectrum of compound 27l

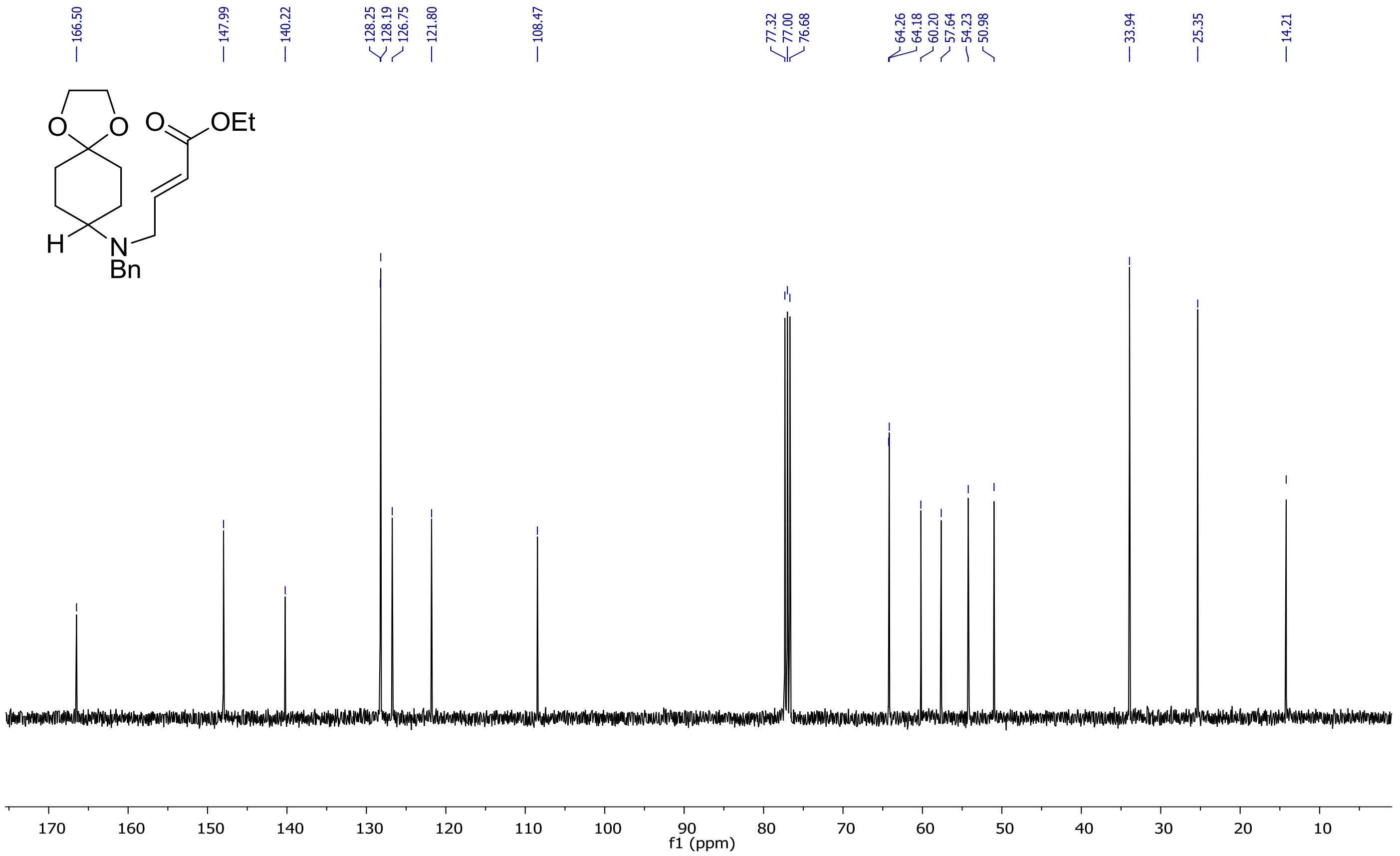

^{1}H NMR Spectrum of compound 2l

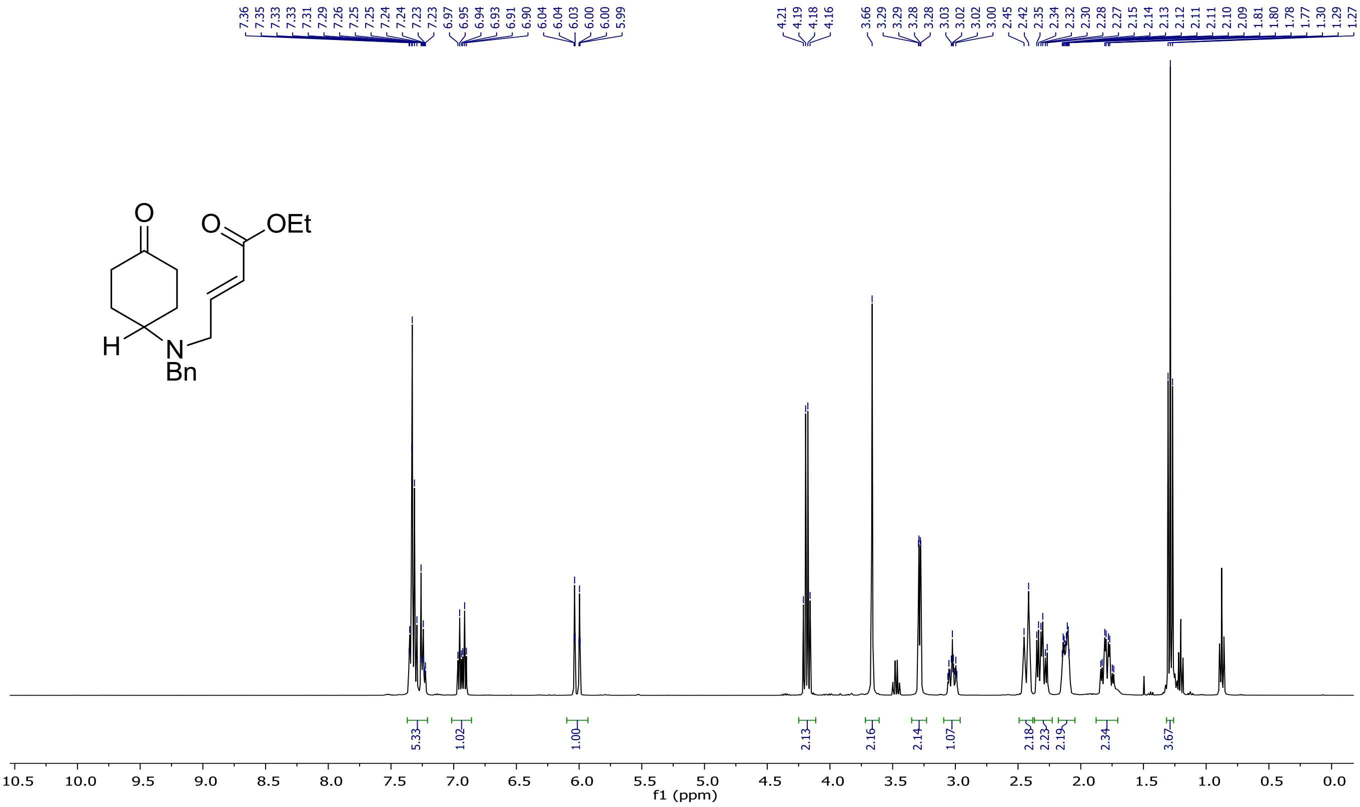

^{13}C NMR Spectrum of compound 2l

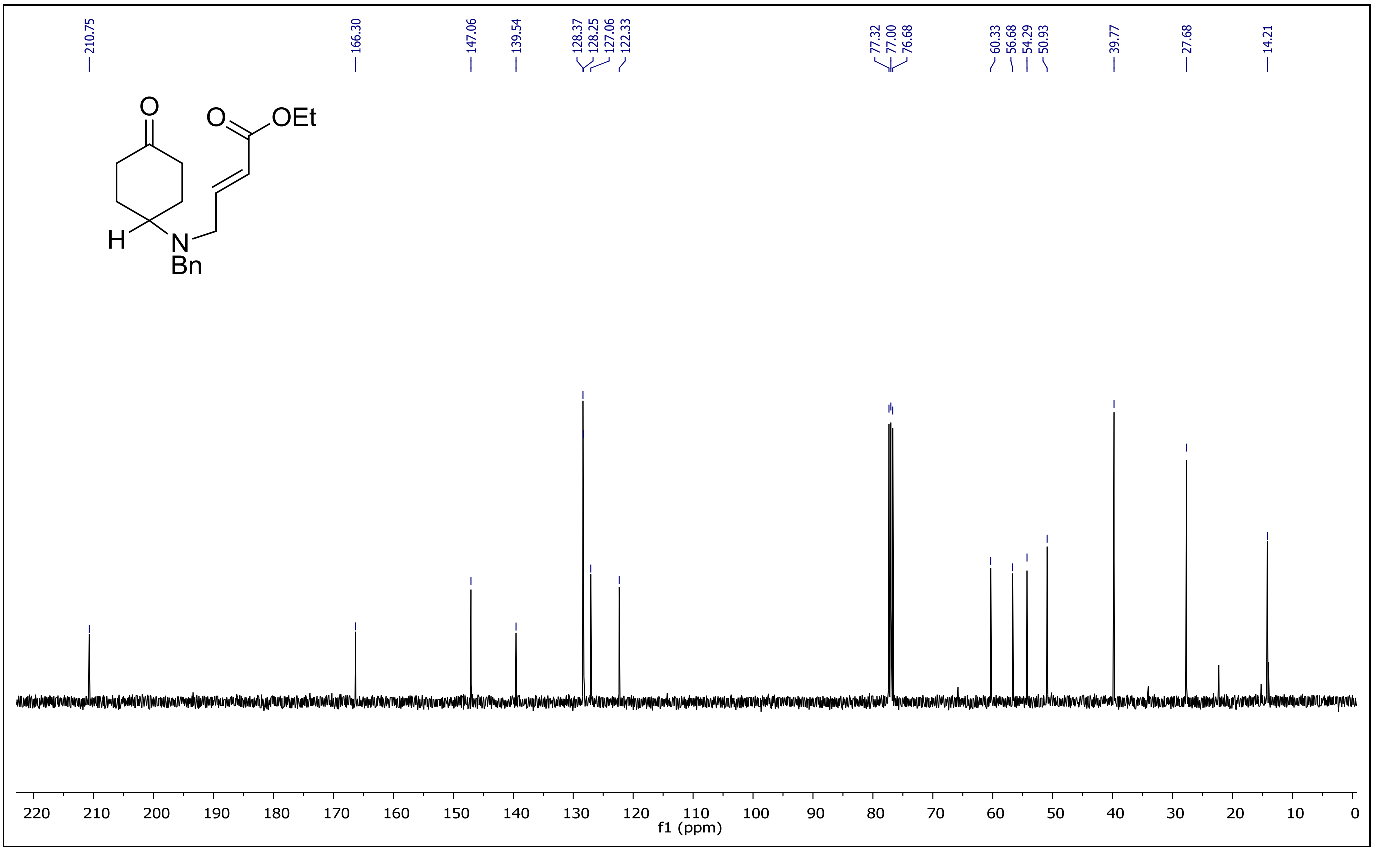

^{1}H NMR Spectrum of compound 27m

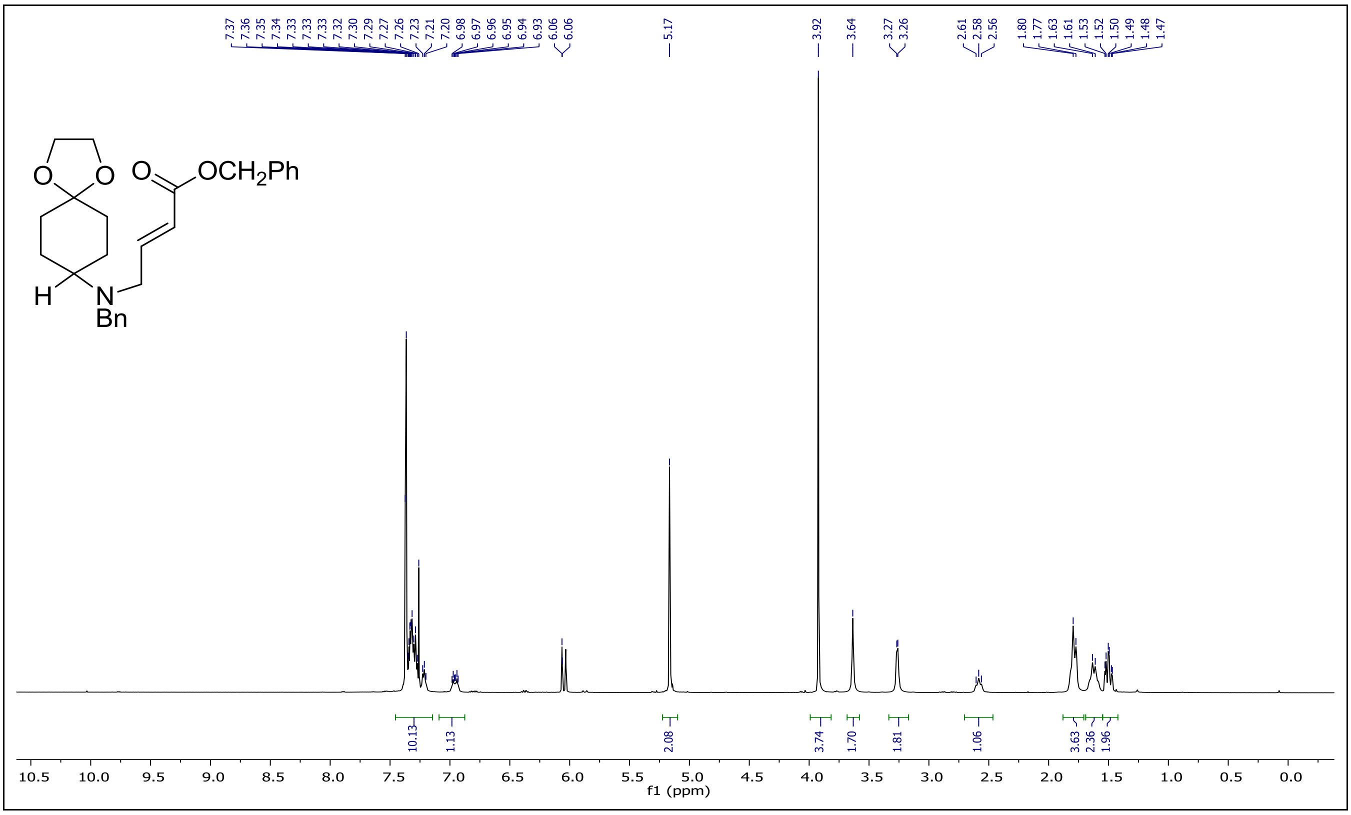

^{13}C NMR Spectrum of compound 27m

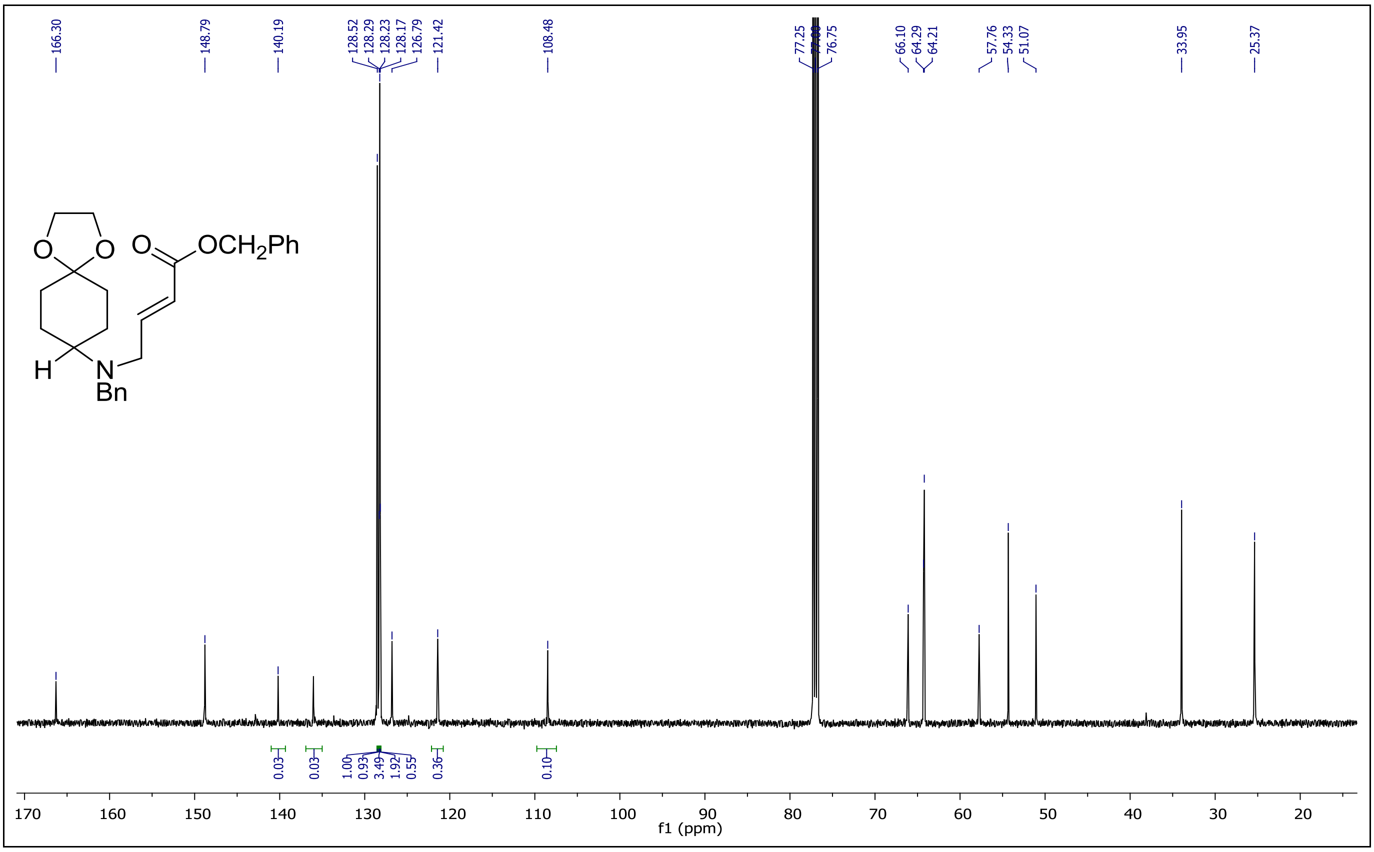

^{1}H NMR Spectrum of compound 2m

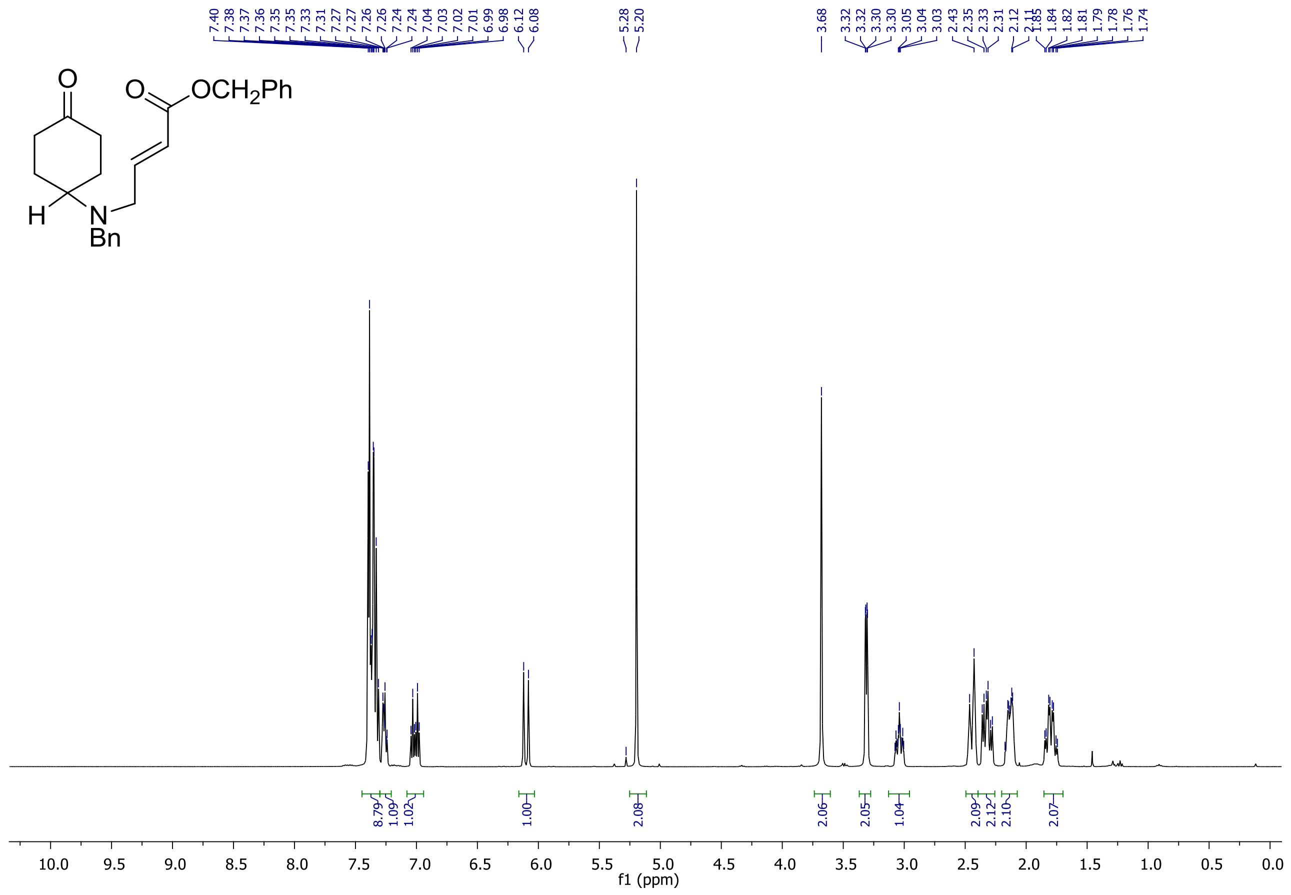

^{13}C NMR Spectrum of compound 2m

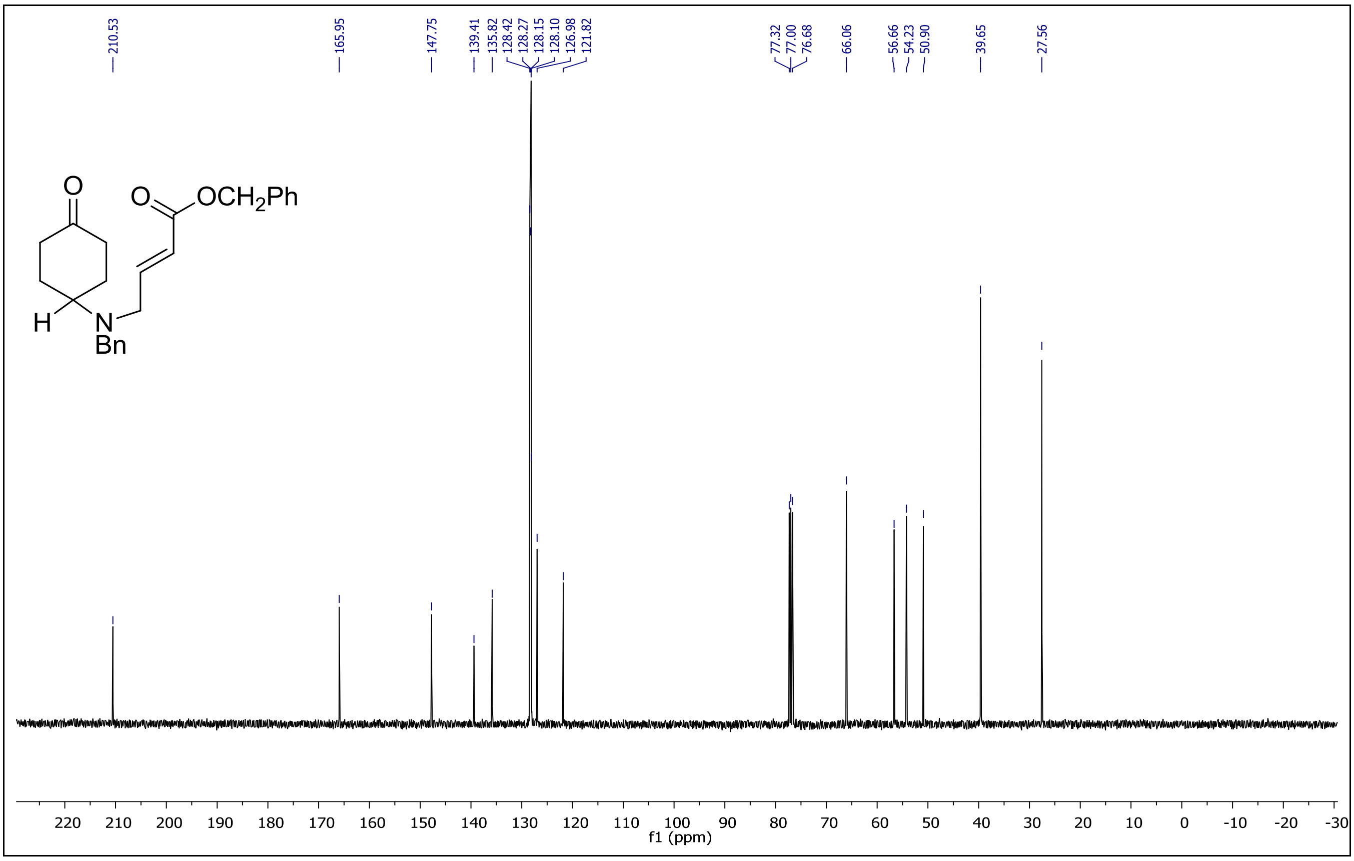

^{1}H NMR Spectrum of compound 27n

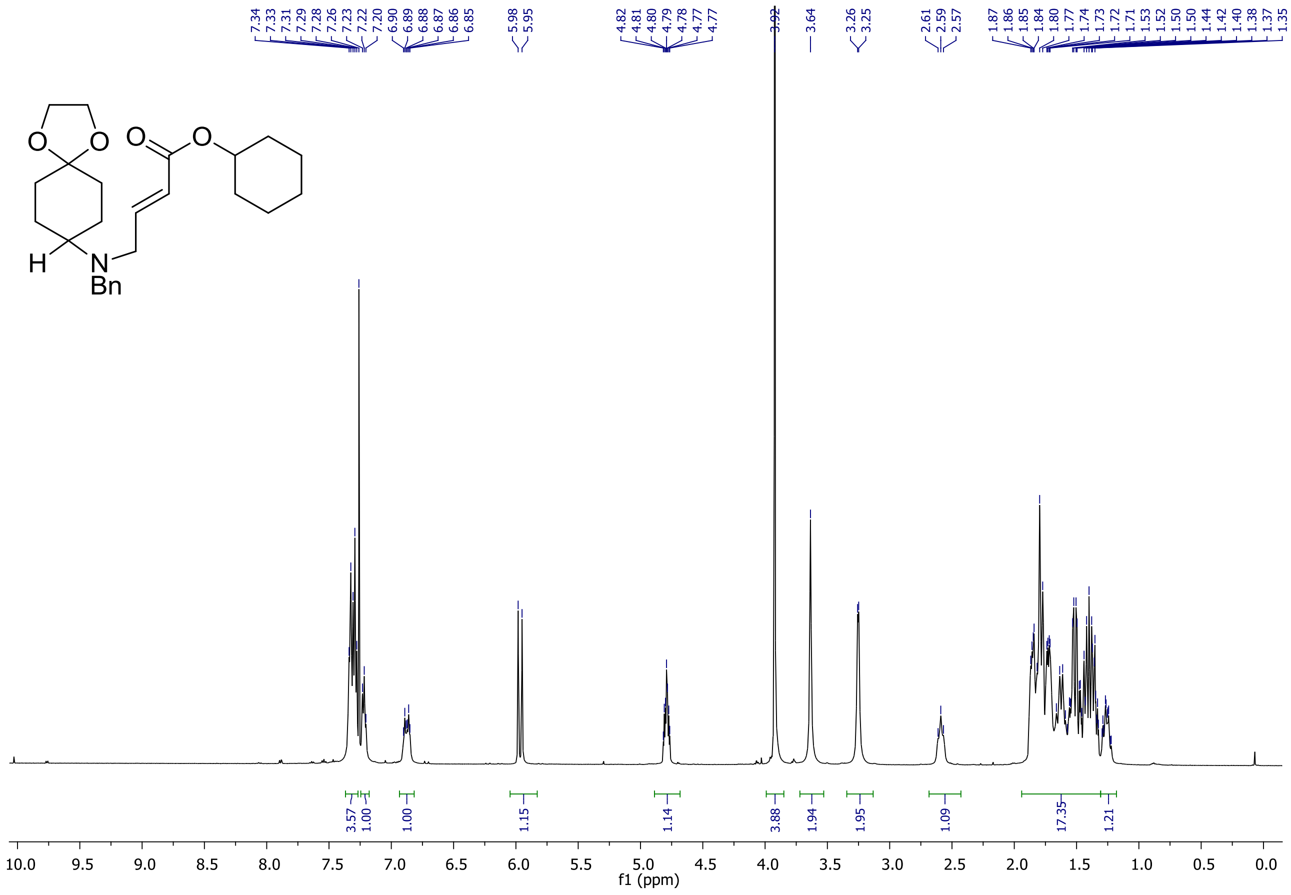

^{13}C NMR Spectrum of compound 27n

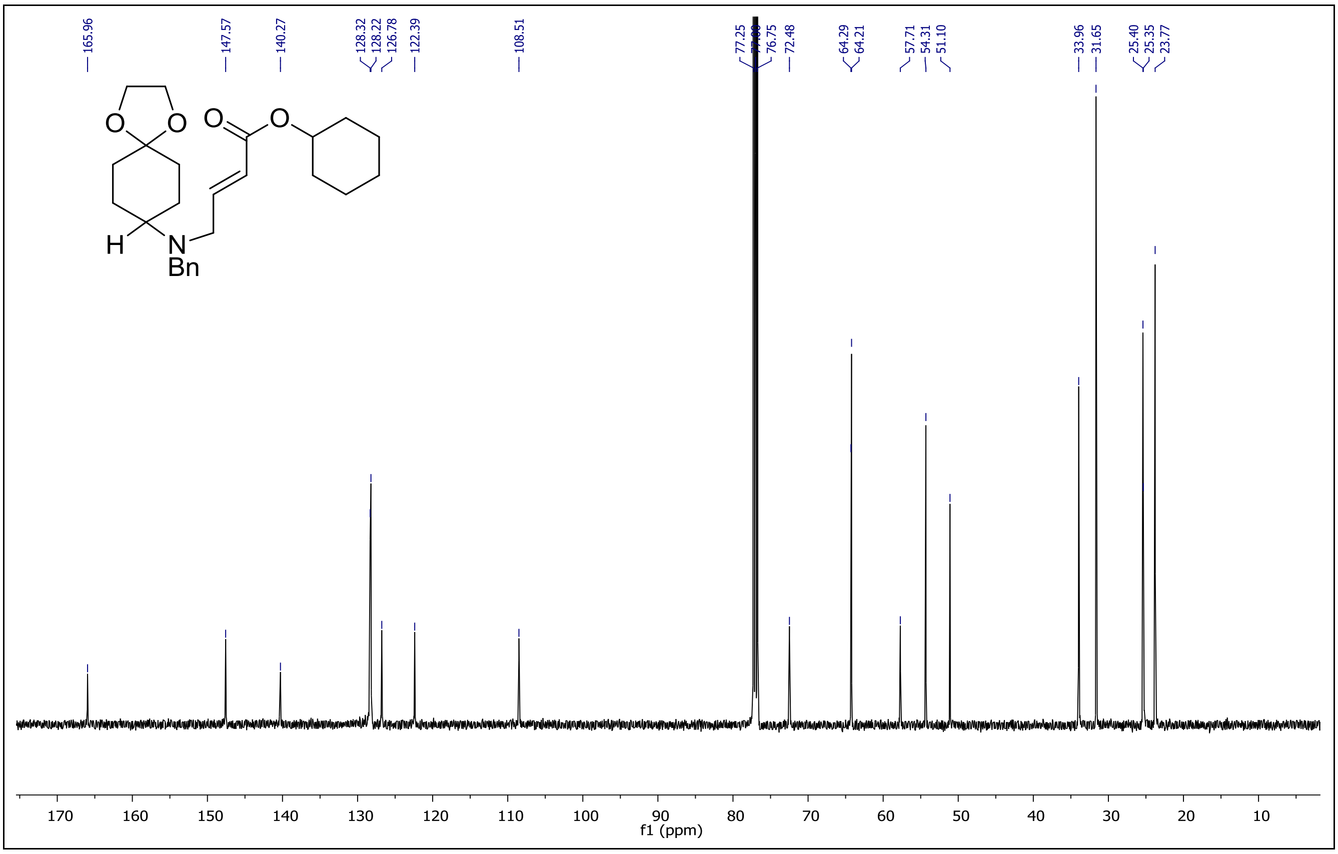

^{1}H NMR Spectrum of compound 2n

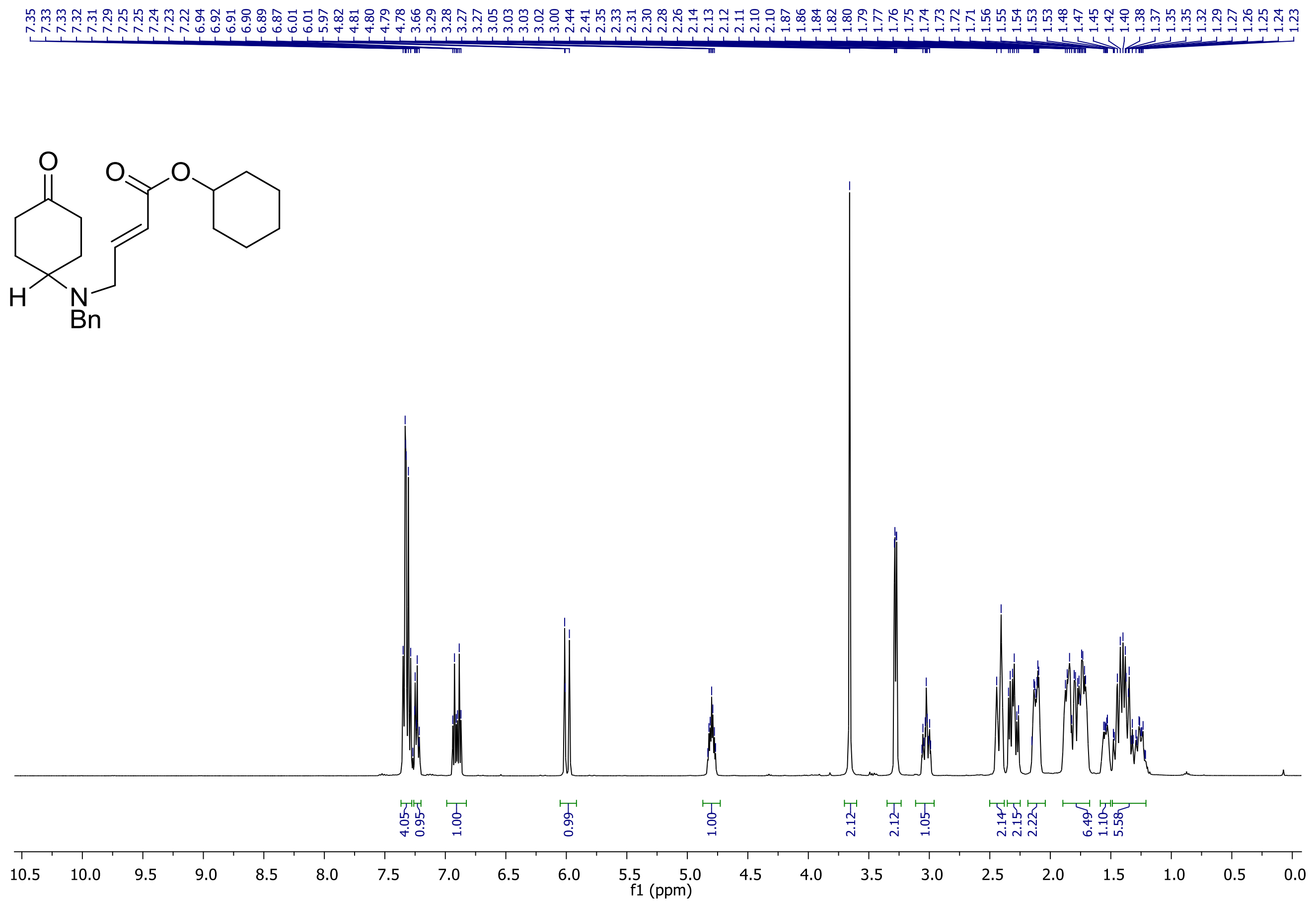

^{13}C NMR Spectrum of compound 2o

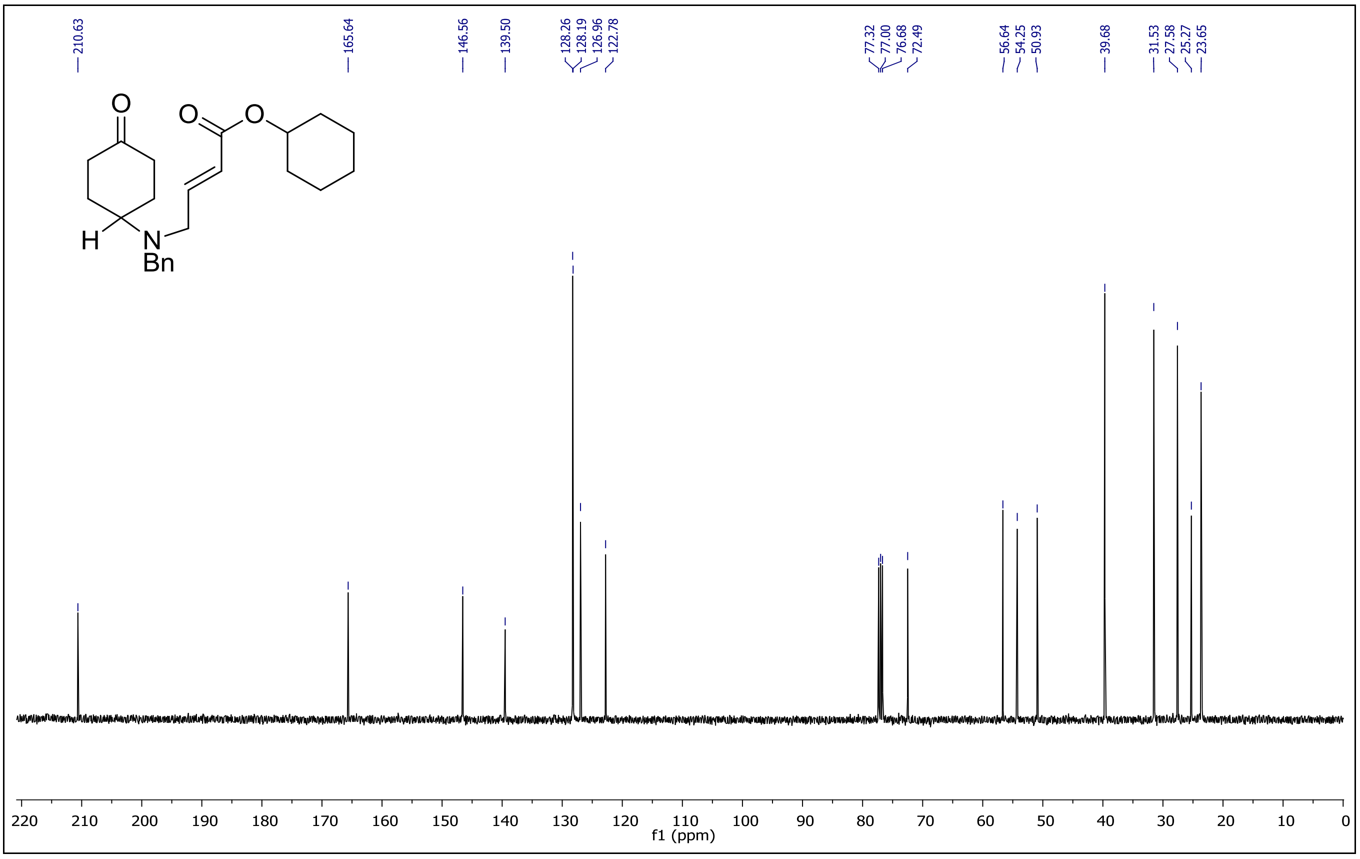

^{1}H NMR Spectrum of compound 27o

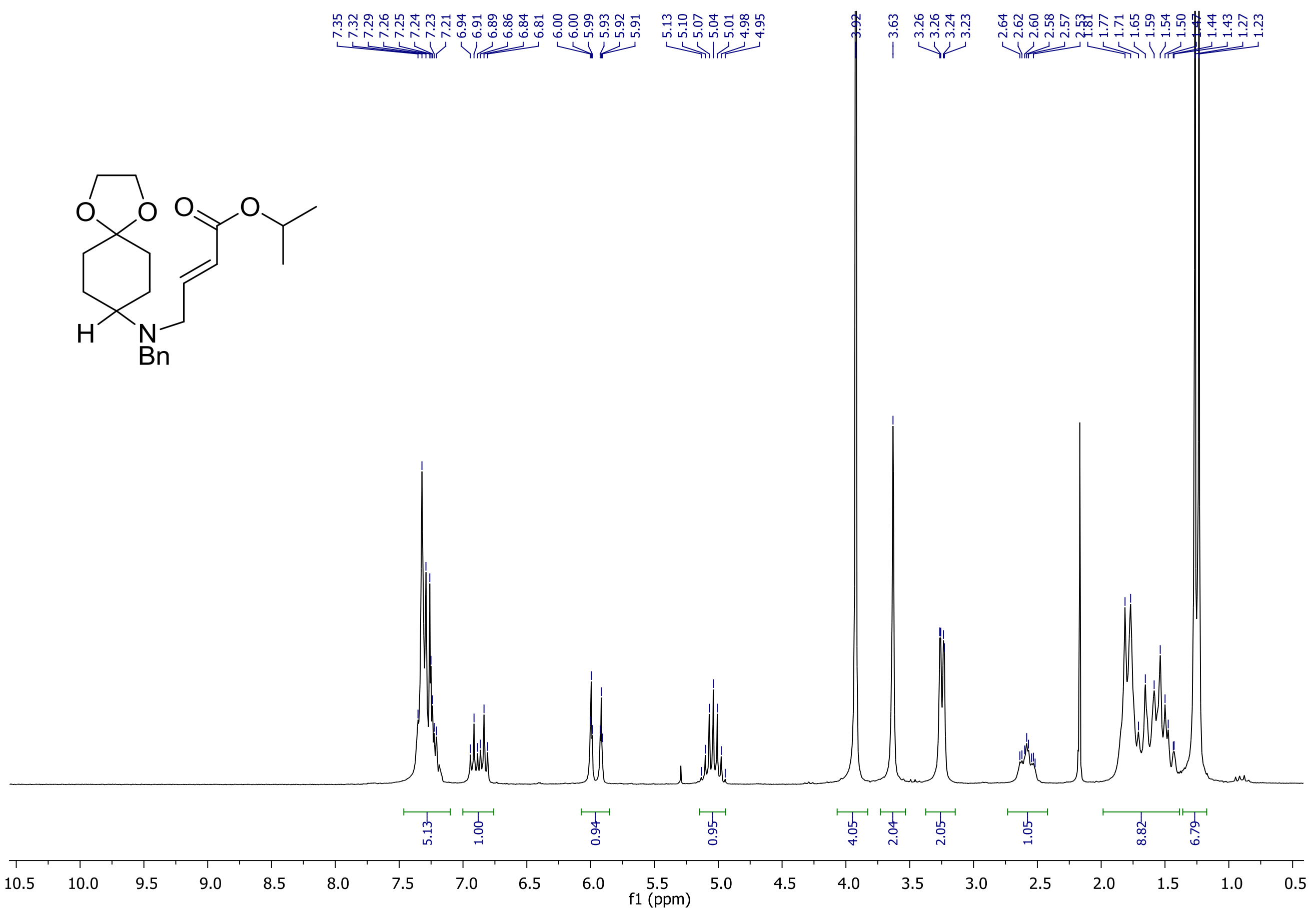

^{13}C NMR Spectrum of compound 27o

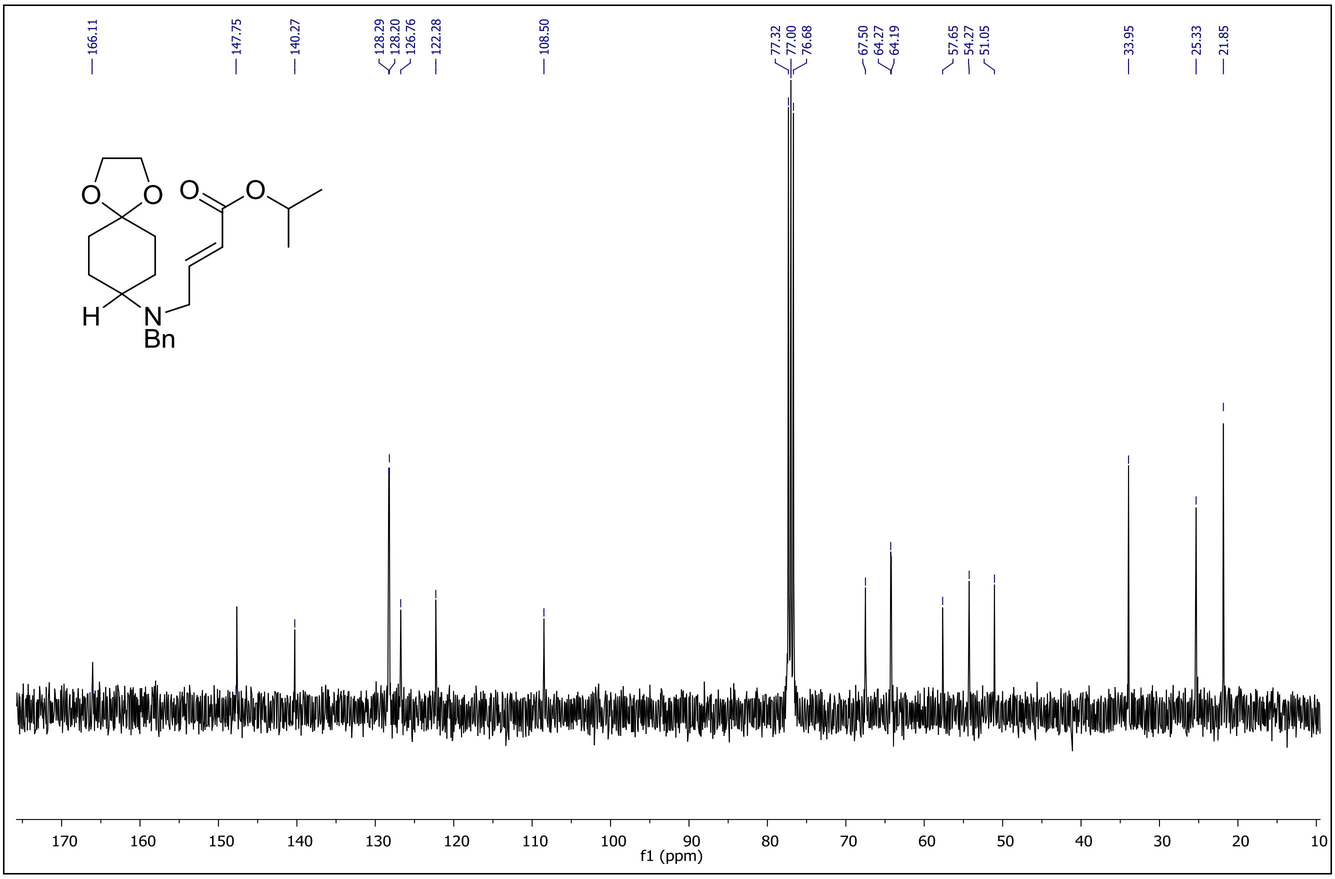

^{1}H NMR Spectrum of compound 2o

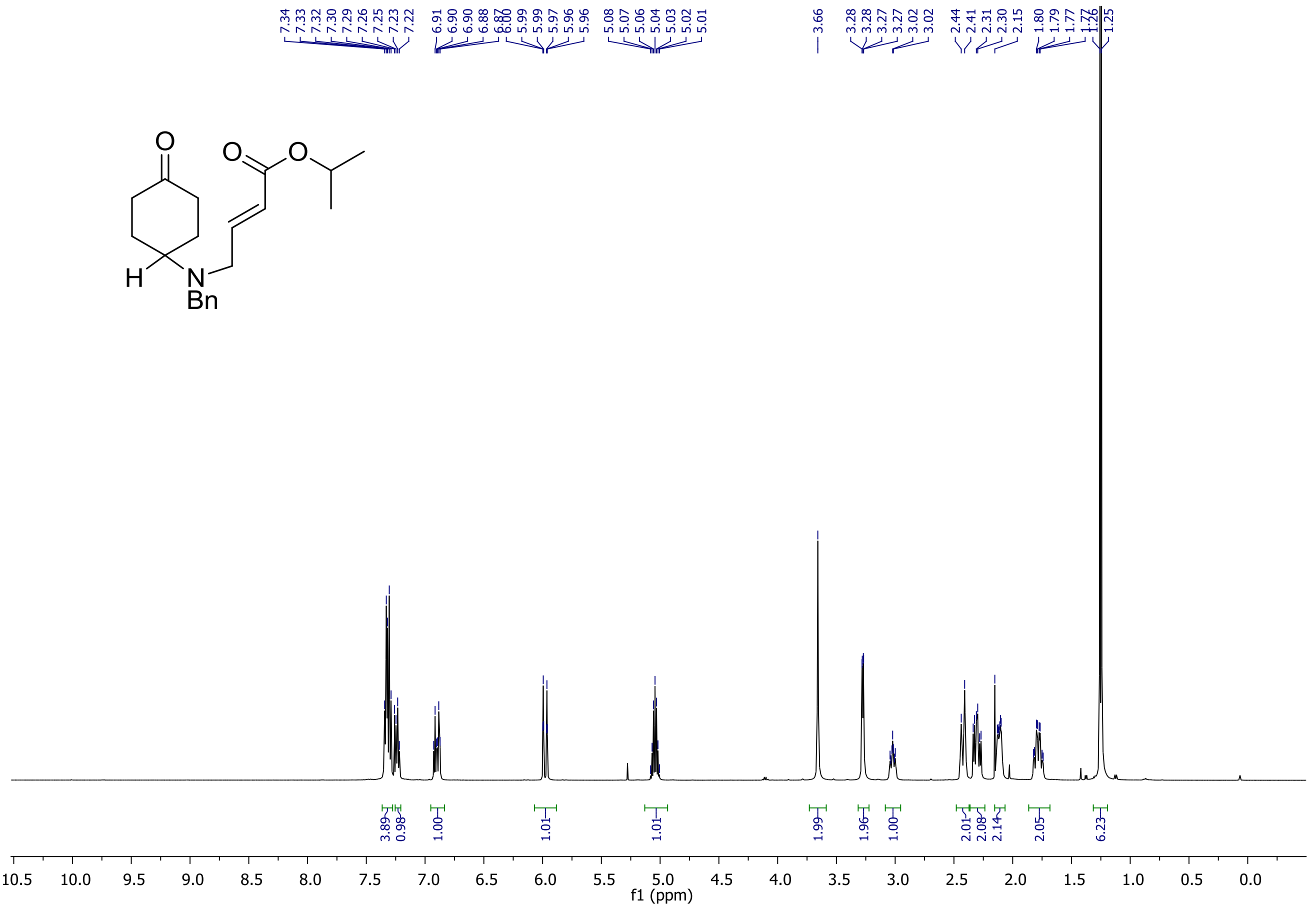

^{13}C NMR Spectrum of compound 2o

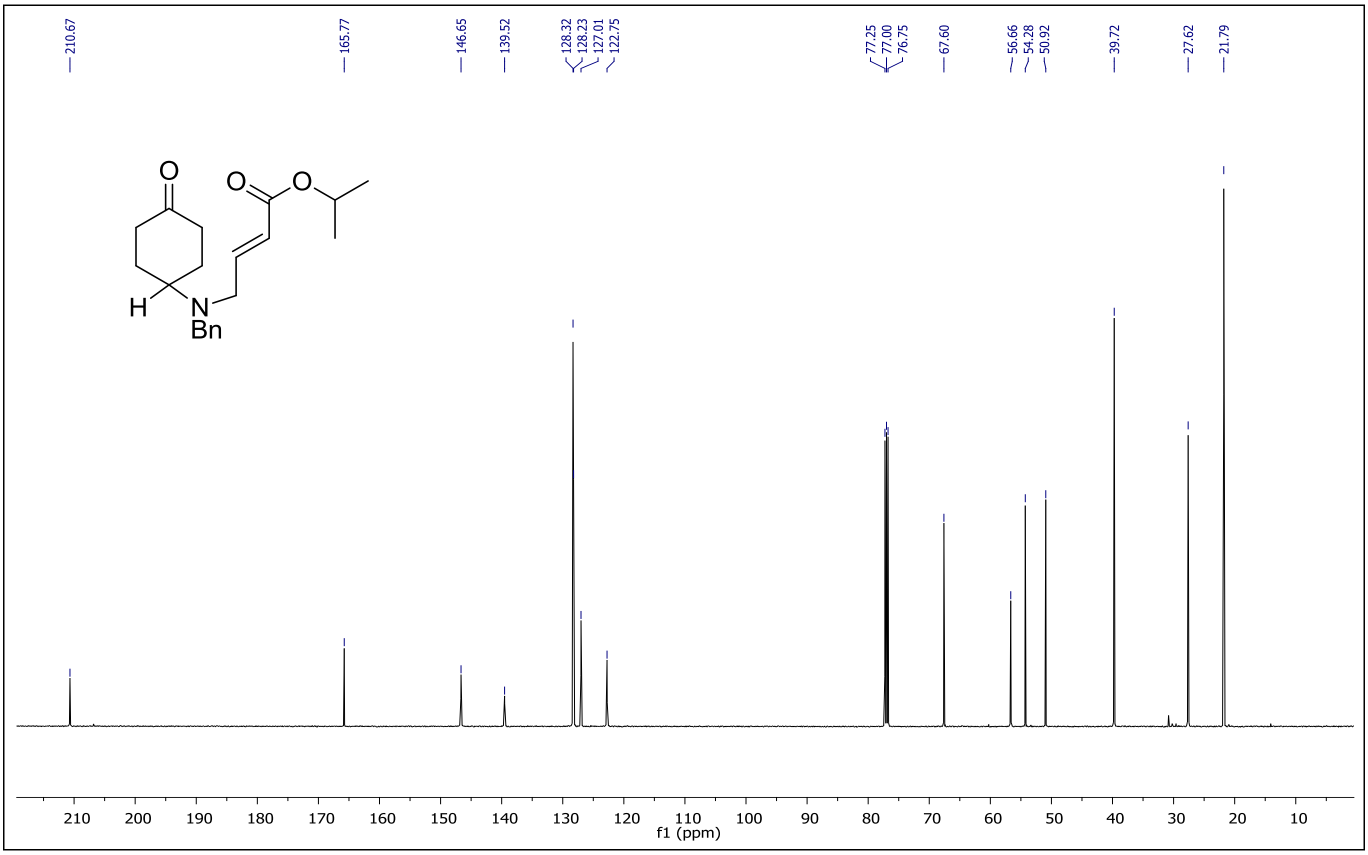

^{1}H NMR Spectrum of compound 27p

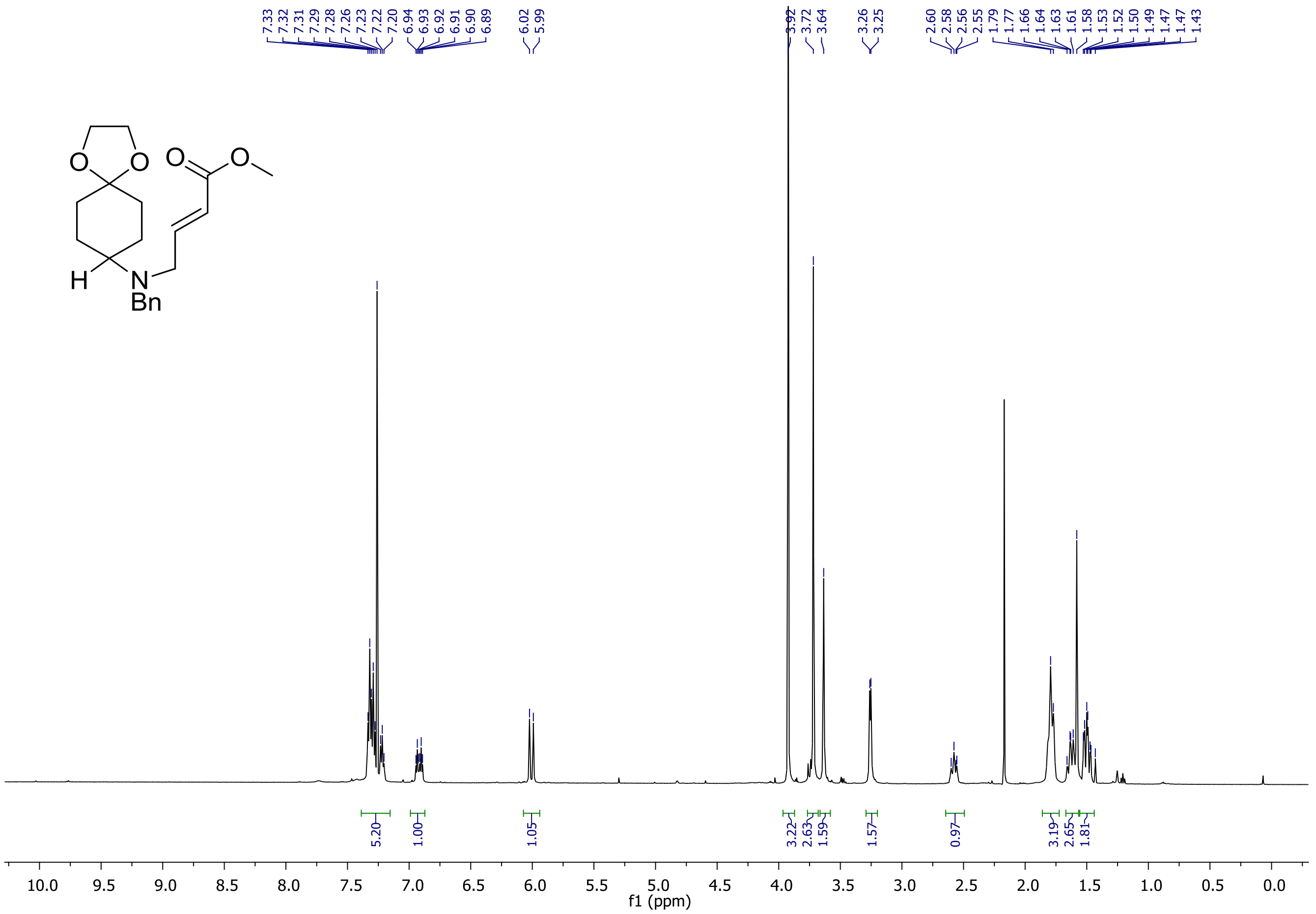

^{13}C NMR Spectrum of compound 27p

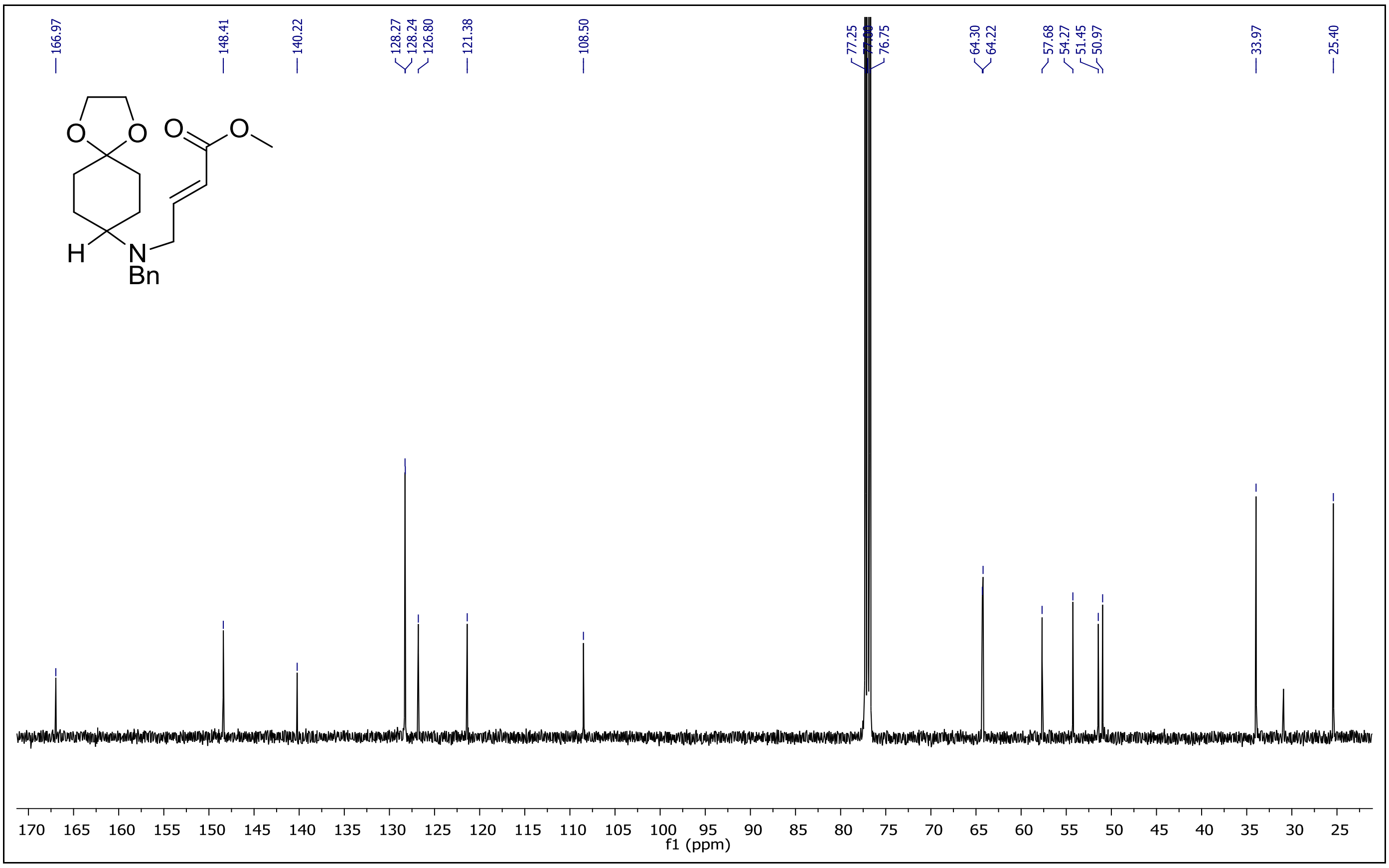

^{1}H NMR Spectrum of compound 2p

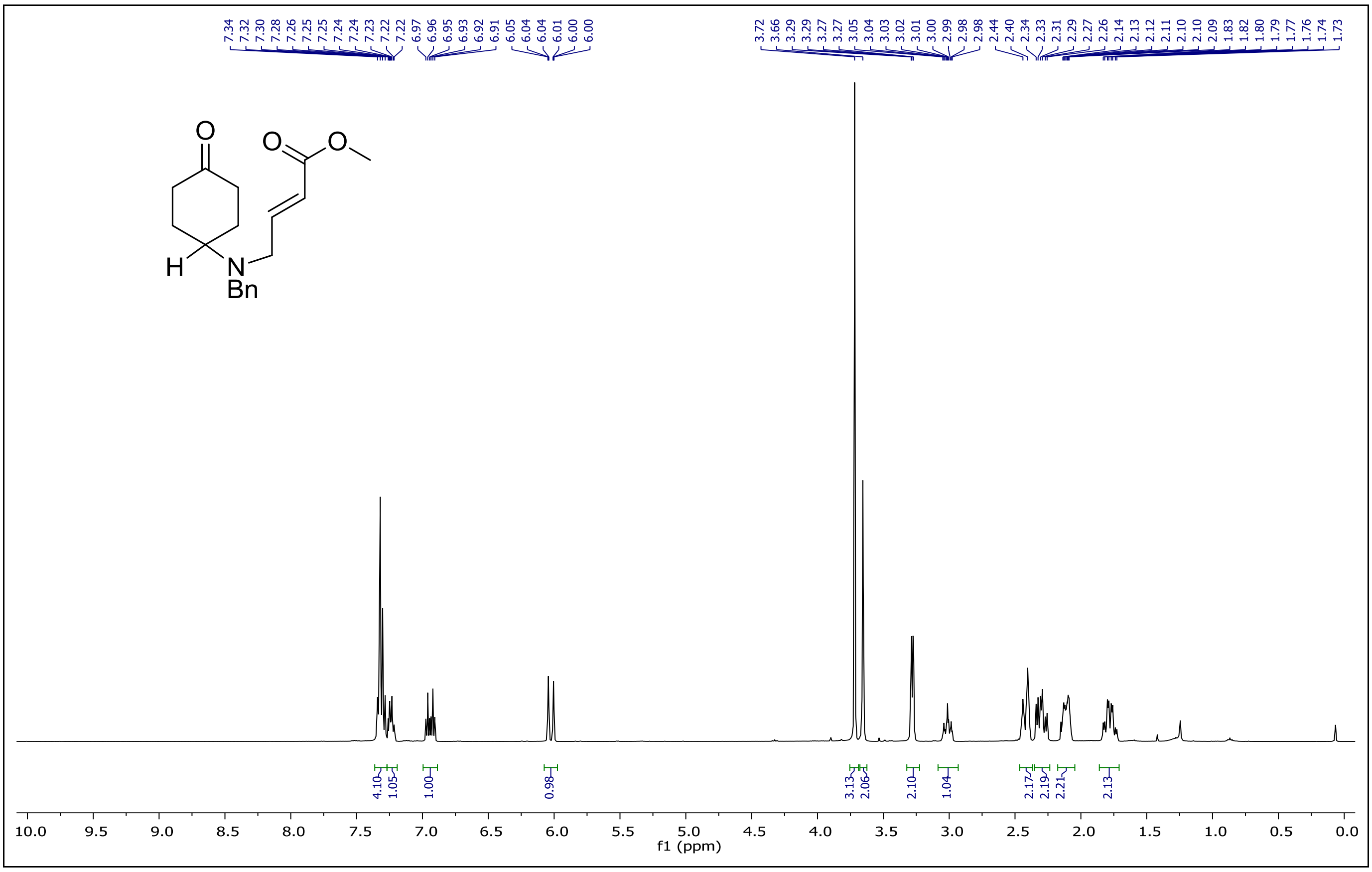

^{13}C NMR Spectrum of compound 2p

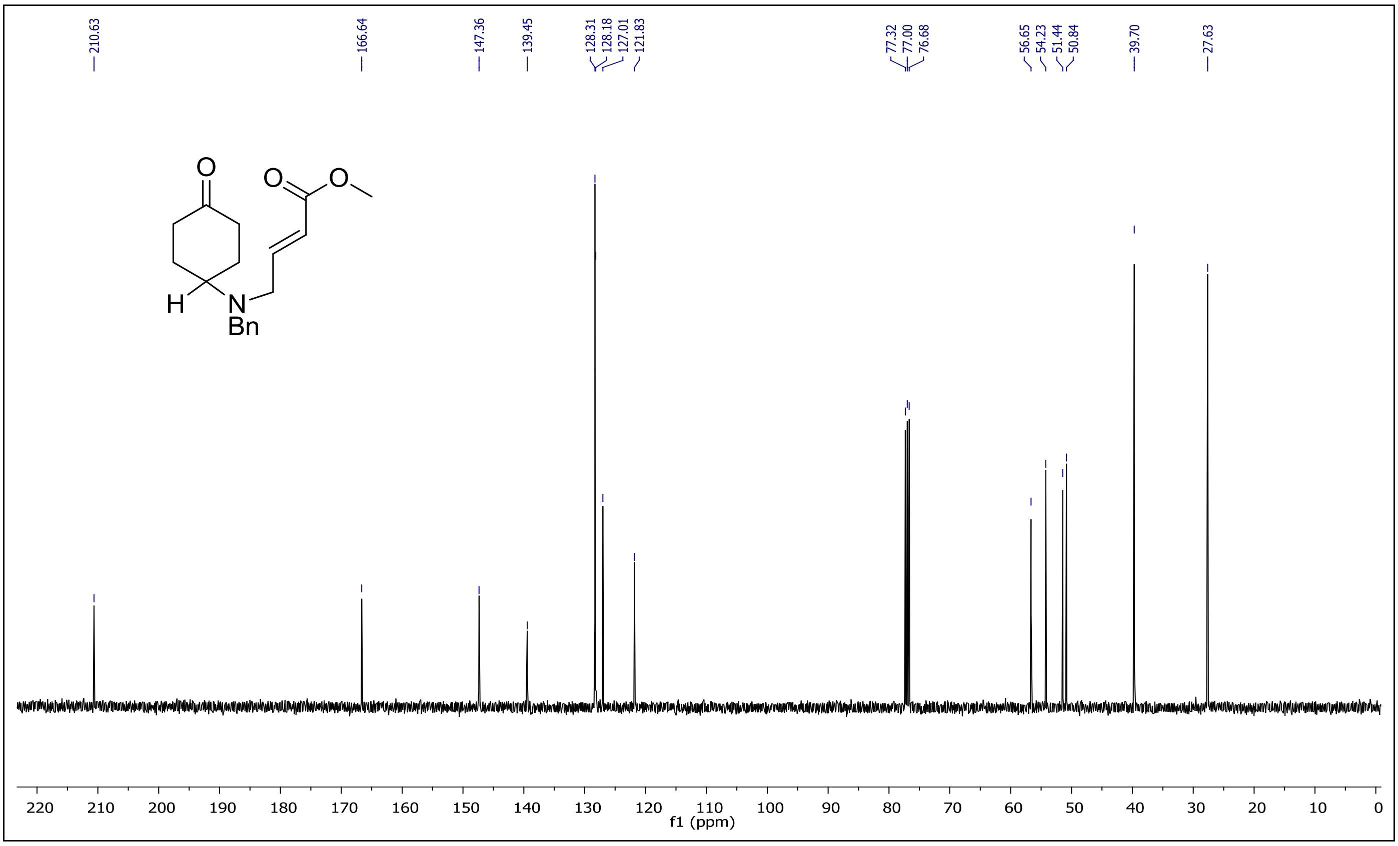

^{1}H NMR Spectrum of compound 27q

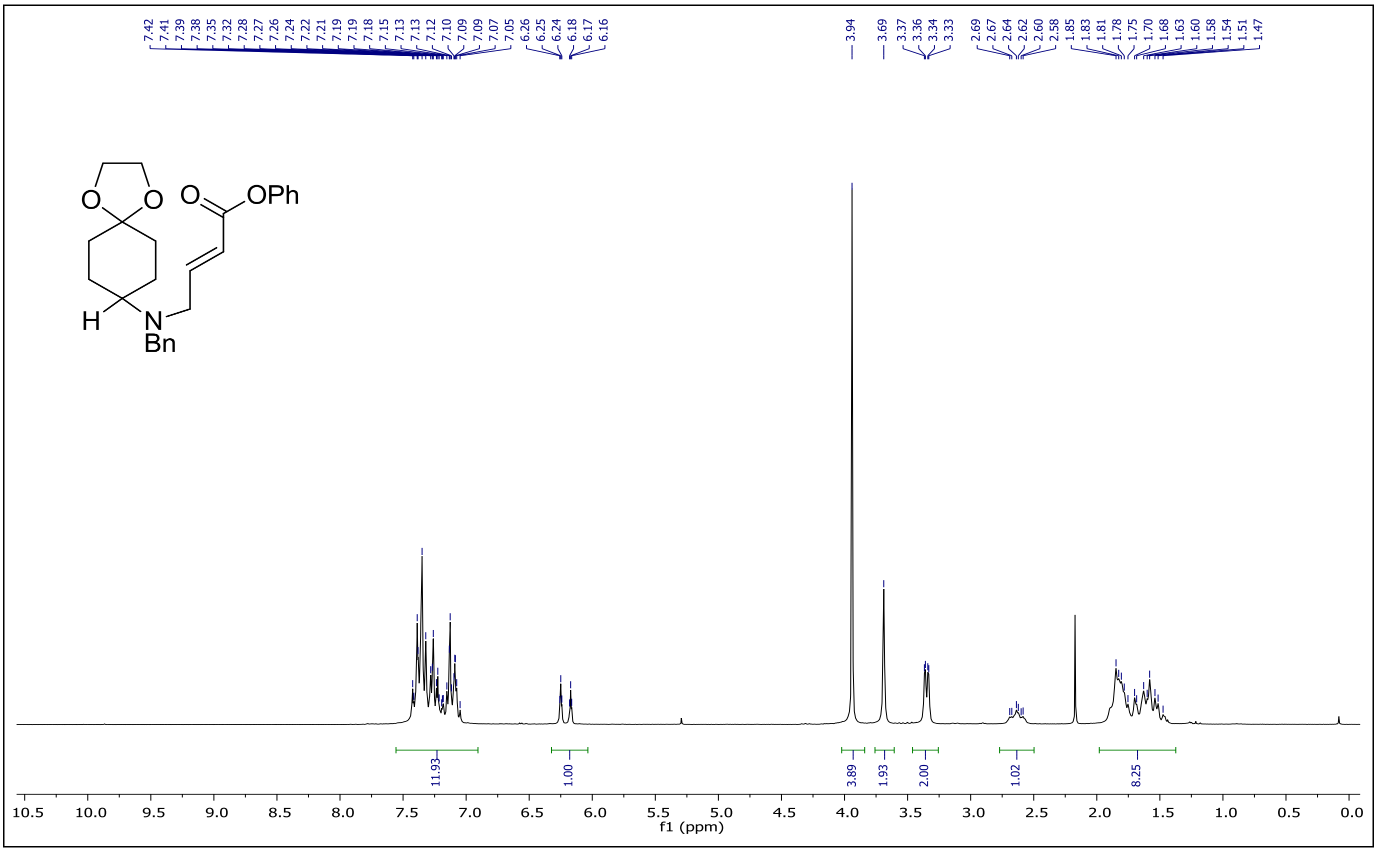

^{13}C NMR Spectrum of compound 27q

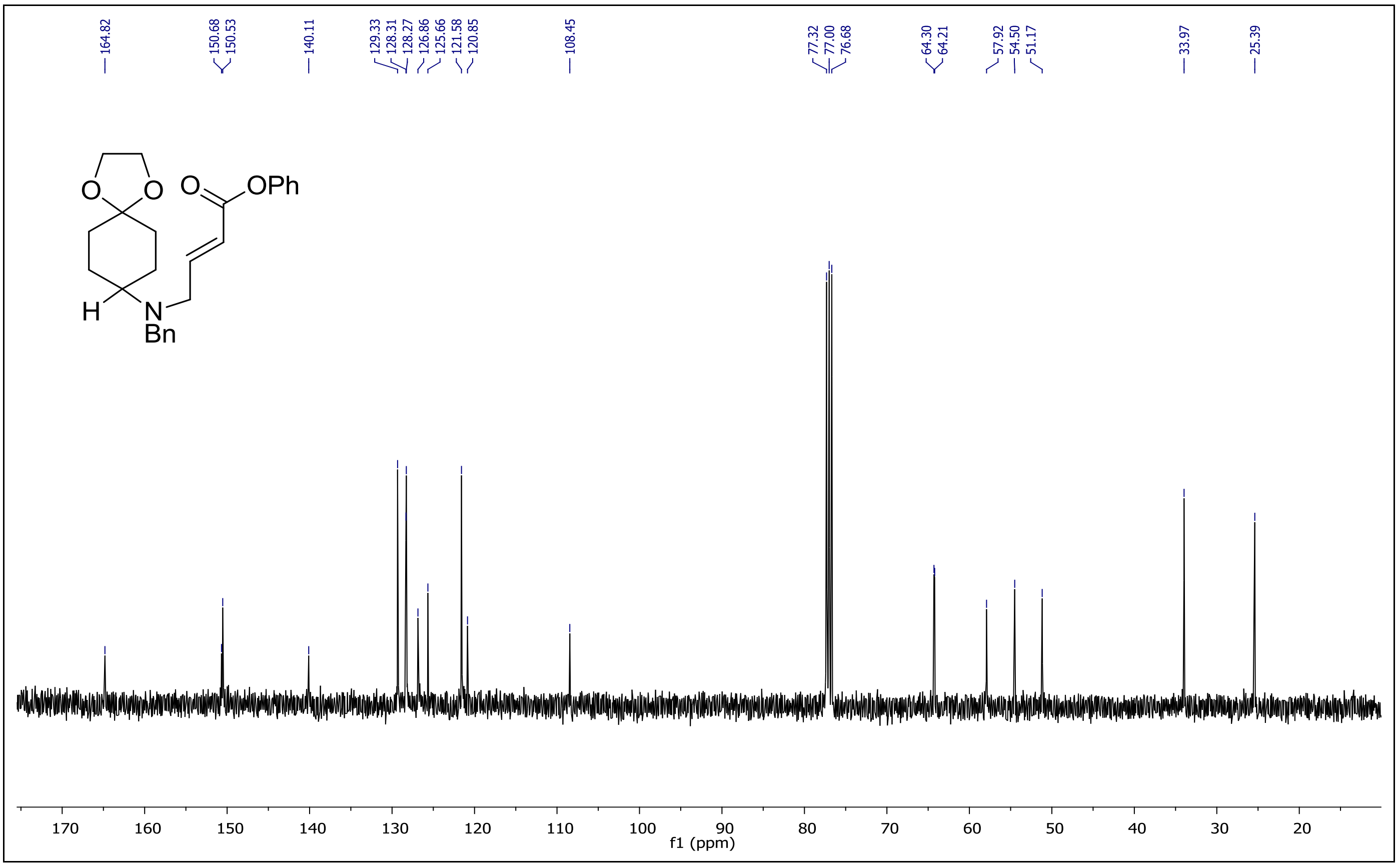

^{1}H NMR Spectrum of compound 2q

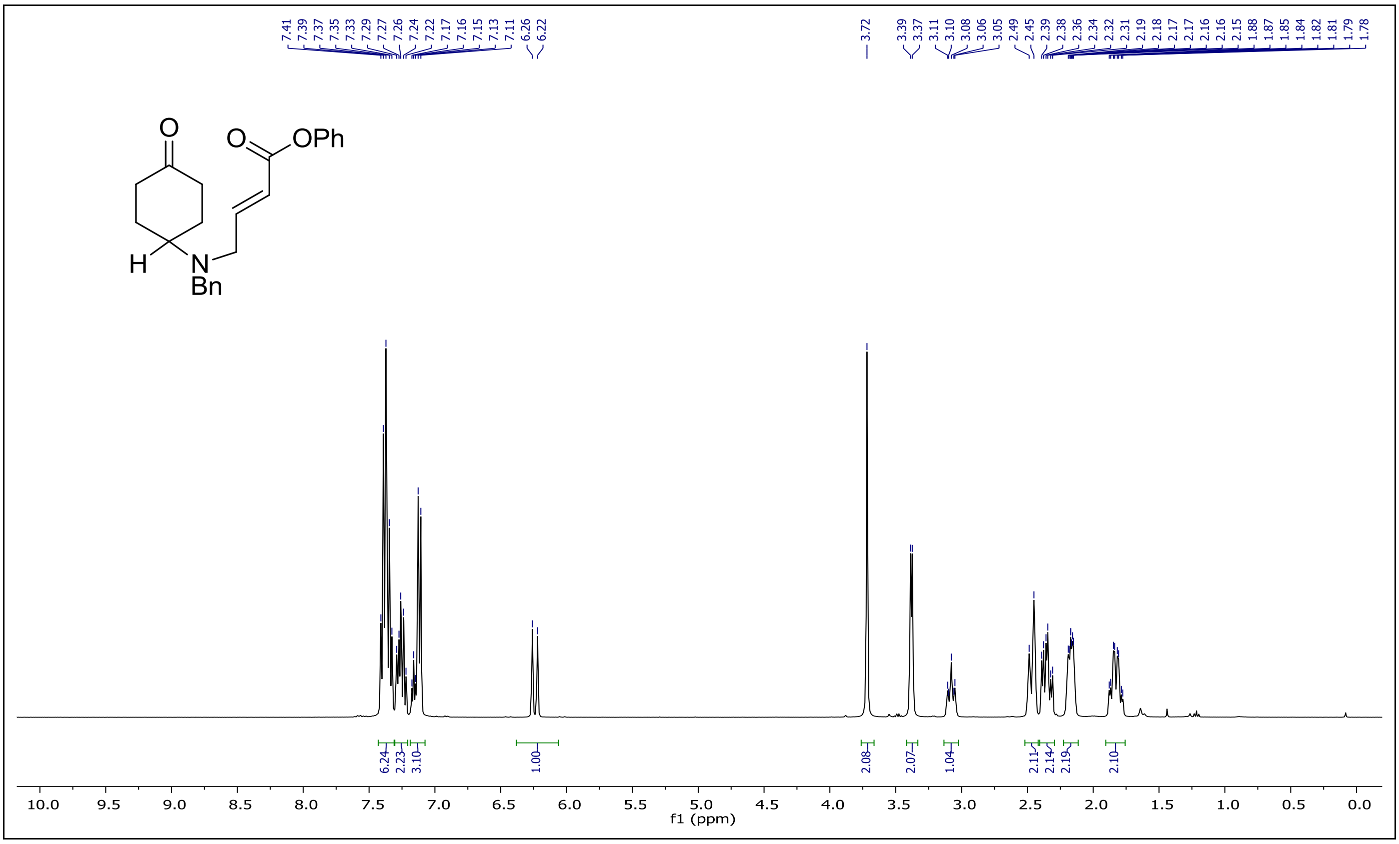

^{13}C NMR Spectrum of compound 2q

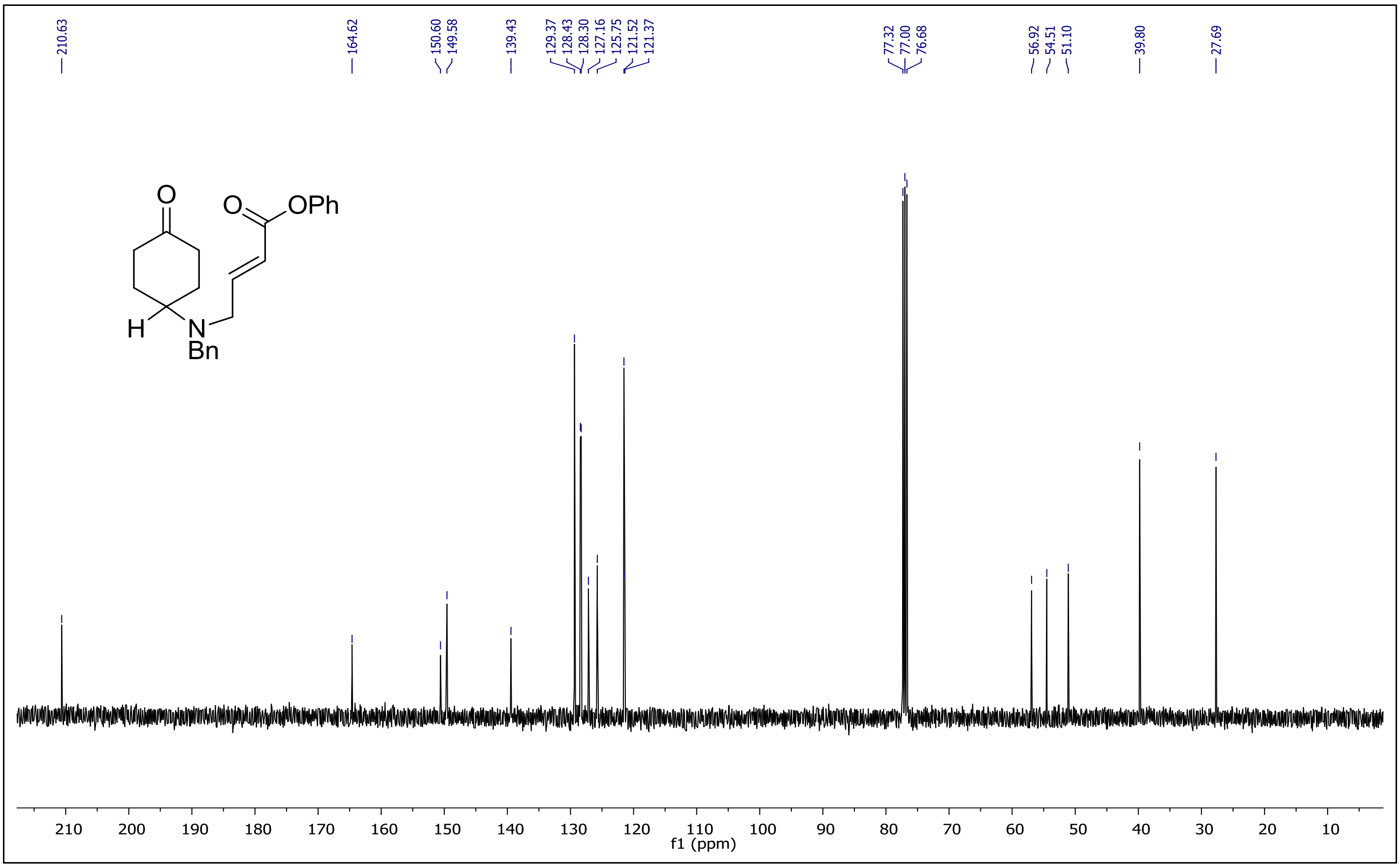

^{1}H NMR Spectrum of compound 28

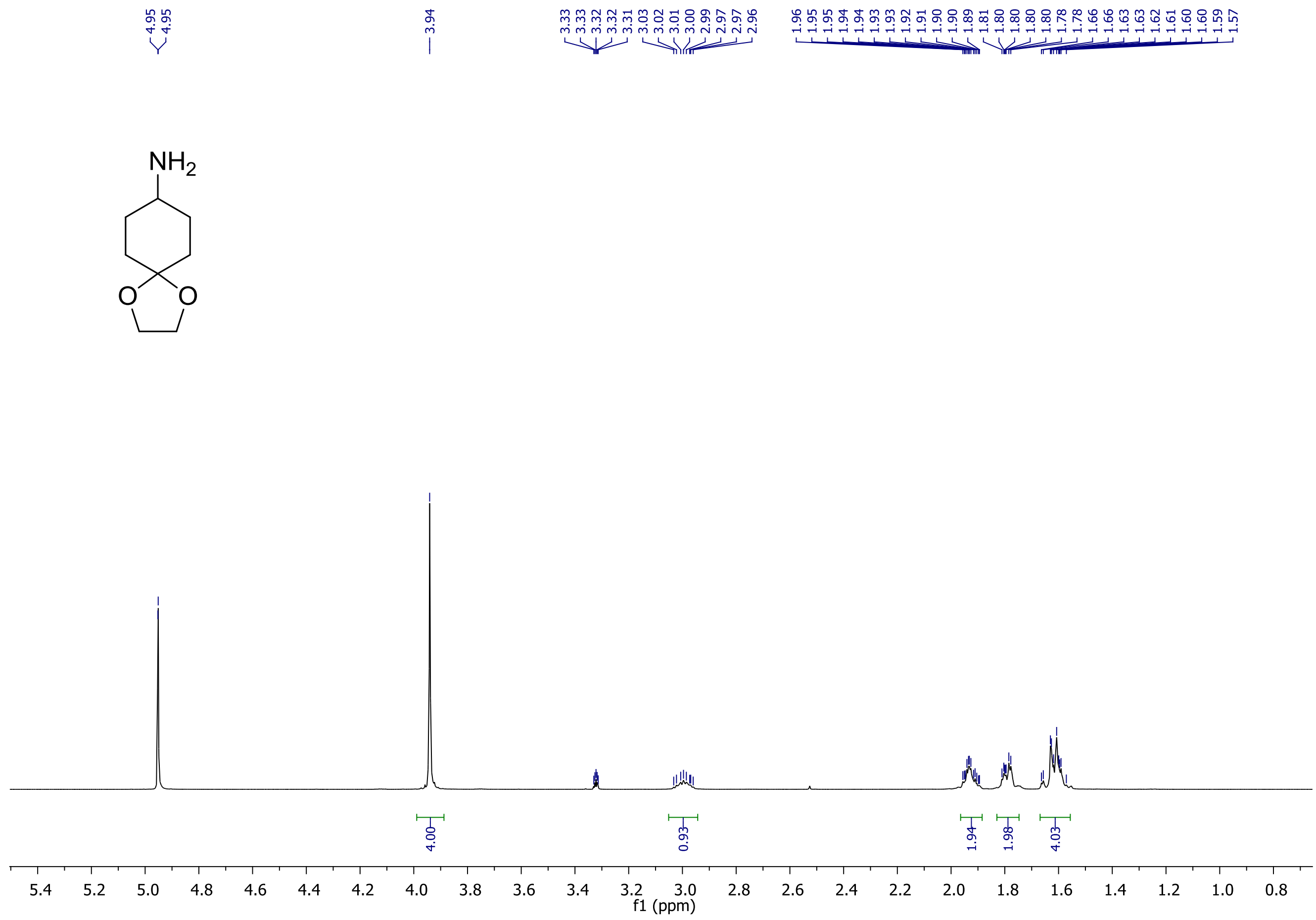

^{13}C NMR Spectrum of compound 28

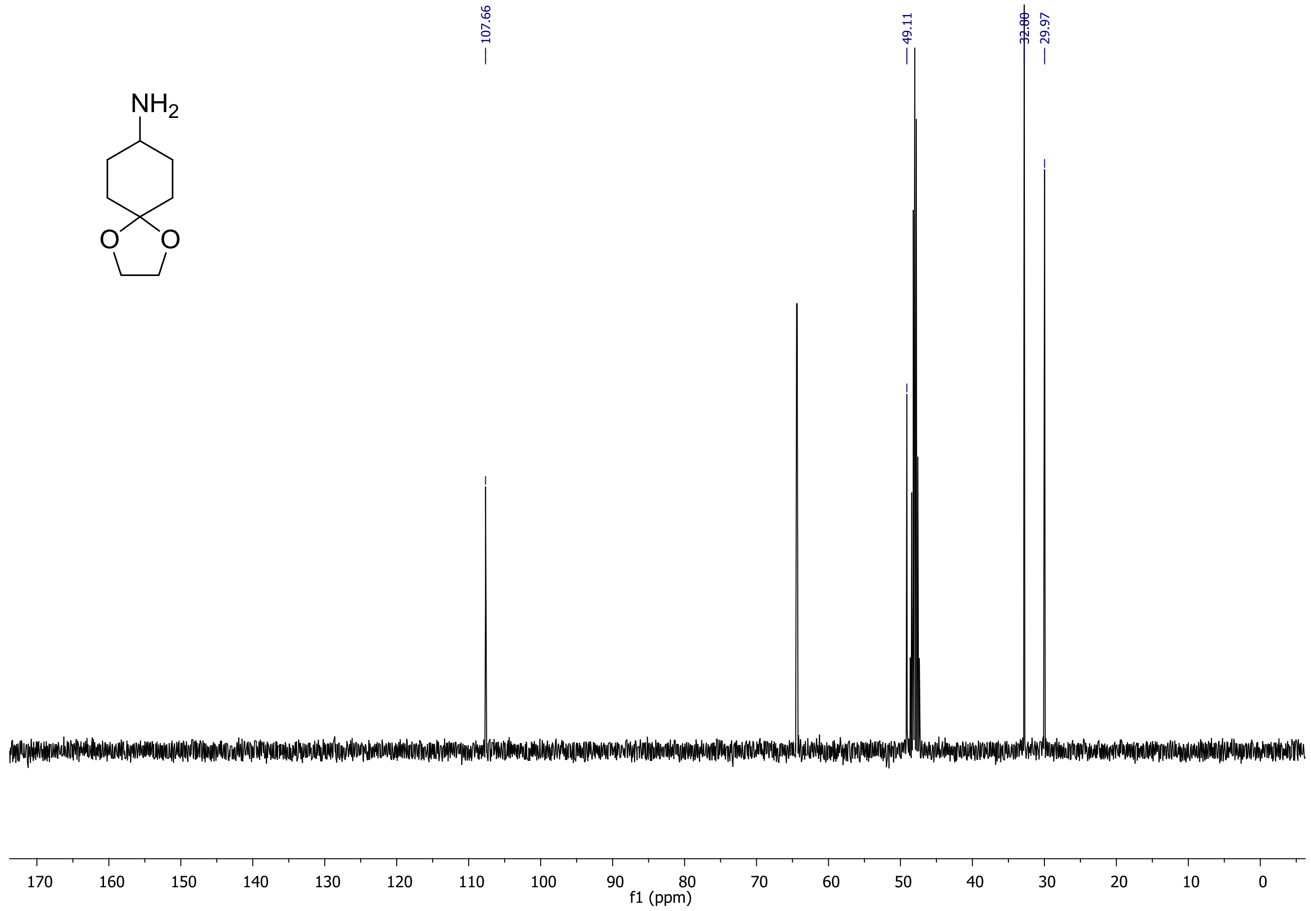

^{1}H NMR Spectrum of compound 29

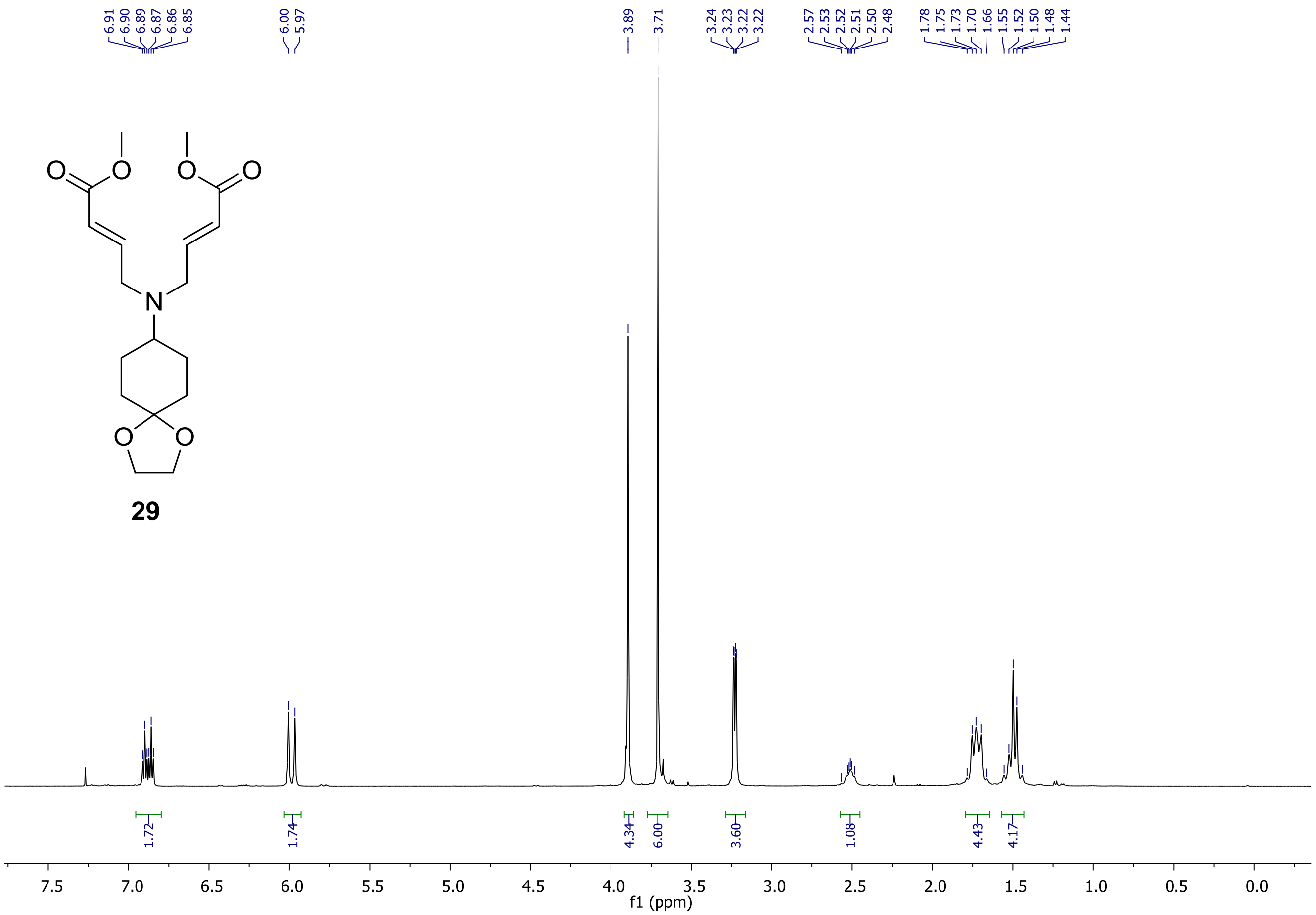

^{13}C NMR Spectrum of compound 29

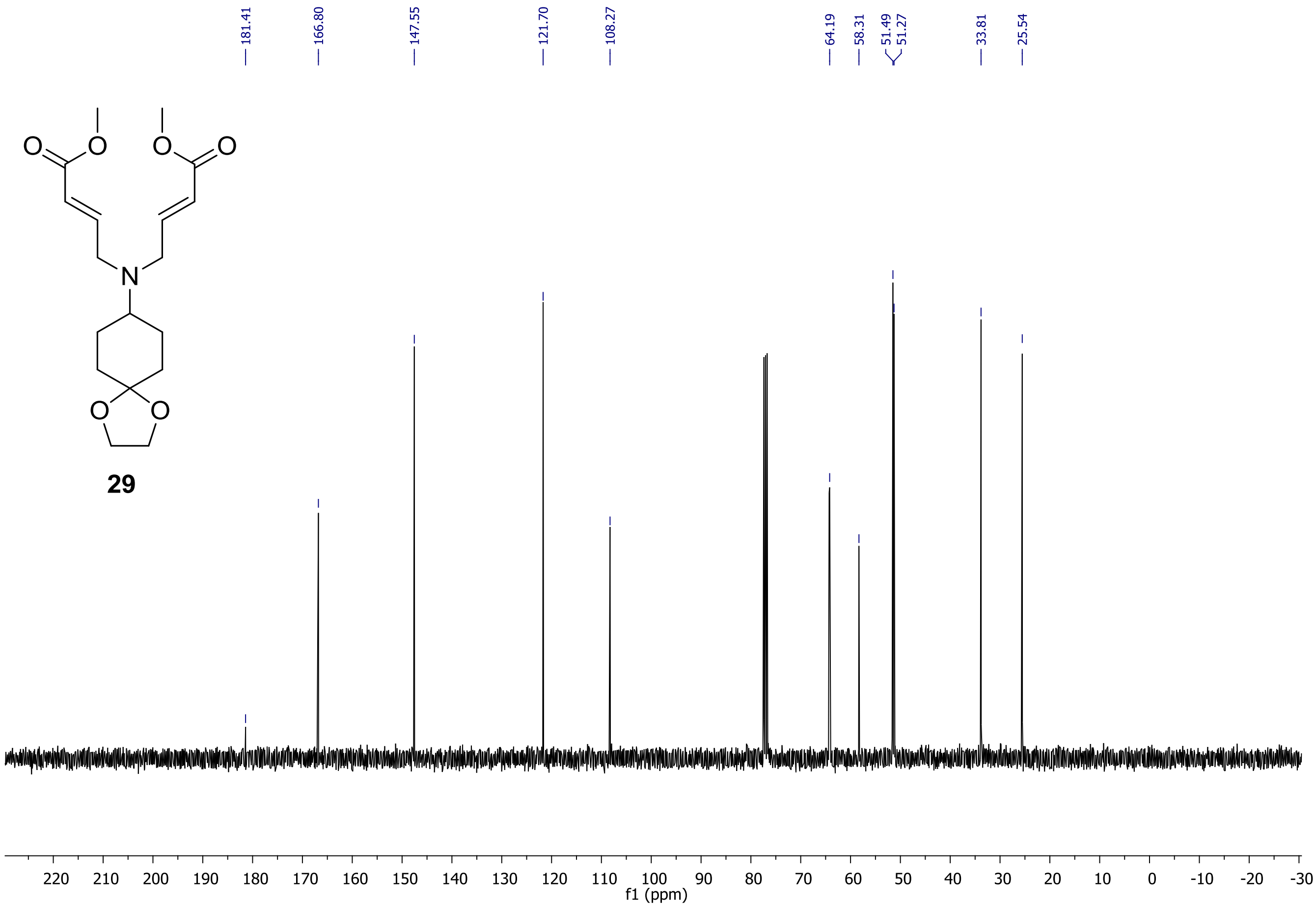

^{1}H NMR Spectrum of compound 30

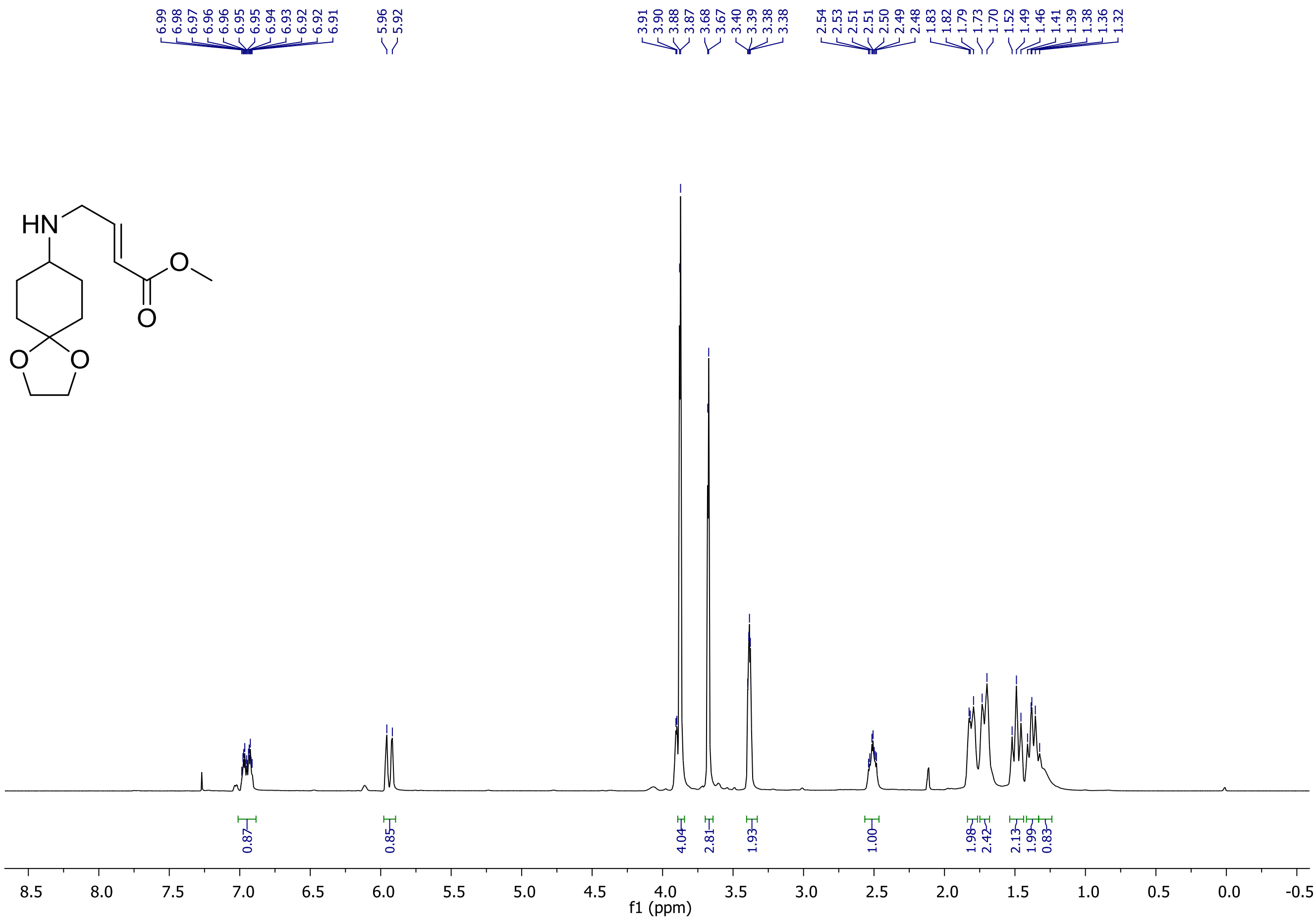

^{13}C NMR Spectrum of compound 30

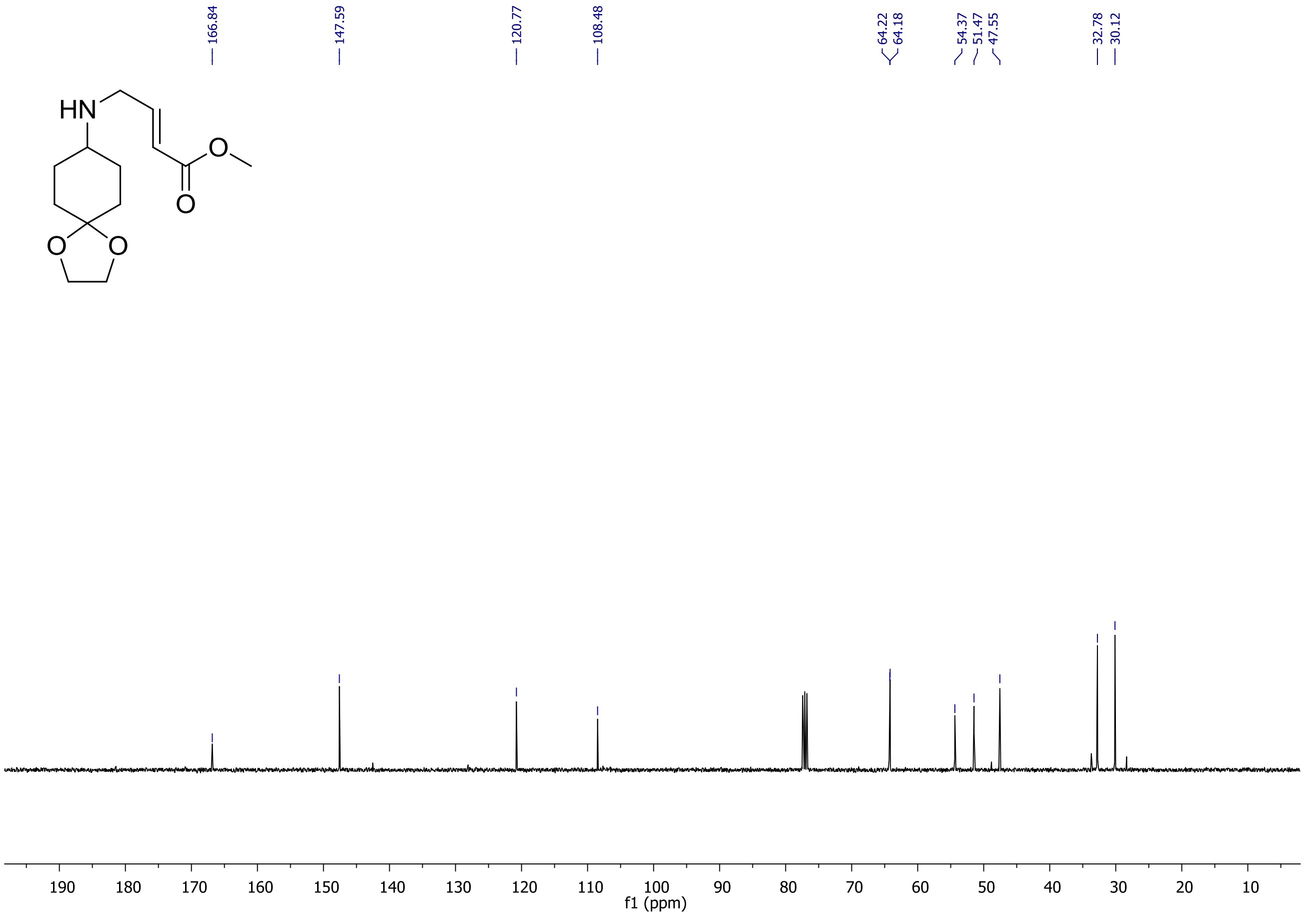

¹H NMR Spectrum of compound 31

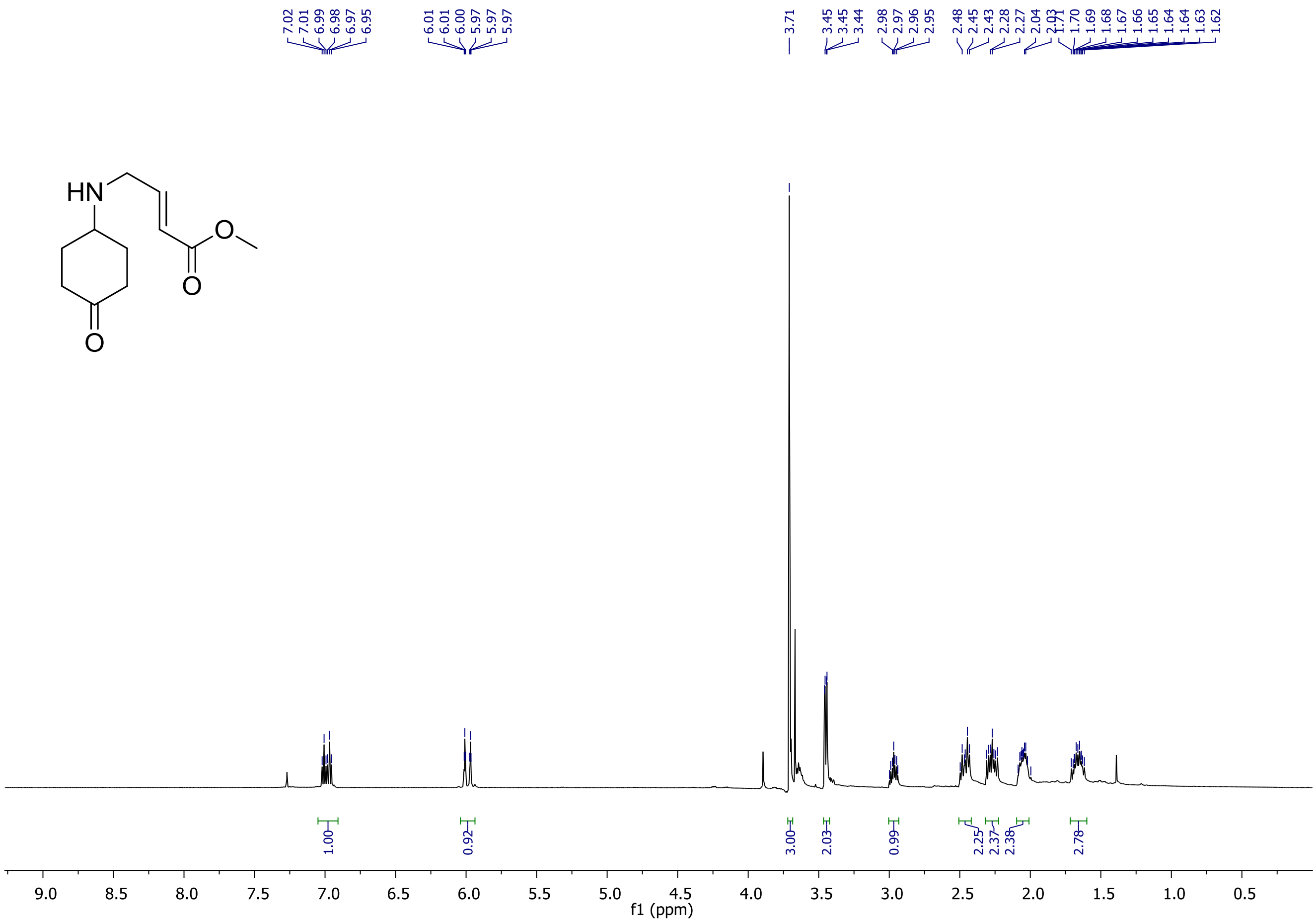

¹³C NMR Spectrum of compound 31

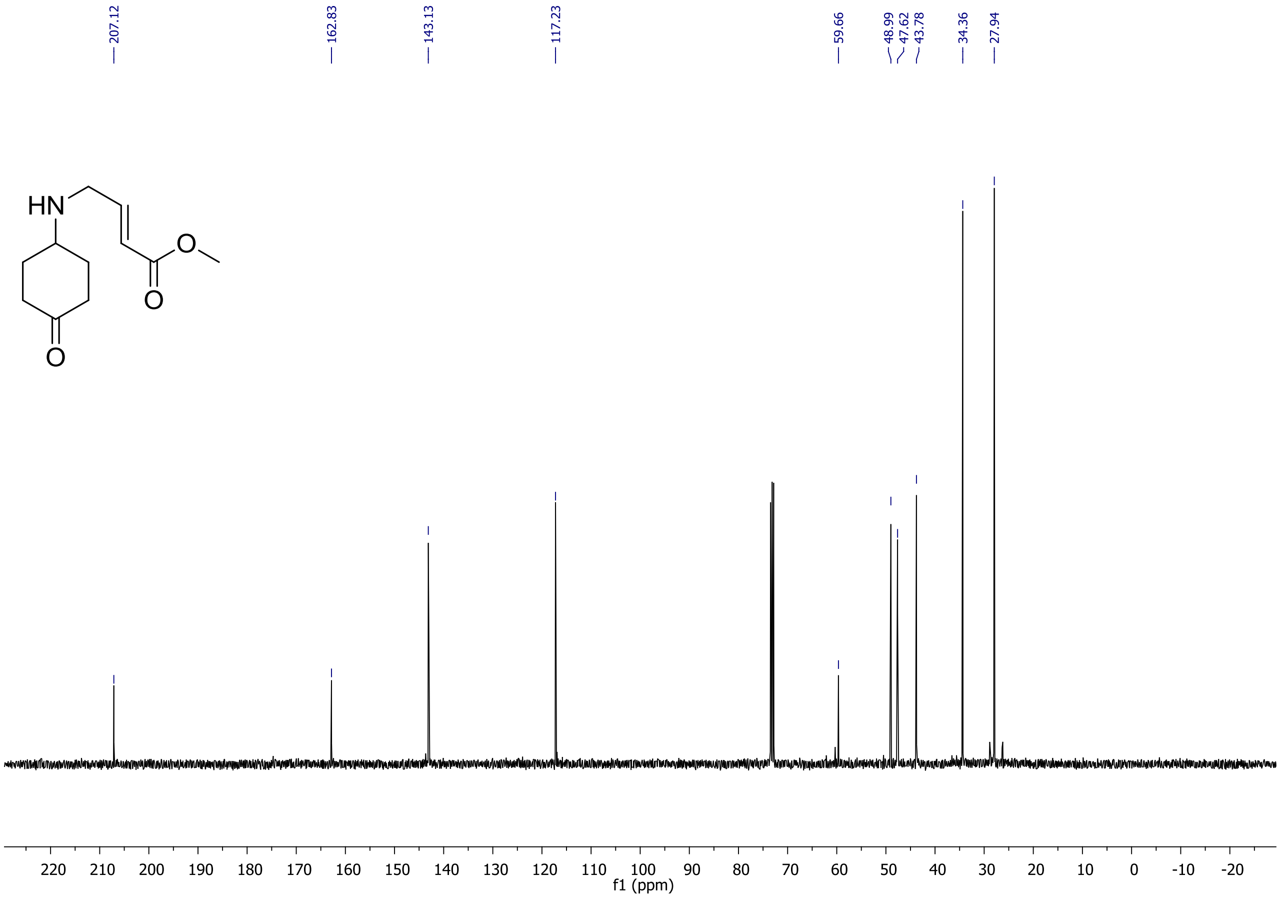

¹H NMR Spectrum of compound 2r

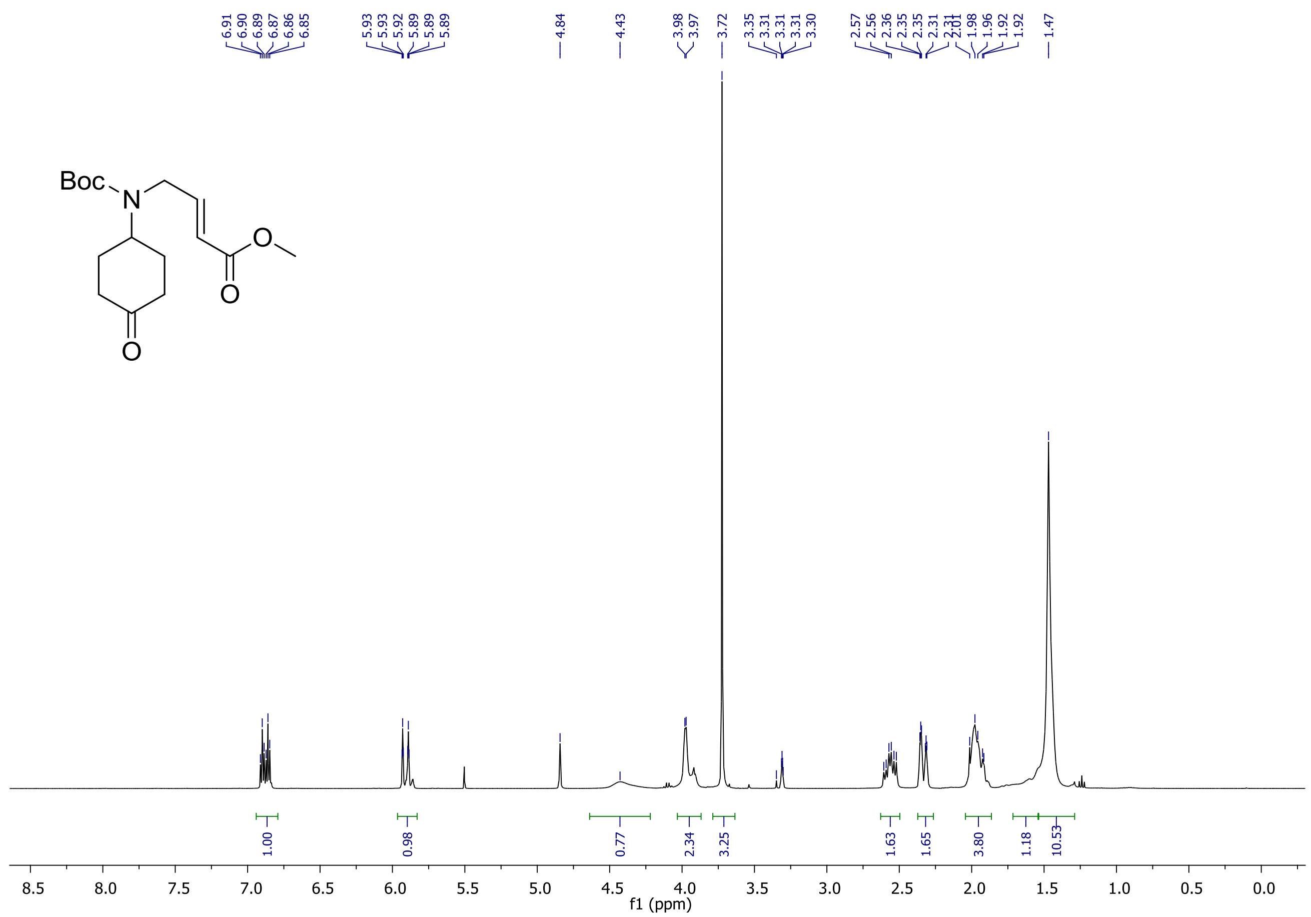

^{13}C NMR Spectrum of compound 2r

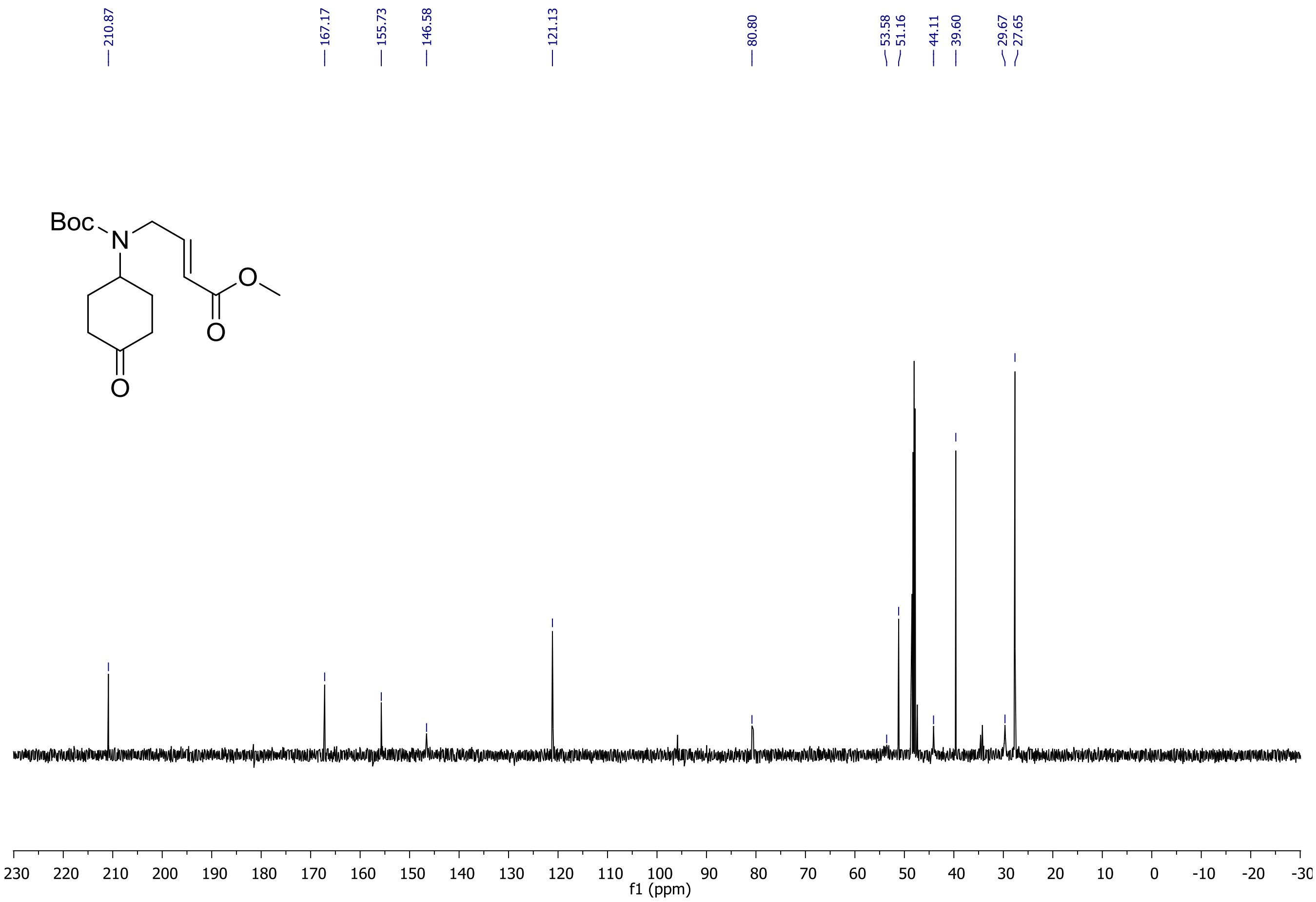

^{1}H NMR Spectrum of compound 2t

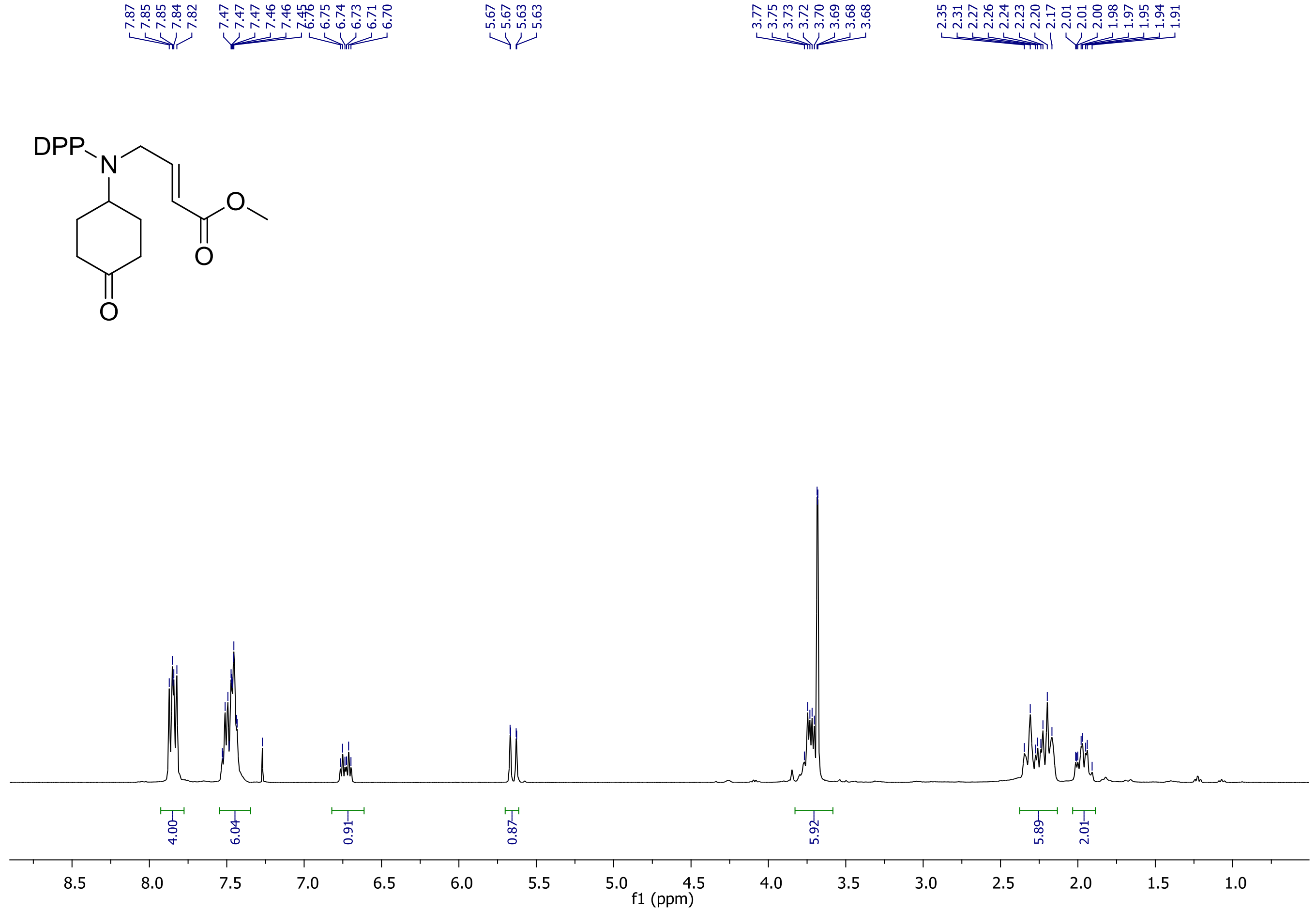

^{13}C NMR Spectrum of compound 2t

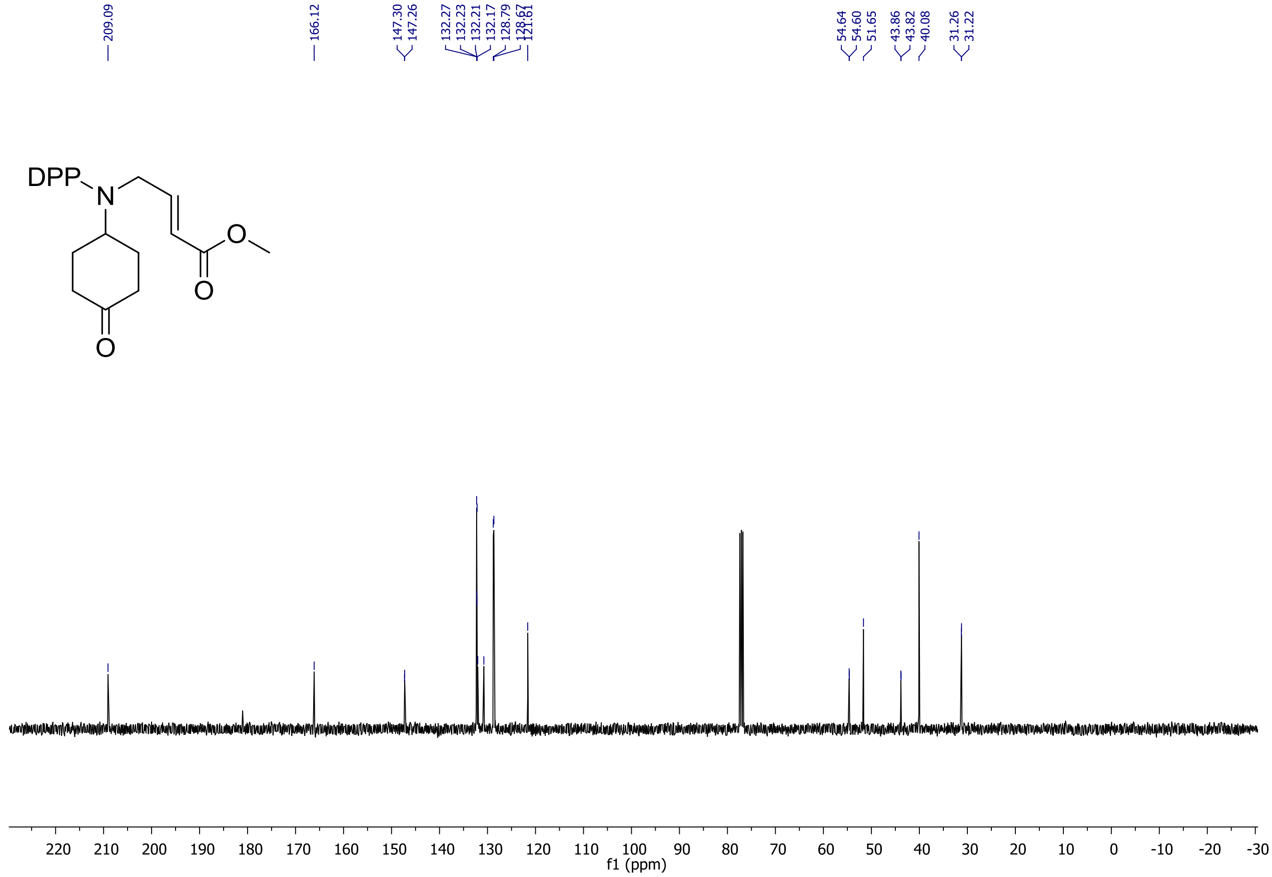

^{1}H NMR Spectrum of compound 32

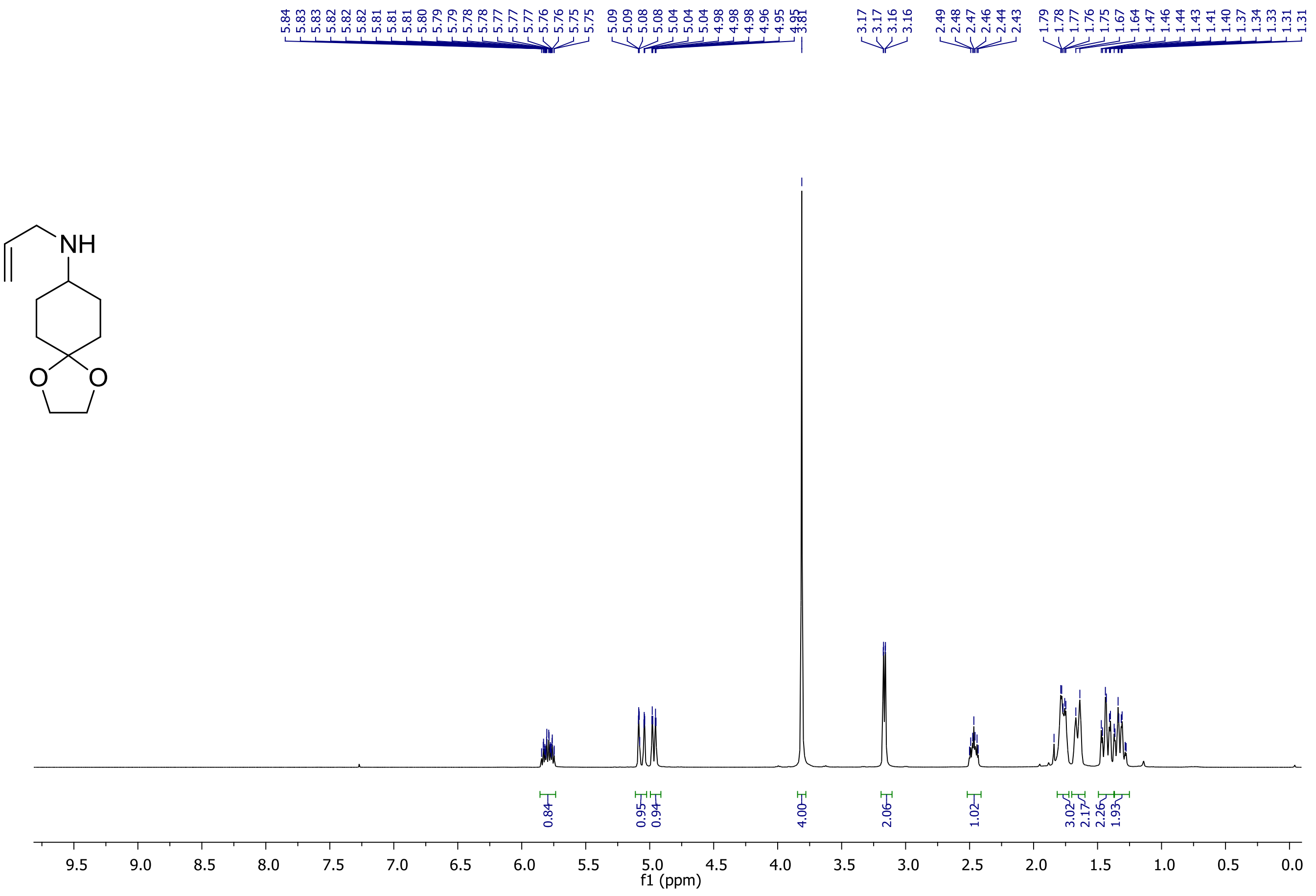

^{13}C NMR Spectrum of compound 32

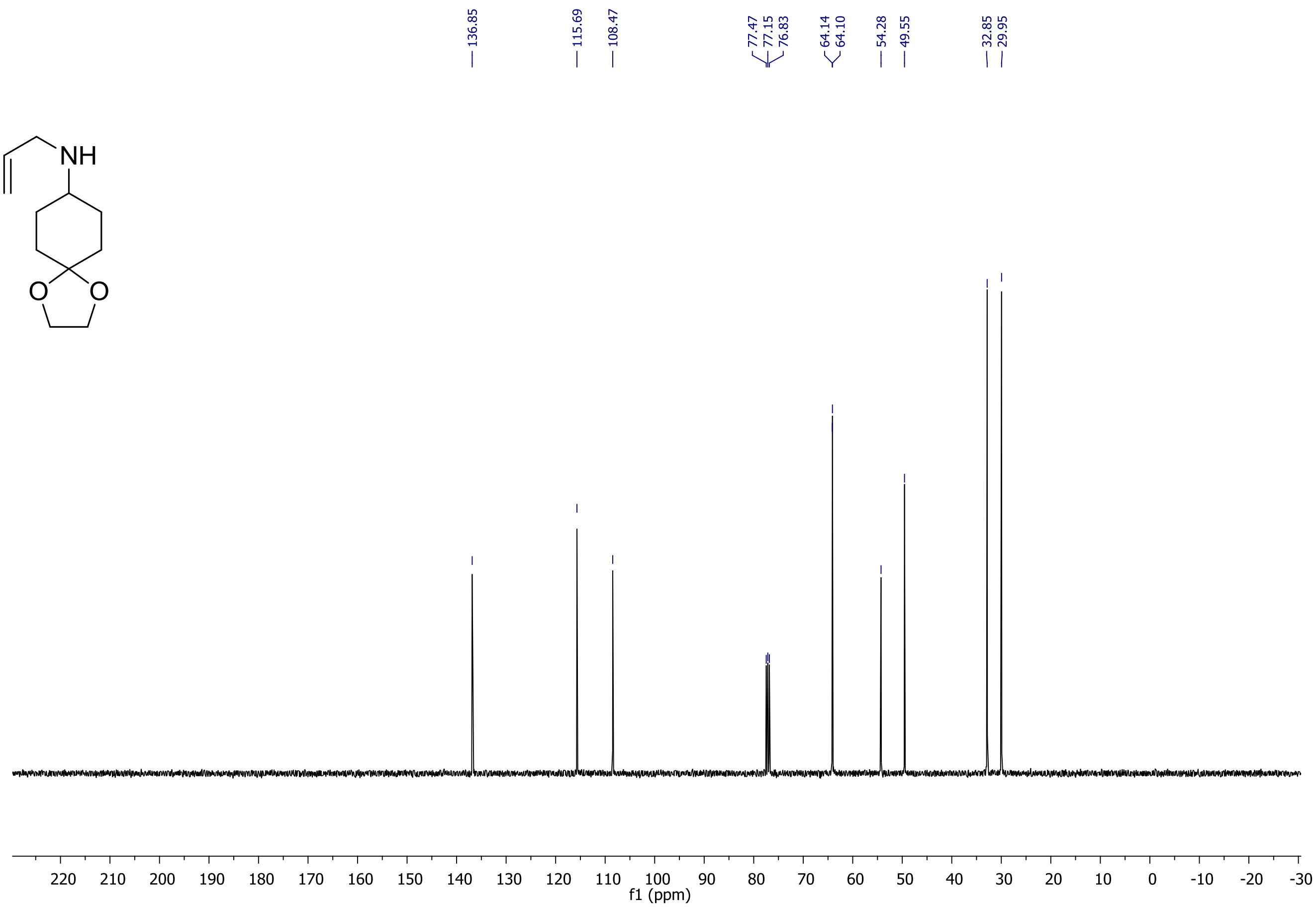

^{1}H NMR Spectrum of compound 33

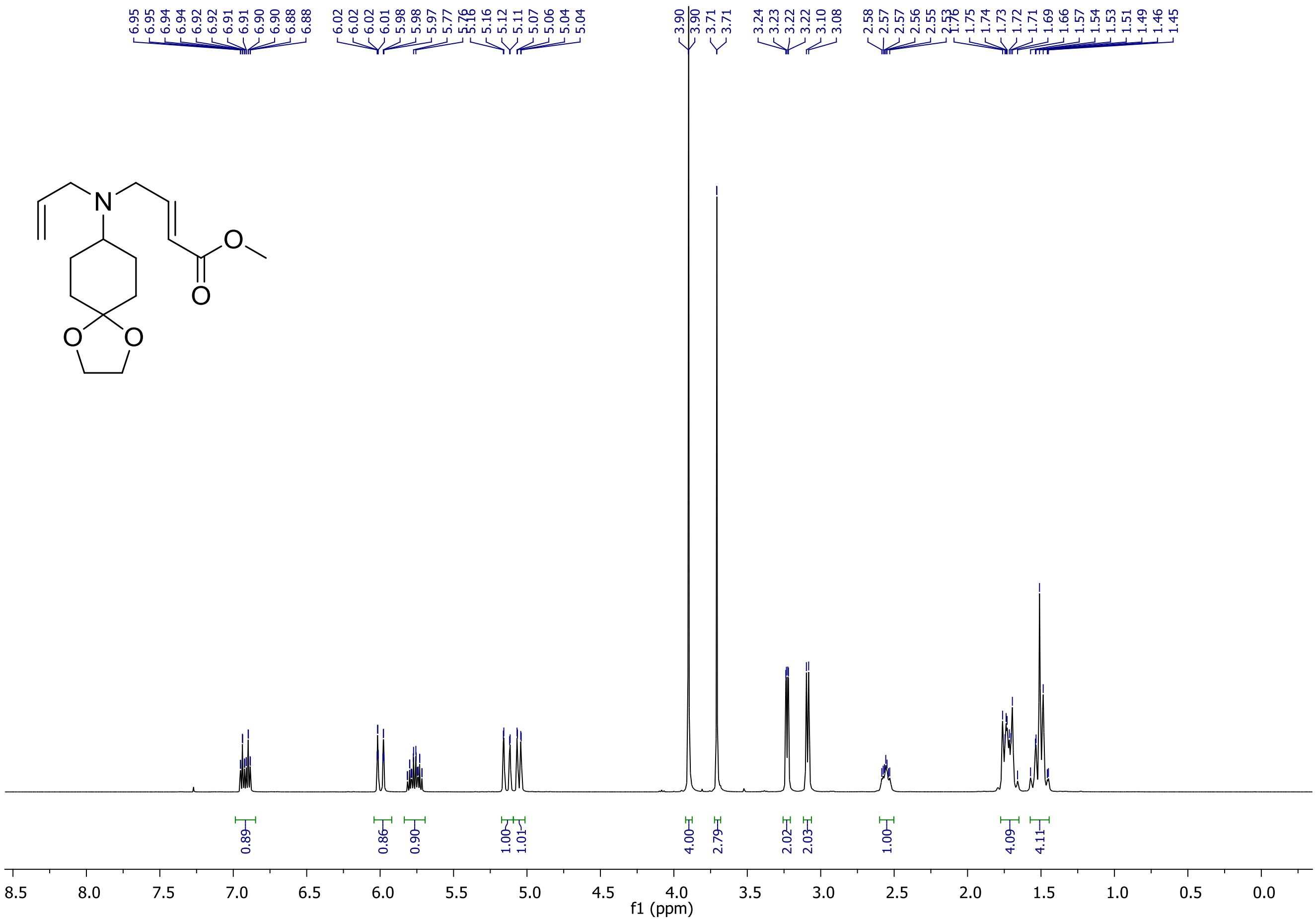

^{13}C NMR Spectrum of compound 33

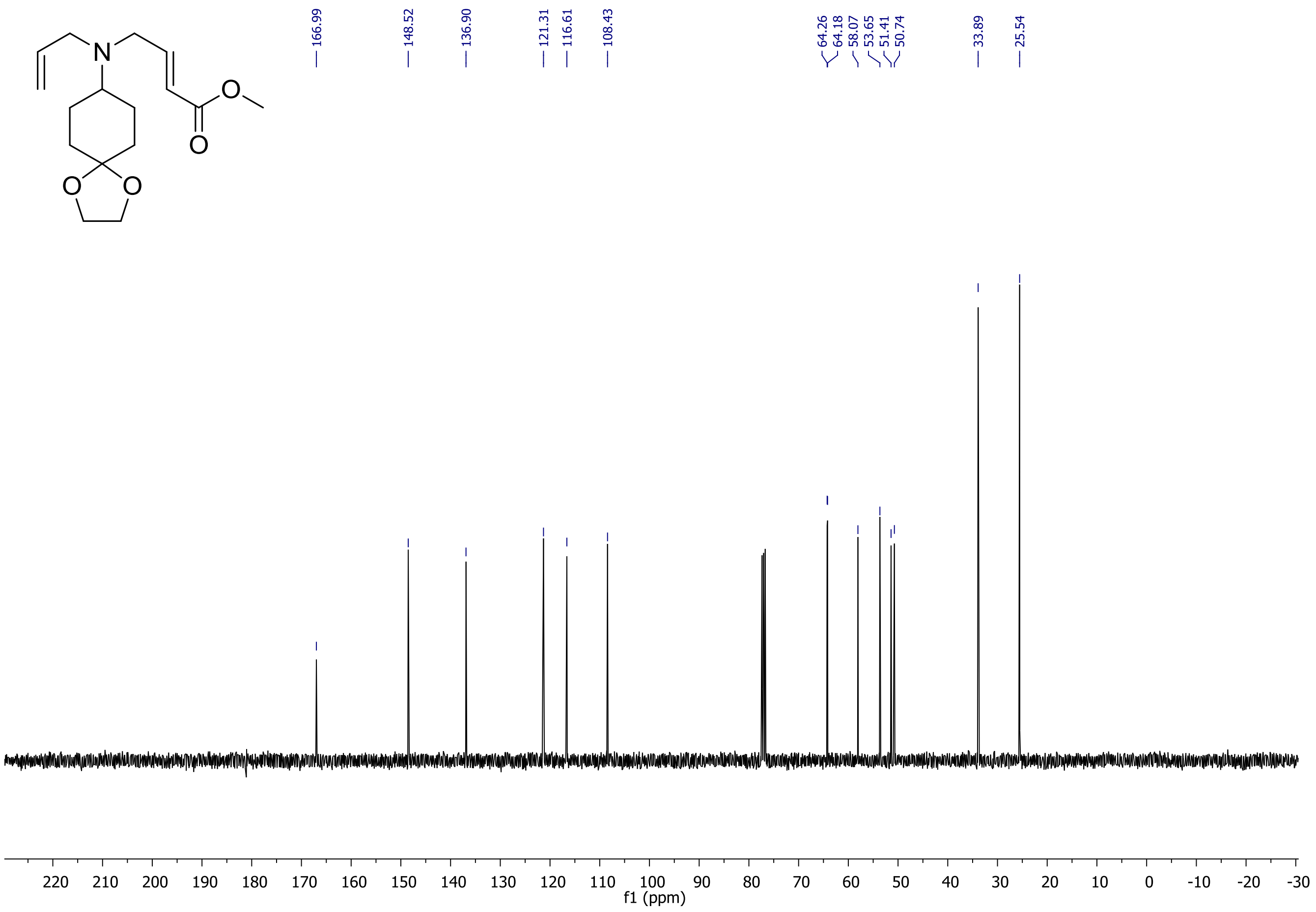

^{1}H NMR Spectrum of compound 2s

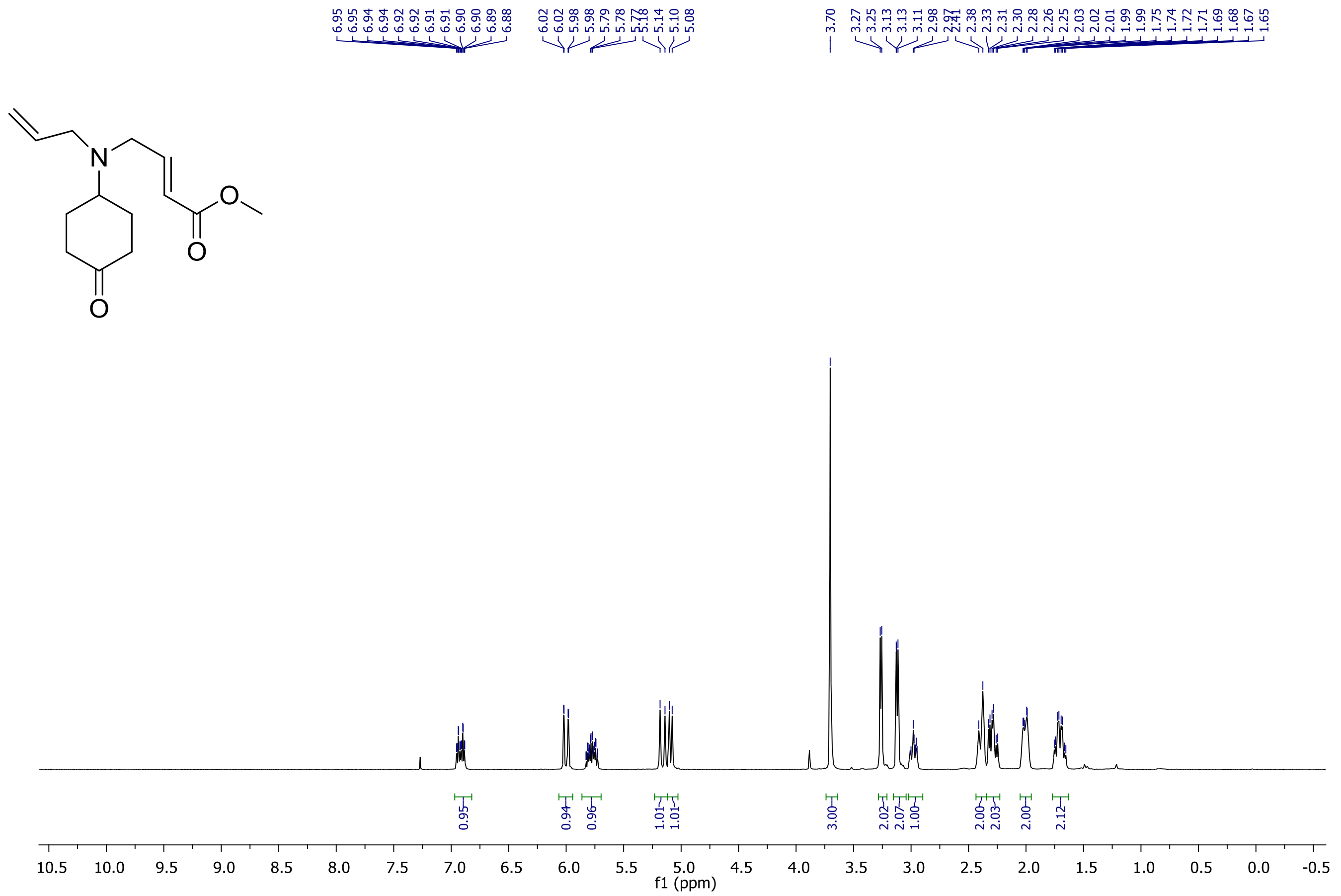

^{13}C NMR Spectrum of compound 2s

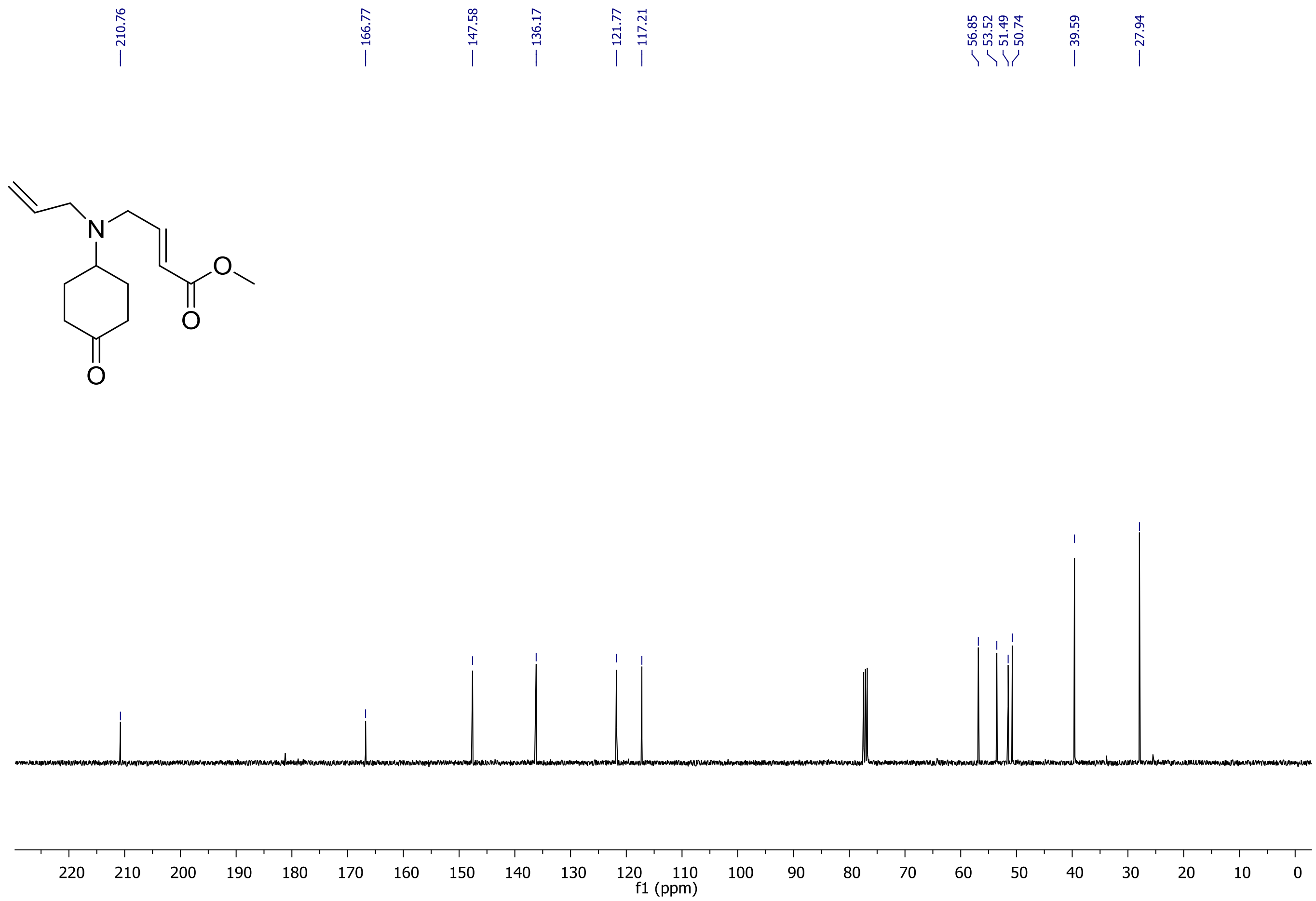

¹H NMR Spectrum of compound 2u

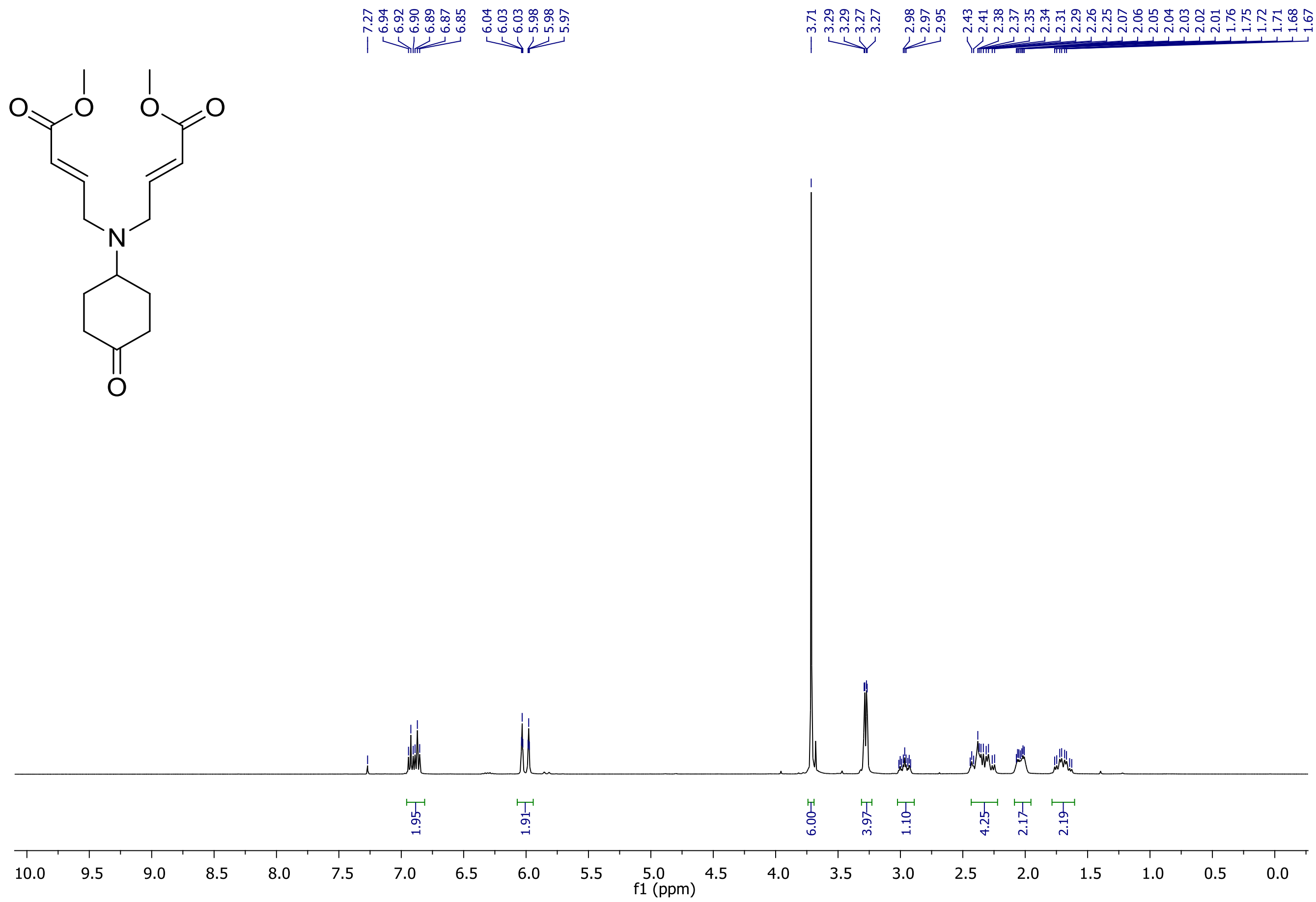

¹³C NMR Spectrum of compound 2u

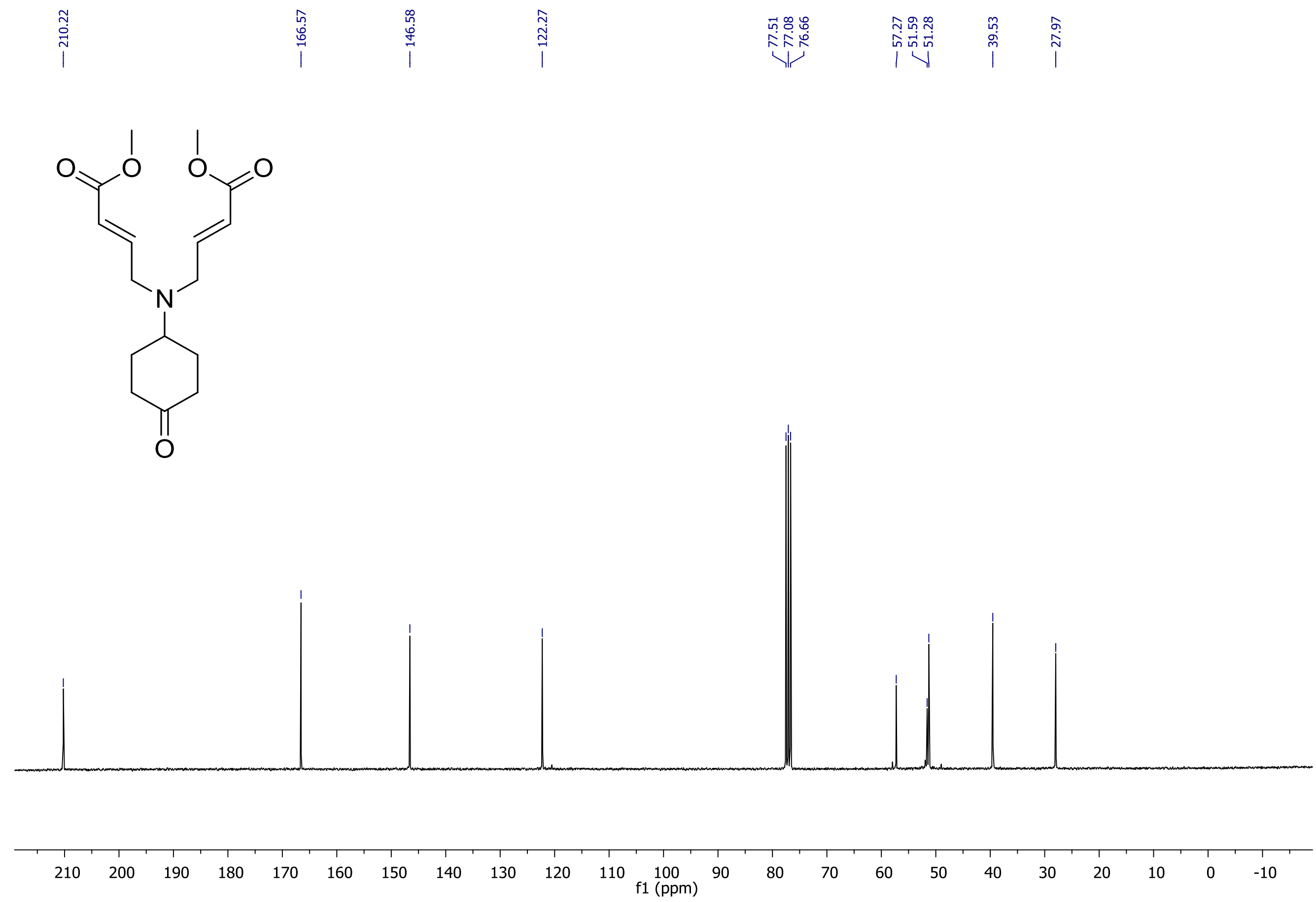

^{1}H NMR Spectrum of compound 2v

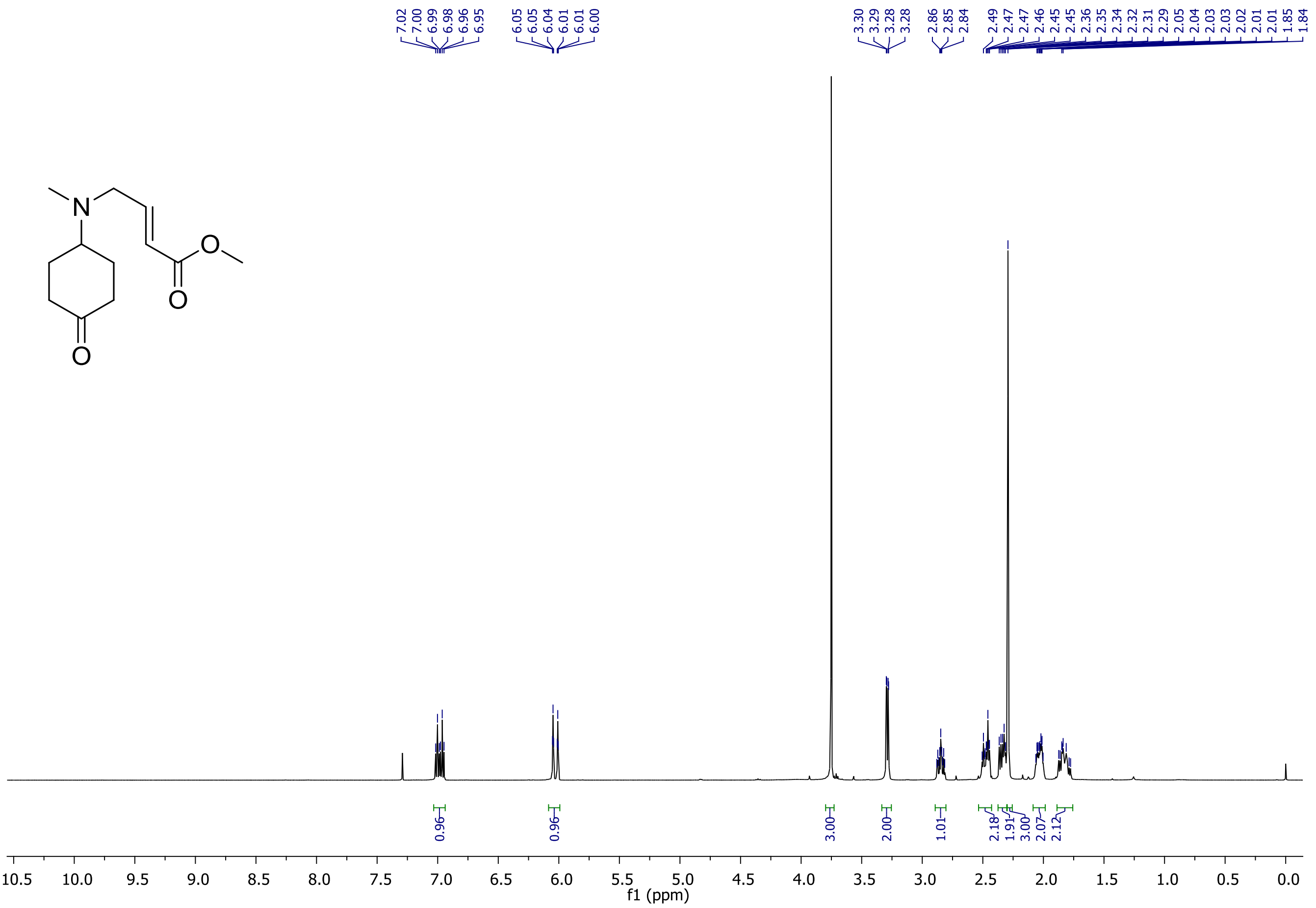

^{13}C NMR Spectrum of compound 2v

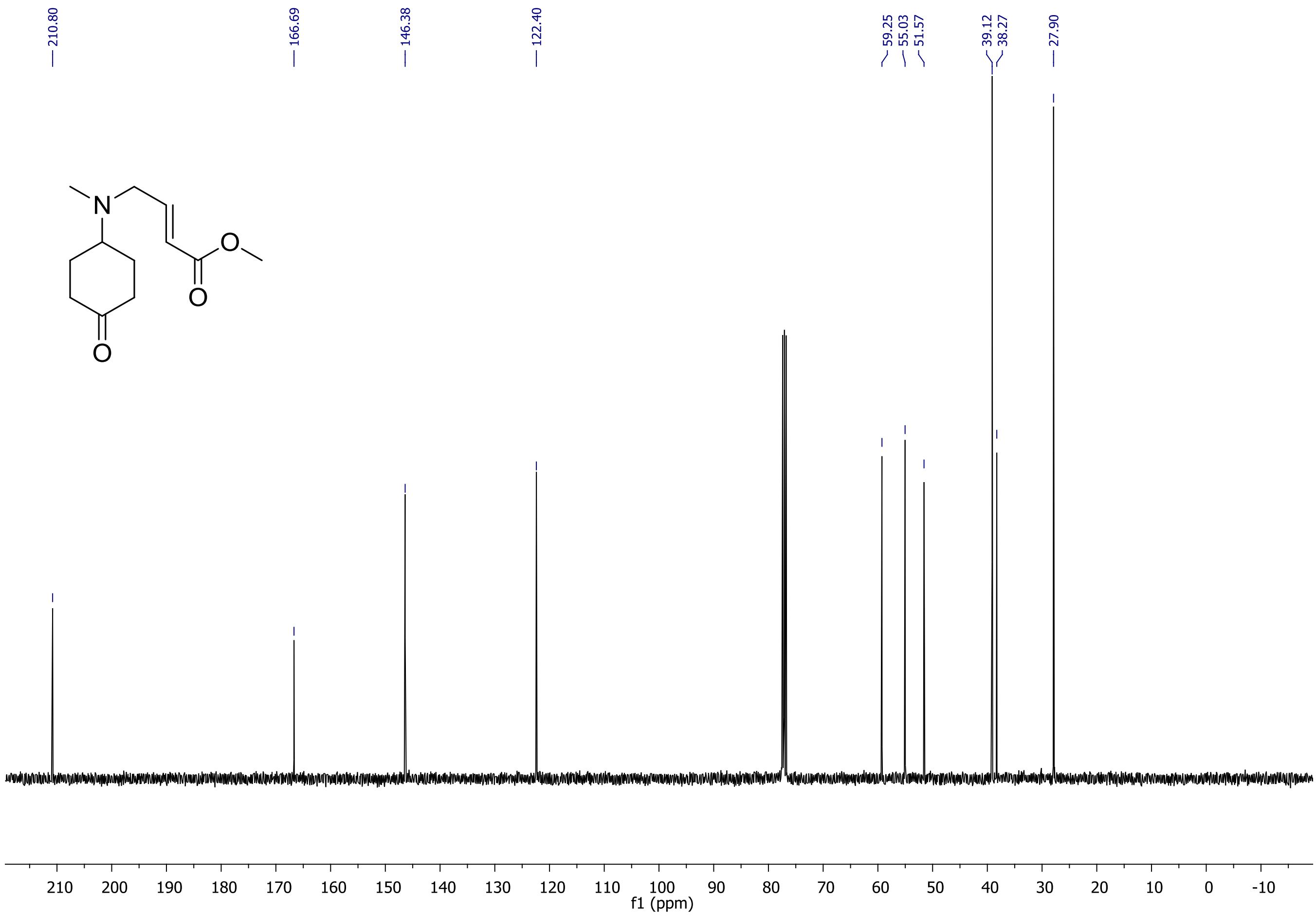

^{1}H NMR Spectrum of compound 3a

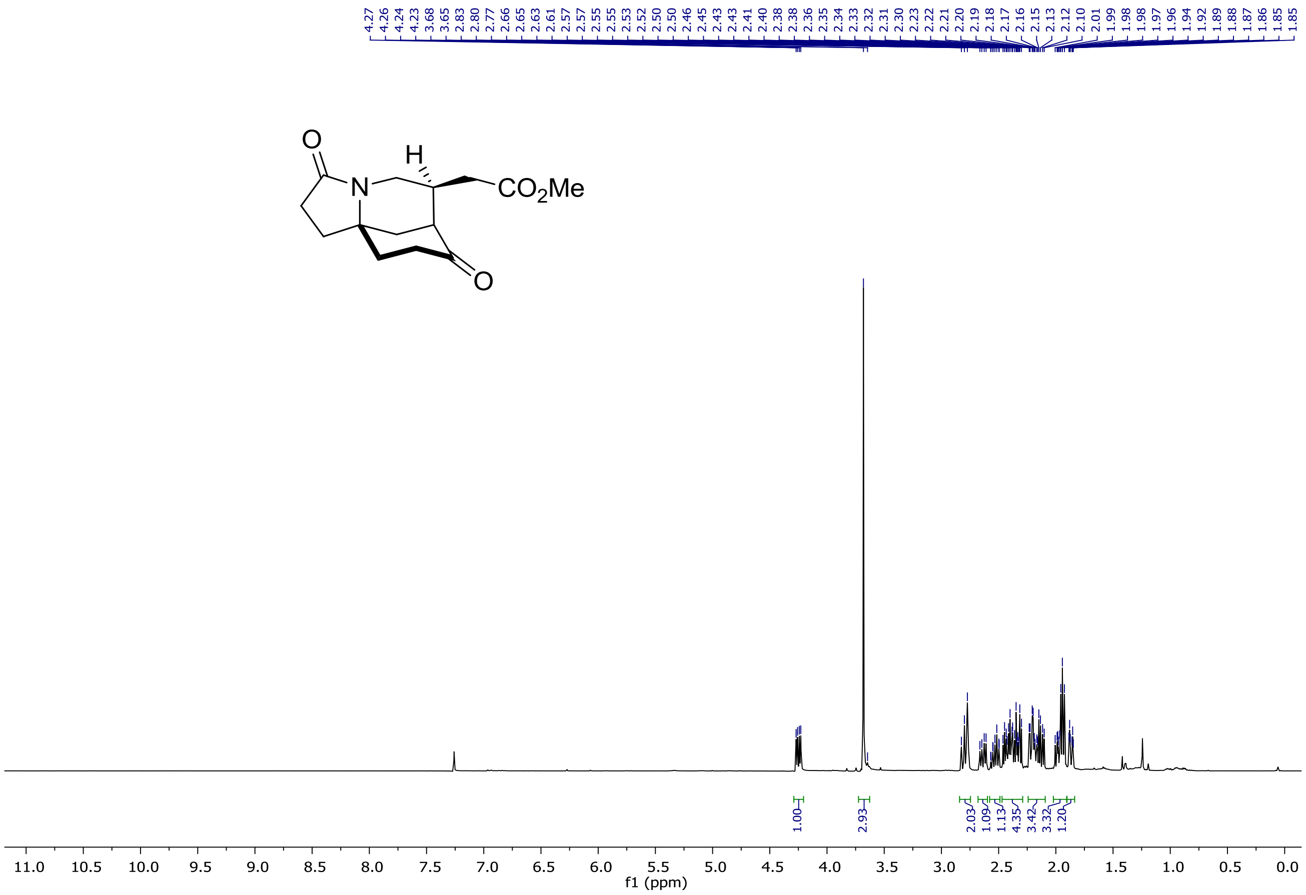

^{13}C NMR Spectrum of compound 3a

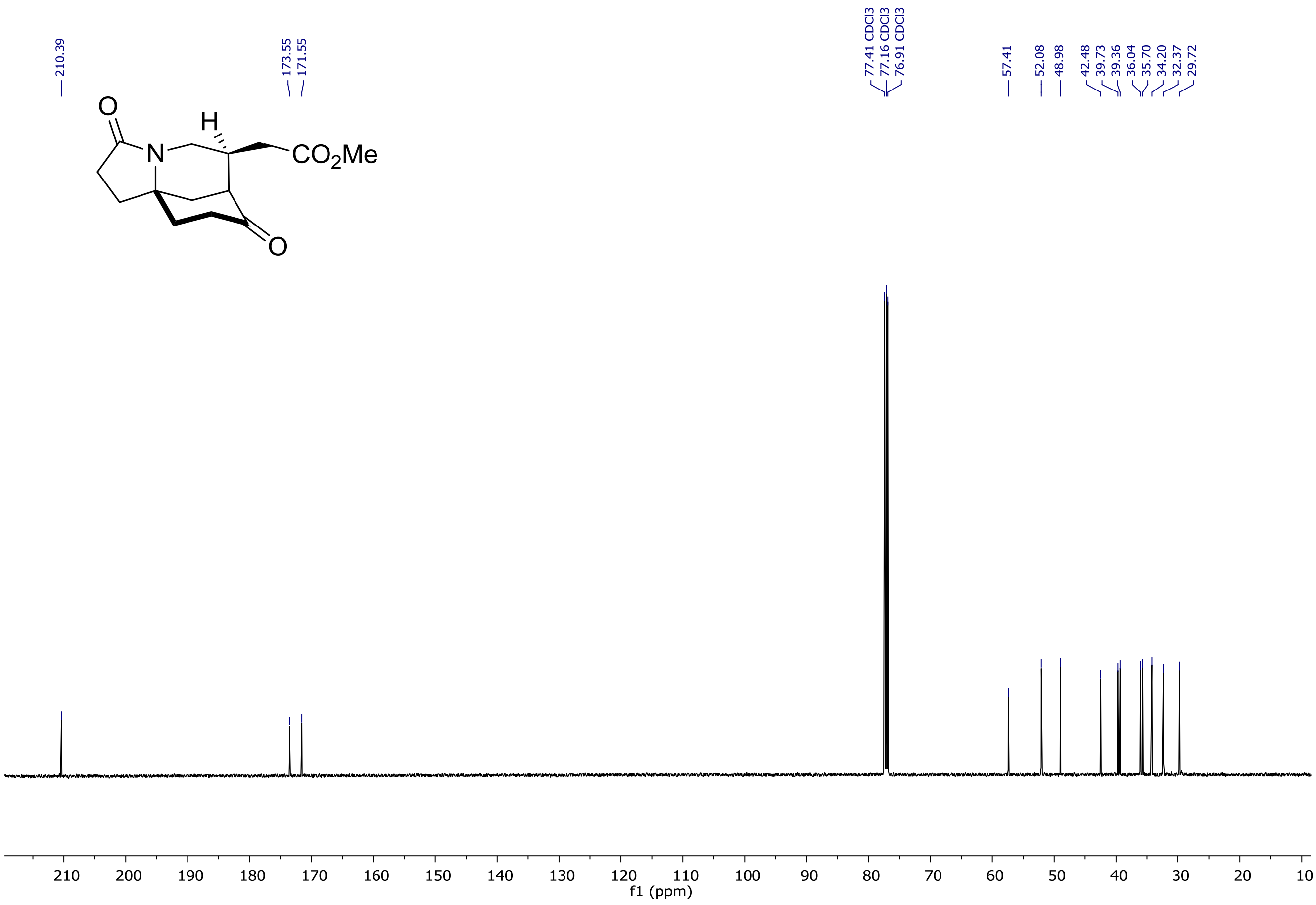

^{1}H NMR Spectrum of compound 3b

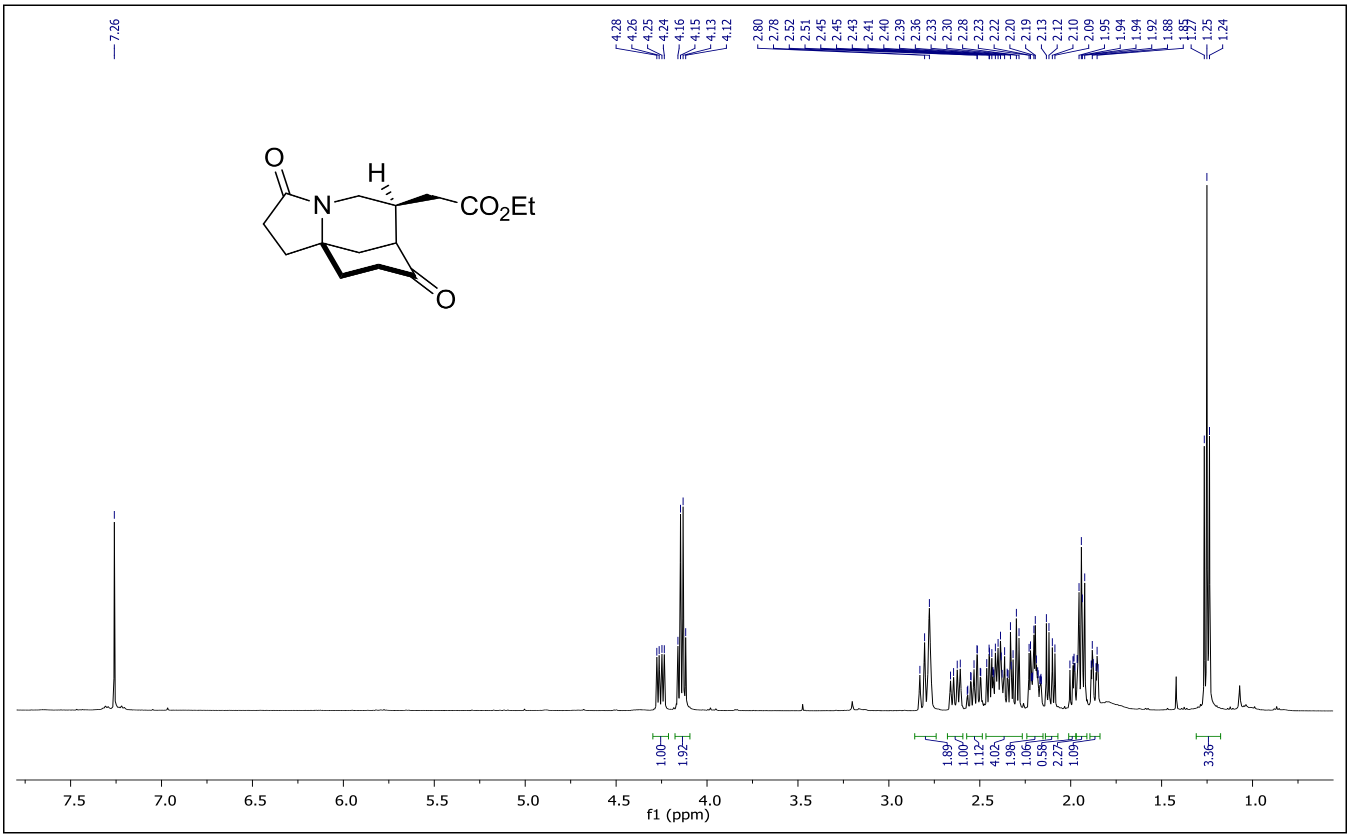

^{13}C NMR Spectrum of compound 3b

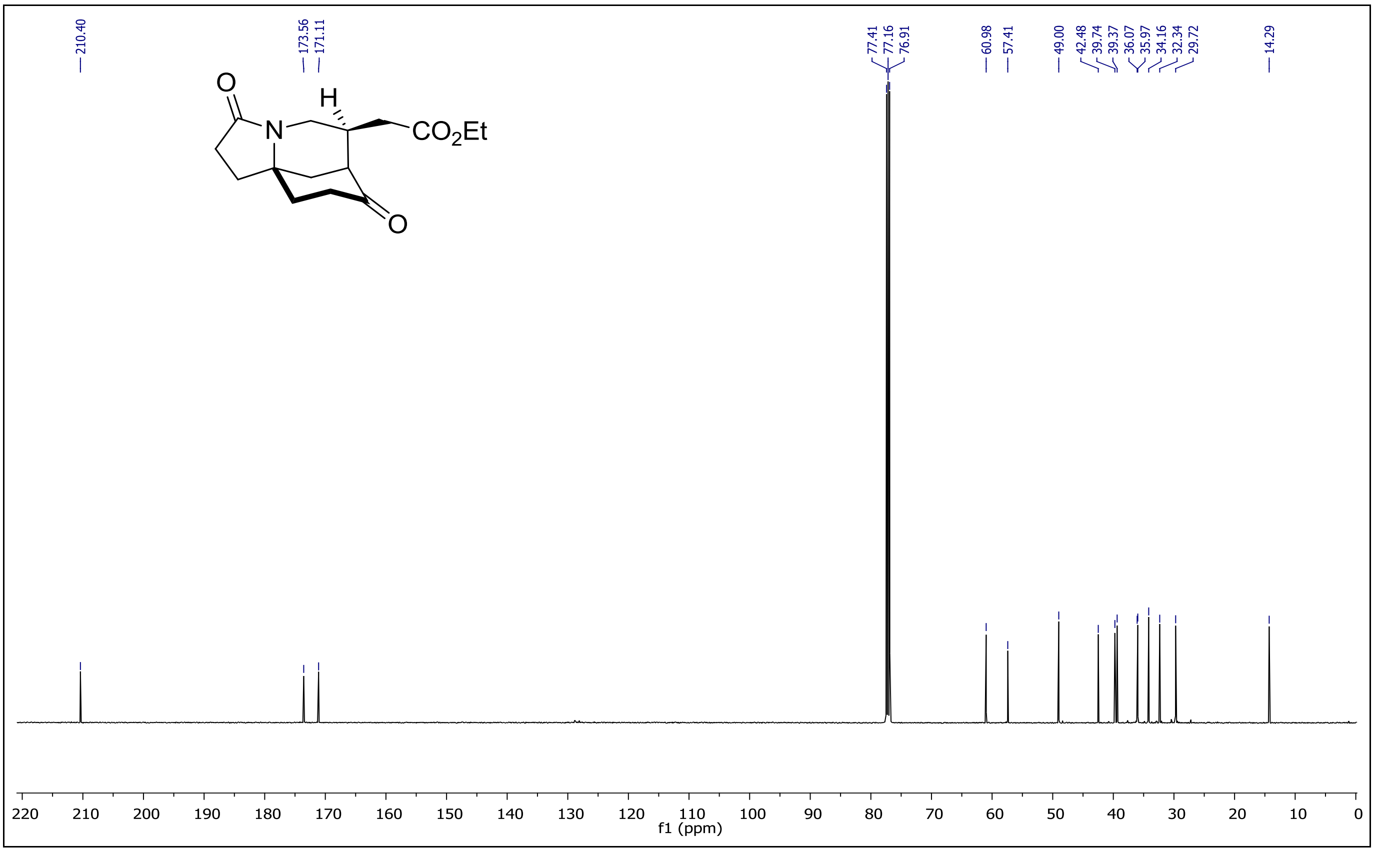

^{1}H NMR Spectrum of compound 3c

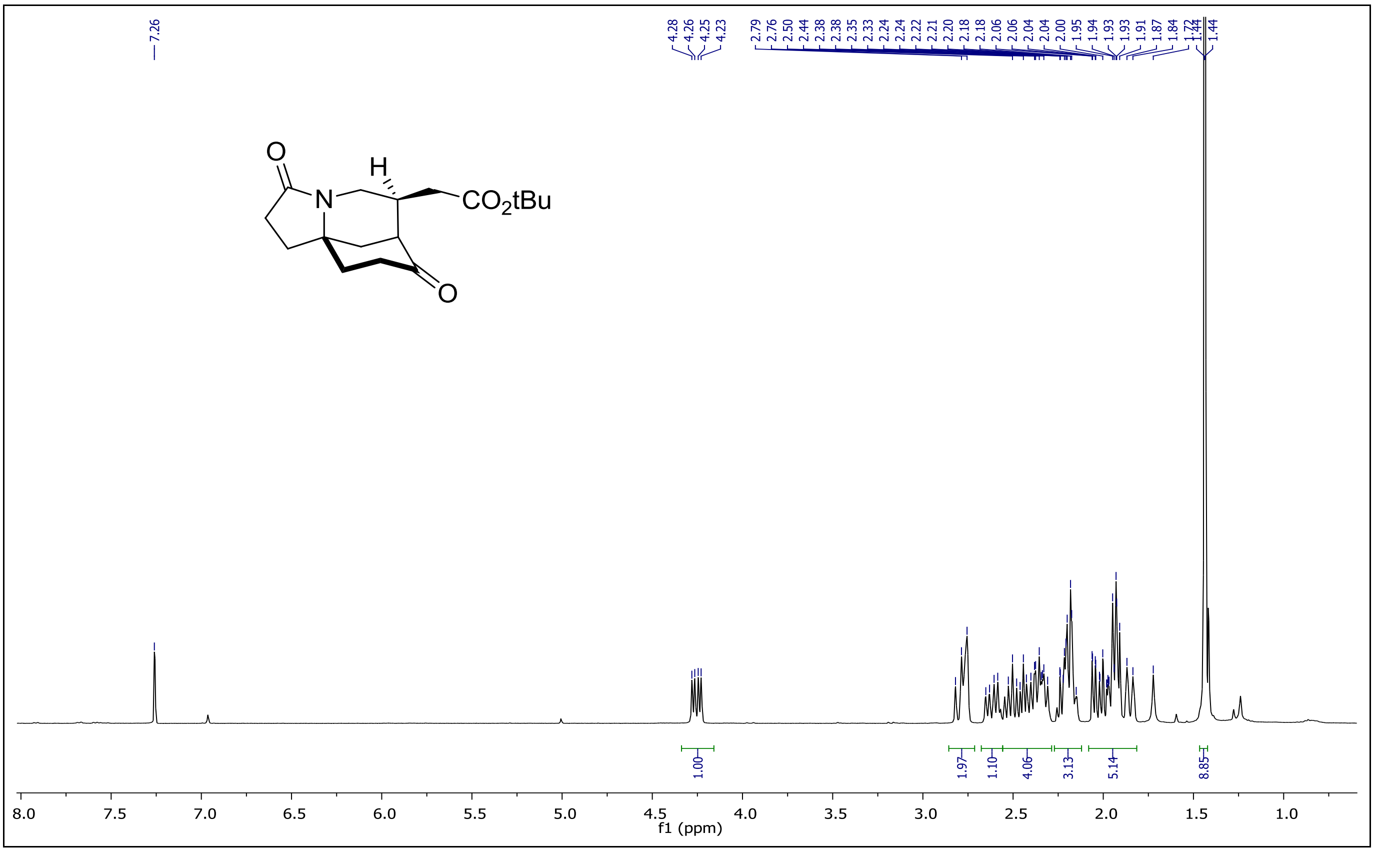

^{13}C NMR Spectrum of compound 3c

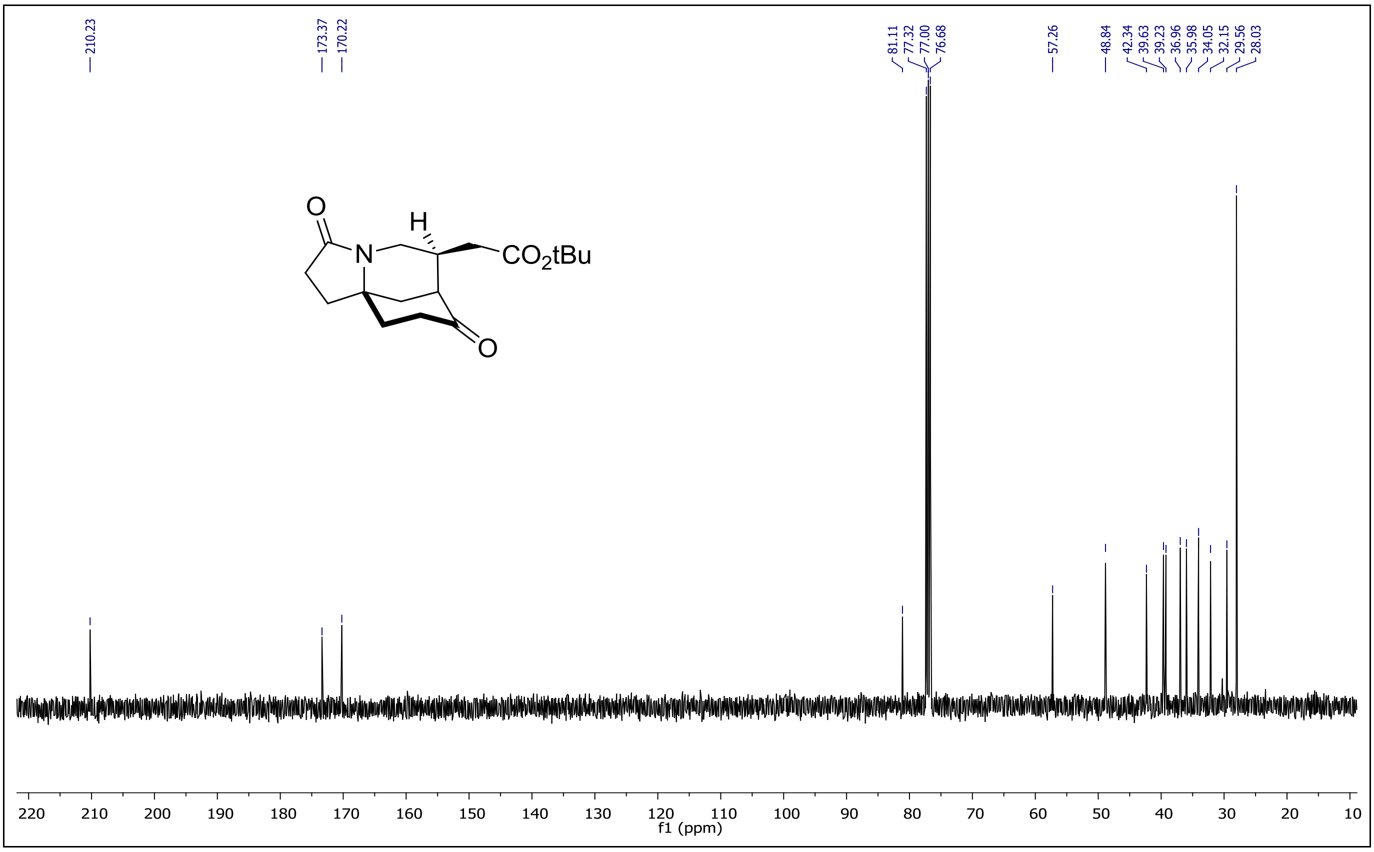

^{1}H NMR Spectrum of compound 3d

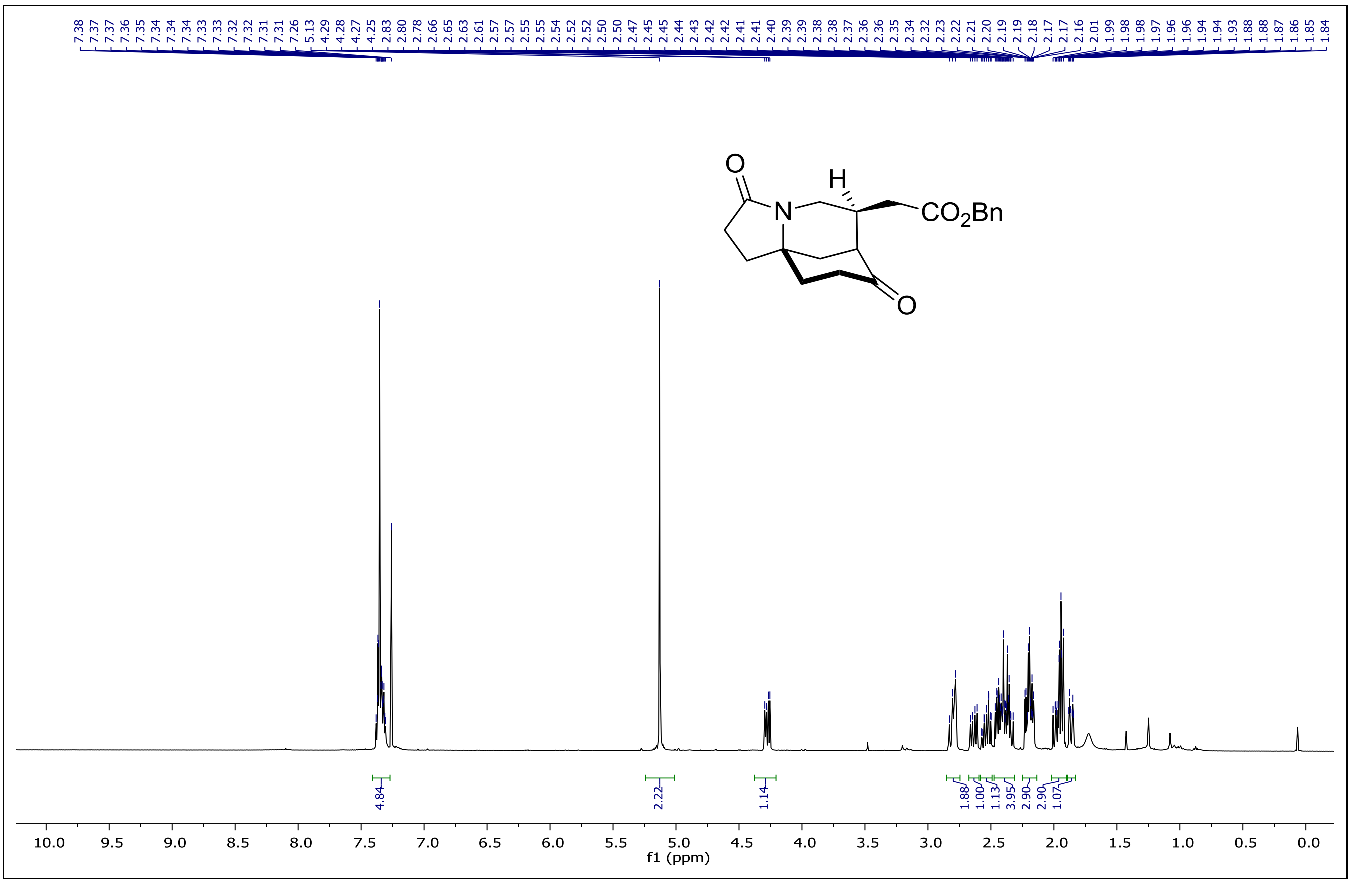

^{13}C NMR Spectrum of compound 3d

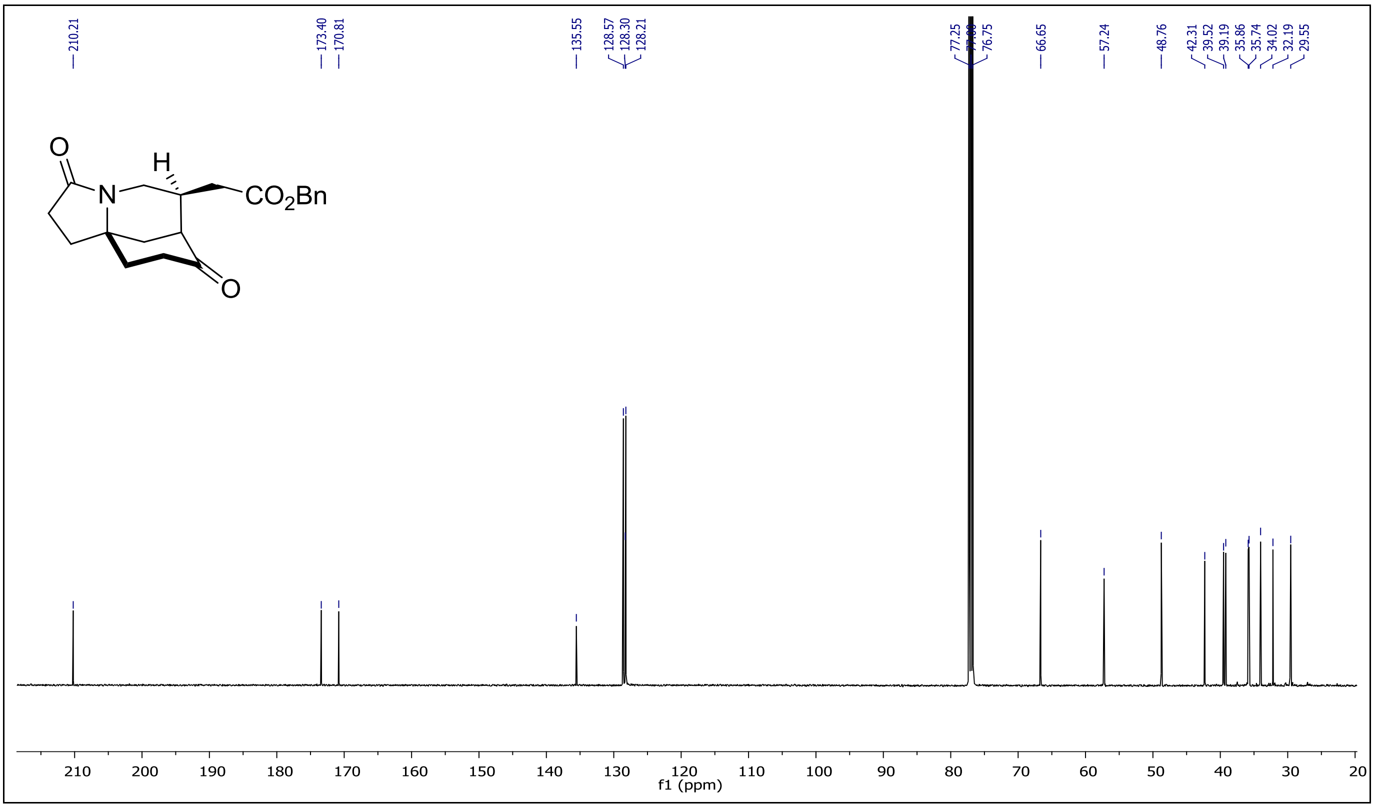

^{1}H NMR Spectrum of compound 3e

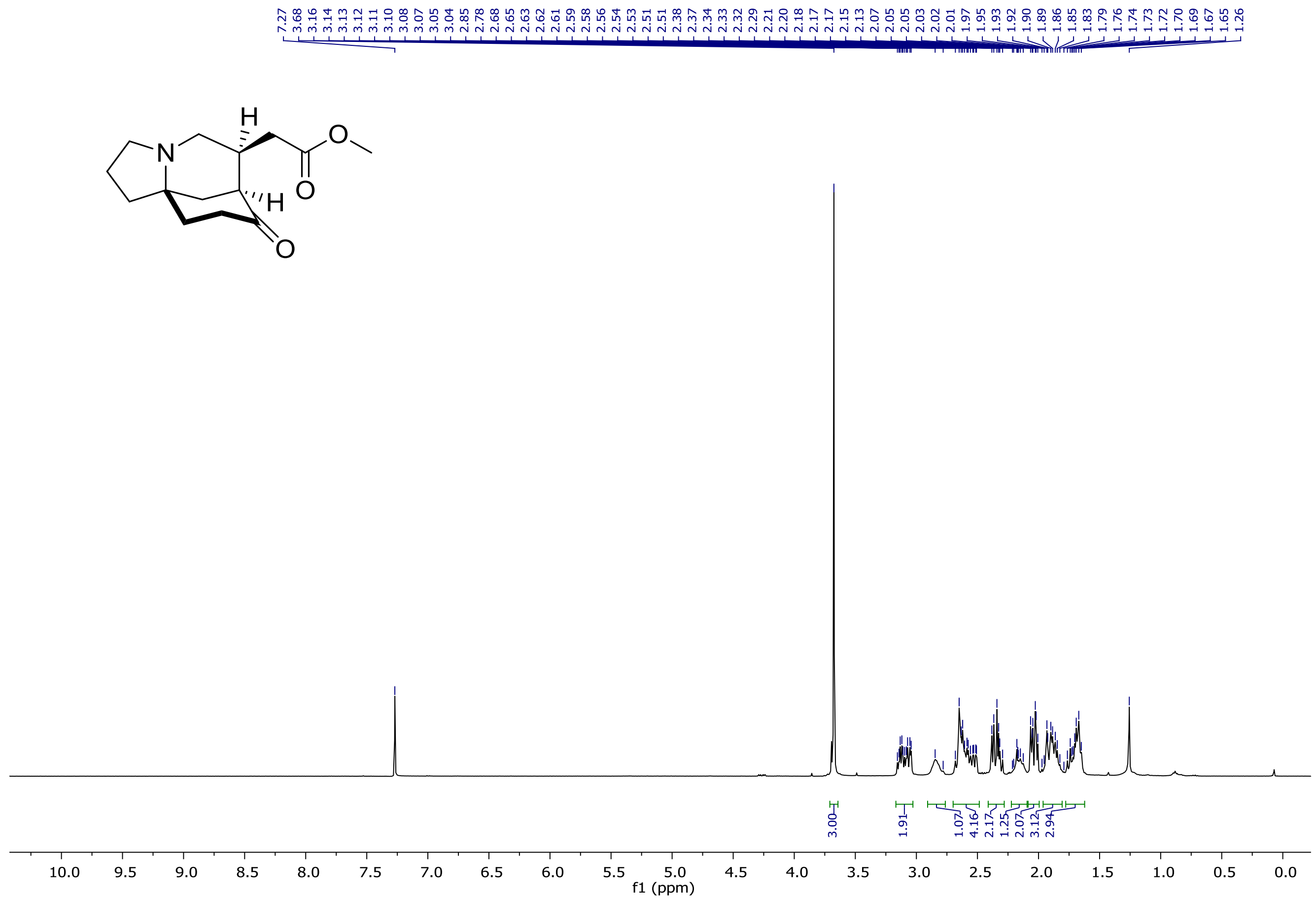

^{13}C NMR Spectrum of compound 3e

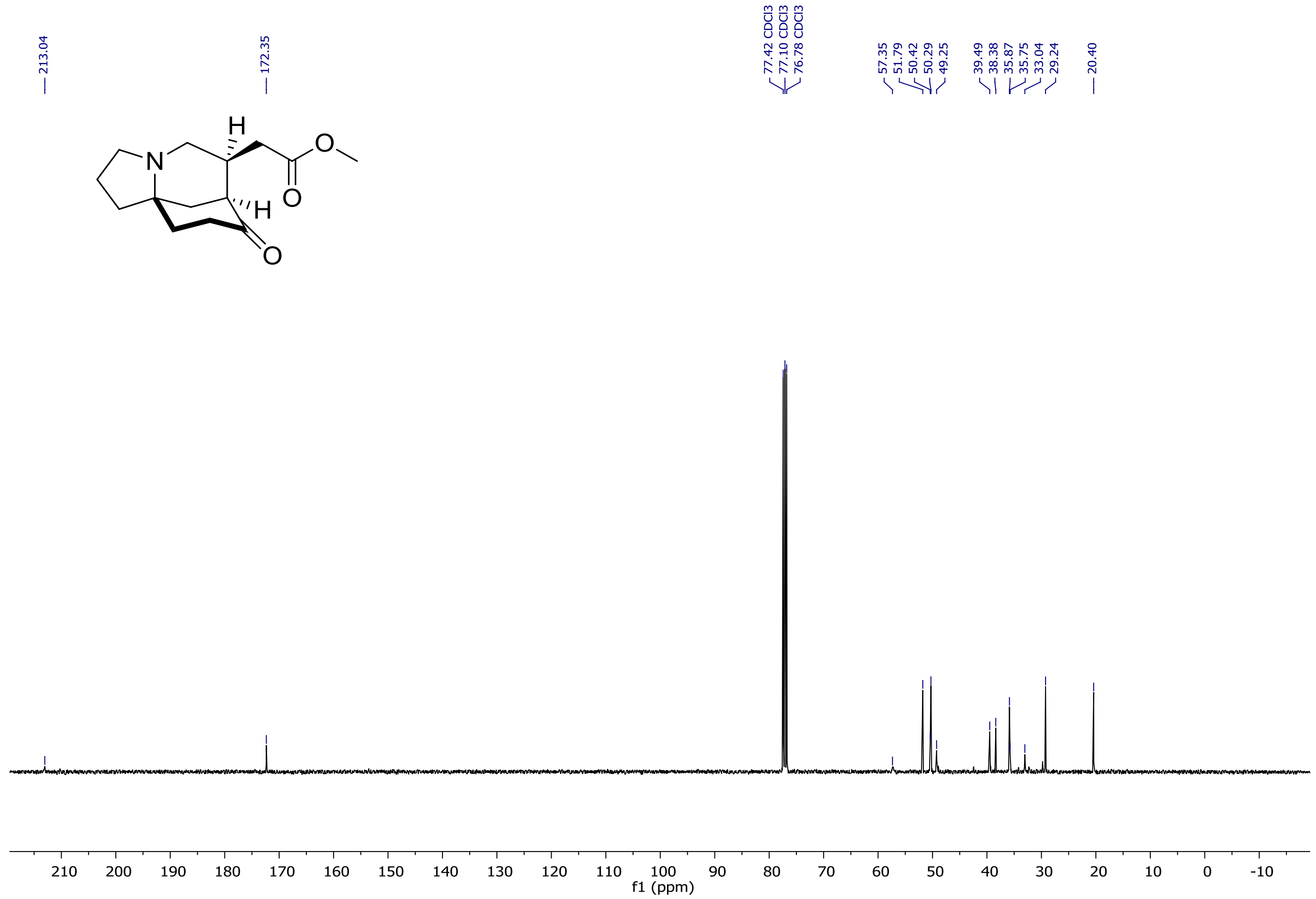

¹H NMR Spectrum of compound 3f

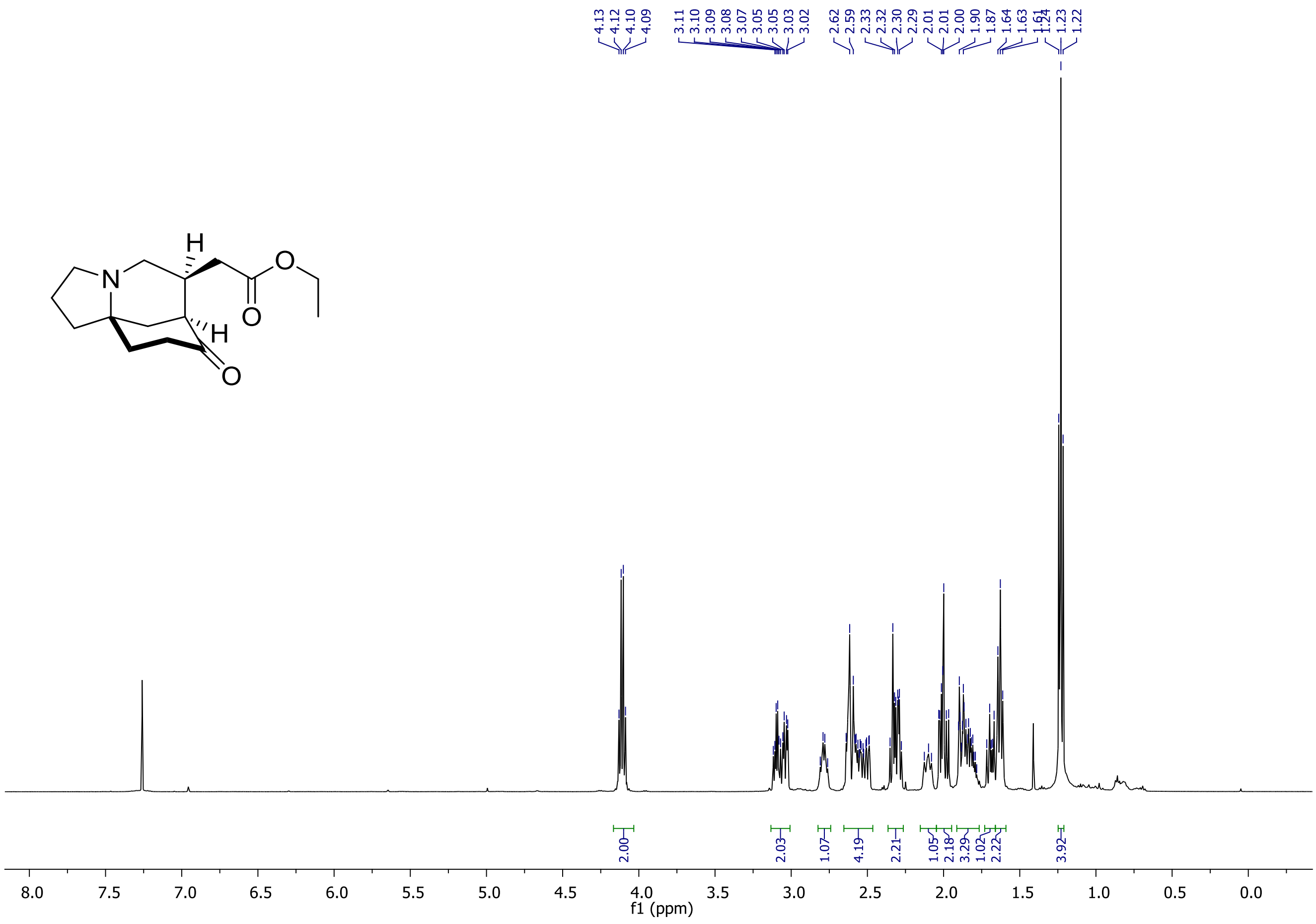

¹³C NMR Spectrum of compound 3f

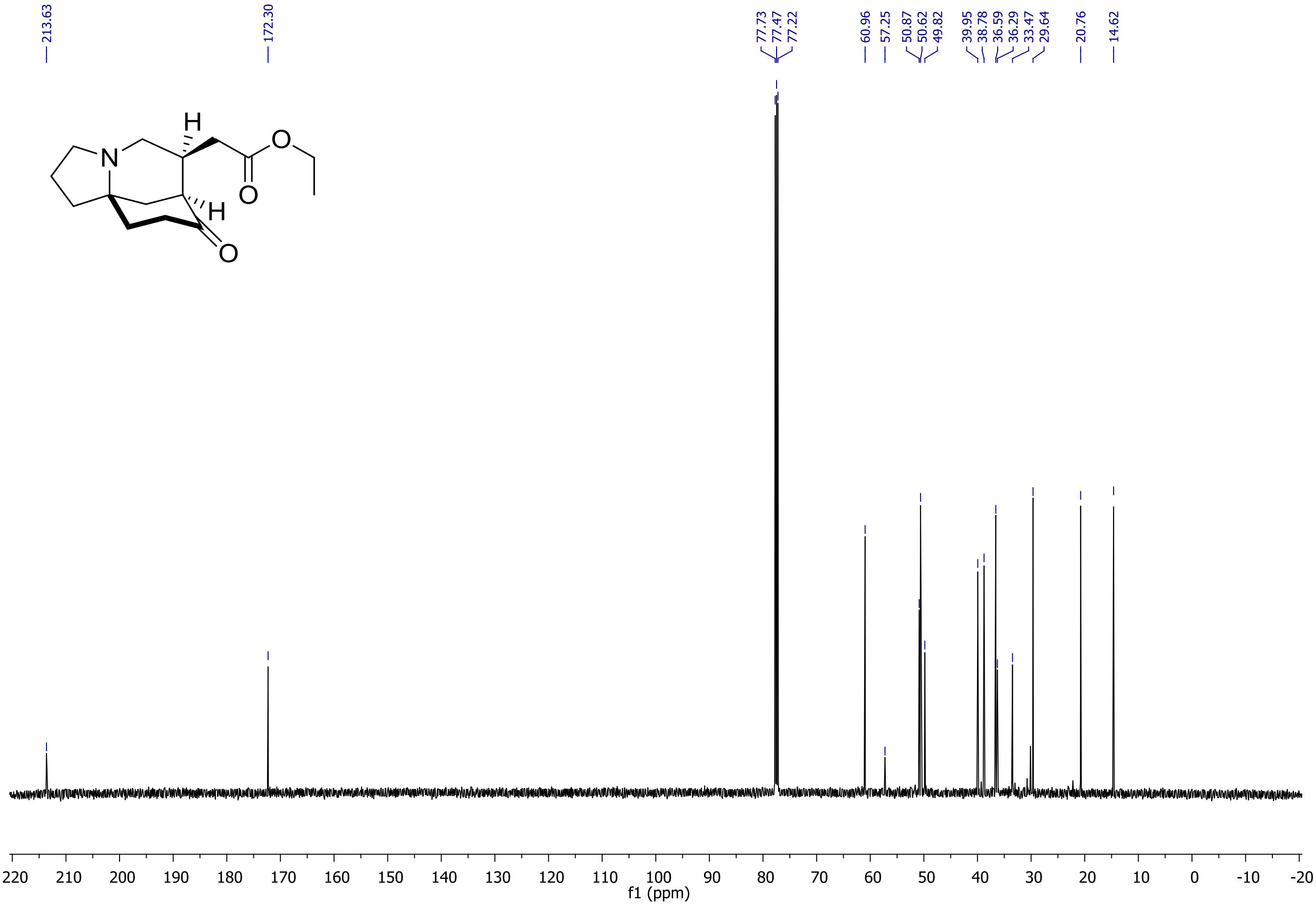

^{1}H NMR Spectrum of compound 3g

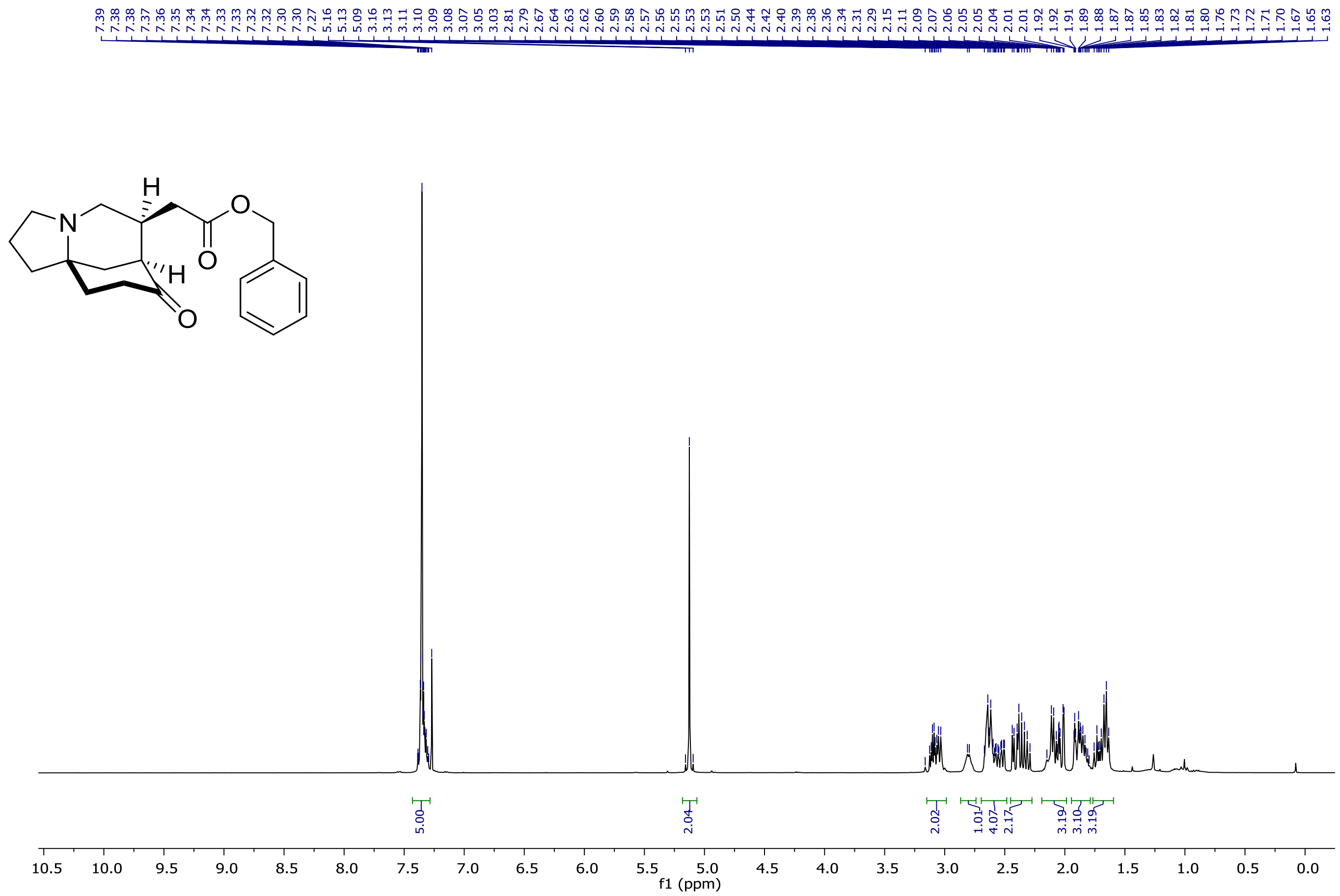

^{13}C NMR Spectrum of compound 3g

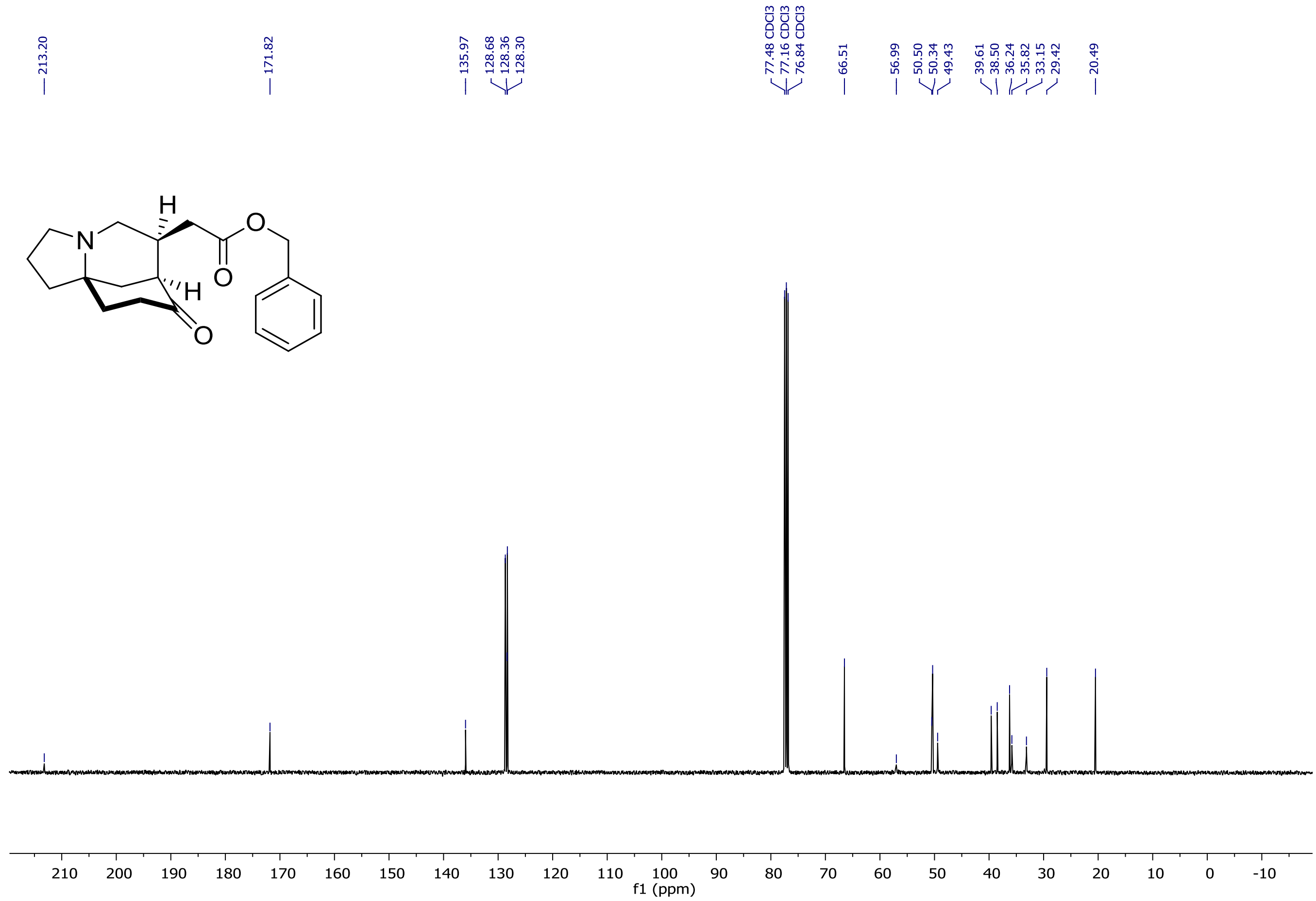

¹H NMR Spectrum of compound 3h

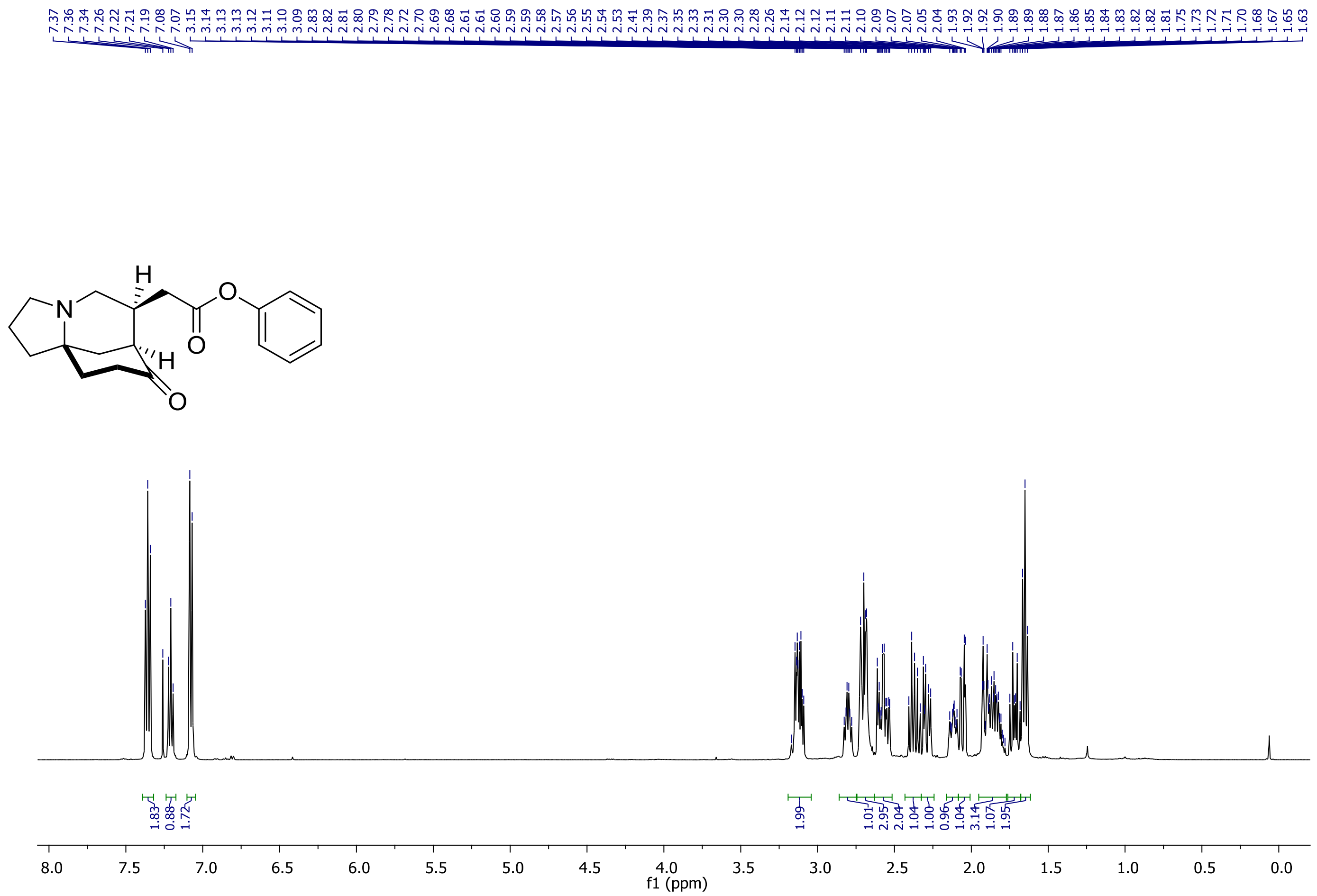

¹³C NMR Spectrum of compound 3h

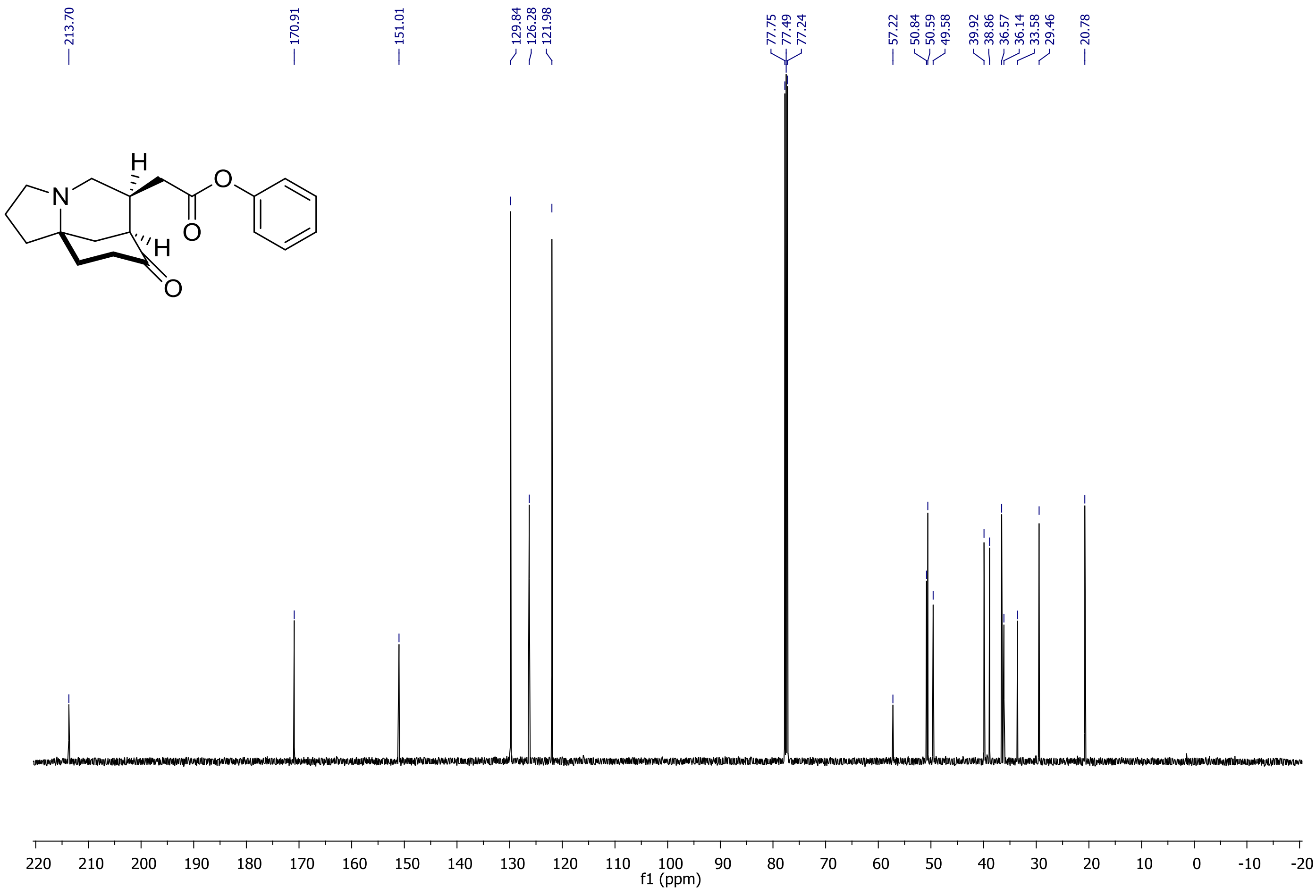

^{1}H NMR Spectrum of compound 3i

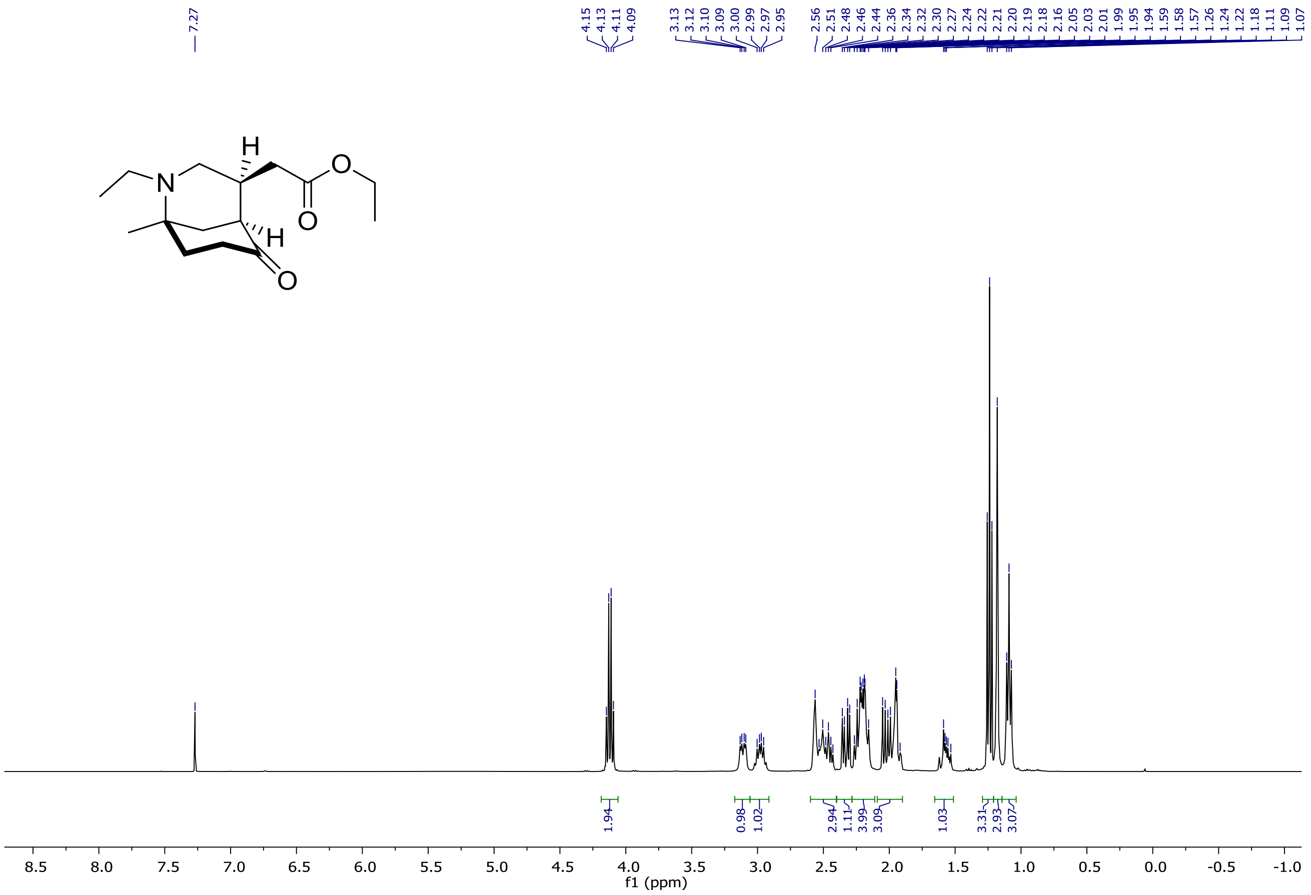

^{13}C NMR Spectrum of compound 3i

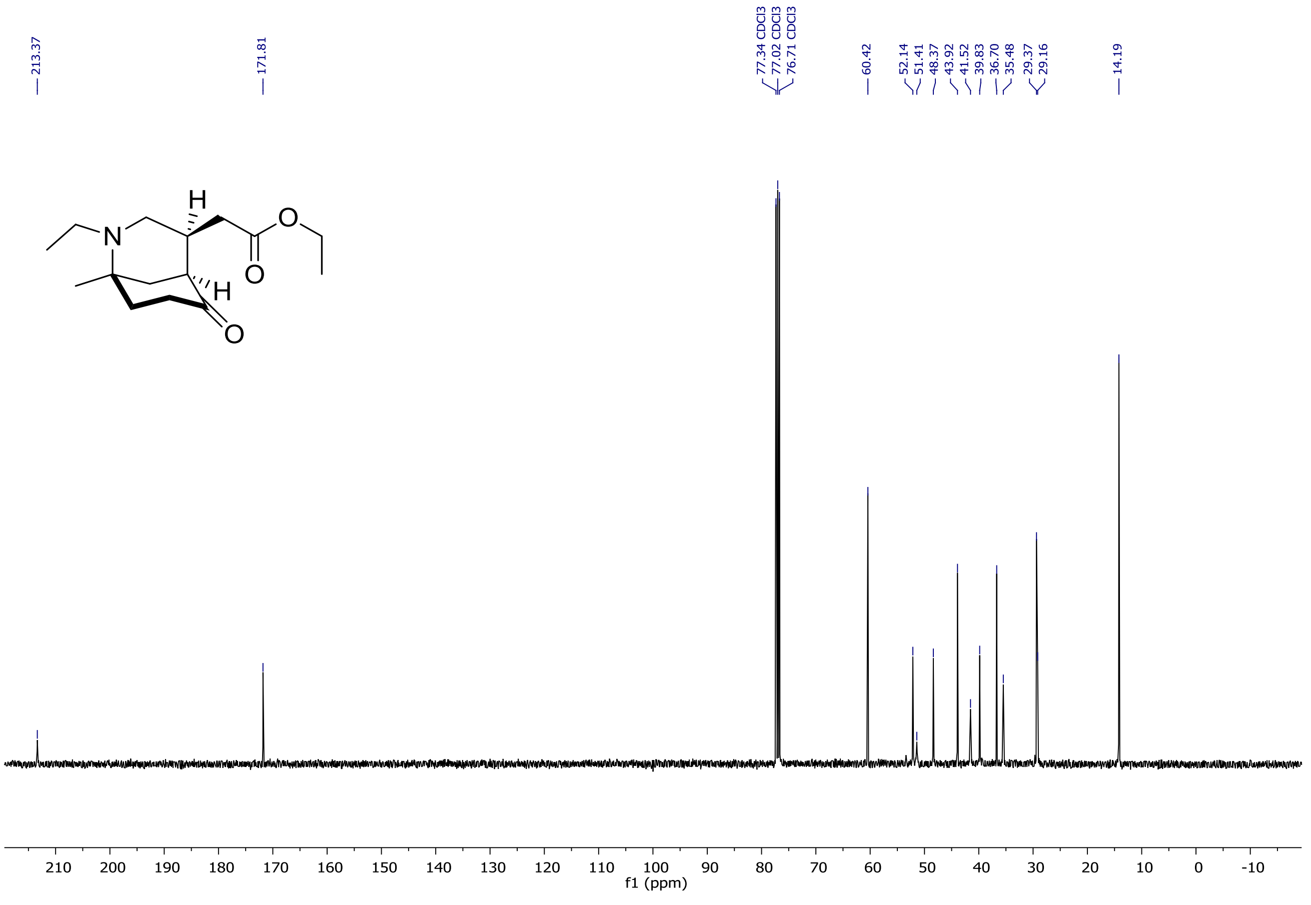

^{1}H NMR Spectrum of compound 3j

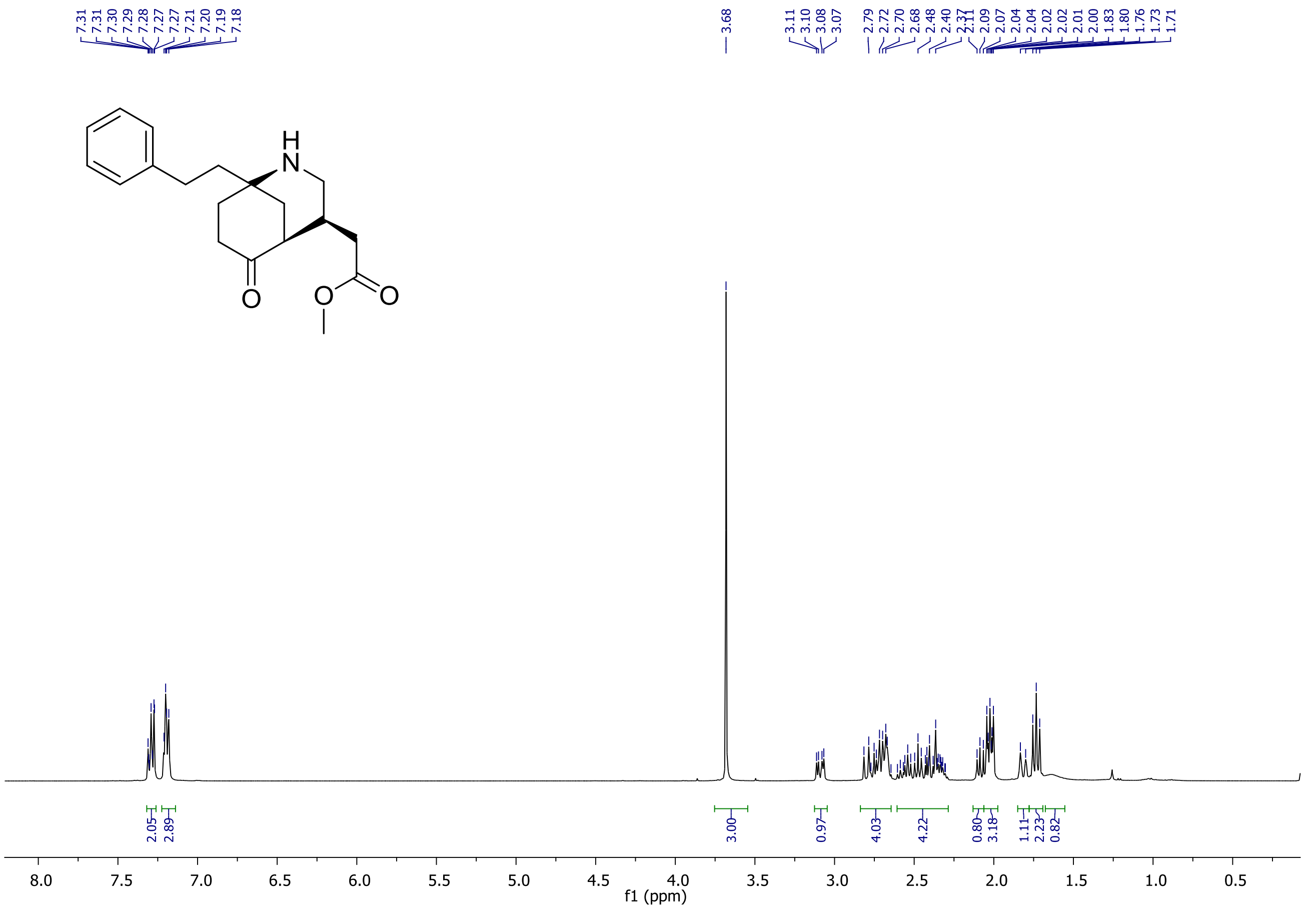

^{13}C NMR Spectrum of compound 3j

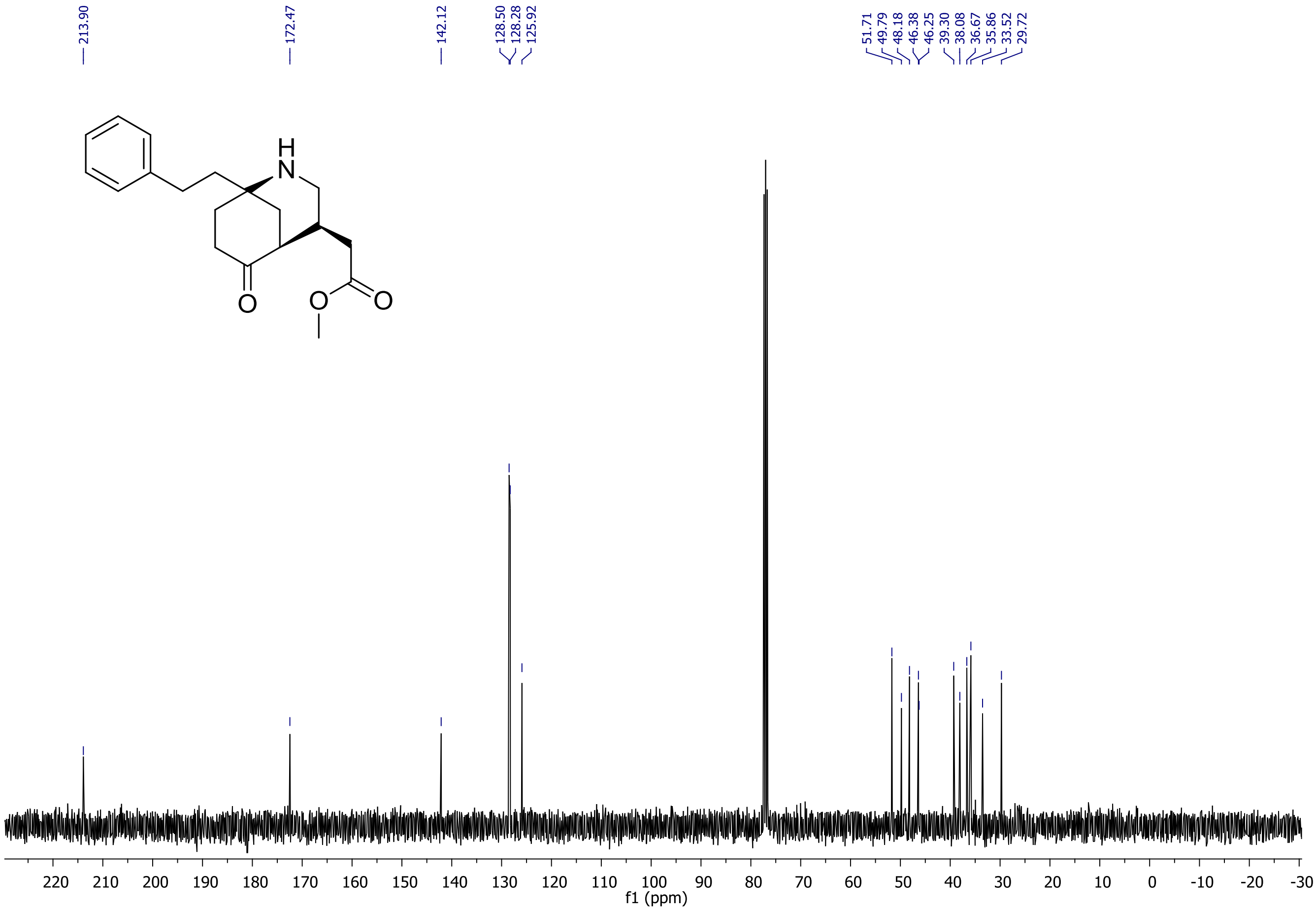

^{1}H NMR Spectrum of compound 3k

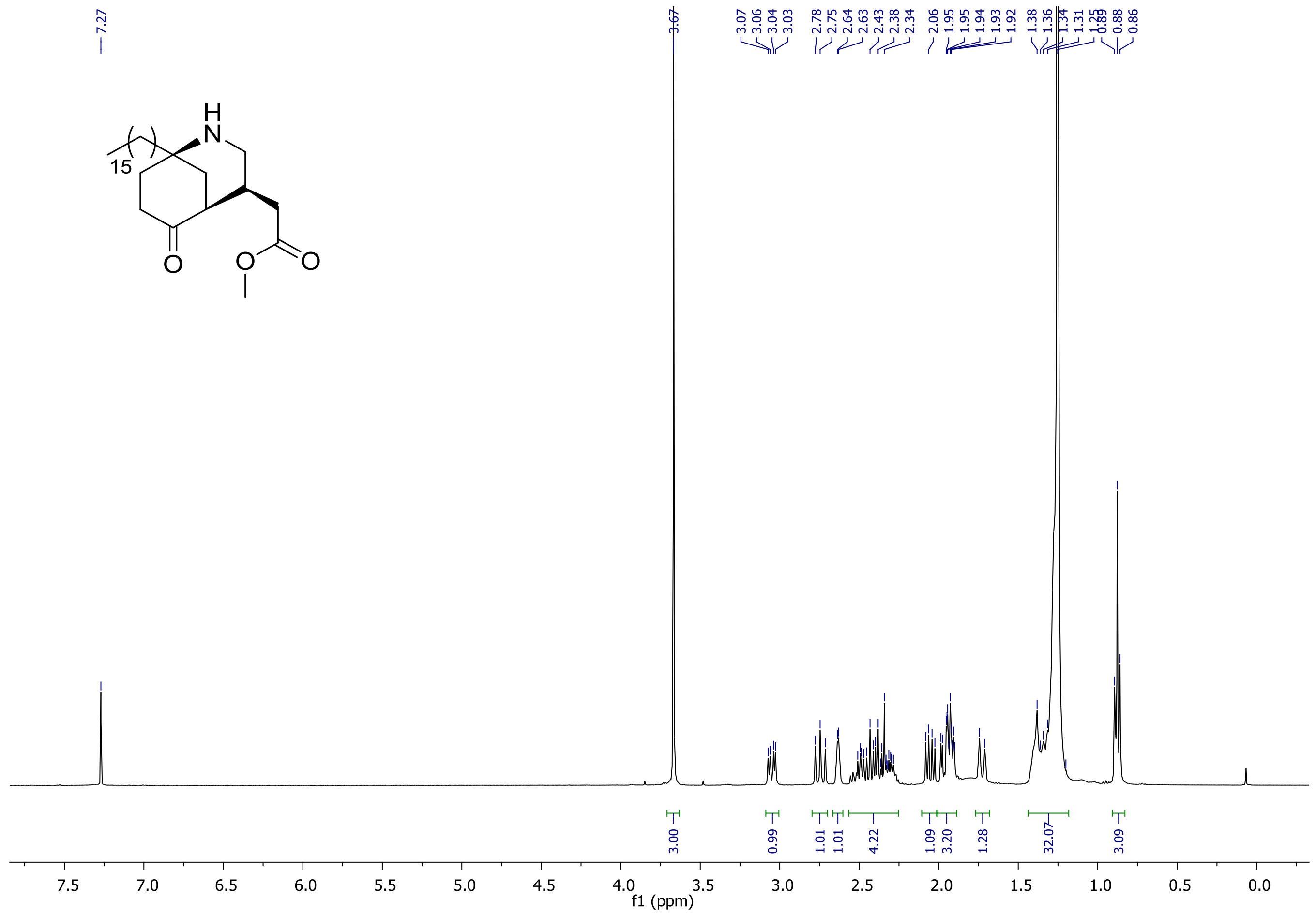

^{13}C NMR Spectrum of compound 3k

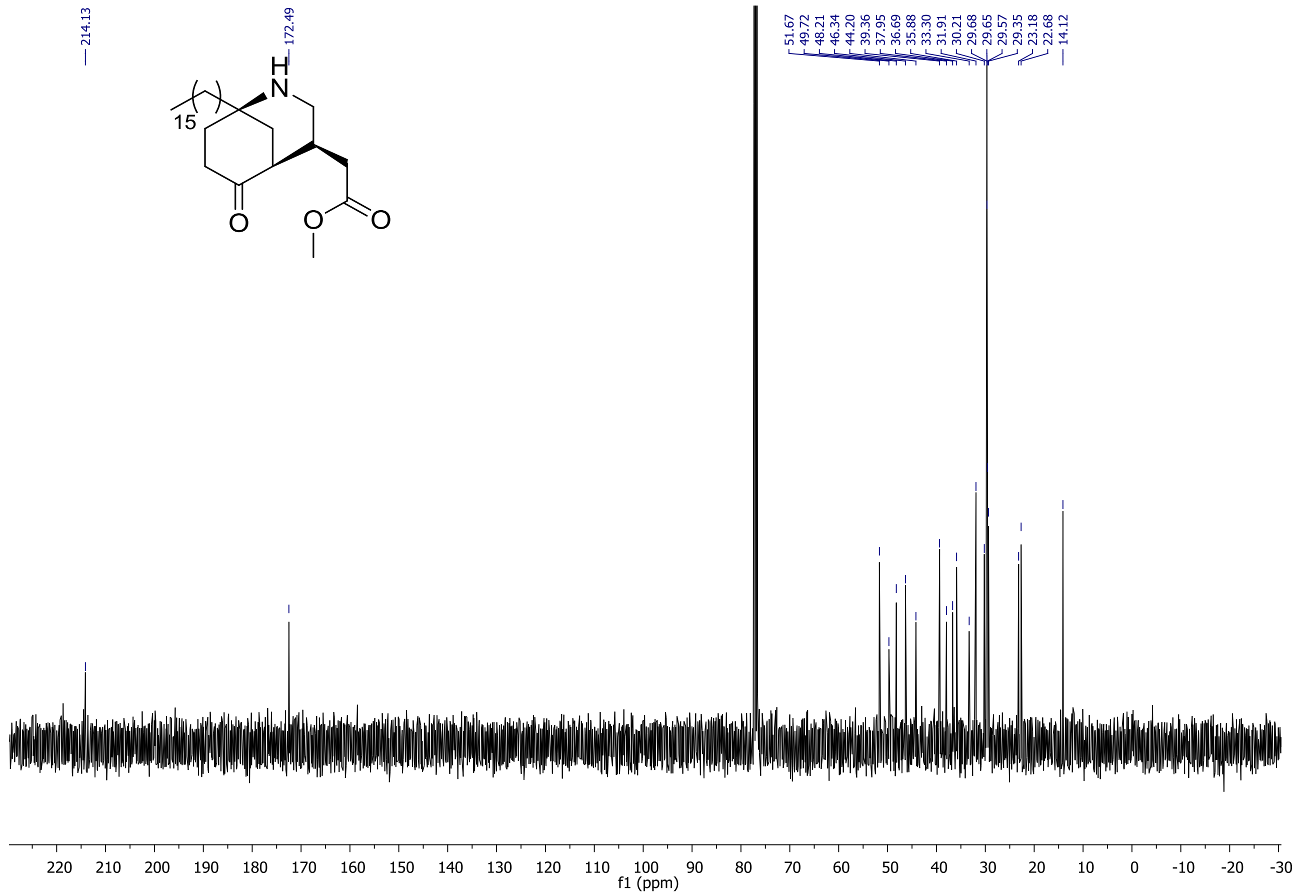

^{1}H NMR Spectrum of compound 3l

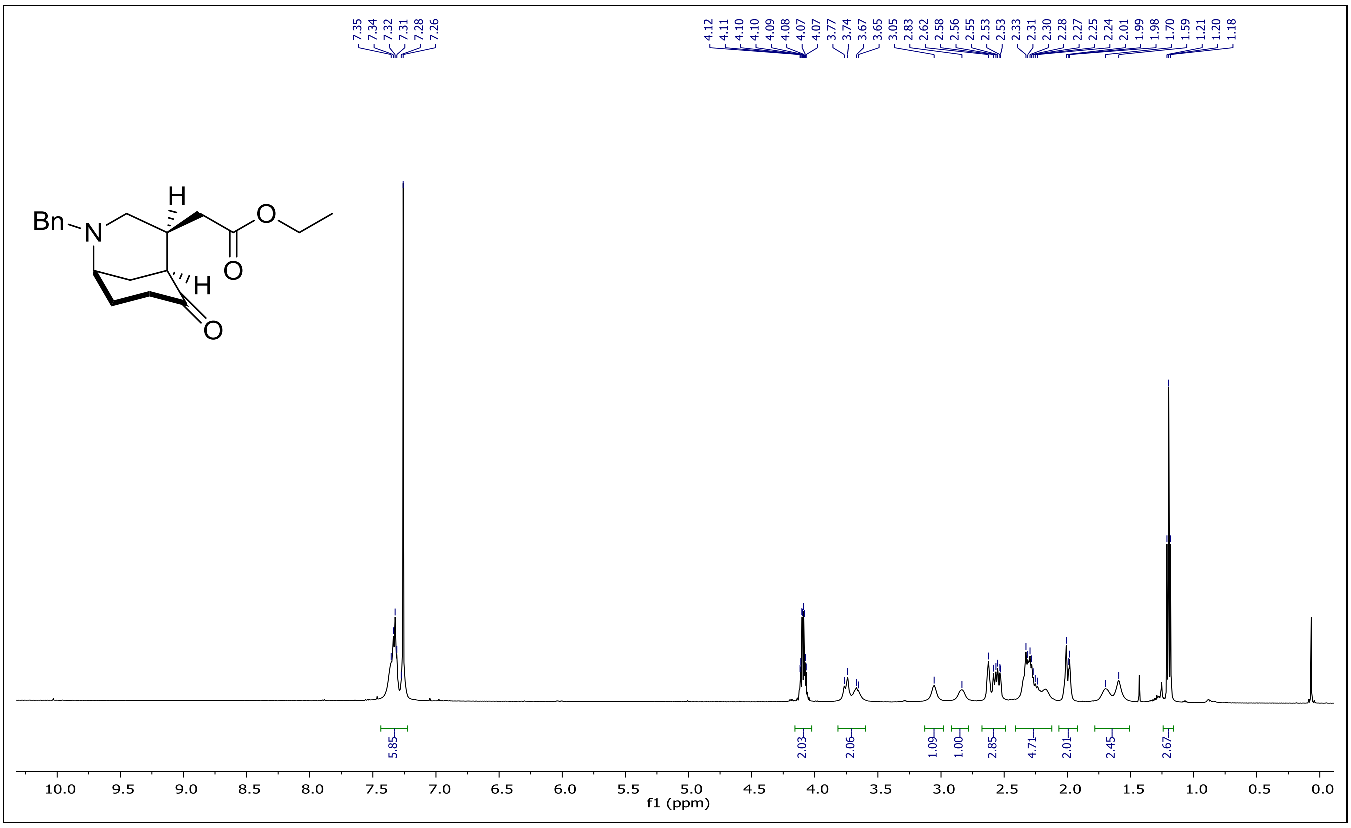

^{13}C NMR Spectrum of compound 3l

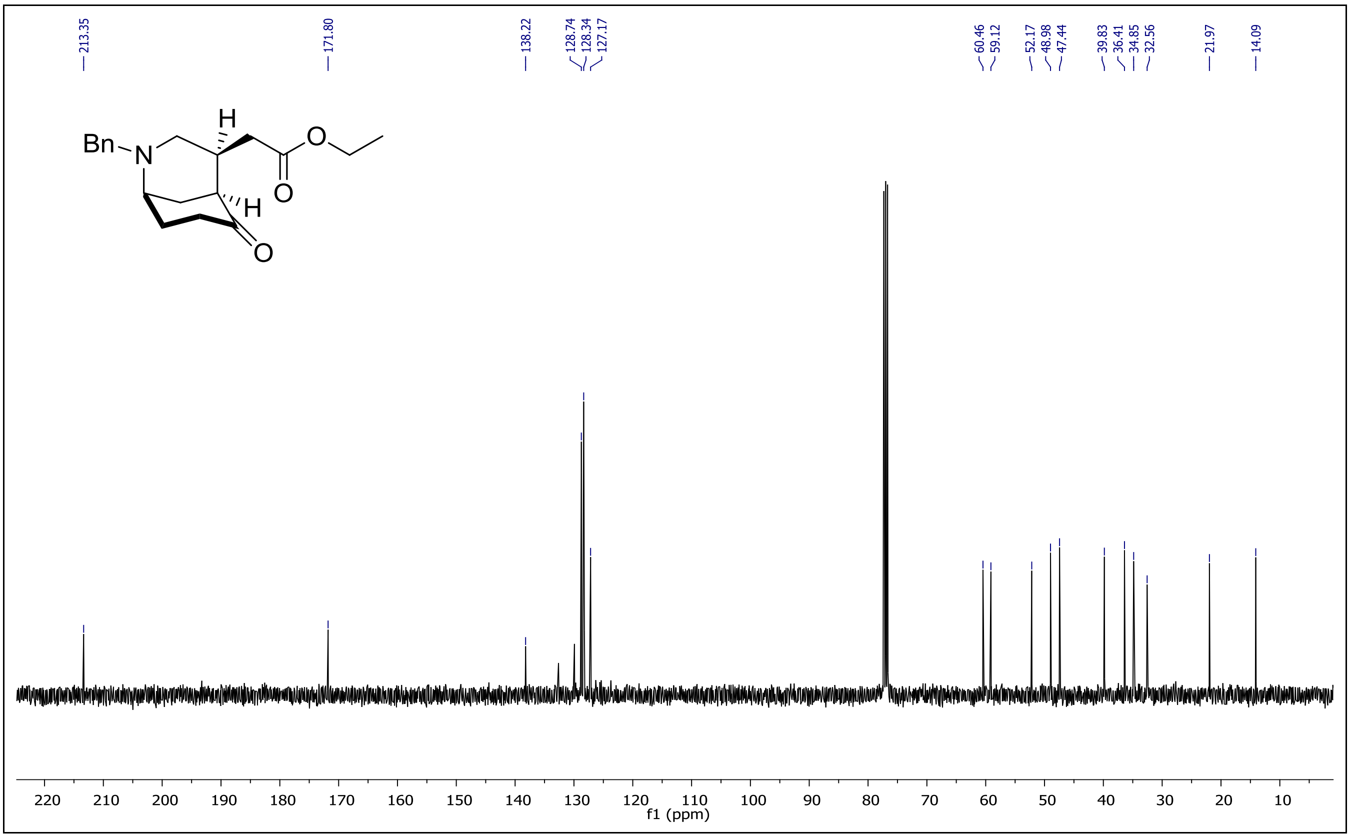

^{1}H NMR Spectrum of compound 3m

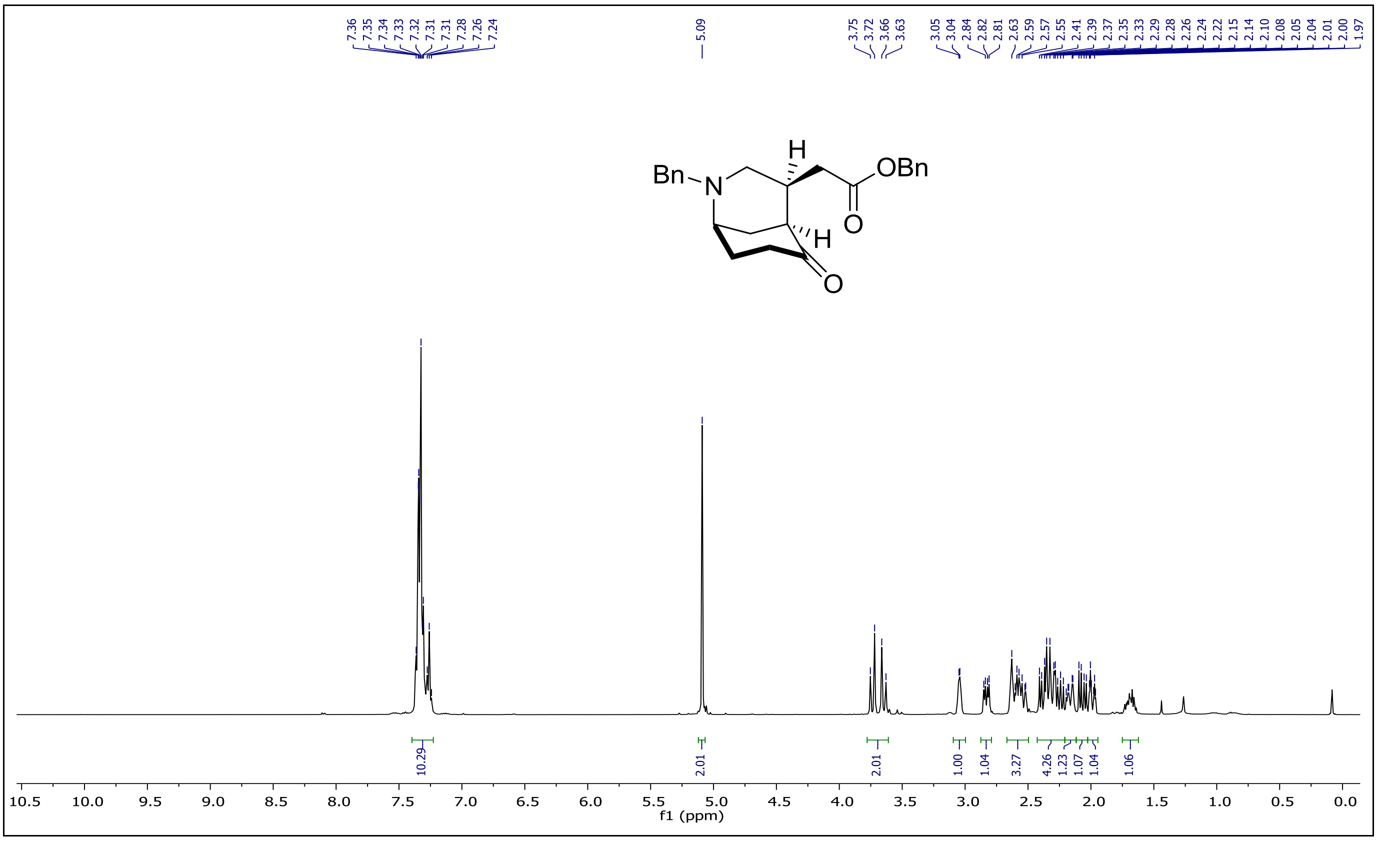

^{13}C NMR Spectrum of compound 3m

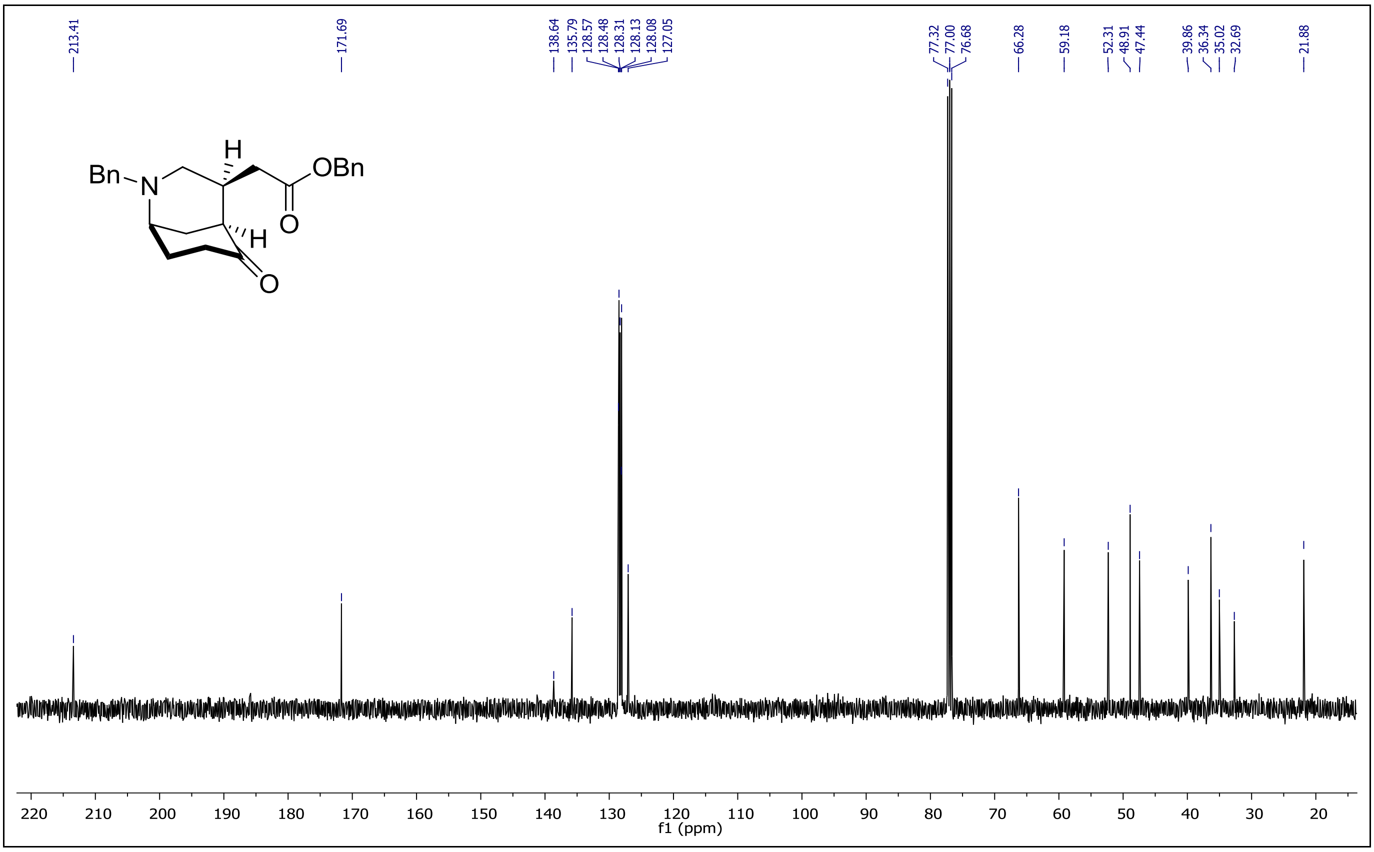

H NMR Spectrum of compound 3n

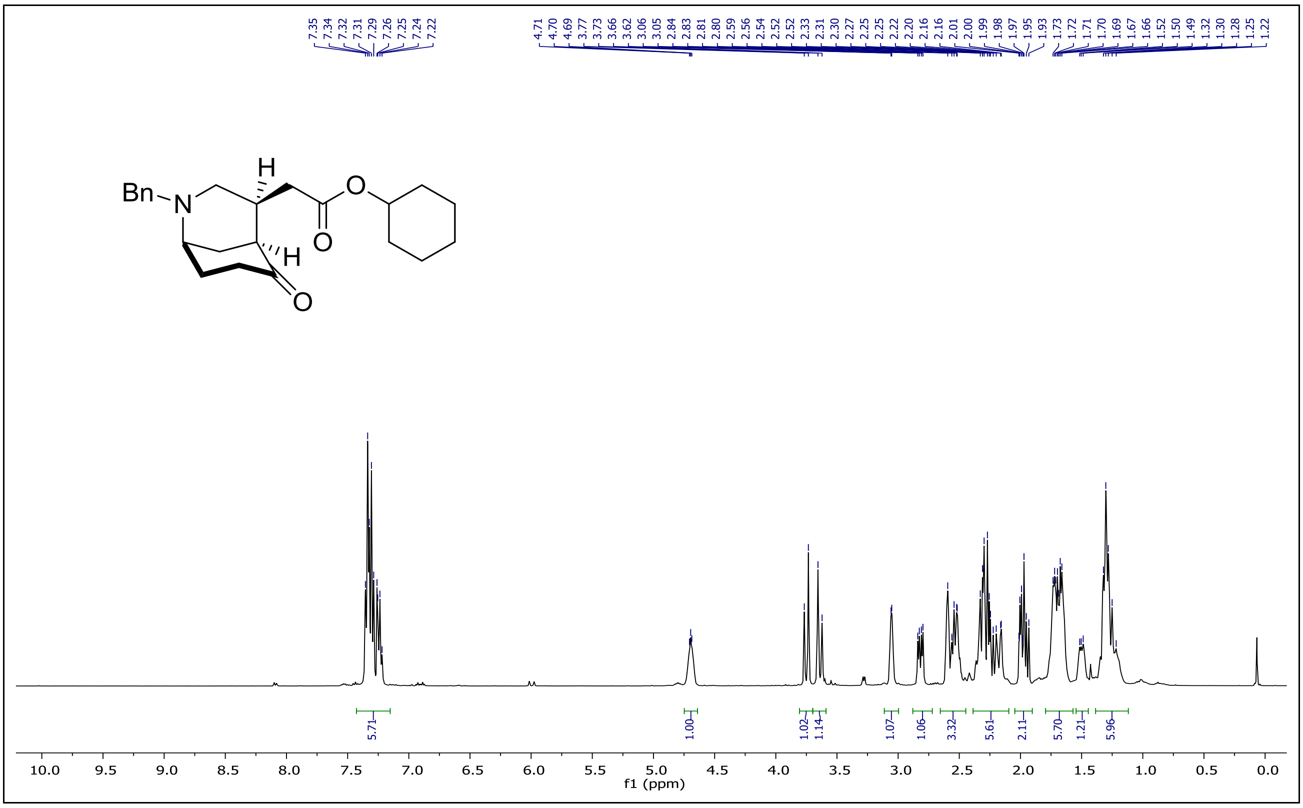

^{13}C NMR Spectrum of compound 3n

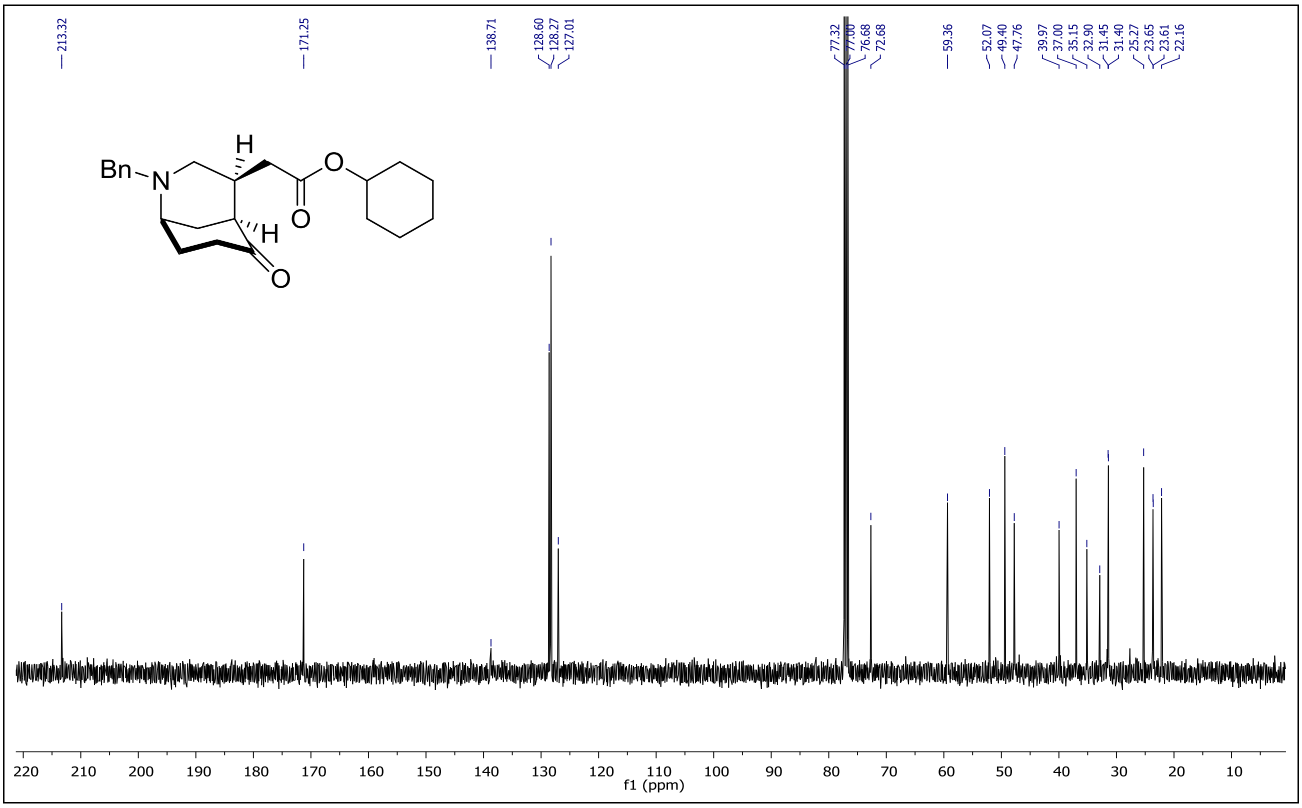

^{1}H NMR Spectrum of compound 3o

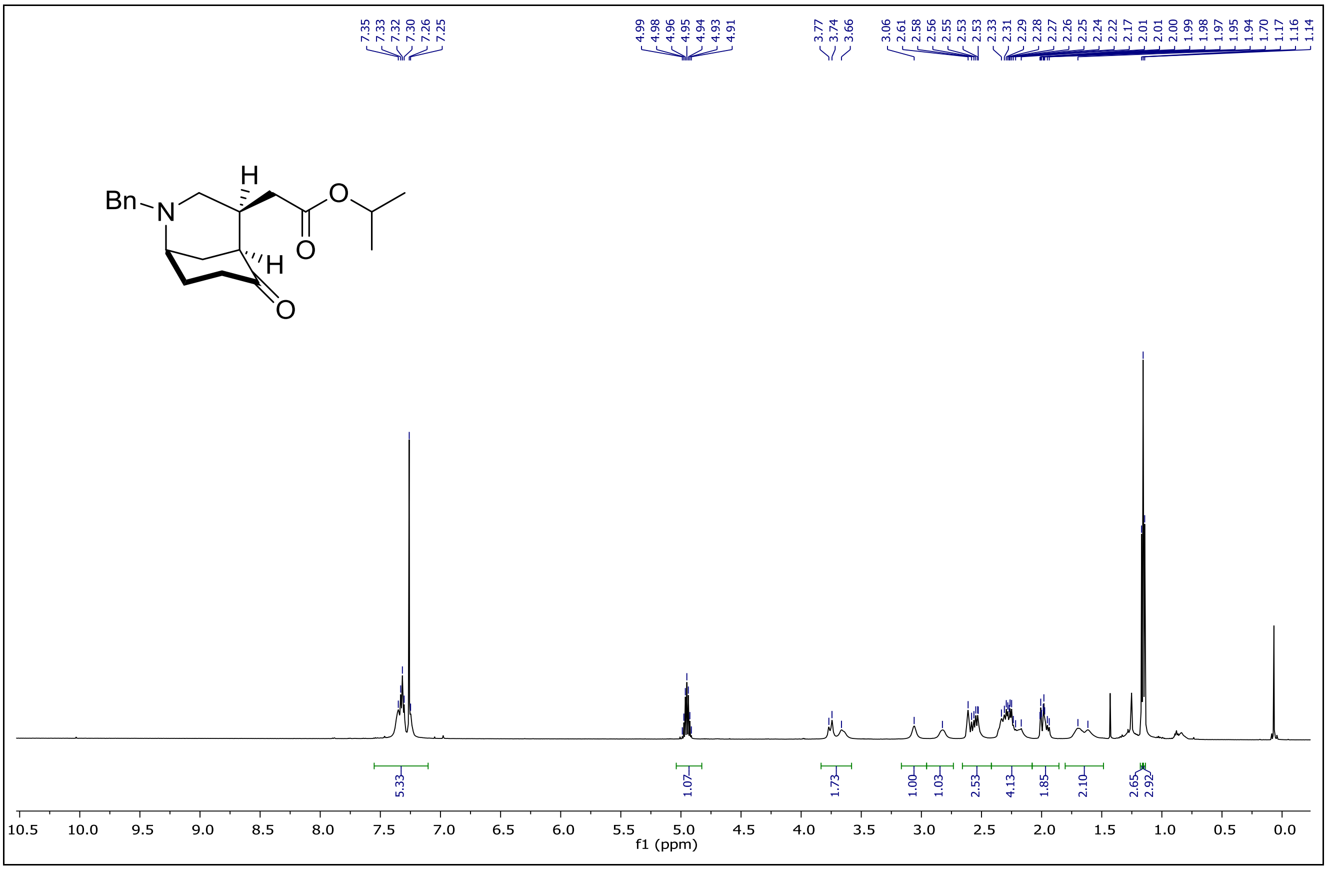

^{13}C NMR Spectrum of compound 3o

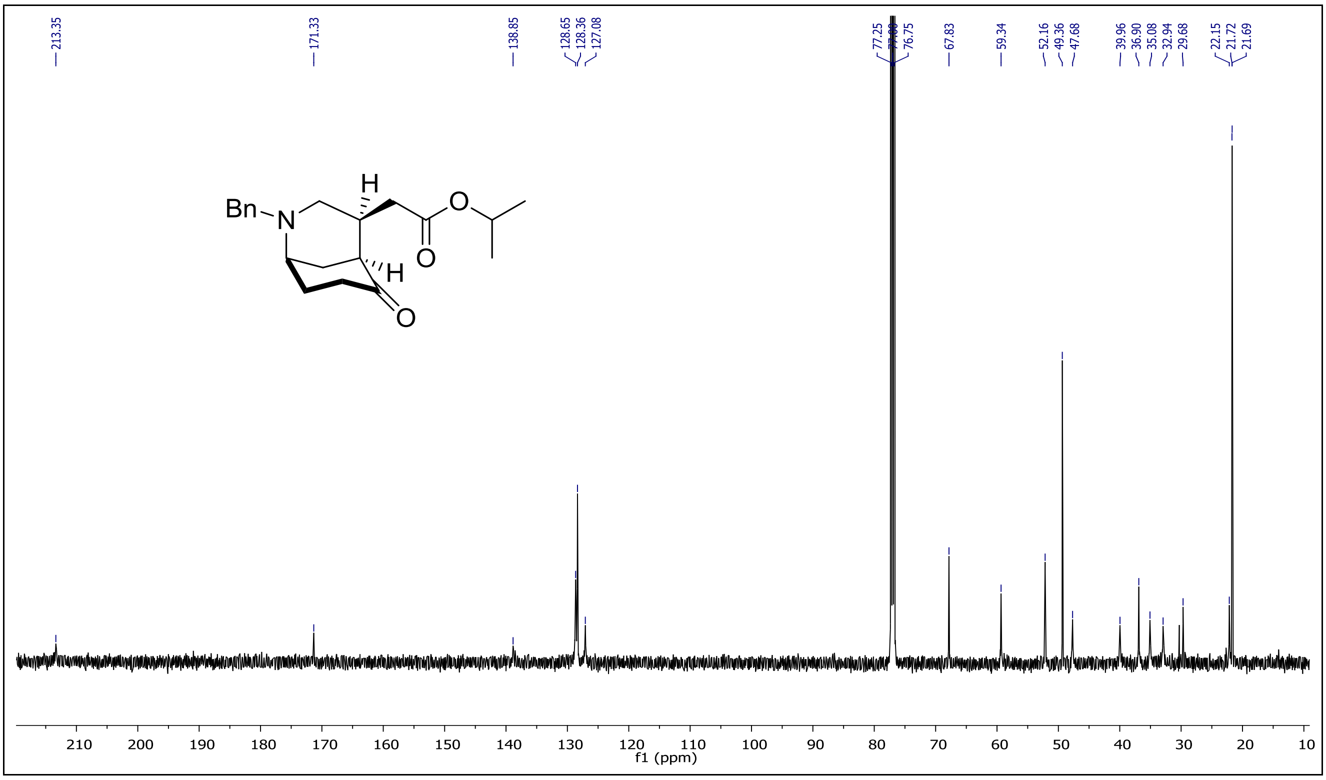

¹H NMR Spectrum of compound 3p

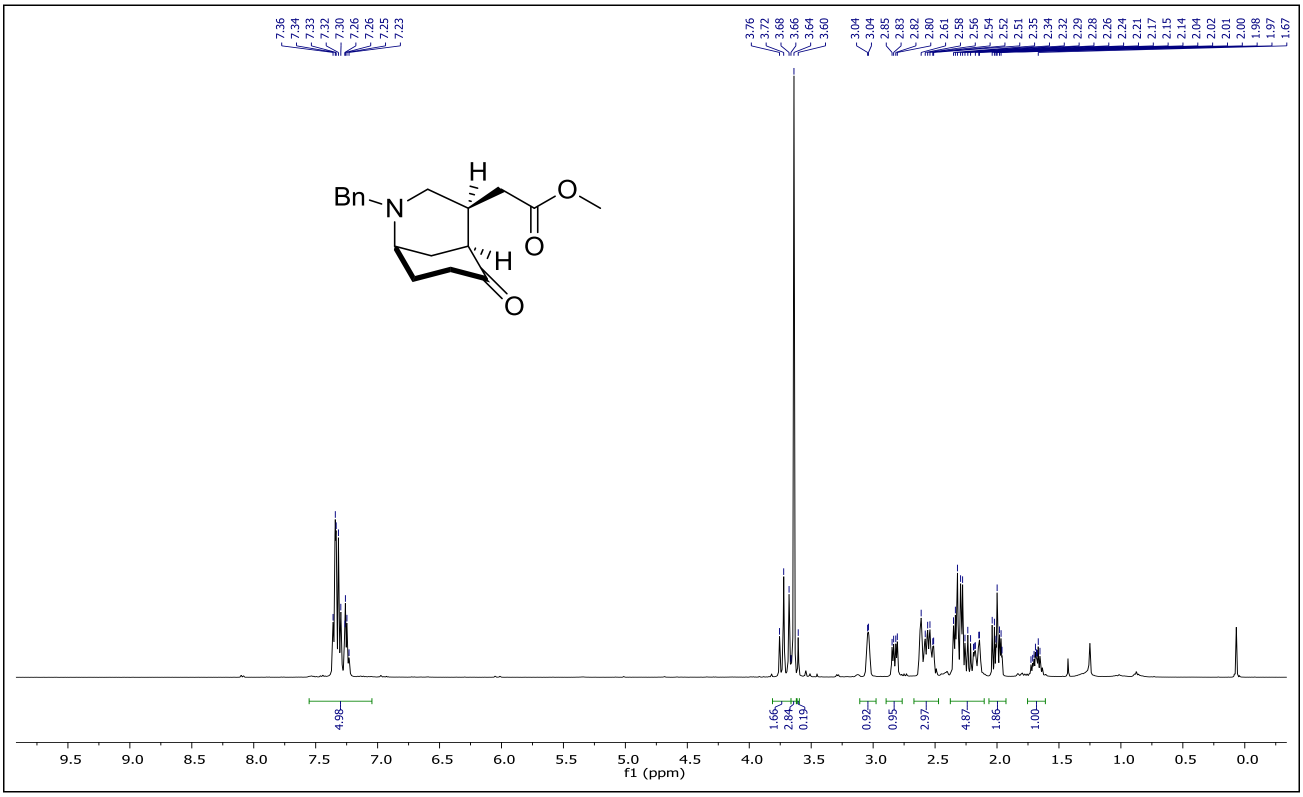

¹³C NMR Spectrum of compound 3p

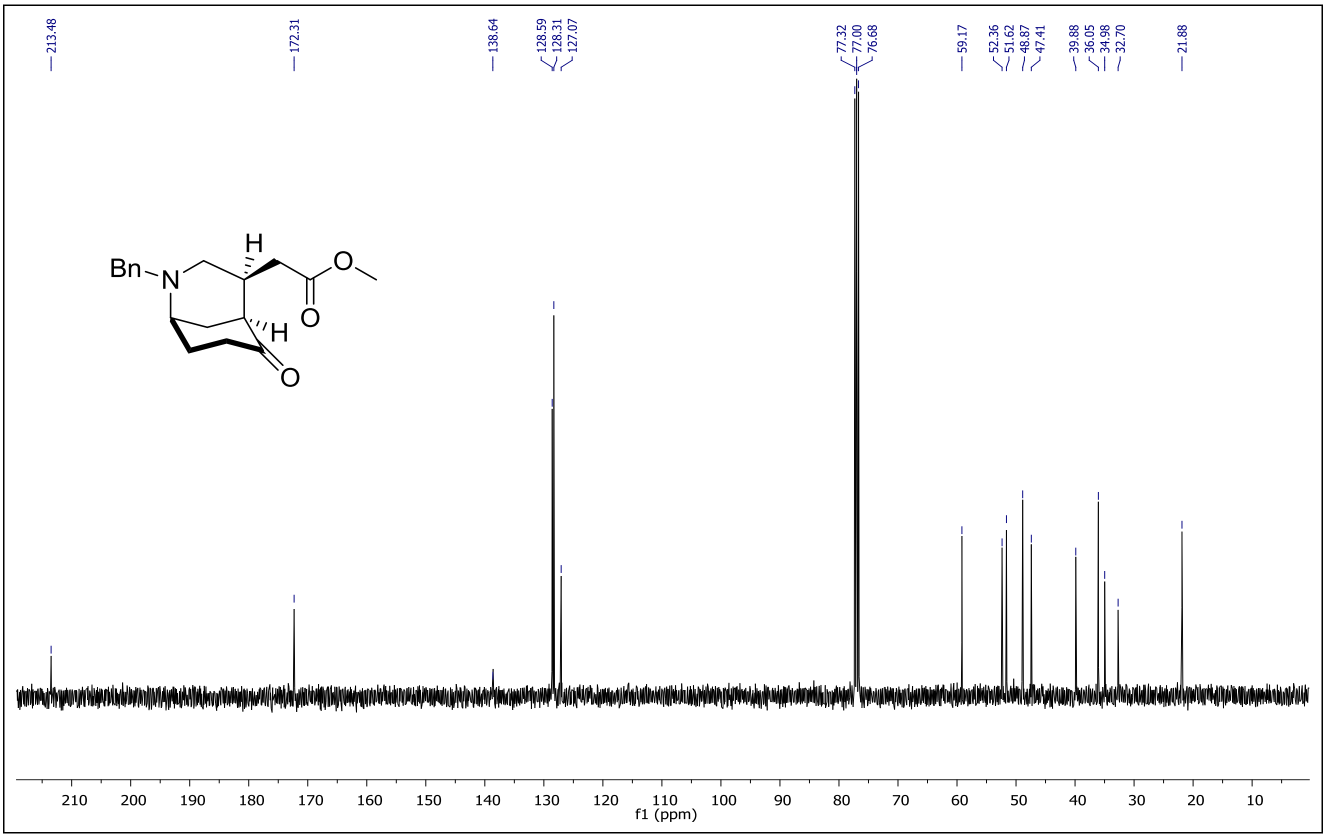

^{1}H NMR Spectrum of compound 3q

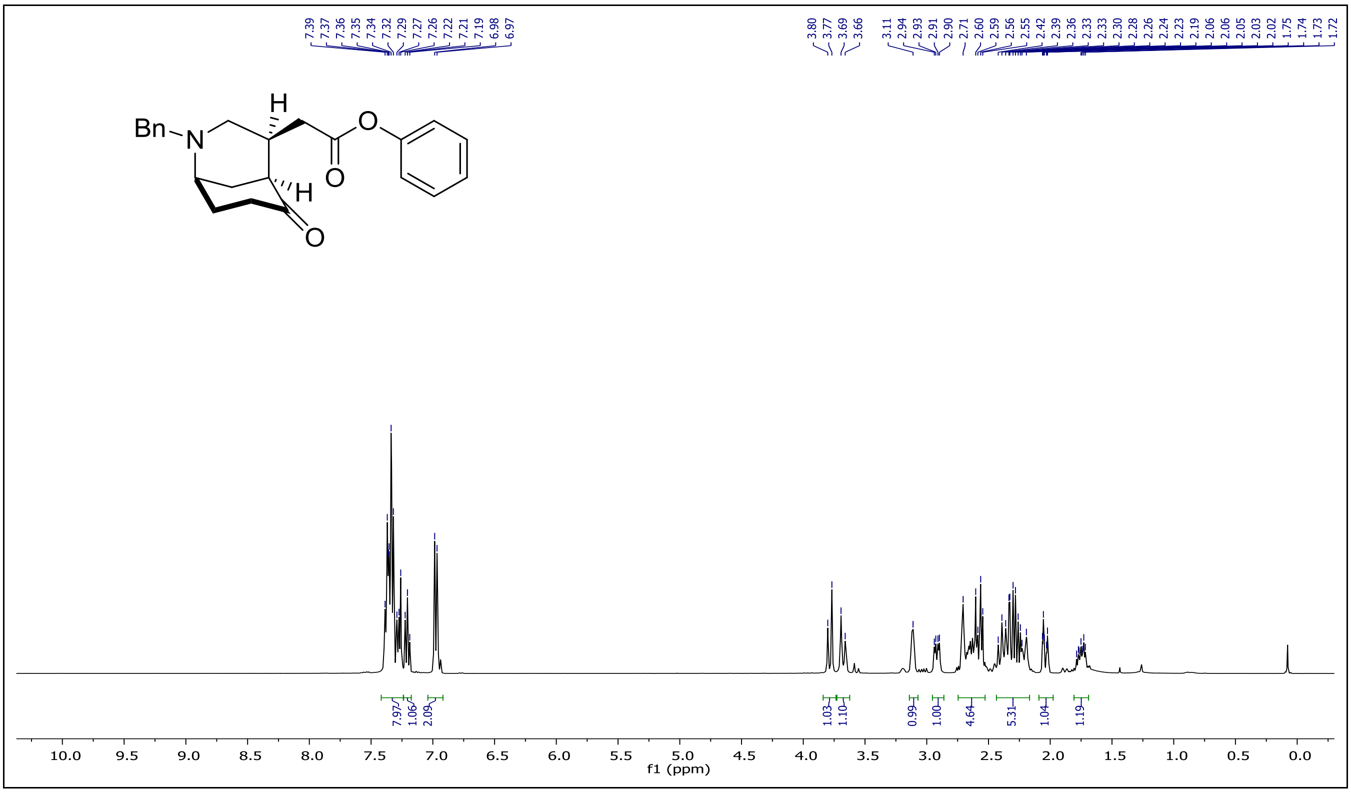

^{13}C NMR Spectrum of compound 3q

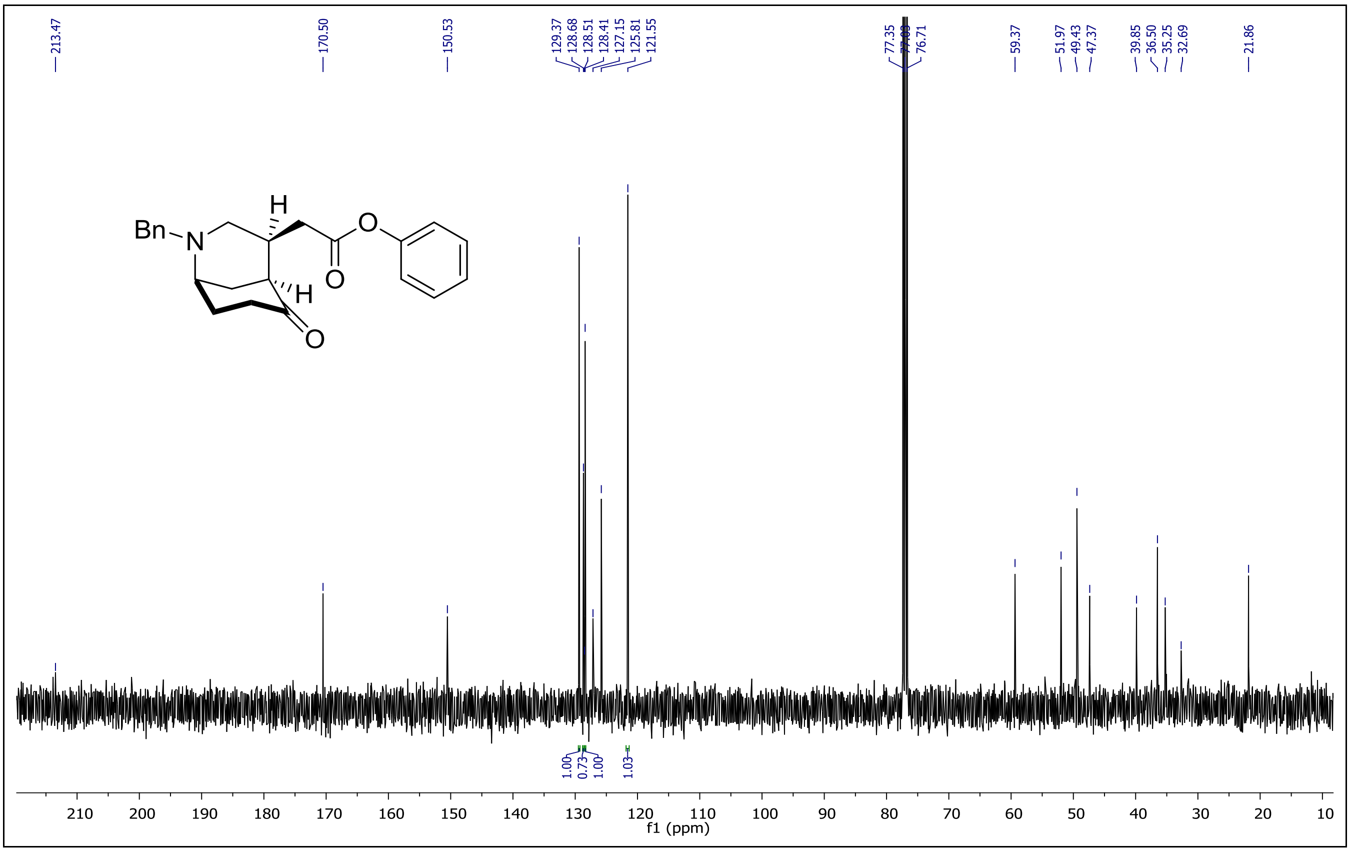

^{1}H NMR Spectrum of compound 3r

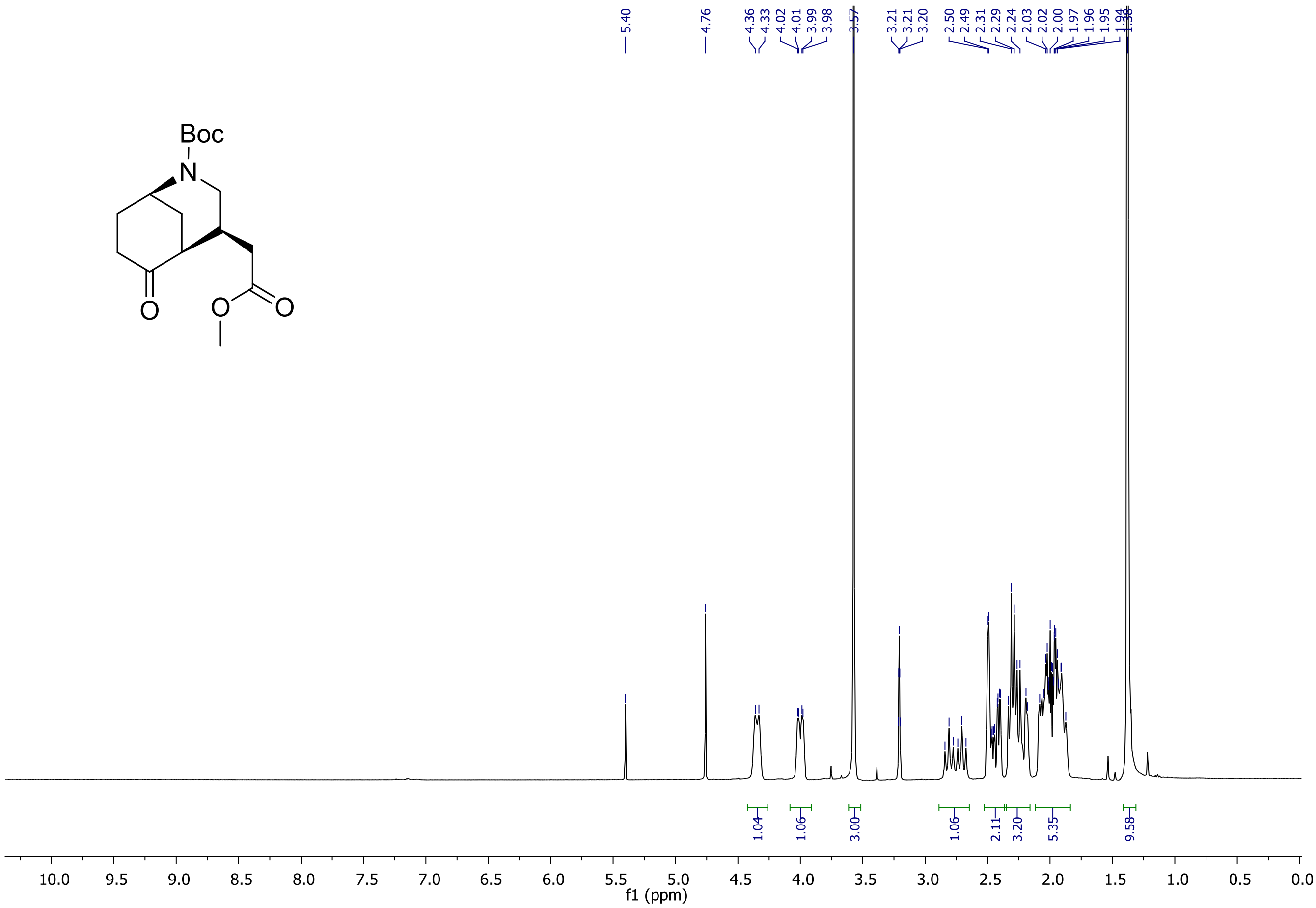

^{13}C NMR Spectrum of compound 3r

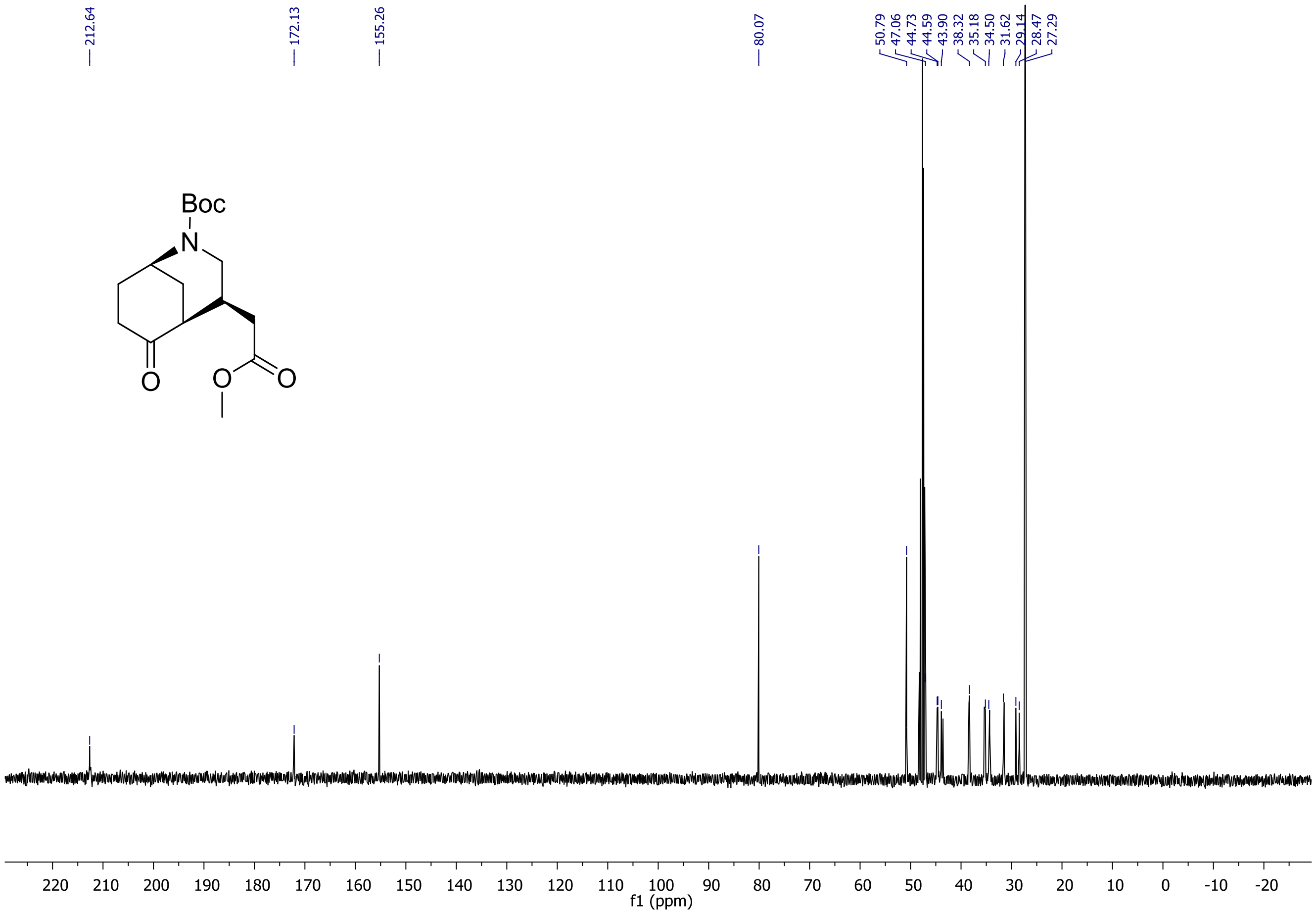

^{1}H NMR Spectrum of compound 3s

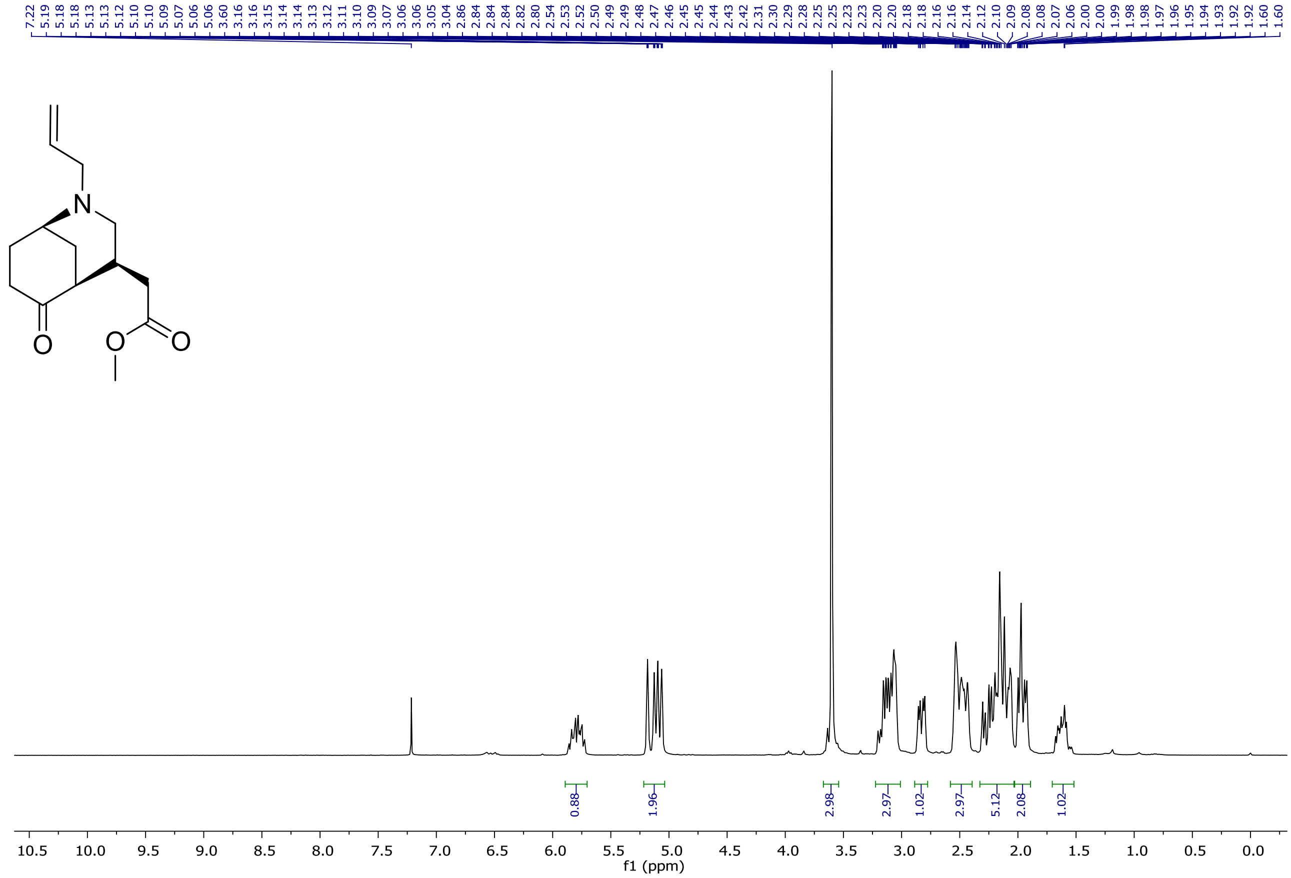

^{13}C NMR Spectrum of compound 3s

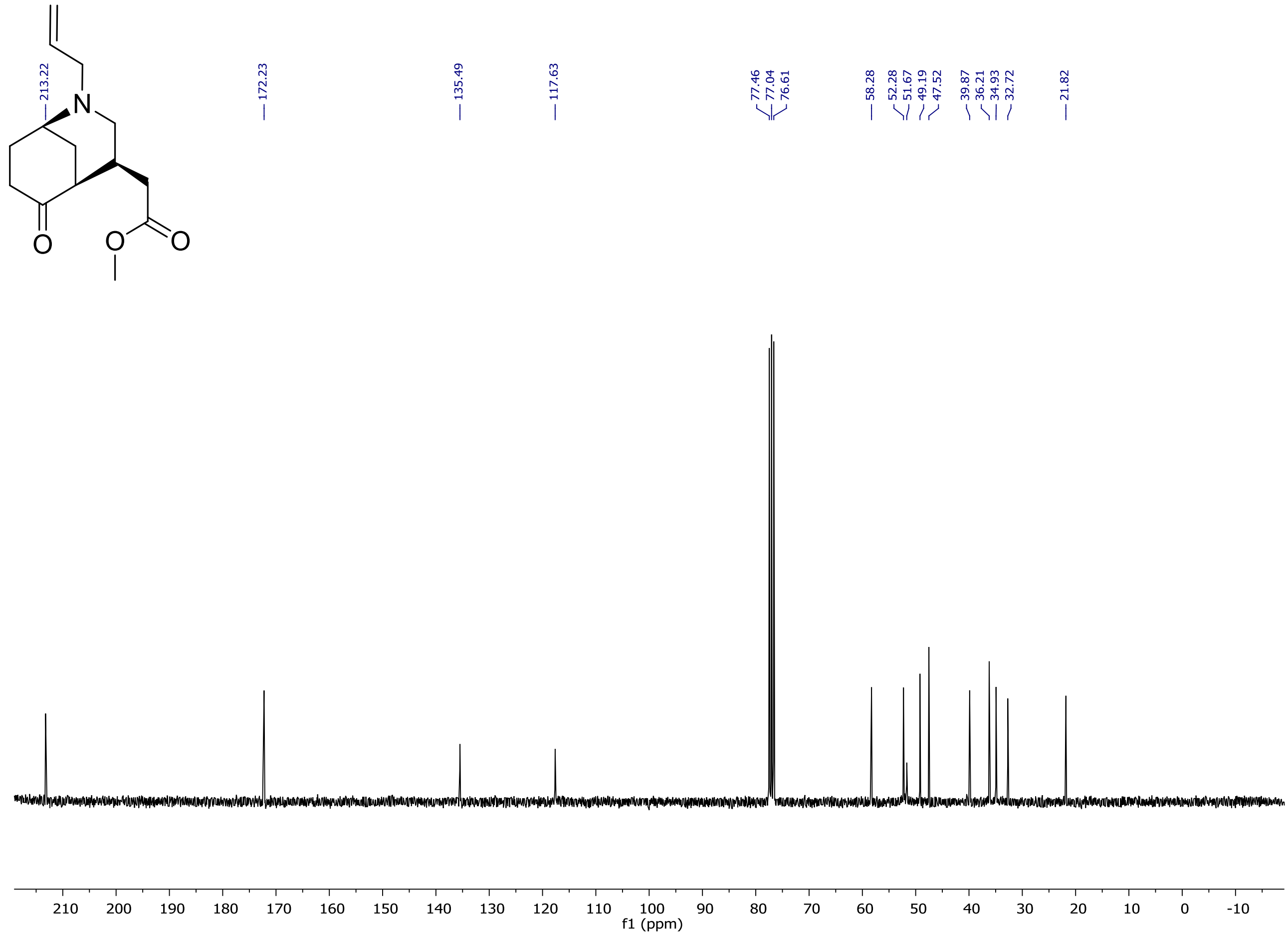

^{1}H NMR Spectrum of compound 3t

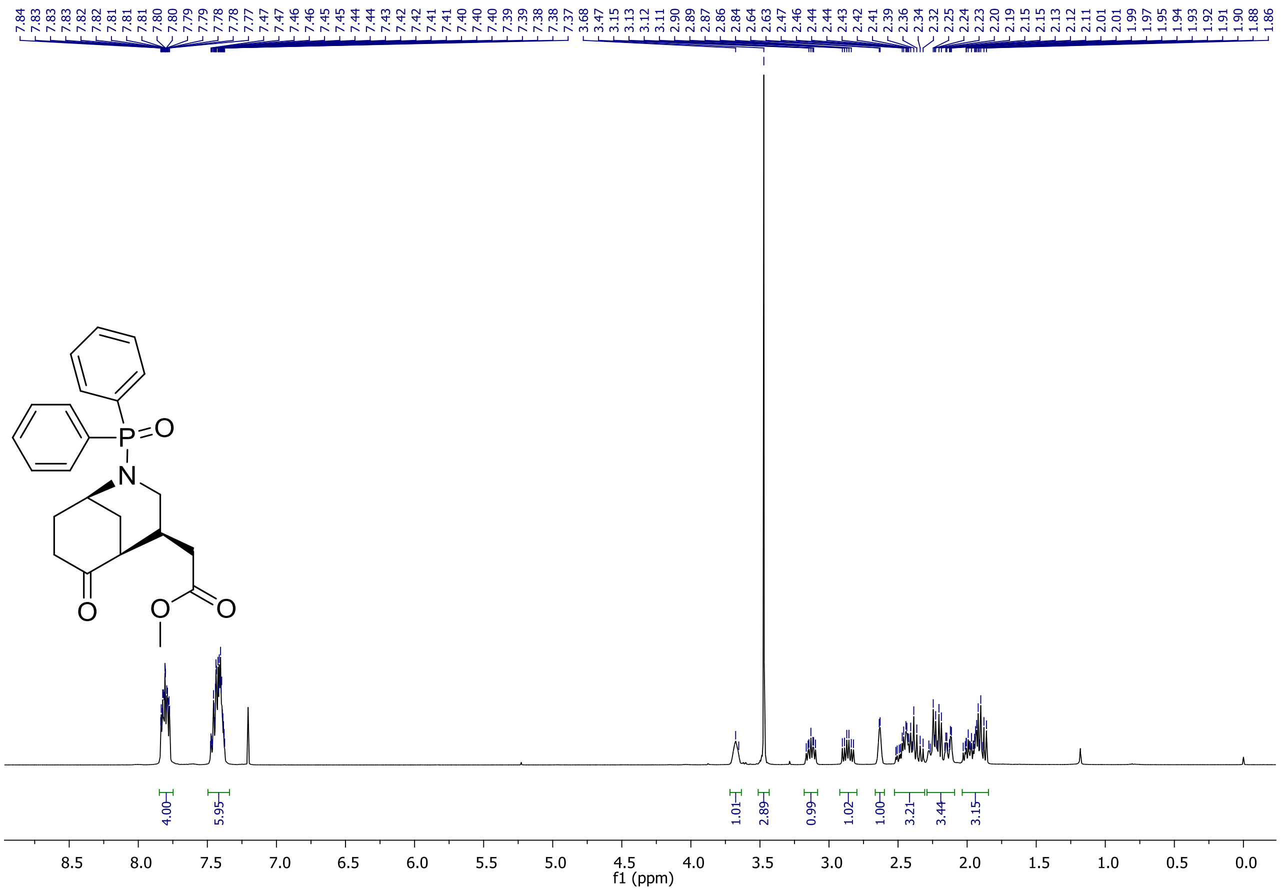

^{13}C NMR Spectrum of compound 3t

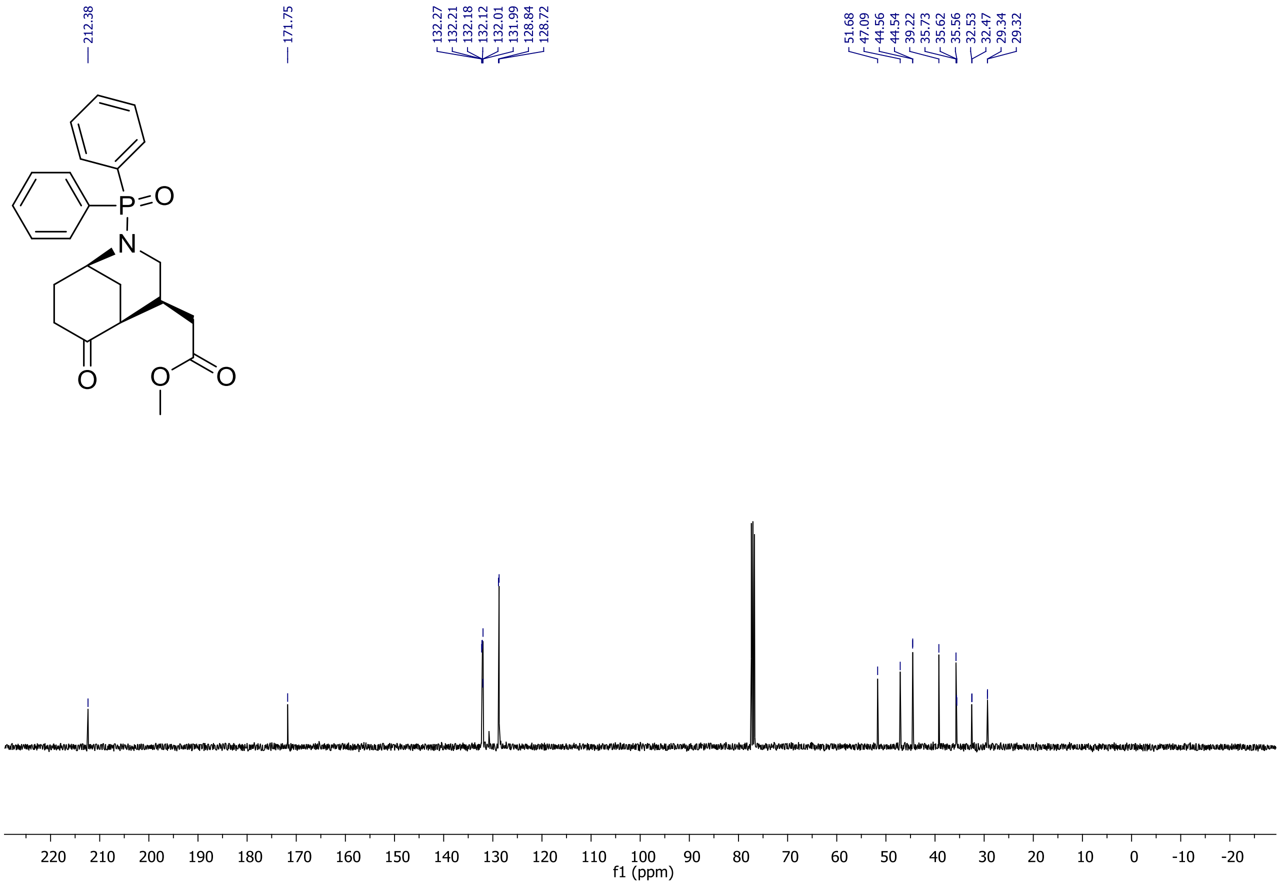

^{1}H NMR Spectrum of compound 3u

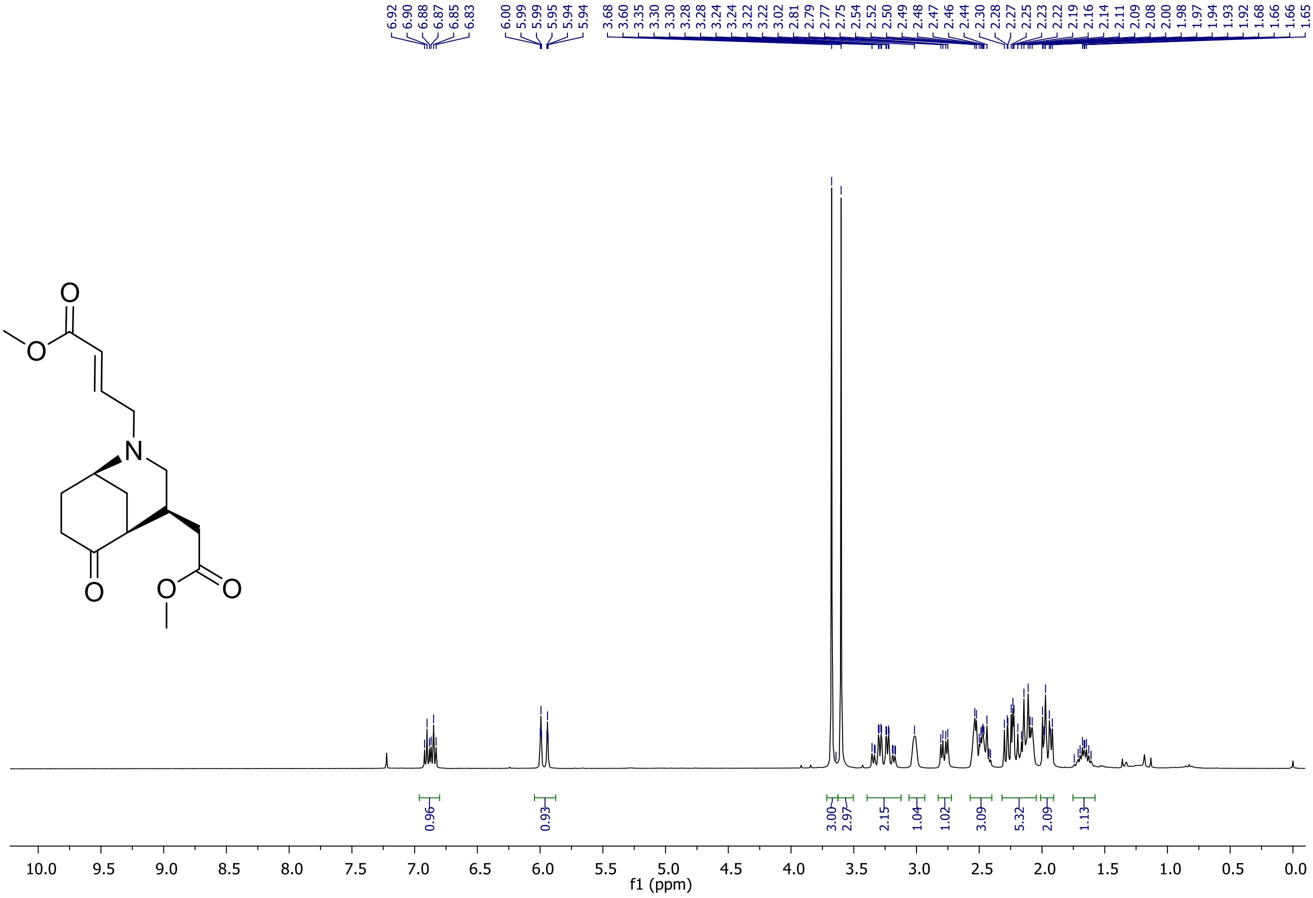

^{13}C NMR Spectrum of compound 3u

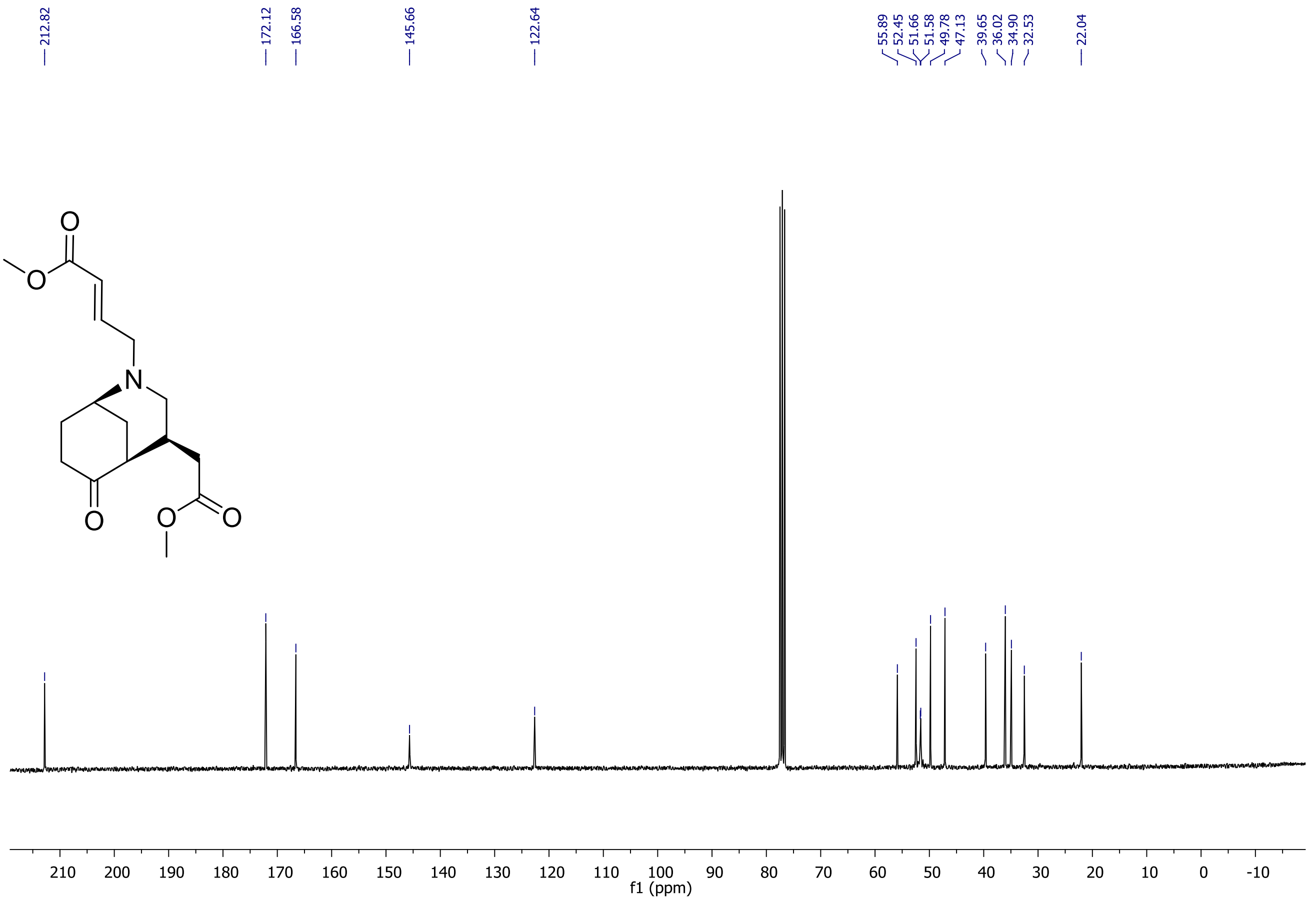

^{1}H NMR Spectrum of compound 3v

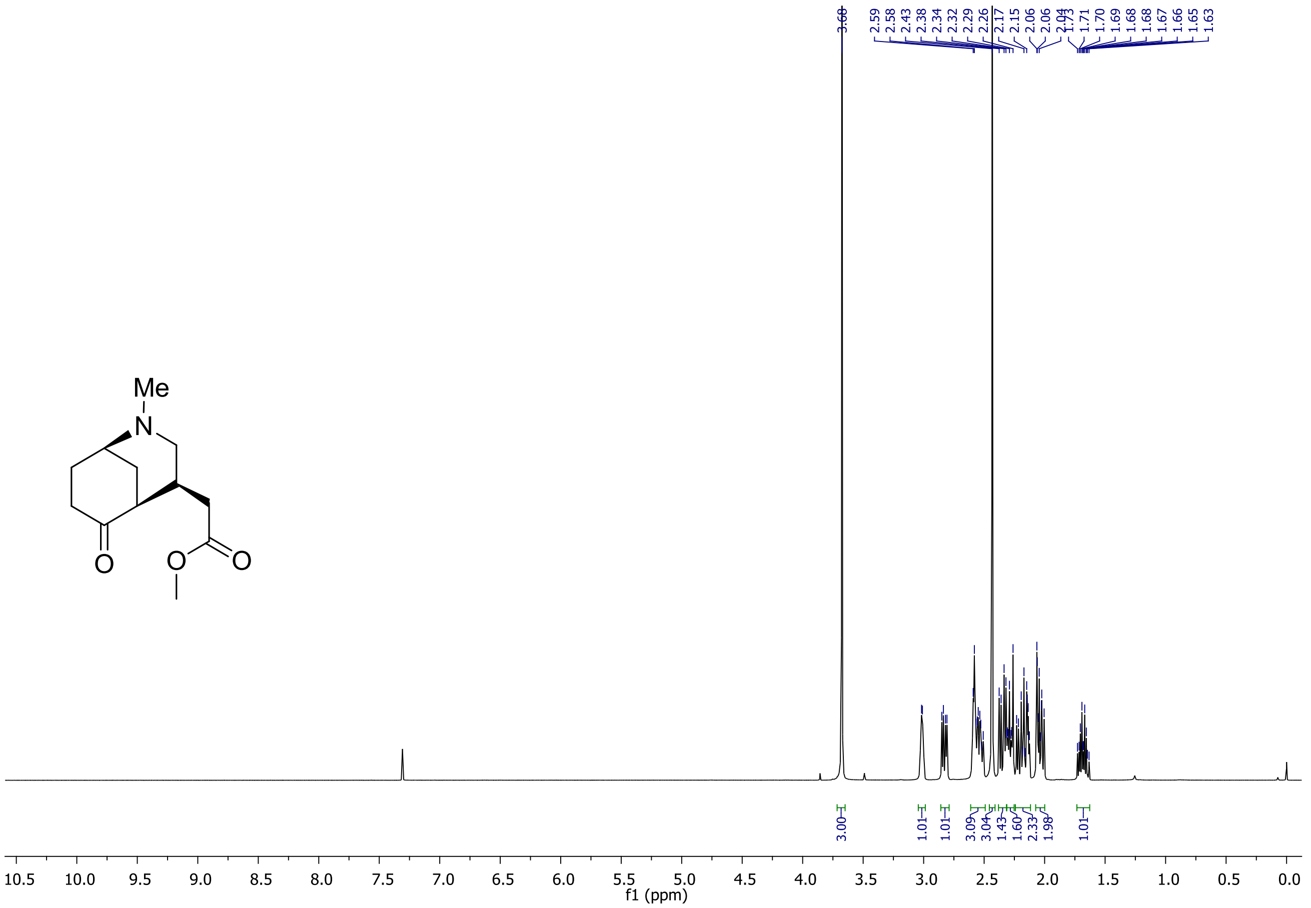

^{13}C NMR Spectrum of compound 3v

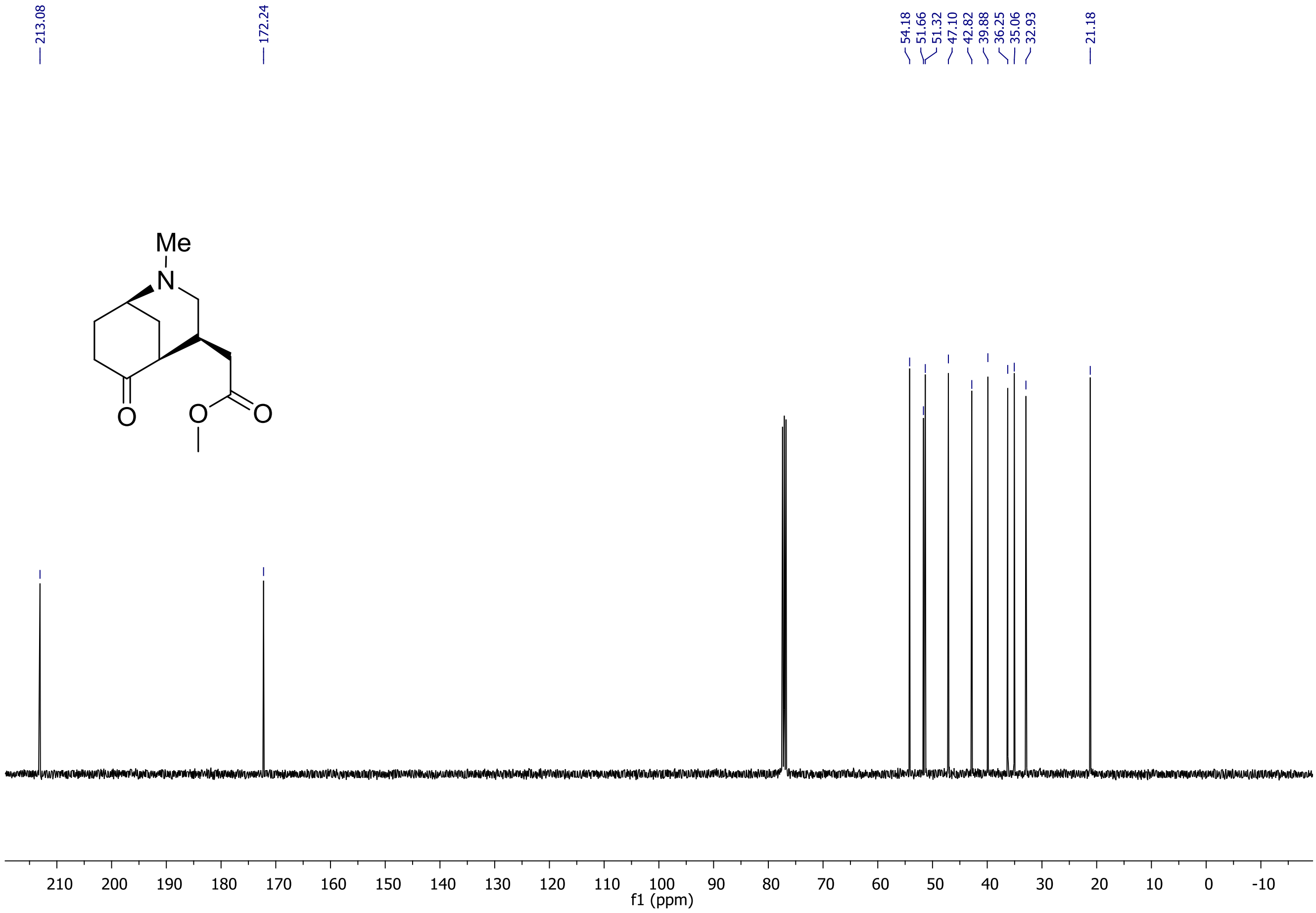

^{1}H NMR Spectrum of compound 35

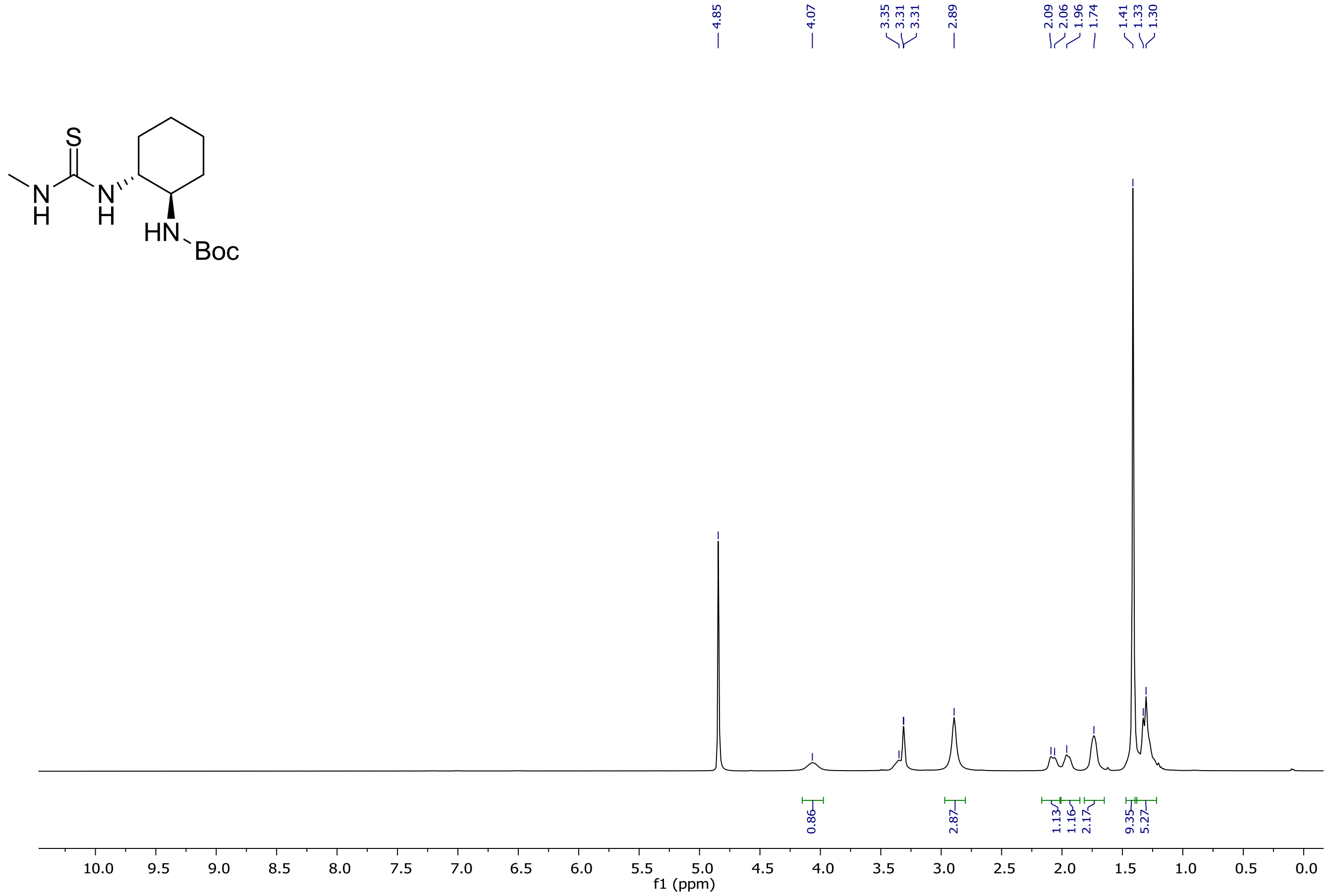

^{13}C NMR Spectrum of compound 35

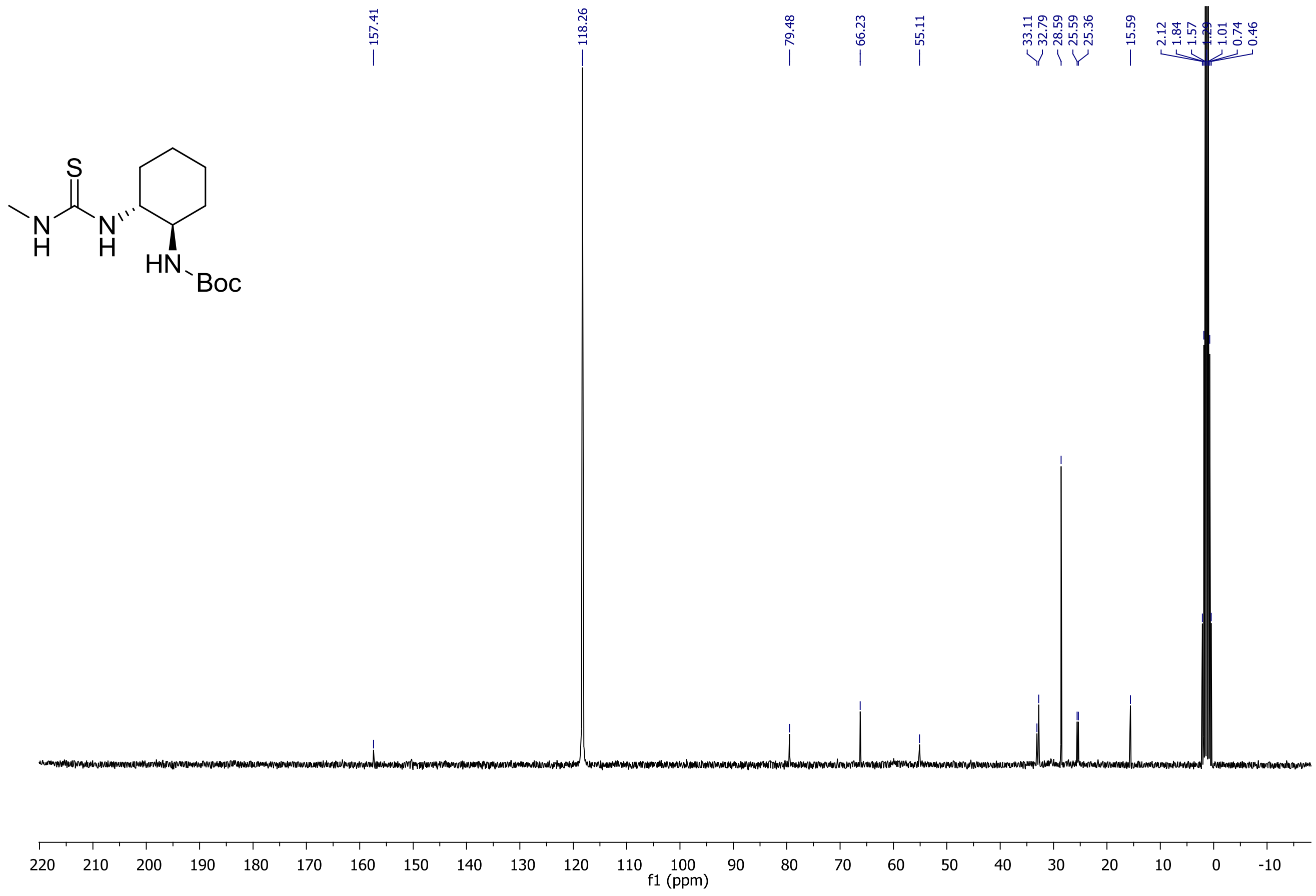

^{1}H NMR Spectrum of compound 36

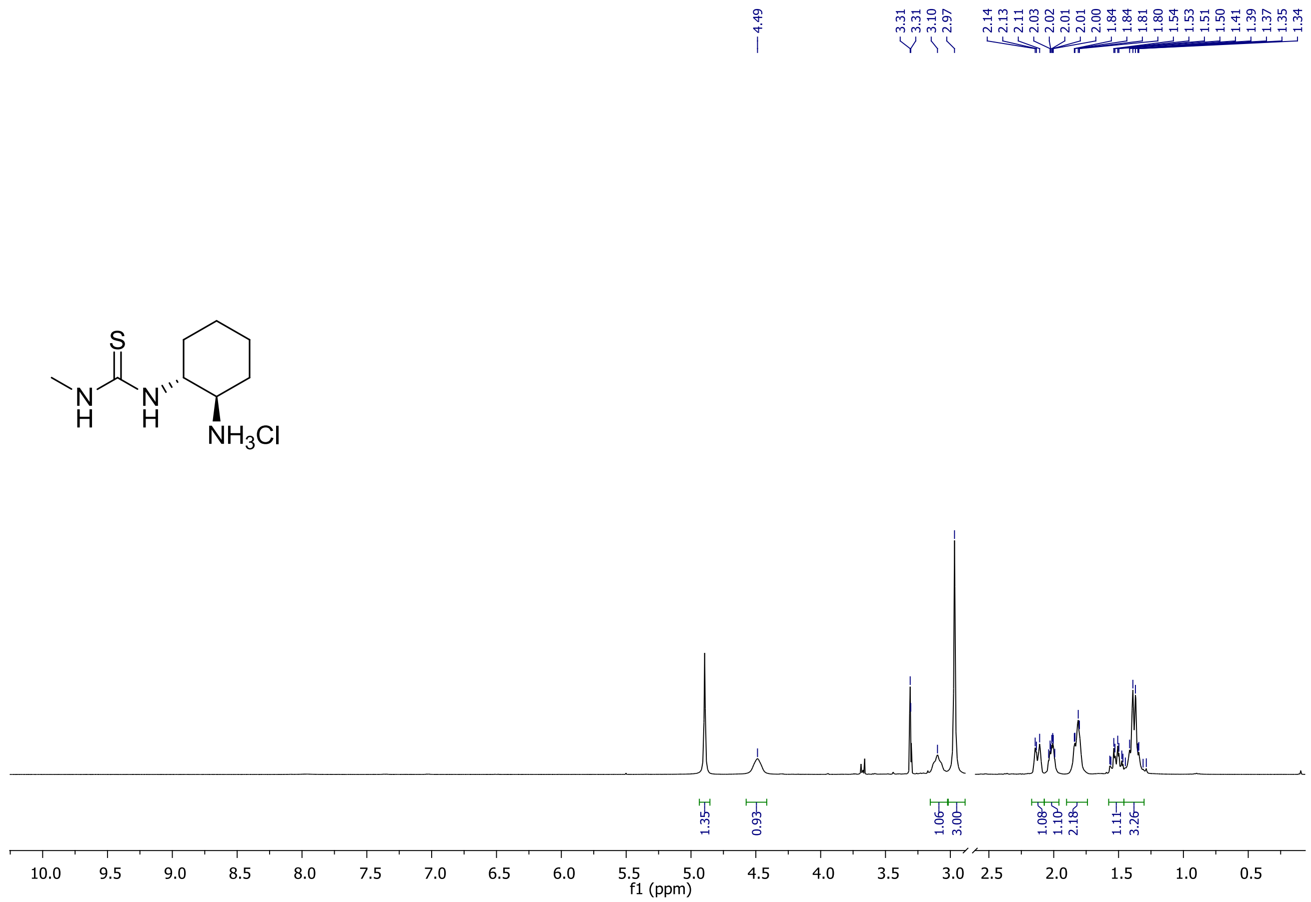

^{13}C NMR Spectrum of compound 36

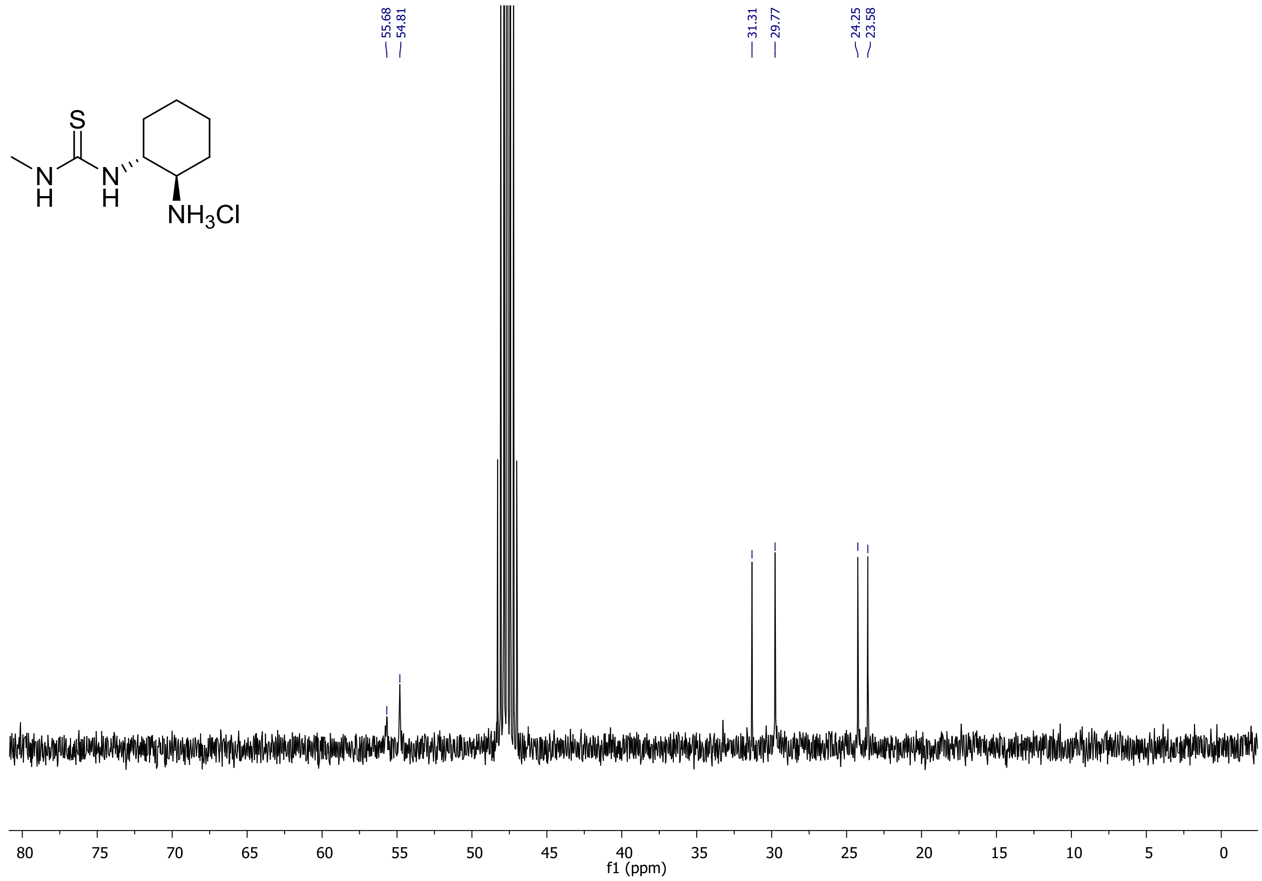

^{1}H NMR Spectrum of compound 4l

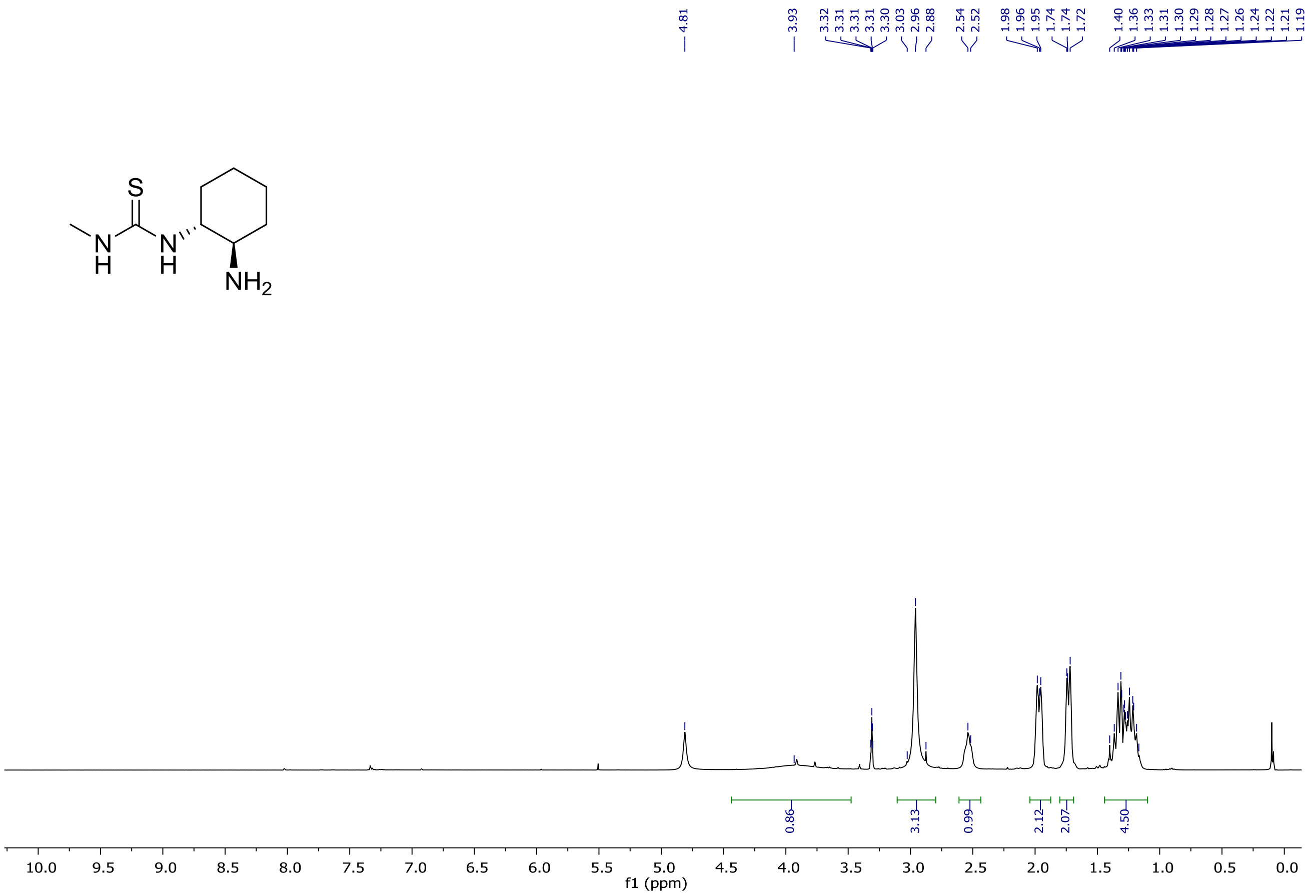

^{13}C NMR Spectrum of compound 4l

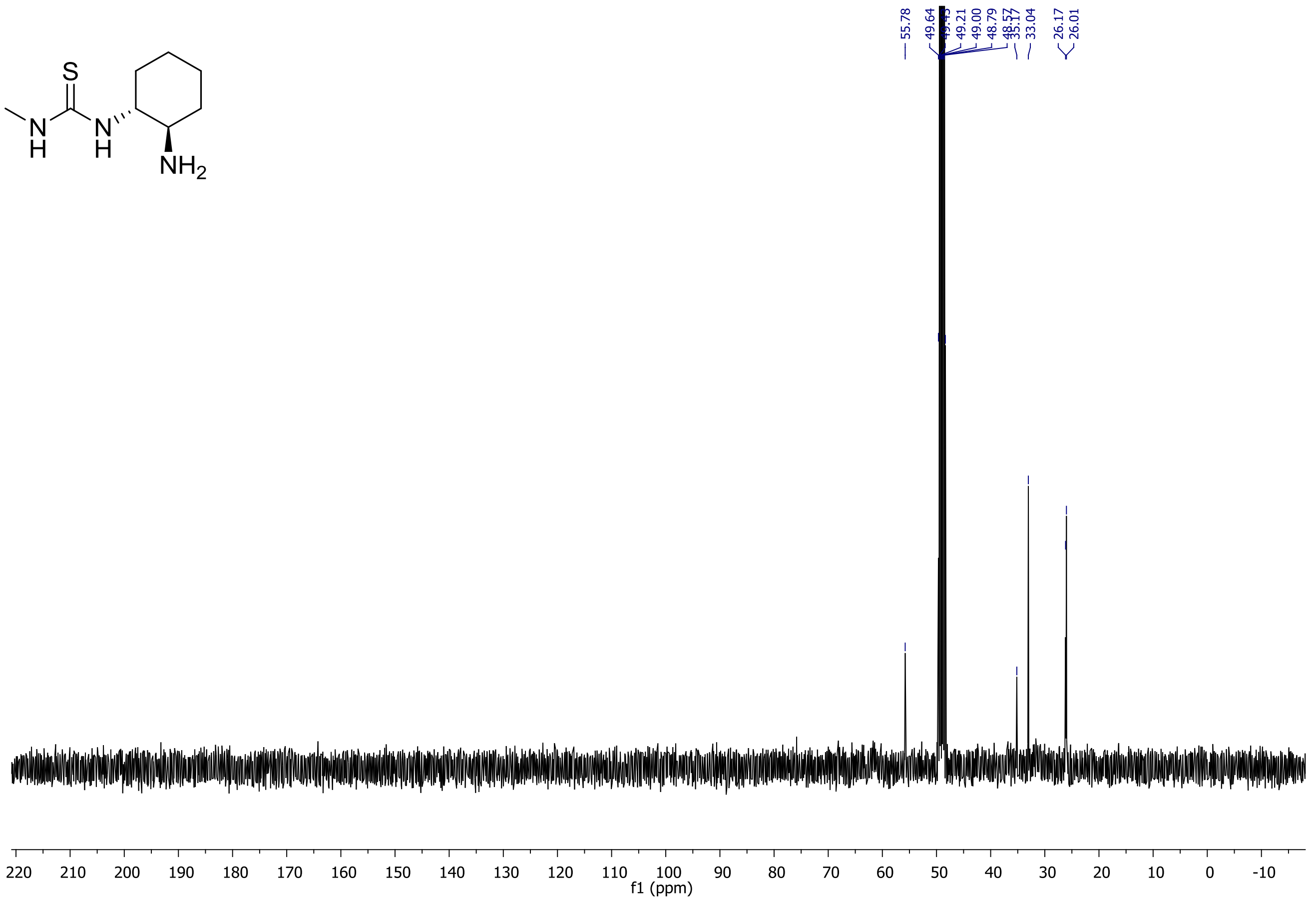

^{1}H NMR Spectrum of compound 37

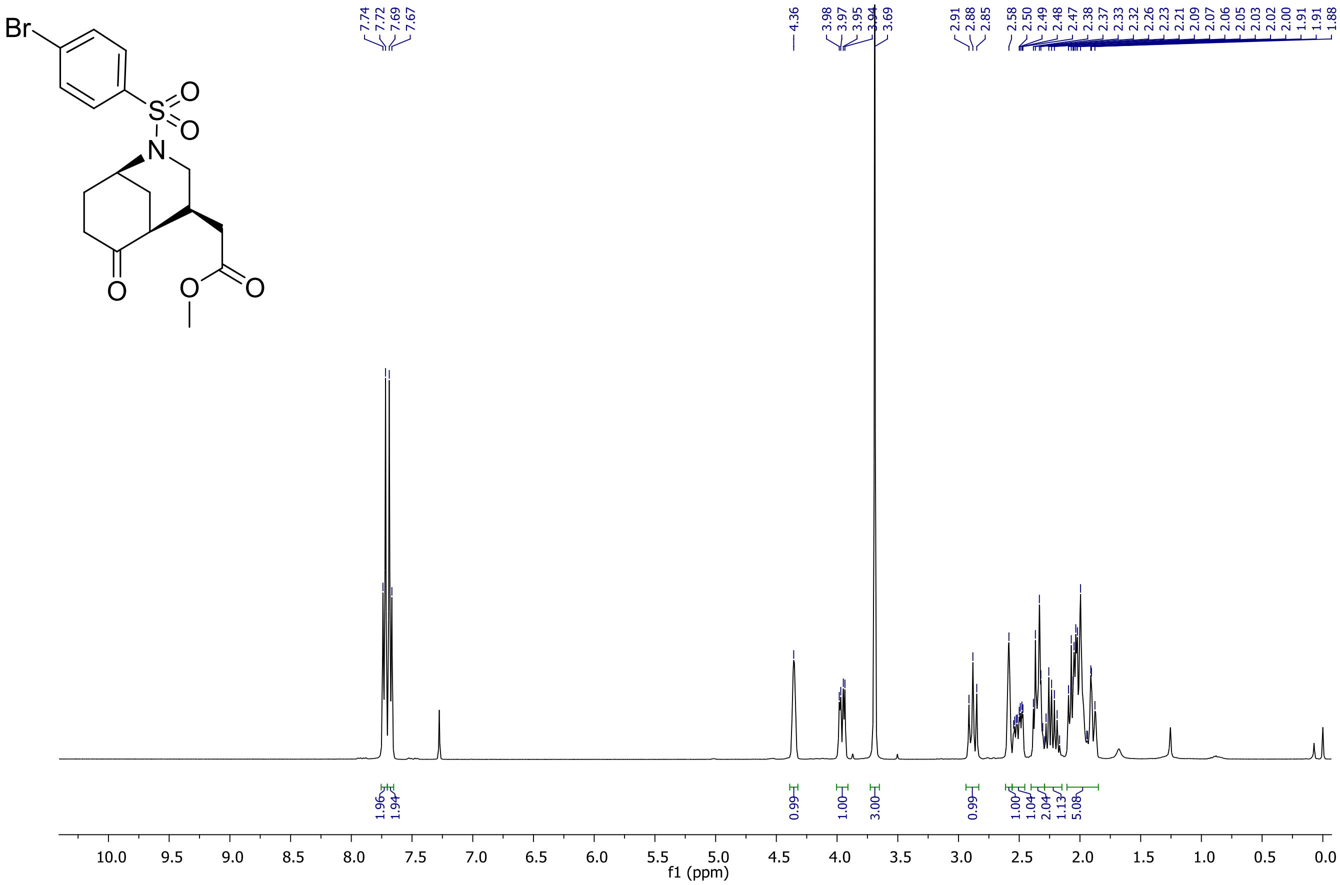

^{13}C NMR Spectrum of compound 37

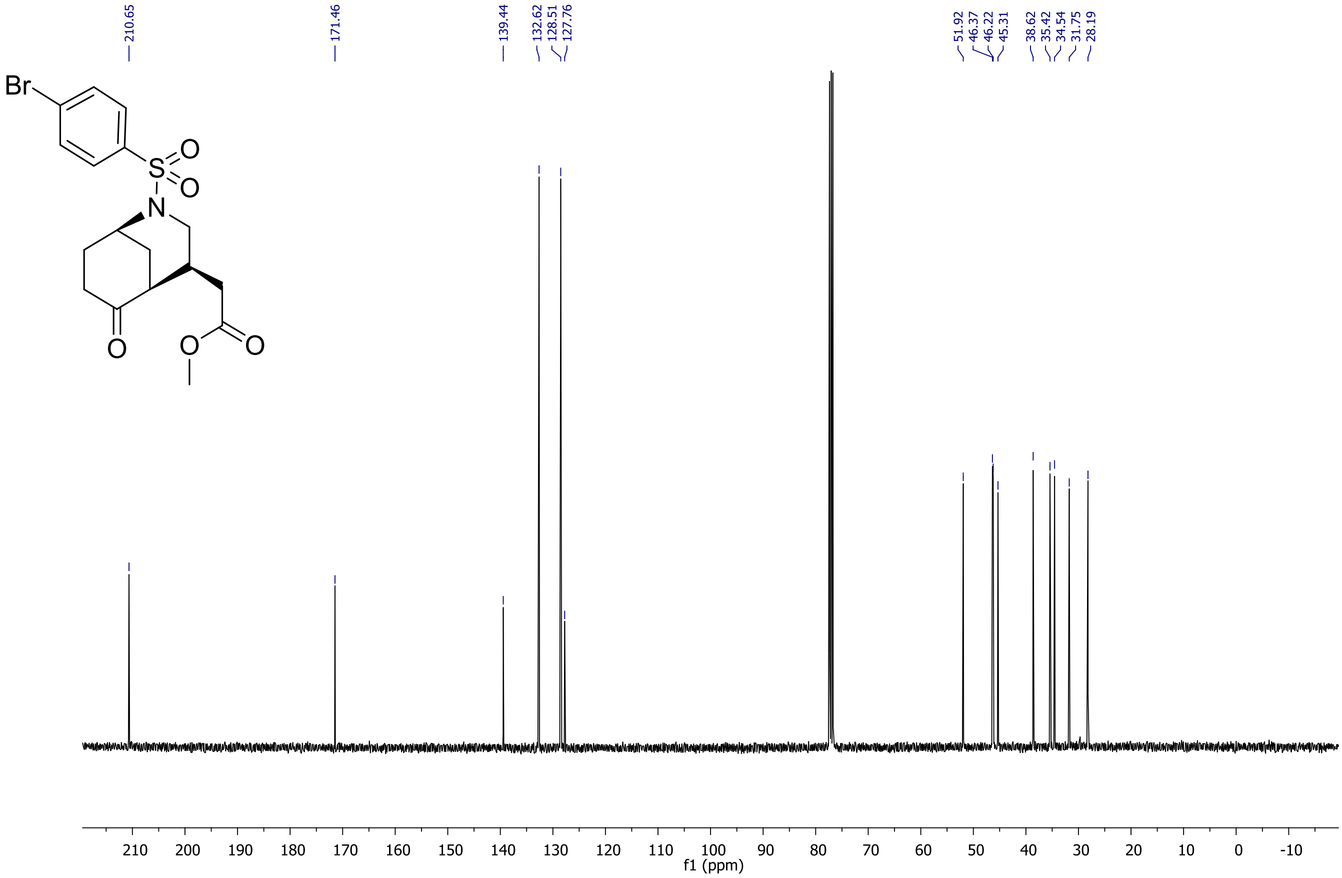

HPLC traces for racemic and enantioenriched 3a

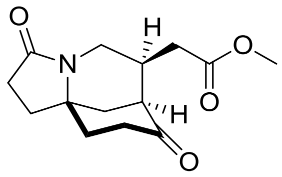

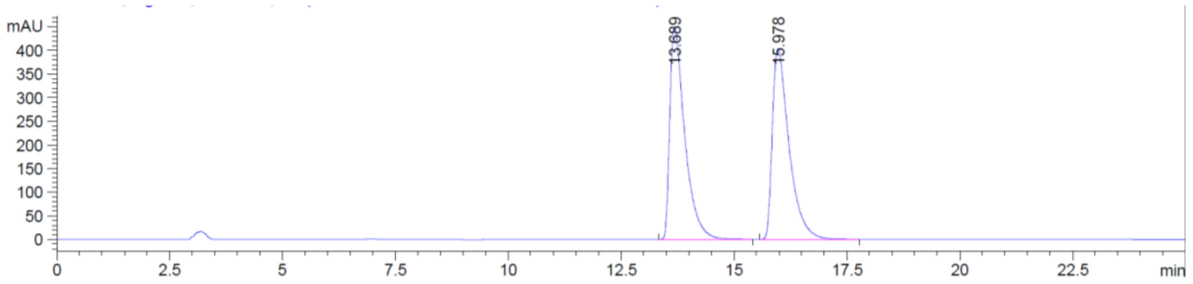

Signal 3: DAD1 C, Sig=210,8 Ref=360,100

Peak #	RetTime [min]	Type	Width [min]	Area [mAU*s]	Height [mAU]	Area %
1	13.689	BB	0.3461	1.04244e4	450.19766	50.0307
2	15.978	BB	0.3888	1.04116e4	403.78900	49.9693

Totals : 2.08360e4 853.98666

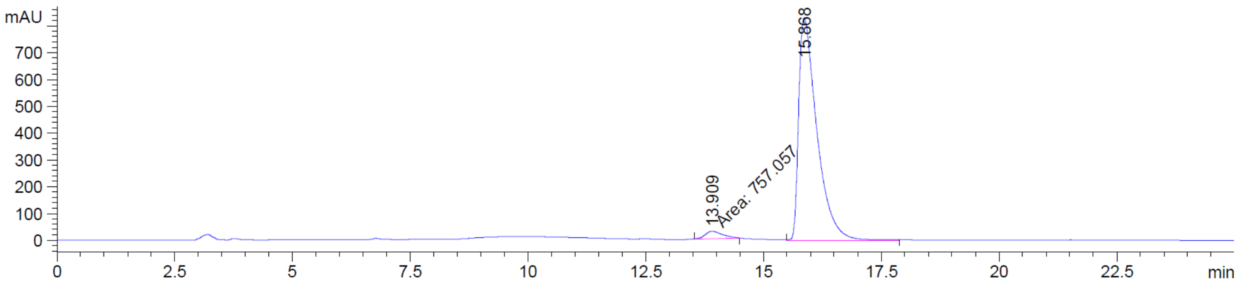

Signal 3: DAD1 C, Sig=210,8 Ref=360,100

Peak #	RetTime [min]	Type	Width [min]	Area [mAU*s]	Height [mAU]	Area %
1	13.909	MM	0.4435	757.05731	28.44970	3.3014
2	15.868	BB	0.4000	2.21743e4	829.09570	96.6986

Totals : 2.29314e4 857.54540

HPLC traces for racemic and enantioenriched 3b

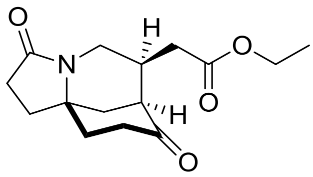

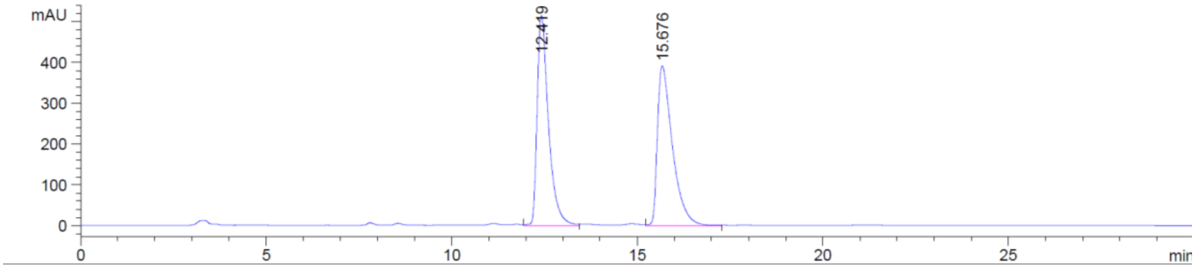

Signal 3: DAD1 C, Sig=210,8 Ref=360,100

Peak #	RetTime [min]	Type	Width [min]	Area [mAU*s]	Height [mAU]	Area %
1	12.419	VV	0.3243	1.09404e4	514.07703	49.9655
2	15.676	VB	0.4191	1.09555e4	390.69662	50.0345
Totals :				2.18960e4	904.77365	

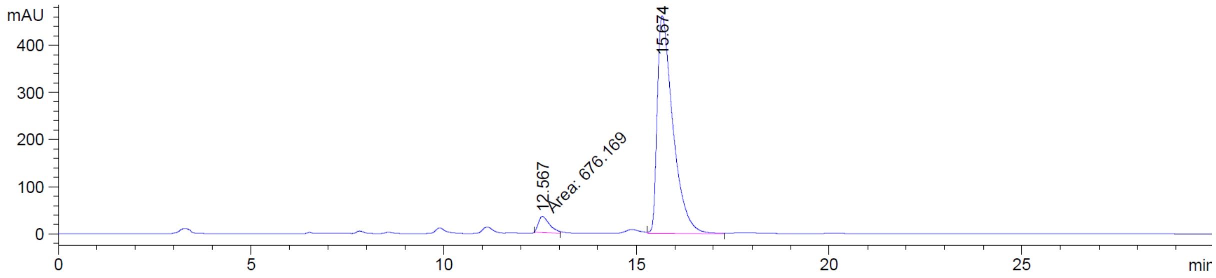

Signal 3: DAD1 C, Sig=210,8 Ref=360,100

Peak #	RetTime [min]	Type	Width [min]	Area [mAU*s]	Height [mAU]	Area %
1	12.567	MM	0.3355	676.16870	33.59481	4.9312
2	15.674	VB	0.4217	1.30359e4	461.25882	95.0688
Totals :				1.37121e4	494.85363	

HPLC traces for racemic and enantioenriched 3c

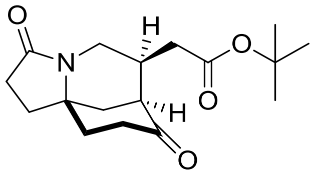

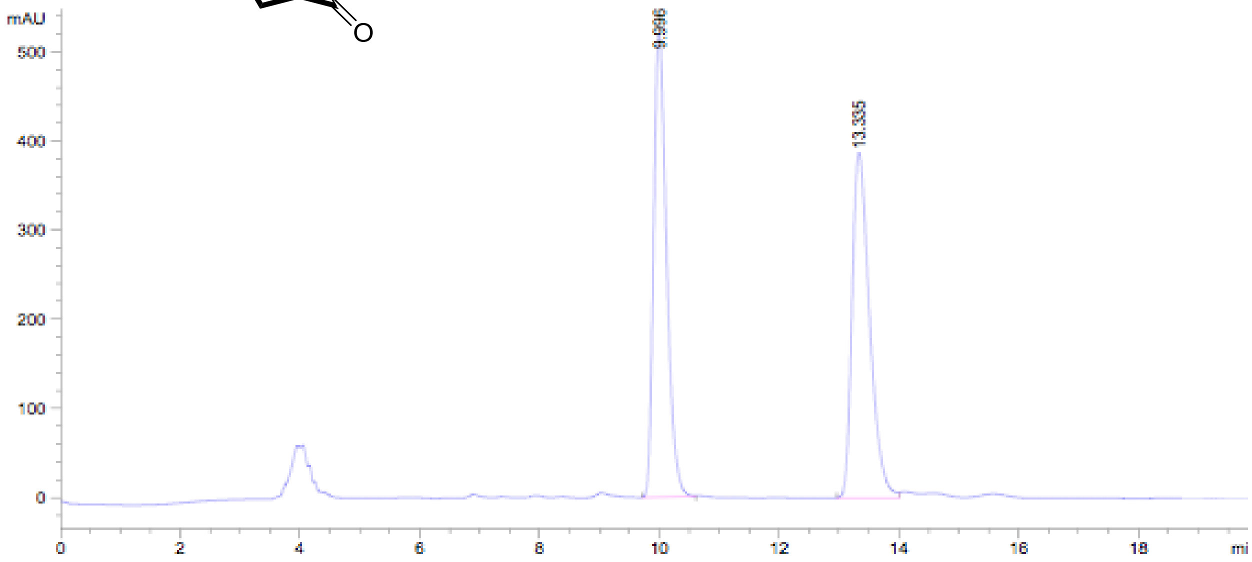

Peak #	RetTime [min]	Type	Width [min]	Area [mAU*s]	Height [mAU]	Area %
1	9.996	BB	0.2307	7803.37842	521.02563	49.7178
2	13.335	BB	0.3129	7891.95508	387.06482	50.2822

Totals : 1.56953e4 908.09045

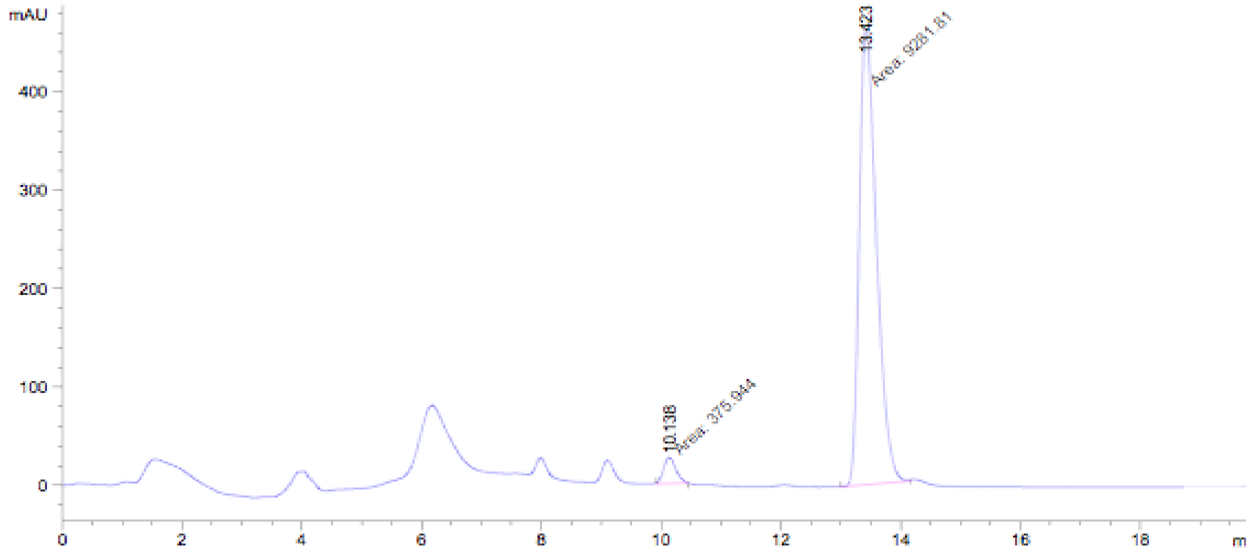

Peak #	RetTime [min]	Type	Width [min]	Area [mAU*s]	Height [mAU]	Area %
1	10.138	MM	0.2373	375.94394	26.39934	3.8927
2	13.423	MM	0.3338	9281.81445	463.48447	96.1073

HPLC traces for racemic and enantioenriched 3d

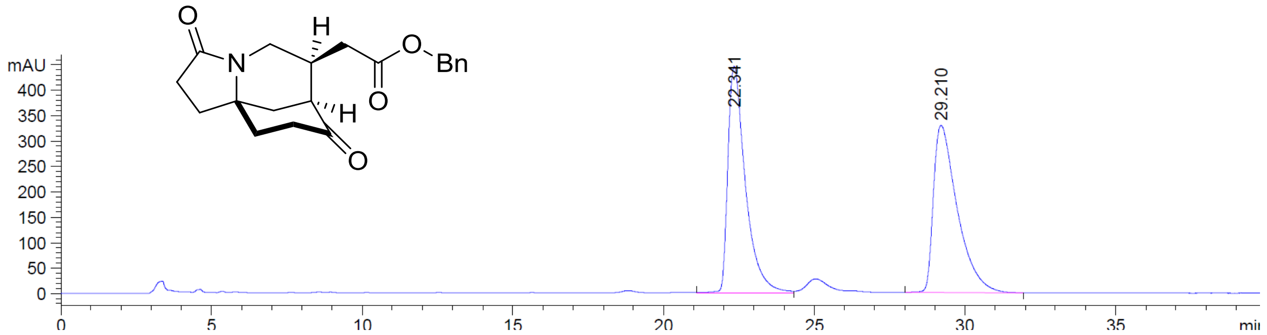

Signal 3: DAD1 C, Sig=210,8 Ref=360,100

Peak #	RetTime [min]	Type	Width [min]	Area [mAU*s]	Height [mAU]	Area %
1	22.341	VV	0.6045	1.80676e4	446.11331	49.8182
2	29.210	BB	0.8210	1.81995e4	329.16943	50.1818
Totals :				3.62671e4	775.28275	

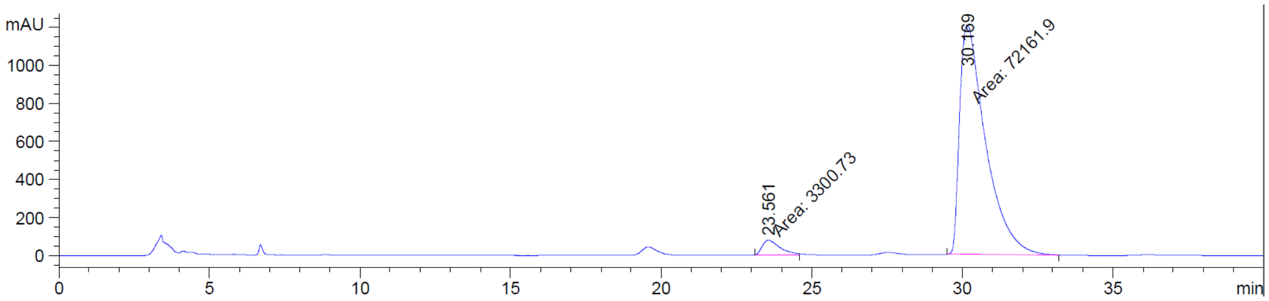

Signal 3: DAD1 C, Sig=210,8 Ref=360,100

Peak #	RetTime [min]	Type	Width [min]	Area [mAU*s]	Height [mAU]	Area %
1	23.561	MM	0.7003	3300.72510	78.55589	4.3740
2	30.169	MM	0.9980	7.21619e4	1205.10522	95.6260
Totals :				7.54626e4	1283.66111	

HPLC traces for racemic and enantioenriched 3e

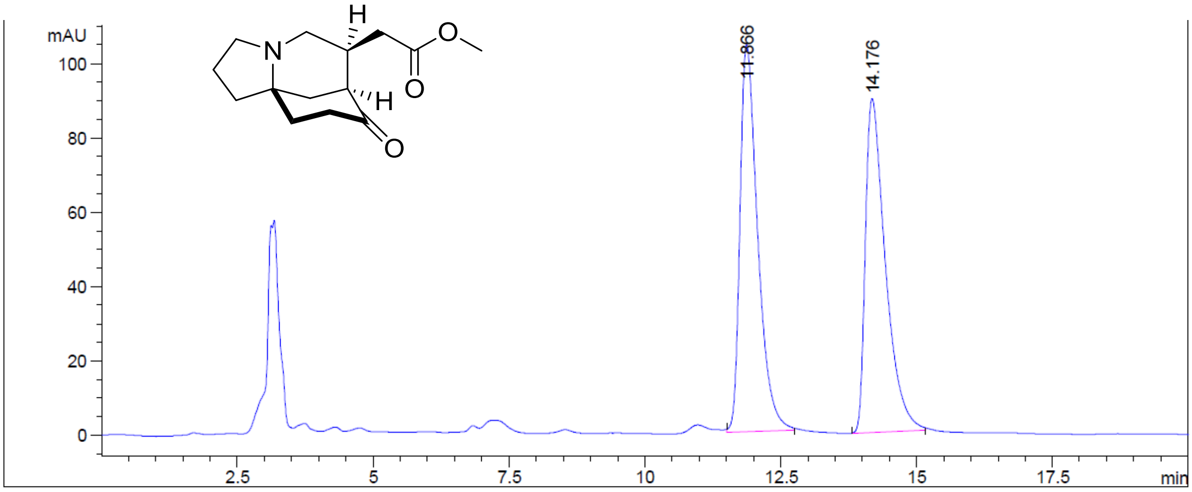

Signal 2: DAD1 B, Sig=210,8 Ref=360,100

Peak #	RetTime [min]	Type	Width [min]	Area [mAU*s]	Height [mAU]	Area %
1	11.866	BB	0.3410	2347.52637	104.11831	50.5560
2	14.176	BB	0.3823	2295.89136	89.80228	49.4440

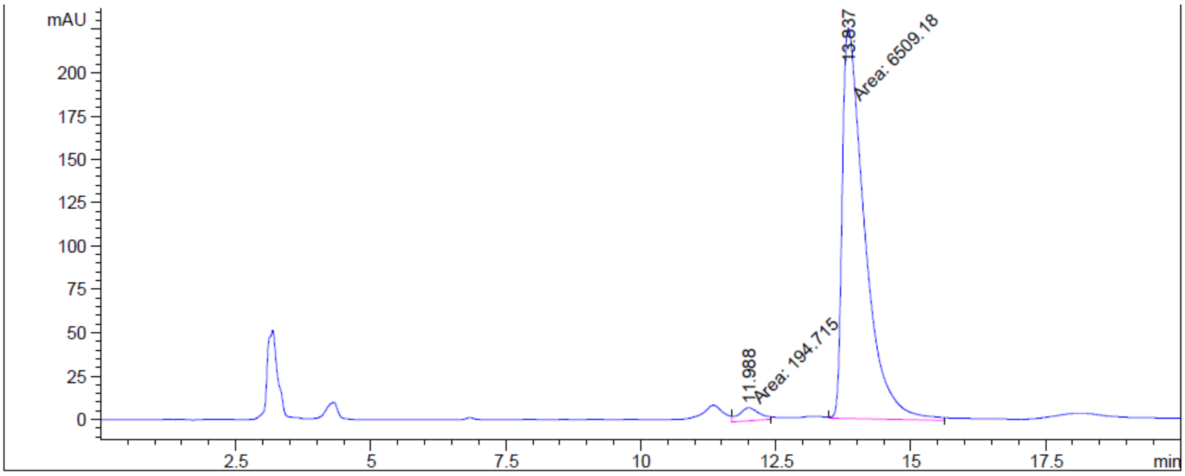

Signal 2: DAD1 B, Sig=210,8 Ref=360,100

Peak #	RetTime [min]	Type	Width [min]	Area [mAU*s]	Height [mAU]	Area %
1	11.988	MM	0.4324	194.71451	7.50544	2.9045
2	13.837	MM	0.4822	6509.18311	224.99445	97.0955

Totals : 6703.89761 232.49989

HPLC traces for racemic and enantioenriched 3f

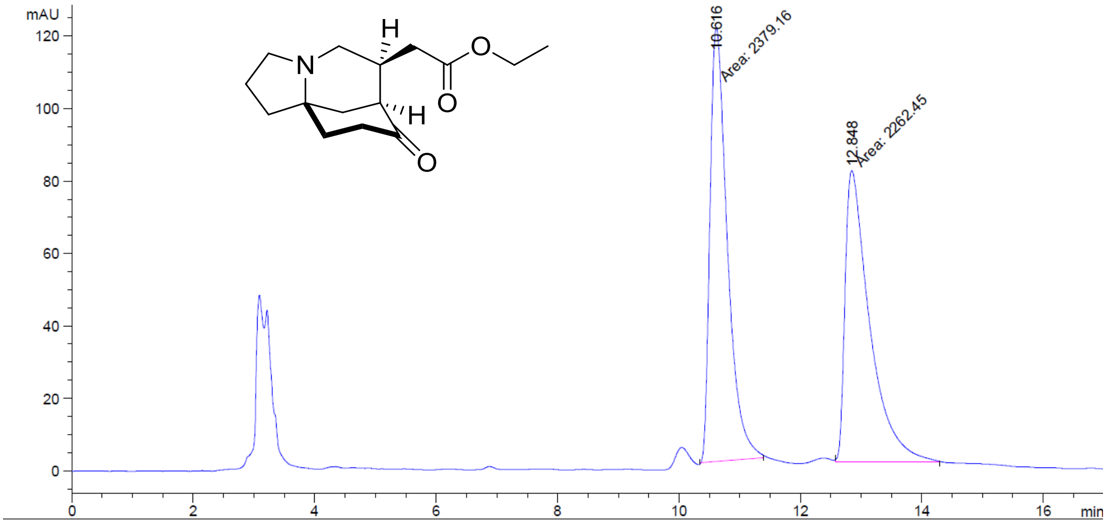

Signal 1: DAD1 C, Sig=210,8 Ref=360,100

Peak #	RetTime [min]	Type	Width [min]	Area [mAU*s]	Height [mAU]	Area %
1	10.616	MM	0.3305	2379.15747	119.96101	51.2572
2	12.848	MM	0.4683	2262.44775	80.51263	48.7428

Totals : 4641.60522 200.47363

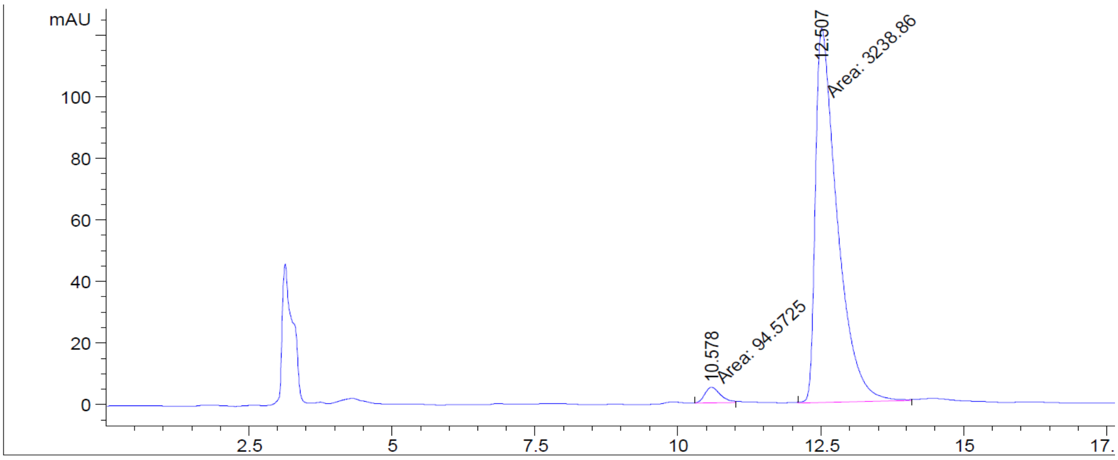

Signal 2: DAD1 B, Sig=210,8 Ref=360,100

Peak #	RetTime [min]	Type	Width [min]	Area [mAU*s]	Height [mAU]	Area %
1	10.578	MM	0.3131	94.57246	5.03468	2.8371
2	12.507	MM	0.4435	3238.86230	121.72528	97.1629

HPLC traces for racemic and enantioenriched 3g

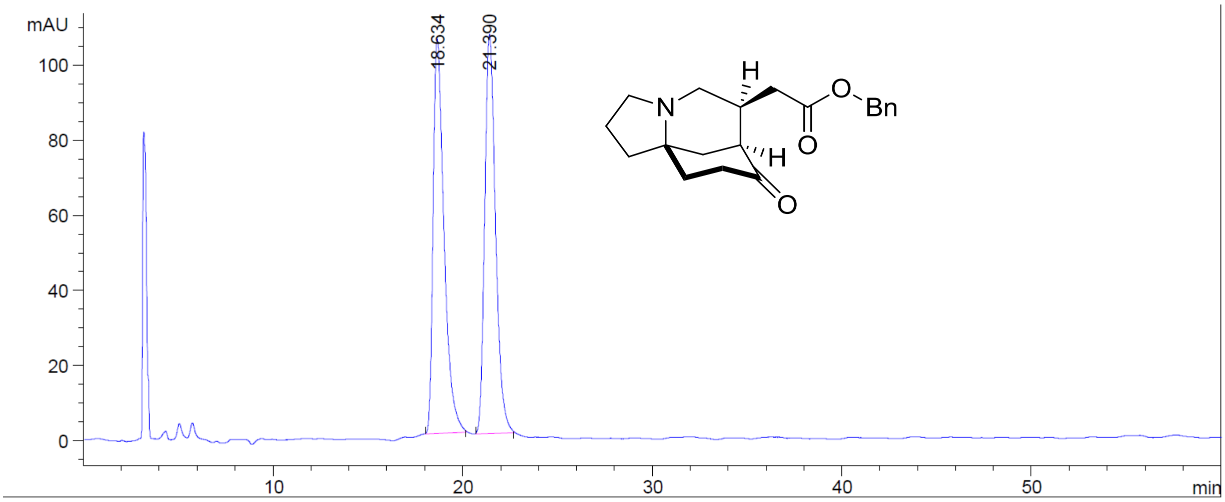


```
Signal 2: DAD1 B, Sig=210,8 Ref=360,100
```

Peak #	RetTime [min]	Type	Width [min]	Area [mAU*s]	Height [mAU]	Area %
1	18.634	BB	0.6055	4202.92920	104.88960	49.9082
2	21.390	BB	0.6088	4218.38916	106.33868	50.0918

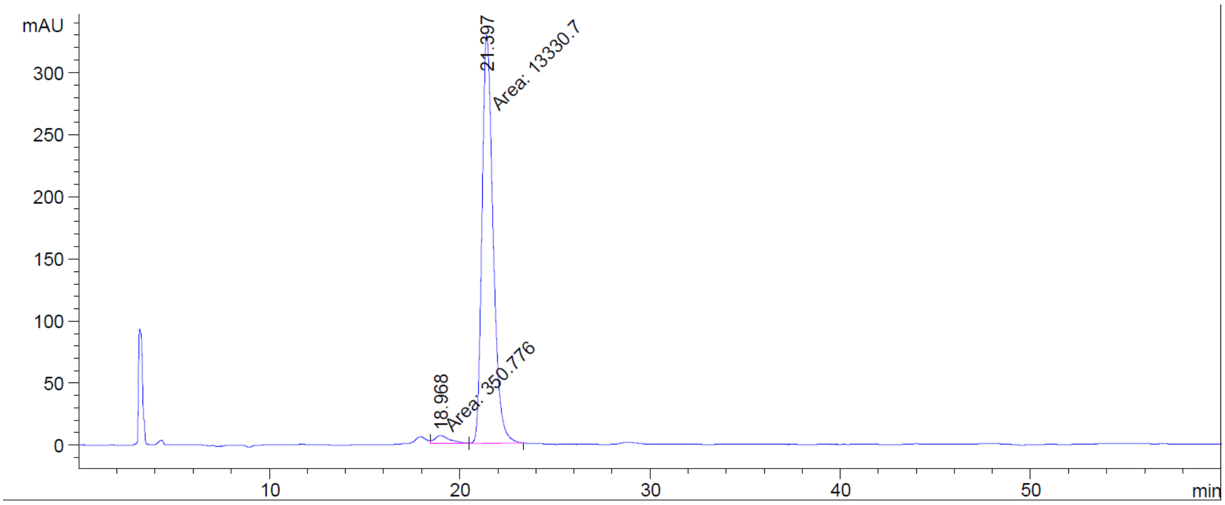


```
Signal 2: DAD1 B, Sig=210,8 Ref=360,100
```

Peak #	RetTime [min]	Type	Width [min]	Area [mAU*s]	Height [mAU]	Area %
1	18.968	FM	0.9203	350.77621	6.35251	2.5639
2	21.397	FM	0.6757	1.33307e4	328.79370	97.4361

HPLC traces for racemic and enantioenriched 3h

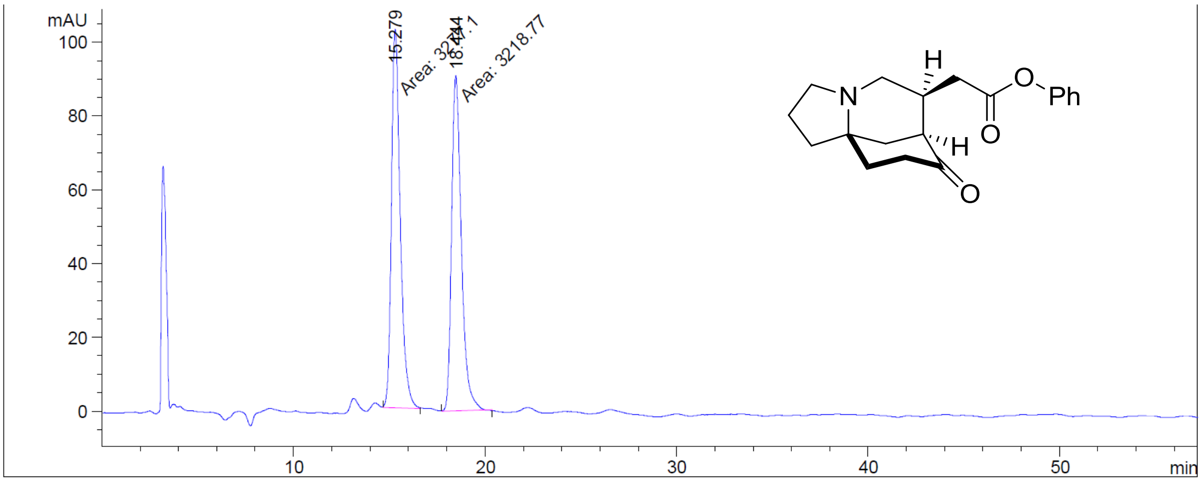

Signal 2: DAD1 B, Sig=210,8 Ref=360,100

Peak #	RetTime [min]	Type	Width [min]	Area [mAU*s]	Height [mAU]	Area %
1	15.279	MM	0.5321	3277.09521	102.64602	50.4490
2	18.444	MM	0.5900	3218.76855	90.92614	49.5510

Totals : 6495.86377 193.57216

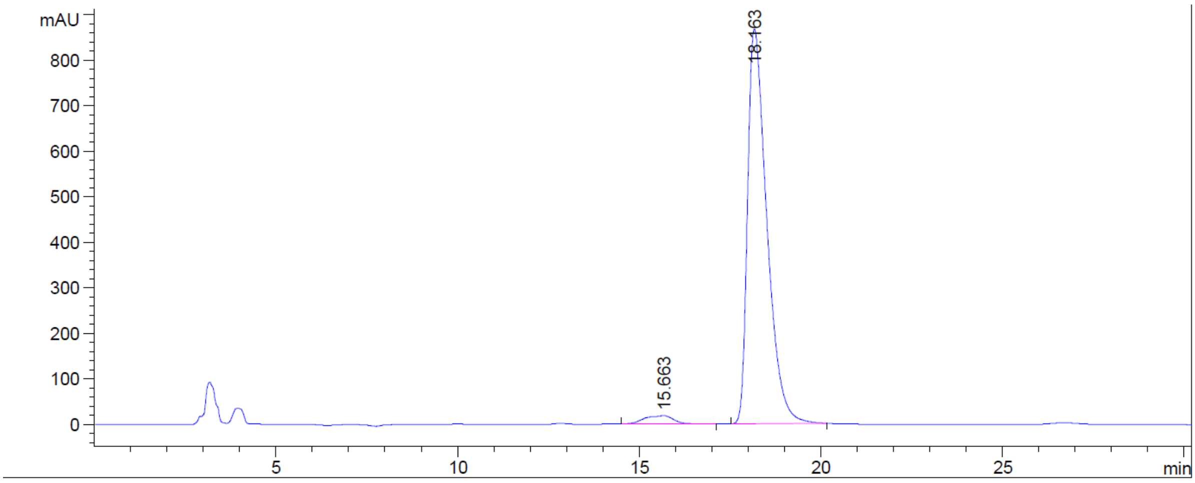

Signal 2: DAD1 B, Sig=210,8 Ref=360,100

Peak #	RetTime [min]	Type	Width [min]	Area [mAU*s]	Height [mAU]	Area %
1	15.663	BB	0.7534	1027.81299	18.35126	3.1182
2	18.163	BB	0.5597	3.19337e4	866.92487	96.8818

HPLC traces for racemic and enantioenriched 3i

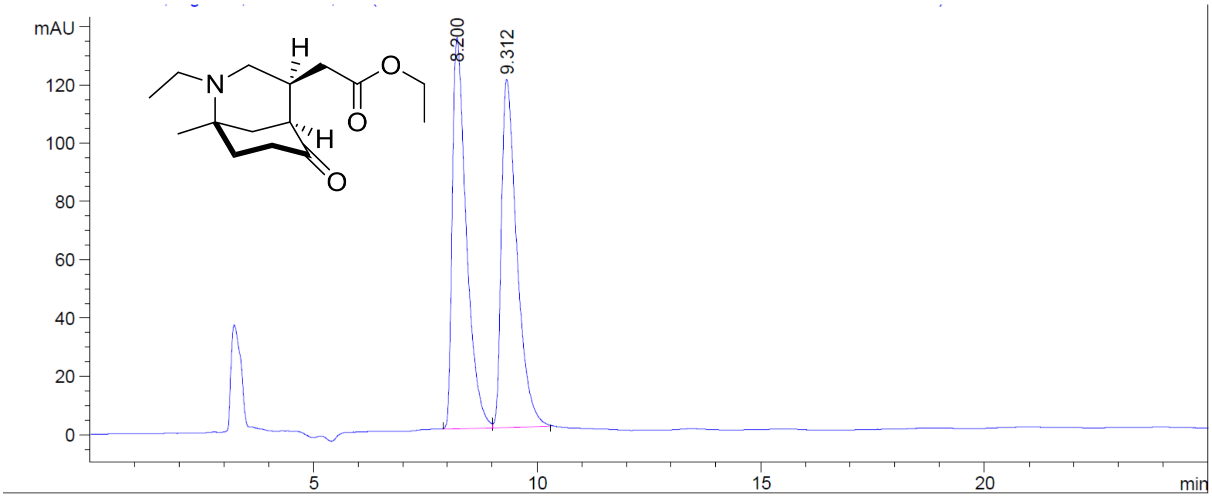

Signal 3: DAD1 C, Sig=210,8 Ref=360,100

Peak #	RetTime [min]	Type	Width [min]	Area [mAU*s]	Height [mAU]	Area %
1	8.200	BV	0.3265	2931.37134	134.38713	50.3559
2	9.312	VB	0.3645	2889.94043	119.34241	49.6441

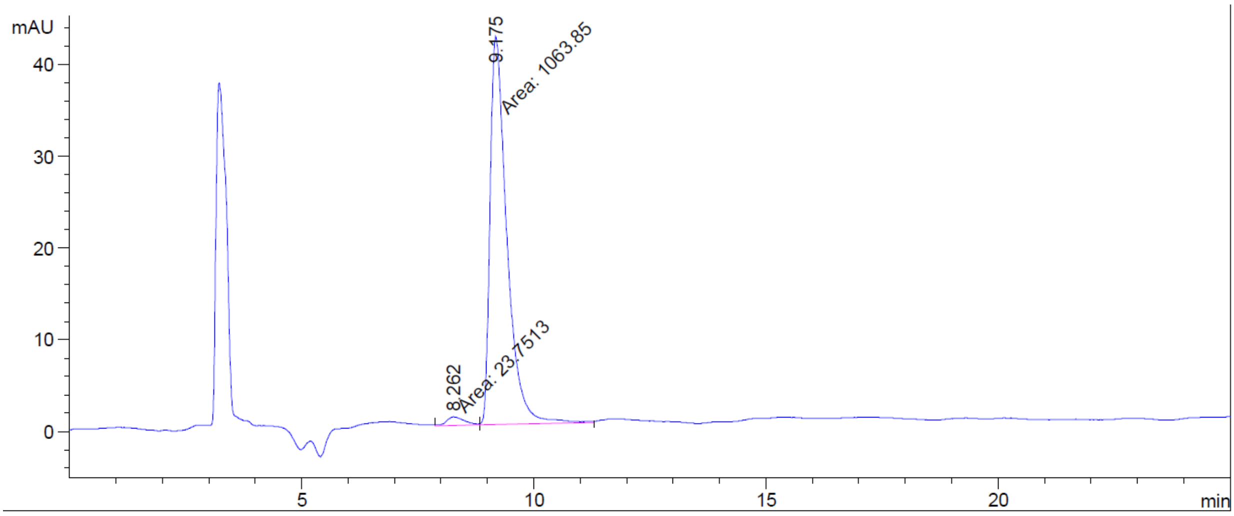

Signal 3: DAD1 C, Sig=210,8 Ref=360,100

Peak #	RetTime [min]	Type	Width [min]	Area [mAU*s]	Height [mAU]	Area %
1	8.262	MM	0.4309	23.75130	9.18626e-1	2.1838
2	9.175	MM	0.4197	1063.84937	42.24476	97.8162

HPLC traces for racemic and enantioenriched 3j

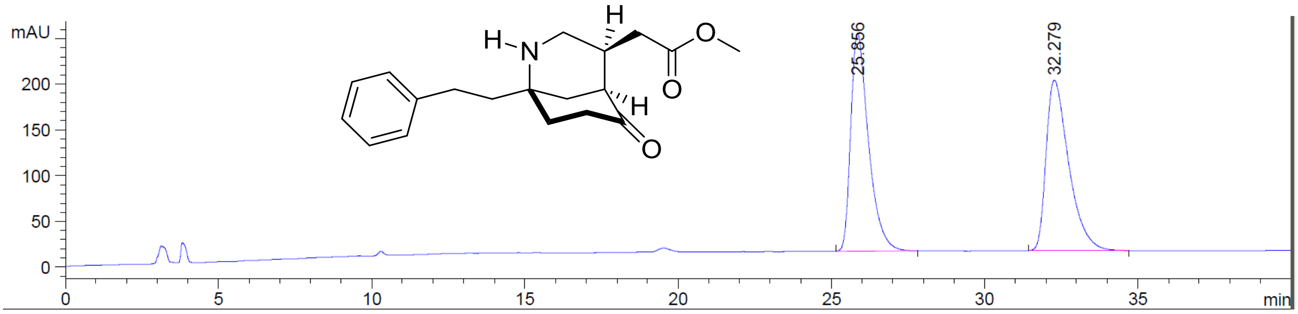

Signal 3: DAD1 C, Sig=210,8 Ref=360,100

Peak #	RetTime [min]	Type	Width [min]	Area [mAU*s]	Height [mAU]	Area %
1	25.856	BB	0.6109	9615.61426	238.22517	49.9694
2	32.279	BB	0.7820	9627.37402	186.57584	50.0306

Totals : 1.92430e4 424.80101

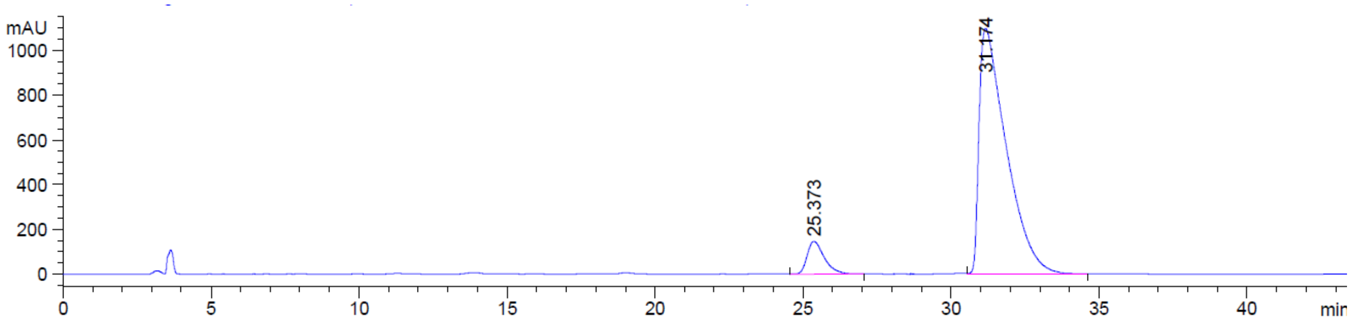

Signal 3: DAD1 C, Sig=210,8 Ref=360,100

Peak #	RetTime [min]	Type	Width [min]	Area [mAU*s]	Height [mAU]	Area %
1	25.373	VB	0.6151	5907.99512	146.29539	7.7788
2	31.174	VB	0.9216	7.00423e4	1095.92273	92.2212

Totals : 7.59503e4 1242.21812

HPLC traces for racemic and enantioenriched 3k

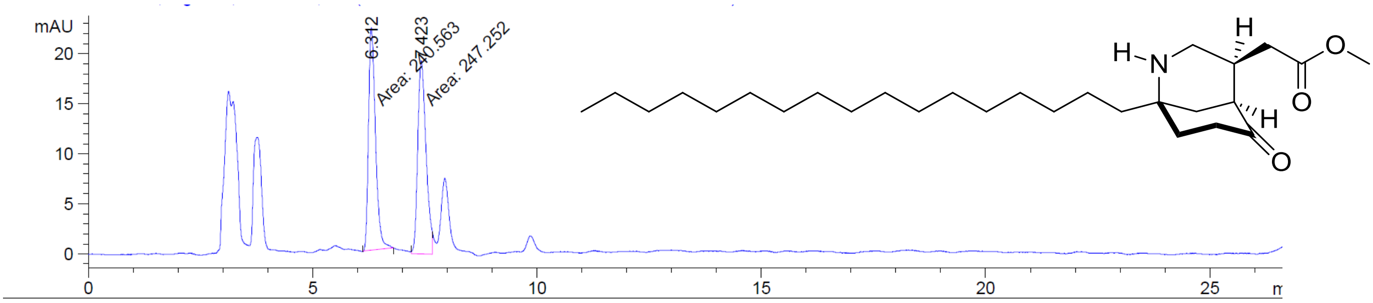

Signal 3: DAD1 C, Sig=210,8 Ref=360,100

Peak #	RetTime [min]	Type	Width [min]	Area [mAU*s]	Height [mAU]	Area %
1	6.312	MM	0.1797	240.56291	22.30534	49.3144
2	7.423	MF	0.2134	247.25163	19.31485	50.6856

Totals : 487.81454 41.62019

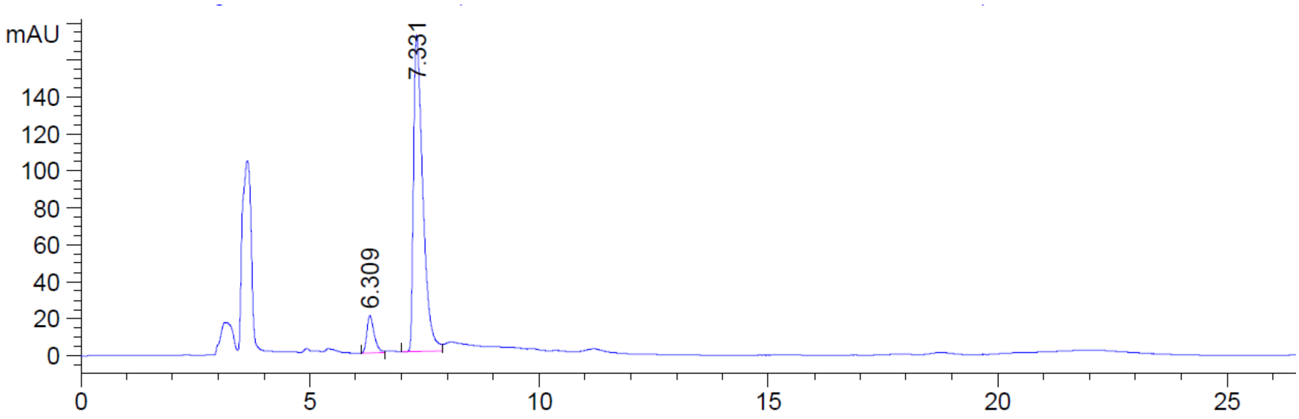

Signal 3: DAD1 C, Sig=210,8 Ref=360,100

Peak #	RetTime [min]	Type	Width [min]	Area [mAU*s]	Height [mAU]	Area %
1	6.309	BV	0.1701	224.55870	20.13482	8.4816
2	7.331	VV	0.2169	2423.03467	171.48003	91.5184

Totals : 2647.59337 191.61485

HPLC traces for racemic and enantioenriched 3l

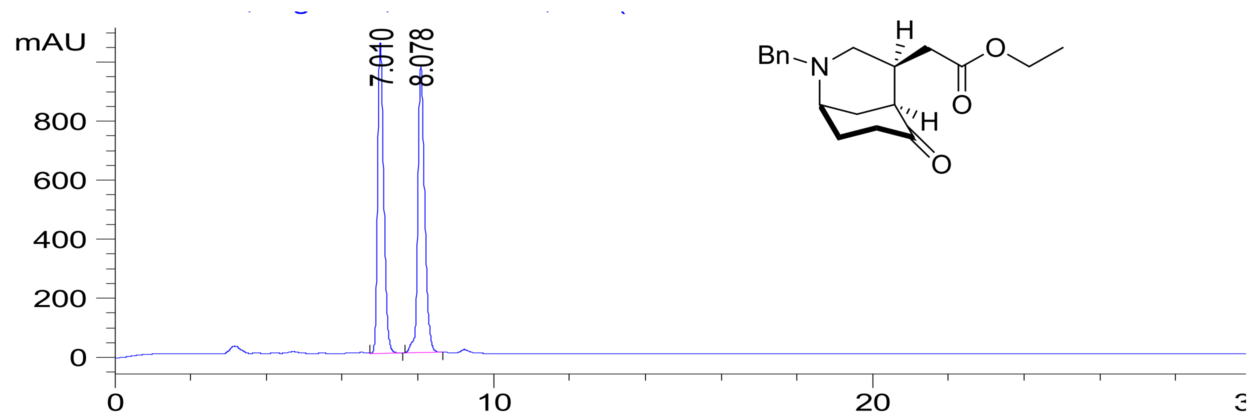


```
Signal 3: DAD1 C, Sig=210,8 Ref=360,100

Peak RetTime Type  Width     Area       Height      Area
  #   [min]        [min]   [mAU*s]      [mAU]        %
----|-------|----|-------|----------|----------|--------|
   1   7.010 VB    0.1721 1.17155e4  1050.54333  48.7907
   2   8.078 BB    0.1922 1.22962e4   974.74359  51.2093

Totals :                  2.40116e4  2025.28693
```

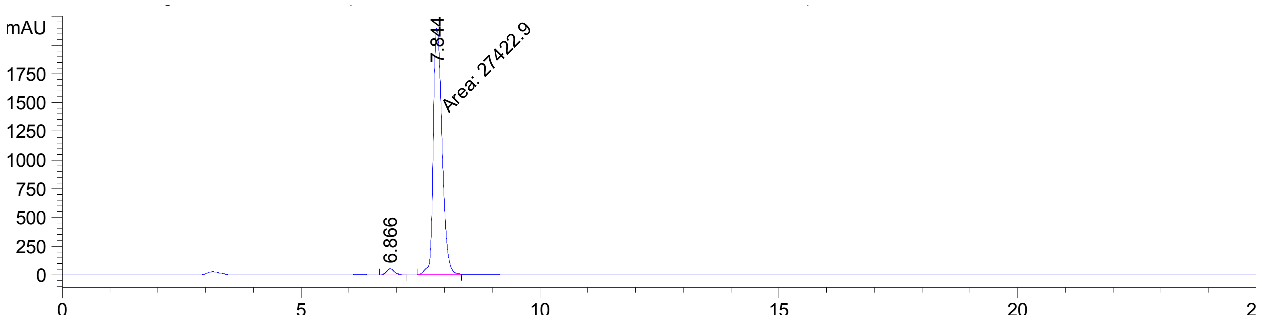


```
Signal 3: DAD1 C, Sig=210,8 Ref=360,100

Peak RetTime Type  Width     Area       Height      Area
  #   [min]        [min]   [mAU*s]      [mAU]        %
----|-------|----|-------|----------|----------|--------|
   1   6.866 BB    0.1667  577.52264   53.17301   2.0626
   2   7.844 MM    0.2134 2.74229e4  2141.57251  97.9374

Totals :                  2.80004e4  2194.74552
```

HPLC traces for racemic and enantioenriched 3m

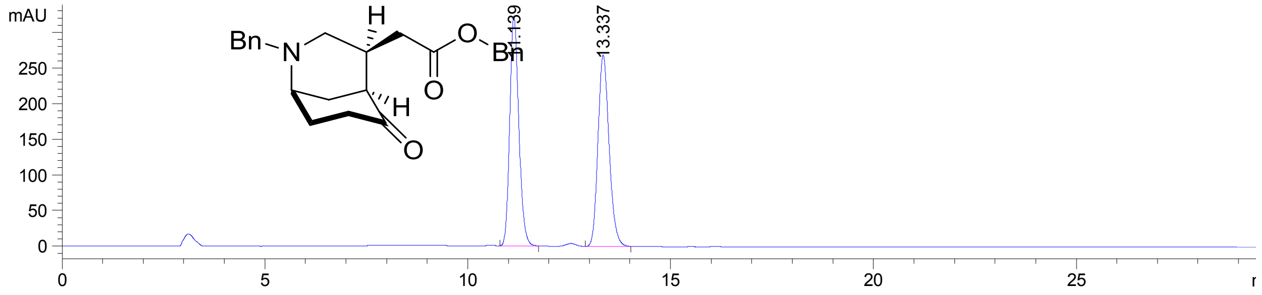

Signal 3: DAD1 C, Sig=210,8 Ref=360,100

Peak #	RetTime [min]	Type	Width [min]	Area [mAU*s]	Height [mAU]	Area %
1	11.139	BB	0.2402	5035.55908	322.46524	49.6041
2	13.337	VB	0.2931	5115.94678	268.74637	50.3959

Totals : 1.01515e4 591.21161

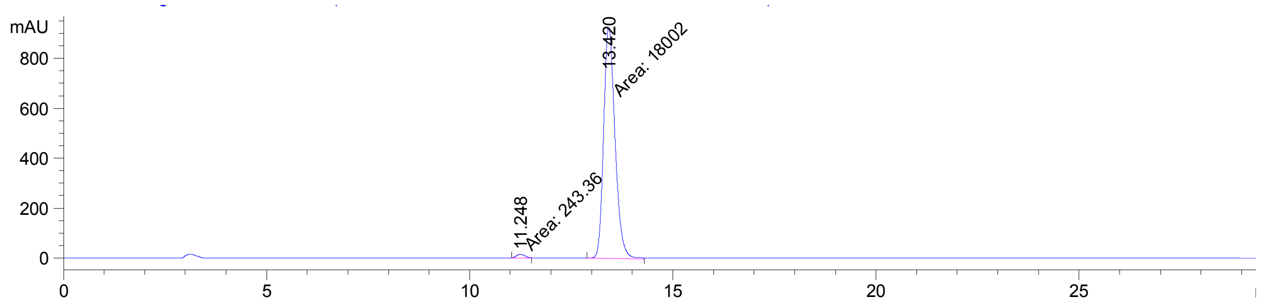

Signal 3: DAD1 C, Sig=210,8 Ref=360,100

Peak #	RetTime [min]	Type	Width [min]	Area [mAU*s]	Height [mAU]	Area %
1	11.248	MM	0.2588	243.35956	15.67101	1.3338
2	13.420	MM	0.3241	1.80020e4	925.73700	98.6662

Totals : 1.82454e4 941.40801

HPLC traces for racemic and enantioenriched 3n

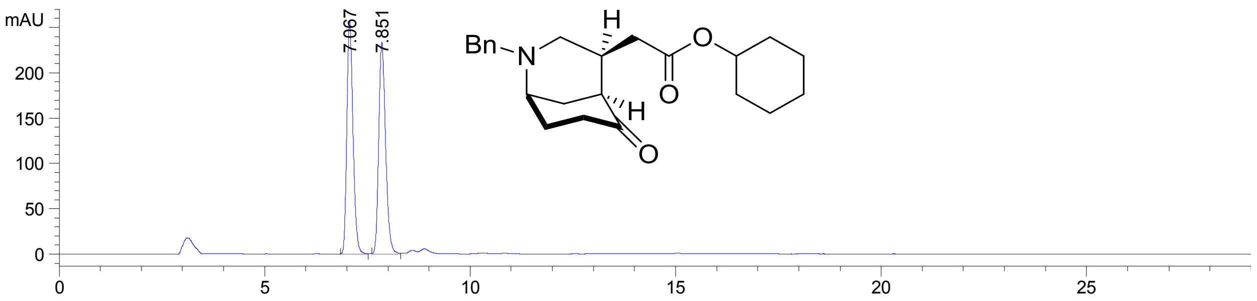

Signal 3: DAD1 C, Sig=210,8 Ref=360,100

Peak #	RetTime [min]	Type	Width [min]	Area [mAU*s]	Height [mAU]	Area %
1	7.067	BB	0.1578	2647.07373	257.80960	49.6496
2	7.851	BB	0.1762	2684.43555	233.31787	50.3504

Totals : 5331.50928 491.12747

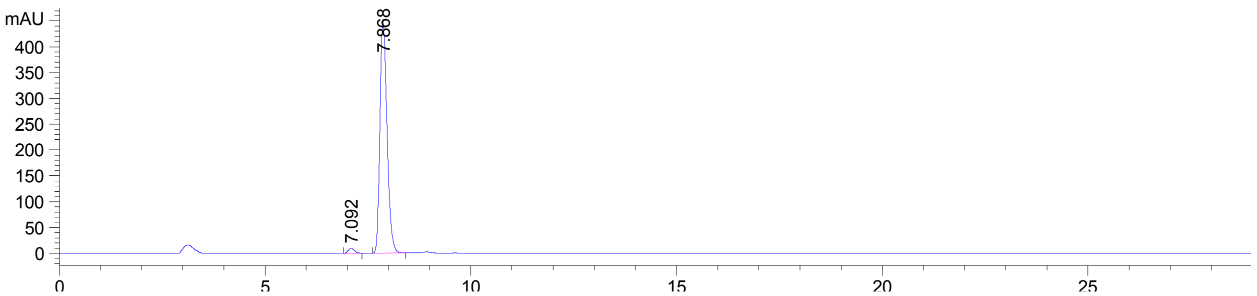

Signal 3: DAD1 C, Sig=210,8 Ref=360,100

Peak #	RetTime [min]	Type	Width [min]	Area [mAU*s]	Height [mAU]	Area %
1	7.092	BB	0.1558	99.66605	9.86672	1.8173
2	7.868	BB	0.1819	5384.75293	452.38721	98.1827

Totals : 5484.41898 462.25393

HPLC traces for racemic and enantioenriched 3o

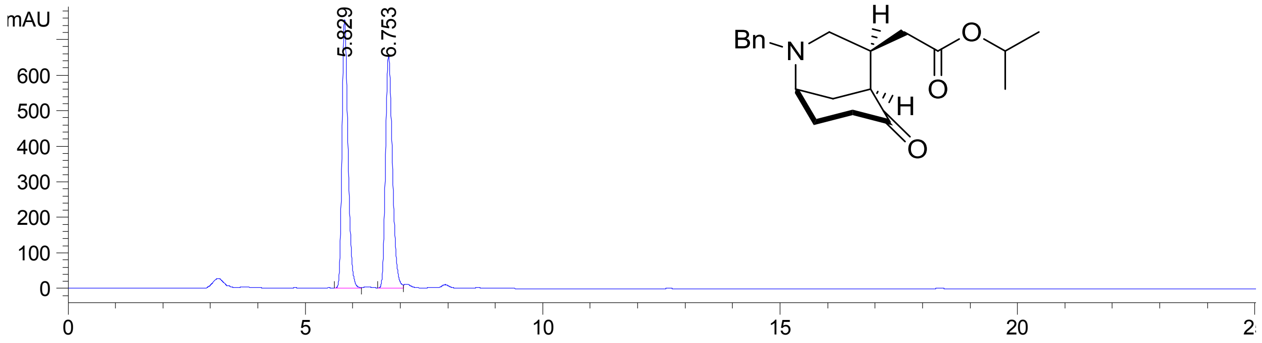

Signal 3: DAD1 C, Sig=210,8 Ref=360,100

Peak #	RetTime [min]	Type	Width [min]	Area [mAU*s]	Height [mAU]	Area %
1	5.829	VV	0.1357	6645.74707	755.06787	49.9127
2	6.753	VV	0.1579	6668.99072	654.53467	50.0873

Totals : 1.33147e4 1409.60254

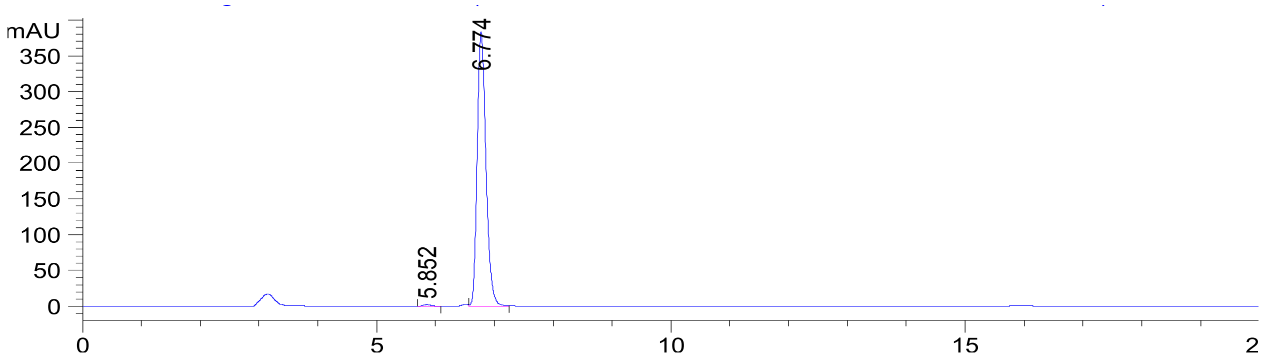

Signal 3: DAD1 C, Sig=210,8 Ref=360,100

Peak #	RetTime [min]	Type	Width [min]	Area [mAU*s]	Height [mAU]	Area %
1	5.852	BB	0.1383	20.31569	2.27326	0.5113
2	6.774	VB	0.1592	3953.04614	383.73468	99.4887

Totals : 3973.36183 386.00794

HPLC traces for racemic and enantioenriched 3p

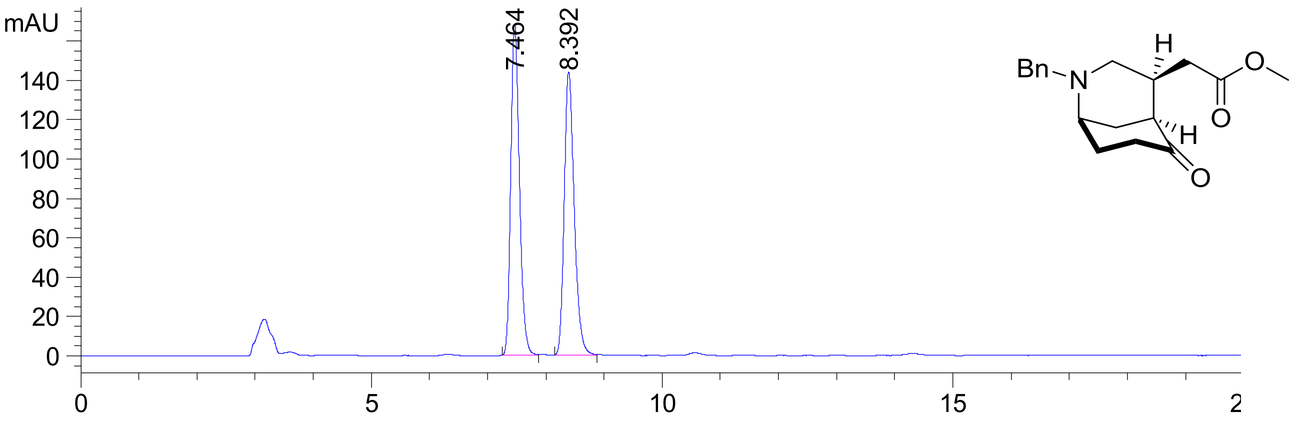

Signal 3: DAD1 C, Sig=210,8 Ref=360,100

Peak #	RetTime [min]	Type	Width [min]	Area [mAU*s]	Height [mAU]	Area %
1	7.464	BB	0.1578	1730.14514	168.51022	50.1176
2	8.392	BB	0.1837	1722.02551	143.79132	49.8824

Totals : 3452.17065 312.30154

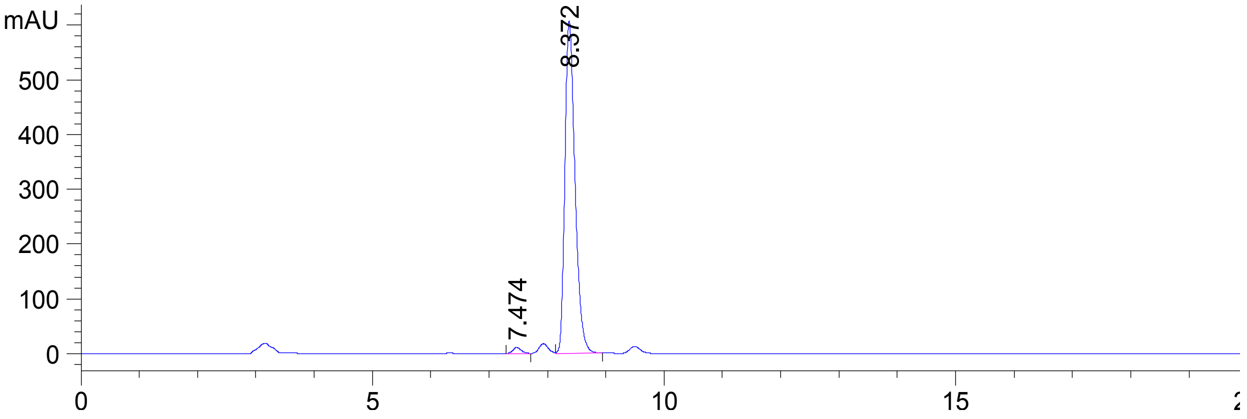

Signal 3: DAD1 C, Sig=210,8 Ref=360,100

Peak #	RetTime [min]	Type	Width [min]	Area [mAU*s]	Height [mAU]	Area %
1	7.474	BV	0.1530	109.60136	10.93092	1.4750
2	8.372	VB	0.1857	7320.99951	607.06958	98.5250

Totals : 7430.60087 618.00050

HPLC traces for racemic and enantioenriched 3q

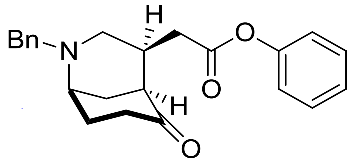

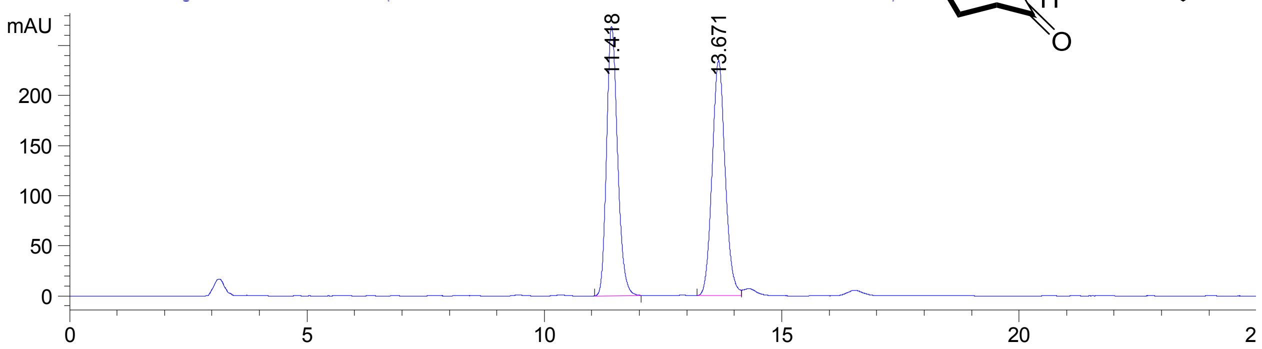

Signal 3: DAD1 C, Sig=210,8 Ref=360,100

Peak #	RetTime [min]	Type	Width [min]	Area [mAU*s]	Height [mAU]	Area %
1	11.418	BB	0.2462	4313.85010	268.91016	48.9700
2	13.671	BV	0.2957	4495.31055	234.42865	51.0300

Totals : 8809.16064 503.33881

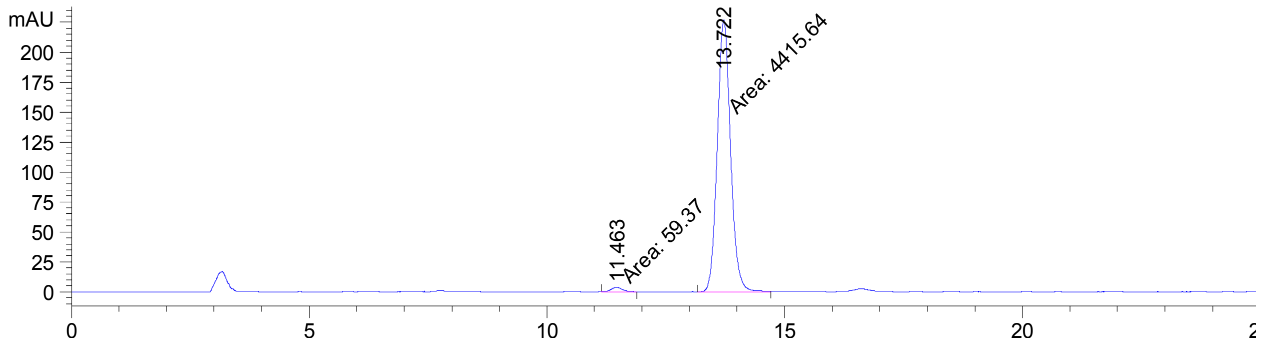

Signal 3: DAD1 C, Sig=210,8 Ref=360,100

Peak #	RetTime [min]	Type	Width [min]	Area [mAU*s]	Height [mAU]	Area %
1	11.463	MM	0.2756	59.36995	3.58982	1.3267
2	13.722	MM	0.3241	4415.64160	227.10223	98.6733

Totals : 4475.01155 230.69206

HPLC traces for racemic and enantioenriched 3r

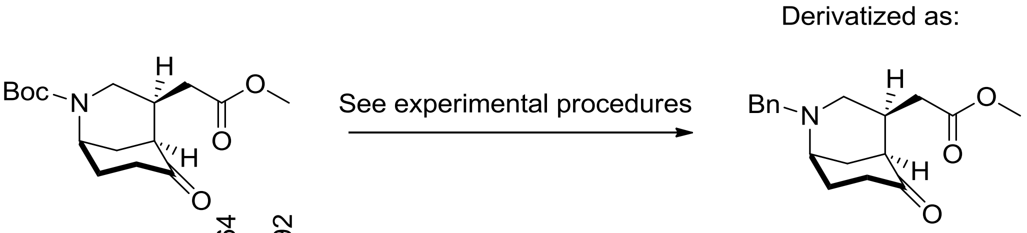

3q

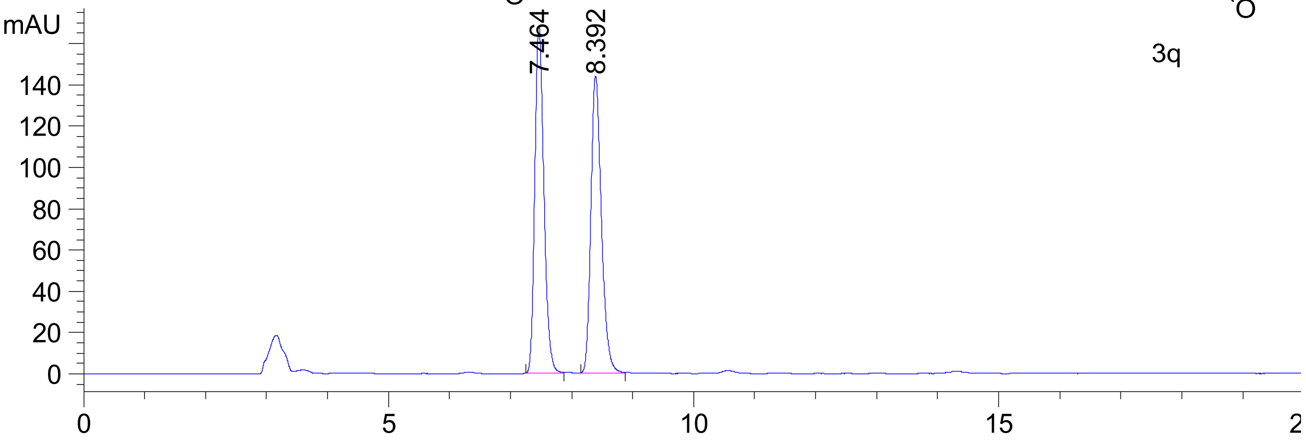


```
Signal 3: DAD1 C, Sig=210,8 Ref=360,100

Peak RetTime Type  Width     Area       Height      Area
  #   [min]        [min]   [mAU*s]      [mAU]        %
----|-------|----|-------|----------|----------|--------|
   1   7.464 BB    0.1578 1730.14514  168.51022  50.1176
   2   8.392 BB    0.1837 1722.02551  143.79132  49.8824

Totals :                  3452.17065  312.30154
```

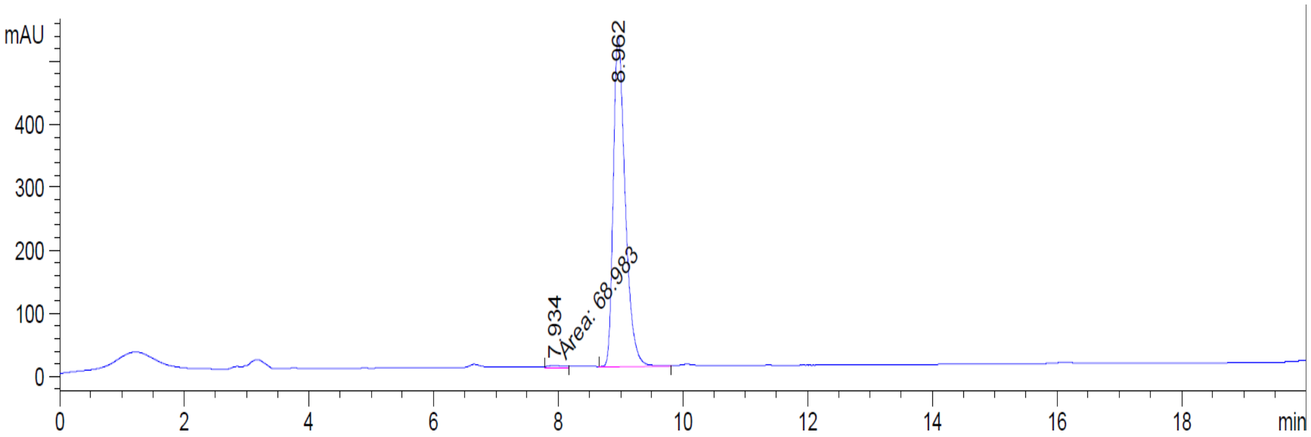


```
Signal 3: DAD1 C, Sig=210,8 Ref=360,100

Peak RetTime Type  Width     Area       Height      Area
  #   [min]        [min]   [mAU*s]      [mAU]        %
----|-------|----|-------|----------|----------|--------|
   1   7.934 FM    0.2680   68.98302    4.28946   0.9675
   2   8.962 VV    0.2048 7061.15283  525.45081  99.0325

Totals :                  7130.13586  529.74027
```

HPLC traces for racemic and enantioenriched 3s

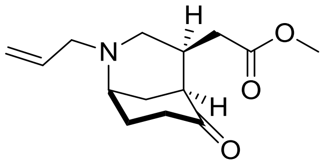

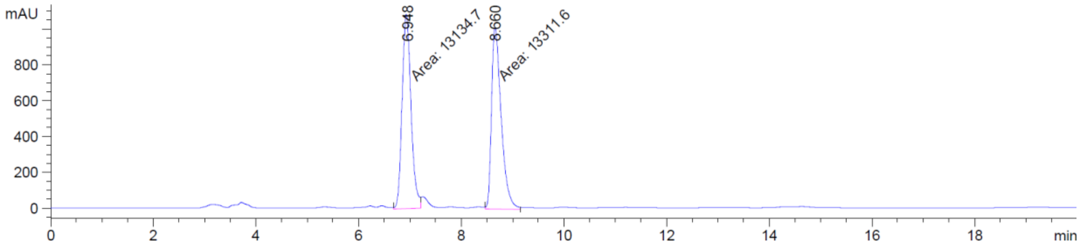

Signal 3: DAD1 C, Sig=210,8 Ref=360,100

Peak #	RetTime [min]	Type	Width [min]	Area [mAU*s]	Height [mAU]	Area %
1	6.948	MF	0.2010	1.31347e4	1089.35876	49.6656
2	8.660	MM	0.2180	1.33116e4	1017.62366	50.3344

Totals : 2.64463e4 2106.98242

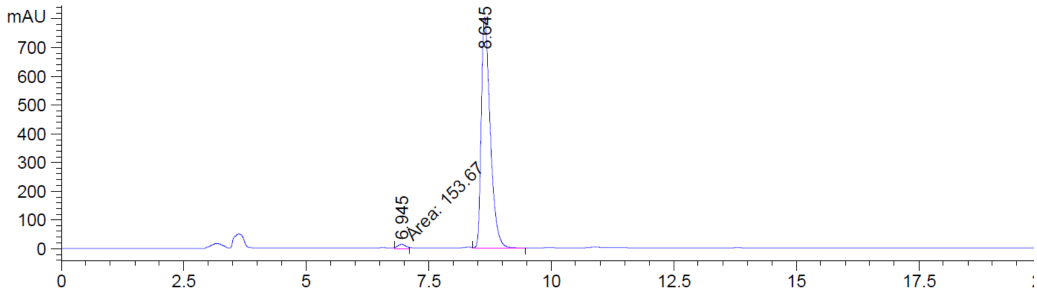

Signal 1: DAD1 C, Sig=210,8 Ref=360,100

Peak #	RetTime [min]	Type	Width [min]	Area [mAU*s]	Height [mAU]	Area %
1	6.945	MM	0.1865	153.66982	13.73542	1.4771
2	8.645	VB	0.1970	1.02495e4	802.72528	98.5229

Totals : 1.04032e4 816.46070

HPLC traces for racemic and enantioenriched 3t

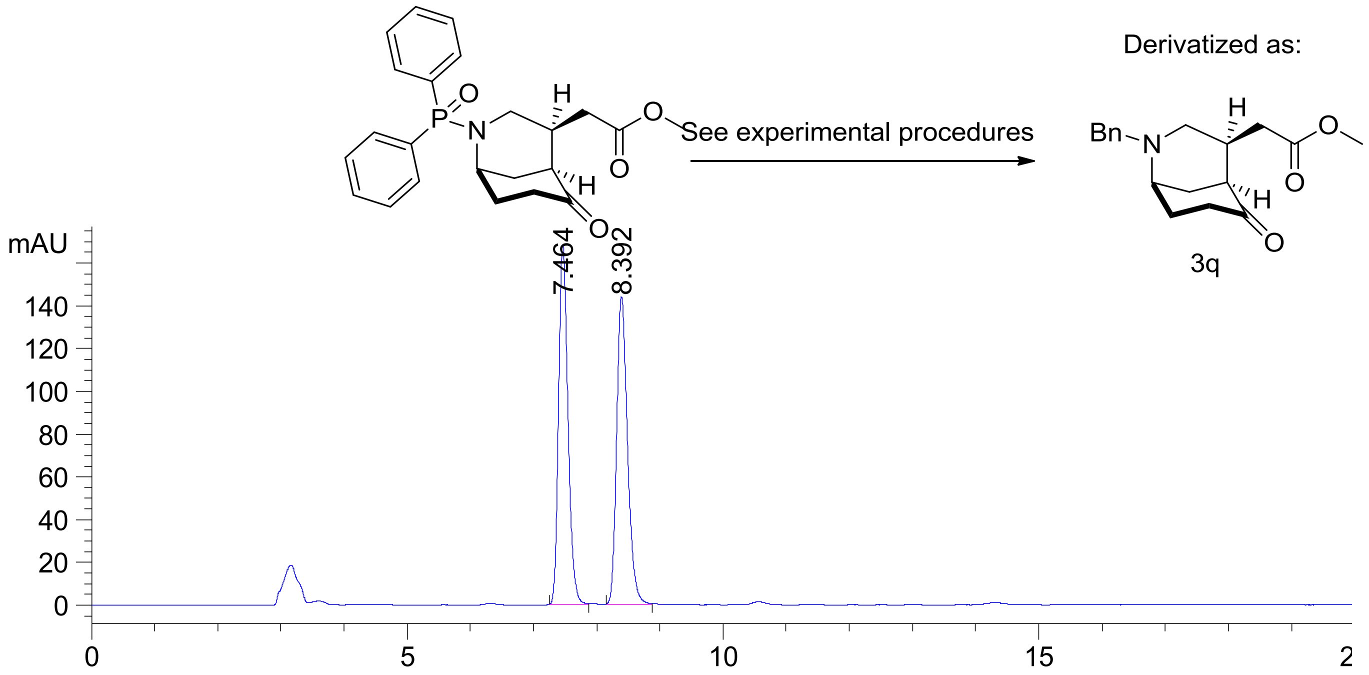

Signal 3: DAD1 C, Sig=210,8 Ref=360,100

Peak #	RetTime [min]	Type	Width [min]	Area [mAU*s]	Height [mAU]	Area %
1	7.464	BB	0.1578	1730.14514	168.51022	50.1176
2	8.392	BB	0.1837	1722.02551	143.79132	49.8824

Totals : 3452.17065 312.30154

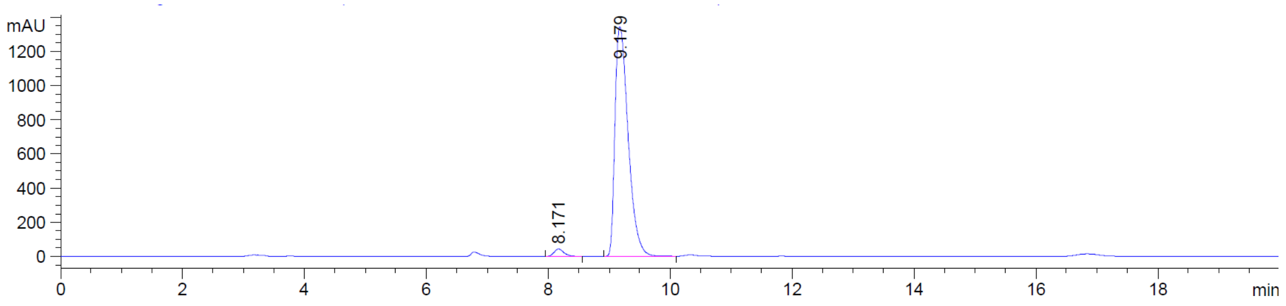

Signal 3: DAD1 C, Sig=210,8 Ref=360,100

Peak #	RetTime [min]	Type	Width [min]	Area [mAU*s]	Height [mAU]	Area %
1	8.171	BV	0.1783	500.23459	43.45150	2.4117
2	9.179	VV	0.2359	2.02415e4	1342.31384	97.5883

Totals : 2.07418e4 1385.76534

HPLC traces for racemic and enantioenriched 3u

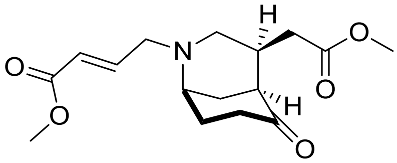

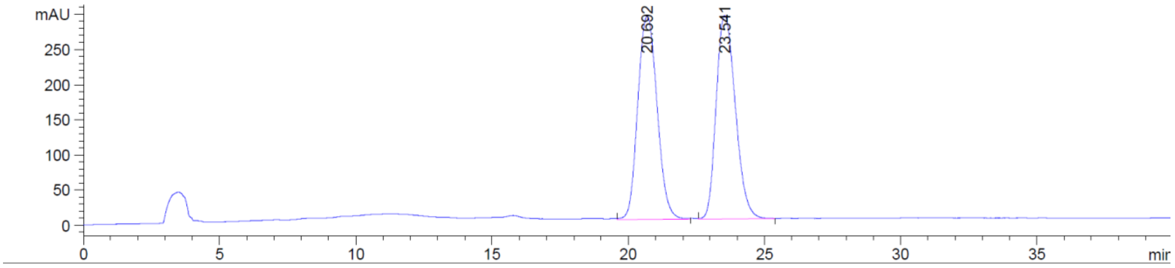

Signal 3: DAD1 C, Sig=210,8 Ref=360,100

Peak #	RetTime [min]	Type	Width [min]	Area [mAU*s]	Height [mAU]	Area %
1	20.692	BB	0.7881	1.42738e4	289.06870	50.1352
2	23.541	BB	0.7767	1.41968e4	289.20917	49.8648

Totals : 2.84705e4 578.27786

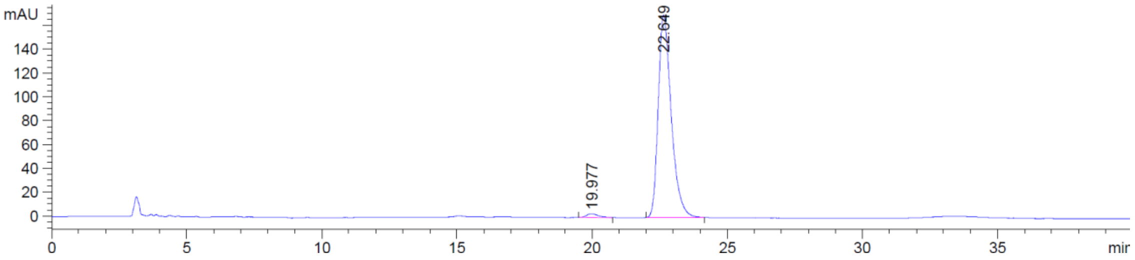

Signal 3: DAD1 C, Sig=210,8 Ref=360,100

Peak #	RetTime [min]	Type	Width [min]	Area [mAU*s]	Height [mAU]	Area %
1	19.977	BB	0.4132	96.51253	3.18834	1.6644
2	22.649	BB	0.5116	5702.29834	169.89583	98.3356

Totals : 5798.81087 173.08417

HPLC traces for racemic and enantioenriched 3v using Jacobsen's catalyst

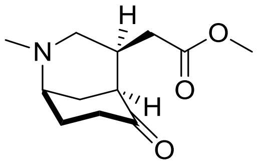

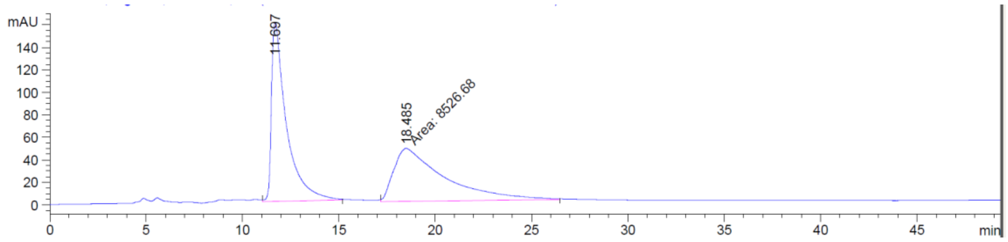

Signal 3: DAD1 C, Sig=210,8 Ref=360,100

Peak #	RetTime [min]	Type	Width [min]	Area [mAU*s]	Height [mAU]	Area %
1	11.697	VB	0.7577	8581.01465	158.99191	50.1588
2	18.485	MM	3.0188	8526.67969	47.07588	49.8412

Totals : 1.71077e4 206.06779

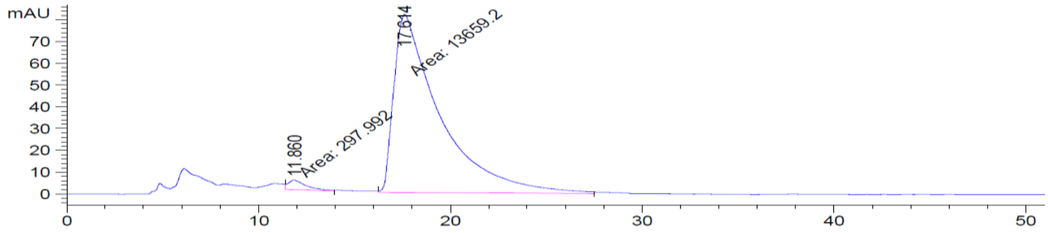

Signal 3: DAD1 C, Sig=210,8 Ref=360,100

Peak #	RetTime [min]	Type	Width [min]	Area [mAU*s]	Height [mAU]	Area %
1	11.860	FM	1.1238	297.99216	4.41943	2.1350
2	17.614	MM	2.7801	1.36592e4	81.88615	97.8650

Totals : 1.39572e4 86.30558

HPLC traces for racemic and enantioenriched 3v using 4l as catalyst

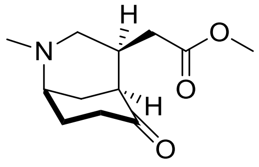

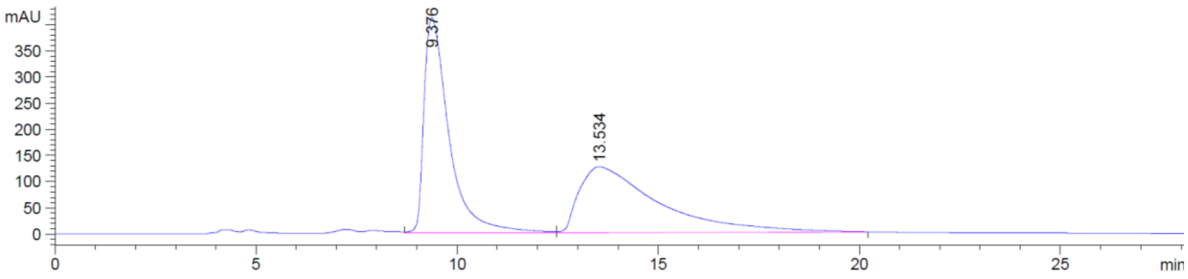

Signal 3: DAD1 C, Sig=210,8 Ref=360,100

Peak #	RetTime [min]	Type	Width [min]	Area [mAU*s]	Height [mAU]	Area %
1	9.376	VV	0.6637	1.79112e4	411.55771	50.8195
2	13.534	VB	1.9017	1.73335e4	126.25711	49.1805

Totals : 3.52447e4 537.81482

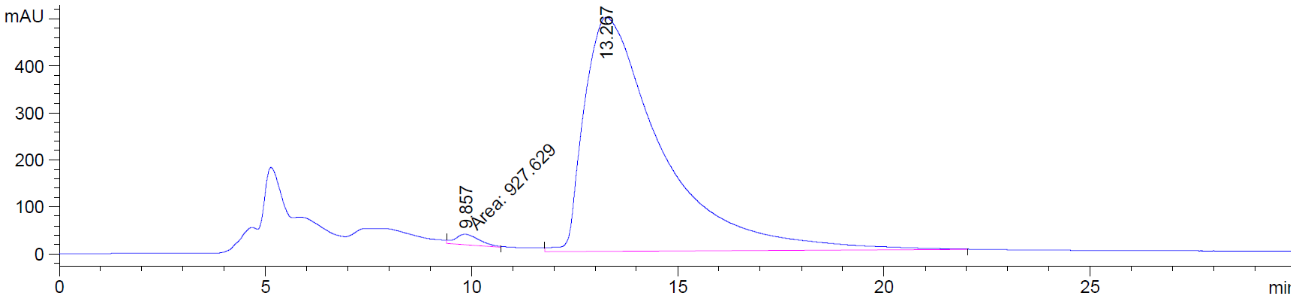

Signal 3: DAD1 C, Sig=210,8 Ref=360,100

Peak #	RetTime [min]	Type	Width [min]	Area [mAU*s]	Height [mAU]	Area %
1	9.857	FM	0.6844	927.62946	22.58878	1.4021
2	13.267	BB	1.9446	6.52328e4	497.86801	98.5979

Totals : 6.61605e4 520.45679

Computational Supporting Information:

Table of Contents

1. Computational Methods 176

3. Comparison of M06-2X and B3LYP-D3 Results 179

4. Analysis of Enantioselectivity 180

5. Computational References 181

6. Cartesian Coordinates 182

1. Computational Methods

All density functional theory (DFT) calculations were performed with the *Gaussian 09* program[1] using the M06-2X functional.[2] This is a hybrid meta-generalized gradient approximation (GGA) density functional, which through the inclusion of a local spin kinetic energy density term in the exchange-correlation functional, has been shown to be effective at modelling thermochemical and kinetic parameters, particularly where non-local dispersion interactions play a role.[3] The valence triple-ζ polarized 6-311+G(d,p) basis set[4] was used for all geometry optimizations throughout. Recent computational studies have shown that for enamine catalysis a smaller 6-31G(d) basis set is responsible for significant basis set superposition errors, and that corrections for dispersion (either by explicit DFT+D corrections or through functionals capturing medium-range correlation) are required for chemical accuracy.[5] Comparison of the stationary points and relative energetics obtained at this level of theory with other density functionals was performed, showing that our conclusions were unaffected. The B3LYP-D3/6-311+G(d,p)[6] computed free energy profile (with the zero-damping D3 method at short range) shown **in Fig. S3** illustrates this point. An implicit description of the reaction medium, dichloromethane, was included in all such optimizations through the use of a conductor-like polarizable continuum solvation model (CPCM).[7] Gibbs free energies were evaluated at 318 K (the reaction temperature), for which the vibrational entropy contributions were computed using a free-rotor approximation for low frequency modes. A smooth damping function centred about a frequency of 100 cm^{-1} was used to switch between the harmonic approximation for vibrations above this value and the free-rotor approximation below.[8] A conventional standard state of 1 mol/l in solution for all species was employed in calculating the translational entropies via the Sackur-Tetrode expression: in practice this has no effect on the computed mechanism since no associative or dissociative steps were explicitly calculated. Vibrational frequencies were computed for all stationary points to confirm them as either minima or transition structures (TSs), possessing zero or a single imaginary frequency, respectively. Intrinsic reaction coordinate (IRC) calculations[9] confirmed the connection of each TS to the appropriate species on either side of the barrier on the potential energy surface (PES). Molecular graphics have been produced with *Pymol*[10] and with *CYLview*.[11]

In computing the mechanism with a flexible thiourea catalyst, low energy conformations for each stationary point were located using a locally modified form of Still's Monte Carlo Multiple Minimum (MCMM) conformational search[12] employing a semi-empirical PM6-DH2 Hamiltonian[13] (with transferable corrections for dispersion and hydrogen-bonding) interfaced through Mopac.[14] In the case of transition structures, the "core" geometry previously established with methylamine were constrained while conformations of the flexible aminocyclohexylthiourea catalyst were sampled. Those conformations within 10.0 kJ/mol of the global minium located at this level of theory were subsequently reoptimized to minima or saddle points as appropriate at the M06-2X/6-31G(d) level, and then again at the which the M06-2X/6-311+G(d,p) level of theory.

2. Full free energy profile for primary amine catalyzed intramolecular-Michael addition

We have used DFT calculations to investigate the mechanism of cyclization catalyzed by an achiral amine, here modelled as methylamine (*n*-propylamine was used experimentally), and to understand the exclusive formation of the *endo*-diastereomer. All species have been optimized at the M06-2X/6-311+G(d,p) level of theory including a continuum description of dichloromethane solvent, and the resulting free energy profile is shown in **Figure S1**.

The condensation of ketoester substrate **01** with methylamine is computed to be marginally endergonic (by 0.7 kcal/mol) in forming ketimine **02.** There are two possible enamine configurations which may then form, of which the *s-cis* enamine **03** (the methyl group eclipsing the C=C bond) is more stable than the *s-trans* enamine **04** (the N-H bond eclipsing the C=C bond) by 2.9 kcal/mol. We have not computed the activation barriers for these steps since relative stereochemistry in the product is not set at this stage of the reaction and so these steps occur prior to the stereoselectivity-determining step(s). However, we note that computations have been used previously to suggest that the presence of an acid co-catalyst will accelerate condensation and imine:enamine tautomerization steps.[15]

Competing pathways leading to C-C bond formation were explored fully for both (*s-cis* and *s-trans*) enamine configurations. For *s-cis* enamine **03**, the only possible intramolecular reaction was found to be an "ene-like" process (via **TS-12**), in which C-C formation and N-H···Cα proton transfer occur in concert to form the *endo*-adduct. However, we may confidently assert that, with a computed activation barrier upwards of 30 kcal/mol, this reaction will not take place. The *s-trans* enamine, however, was found to have much lower barrier(s) towards C-C bond formation due to the favorable orientation of the N-H bond towards the developing oxyanionic group of the ester. Thus, a Curtin-Hammett scenario[16] emerges in which cyclization occurs exclusively through the thermodynamically less favorable, but considerably more reactive *s-trans* enamine. How then, does this lead to complete control over diastereoselectivity?

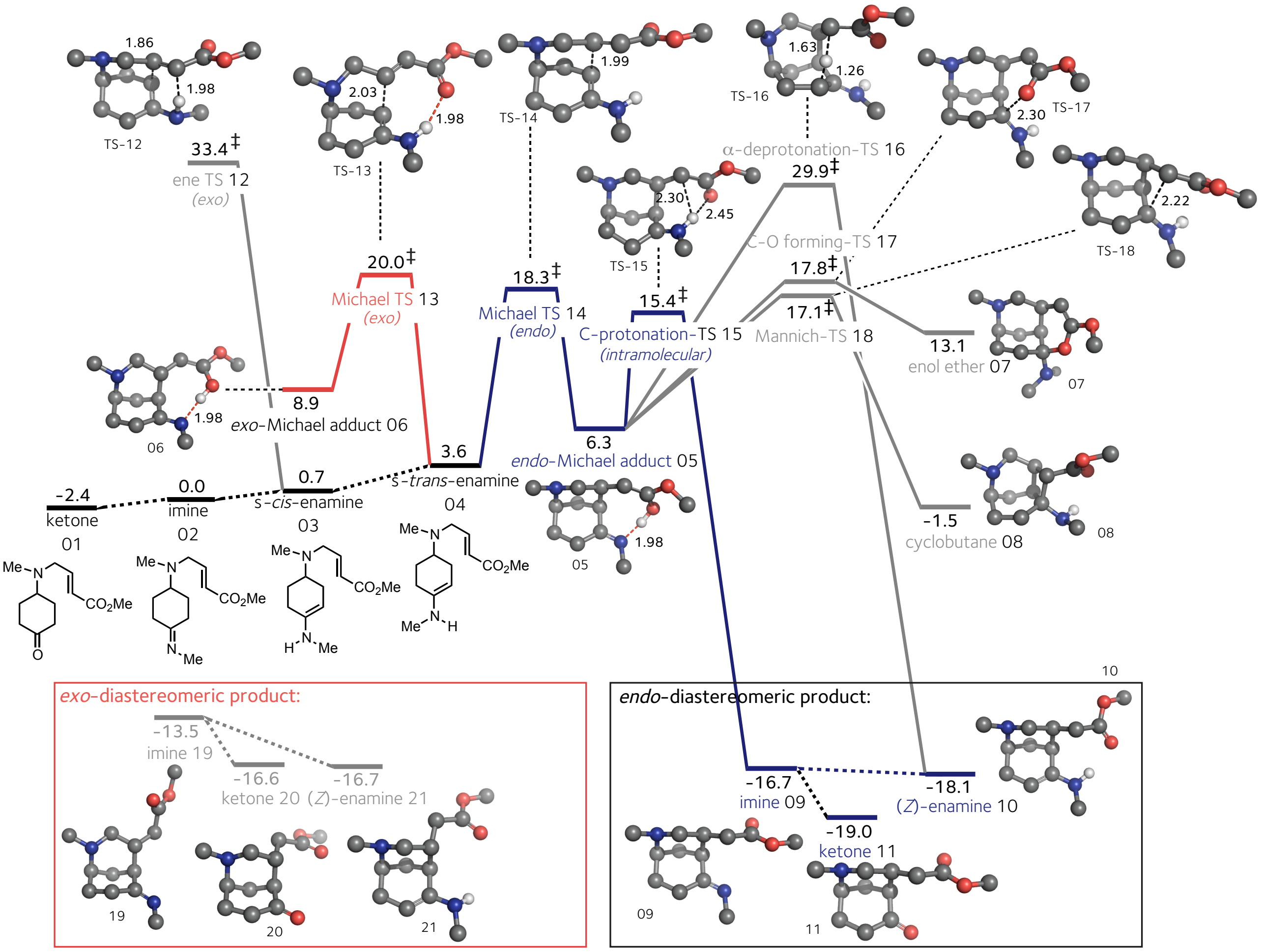

Figure S1. CPCM-M06-2X/6-311+G(d,p) computed free energy profile for diastereoselective intramolecular Michael addition catalyzed by methylamine (G_{rel} in kcal/mol at 45 °C, 1 mol/l). Optimized structures shown with selected distances in Å.

Formation of the major *endo*-Michael adduct was found to proceed via selectivity-determining **TS-14**. Conjugate addition of the enamine to the β-carbon in this TS occurs with a perfectly staggered conformation (**Figure S2**) about the forming C-C bond and has an activation barrier of 14.7 kcal/mol relative to enamine **03**. This TS leads (as established by IRC calculation) to N-H proton transfer to the oxygen atom of the ester-enolate in intermediate **05**, which is stabilized by an intramolecular enolic OH···N hydrogen bond. The most likely fate of this intermediate is then an irreversible imine-assisted tautomerization via **TS-15**, with a barrier of 9.1 kcal/mol, to form the *endo*-ester adduct **09,** which is significantly exergonic with respect to starting iminium/enamine. Interestingly, gas-phase optimizations performed with smaller basis sets, such as 6-31G(d), indicated that evolution from **TS-14** leads directly to imine **09,** without an intervening intermediate rather than the stepwise mechanism obtained with larger basis sets and inclusion of solvent. Nevertheless, all levels and basis sets examined confirm that reaction via **TS-14** will be the dominant pathway.

With a primary amine organocatalyst, the enamine has a labile N-H proton which is readily transferred to the ester-enolate upon conjugate addition. For the major *endo*-diastereromer, Cα-protonation is rapid and irreversible and, importantly, does not require an external proton source to achieve this step. Note that this mechanistic scenario differs from that encountered in the Michael additions of pyrrolidine-enamines to nitro-olefins since there is no comparable labile N-H proton, and so protonolysis by an acidic co-catalyst (of the dihydrooxazine oxide intermediate formed) following conjugate addition is turnover-limiting.[17] In the case for organocatalysts with an internal carboxylic acid, or where strongly acidic additives are used, C-C bond formation has been found as rate-limiting in conjugate additions of aldehyde-derived enamines to nitroolefins.[18] With primary enamines the iminium species generated upon conjugate addition has an acidic N-H proton which is transferred to the Michael-adduct making this C-C bond formation irreversible and hence selectivity determining.

We were able to locate transition structures and intermediates corresponding to the formation of two cyclic intermediates, cyclobutane **08** and enol ether **07**, both of which have been observed experimentally in the Michael additions of pyrrolidine-enamines to nitro-olefins.[19] Here, however, we predict that both are kinetically disfavoured with respect to internal proton-transfer (**TS-15**) and as a result, are **not** formed. The cyclobutane intermediate, however, is predicted to be thermodynamically more stable than the reactant enamine by 5.1 kcal/mol, while the cyclic 6-membered enol ether lies much higher in free energy.

The *exo*-reaction pathway, via **TS-13,** which has a higher activation barrier than **TS-14** of 16.4 kcal/mol, is kinetically disfavored relative to the *endo*-pathway. Although the N-H proton is able to stabilize the developing oxyanion, eclipsing interactions occur about the incipient C-C bond (**Fig. S2**), which result from adopting a conformation necessary to maintain the NH···O interaction. No other conformation of this TS was detected. Also important is the fact that, unlike the *endo*-diastereomer, there is no analogous intramolecular mechanism leading to protonation of the ester α-carbon (since this is oriented too far away from the iminium) and so the most likely fate of *exo*-adduct **6** is a retro-Michael opening back to the starting enamine. Thus we may conclude that the exclusive favorability of the *endo*-diasteromer results from irreversible, stepwise, C-C bond formation and intramolecular proton transfer, in which selectivity-determining **TS-14** is perfectly staggered. The C-C bond forming step leading to the minor, *exo*-diastereomer is kinetically disfavored and intramolecular proton transfer is not geometrically possible, such that this pathway is reversible.

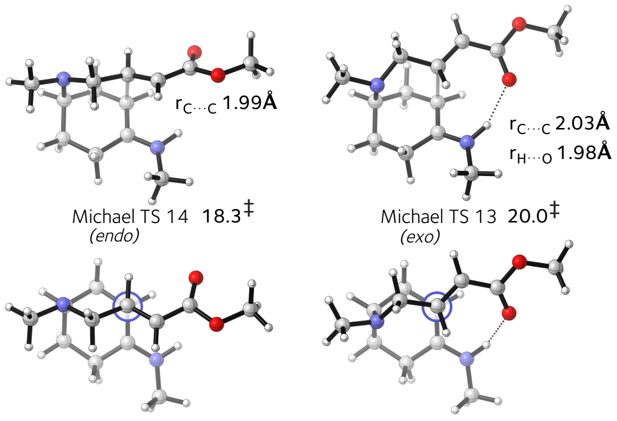

Figure S2. Analysis of the competing diastereomeric Michael transition structures (CPCM-M06-2X/6-311+G(d,p)) for formation of the *exo*- and *endo*-Michael adducts.

Once formed, *endo*-adduct **09** is sufficiently stable such that reverse (i.e. retro-Michael) reaction is highly improbable. The relative stability of product enamine **10** with respect to **09** (1.4 kcal/mol lower in free energy) is such that, if tautomerization is rapid with respect to hydrolysis, it would be an observable intermediate. Hydrolysis to afford ketone **11** and regenerate the amine catalyst is computed to be exergonic.

3. Comparison of M06-2X and B3LYP-D3 results

All stationary points located on the M06-2X potential energy surface for methylamine-catalyzed conjugate addition were subsequently reoptimized at the B3LYP-D3 level of theory, using the same 6-311+G(d,p) basis set and implicit treatment of dichloromethane solvation. The recomputed free energy profile is shown below in **Fig. S3**. The geometries of all stationary points are not substantially altered between M06-2X and B3LYP-D3, and the relative energetics from both approaches lead to identical mechanistic conclusions. The *endo*-Michael pathway is kinetically favored and irreversible, while the *exo*-pathway is reversible. Alternative cyclization pathways, forming 4-membered cyclobutane or 6-membered enol ether intermediates are viable but disfavored relative to proton transfer which irreversibly forms the *endo*-Michael adduct. This validation gives us confidence that the conclusions are robust with regard to the use of different model chemistries.

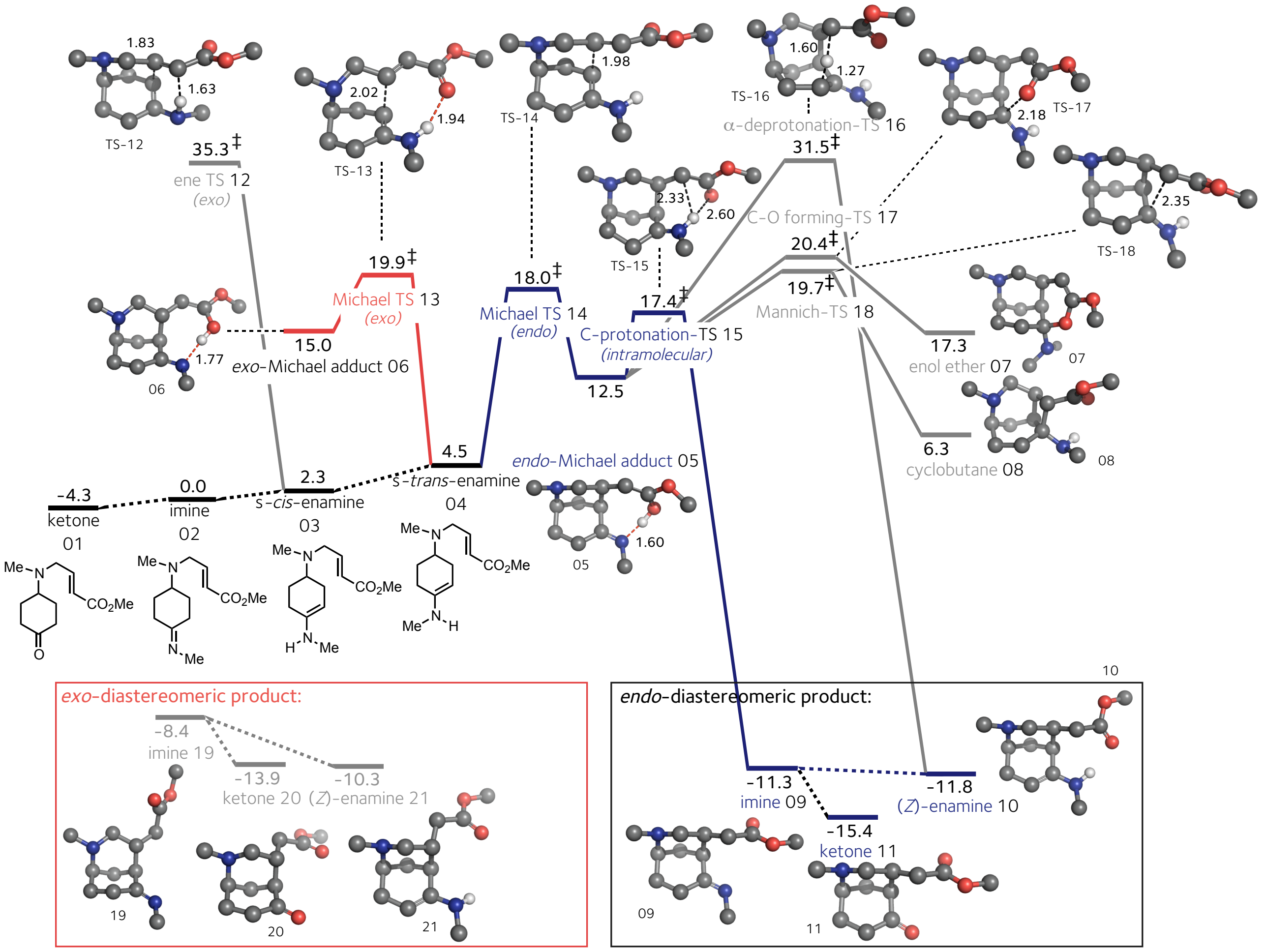

Figure S3. B3LYP-D3/6-311+G(d,p) computed free energy profile for diastereoselective intramolecular Michael addition catalyzed by methylamine (G_{rel} in kcal/mol at 45 °C, 1 mol/l). Optimized structures shown with selected distances in Å.

4. Analysis of enantioselectivity

Stereoselective intramolecular conjugate addition was studied with a chiral aminocyclohexylthiourea catalyst, with the resulting free energy profile shown in **Fig. S4**. With a chiral primary amine the absolute sense of stereoinduction could, in principle, be set during enamine formation since substrate desymmetrization takes place. However, this would require irreversible enamine formation, and in our experiments a (*Z*)-configured Michael acceptor was found to react with no enantioinduction, implicating the participation of this functional group in determining the stereochemistry. We located TSs in which C-C bond formation occurs leading to four diastereomeric Michael adducts. As for methylamine catalysis, formation of the *exo*-adducts was found to be disfavoured relative to the *endo*-adducts, and so only formation of the major diastereomer (both enantiomers) is displayed in **Fig. S4**. The geometries and relative free energies for all stereoisomeric TSs are shown in **Fig. S5**.

With a thiourea present in the catalyst the mechanism of cyclization is stepwise: C-C bond formation via a Michael addition occurs (**TS-26** and **TS-32**) and is then proceeded by intramolecular proton transfer to the ester α-carbon (**TS-28** and **TS-34**). As was found earlier, the Michael adducts lie far below the starting enamines in free energy and so the cyclization is predicted to be irreversible once proton transfer has taken place. For the formation of the two enantiomers shown in **Fig. S5** the stereodetermining step is predicted to be proton-transfer from thiourea to the ester enolate, which, with a free energy difference $\Delta\Delta G^{\ddagger}$ of 2.4 kcal/mol leads to a computed enantioselectivity of 96% ee. Experimentally, a truncated thiourea catalyst **4l** leads to formation of this enantiomer in 96% ee. We predict intermolecular protonation will be uncompetitive since the barrier to intramolecular proton transfer is smaller than the entropic cost alone (ca. 12 kcal/mol) associated with a bimolecular process.

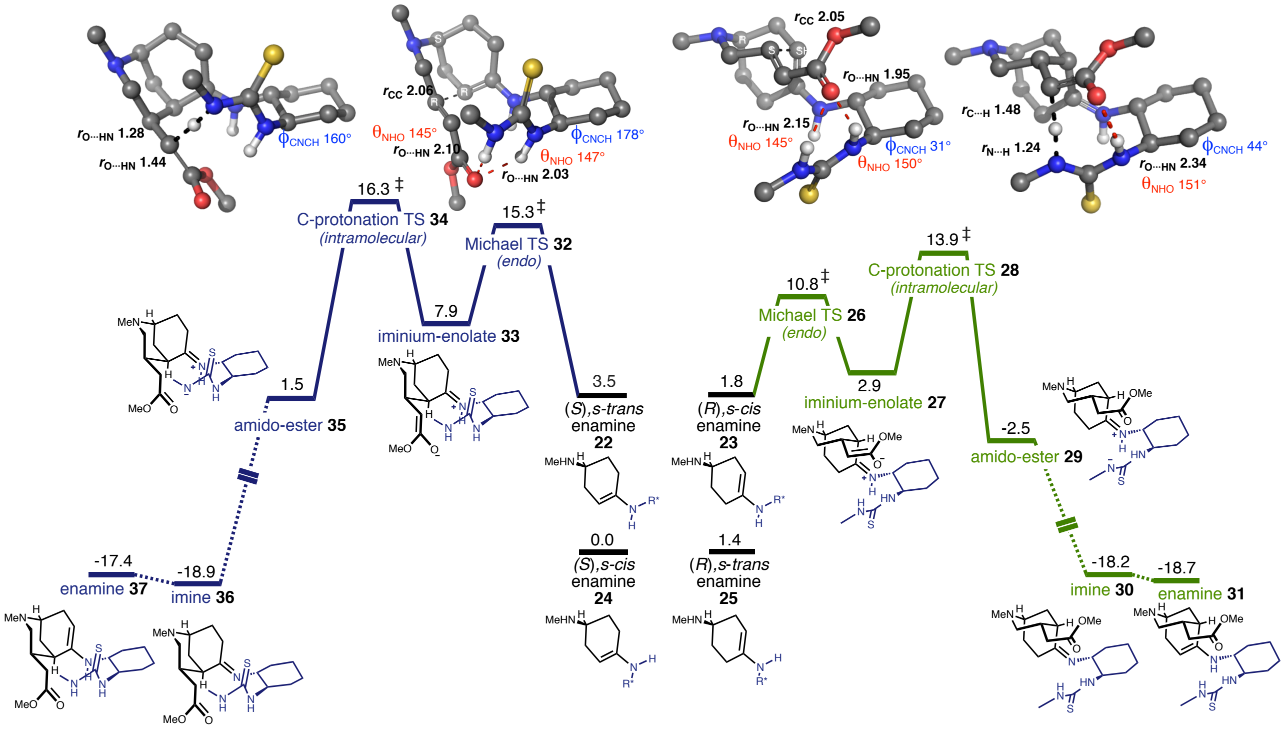

Figure S4. CPCM-M06-2X/6-311+G(d,p) computed free energy profile for diastereoselective intramolecular Michael addition catalyzed by thiourea **4l** (G_{rel} in kcal/mol at 45 °C, 1 mol/l). Optimized structures shown with selected distances in Å.

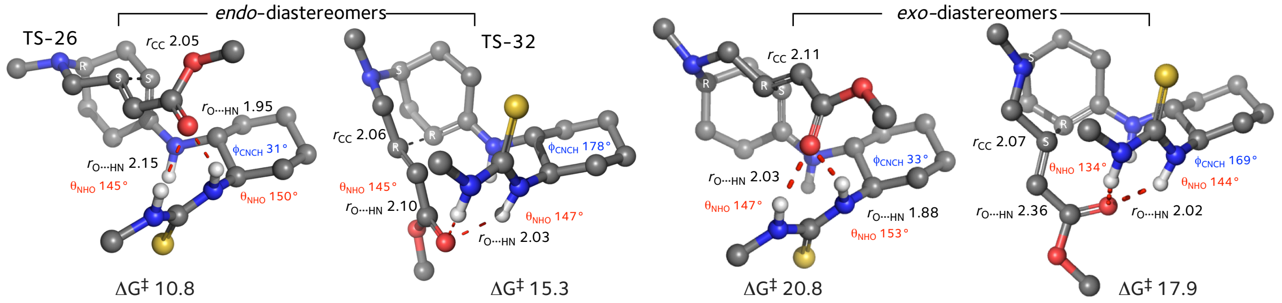

Figure S5. CPCM-M06-2X/6-311+G(d,p) diastereomeric TSs for the formation of pairs of *endo*- and *exo*-enantiomers. Optimized structures shown with selected distances in Å.

Both the major and minor enantiomer of the *endo*-Michael adduct benefit from the stabilizing hydrogen-bonding interactions between ester and thiourea and so we must look elsewhere to explain the energetic preference. The enamine configuration differs between the two pathways, being *s-cis* for the major and *s-trans* for the minor enantiomers, however, in the achiral case studied (methylamine catalysis) the latter of these enamine configurations was computed to be more stable and so this geometric difference is unlikely to account for the preference for the former. We have discovered that the origins of stereoinduction instead lie in the conformation of the cyclohexylthiourea, which has a 4 kcal/mol preference for the cyclohexyl C-H to lie nearly syn-coplanar to the N-C(=S) bond. The conformational energy profile about the (S=)C-N-C-H dihedral angle is shown below. In forming the major enantiomer both **TS-26** and **TS-28** adopt conformations in which this preference is satisfied, while for the minor enantiomer both **TS-32** and **TS-34** enforce disfavoured cyclohexylthiourea conformations in which CH and NH bonds are staggered.

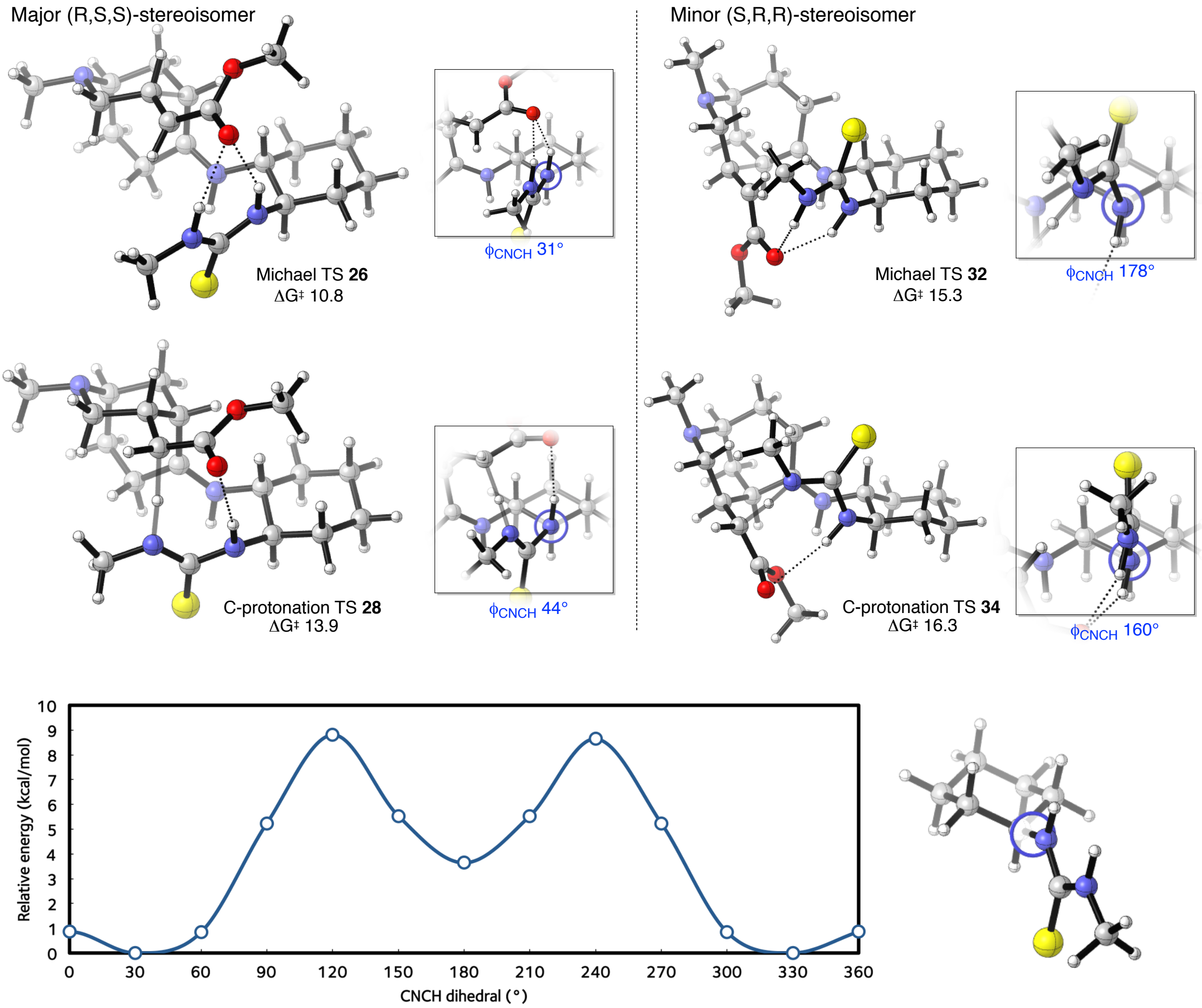

Figure S6. Analysis of the competing thiourea-catalyzed transition structures (CPCM-M06-2X/6-311+G(d,p)) leading to formation of the *enantiomeric endo*-Michael adducts. M06-2X/6-311+G(d,p) relaxed dihedral scan shown for cyclohexylthiourea.

Formation of the major enantiomer is also possible via an *s-trans* enamine configuration (**Fig. S7**), although since no hydrogen-bonding interactions are possible with the ester group this pathway is disfavoured by 6.3 kcal/mol relative to **TS-26**. This *s-trans* TS is less stable than proton transfer in **TS-28** and so is not populated to any significant degree. This alternative pathway is essentially the same as that computed for the methylamine-catalyzed case and has a similar activation barrier. Alternative mechanisms were considered involving the acid cocatalyst, in which the acidic proton activates the ester group towards conjugate addition or a protonated enammonium species undergoes an ene-type reaction. Both alternatives were found to possess larger activation barriers (25.1 and 16.8 kcal/mol, respectively) and so were disregarded.

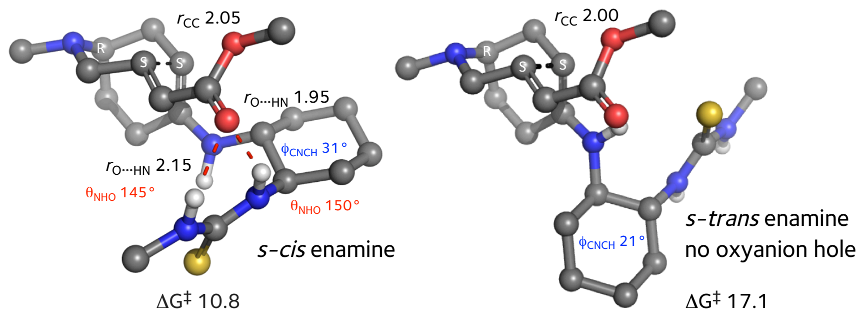

Figure S7. CPCM-M06-2X/6-311+G(d,p) transition structures leading to the major enantiomer. Hydrogen bonding between the thiourea and Michael acceptor is only possible via an *s-cis* enamine configuration and not in the *s-trans* configuration.

5. Computational References

1. *Gaussian 09*, Revision D.01, M. J. Frisch, G. W. Trucks, H. B. Schlegel, G. E. Scuseria, M. A. Robb, J. R. Cheeseman, G. Scalmani, V. Barone, B. Mennucci, G. A. Petersson, H. Nakatsuji, M. Caricato, X. Li, H. P. Hratchian, A. F. Izmaylov, J. Bloino, G. Zheng, J. L. Sonnenberg, M. Hada, M. Ehara, K. Toyota, R. Fukuda, J. Hasegawa, M. Ishida, T. Nakajima, Y. Honda, O. Kitao, H. Nakai, T. Vreven, J. A. Montgomery, Jr., J. E. Peralta, F. Ogliaro, M. Bearpark, J. J. Heyd, E. Brothers, K. N. Kudin, V. N. Staroverov, R. Kobayashi, J. Normand, K. Raghavachari, A. Rendell, J. C. Burant, S. S. Iyengar, J. Tomasi, M. Cossi, N. Rega, J. M. Millam, M. Klene, J. E. Knox, J. B. Cross, V. Bakken, C. Adamo, J. Jaramillo, R. Gomperts, R. E. Stratmann, O. Yazyev, A. J. Austin, R. Cammi, C. Pomelli, J. W. Ochterski, R. L. Martin, K. Morokuma, V. G. Zakrzewski, G. A. Voth, P. Salvador, J. J. Dannenberg, S. Dapprich, A. D. Daniels, Ö. Farkas, J. B. Foresman, J. V. Ortiz, J. Cioslowski, and D. J. Fox, Gaussian, Inc., Wallingford CT, 2009.
2. Zhao, Y.; Truhlar, D. G. *Theor. Chem. Acc.* **2008**, *120*, 215.
3. (a) Pieniazek, S. N.; Houk, K. N. *Angew. Chem. Int. Ed.* **2006**, *45*, 1442-1445; (b) Pieniazek, S. N.; Clemente, F. R.; Houk, K. N. *Angew. Chem. Int. Ed.* **2008**, *47*, 7746-7749 (c) Paton, R. S.; Mackey, J. L.; Kim, W. H.; Lee, J. H.; Danishefsky, S. J.; Houk, K. N. *J. Am. Chem. Soc.* **2010**, *132*, 9335; (d) Paton, R. S.; Kim, S.; Ross, A. G.; Danishefsky, S. J.; Houk, K. N. *Angew. Chem. Int. Ed.* **2011**, *44*, 10366; (e) Paton, R. S.; Steinhardt, S. E.; Vanderwal, C. D.; Houk, K. N. *J. Am. Chem. Soc.* **2011**, *133*, 3895; (f) Albrecht, Ł.; Dickmeiss, G.; Cruz Acosta, F.; Rodríguez-Escrich, C.; Davis, R. L.; Jørgensen, K. A. *J. Am. Chem. Soc.* **2012**, *134*, 2543–6; (g) Hong, X.; Liang, Y.; Griffith, A. K.; Lambert, T. H.; Houk, K. N. *Chem. Sci.* **2014**, *5*, 471; (h) Dieckmann, A.; Breugst, M.; Houk, K. N. *J. Am. Chem. Soc.* **2013**, *135*, 3237–42.
4. (a) McLean, A. D.; Chandler, G. S. *J. Chem. Phys.* **1980**, *72*, 5639; (b) Raghavachari, K.; Binkley, J. S.; Seeger, R.; Pople, J. A. *J. Chem. Phys.* **1980**, *72*, 650.
5. Armstrong, A.; Boto, R. A.; Dingwall, P.; Contreras-García, J.; Harvey, M. J.; Mason N. J.; Rzepa, H. S. *Chem. Sci.* **2014**, **5**, 2057-2071.
6. (a) Becke, A.D. *J. Chem. Phys.* **1993**, *98*, 5648; (b) Lee, C.; Yang, W.; Parr, R. G. *Phys. Rev. B* **1988**, *37*, 785-789; (c) Vosko, S. H.; Wilk, L.; Nusair, M. *Can. J. Phys.* **1980**, *58*, 1200-1211; (d) Stephens, P. J.; Devlin, F. J.; Chabalowski, C. F.; Frisch, M. J. *J.Phys.Chem.* **1994**, *98*, 11623-11627. D3-correction: (e) Grimme, S.; Antony, J.; Ehrlich, S.; Krieg, H. *J. Chem. Phys.* **2010**, *132*, 154104-154114; (f) Grimme, S.; Ehrlich, S.; Georigk, L. *J. Comp. Chem.* **2011**, *32*, 1456-1465.
7. (a) Barone, V.; Cossi, M. *J. Phys. Chem. A* **1998**, *102*, 1995; (b) Cossi, M.; Rega, N.; Scalmani, G.; Barone, V. *J. Comput. Chem.* **2003**, *24*, 669; (c) solute cavities were defined by UFF atomic radii: Y. Takano, K. N. Houk. *J. Chem. Theory. Comput.* **2005**, *1*, 70.
8. (a) Ribeiro, R. F.; Marenich, A. V.; Cramer, C. J.; Truhlar D. G. *J. Phys. Chem. B*, **2011**, *115*, 14556; (b) Grimme, S. *Chem. Eur. J.* **2012**, *18*, 9955.
9. (a) Hratchian, H. P.; Schlegel, H. B. *J. Chem. Phys.* **2004**, *120*, 9918; (b) Hratchian, H. P.; Schlegel, H. B. *J. Chem. Theor. Comp.* **2005**, *1*, 61.
10. The PyMOL Molecular Graphics System, Version 1.5.0.4 Schrödinger, LLC.
11. CYLview v1.0.561, Legault, C. Y. University of Sherbrooke, Canada, 2012
12. Chang, G.; Guida, W. C.; Still, W. C. *J. Am. Chem. Soc.* **1989**, *111*, 4379-4386.
13. (a) Korth, M.; Pitonák, M.; Rezác, J.; Hobza, P. *J. Chem. Theory Comput.* **2010**, *6*, 344; (b) Rezác, J.; Fanfrlik, J.; Salahub, D.; Hobza, P. *J. Chem. Theory Comput.* **2009**, *5*, 1749.
14. MOPAC2012, Stewart, J. J. P. Stewart Computational Chemistry, Colorado Springs, CO, USA, http://OpenMOPAC.net

15. Patil, M.P.; Sunoj, R. B. *J. Org. Chem.* **2007**, *72*, 8202.
16. Seeman, J. I. *Chem. Rev.* **1983**, *83*, 83.
17. Sahoo, G.; Rahaman, H.; Madarász, A.; Pápai, I.; Melarto, M.; Valkonen, A.; Pihko, P. M. *Angew. Chem. Int. Ed.* **2012**, *51*, 13144.
18. Duschmalé. J; Wiest, J.; Wiesner, M.; Wennemers, H. *Chem. Sci.* **2013**, *4*, 1312.
19. (a) Patora-Komisarska, K.; Benohoud, M.; Ishikawa, H.; Seebach, D.; Hayashi, Y. *Helv. Chim. Acta* **2011**, *94*, 719-745. (b) Burés, J.; Armstrong, A.; Blackmond, D. G. *J. Am. Chem. Soc.* **2011**, 133, 8822–8825. Burés, J.; Armstrong, A.; Blackmond, D. G. *J. Am. Chem. Soc.* **2012**, 134, 6741–6750. (d) Seebach, D.; Sun, X.; Ebert, M.; Schweizer, W. B.; Purkayastha, N.; Beck, A. K.; Duschmale, J. *Helv. Chim. Acta* **2013**, *96*, 795-852.

6. Cartesian Coordinates

CPCM-M06-2X/6-311+G(d,p) optimized stationary points; energy and G(318K) in Hartree; imaginary frequencies (where appropriate) in wavenumbers. All structures are also supplied as separate MOL2 formatted files in the accompanying zip archive.

01:

E = -749.02529; G = -748.76557

C	-2.23725	-1.43388	-1.16261
C	-1.88542	0.06144	-1.18058
H	-2.66044	-1.76735	-2.11039
H	-1.13155	0.24315	-1.95098
C	-1.38206	0.51926	0.19740
C	-3.21255	-1.74982	-0.04928
C	-2.82560	-1.22455	1.31614
C	-2.45938	0.26617	1.25369
H	-0.52395	-0.11084	0.46343
H	-3.36130	0.84001	1.01863
H	-2.10003	0.60387	2.22776
H	-3.64083	-1.41507	2.01421
H	-1.32625	-2.01440	-0.97097
H	-2.77580	0.63727	-1.45493
N	-0.89585	1.90575	0.25558
C	-1.85519	2.89841	-0.21711
H	-2.81817	2.76427	0.27499
H	-1.48712	3.89474	0.03557
H	-2.01227	2.86050	-1.30590
C	0.38099	2.08752	-0.42822
H	0.32133	1.88791	-1.51063
H	0.65616	3.14312	-0.31824
C	1.47227	1.26770	0.18611
H	1.55464	1.29881	1.27096
C	2.34308	0.53732	-0.50671
H	2.30279	0.45751	-1.58744
C	3.42030	-0.20626	0.18630
O	4.18640	-0.87236	-0.68682
O	3.60644	-0.23006	1.37929
C	5.26502	-1.62562	-0.12277
H	5.76818	-2.09552	-0.96289
H	5.94657	-0.96363	0.41148
H	4.87951	-2.37930	0.56366
H	-1.95005	-1.79669	1.64696
O	-4.23231	-2.37442	-0.23978

02:

E = -768.43425; G = -768.13664

C	-2.09711	-0.92630	-1.36838
C	-1.54167	0.50058	-1.25667
H	-2.55904	-1.09885	-2.34081
H	-0.78095	0.65983	-2.02611
C	-0.95918	0.74609	0.14294
C	-3.11499	-1.19126	-0.28516
C	-2.60163	-0.91817	1.11272
C	-2.03246	0.50746	1.20685
H	-0.17639	-0.00463	0.31077
H	-2.85249	1.22206	1.08128
H	-1.60128	0.67251	2.19672
H	-3.37270	-1.06780	1.86686
H	-1.27195	-1.64004	-1.25146
H	-2.35207	1.21324	-1.44590
N	-0.31066	2.05415	0.33086
C	-1.13511	3.19244	-0.06040
H	-2.12332	3.12066	0.39386
H	-0.66724	4.11105	0.29952
H	-1.26059	3.27882	-1.15057
C	0.99343	2.13807	-0.31782
H	0.94005	2.01514	-1.41204
H	1.37985	3.14773	-0.13468
C	1.97432	1.16707	0.26209
H	2.01499	1.10553	1.34803
C	2.79974	0.41108	-0.45836

H	2.79747	0.42007	-1.54252
C	3.77431	-0.48318	0.20729
O	4.52390	-1.13512	-0.69105
O	3.90003	-0.62848	1.39973
C	5.50686	-2.02670	-0.15451
H	6.01847	-2.45285	-1.01272
H	6.20651	-1.47914	0.47678
H	5.02519	-2.80957	0.43129
N	-4.27931	-1.58035	-0.61222
C	-5.26802	-1.83128	0.43360
H	-4.93111	-2.59626	1.14003
H	-5.49940	-0.92325	0.99945
H	-6.18869	-2.18185	-0.03118
H	-1.79789	-1.63573	1.31845

03:

E = -768.43316; G = -768.13552

C	2.20213	-0.54704	1.41739
C	1.68692	0.87744	1.22856
H	2.83989	-0.60414	2.30650
H	0.99487	1.13166	2.03657
C	1.00868	1.00037	-0.14056
C	2.97989	-1.03230	0.21716
C	2.92405	-0.40802	-0.97171
C	2.04318	0.78708	-1.24387
H	0.28513	0.18014	-0.22124
H	2.65907	1.68774	-1.35768
H	1.51782	0.65784	-2.19517
H	3.51394	-0.77822	-1.80288
H	1.36534	-1.23302	1.59369
H	2.52706	1.57842	1.28305
N	0.24763	2.24258	-0.35833
C	0.97250	3.45322	0.01227
H	1.96414	3.45714	-0.44023
H	0.42918	4.32407	-0.36042
H	1.09121	3.56566	1.10114
C	-1.05427	2.22361	0.29877
H	-0.98233	2.09917	1.39210
H	-1.52009	3.20086	0.12456
C	-1.96221	1.18179	-0.27738
H	-1.99842	1.11354	-1.36303
C	-2.72972	0.37087	0.44744
H	-2.72430	0.38352	1.53155
C	-3.63547	-0.59782	-0.21038
O	-4.31641	-1.31305	0.69504
O	-3.76442	-0.74972	-1.40161
C	-5.22207	-2.28823	0.16862

H	-5.67633	-2.76550	1.03218
H	-5.98201	-1.80434	-0.44492
H	-4.68125	-3.01884	-0.43273
N	3.68938	-2.20977	0.45027
H	4.04330	-2.28310	1.39389
C	4.60403	-2.70222	-0.56021
H	4.04661	-2.99883	-1.45205
H	5.34998	-1.95330	-0.85895
H	5.11908	-3.58051	-0.17348

04:

E = -768.42945; G = -768.13098

C	-2.38475	-0.68608	-1.07577
C	-1.86863	0.75154	-1.10276
H	-3.10906	-0.84412	-1.88086
H	-1.25185	0.90677	-1.99272
C	-1.07862	1.05384	0.17568
C	-3.04195	-1.02130	0.24188
C	-2.86392	-0.27143	1.33887
C	-2.01253	0.97120	1.38191
H	-0.33297	0.25921	0.29784
H	-2.64876	1.86399	1.42677
H	-1.40886	0.98112	2.29428
H	-3.37126	-0.55944	2.25669
H	-1.55921	-1.38445	-1.25985
H	-2.72039	1.43712	-1.17112
N	-0.33026	2.32191	0.16635
C	-1.10703	3.46500	-0.30100
H	-2.06028	3.51729	0.22523
H	-0.55554	4.38291	-0.08775
H	-1.31218	3.43255	-1.38219
C	0.91949	2.22620	-0.58052
H	0.77090	1.93119	-1.63262
H	1.36707	3.22694	-0.59442
C	1.89632	1.30374	0.08045
H	2.04182	1.43644	1.15088
C	2.59470	0.36468	-0.55412
H	2.47993	0.17454	-1.61544
C	3.56745	-0.47484	0.18136
O	4.15068	-1.36504	-0.63271
O	3.82098	-0.39530	1.35945
C	5.10829	-2.23516	-0.02093
H	5.46809	-2.88323	-0.81490
H	5.92847	-1.65478	0.40158
H	4.63530	-2.82093	0.76718
N	-3.90988	-2.12938	0.21600
H	-4.25344	-2.33935	1.14554

C	-3.43344	-3.33888	-0.45173
H	-3.30131	-3.15296	-1.51819
H	-2.48329	-3.70935	-0.04462
H	-4.18783	-4.11751	-0.34164

05:

E = -768.43049; G = -768.12655

C	-1.40622	1.69272	-0.71924
C	-2.80232	1.22185	-0.27282
H	-1.16263	1.28238	-1.70705
H	-3.25633	2.00712	0.33859
C	-2.81836	-0.05826	0.58368
C	-0.25417	1.31639	0.18637
C	-0.40657	0.16100	1.15824
C	-1.82705	0.11861	1.72904
H	-3.82776	-0.15722	0.99526
H	-1.91066	-0.71146	2.43458
H	-2.05125	1.04330	2.26863
H	0.31491	0.30589	1.96306
H	-1.40979	2.77738	-0.85147
H	-3.44444	1.10937	-1.14918
N	-2.54193	-1.32428	-0.10505
C	-1.17881	-1.47673	-0.60213
H	-1.06561	-2.51026	-0.94459
H	-0.98192	-0.84070	-1.48286
C	-0.12622	-1.19933	0.47751
H	-0.29439	-1.94595	1.26642
C	1.25028	-1.42865	-0.09787
H	1.32868	-2.25801	-0.79358
C	2.42692	-0.86871	0.22256
O	3.56715	-1.44934	-0.25256
O	2.66058	0.19400	1.00316
C	4.60446	-0.55160	-0.63878
H	4.22970	0.18211	-1.35869
H	5.02145	-0.03321	0.22426
H	5.37024	-1.16504	-1.10899
N	0.88631	1.88339	0.13307
H	2.00812	0.94062	0.76354
C	1.14029	2.95765	-0.81766
H	0.57111	3.85490	-0.55689
H	2.20013	3.20595	-0.79042
H	0.87397	2.66740	-1.83842
C	-3.50858	-1.62864	-1.14524
H	-3.38318	-2.66565	-1.46513
H	-4.52146	-1.50967	-0.75387
H	-3.40333	-0.98807	-2.03616

06:

E = -768.42547; G = -768.12245

C	-2.03019	1.71723	-0.20355
C	-3.08870	0.84751	0.49363
H	-2.03924	1.49211	-1.27794
H	-3.38289	1.30220	1.44468
C	-2.57529	-0.56912	0.77126
C	-0.59867	1.48840	0.23154
C	-0.22060	0.15307	0.82837
C	-1.34106	-0.45344	1.66304
H	-3.35225	-1.13601	1.30171
H	-1.02967	-1.42442	2.05398
H	-1.57641	0.17926	2.52289
H	0.66817	0.30868	1.44100
H	-2.28304	2.77573	-0.11350
H	-3.98271	0.80988	-0.13435
N	-2.24778	-1.24352	-0.50458
C	-3.30578	-2.14595	-0.92344
H	-4.25898	-1.61286	-0.94150
H	-3.10424	-2.51396	-1.93166
H	-3.41005	-3.01297	-0.24943
C	-0.93338	-1.87557	-0.52908
H	-0.82776	-2.66193	0.23857
H	-0.80871	-2.36386	-1.49909
C	0.15006	-0.80957	-0.36315
H	0.12670	-0.20857	-1.28103
C	1.52566	-1.39530	-0.21087
H	1.65949	-2.46011	-0.06538
C	2.62802	-0.63608	-0.25955
O	3.85881	-1.20457	-0.23981
O	2.68944	0.70107	-0.38405
C	4.83526	-0.54104	0.56328
H	4.46309	-0.41009	1.58317
H	5.70702	-1.19135	0.57155
H	5.09826	0.42886	0.14210
N	0.37466	2.26852	-0.03373
H	1.84503	1.19339	-0.21524
C	0.12416	3.52004	-0.73844
H	1.07485	4.01552	-0.92867
H	-0.37731	3.34823	-1.69666
H	-0.50046	4.19434	-0.14445

07:

E -768.42394; G= -768.11573

C	0.10082	0.74556	1.21764
C	1.63437	0.91796	1.21678

H	-0.16825	-0.25887	1.54283
H	1.87790	1.98257	1.27323
C	2.37668	0.35129	-0.02031
C	-0.55803	0.94146	-0.15099
C	0.23434	0.20673	-1.24726
C	1.65543	0.76084	-1.30094
H	3.38397	0.77976	-0.02237
H	2.19123	0.33977	-2.15491
H	1.64417	1.84803	-1.41211
H	-0.29099	0.40208	-2.18991
H	-0.36282	1.42664	1.93510
H	2.04045	0.46990	2.12844
N	2.55857	-1.10212	-0.06520
C	1.30001	-1.83428	-0.02898
H	1.51158	-2.88431	-0.25760
H	0.86732	-1.82969	0.98368
C	0.25995	-1.32253	-1.05198
H	0.55049	-1.73911	-2.02337
C	-1.11257	-1.80495	-0.68499
H	-1.37438	-2.85254	-0.74720
C	-2.05221	-0.95138	-0.27037
O	-3.31928	-1.35253	-0.03023
O	-1.90065	0.37730	-0.09093
C	-3.98797	-0.70680	1.04849
H	-4.17769	0.34270	0.82400
H	-4.92808	-1.23991	1.17289
H	-3.39754	-0.78139	1.96676
N	-0.79700	2.32346	-0.47146
H	-0.94842	2.44634	-1.46378
C	-0.07115	3.43135	0.11989
H	-0.13353	3.39587	1.20774
C	3.47508	-1.60359	0.93930
H	3.69075	-2.65561	0.73795
H	4.41484	-1.04859	0.89370
H	3.07814	-1.53513	1.96657
H	-0.55868	4.35583	-0.19382
H	0.98940	3.49818	-0.15957

08:

E = -768.44511; G = -768.13897

C	-0.75830	1.50241	-1.07867
C	-2.29538	1.41729	-0.93404
H	-0.45200	0.96318	-1.97703
H	-2.69552	2.37598	-0.58901
C	-2.73462	0.33613	0.04927
C	0.10058	0.99466	0.11091
C	-0.65427	0.47199	1.36875
C	-2.15367	0.69116	1.42045
H	-3.83038	0.32771	0.11923
H	-2.58445	0.07081	2.21170
H	-2.40341	1.73042	1.64972
H	-0.15651	0.81243	2.27924
H	-0.50111	2.54978	-1.24421
H	-2.73392	1.22647	-1.91731
N	-2.26116	-0.97789	-0.44228
C	-3.31872	-1.73623	-1.08270
H	-3.80463	-1.12216	-1.84431
H	-2.89890	-2.61649	-1.57494
H	-4.09042	-2.07277	-0.36918
C	-1.50687	-1.77040	0.51698
H	-2.12079	-2.08527	1.37976
H	-1.17570	-2.68334	0.01167
C	-0.29578	-0.98928	1.00626
H	0.22211	-1.53952	1.79277
C	0.63310	-0.50386	-0.13722
H	0.39436	-0.90524	-1.12055
C	2.07876	-0.70296	0.17545
O	2.77746	-1.18263	-0.86475
O	2.59331	-0.45373	1.24281
C	4.18374	-1.34188	-0.65184
H	4.36760	-2.03924	0.16519
H	4.58112	-1.73323	-1.58427
H	4.63930	-0.37894	-0.41677
N	1.08340	2.00266	0.46818
H	1.58902	1.68220	1.28814
C	2.05283	2.31601	-0.57967
H	2.85226	2.92132	-0.15151
H	2.50623	1.42650	-1.04141
H	1.58536	2.89671	-1.37627

09:

E = -768.46699; G = -768.16332

C	-1.53255	1.78276	-0.45955
C	-2.83235	1.16935	0.10076
H	-1.40656	1.51697	-1.51641
H	-3.22607	1.84301	0.86782
C	-2.68962	-0.21037	0.77389
C	-0.25194	1.37989	0.25102
C	-0.23278	0.06344	1.00064
C	-1.53713	-0.15125	1.77048
H	-3.62270	-0.40403	1.31245
H	-1.48328	-1.08514	2.33592
H	-1.70269	0.66515	2.47969
H	0.61787	0.09753	1.68258

H	-1.61887	2.87158	-0.44626
H	-3.58770	1.13432	-0.68801
N	-2.49748	-1.36531	-0.11271
C	-1.24566	-1.35270	-0.85994
H	-1.14540	-2.32192	-1.35992
H	-1.25911	-0.59389	-1.66226
C	-0.03134	-1.13552	0.04407
H	0.07202	-2.01837	0.68404
C	1.22324	-1.01197	-0.81481
H	1.30872	-1.88166	-1.47726
C	2.49757	-0.96461	-0.01051
O	3.50009	-0.42261	-0.71168
O	2.63543	-1.38853	1.10992
C	4.76964	-0.37370	-0.05176
H	5.09772	-1.37907	0.21220
H	5.45564	0.07620	-0.76396
H	4.70161	0.23459	0.85017
N	0.83291	2.04476	0.20710
H	1.19057	-0.12970	-1.45767
C	0.86962	3.28170	-0.56446
H	0.25400	4.05992	-0.10004
H	1.89602	3.64449	-0.60161
H	0.51479	3.14246	-1.59160
C	-3.62460	-1.59751	-0.99850
H	-4.55199	-1.60269	-0.42156
H	-3.71586	-0.84086	-1.79494
H	-3.51347	-2.57300	-1.47803

10:

E = -768.47078; G = -768.16545

C	-1.85598	1.69706	-0.08094
C	-2.97579	0.72663	0.20769
H	-3.69957	1.20873	0.87689
C	-2.52798	-0.60041	0.86247
C	-0.57668	1.47924	0.27284
C	-0.17101	0.16874	0.91851
C	-1.33051	-0.34863	1.77562
H	-3.36042	-0.98947	1.45564
H	-1.05037	-1.27994	2.27453
H	-1.58799	0.39142	2.53772
H	0.70386	0.35247	1.55137
H	-3.53383	0.50968	-0.71008
N	-2.16568	-1.67626	-0.07422
C	-1.04932	-1.31829	-0.93888
H	-0.80336	-2.18978	-1.55385
H	-1.31587	-0.49624	-1.62662
C	0.18208	-0.92062	-0.12490
H	0.50830	-1.80554	0.43406
C	1.30801	-0.51527	-1.09182
H	1.44694	-1.30379	-1.83440
C	2.60553	-0.31104	-0.35913
O	3.24875	-1.45979	-0.14772
O	3.02204	0.75388	0.03857
C	4.47238	-1.37166	0.59400
H	4.28447	-0.94951	1.58085
H	4.84005	-2.39022	0.67650
H	5.18896	-0.74762	0.06065
N	0.44396	2.41000	0.12884
H	1.04961	0.41280	-1.60612
C	0.23926	3.56264	-0.72111
H	-0.50240	4.23631	-0.28280
H	1.17837	4.10671	-0.81342
H	-0.11277	3.28255	-1.72415
C	-3.29017	-2.17151	-0.84931
H	-4.11721	-2.41685	-0.17949
H	-3.65582	-1.45549	-1.60254
H	-2.99296	-3.08209	-1.37498
H	-2.13830	2.63782	-0.54195
H	1.37713	2.01793	0.10274

11:

E = -749.05691; G = -748.79208

C	-1.50964	1.76913	-0.92113
C	-2.80057	1.28288	-0.23684
H	-3.20880	2.10673	0.35594
C	-2.62307	0.09437	0.73206
C	-0.23246	1.59726	-0.12093
C	-0.16966	0.46884	0.88932
C	-1.47494	0.40004	1.68823
H	-3.55080	0.00714	1.30562
H	-1.39687	-0.38353	2.44597
H	-1.66027	1.34846	2.20058
H	0.68157	0.67491	1.54071
H	-3.55291	1.05124	-0.99436
N	-2.39984	-1.22713	0.13289
C	-1.15160	-1.35894	-0.60854
H	-1.01914	-2.41781	-0.85383
H	-1.19598	-0.82892	-1.57581
C	0.06521	-0.89435	0.19592
H	0.22385	-1.61175	1.00705
C	1.30029	-0.89489	-0.70143
H	1.41456	-1.87550	-1.17786
C	2.58253	-0.63318	0.04956
O	3.54197	-0.18007	-0.76207

O	2.75263	-0.82759	1.22727
C	4.81346	0.06938	-0.15075
H	5.21082	-0.84905	0.28101
H	5.45850	0.42684	-0.94815
H	4.71208	0.82504	0.62784
H	1.21775	-0.16587	-1.51167
C	-3.52091	-1.68878	-0.66726
H	-4.44769	-1.58516	-0.09901
H	-3.63086	-1.13905	-1.61617
H	-3.38265	-2.74519	-0.90884
H	-1.33720	1.22081	-1.85449
H	-1.57966	2.82149	-1.20315
O	0.72675	2.31249	-0.32030

TS-12:
E = -768.38191; G = -768.08335
imaginary freq. 845.7 cm^{-1}

C	-1.70188	1.50166	-1.12799
C	-3.01616	1.01955	-0.49059
H	-1.41350	0.88106	-1.98794
H	-3.52949	1.88187	-0.05469
C	-2.85238	-0.01395	0.64203
C	-0.51664	1.54176	-0.20739
C	-0.53417	0.80916	0.99661
C	-1.85752	0.51423	1.66895
H	-3.82674	-0.12554	1.12655
H	-1.71725	-0.22716	2.45846
H	-2.24222	1.42636	2.13339
H	0.29214	0.97946	1.67821
H	-1.82879	2.50556	-1.54222
H	-3.67938	0.63045	-1.26665
N	-2.45340	-1.36878	0.24711
C	-1.17006	-1.46902	-0.43727
H	-0.93008	-2.53526	-0.51252
H	-1.23344	-1.10445	-1.47605
C	0.00954	-0.81203	0.26180
H	0.19280	-1.22013	1.25567
C	1.19036	-0.72132	-0.55140
H	1.16220	-1.08981	-1.57169
C	2.47550	-0.73033	0.11362
O	3.51059	-0.88025	-0.74692
O	2.65951	-0.58562	1.31143
C	4.81149	-0.82723	-0.16396
H	4.97841	0.14135	0.30976
H	4.93337	-1.61476	0.58023
H	5.51100	-0.97103	-0.98355
N	0.65762	1.88509	-0.78392

C	1.74794	2.38511	0.07364
H	1.97424	1.76610	0.94358
H	2.64721	2.44314	-0.54107
H	1.49586	3.39280	0.40576
C	-3.47847	-2.05373	-0.52420
H	-3.20938	-3.10707	-0.63031
H	-4.43450	-1.99475	-0.00035
H	-3.60910	-1.63705	-1.53603
H	1.03093	0.82836	-1.07003

TS-13:
E = -768.40604; G = -768.10470
imaginary freq. 486.4 cm^{-1}

C	-2.19779	1.61906	0.03137
C	-3.15024	0.61379	0.69000
H	-2.27761	1.54630	-1.05956
H	-3.41843	0.94304	1.69780
C	-2.49847	-0.77125	0.77869
C	-0.74980	1.40604	0.37718
C	-0.29275	0.26719	1.05070
C	-1.28667	-0.65458	1.70254
H	-3.21022	-1.48865	1.21174
H	-0.81961	-1.62854	1.87404
H	-1.62335	-0.29687	2.68078
H	0.68885	0.36104	1.49807
H	-2.47886	2.64479	0.29022
H	-4.07502	0.57211	0.10954
N	-2.11390	-1.19817	-0.58283
C	-3.16606	-1.99252	-1.19565
H	-4.11658	-1.45852	-1.13337
H	-2.94142	-2.16080	-2.25081
H	-3.29332	-2.97240	-0.70551
C	-0.81176	-1.84243	-0.69406
H	-0.70472	-2.71109	-0.02293
H	-0.73046	-2.23055	-1.71465
C	0.34204	-0.88315	-0.49873
H	0.34733	-0.05072	-1.20075
C	1.60021	-1.40551	-0.16977
H	1.71989	-2.41137	0.21231
C	2.70198	-0.51585	-0.13367
O	3.89761	-1.11775	0.12776
O	2.64816	0.71313	-0.28471
C	5.02200	-0.25239	0.23181
H	4.87147	0.49038	1.01677
H	5.86764	-0.89080	0.47824
H	5.20548	0.26258	-0.71251
N	0.14425	2.25019	-0.14279

H	1.12083	1.95885	-0.07934
C	-0.17530	3.43618	-0.91877
H	0.75839	3.89874	-1.23085
H	-0.75548	3.18875	-1.81086
H	-0.73779	4.15903	-0.32326

TS-14:
E = -768.40812; G = -768.10755
imaginary freq. 411.9 cm^{-1}

C	-1.82844	1.73780	-0.17484
C	-3.03993	0.91936	0.29591
H	-1.68585	1.62753	-1.25700
H	-3.48877	1.41189	1.16342
C	-2.70681	-0.52113	0.72847
C	-0.53683	1.36032	0.49391
C	-0.40910	0.21250	1.27361
C	-1.64208	-0.46579	1.81809
H	-3.62041	-0.94489	1.15704
H	-1.39123	-1.48117	2.13524
H	-2.03978	0.05927	2.69246
H	0.50520	0.12207	1.85549
H	-2.01210	2.80496	-0.01490
H	-3.80126	0.92128	-0.48711
N	-2.30818	-1.45915	-0.32986
C	-1.04255	-1.21465	-1.01968
H	-0.86478	-2.08790	-1.65793
H	-1.09905	-0.35215	-1.70591
C	0.17188	-1.06472	-0.13311
H	0.26050	-1.82957	0.63641
C	1.37444	-0.67011	-0.73526
H	1.39400	-0.19656	-1.70900
C	2.58866	-0.77907	-0.00416
O	3.68286	-0.35698	-0.71402
O	2.71758	-1.17412	1.15362
C	4.92865	-0.43595	-0.03241
H	4.91945	0.17444	0.87220
H	5.15932	-1.46701	0.24071
H	5.67472	-0.05938	-0.72910
N	0.56062	2.03619	0.14461
H	1.43707	1.71174	0.52967
C	0.62133	3.08757	-0.85903
H	-0.00546	3.93508	-0.57492
H	1.65127	3.42888	-0.93105
H	0.30511	2.72425	-1.83937
C	-3.36626	-1.67550	-1.30321
H	-3.12169	-2.54437	-1.91859
H	-4.30811	-1.87463	-0.78782
H	-3.51657	-0.81797	-1.97972

TS-15:
E = -768.41491; G = -768.11205
imaginary freq. 111.9 cm^{-1}

C	-1.73524	2.04042	0.12119
C	-2.87542	1.03256	-0.13884
H	-1.70243	2.79791	-0.66129
H	-3.81562	1.52638	0.10856
C	-2.75132	-0.30240	0.65649
C	-0.41911	1.36263	0.29571
C	-0.34666	0.11191	1.08986
C	-1.69520	-0.16414	1.75340
H	-3.71835	-0.52562	1.11229
H	-1.63683	-1.09301	2.32302
H	-1.96920	0.63615	2.44628
H	0.47306	0.20161	1.80671
H	-1.92207	2.56558	1.06682
H	-2.91019	0.81998	-1.20916
N	-2.40906	-1.46903	-0.16724
C	-1.11366	-1.34775	-0.82564
H	-0.92547	-2.27333	-1.37678
H	-1.10819	-0.53190	-1.57685
C	0.02888	-1.11932	0.16355
H	0.01648	-1.95075	0.88222
C	1.33629	-1.03240	-0.55604
H	1.37290	-1.29011	-1.60727
C	2.51932	-0.77433	0.11199
O	3.65804	-0.88148	-0.68566
O	2.65865	-0.40966	1.30451
C	4.87571	-0.49112	-0.07584
H	5.09427	-1.09486	0.80714
H	5.65002	-0.64435	-0.82665
H	4.85709	0.56099	0.22005
N	0.66937	1.81392	-0.23405
H	1.49051	1.21606	-0.11486
C	0.80551	2.99797	-1.08241
H	0.28204	2.85110	-2.02722
H	0.40701	3.87203	-0.57001
H	1.86340	3.14897	-1.27800
C	-3.45431	-1.82596	-1.11142
H	-4.40623	-1.92594	-0.58521
H	-3.58408	-1.09303	-1.92428
H	-3.21244	-2.78742	-1.56934

TS-16:

E = -768.38822; G = -768.08898
imaginary freq. 966.7 cm^{-1}

C	0.20306	1.04663	1.21772
C	1.66118	0.73086	1.58852
H	-0.25396	-0.07726	0.89260
H	2.09078	1.62641	2.04987
C	2.60632	0.34985	0.41810
C	-0.02112	1.59554	-0.07357
C	0.78284	0.96752	-1.14338
C	2.26874	1.21456	-0.79053
H	3.61954	0.58176	0.76973
H	2.91552	0.96758	-1.63328
H	2.42713	2.27088	-0.55890
H	0.54645	1.41863	-2.10871
H	-0.37409	1.46877	2.03660
H	1.67781	-0.04610	2.35609
N	2.61966	-1.04712	-0.02980
C	1.81668	-1.34633	-1.21630
C	0.46182	-0.62882	-1.24153
H	0.01831	-0.72993	-2.23740
C	-0.44494	-1.25489	-0.21201
H	-0.13400	-2.20442	0.20497
C	-1.83616	-1.03282	-0.29536
O	-2.59066	-1.95001	0.39933
O	-2.38983	-0.07511	-0.84630
C	-3.98338	-1.67723	0.46740
H	-4.17320	-0.71941	0.95602
H	-4.42981	-1.65787	-0.52843
H	-4.41884	-2.48451	1.05327
N	-0.97027	2.46330	-0.38388
H	-1.19878	2.53304	-1.36680
C	-2.00871	2.87906	0.54216
H	-1.56482	3.27909	1.45355
H	-2.60497	3.65688	0.07094
H	-2.64725	2.02260	0.78347
C	2.62740	-2.03994	1.02596
H	2.87152	-3.01648	0.60229
H	3.40942	-1.78650	1.74906
H	1.67865	-2.13133	1.57411
H	1.65690	-2.42519	-1.25483
H	2.38340	-1.08293	-2.11589

TS-17:
E = -768.41390; G = -768.10825
imaginary freq. 161.0 cm^{-1}

C	-2.60980	0.97909	0.30846
C	-2.97851	-0.42783	0.71697
H	-3.58574	-0.39216	1.62971
C	-1.77046	-1.35684	0.98054
C	-1.34032	1.40501	0.20580
C	-0.18398	0.48711	0.53575
C	-0.63175	-0.53783	1.57996
H	-2.08042	-2.12938	1.69004
H	0.18968	-1.19765	1.86595
H	-0.97784	-0.02025	2.47856
H	0.63606	1.09425	0.93294
H	-3.62885	-0.87590	-0.04346
N	-1.24506	-2.07825	-0.19210
C	-0.77802	-1.18572	-1.25072
H	-0.37268	-1.80465	-2.05742
H	-1.61318	-0.60658	-1.67778
C	0.30321	-0.22376	-0.75021
N	-0.96015	2.64970	-0.28974
C	-1.96968	3.66671	-0.50027
H	-2.56687	3.86266	0.40070
H	-1.48382	4.59054	-0.81121
H	-2.65121	3.35454	-1.29563
C	-2.17447	-3.06154	-0.72110
H	-2.52949	-3.70512	0.08672
H	-3.04985	-2.61367	-1.21833
H	-1.66029	-3.68541	-1.45615
H	-3.43096	1.64695	0.07005
H	-0.09754	2.99531	0.10805
H	0.45808	0.53887	-1.51861
C	1.63024	-0.95139	-0.53900
H	1.90874	-1.49150	-1.45135
H	1.57074	-1.70389	0.24859
C	2.77822	-0.01909	-0.24564
O	2.79787	1.16546	-0.47321
O	3.82124	-0.67287	0.28140
C	4.99162	0.11273	0.53388
H	4.76629	0.90969	1.24229
H	5.72237	-0.57359	0.95234
H	5.36252	0.54611	-0.39496

TS-18:
E = -768.41316; G = -768.10944
imaginary freq. 283.9 cm^{-1}

C	-0.66785	1.46750	-1.02740
C	-2.17511	1.63997	-0.76522
H	-0.53821	0.64696	-1.73172
H	-2.38028	2.60714	-0.29625
C	-2.69215	0.51479	0.13542
C	0.10213	1.10513	0.21648

C	-0.53306	0.26844	1.31518
C	-2.00296	0.64143	1.49606
H	-3.77547	0.62613	0.27403
H	-2.44979	-0.02273	2.23886
H	-2.11855	1.66454	1.86314
H	0.03103	0.43455	2.23425
H	-0.25204	2.36635	-1.48253
H	-2.69023	1.62529	-1.72863
N	-2.39725	-0.78285	-0.50925
C	-3.57051	-1.34783	-1.15050
H	-4.01683	-0.61159	-1.82300
H	-3.28560	-2.21915	-1.74405
H	-4.33883	-1.65981	-0.42223
C	-1.72080	-1.74604	0.35202
H	-2.35558	-2.04162	1.20522
H	-1.53819	-2.65145	-0.23365
C	-0.37710	-1.20362	0.85125
H	-0.08434	-1.80413	1.72108
C	0.69331	-1.24015	-0.22046
H	0.45901	-1.64812	-1.19608
C	2.01624	-1.01993	0.11590
O	2.91828	-1.24517	-0.91793
O	2.45385	-0.58150	1.20806
C	4.26016	-0.87217	-0.65909
H	4.66577	-1.40797	0.20098
H	4.82308	-1.13239	-1.55443
H	4.34797	0.20173	-0.47201
N	1.21625	1.71678	0.51345
H	1.72146	1.30458	1.30061
C	2.04657	2.44548	-0.44085
H	2.97467	2.71275	0.05814
H	2.26844	1.81087	-1.30317
H	1.54913	3.35596	-0.77164

19:
E = -768.46090; G = -768.15822

C	2.61711	-0.28600	-0.88474
C	3.02885	1.03655	-0.21578
H	1.91488	-0.05227	-1.69317
H	3.86987	0.86725	0.46496
C	1.86099	1.64440	0.56851
C	1.90293	-1.22183	0.06949
C	0.83073	-0.58201	0.93064
C	1.41439	0.64542	1.63748
H	2.19680	2.56619	1.06056
H	0.67693	1.08581	2.31275
H	2.27715	0.36068	2.24590
H	0.52995	-1.33398	1.66277
H	3.48277	-0.76766	-1.33714
H	3.36459	1.73727	-0.98510
N	0.76148	1.95965	-0.36923
C	0.65593	3.38305	-0.63396
H	1.62132	3.76946	-0.96835
H	-0.07296	3.55878	-1.42810
H	0.34468	3.95617	0.25609
C	-0.52040	1.36901	-0.01621
H	-0.92162	1.77791	0.93017
H	-1.23643	1.62581	-0.80018
C	-0.40978	-0.15694	0.08262
H	-0.30106	-0.54618	-0.93432
C	-1.68106	-0.75138	0.68281
H	-1.86148	-0.36532	1.69008
C	-2.91185	-0.51697	-0.15858
O	-4.03142	-0.62616	0.56576
O	-2.91649	-0.28956	-1.34264
C	-5.26120	-0.47818	-0.15381
H	-5.33760	-1.23797	-0.93145
H	-6.04973	-0.60498	0.58245
H	-5.31617	0.51190	-0.60617
N	2.10070	-2.47149	0.19772
H	-1.57875	-1.83828	0.77761
C	3.10948	-3.12438	-0.63231
H	3.10713	-4.19129	-0.41218
H	2.90710	-2.99564	-1.70026
H	4.11385	-2.73945	-0.42820

20:
E = -749.05365; G = -748.78819

C	-2.61288	1.54193	-0.18381
C	-3.06722	0.36014	0.69684
H	-3.42859	0.75825	1.64911
C	-1.96958	-0.66785	1.04545
C	-1.14009	1.91401	-0.11908
C	-0.14291	0.86074	0.33180
C	-0.72513	0.07945	1.51534
H	-2.35003	-1.27935	1.86905
H	0.01067	-0.62092	1.91490
H	-0.99239	0.76682	2.32362
H	0.76235	1.40286	0.61274
H	-3.92122	-0.13706	0.23147
N	-1.58830	-1.61442	-0.01153
C	-1.01141	-0.98022	-1.19249
H	-0.68558	-1.77363	-1.87222
H	-1.76416	-0.39662	-1.74535

C	0.19080	-0.09800	-0.83951
C	-2.66993	-2.50904	-0.38765
H	-3.09884	-2.96561	0.50677
H	-3.47751	-2.00346	-0.94132
H	-2.27663	-3.30415	-1.02514
H	-2.80787	1.33523	-1.24128
H	0.44735	0.50567	-1.71484
C	1.40289	-0.97085	-0.50267
H	1.60293	-1.65108	-1.33840
H	1.22572	-1.60025	0.36952
C	2.66908	-0.17627	-0.30440
O	2.90217	0.89610	-0.80385
O	3.54092	-0.81994	0.47956
C	4.80150	-0.16876	0.67992
H	4.65194	0.80291	1.15026
H	5.37261	-0.82459	1.33072
H	5.31188	-0.03583	-0.27380
H	-3.17848	2.44687	0.04729
O	-0.77268	3.01925	-0.45207

21:

E = -768.46748; G = -768.16322

C	-2.60980	0.97909	0.30846
C	-2.97851	-0.42783	0.71697
H	-3.58574	-0.39216	1.62971
C	-1.77046	-1.35684	0.98054
C	-1.34032	1.40501	0.20580
C	-0.18398	0.48711	0.53575
C	-0.63175	-0.53783	1.57996
H	-2.08042	-2.12938	1.69004
H	0.18968	-1.19765	1.86595
H	-0.97784	-0.02025	2.47856
H	0.63606	1.09425	0.93294
H	-3.62885	-0.87590	-0.04346
N	-1.24506	-2.07825	-0.19210
C	-0.77802	-1.18572	-1.25072
H	-0.37268	-1.80465	-2.05742
H	-1.61318	-0.60658	-1.67778
C	0.30321	-0.22376	-0.75021
N	-0.96015	2.64970	-0.28974
C	-1.96968	3.66671	-0.50027
H	-2.56687	3.86266	0.40070
H	-1.48382	4.59054	-0.81121
H	-2.65121	3.35454	-1.29563
C	-2.17447	-3.06154	-0.72110
H	-2.52949	-3.70512	0.08672
H	-3.04985	-2.61367	-1.21833
H	-1.66029	-3.68541	-1.45615
H	-3.43096	1.64695	0.07005
H	-0.09754	2.99531	0.10805
H	0.45808	0.53887	-1.51861
C	1.63024	-0.95139	-0.53900
H	1.90874	-1.49150	-1.45135
H	1.57074	-1.70389	0.24859
C	2.77822	-0.01909	-0.24564
O	2.79787	1.16546	-0.47321
O	3.82124	-0.67287	0.28140
C	4.99162	0.11273	0.53388
H	4.76629	0.90969	1.24229
H	5.72237	-0.57359	0.95234
H	5.36252	0.54611	-0.39496

TS-26:

E = -1550.07066; G =-1549.58536

imaginary freq. 397.1 cm^{-1}

C	2.03110	0.17000	-2.01490
C	3.48700	-0.31960	-2.03000
H	1.96610	1.21000	-1.67230
H	3.58890	-1.09220	-2.79790
C	3.97640	-0.94600	-0.70930
C	1.10860	-0.63970	-1.14170
C	1.60610	-1.49130	-0.16140
C	3.00720	-2.04290	-0.28470
H	4.94770	-1.40670	-0.91340
H	3.32750	-2.45100	0.67730
H	3.03850	-2.86250	-1.01040
H	0.89170	-2.11230	0.36840
H	1.62960	0.17030	-3.03210
H	4.14160	0.50120	-2.33150
N	4.21290	-0.03040	0.41260
C	3.05800	0.71410	0.89980
H	3.38790	1.24550	1.80030
H	2.74070	1.50020	0.19540
C	1.85440	-0.09820	1.32280
H	2.08480	-0.93280	1.98000
C	0.69620	0.63500	1.61400
H	0.57200	1.63530	1.21550
C	-0.37400	0.13520	2.38670
O	-0.22920	-1.16440	2.77160
O	-1.40680	0.75350	2.69880
C	-1.28200	-1.71070	3.56200
H	-1.40480	-1.14900	4.48870
H	-2.22460	-1.70270	3.01210
H	-0.98750	-2.73460	3.78070

N	-0.18460	-0.32850	-1.23730
H	-0.43750	0.41690	-1.88200
C	-1.30310	-1.11150	-0.71120
C	-1.47850	-2.41630	-1.49720
C	-2.58580	-0.26530	-0.77890
C	-2.66330	-3.22020	-0.96560
H	-1.64290	-2.16340	-2.55150
H	-0.55160	-2.99390	-1.44440
C	-3.78600	-1.07450	-0.28320
H	-2.75350	0.04170	-1.81510
N	-2.46760	0.93680	0.02980
C	-3.94480	-2.39080	-1.04250
H	-2.77020	-4.14700	-1.53370
H	-2.47270	-3.50260	0.07710
H	-4.68210	-0.45670	-0.37530
H	-3.64820	-1.28460	0.78540
H	-2.42780	0.76700	1.03460
C	-1.97900	2.13060	-0.36470
H	-4.17440	-2.17790	-2.09280
H	-4.79110	-2.94960	-0.63640
N	-1.69250	2.97260	0.64060
H	-1.67250	2.56660	1.57340
C	-1.11510	4.29200	0.46070
H	-1.10620	-1.34440	0.34150
C	5.31490	0.88530	0.15810
H	5.57750	1.40280	1.08340
H	6.18850	0.32520	-0.18100
H	5.07760	1.65080	-0.59820
S	-1.76660	2.57250	-2.00090
H	-1.76260	4.91440	-0.15640
H	-0.13310	4.23220	-0.01570
H	-1.01200	4.74690	1.44420

27:

E = -1550.0857; G = -1549.5980

C	1.90610	0.46820	-1.78980
C	3.28300	-0.17990	-2.03700
H	2.00140	1.36030	-1.15960
H	3.22040	-0.77770	-2.95050
C	3.77710	-1.11750	-0.91340
C	0.94930	-0.41620	-1.05630
C	1.47880	-1.29120	0.02140
C	2.66550	-2.08930	-0.54000
H	4.62240	-1.68190	-1.31800
H	3.01760	-2.77720	0.23170
H	2.36200	-2.67970	-1.40960
H	0.70280	-1.96620	0.37820
H	1.45930	0.80410	-2.72790
H	4.01370	0.60780	-2.23030
N	4.26320	-0.48090	0.31340
C	3.25700	0.30910	1.01700
H	3.66380	0.57580	1.99730
H	3.07450	1.26850	0.50340
C	1.93180	-0.42650	1.25510
H	2.11650	-1.18880	2.02060
C	0.88700	0.54790	1.71970
H	1.02040	1.60060	1.49510
C	-0.27090	0.19010	2.35550
O	-0.41470	-1.18540	2.55440
O	-1.23090	0.93550	2.73250
C	-1.48570	-1.58970	3.39300
H	-2.45160	-1.27630	2.99240
H	-1.44070	-2.67720	3.43580
H	-1.37750	-1.17780	4.39910
N	-0.30020	-0.35190	-1.36250
H	-0.56630	0.33060	-2.08110
C	-1.43370	-1.06810	-0.75060
C	-1.72380	-2.34370	-1.54150
C	-2.64640	-0.12060	-0.71630
C	-2.93890	-3.06350	-0.95770
H	-1.91450	-2.07690	-2.58760
H	-0.83760	-2.98440	-1.52490
C	-3.86420	-0.86190	-0.16030
H	-2.86430	0.22400	-1.73090
N	-2.36360	1.04110	0.10620
C	-4.15670	-2.13970	-0.94560
H	-3.14610	-3.96550	-1.53720
H	-2.71310	-3.38210	0.06690
H	-4.71880	-0.18250	-0.17880
H	-3.67440	-1.11460	0.89050
H	-2.27250	0.85730	1.11160
C	-1.75220	2.17560	-0.30710
H	-4.42660	-1.88050	-1.97570
H	-5.01750	-2.64940	-0.50770
S	-1.57400	2.60380	-1.95580
N	-1.31030	2.95630	0.68410
H	-1.27100	2.51120	1.60730
C	-0.56480	4.18350	0.47940
H	-1.14760	4.89630	-0.10360
H	0.37600	3.99300	-0.04460
H	-0.35280	4.61130	1.45790
H	-1.16250	-1.30810	0.28100
C	5.47310	0.29800	0.10980
H	5.88280	0.58930	1.07950
H	6.21910	-0.30560	-0.41160

H	5.30130	1.21870	-0.47100

TS-28:
E = -1550.06561; G = -1549.58041
imaginary freq. 1152.5 cm^{-1}

C	1.80260	1.25200	-1.32880
C	3.09950	0.72830	-1.97580
H	2.00250	1.63560	-0.32140
H	2.96160	0.72340	-3.06050
C	3.48950	-0.70420	-1.55690
C	0.75830	0.19930	-1.13400
C	1.18470	-1.16890	-0.70990
C	2.27680	-1.61400	-1.70390
H	4.26840	-1.03980	-2.24810
H	2.54850	-2.64600	-1.47260
H	1.90090	-1.58410	-2.73050
H	0.34110	-1.85200	-0.76790
H	1.38990	2.08810	-1.89680
H	3.90820	1.43060	-1.76630
N	4.04250	-0.87180	-0.21080
C	3.11810	-0.55400	0.87130
H	3.57060	-0.89920	1.80650
H	2.98780	0.53540	0.99260
C	1.75600	-1.24250	0.73760
H	1.93850	-2.31770	0.86810
C	0.82820	-0.71460	1.82290
H	1.36410	-0.46560	2.74240
C	-0.35320	-1.46360	2.16180
O	-0.66060	-2.46270	1.27650
O	-1.11200	-1.23070	3.09890
C	-1.81790	-3.24080	1.57800
H	-2.71040	-2.61640	1.62920
H	-1.91100	-3.96270	0.76950
H	-1.69410	-3.75610	2.53110
N	-0.46870	0.51640	-1.37750
H	-0.63270	1.48340	-1.67530
C	-1.70390	-0.23780	-1.10120
C	-2.34730	-0.73590	-2.39290
C	-2.64350	0.69400	-0.30740
C	-3.65100	-1.46560	-2.06340
H	-2.55140	0.11940	-3.04770
H	-1.64850	-1.39330	-2.91680
C	-3.95020	-0.03250	-0.00360
H	-2.85900	1.57800	-0.91420
N	-1.99970	1.14700	0.91430
C	-4.60440	-0.56040	-1.28180
H	-4.12120	-1.81030	-2.98660
H	-3.42210	-2.35700	-1.46700
H	-4.61470	0.65630	0.52230
H	-3.74640	-0.86630	0.67900
H	-1.85060	0.42260	1.61370
C	-1.02450	2.12860	0.92690
H	-4.89780	0.28640	-1.91260
H	-5.51840	-1.10240	-1.03020
S	-1.16550	3.51800	-0.08160
N	-0.02550	1.87060	1.74260
H	0.32410	0.67920	1.75140
C	1.01530	2.84020	2.01590
H	0.63620	3.65820	2.63410
H	1.42860	3.28000	1.10290
H	1.81120	2.33160	2.56490
H	-1.44150	-1.08230	-0.46060
C	5.31100	-0.18810	-0.02260
H	5.76600	-0.52330	0.91220
H	5.98820	-0.43400	-0.84310
H	5.20810	0.90780	0.02900

29:
E = -1550.095245; G = -1549.60649

C	1.84860	1.34160	-1.18950
C	3.17870	0.87860	-1.80970
H	1.99860	1.70350	-0.16450
H	3.08650	0.92910	-2.89800
C	3.58110	-0.56500	-1.45230
C	0.81680	0.26060	-1.07040
C	1.25220	-1.14090	-0.75700
C	2.40120	-1.49100	-1.72210
H	4.40470	-0.84450	-2.11550
H	2.68810	-2.53050	-1.55190
H	2.07280	-1.39300	-2.76010
H	0.42320	-1.83070	-0.91530
H	1.42870	2.18520	-1.73980
H	3.96440	1.58260	-1.53070
N	4.06710	-0.79630	-0.08850
C	3.07980	-0.58210	0.96140
H	3.49840	-0.97070	1.89510
H	2.89080	0.48930	1.14450
C	1.76650	-1.32220	0.69620
H	1.97630	-2.39250	0.77530
C	0.74520	-0.93710	1.76500
H	1.21430	-1.01750	2.75320
C	-0.49760	-1.78760	1.85100
O	-0.36760	-2.98150	1.27070
O	-1.51500	-1.43640	2.40190

C	-1.50100	-3.85760	1.36750
H	-2.37610	-3.39130	0.91460
H	-1.22320	-4.75740	0.82670
H	-1.70870	-4.08280	2.41280
N	-0.41430	0.58950	-1.24620
H	-0.60380	1.59740	-1.41510
C	-1.63150	-0.19810	-1.00400
C	-2.22350	-0.72450	-2.30990
C	-2.63270	0.70810	-0.25110
C	-3.49410	-1.52130	-2.00750
H	-2.45550	0.12640	-2.96080
H	-1.48480	-1.34280	-2.82730
C	-3.88540	-0.11040	0.05770
H	-2.90390	1.53150	-0.91790
N	-2.07960	1.28690	0.95570
C	-4.50230	-0.68060	-1.22140
H	-3.93570	-1.87430	-2.94180
H	-3.22770	-2.41130	-1.42530
H	-4.59760	0.53400	0.57760
H	-3.62070	-0.92290	0.74680
H	-1.90750	0.61760	1.70110
C	-1.15090	2.34110	0.97350
H	-4.84650	0.14760	-1.85140
H	-5.38110	-1.28080	-0.97490
S	-1.20580	3.51490	-0.34380
N	-0.35440	2.35590	1.98490
H	0.42310	0.10950	1.68320
C	0.56140	3.46870	2.13850
H	0.03440	4.41940	2.28400
H	1.21000	3.59910	1.26350
H	1.19020	3.29000	3.01290
H	-1.37120	-1.03870	-0.35730
C	5.29850	-0.08290	0.20970
H	5.71440	-0.45850	1.14720
H	6.02680	-0.25700	-0.58480
H	5.15280	1.00340	0.31810

30:

E = -1550.1192; G = -1549.63158

C	1.75900	0.97200	-1.76620
C	3.11620	0.43440	-2.26260
H	1.90950	1.82940	-1.10430
H	2.94310	-0.13640	-3.18090
C	3.83810	-0.51260	-1.28170
C	0.94110	-0.06610	-1.02590
C	1.71680	-0.92360	-0.03890
C	2.86730	-1.59020	-0.80990
H	4.65840	-0.98890	-1.82750
H	3.38660	-2.29630	-0.15680
H	2.47370	-2.14260	-1.66800
H	1.06810	-1.70090	0.36590
H	1.16050	1.32960	-2.60520
H	3.76480	1.26980	-2.53810
N	4.45320	0.10300	-0.09880
C	3.50890	0.74680	0.80540
H	4.04170	0.97930	1.73390
H	3.16550	1.71600	0.40880
C	2.31070	-0.14470	1.16000
H	2.67530	-0.91100	1.85060
C	1.27130	0.71070	1.88460
H	0.83960	1.46450	1.21840
C	0.11630	-0.03080	2.50420
O	0.41030	-1.27420	2.85920
O	-0.97970	0.46000	2.68710
C	-0.64510	-2.03440	3.46880
H	-1.47540	-2.14300	2.77020
H	-0.21110	-3.00300	3.69800
H	-0.98850	-1.53850	4.37550
N	-0.28840	-0.17430	-1.33400
C	-1.16610	-1.16620	-0.73100
C	-1.12690	-2.45300	-1.56340
C	-2.59030	-0.58560	-0.68370
C	-2.13920	-3.48210	-1.06350
H	-1.34430	-2.18990	-2.60520
H	-0.11050	-2.85800	-1.54020
C	-3.60440	-1.62550	-0.20710
H	-2.85500	-0.23280	-1.68210
N	-2.60660	0.56540	0.20790
C	-3.54970	-2.89250	-1.05990
H	-2.09990	-4.37670	-1.68980
H	-1.87260	-3.79110	-0.04530
H	-4.60160	-1.17880	-0.22770
H	-3.38910	-1.88190	0.83880
H	-2.28920	0.36420	1.15100
C	-2.56880	1.86840	-0.14680
H	-3.84170	-2.65000	-2.08850
H	-4.27050	-3.62370	-0.68630
S	-3.08060	2.45330	-1.65830
N	-2.13490	2.69430	0.82320
H	-1.76160	2.26930	1.66470
C	-2.12090	4.14210	0.72660
H	-3.12380	4.52980	0.54130
H	-1.46630	4.47600	-0.08030
H	-1.75370	4.53460	1.67330
H	-0.88570	-1.41420	0.30560

C	5.54260	1.00770	-0.42420
H	6.08010	1.27200	0.48960
H	6.24110	0.51460	-1.10350
H	5.20110	1.94370	-0.89490
H	1.74990	1.25780	2.70600

31:
E = -1550.1211; G = -1549.63245

C	1.82600	-0.19960	-2.13830
C	3.32420	-0.34820	-2.21550
H	3.56620	-1.08220	-2.99370
C	3.98560	-0.80150	-0.89350
C	1.10140	-0.45310	-1.04020
C	1.77270	-0.94100	0.22590
C	3.03320	-1.72950	-0.14520
H	4.90510	-1.34070	-1.13750
H	3.52000	-2.10830	0.75690
H	2.76720	-2.58030	-0.77870
H	1.09380	-1.59380	0.77920
H	3.77610	0.59060	-2.55530
N	4.38460	0.27880	0.02210
C	3.26090	1.08560	0.48280
H	3.64380	1.81290	1.20590
H	2.81040	1.66030	-0.34670
C	2.18580	0.22900	1.15400
H	2.62940	-0.22100	2.04820
C	1.03470	1.13690	1.58700
H	0.54280	1.59950	0.72780
C	-0.05260	0.49870	2.40970
O	0.30400	-0.64300	2.98630
O	-1.15600	0.98440	2.56610
C	-0.68970	-1.28880	3.79580
H	-1.56160	-1.53690	3.18930
H	-0.21820	-2.19160	4.17250
H	-0.98720	-0.63700	4.61610
N	-0.27820	-0.17780	-0.96690
H	-0.62340	0.21220	-1.84010
C	-1.19630	-1.20150	-0.46290
C	-1.06040	-2.55840	-1.16080
C	-2.63690	-0.67790	-0.59470
C	-2.05590	-3.57250	-0.59760
H	-1.23700	-2.41410	-2.23460
H	-0.03240	-2.91860	-1.05260
C	-3.63730	-1.69040	-0.03900
H	-2.85080	-0.50260	-1.65230
N	-2.78520	0.59960	0.09130
C	-3.48770	-3.05000	-0.72280
H	-1.95170	-4.53030	-1.11290
H	-1.82770	-3.75230	0.46030
H	-4.64730	-1.29480	-0.16950
H	-3.46940	-1.80130	1.04030
H	-2.58150	0.55110	1.08550
C	-2.54200	1.80800	-0.47650
H	-3.74200	-2.94750	-1.78420
H	-4.19580	-3.76210	-0.29230
S	-2.72190	2.11060	-2.14250
N	-2.19480	2.77580	0.38400
H	-1.93530	2.48770	1.32200
C	-1.96860	4.16040	0.01230
H	-2.86350	4.58920	-0.43980
H	-1.14490	4.24770	-0.69900
H	-1.72460	4.71230	0.91810
H	-1.00270	-1.34600	0.61030
C	5.44660	1.11830	-0.50640
H	5.82450	1.76580	0.28830
H	6.26920	0.49180	-0.85810
H	5.12370	1.76480	-1.33790
H	1.42660	1.95260	2.20730
H	1.31620	0.13880	-3.03810

TS-32:
E = -1550.06253; G = -1549.57813
imaginary freq. 411.6 cm^{-1}

C	-1.13000	-2.18530	0.34680
C	-2.57340	-2.69030	0.51170
H	-0.79190	-1.65240	1.24550
H	-2.69460	-3.60030	-0.08330
C	-3.67710	-1.71770	0.04930
C	-0.93540	-1.24530	-0.81020
C	-2.01440	-0.65030	-1.44980
C	-3.38450	-1.27370	-1.37810
H	-4.61490	-2.28160	0.04840
H	-4.13850	-0.54710	-1.69160
H	-3.46100	-2.13210	-2.05370
H	-1.78350	-0.06440	-2.33590
H	-0.45440	-3.03900	0.24500
H	-2.73350	-2.98130	1.55230
N	-3.93680	-0.54530	0.89270
C	-2.84210	0.40720	1.05090
H	-3.25030	1.25730	1.61060
H	-2.02890	0.00400	1.67880
C	-2.24800	0.97990	-0.21700
H	-2.97090	1.34990	-0.93970
C	-1.05730	1.69270	-0.06330

H	-0.46640	1.54250	0.83020
C	-0.46990	2.50150	-1.06650
O	-1.18060	2.55940	-2.22540
O	0.60790	3.11290	-0.97570
C	-0.63450	3.37390	-3.26030
H	-1.33260	3.30340	-4.09110
H	-0.54520	4.40990	-2.93140
H	0.34790	3.00940	-3.56320
N	0.30160	-0.79770	-1.06330
H	0.39240	-0.18880	-1.86880
C	-4.45570	-0.91060	2.20260
H	-4.83580	-0.01810	2.70480
H	-5.27960	-1.61770	2.08870
H	-3.69560	-1.36610	2.85780
C	1.55130	-1.35430	-0.55000
C	2.64540	-0.27170	-0.58190
C	1.98800	-2.56120	-1.39340
C	4.00670	-0.82830	-0.14850
H	2.74520	0.03670	-1.63080
N	2.28620	0.96070	0.11330
C	3.33170	-3.11920	-0.92890
H	2.06370	-2.24090	-2.44010
H	1.20790	-3.32620	-1.35070
C	4.40700	-2.03570	-0.99560
H	4.74480	-0.02740	-0.23870
H	3.95780	-1.11420	0.90400
C	2.01130	1.14670	1.42130
H	2.02030	1.73860	-0.48570
H	3.60910	-3.97770	-1.54510
H	3.24080	-3.47950	0.10250
H	4.54410	-1.72720	-2.03900
H	5.36690	-2.42520	-0.64840
N	1.67530	2.41550	1.73270
H	1.45290	3.02120	0.94610
C	1.18710	2.82430	3.03670
H	1.39490	-1.65400	0.48660
S	2.05350	-0.07090	2.60970
H	0.26680	2.29680	3.30420
H	1.93380	2.62510	3.80480
H	0.99160	3.89440	2.99620

33:

E = -1550.0789; G = -1549.58993

C	-1.13000	-2.18530	0.34680
C	-2.57340	-2.69030	0.51170
H	-0.79190	-1.65240	1.24550
H	-2.69460	-3.60030	-0.08330
C	-3.67710	-1.71770	0.04930
C	-0.93540	-1.24530	-0.81020
C	-2.01440	-0.65030	-1.44980
C	-3.38450	-1.27370	-1.37810
H	-4.61490	-2.28160	0.04840
H	-4.13850	-0.54710	-1.69160
H	-3.46100	-2.13210	-2.05370
H	-1.78350	-0.06440	-2.33590
H	-0.45440	-3.03900	0.24500
H	-2.73350	-2.98130	1.55230
N	-3.93680	-0.54530	0.89270
C	-2.84210	0.40720	1.05090
H	-3.25030	1.25730	1.61060
H	-2.02890	0.00400	1.67880
C	-2.24800	0.97990	-0.21700
H	-2.97090	1.34990	-0.93970
C	-1.05730	1.69270	-0.06330
H	-0.46640	1.54250	0.83020
C	-0.46990	2.50150	-1.06650
O	-1.18060	2.55940	-2.22540
O	0.60790	3.11290	-0.97570
C	-0.63450	3.37390	-3.26030
H	-1.33260	3.30340	-4.09110
H	-0.54520	4.40990	-2.93140
H	0.34790	3.00940	-3.56320
N	0.30160	-0.79770	-1.06330
H	0.39240	-0.18880	-1.86880
C	-4.45570	-0.91060	2.20260
H	-4.83580	-0.01810	2.70480
H	-5.27960	-1.61770	2.08870
H	-3.69560	-1.36610	2.85780
C	1.55130	-1.35430	-0.55000
C	2.64540	-0.27170	-0.58190
C	1.98800	-2.56120	-1.39340
C	4.00670	-0.82830	-0.14850
H	2.74520	0.03670	-1.63080
N	2.28620	0.96070	0.11330
C	3.33170	-3.11920	-0.92890
H	2.06370	-2.24090	-2.44010
H	1.20790	-3.32620	-1.35070
C	4.40700	-2.03570	-0.99560
H	4.74480	-0.02740	-0.23870
H	3.95780	-1.11420	0.90400
C	2.01130	1.14670	1.42130
H	2.02030	1.73860	-0.48570
H	3.60910	-3.97770	-1.54510
H	3.24080	-3.47950	0.10250
H	4.54410	-1.72720	-2.03900

H	5.36690	-2.42520	-0.64840
N	1.67530	2.41550	1.73270
H	1.45290	3.02120	0.94610
C	1.18710	2.82430	3.03670
H	1.39490	-1.65400	0.48660
S	2.05350	-0.07090	2.60970
H	0.26680	2.29680	3.30420
H	1.93380	2.62510	3.80480
H	0.99160	3.89440	2.99620

TS-34:
E = -1550.061117; G = -1549.57656
imaginary freq. 1248.1 cm^{-1}

C	-0.96160	-1.65810	-0.76580
C	-2.32150	-2.12100	-1.33420
H	-0.97180	-1.66110	0.33200
H	-2.13570	-2.61050	-2.29360
C	-3.36600	-1.01460	-1.60020
C	-0.56930	-0.26740	-1.14670
C	-1.62900	0.75760	-1.42200
C	-2.69250	0.13270	-2.33640
H	-4.13320	-1.45250	-2.24510
H	-3.42700	0.89870	-2.59310
H	-2.23580	-0.22540	-3.26330
H	-1.16500	1.60630	-1.92090
H	-0.17930	-2.36270	-1.04870
H	-2.72900	-2.88640	-0.67160
N	-4.07240	-0.46810	-0.43920
C	-3.19090	0.19120	0.51470
H	-3.81460	0.66870	1.27710
H	-2.56710	-0.54190	1.05210
C	-2.29720	1.27050	-0.11450
H	-2.96390	2.06820	-0.46960
C	-1.36280	1.77990	0.97910
H	-1.92620	2.06290	1.87110
C	-0.32200	2.74920	0.77940
O	0.13220	2.87960	-0.51710
O	0.25520	3.36360	1.66920
C	1.19300	3.82020	-0.71210
H	0.87080	4.82240	-0.43070
H	2.06860	3.54990	-0.12020
H	1.42870	3.78650	-1.77400
N	0.65990	0.11030	-1.23960
H	0.78340	1.10660	-1.43520
C	-4.90810	-1.45070	0.23200
H	-5.54050	-0.94550	0.96520
H	-5.55330	-1.94570	-0.49690
H	-4.32660	-2.22070	0.76420
C	1.90750	-0.62590	-0.99100
C	2.67570	0.11530	0.12260
C	2.72090	-0.70160	-2.28310
C	4.03820	-0.54290	0.35950
H	2.87090	1.13070	-0.24740
N	1.86640	0.30660	1.32290
C	4.07950	-1.35370	-2.02270
H	2.86920	0.31370	-2.67140
H	2.15280	-1.25830	-3.03240
C	4.84640	-0.59740	-0.93730
H	4.56230	0.03120	1.12740
H	3.88220	-1.55000	0.75060
C	1.05530	-0.64600	1.91760
H	1.52130	1.25060	1.45600
H	4.65200	-1.38150	-2.95230
H	3.92810	-2.39160	-1.70540
H	5.05710	0.42090	-1.28590
H	5.81020	-1.07650	-0.75150
S	1.52930	-2.29390	2.03140
N	-0.07290	-0.15150	2.38570
H	-0.62830	0.76110	1.68660
C	-0.96690	-0.95360	3.19350
H	-0.47830	-1.27950	4.11560
H	-1.31320	-1.85370	2.67110
H	-1.83310	-0.33940	3.45010
H	1.65700	-1.62290	-0.63200

35:
E = -1550.08907; G = -1549.60025

C	-0.43130	-1.70480	-1.09700
C	-1.66530	-2.37440	-1.72820
H	-0.35430	-1.94220	-0.02710
H	-1.43880	-2.59970	-2.77360
C	-2.95070	-1.52640	-1.72900
C	-0.40690	-0.21040	-1.15110
C	-1.68840	0.57470	-1.30250
C	-2.61340	-0.15000	-2.28960
H	-3.65830	-2.01830	-2.40270
H	-3.51990	0.44480	-2.41770
H	-2.13290	-0.25160	-3.26590
H	-1.43350	1.56350	-1.68850
H	0.48010	-2.10760	-1.53930
H	-1.83060	-3.33380	-1.23510
N	-3.65360	-1.37260	-0.45090
C	-2.92850	-0.61360	0.56280
H	-3.61040	-0.44100	1.40100

H	-2.07010	-1.16990	0.97510
C	-2.45510	0.73910	0.03270
H	-3.34190	1.32930	-0.22340
C	-1.67150	1.46860	1.13680
H	-2.32580	1.60110	1.99970
C	-1.17120	2.81410	0.68980
O	-1.92060	3.81800	1.12530
O	-0.19720	2.98760	-0.01770
C	-1.52440	5.13600	0.71420
H	-0.52320	5.35500	1.08330
H	-1.54070	5.20840	-0.37260
H	-2.25210	5.81040	1.15500
N	0.69060	0.45720	-1.04800
H	0.58310	1.47690	-0.97940
C	-4.12300	-2.63690	0.09410
H	-4.81150	-2.44070	0.91900
H	-4.65930	-3.19260	-0.67790
H	-3.30870	-3.27100	0.47880
C	2.07930	-0.02240	-0.94910
C	2.70450	0.54050	0.34340
C	2.83800	0.43360	-2.19830
C	4.19430	0.18520	0.40860
H	2.63970	1.63480	0.27680
N	1.96290	0.19200	1.55270
C	4.31570	0.05640	-2.09750
H	2.74470	1.52250	-2.29650
H	2.37490	-0.01500	-3.08110
C	4.93880	0.65950	-0.83900
H	4.60580	0.64490	1.31060
H	4.29260	-0.89660	0.51740
C	1.37580	-1.06360	1.81950
H	1.33000	0.92570	1.84660
H	4.83900	0.39790	-2.99350
H	4.41020	-1.03500	-2.06400
H	4.89710	1.75370	-0.90520
H	5.99370	0.38420	-0.76840
S	2.24530	-2.52210	1.40220
N	0.22690	-0.99390	2.40990
H	-0.82300	0.85280	1.45190
C	-0.39700	-2.22030	2.85950
H	0.23570	-2.77180	3.56580
H	-0.61080	-2.91320	2.03300
H	-1.34100	-1.97730	3.35290
H	2.07220	-1.10830	-0.87660

36:

E = -1550.1200; G = -1549.63269

C	-0.84970	-2.14770	0.37750
C	-2.21070	-2.87210	0.35720
H	-0.68280	-1.66820	1.34860
H	-2.14090	-3.71730	-0.33450
C	-3.41080	-2.03050	-0.11720
C	-0.66860	-1.07380	-0.68730
C	-1.91310	-0.38150	-1.21170
C	-3.04930	-1.37840	-1.44660
H	-4.24750	-2.71960	-0.27000
H	-3.91780	-0.85820	-1.85940
H	-2.74170	-2.14630	-2.16240
H	-1.63180	0.10880	-2.14520
H	-0.05880	-2.89290	0.27900
H	-2.40520	-3.30150	1.34300
N	-3.90840	-0.99990	0.80380
C	-2.97720	0.09350	1.05760
H	-3.51810	0.87050	1.60830
H	-2.14600	-0.22050	1.71340
C	-2.42100	0.70310	-0.23210
H	-3.24440	1.21240	-0.74260
C	-1.35370	1.73120	0.13740
H	-1.77480	2.46610	0.83540
C	-0.76720	2.53270	-0.99680
O	-1.58640	2.66200	-2.03340
O	0.32720	3.05790	-0.96300
C	-1.09820	3.43590	-3.13860
H	-0.89430	4.45740	-2.81930
H	-0.18820	2.98600	-3.53450
H	-1.89030	3.41640	-3.88140
N	0.45030	-0.66230	-1.13410
C	-4.40560	-1.54820	2.05430
H	-4.94670	-0.77190	2.60070
H	-5.09570	-2.36930	1.84830
H	-3.60410	-1.92390	2.71040
C	1.69620	-1.23100	-0.63210
C	2.75120	-0.11590	-0.59650
C	2.17480	-2.35060	-1.56390
C	4.11910	-0.63920	-0.15480
H	2.85010	0.24520	-1.62640
N	2.30920	1.07290	0.13910
C	3.54000	-2.88830	-1.13400
H	2.23480	-1.95060	-2.58350
H	1.42710	-3.14930	-1.57760
C	4.57720	-1.76530	-1.08440
H	4.83370	0.18830	-0.16390
H	4.05330	-1.00800	0.87110
C	1.96480	1.19930	1.43610
H	2.01080	1.83870	-0.45360

H	3.86330	-3.67540	-1.82000
H	3.45260	-3.34390	-0.14050
H	4.72510	-1.36690	-2.09560
H	5.54270	-2.15210	-0.74920
S	2.06950	-0.03750	2.59890
N	1.50600	2.42580	1.76910
H	1.29580	3.05470	1.00240
C	0.94070	2.75780	3.06360
H	1.67100	2.59150	3.85490
H	0.05290	2.15640	3.28020
H	0.66700	3.81160	3.04460
H	-0.51690	1.25480	0.65250
H	1.59500	-1.62370	0.38280

37:

E = -1550.1177; G = -1549.63030

C	-0.98500	-2.23970	0.14650
C	-2.35000	-2.75190	0.54220
H	-2.44890	-3.78840	0.19750
C	-3.53930	-1.94040	-0.02360
C	-0.79830	-1.17800	-0.65300
C	-1.98440	-0.41650	-1.20460
C	-3.16500	-1.37230	-1.38930
H	-4.39440	-2.61380	-0.12890
H	-4.02160	-0.84230	-1.81380
H	-2.88570	-2.18240	-2.06810
H	-1.70660	0.01590	-2.17120
H	-0.13540	-2.79460	0.53110
H	-2.42810	-2.80420	1.63430
N	-4.00690	-0.82670	0.81740
C	-2.97820	0.17910	1.04610
H	-3.42710	0.99750	1.61850
H	-2.14940	-0.22460	1.65650
C	-2.41840	0.73560	-0.26480
H	-3.22410	1.27370	-0.77470
C	-1.30080	1.71870	0.08190
H	-1.68970	2.49140	0.75760
C	-0.66480	2.46390	-1.06240
O	-1.43270	2.53850	-2.14400
O	0.42890	2.99020	-1.00760
C	-0.89790	3.26680	-3.25860
H	-0.71760	4.30360	-2.97700
H	0.03380	2.80970	-3.59110
H	-1.65310	3.20550	-4.03670
N	0.43600	-0.61010	-0.98450
C	-4.59680	-1.26010	2.07290
H	-5.09090	-0.41110	2.55130
H	-5.34720	-2.03000	1.88060
H	-3.86140	-1.66330	2.78690
C	1.68930	-1.22040	-0.57640
C	2.79860	-0.15400	-0.56340
C	2.10890	-2.39390	-1.47050
C	4.14410	-0.74240	-0.12840
H	2.91670	0.19250	-1.59820
N	2.43120	1.06330	0.16500
C	3.44560	-2.98990	-1.02850
H	2.19440	-2.02930	-2.50280
H	1.31780	-3.14840	-1.46010
C	4.53570	-1.91740	-1.02610
H	4.90030	0.04610	-0.16850
H	4.07030	-1.07660	0.90870
C	2.03270	1.19320	1.44900
H	2.13430	1.82310	-0.43620
H	3.72460	-3.81840	-1.68450
H	3.34150	-3.40260	-0.01780
H	4.69070	-1.55990	-2.05150
H	5.48640	-2.33700	-0.68780
S	2.12660	-0.02640	2.62930
N	1.54630	2.41530	1.75290
H	1.33290	3.02520	0.97160
C	0.96200	2.76190	3.03490
H	1.68550	2.61880	3.83700
H	0.08020	2.15170	3.25120
H	0.67420	3.81130	2.99500
H	-0.49310	1.21040	0.61210
H	1.56220	-1.57200	0.44900
H	0.45990	-0.23050	-1.92520
